# On aggregation invariance of multinomial processing tree models

**DOI:** 10.3758/s13428-024-02497-y

**Published:** 2024-10-14

**Authors:** Edgar Erdfelder, Julian Quevedo Pütter, Martin Schnuerch

**Affiliations:** 1 Department of Psychology, School of Social Sciences, University of Mannheim, Room B 118, A5, 68159 Mannheim, Germany; 2 Department of Psychology, School of Social Sciences, University of Mannheim, Room 518, L 13, 15, 68161 Mannheim, Germany

**Keywords:** Multinomial processing tree (MPT) modeling, Data aggregation, Aggregation invariance, Robustness

## Abstract

Multinomial processing tree (MPT) models are prominent and frequently used tools to model and measure cognitive processes underlying responses in many experimental paradigms. Although MPT models typically refer to cognitive processes within single individuals, they have often been applied to group data aggregated across individuals. We investigate the conditions under which MPT analyses of aggregate data make sense. After introducing the notions of structural and empirical aggregation invariance of MPT models, we show that any MPT model that holds at the level of single individuals must also hold at the aggregate level when it is both structurally and empirically aggregation invariant. Moreover, group-level parameters of aggregation-invariant MPT models are equivalent to the expected values (i.e., means) of the corresponding individual parameters. To investigate the robustness of MPT results for aggregate data when one or both invariance conditions are violated, we additionally performed a series of simulation studies, systematically manipulating (1) the sample sizes in different trees of the model, (2) model parameterization, (3) means and variances of crucial model parameters, and (4) their correlations with other parameters of the respective MPT model. Overall, our results show that MPT parameter estimates based on aggregate data are trustworthy under rather general conditions, provided that a few preconditions are met.

**Supplementary Information:**

The online version contains supplementary material available at 10.3758/s13428-024-02497-y.

## Introduction

Quantitative psychological laws typically refer to single individuals. However, empirical tests of these laws have often used data aggregated across individuals. Not surprisingly, there is a long-standing debate in experimental psychology whether tests of universal laws based on aggregated data make sense at all and, if so, under which conditions they are meaningful. In mathematical learning psychology, this debate dates back to the 1950s (see, e.g., Bakan, 1954; Estes, 1956; Hayes, 1953; Sidman, 1952). It soon became apparent that many functions hypothesized to describe individual learning processes across trials (or developmental growth processes across time) are not aggregation invariant, that is, the mean curve for a group of individuals and the curve that applies to single individuals are not of the same type. This holds, for example, for exponential functions (e.g., Anderson and Tweney, 1981; Sidman, 1952; Murre, 2023; Murre and Chessa, 2011) or for Gompertz functions (e.g., Tanner, 1970). Just as the mean of exponential functions with different parameters is not an exponential function, the average of several Gompertz functions is not of the Gompertz type. Linear functions, in contrast, are aggregation invariant: When all individuals follow a linear law, then the data averaged across individuals will also conform to a linear function, with intercept and slope for the aggregate data obtained by taking the expectation of the individual intercepts and slopes, respectively. For example, Sternberg’s law of memory scanning is aggregation invariant. According to this law, the response time $T_n$ of individual *n* required to identify a target in short-term memory (STM) depends linearly on the set size *M* of items currently maintained in STM (Sternberg, 1966). Hence, $T_n = a_n + b_n \cdot M + F_n$, where $a_n$ and $b_n$ are, respectively, the intercept and the slope of person *n*, while $F_n$ denotes an independent and identically distributed (i.i.d) random error component with conditional expectation $E(F_n | M) = 0$ for each individual and set size. Although $a_n$ and $b_n$ may vary between individuals and should thus be seen as realizations of random variables *A* and *B*, respectively, testing the law based on response time data aggregated across individuals is meaningful. According to elementary rules of expectations (see, e.g., Appendix B in Hays, 1973), a linear law is implied also for the aggregate means: $E(T | M) = E(A) + E(B) \cdot M$. Similar arguments can be made for some nonlinear functions that can, however, be transformed into linear functions. This applies, for example, to logarithmic or power laws that become linear after transforming one or both variables involved logarithmically.

Following the pioneering work of Sidman (1952), others have continued to work out criteria for deciding whether specific quantitative laws are aggregation invariant or not, and how to test quantitative laws in an efficient and valid way when they fail to be aggregation invariant (e.g., Bakan, 1954; Estes, 1956; Hayes, 1953; Murre, 2023; Murre and Chessa, 2011). Surprisingly, however, the issue of aggregation invariance has largely been neglected in the cognitive modeling literature that developed in the past five decades. Formalized as parameterized statistical models, cognitive models typically apply to single individuals, just as universal quantitative laws do. If we evaluate such models based on group data, we encounter virtually the same methodological problem already discussed by Sidman: When a parameterized statistical model holds for single individuals, does it then also hold for data aggregated across individuals? Or conversely: When a model fits aggregate data quite nicely, is it possible nevertheless that the same model does not apply to the data of any single participant (e.g., Ashby et al., 1994)?

Of course, cognitive modelers have always been cognizant of the fact that aggregation across individuals is problematic and may foster biased results whenever individuals differ in their parameters (see, e.g., Chechile, 2009; Cohen et al., 2008; Estes and Maddox, 2005; Riefer and Batchelder, 1991; Rouder and Batchelder, 1998). However, rather than scrutinizing analytically for which model types and which model parameters between-participant heterogeneity may cause troubles and for which others it might be harmless, researchers have typically engaged in Monte Carlo simulations to identify model features for which a certain degree of parameter heterogeneity can be considered as “still tolerable”. The typical result of these studies is that aggregation results are robust and quite valid for small degrees of heterogeneity, at least when models are sufficiently simple (Chechile, 2009; Cohen et al., 2008; Estes & Maddox, 2005; Riefer & Batchelder, 1991; Rouder & Batchelder, 1998). In such cases, aggregate analyses can even be considered advantageous compared to single-participant analyses because the latter are often plagued by unreliable and systematically biased parameter estimates as a consequence of scarce data per participant. For larger degrees of parameter heterogeneity, however, aggregate analyses may cause more serious biases for some parameters at least, so that they cannot be recommended for general use (Chechile, 2009; Cohen et al., 2008; Estes & Maddox, 2005; Riefer & Batchelder, 1991; Rouder & Batchelder, 1998; Siegler, 1987).

In this paper, we address the issue of aggregation invariance for a prominent and frequently used class of statistical models in the cognitive modeling literature, namely, multinomial processing tree (MPT) models (for an introduction and tutorial, see Schmidt et al., 2023). MPT models have been very influential in different subdisciplines of psychology, particularly in various branches of cognitive psychology and social cognition research (for reviews, see Batchelder and Riefer, 1999; Erdfelder et al., 2009; Erdfelder et al., 2020; Hütter and Klauer, 2016). By far the most applications of MPT models so far made use of maximum likelihood (ML) estimation for aggregated data, assuming homogeneity of parameters across participants and items or negligible parameter variability that does not systematically bias the results (Chechile, 2009; Riefer & Batchelder, 1991; Rouder & Batchelder, 1998). Although extensions to hierarchical MPT models have become available in the past 15 years that allow for parameter heterogeneity between participants^1^ (Klauer, 2006, 2010; Lee et al., 2020; Nestler & Erdfelder, 2023; Smith & Batchelder, 2010), such models have often been used as comparison standards only to show that their group-level mean parameters produce results very similar to those obtained with ML estimates for aggregated data.

In sum, in most MPT model applications, it is typically taken for granted that MPT models are (approximately) aggregation invariant. The goal of the present research is to investigate this assumption critically and in detail, thereby fostering deeper insights in the conditions under which MPT models are indeed perfectly aggregation invariant and in alternative conditions under which they are not. In addition, we investigate whether there are simple ways to transform MPT models that violate aggregation invariance into psychologically meaningful MPT models that do not. We also suggest experimental design techniques that attenuate consequences of aggregation invariance violations. Finally, using Monte Carlo simulations, we assess how serious the consequences of such violations are under various context conditions.

Importantly, by focusing on aggregation invariance properties of MPT models, we do not mean to imply that aggregation across individuals is the default method that should generally be preferred in MPT analyses. Quite to the contrary, hierarchical MPT models have important advantages such as automatically accounting for parameter variability in model tests. The latent-trait model (Klauer, 2010; Heck et al., 2018; Nestler & Erdfelder, 2023), in particular, allows more than just estimating group-level mean parameters, for example, assessing correlations among parameters and explaining variability in parameters as a function of covariates. Despite these advantages, however, almost all MPT model applications until 2010 (cf., Erdfelder et al., 2009) and even most applications thereafter were based on aggregated data (e.g., Hütter & Klauer, 2016). Moreover, when there are very few responses (or just a single response) per participant (see Klauer et al., 2007, for an example), there is no alternative to data aggregation (Schmidt et al., 2023). Because researchers employing MPT models are typically interested in group-level mean parameters in the first place, it is of considerable interest to scrutinize (1) how well MPT parameters of aggregated data approximate mean parameters and (2) which properties of the MPT model and the data affect approximation accuracy. These questions motivate our current research.

Our article is organized as follows: In the next section, we introduce MPT models conceptually and then more formally. We begin with a description of the pair-clustering model (Batchelder & Riefer, 1980, 1986). This MPT model serves as our running example throughout this article. It is ideally suited not only to illustrate possible problems with aggregation invariance but also to suggest solutions to these problems. After introducing the pair-clustering model, we will outline and explain a prerequisite for investigating aggregation invariance, namely, the formal model equation structure that applies to any MPT model. In Section 3, building on this general model equation structure, we define the concepts of (1) structural aggregation invariance and (2) empirical aggregation invariance and show analytically that any MPT model that satisfies properties (1) and (2) must necessarily be aggregation invariant, that is, it must apply to aggregate data whenever it holds for any single participant. Based on this result, we will also suggest methods to eliminate, or at least attenuate, problems caused by violations of structural aggregation invariance. This is followed by an extensive Monte Carlo simulation experiment in Section 4. In this section, based on the hypotheses that can be derived from Section 3, we test predictions and assess consequences of violations of structural and empirical aggregation invariance under a variety of context conditions. In the Appendix, we additionally analyze the consequences of structural and empirical aggregation invariance for group-level mean parameters of the major classes of hierarchical MPT models proposed so far. Finally, in the Discussion (Section 3), we outline implications of our analytical and simulation results for empirical applications of MPT models, emphasizing the conditions under which MPT parameter estimation results based on aggregated data are trustworthy in general versus potentially problematic.

## MPT models

MPT models are models for categorical data that typically originate from discrete responses of participants in cognitive paradigms or judgment tasks. A simple example would be *yes* or *no* responses in recognition or detection tests. Response frequencies across the *J* possible response categories are assumed to follow a binomial (if $J=2$) or a multinomial distribution (if $J>2$) for each condition observed. If more than one condition is observed (e.g., separate observations for targets and lures in a recognition test), independence of observations in different conditions is assumed in addition, hence implying a joint multinomial distribution of response counts across conditions in the general case.

Rather than just describing the frequency data, MPT models aim at explaining the observed response distribution in terms of latent psychological processes underlying different responses. Each relevant psychological process *s*, $s = 1,..., S$, is assumed to result in a specific cognitive or affective state with latent probability $\theta _s$ or $1 - \theta _s$, respectively, depending on whether this process is successful or not. In a recognition task, for example, $\theta _1 = D$ could represent the probability that the recognition process is successful. Analogously, $(1-\theta _1) = (1-D)$ would denote the probability of an unsuccessful recognition attempt so that an additional guessing process is required to determine a response. Multinomial processing tree models describe the possible combinations of such latent process outcomes that may give rise to different responses by proposing a processing tree structure (hence the name). Often, several process sequences in a processing tree may result in the same response. To illustrate, a *yes* response to a target item in the recognition test – a so-called *hit* – may either result from recognizing the item with probability *D* or from not recognizing the item followed by guessing that the item was old with probability $(1-D) \cdot g$. Since both processing routes are disjoint, the total probability of a *hit* is just the sum of the two branch probabilities, $p(hit) = D + (1-D) \cdot g$. In this way, all response probabilities are reparameterized as functions of *S* latent parameters $\theta _s$, each of which is an element of the interval admissible for probabilities, that is, the unit interval [0, 1].

Empirical applications of MPT models aim at testing the goodness-of-fit of a proposed model (or the selection among several candidate models, cf. Heck et al., 2014) and to estimate the parameters $\theta _s$, $s = 1,..., S$, given observed response frequencies for the selected cognitive paradigm. Since the parameters typically represent outcomes of cognitive processes, we may conceive MPT models as statistical tools that enable “the measurement of cognitive processes” (Riefer & Batchelder, 1988). A prominent and quite typical example of the MPT model class is the pair-clustering model we introduce next.

**Fig. 1:**
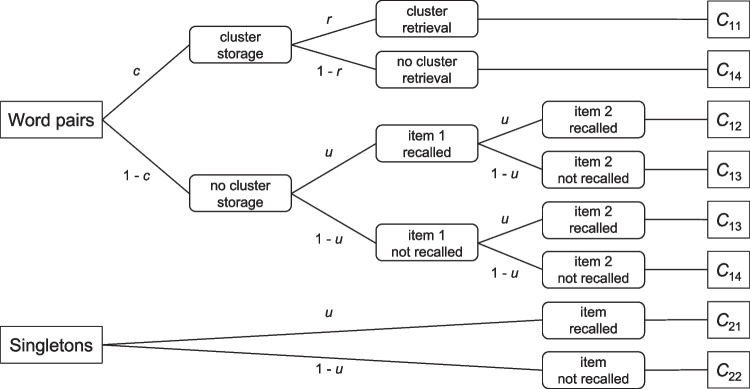
The pair-clustering multinomial processing tree (MPT) model. $C_{11}$ = Words of a pair recalled adjacently; $C_{12}$ = Words of a pair recalled nonadjacently; $C_{13}$ = One word of pair recalled; $C_{14}$ = None of the two words recalled; $C_{21}$ = Singleton recalled; $C_{22}$ = Singleton not recalled; *c* = probability that a word pair is stored as a cluster; *r* = probability that a previously stored cluster is successfully retrieved; *u* = probability that a single word is stored and retrieved (modified from Fig. 2 of Riefer & Batchelder, 1988)

### Running example: The pair-clustering model

The pair-clustering model was one of the first MPT models proposed in the psychological literature (Batchelder & Riefer, 1980, 1986). It aims at explaining free recall of semantically related word pairs and unrelated single items in terms of three basic memory processes, namely, (1) storage of a word pair as a cluster in memory, (2) retrieval of a stored cluster from memory, and (3) single item storage and retrieval. To estimate the outcome probabilities of these three processes, Batchelder and Riefer proposed a free recall paradigm in which participants learn a list of words composed of $m_1$ pairs of semantically related words (e.g., chair, table) and $m_2$ single unrelated words. Only one word is presented at a time, with a fixed lag between words belonging to the same pair (to ensure similar cluster storage probabilities for different word pairs) but otherwise in a randomized sequence. Thus, there are $2 \cdot m_1 + m_2$ words in the to-be-learned list in total, typically embedded in a primacy and a recency buffer of about four words at the beginning and the end of the list, respectively, to absorb primacy and recency effects in free recall. All buffer items are excluded from data analyses.

Following a retention interval, participants are asked to recall all items they can remember in any order. Based on the free recall protocol, word pairs are then assigned to one of four possible response categories: both words are recalled adjacently ($C_{11}$), both words are recalled nonadjacently ($C_{12}$), only one word of the pair is recalled ($C_{13}$), and neither word is recalled ($C_{14}$). Singletons are just scored as recalled ($C_{21}$) versus not recalled ($C_{22}$).

As outlined in the previous section, MPT models can be illustrated as processing trees in which the root node represents the beginning of a processing sequence for each item type or condition. Hence, there are two processing trees for word pairs and singletons in the pair-clustering model. The leaf nodes of each tree represent the observable response categories in which each processing sequence (i.e., each branch in a tree) terminates. The intermediate nodes represent outcomes of latent cognitive processes or mental states. Thus, each branch in a tree describes a possible sequence of latent cognitive processes leading to a specific observable response in the task.

Figure 1 illustrates the latent processes proposed in the pair-clustering MPT model. The model assumes three latent states: With probability *c*, a word pair is stored as a cluster. With probability *r*, a pair previously stored as a cluster is successfully retrieved as a cluster. With probability *u*, a single word is stored and retrieved. If a word pair was not stored as a cluster, storage and retrieval of any of its single components is assumed to be equivalent to storage and retrieval of a singleton. Therefore, parameter *u* appears in both trees. Note that the pair-clustering model makes several simplifying assumptions, such as the all-or-none nature of cluster retrieval and conditional independence of single word storage and retrieval for unclustered words of a pair.

Based on the processing tree diagrams in Fig. 1, the response probabilities $p(C_{kj})$ for the two trees and the six categories of the pair-clustering model can be rewritten as functions of the latent process probabilities as outlined next.

### General model equation structure of MPT models

As illustrated in the previous section, MPT models aim at explaining probabilities of observable responses as a function of probabilities of latent states that represent outcomes of underlying cognitive processes. In the pair-clustering model, the relevant processes are cluster storage, cluster retrieval, and single word storage plus retrieval, and the latent probabilities represent success (*c*, *r*, and *u*, respectively) versus failure of these three processes ($(1-c)$, $(1-r)$, and $(1-u)$, respectively). In general, MPT models include *K* trees with $J_{k}$ response categories $C_{kj}$ in tree *k*, $k = 1,..., K$, $j = 1,..., J_{k}$, and $S \le \sum _{k=1}^{K}(J_{k}-1)$ parameters $\theta _{s}$, $s = 1,..., S$, each of which is an element of [0, 1]. In the pair-clustering model, for example, $K = 2$, $J_{1} = 4$, $J_{2} = 2$, and $S = 3$, and the three parameters are $\theta _{1} = c$, $\theta _{2} = r$ and $\theta _{3} = u$, respectively. The general structure of the MPT model equation^2^ that describes how response probabilities $p(C_{kj})$ depend on the *S* parameters collected in the parameter vector $\varvec{\theta }$ is given by

1 $$\begin{aligned} p(C_{kj} | \varvec{\theta } ) = \sum _{i = 1}^{I_{kj}} \prod _{s = 1}^{S} \theta _{s}^{a_{skji}}( 1 -\theta _{s})^{b_{skji}} . \end{aligned}$$

Here, $I_{kj}$ denotes the number of branches that terminate in category $C_{kj}$, while $a_{skji}$ and $b_{skji}$ are count variables (i.e., integers) that indicate how often a process parameter $\theta _{s}$ and its complement $(1-\theta _{s})$, respectively, appear in the *i*-th branch that terminates in category $C_{kj}$ (see Hu & Batchelder, 1994). For instance, there is only a single branch that terminates in category $C_{11}$ of the pair-clustering model (i.e., the first branch in the first tree). This branch is characterized by successful cluster storage with probability $\theta _{1} = c$ followed by successful cluster retrieval with conditional probability $\theta _{2} = r$ and has thus an overall probability of $c \cdot r$. Hence, $a_{1111} = a_{2111} = 1$, whereas all other $a_{s111}$ and $b_{s111}$ counts are zero for this branch. In contrast, in the single branch that terminates in category $C_{12}$ (third branch in Fig. 1), parameter $\theta _{3} = u$ occurs twice next to $(1-\theta _{1}) = (1-c)$ (i.e., the branch probability is $(1-c) \cdot u^{2}$). For this branch, $b_{1121} = 1$ and $a_{3121} = 2$, whereas all other count variables are zero. The $a_{skji}$ and $b_{skji}$ counts for the other branches of both trees can be derived accordingly.

The general notation introduced in Eq. 1 has a number of advantages. It allows for a simple description of different MPT models for the same response category system and for an analysis of different models with the same general software tool. In essence, for a given paradigm and category system, differences between MPT models boil down to different matrices of $a_{skji}$ and $b_{skji}$ count variables. More importantly in the present context, the general notation also enables us to analyze consequences of aggregating data across individuals when an MPT model holds for each individual, but parameters may vary between individuals.

## Aggregation invariance of MPT models

The general model equation structure shown in Eq. 1 refers to a single participant with a participant-specific parameter vector $\varvec{\theta }$. When we assume that the same model (defined by the $a_{skji}$ and $b_{skji}$ count variables) holds for each sampled individual, but participants may differ in their parameter values, the parameter vector $\varvec{\theta }$ becomes a random vector $\varvec{\Theta }$, with $\varvec{\theta }_{n}$ being the realization of this random vector for participant $n, n = 1,..., N$. By implication, the response category probabilities $p(C_{kj})$ predicted by the model also become random variables $P(C_{kj})$ with the general structure

2 $$\begin{aligned} P(C_{kj} | \varvec{\Theta } ) = \sum _{i = 1}^{I_{kj}} \prod _{s = 1}^{S} \Theta _{s}^{a_{skji}}( 1 -\Theta _{s})^{b_{skji}}. \end{aligned}$$

We can now analyze whether the expected frequencies $N_{k} \cdot \textit{E}(P(C_{kj} | \varvec{\Theta }))$ in tree $k, k = 1,..., K$, for data aggregated across participants follow the same MPT model supposed to hold for each participant.

### Structural and empirical aggregation invariance

To facilitate our analysis, we first introduce two important concepts, namely, (1) structural aggregation invariance and (2) empirical aggregation invariance of MPT models.

#### Definition 1

**(Structural aggregation invariance of MPT models).** We call MPT models *structurally aggregation invariant* (SAI), when (1) all count variables $a_{skji}$ and $b_{skji}$ of this model are either 0 or 1 and, in addition, (2) $(a_{skji} = 1) \Rightarrow (b_{skji} = 0)$ as well as $(b_{skji} = 1) \Rightarrow (a_{skji} = 0)$ for all *s*, *k*, *j*, and *i*.

Definition 1 entails that, in MPT models with the SAI property, parameters or their complements never occur more than once in a branch. Furthermore, a parameter and its complement never co-occur in the same branch. Note that the pair-clustering model is not structurally aggregation invariant because the last four branches for word pairs violate this definition (see the upper tree in Fig. 1). Also note that the same model can be easily transformed in a structurally aggregation invariant model version by introducing a fourth parameter *a* that applies to single word storage plus retrieval. For example, parameter *a* could represent successful storage and retrieval of the second word of an unclustered pair in the first tree and, in addition, successful storage and retrieval of a singleton in the second tree. Parameter *u* would remain for the first word of a pair so that no parameter occurs repeatedly in any branch. In the current case, such a model extension leads to a saturated model (with four parameters and four independent model equations) which is still identified. In other MPT models, analogous transformations may lead to overparameterization such that the resulting SAI model is no longer identifiable. Hence, it may not always be possible to transform a model that violates structural aggregation invariance in an identifiable SAI model.

#### Definition 2

**(Empirical aggregation invariance of MPT models).** For all *s*, *k*, *j*, and *i*, we define $$x_{skji} = 1$$ if $$(a_{skji} + b_{skji}) > 0$$ and $$x_{skji} = 0 $$ otherwise. Also, for all *k*, *j*, and *i*, let $$F_{\varvec{\Theta }^{\mathbf {(kji)}}} (\varvec{\theta ^{(kji)}})$$ denote the joint cumulative distribution function of the subset of random variables $$\Theta _{s}$$ for which $$x _{skji} = 1$$ (i.e., only those parameters that co-occur in the *i*-th branch terminating in category $$C_{kj}$$). Correspondingly, $$F_{\Theta _{s}} (\theta _{s})$$ denotes the cumulative distribution function of the random variable $$\Theta _{s}$$. We call MPT models *empirically aggregation invariant* (EAI), when $$F_{\varvec{\Theta }^{\mathbf {(kji)}}} (\varvec{\theta ^{(kji)}}) = \prod _{s = 1}^{S} F_{\Theta _{s}} (\theta _{s})^{x_{skji}}$$ holds for all vectors of parameter values $$\varvec{\theta ^{(kji)}}$$ and all tree branches *i* that terminate in any response category $$C_{kj}$$.

Definition 2 requires that, in MPT models with the EAI property, all parameters and parameter complements that co-occur in any tree branch are mutually independent. Whether this is the case is ultimately an empirical question that depends on the data at hand. Hence, a specific MPT model can have the EAI property for one data set but not for another. The SAI property, in contrast, is a fixed structural property that either does or does not hold for an MPT model, irrespective of the data considered.

#### Observation 1

**(Aggregation invariance of MPT models).** Consider a structurally aggregation invariant MPT model that holds for each sampled individual with parameters that may vary between individuals (cf., Eq. 2). Also assume that parameters satisfy the conditions of empirical aggregation invariance. Then the same model must hold for the response frequencies aggregated across participants, and the *S* parameters of the aggregate model correspond to the expectation of the parameter vector $\textit{E}(\varvec{\Theta })$.

Intuitively, Observation 1 describes what one would hope to get from an analysis of aggregated data, namely, that such an analysis reflects the same model which also holds for individuals, but with estimated parameters that correspond to the group-level means of the individual parameters. Observation 1 states that this result must hold whenever the SAI and EAI properties both hold. Interestingly, in this case the variances of parameters across participants do not matter at all. Even if individuals are extremely heterogeneous, aggregation invariance will still hold. Also note that the distribution of $\varvec{\Theta }$ is not constrained in any way. It can be any distribution on the parameter space $[0, 1]^{S}$, even a complex mixture distribution.

The proof of Observation 1 is straightforward (see pp. 230-233 in Erdfelder, 2000). Using well-known rules of expectations (see, e.g., Appendix B in Hays, 1973, Rules 5–7), we see that the following must hold for the expected frequencies of the aggregate data:

3 $$\begin{aligned} N_{k} \cdot E(P(C_{kj} | \varvec{\Theta }))&= N_{k} \cdot E{(\sum _{i = 1}^{I_{kj}} \prod _{s = 1}^{S} \Theta _{s}^{a_{skji}}(1 -\Theta _{s})^{b_{skji}})} \end{aligned}$$

4 $$\begin{aligned}&= N_{k} \cdot (\sum _{i = 1}^{I_{kj}} \prod _{s = 1}^{S} (E(\Theta _{s}))^{a_{skji}}(1-E(\Theta _{s}))^{b_{skji}}). \end{aligned}$$

To derive (4) from (3), we make use of two standard rules of expectations: First, the expectation of a sum of random variables is always equal to the sum of their expected values. Second, the expectation of a product of random variables equals the product of their expected values if these random variables are mutually independent (which they are because of the EAI property). By combining these rules with the fact that all exponents $a_{skji}$ and $b_{skji}$ must be either 0 or 1 (because of the SAI property), (4) follows.

Although very useful when combined with the SAI property according to Definition 1, the concept of EAI as introduced in Definition 2 has the disadvantage that it is difficult to assess empirically. Fortunately, a weaker definition exists that is easier to assess empirically (see the Appendix, Corollary 2) and suffices to derive Observation 1 in most practically relevant cases.

#### Definition 3

**(Weak empirical aggregation invariance of MPT models).** We call MPT models *weakly empirically aggregation invariant* (WEAI), when $\textrm{Cov}(\Theta _{s}, \Theta _{s'}) = 0$ holds for all parameter pairs $\Theta _{s}$ and $\Theta _{s'}$ with (1) $a_{skji} > 0$ and $a_{s'kji} > 0$, (2) $a_{skji} > 0$ and $b_{s'kji} > 0$, (3) $b_{skji} > 0$ and $a_{s'kji} > 0$, or (4) $b_{skji} > 0$ and $b_{s'kji} > 0$ for at least one branch *i* terminating in at least one response category $C_{kj}$.

Definition 3 entails that, in MPT models with the WEAI property, parameters or their complements need to be uncorrelated if they co-occur in the same tree branch. Note that WEAI is only a necessary but not a sufficient condition for EAI according to Definition 2 and therefore does not guarantee aggregation invariance in general (see the Appendix, Table 1, for an example of a three-parameter MPT model that satisfies SAI and WEAI but violates EAI and is therefore inconsistent with Observation 1). This notwithstanding, WEAI suffices to derive Observation 1 under either of two conditions that cover many practically relevant applications. First, when no more than two parameters occur in any branch of an MPT model – as is the case, for example, in the pair-clustering model – aggregation invariance is implied for all models that satisfy SAI and WEAI. Second, if we focus on distributions of the MPT parameter vector $\varvec{\Theta }$ that exclude higher-order dependencies and allow for bivariate dependencies (if any) only – for example, on multivariate normal distributions of probit-transformed parameters as assumed in the latent-trait model of Klauer (2010) – then WEAI combined with SAI also suffices to derive Observation 1, irrespective of the number of parameters that occur in the same tree branch (see the Appendix for implications concerning hierarchical MPT models).

To illustrate Definition 3, we return to our pair-clustering example with three parameters *c*, *r*, and *u*. We see that parameters *c* and *r* co-occur in the first branch whereas *c* and *u* appear in branches three to six of the first processing tree. Hence, $\textrm{Cov}(\Theta _{1}, \Theta _{2}) = \textrm{Cov}(\Theta _{1}, \Theta _{3}) = 0$ must hold for the pair-clustering model to be WEAI. In contrast, since parameters *r* und *u* never co-occur in any branch of any tree, the covariance $\textrm{Cov}(\Theta _{2}, \Theta _{3})$ can be of any size. This will not affect WEAI (or EAI) in any way. Note that we need not consider covariances involving complements of parameters separately because such covariances may only differ in sign and not in absolute value from the covariances of the corresponding parameters.

Importantly, violations of SAI would prevent the same conclusion because in general $\textit{E}(\Theta _{s}^{a_{skji}}) \ne (\textit{E}(\Theta _{s}))^{a_{skji}}$ when $a_{skji} > 1$. Similarly, violations of WEAI would lead to discrepancies between Eq. (3) and Eq. (4). The degree of such discrepancies (hence, the bias introduced by aggregation) will depend on the size of the parameter correlations that conflict with WEAI. It will also depend on the variances of model parameters, but only on those involved in violations of SAI or WEAI. If these variances approach zero (i.e., if variability between participants in crucial parameters is small), then violations of SAI or WEAI may be negligible and aggregation invariance will hold approximately. Conversely, the larger the variances of crucial parameters, the larger the bias introduced by aggregation.

### Factors that affect possible aggregation bias

Observation 1 along with Definitions 1 to 3 provides us with a powerful framework to derive predictions concerning the robustness of MPT analyses based on aggregated data. The probably most important prediction refers to MPT models and associated data preselected to be in line with the SAI and EAI properties. For such model-data combinations, Observation 1 applies. Hence, the aggregate model must be valid when the respective MPT model holds at the individual level, and the aggregate model provides us with parameters that represent the means of the corresponding individual parameters.

What happens if Observation 1 does not apply? Consider the pair-clustering model as an example. Since no more than two parameters occur in any branch of this model, only deviations from SAI and WEAI need to be considered. Hence, deviations from aggregation invariance depend on a subset of variances and correlations of the random variables *C*, *R*, and *U* that comprise the individual *c*, *r*, and *u* parameters, respectively. Specifically, just as the variance of *U*, *Var*(*U*), is crucial (i.e., the smaller this variance, the more the model behaves like a SAI model) so are the two correlations between *C* and *R* as well as *C* and *U* (i.e., the smaller these two correlations, the less violation of WEAI). The standard three-parameter pair-clustering model is completely aggregation invariant model if and only if (1) $U = u$ is a constant that does not vary between participants and (2) the correlation between *C* and *R* is zero, irrespective of all other parameter variances and correlations.^3^

Another interesting prediction refers to MPT models that violate SAI but can be transformed in SAI-conform models by including additional parameters. Extending the pair-clustering model to a model with four parameters *c*, *r*, *a*, and *u* as outlined above should essentially remedy aggregation bias due to SAI violation. Similar remedies might apply to other MPT models that violate SAI, provided they have $S < \sum _{k=1}^{K}(J_{k}-1)$ parameters so that the model does not become overparametrized when including an additional parameter.

A different remedy might apply when a parameter occurs in different trees of an MPT model but produces SAI violation only in one of these trees. An example is the *u* parameter in the standard three-parameter pair-clustering model: It violates SAI only for the word-pair tree but not for the singleton tree (see Fig. 1). Clearly, the proportion of word pairs in the study list should moderate the degree of possible aggregation bias: The larger the proportion of word pairs (hence, the smaller the proportion of singletons), the stronger the overall impact of SAI violation. By implication, the impact of SAI violation is largest when only word pairs enter the estimation of *c*, *r*, and *u*. In contrast, by including a large proportion of singletons into the estimation of the same three parameters, aggregation bias should diminish to some degree.

All predictions considered so far refer to the population level, that is, to the true parameters underlying a sample of data. If sample data are used to estimate these parameters using asymptotically unbiased procedures such as ML estimation, basically the same results should hold, provided the number *M* of responses per participant and the number *N* of participants in the sample is sufficiently large. Since many estimators (including ML) are not necessarily unbiased for small samples, results might look different if, say, $N = 1$ and $M = 16$ applies. For the pair-clustering model, this corresponds to estimation for a single participant based on $m_1= 8$ word pairs and $m_2 = 8$ singletons presented in the learning phase. As already shown by Riefer and Batchelder (1991), the ML estimate of *r* tends to have a positive bias in small samples. Hence, the mean estimate of *r* would overestimate the true *r* systematically in such cases. Notably, this happens because of small-sample estimation bias, not as a consequence of aggregation bias induced by parameter heterogeneity. This instructive example shows that it is in general not a good idea to avoid data aggregation across participants and analyze single-participant data instead, followed by averaging the individual parameter estimates across participants. Because the individual estimates may suffer from systematic estimation bias *in the same direction* if *M* is small, this estimation bias will also compromise the estimates averaged across participants. This holds even if *N* is very large.

A final prediction concerns the behavior of the $G^2$ goodness-of-fit test for aggregate data – the by far most frequently used model test in past applications of MPT models (Erdfelder et al., 2009; Hu & Batchelder, 1994; Riefer & Batchelder, 1988; Schmidt et al., 2023). For the pair-clustering model, the null hypothesis of equal *u* parameters for word pairs and singletons serves as the goodness-of-fit criterion (Batchelder & Riefer, 1986; Riefer & Batchelder, 1988). The standard $G^2$ test is based on multinomial sampling theory and thus assumes homogeneity of parameters across participants. Hence, it ignores parameter variance between individuals as a source of variability in the data, resulting in a systematic underestimation of standard errors (see Appendix A in Klauer, 2006). Consequently, the $G^2$ goodness-of-fit test based on aggregated data is biased against $H_0$ when *U* varies between individuals. This causes model rejection rates exceeding the nominal $\alpha$ under $H_0$, the more so the larger the variance between individuals and the larger the sample size. This prediction converges with results of a previous simulation study for the pair-clustering model (Riefer & Batchelder, 1991). However, we will replicate this finding as part of our simulation study to assess possible dependencies of the bias against $H_0$ on specifics of the simulation setting. We will also compare the aggregate $G^2$ test with alternative $G^2$ sum tests that are more reasonable in case of parameter heterogeneity.

## A Monte Carlo experiment

In the present section, we report a Monte Carlo experiment primarily designed to assess the robustness of complete-pooling Maximum Likelihood (ML) parameter estimates when a model holds for each individual, but parameters vary between individuals. In addition, we also consider the robustness of the likelihood-ratio $G^2$ test for aggregated data and alternative $G^2$ sum tests for individual frequency data. We use the pair-clustering MPT model for the Monte Carlo experiment because this model (1) is quite typical for the MPT model family in general (in terms of number of trees, parameters, and branches), (2) is well established and has been used since decades, (3) allows to investigate consequences of SAI violations, EAI violations, and combinations thereof, and (4) provides for the possibility to assess the effectiveness of two methods to eliminate or at least attenuate aggregation bias induced by SAI violations.

As outlined in the previous section, our robustness predictions refer to asymptotic properties of parameter estimates and should thus be most obvious in simulation results when the number of simulated participants and responses is large. However, to assess the trustworthiness of complete-pooling estimates for different scenarios of practical relevance, we consider not only the large sample case but also interesting small sample cases for both the number *M* of responses per participant (i.e., the number of word pairs and singletons) and the number *N* of participants.

We use the latent-trait model as the data generating model for our simulations (Klauer, 2010; Heck et al., 2018; Nestler & Erdfelder, 2023). According to this hierarchical MPT model, the probit-transformations of *C*, *R*, and *U* (plus *A* in case of the four-parameter model version) follow a joint multivariate normal distribution across individuals. The main advantage when using the latent-trait model is the flexibility in defining standard deviations and correlations in line with requirements concerning the SAI and EAI properties of the MPT model. Another advantage is that the group-level means of the data generating model provide a natural frame of reference for calculating estimation bias.

### Design

The core dependent variable of our simulation experiment is the bias (B) in the ML parameter estimates for the aggregated data, that is, B = (average ML estimate – true group-level mean parameter). In addition, we investigated rejection rates of model tests, that is, observed proportions of $G^2$ (aggregate vs. sum) statistics significant at the $5\%$ level under the null hypothesis of the three-parameter pair-clustering model.

The independent variables of our Monte Carlo experiment comprise (1) the version of the pair-clustering model considered (the original version with three parameters that violates SAI vs. the extended version with four parameters that does not), (2) the proportion of word pairs in the study list (50%, i.e., as many singletons as word pairs, vs. 100%, i.e., only word pairs, no singletons), (3) the number of word pairs per participant ($m_1 = 20$ vs. $m_1 = 8$ vs. $m_1 = 4$), and (4) the number of participants ($N = 1000$ vs. $N = 10$ vs. $N = 1$). Note that the $N = 1$ simulations are relevant for the no-pooling approach in MPT modeling where parameters are estimated separately for each participant. These single-subject estimates are then typically entered in ANOVA, correlation, or regression analyses in a separate second step. Our simulations can reveal how no-pooling approaches compare to complete-pooling approaches in terms of reducing estimation bias.

In addition, we manipulated group-level means of *C* and *R*, standard deviations of *C*, *R*, and *U*, and correlations between model parameters scale in three levels each. This results in another six factors of our Monte Carlo experiment, namely, (5) means of *C* ($\mu _C =.20,.50,.80$), (6) means of *R* ($\mu _R =.20,.50,.80$), (7) standard deviation of either parameter ($\sigma =.00,.15,.30$), (8 and 9) correlations between parameters co-occurring within branches (i.e., $\rho _{CR} =.00,.25,.50$ and $\rho _{CU} =.00,.25,.50$, respectively), and (10) correlations between parameters located in different branches ($\rho _{UR} =.00,.25,.50$). While means and standard deviations refer to the probability scale, correlation levels hold exactly on the probit-scale and only approximately on the probability scale. However, correlation approximation accuracy is very high, especially when parameter means are .50. Only when parameter distributions are shifted towards the boundaries 0 or 1, minor deviations from the nominal correlations are observable on the probability scale. Note that we included even extreme cases of heterogeneity such as $\sigma =.30$ (roughly corresponding to the standard deviation of a uniform distribution on the unit interval [0, 1]) to make sure that our Monte Carlo experiment explores the full range of parameter variability that is conceivable in principle.

For the three-parameter pair-clustering model, cross classification of the independent variables (2) to (10) results in $2 \cdot 3^8 = 13,122$ simulation conditions. Of these, 4212 conditions are inadmissible because they combine nonzero correlations between parameters with standard deviation $\sigma = 0$, which is not possible. After exclusion of these conditions, the final number of admissible parameter conditions was 8910.

For the four-parameter model extension that includes a fourth parameter *A*, only the model that includes both word pairs and singletons is identifiable. Hence, we used an equal number of word pairs and singletons. We focused on $E(C) = E(R) = 0.2$ and $E(C) = E(R) = 0.5$ in these simulations because an expectation of 0.8 produced virtually the same results as an expectation of 0.2. Both the number of pairs and singletons ($m_1 = m_2 = 20$ vs. $m_1 = m_2 = 4$) and the number of participants ($N = 1000$ vs. $N = 10$) were manipulated in two levels. While the standard deviations as well as the correlations $\rho _{CR}$, $\rho _{CU}$, and $\rho _{UR}$ were varied using the same three levels as before, parameter *A* was always uncorrelated with *U* ($\rho _{AU} = 0$) and had the same correlations with other parameters as *U* had (i.e., $\rho _{CA} = \rho _{CU}$ and $\rho _{AR} = \rho _{UR}$).^4^ Overall, this results in another $2^3 \cdot 3^4 = 648$ simulation settings, 208 of which were again inadmissible because they combine nonzero correlations between parameters with standard deviation $\sigma = 0$. Thus, the final number of admissible simulation settings for the four-parameter pair-clustering model was 440.

For all simulation scenarios, the mean of parameter *U* (and *A*, if considered) was fixed at .50. A total of 1000 Monte Carlo samples was drawn from the data-generating model for each simulation condition. The bias was computed for the average ML estimate across the 1000 samples.

### Implementation

The simulation was conducted in R, using TreeBUGS (Heck et al., 2018) to generate samples from the latent-trait model in line with the settings of the Monte Carlo study. MPTinR (Singmann & Kellen, 2013) was used to analyze the aggregated data based on the corresponding version of the pair-clustering model. Both the R code of the Monte Carlo experiment and all simulation results are available on the OSF (https://osf.io/agwhs).

### Results

We first consider simulation results for the original three-parameter version of the pair-clustering model (Batchelder & Riefer, 1980, 1986). As expected, the correlation between *U* and *R* – a correlation between parameters in different branches that cannot affect aggregation bias – had no effect at all on aggregate estimates and is therefore not considered in the figures below. We mainly focus on simulation scenarios with all parameter means located in the center of the parameter space (i.e., $E(C) = E(R) = E(U) =.50$) and discuss effects of means closer to the boundary of the parameter space subsequently. Bias is classified as clearly noticeable when the deviation of the average ML estimate from the true parameter mean exceeds .10.

**Fig. 2:**
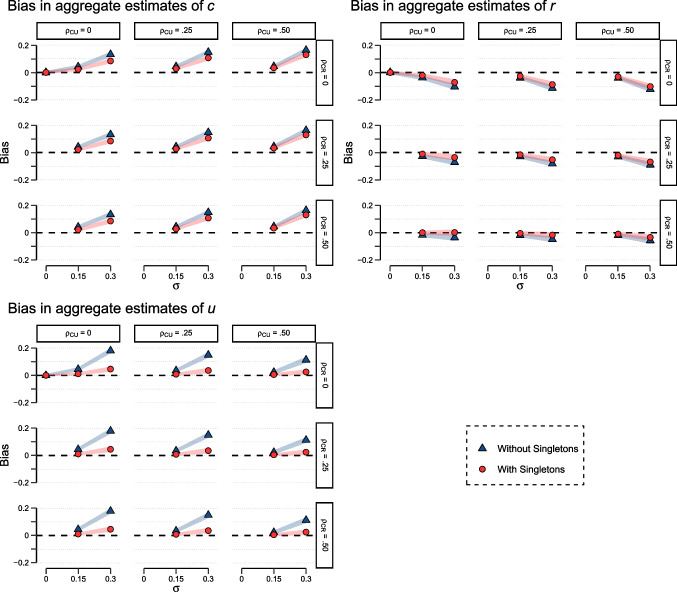
Bias for parameter estimates of *c* (*top left*), *r* (*top right*), and *u* (*bottom*) in the three-parameter pair-clustering model as a function of parameter variability $\sigma$, separately for models with (*red*) vs. without (*blue*) inclusion of singletons and different parameter correlations. $\sigma$ = Standard deviations of *C*, *R*, and *U* across individuals; $\rho _{CU}$, $\rho _{CR}$ = Correlation between *C* and *U*, as well as *C* and *R*, respectively. The widths of the red and blue lines indicate the upper and lower 2.5% quantiles based on 1000 simulation runs, respectively

For this scenario, Fig. 2 illustrates large sample aggregation bias (i.e., $N = 1000$ and $m_1 = m_2 = 20$) for each of the three parameters as a function of their standard deviations on the probability scale. This case is most relevant with respect to our predictions. The red lines (i.e., confidence bands) marked with dots illustrate the results obtained when singletons are included into the analysis. In line with the prediction, aggregation bias is generally smaller than in case of the blue lines marked with triangles that represent analyses based on the $m_1 = 20$ word pairs per participant only (Fig. 2). This effect is most pronounced for the parameter that determines singleton performance, that is, parameter *u*.

The plot most relevant for assessing selective violations of SAI is the one in the upper left field for each parameter (i.e., the one with $\rho _{CU} = \rho _{CR} =.00$, indicating that WEAI is met). The remaining eight plots per parameter reflect scenarios where WEAI is violated with respect to one or more branches of the model. Since correlations are impossible if $\sigma = 0$ holds for the variables involved, the respective point is omitted for these eight plots.

As expected, while there is no bias when parameter standard deviations are zero, bias generally increases when variability increases. For parameter estimates of *u*, this bias is positive and generally quite small, except for the case of a very large standard deviation $\sigma =.30$ when only word pairs are considered in the analysis. The primary source of bias in estimating *u* appears to be the violation of SAI since correlations between *C* and *U* or *C* and *R* barely affect the bias, especially when word pairs and singletons are analyzed conjointly. For parameter estimates of *c*, the pattern looks generally similar. However, for this parameter, less is gained from including singletons into the analysis (compared to analyzing word pairs in isolation). Interestingly, aggregate estimates of parameter *r* show negative aggregation bias that increases with parameter variability. This bias is slightly more pronounced when singletons are excluded from analysis. Notably, bias for *r* is affected by EAI violation. More precisely, the larger the correlation between *C* and *R*, the less negative the estimation bias for *r* becomes. This partially compensates for the negative bias evident when EAI holds.

**Fig. 3:**
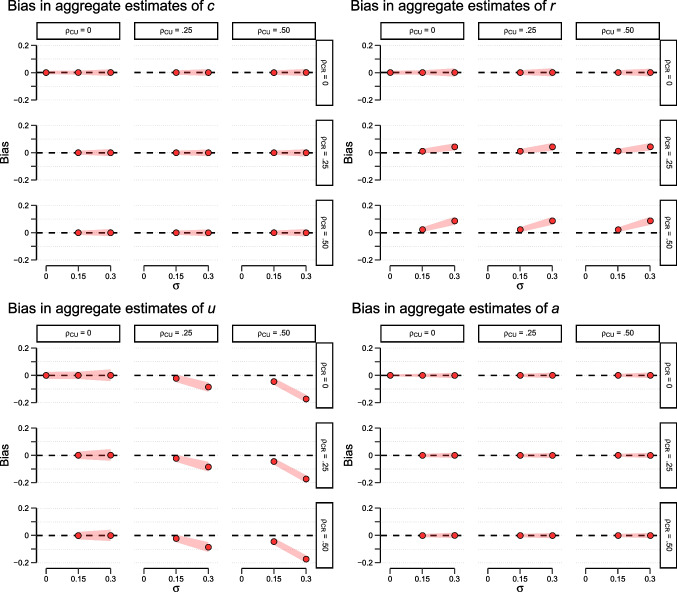
Bias for parameter estimates of *c* (*top left*), *r* (*top right*), *u* (*bottom left*), and *a* (*bottom right*) in the four-parameter pair-clustering model as a function of parameter variability, separately for different parameter correlations. $\sigma$ = Standard deviations of *C*, *R*, *U*, and *A*; $\rho _{CU}$, $\rho _{CR}$ = Correlation between *C* and *U*, as well as *C* and *R*, respectively. The widths of the red lines indicate the upper and lower 2.5% quantiles based on 1000 simulation runs, respectively

Figure 3 illustrates the corresponding results for the four-parameter version of the pair-clustering model. This model requires both word pairs and singletons for analysis so that all simulation results are printed in red color. As before, the figure displays results for $N = 1000$ participants and 20 word pairs plus 20 singletons per participant. As implied by the structural invariance property of this model, for all four parameters, bias completely vanishes when $\rho _{CU} = \rho _{CR} =.00$ holds (upper left plot for each parameter). Only when empirical aggregation invariance is violated, estimation bias may occur. In this case, aggregate estimates of parameter *u* show a clear negative bias when $\rho _{CU} =.50$, but the amount of bias is negligible if $\sigma _U \le .15$. While estimates of *a* and *c* show no bias even if EAI is violated, parameter *r* is more problematic. When a strong correlation between *C* and *R* is combined with very large standard deviations of both parameters, results show a clear positive bias. Again, this bias diminishes when standard deviations do not exceed .15.

For expected values *E*(*C*) and *E*(*R*) closer to the boundary of the parameter space (i.e., .20 vs. .80), the results for the three-parameter pair-clustering model resemble those shown in Figs. 2. We thus refrain from including these results in the current paper and provide the corresponding figures in the supplemental materials only (https://osf.io/agwhs; Supplemental Material I). As is evident from Figures 1 to 5 in Section 1 of our Supplemental Material I, the bias in *u* estimates is not affected by *E*(*C*) and *E*(*R*). However, while the negative bias for parameter *r* increases with the true *E*(*R*), aggregation biases in both *c* and *r* tend to vanish for large cluster storage probabilities (i.e., $E(C) =.80$). A comparison with Figures 6 to 10 in the same section (that refer to $m_1 = 8$ rather than $m_1 = 20$ word pairs) reveals that the number of items per individual has a negligible effect on aggregate estimates when the number of simulated individuals is huge (i.e., $N = 1000$).

We replicated the analyses summarized in Fig. 2 and in Section 1 of the Supplemental Material I for the smallest possible sample size ($N = 1$) (i.e., single-subject analyses) for large versus small item sets studied by each individual. The results are summarized in Figures 11 to 15 (for $m_1 = 20$ word pairs) and Figures 16 to 20 (for $m_1 = 8$ word pairs) of Supplemental Material I. While the bias introduced by parameter heterogeneity shows similar patterns as already discussed for $N = 1000$, the new result in these simulations is small-sample estimation bias in case of parameter homogeneity ($\sigma = 0$). In the pair-clustering model, as already observed by Riefer and Batchelder (1991, p. 323), this latter type of bias is most pronounced for parameter *r* and tends to be positive when both *E*(*C*) and *E*(*R*) are small.

A full table of results for all 8910 conditions of the Monte Carlo simulation for the three-parameter model can be obtained from Supplemental Material II and a corresponding table of all 440 conditions for the four-parameter model from Supplemental Material III (https://osf.io/agwhs).

To investigate the behavior of the $G^2$ goodness-of-fit test for the three-parameter pair-clustering model, our simulations employed the same parameter standard deviations previously assessed by Riefer and Batchelder (1991), that is, $\sigma$ = .00, .10, and .20. These values are reasonable for typical practical applications and facilitate comparisons with their results. In line with Riefer and Batchelder (1991, p. 331, Fig. 9), we observed that $G^2$ tests for aggregated data tend to reject $H_0$ more often than specified by the nominal $\alpha$ level if $\sigma _U > 0$, the more so the larger the heterogeneity and the larger the number of data points. If $\sigma _U \le .10$ and the product $N \cdot M$ is small or moderate (i.e., up to 400), this bias appears negligible. However, for $\sigma _U =.20$ and $N \cdot M \ge 200$ , the rejection rate may exceed the nominal $\alpha$, potentially reaching extreme rejection rates larger than $50\%$ (e.g., if $N = 100$, $m_1 = m_2 = 20$, and $\sigma _U =.20$; see the red dots and confidence bands in Figures 21 to 23, pp. 23–26 in Section 2 of Supplemental Material I). The same figures show that this bias may vanish when the $G^2$ test is conducted separately for each participant and the individual $G^2$ statistics are summed up across participants, resulting in $G^2$ sum statistics that are asymptotically $\chi ^2$-distributed with $df = N$ if the model holds. Categories with zero frequencies may be a problem for individual $G^2$ statistics but can be addressed by adding a small positive constant (in our simulations: +0.10) to all category frequencies when zero cells occur. However, because of scarce data per participant, this asymptotic $G^2$ sum test does not work perfectly and may produce inflated $\alpha$ levels, most obviously in our simulations for sum statistics referring to $N \ge 100$ participants (see the green triangles and confidence bands in Figures 21 to 23, pp. 23–26, Supplemental Material I). As a remedy, one can replace the asymptotic distribution by an empirical estimate of the exact reference distribution using the parametric bootstrap (with 500 bootstrap samples per estimated *p* value in our simulations). This parametric bootstrap kept its nominal significance level nicely (see the blue squares and confidence bounds in Figures 21 to 23, Supplemental Material I), with the single exception of $m_1 = m_2 = 4$ observations (shown in Figure 23). Obviously, such a small number of observations is simply insufficient to allow reasonable model applications at the level of individuals and must thus be avoided in practice.^5^

## Discussion

Results of our Monte Carlo experiment confirmed the main prediction that aggregation bias in MPT parameter estimates increases with heterogeneity in those parameters that are involved in violations of structural or empirical aggregation invariance. Biases vanish completely when both structural and empirical aggregation invariance holds. While biases may occur when either of these conditions is not met, their strength appears negligible in practical applications unless standard deviations of parameters exceed .15.

Fortunately, there are several remedies for potential aggregation biases. One remedy is the inclusion of trees into the model that are SAI and thus provide unbiased parameters. An example is the singleton tree in the pair-clustering model. Hence, including more singletons into the data analyses at least partly compensates for aggregation biases in the word-pair condition. In the pair-clustering model, this pattern is most pronounced for parameter *u*, as is evident from Fig. 2.

An even more effective remedy is the transformation of an MPT model that violates SAI into a model that does not. An example is the four-parameter version of the pair-clustering model that replaces the second occurrence of the *u* parameter in the word-pair branches by an independent *a*  parameter. Whenever possible, MPT modelers of aggregated data should use this technique to eliminate or at least mitigate some of the problems that may otherwise result from aggregation.

However, both remedies focus on SAI only and do not rule out problems caused by EAI violations in general and WEAI violations in particular. Note that WEAI violations essentially depend on within-branch covariances between parameters, for example, cluster storage and cluster retrieval in the pair-clustering model. Since $Cov(C,R) = \sigma _C \cdot \sigma _R \cdot \rho _{CR}$ and techniques to enforce zero correlations between parameters (such as $\rho _{CR} = 0$) do not exist, the only effective technique to minimize bias is to decrease the standard deviations of the parameters involved. For MPT analyses of aggregated data, participant samples should thus be as homogeneous as possible to reduce the risk of strong within-branch covariances between parameters. If this recommendation is combined with the use of structurally aggregation invariant MPT models, results based on the complete-pooling approach are generally trustworthy. This means that, given sufficiently large samples, aggregate parameter estimates provide close approximations to the true expected parameter values. What is more, these complete-pooling estimates will in general outperform no-pooling estimates in which data of participants are analyzed separately and individual estimates are averaged subsequently. Especially if the number of responses per participant is small, the latter approach involves the serious risk that small-sample estimation bias distorts results systematically, even if parameters do not vary across individuals.

A possible objection refers to the fact that our robustness arguments are supported by simulation studies for one MPT model only, the pair-clustering model. Is there reason to expect that our robustness arguments generalize to other MPT models as well? We argue that this is the case, at least for models that fall in the two most frequently employed hierarchical MPT model classes – beta-MPT models (Smith & Batchelder, 2010) and latent-trait MPT models (Klauer, 2010; Heck et al., 2018; Nestler & Erdfelder, 2023). As detailed in the Appendix, the group-level mean parameters of any of these hierarchical models can be approximated very well by MPT parameters based on aggregated data, provided that the respective MPT model is structurally aggregation invariant. First, if a beta-MPT model holds, the aggregate parameters of structurally aggregation invariant MPT models must match the group-level mean parameters, because this model family assumes mutual independence of beta-distributed model parameters (see Appendix, Corollary 1). The same applies to the latent-trait model, but only when model parameters (or parameter complements) that co-occur in the same branches are uncorrelated (see Appendix, Corollary 2). Second, when the latent-trait model holds but at least two same-branch parameters are correlated, then aggregate parameters will not match the group-level mean parameters exactly but still approximate them quite well. To see this, consider a tree branch with two parameters $\Theta _{s}$ and $\Theta _{s'}$. We assume that these two parameters have a correlation of $\rho$ and the same standard deviation $\sigma$ across individuals. Then the definition of the covariance (Appendix, Eq. 5) implies $Cov(\Theta _s, \Theta _{s'}) = E(\Theta _s \cdot \Theta _{s'}) - E(\Theta _s) \cdot E(\Theta _{s'}) = \rho \cdot \sigma ^2$. In other words, the bias in the expected branch probability (i.e., the deviation of the expected branch probability $E(\Theta _s \cdot \Theta _{s'})$ from the corresponding branch probability $E(\Theta _s) \cdot E(\Theta _{s'})$ given uncorrelated parameters) is just the product of the correlation and the variance of the parameters. When $\rho$ and $\sigma$ do not exceed 0.5 and 0.3, respectively, this bias cannot exceed $0.5 \cdot 0.3^2 = 0.045$ (or $-0.045$ if $\rho = -0.5$ holds). Given that even a completely flat uniform distribution on the unit interval [0, 1] has a standard deviation slightly less than 0.3, a maximum $\sigma =.15$ appears more realistic. With this assumption, the maximum bias in the expected branch probability reduces to 0.01125 if $\rho = 0.5$ (or to $-0.01125$ if $\rho = -0.5$). This small bias will have a rather mild effect on parameters. This is in line with our simulation results observed for the pair-clustering model. However, the argument is not limited to this specific model but generalizes to all latent-trait MPT models.

Our theoretical results converge with empirical results of a recent multiverse meta-analysis of 164 published MPT data sets for nine different MPT models, including 13,956 participants in total (Singmann et al., 2024). This multiverse analysis compared group-level mean parameter estimation results for frequentist and Bayesian methods based on no pooling (single subjects), partial pooling (hierarchical modeling), and complete pooling (aggregated data). Notably, absolute divergences between estimates based on different methods were generally small on average (less than .04). This was especially true for structurally aggregation invariant MPT models applied to large samples (providing small standard errors; see Singmann et al., 2024, Figure 5). If larger deviations occurred, they were typically associated with violations of structural aggregation invariance or with smaller samples (i.e., larger standard errors). Overall, the results of this multiverse meta-analysis confirm our robustness argument for structurally aggregation invariant MPT models quite nicely.

While ML parameter estimation based on aggregated data is relatively robust for realistic standard deviations, estimates of standard errors and confidence intervals are more problematic when individual variability is ignored (cf., Klauer, 2006, Appendix A). The same applies to standard $G^2$ goodness-of-fit tests of MPT models (Riefer & Batchelder, 1991). Even if heterogeneity between participants is relatively small, rejection rates of valid MPT models may exceed the nominal $\alpha$-level substantially when the number of data points is large, as revealed by our simulation of the $G^2$ test for aggregated data. Hence, aggregated data should not be used for interval estimates or model tests unless variability between individuals can be shown to be small (e.g., by means of the heterogeneity test proposed by Smith & Batchelder, 2008).

In practical applications of MPT model tests, this bias against $H_0$ is unproblematic if the model test turns out to be insignificant. If a model test becomes significant, however, this may mean that the model itself holds for each participant and is rejected because of parameter heterogeneity only. We recommend additional model assessment methods in these cases, such as descriptive (e.g., graphical) comparisons of observed and expected frequencies across response categories in different trees. If a formal significance test is deemed to be necessary, the $G^2$ goodness-of-fit test can be performed for each participant separately. By adding up the resulting goodness-of-fit statistics across *N* participants, one obtains an overall $G^2$ sum statistic that asymptotically follows a central $\chi ^2$ distribution with $N \cdot df$ degrees of freedom if the respective MPT model holds for each participant (note that this method is implemented in MPTinR, see Singmann & Kellen, 2013). Here, *df* denotes the degrees of freedom for each individual test. As shown in our simulation study of the $G^2$ sum statistic for the pair-clustering model, this modified model test is not without problems either, simply because it is an asymptotic test that requires more responses per participant than usually obtained in MPT applications. However, as also shown in our simulation study, when replacing the asymptotic $\chi ^2$ distribution of the $G^2$ sum statistic with an empirical estimate of the exact distribution obtained via the parametric bootstrap, the bootstrap-estimated *p* value conforms to the nominal $\alpha$ level quite well. The only precondition is that the number of responses per participant is not too small (i.e., at least $m_1 = m_2 = 8$ for the pair-clustering model). For smaller response frequencies, model tests on the individual level are not sensible.

Alternatively, one can of course switch to one of the hierarchical MPT model frameworks (e.g., the latent-trait model, cf. Klauer, 2010; Heck et al., 2018; Matzke et al., 2015; Nestler and Erdfelder, 2023) and assess the goodness of fit accordingly. This is certainly the simplest and most direct method to address individual variability in MPT data. If the heterogeneity test proposed by Smith and Batchelder (2008) indicates significant variability between individuals, resorting to the latent-trait model can be considered a reasonable default option for MPT modeling that has several advantages compared to analyses of aggregated data. This especially holds with respect to standard errors, confidence intervals, and model tests. Yet, even in this case ML estimates based on aggregated data may be useful because they provide reasonable start values for the group-level mean parameters in iterative estimation procedures. This procedure is implemented, for example, in the mptmem R package provided by Nestler and Erdfelder (2023).

To reiterate, our analyses of MPT aggregation invariance properties assume that the respective model holds for each participant in the sample. Note that this assumption is shared by all MPT methods proposed in the literature, irrespective of whether these methods use no-pooling, partial-pooling or complete-pooling approaches. Without this assumption, there is no basis for expecting that methods will converge in their results with respect to group-level means of MPT parameters. Yet, a recent MPT-multiverse analysis of 164 MPT data sets by Singmann et al. (2024) shows strong convergence in estimation results if (1) an MPT model is structurally aggregation invariant and (2) standard errors of estimates converge to zero (or equivalently, when the number of participants and the number of items becomes very large). According to Singmann et al. (2024), this is in line with the assumption that the MPT models included in their multiverse analyses indeed hold for each participant, at least for the data sets at hand. We agree with this assessment.

Clearly, if a model is violated for a subset of participants, then it is not reasonable to aggregate results across all participants. This means that whenever there is a suspicion that an MPT model might not hold for a subset of participants, then a no-pooling approach is mandatory as a first step, that is, a model test for each of the critical participants. If individual goodness-of-fit tests (adjusted for $\alpha$ inflation) suggest model violation, then the respective participants should be removed from the data pool prior to possible aggregation. Otherwise, the results presented and discussed in the current research do not apply.

In sum, MPT parameters based on aggregated data tend to converge with group-level mean parameters, especially if these models are structurally aggregation invariant. This is mirrored in the corresponding parameter estimates, the more so the larger the samples (Singmann et al., 2024). Although not all MPT models are structurally aggregation invariant and violations of empirical aggregation invariance cannot be ruled out in general, countermeasures exist that help increase the robustness of MPT analyses for aggregate data. For realistic assumptions about possible parameter correlations and standard deviations across individuals, approximation accuracy is still satisfactory, even if empirical aggregation invariance is violated to some degree. Thus, ML parameter estimates resulting from aggregate analyses are trustworthy in general, provided that the caveats outlined in this paper are considered. This notwithstanding, especially if sample sizes are small and large within-branch correlations coexist with large standard deviations of parameters, estimation results more strongly depend on the estimation method used (Singmann et al., 2024). Hence, in future research, MPT analyses of aggregated data should be complemented by a multiverse approach that includes hierarchical modeling of the same data whenever feasible.

## Supplementary Information

Below is the link to the electronic supplementary material.

Supplementary file 1 (pdf 486 KB)

Supplementary file 2 (pdf 460 KB)

Supplementary file 3 (pdf 212 KB)

## Data Availability

All analysis scripts and computer code in R required for reproducing the Monte Carlo simulation results reported in this work are provided online on the Open Science Framework (OSF; https://osf.io/agwhs).

## Appendix

### Implications for hierarchical MPT models

The statistical theory outlined in the section on aggregation invariance of MPT models has strong implications for group-level mean parameters of hierarchical MPT models. Recall that all hierarchical MPT models share the assumption that a specific MPT model holds for each participant (i.e., the so-called level 1 model), but parameters may vary between participants. So far, three classes of hierarchical MPT models with individual variability in parameters have been proposed (for an overview, see Schmidt et al., 2023): the beta-MPT model, the latent-trait MPT model, and the latent-class MPT model. In a nutshell, the beta-MPT model (Smith & Batchelder, 2010; Heck et al., 2018) assumes that (1) each of the *S* model parameters $\Theta _{s}$ is beta($a_s$,$b_s$)-distributed across participants with mean $\mu _s = a_s / (a_s + b_s)$ and variance $\sigma _s{^2} = a_s \cdot b_s / ((a_s + b_s + 1) \cdot (a_s + b_s){^2})$ and that (2) all *S* parameters are mutually independent. In contrast, the latent-trait model (Klauer, 2010; Heck et al., 2018; Nestler & Erdfelder, 2023) is based on the assumption that probit-transformations of the *S* parameters follow a joint multivariate normal distribution with mean vector $\varvec{\mu _p}$ and covariance matrix $\varvec{\Sigma _p}$ on the probit scale. While both the beta- and the latent-trait MPT model share the assumption that parameters are continuously distributed, the latent-class MPT model (Klauer, 2006; Stahl & Klauer, 2007) assumes a discrete distribution. According to this model, parameter values may differ between but not within *C* latent classes of participants (i.e., all participants within the same class share their parameter values). In general, $1 < C \le N$, but *C* is typically assumed to be small (i.e., 2, 3, or 4).

With respect to beta-MPT and latent-trait models, Observation 1 has immediate implications, as outlined in the following two corollaries.

#### Corollary 1

**(Equivalence of beta-MPT group-level mean parameters and MPT parameters for aggregated data).** Assume that a beta-MPT model holds in a specific application. Also assume that the corresponding level 1 MPT model is SAI according to Definition 1. Then this MPT model must also hold for aggregated data, and the group-level mean parameter vector *E*$(\varvec{\Theta }) = \varvec{\mu }$ of the beta-MPT model is identical to the parameter vector $\varvec{\theta }$ of the MPT model when applied to aggregated data.

#### Corollary 2

**(Equivalence of latent-trait group-level mean parameters and MPT parameters for aggregated data).** Assume that a latent-trait MPT model holds in a specific application. Also assume that this model is SAI according to Definition 1 and WEAI according to Definition 3. Then this MPT model must also hold for the aggregated data, and the group-level mean parameter vector *E*$(\varvec{\Theta }) = \varvec{\mu }$ on the probability scale matches the parameter vector $\varvec{\theta }$ of the MPT model applied to aggregated data.

Both corollaries follow directly from Observation 1 because (1) the validity of either of the two hierarchical models entails the validity of the MPT model for all individuals and (2) independence of parameters co-occurring within branches. In case of the latent-trait model, this of course only holds conditional on zero covariances of parameters co-occurring within branches, as stated in Corollary 2. Combined with the SAI property of the respective MPT model, aggregation invariance follows. Note that due to the nonlinear nature of the probit-transformation, WEAI for the latent-trait model cannot be assessed directly but only indirectly via the non-diagonal elements of the covariance matrix $\varvec{\Sigma _p}$, which refers to probit-transformed parameters. However, for parameter values not too close to the boundaries of the parameter space, the probit-transformation is approximately linear so that WEAI can be assessed by testing whether the corresponding non-diagonal elements of $\varvec{\Sigma _p}$ are zero. Similarly, because the distribution of latent-trait parameters is symmetrical on the probit scale but in general asymmetrical on the probability scale, the mean parameter vector $\varvec{\mu }$ on the probability scale does in general not match the inverse probit-transformation $\Phi (\varvec{\mu _p})$ of the mean vector on the probit scale (here, $\Phi (.)$ denotes the cumulative distribution function of the standard normal distribution). While $\Phi (\varvec{\mu _p})$ represents the median and the mode of the parameter distribution on the probability scale, it may deviate somewhat from $\varvec{\mu }$.

The situation is more complex for latent-class MPT models. When no more than two parameters per branch occur in such a model, or when stochastic dependencies between parameters are limited to bivariate dependencies (i.e., higher-order dependencies are excluded if there are 3 or more parameters per branch), then aggregation invariance must hold for latent-class MPT models satisfying SAI and WEAI, too. The reason is that expectations of products of two random variables only depend on their covariances in addition to their means (see, e.g., Appendix B in Hays, 1973):

5 $$\begin{aligned} E(\Theta _s \cdot \Theta _{s'}) = E(\Theta _s) \cdot E(\Theta _{s'}) + Cov(\Theta _s, \Theta _{s'}) . \end{aligned}$$

In such cases, the requirement $Cov(\Theta _s, \Theta _{s'}) = 0$ for all parameter pairs co-occurring within branches suffices to guarantee aggregation invariance. If higher order dependencies occur among the parameters of the latent-class model, however, this is not anymore the case.

Consider the HB13 hindsight bias model as an example (e.g., Bayen et al., 2006; Coolin et al., 2015; Coolin et al., 2016; Dehn & Erdfelder, 1998; Erdfelder et al., 2007; Groß & Pachur, 2020). In one of the tree branches of the HB13 model for experimental items (i.e., items receiving feedback following the original judgment (OJ)), three core parameters $\theta _1 = (1-r)$ (probability of OJ recollection failure in hindsight), $\theta _2 = b$ (probability of reconstruction bias in hindsight), and $\theta _3 = c$ (probability of source confusion) co-occur in the same branch, resulting in a source confusion error (i.e., the participant recalls the feedback information as his/her own previous OJ). This single branch suffices to illustrate the problem. To see this, let us assume that a hierarchical HB13 model with $C = 4$ latent classes holds, with parameter values $\theta _1$, $\theta _2$, and $\theta _3$ within latent classes as specified in the left part of Table 1 (columns 3–5). The right part of the table (columns 6–9) provides pairwise and triplewise parameter products, and the last row the expected (i.e., mean) parameters or parameter products across all four classes.

**Table 1:** Hypothetical values of three parameters $\theta _1$, $\theta _2$, and $\theta _3$ and their products within four latent classes, including the implied expected parameter values across classes

Class	size	$\theta _1$	$\theta _2$	$\theta _3$	$\theta _1 \cdot \theta _2$	$\theta _1 \cdot \theta _3$	$\theta _2 \cdot \theta _3$	$\theta _1 \cdot \theta _2 \cdot \theta _3$
1	.25	.20	.20	.20	.04	.04	.04	.008
2	.25	.20	.80	.80	.16	.16	.64	.128
3	.25	.80	.20	.80	.16	.64	.16	.128
4	.25	.80	.80	.20	.64	.16	.16	.128
*E*(.)		.50	.50	.50	.25	.25	.25	.098

*Note*. *E*(.) = Expected value (i.e., mean) of the parameter (or parameter product) across latent classes

It is easily verified that all three parameters are pairwise uncorrelated: For any two parameters, the product of their expectations is identical to the expectation of their products (e.g., *E*$(\theta _1) \cdot$ *E*$(\theta _2) =$ *E*$(\theta _1 \cdot \theta _2) =.25$). However, although WEAI obviously holds for this branch of the HB13 model, EAI does not hold, since *E*$(\theta _1) \cdot$ *E*$(\theta _2) \cdot$ *E*$(\theta _3) =.125$ whereas *E*$(\theta _1 \cdot \theta _2 \cdot \theta _3) =.098$ (see the rightmost column in Table 1). Hence, this hierarchical version of the HB13 model is not empirically aggregation invariant because aggregation across participants predicts the expected frequency for at least one branch of the model incorrectly. Note that this might not only lead to biased parameter estimates when using aggregated data. Since both the beta-MPT and the latent-trait model cannot accommodate higher-order dependencies between parameters, they are both misspecified for this scenario and may also produce biased parameter estimates. Only the latent-class HB13 model with at least four latent classes or no-pooling approaches can capture this scenario properly, the latter of course only when the number of responses per participant is sufficiently large.

## Footnotes

1 In principle, this idea can be extended to hierarchical models with crossed random effects that allow for variability between participants and items (Matzke et al., 2015).

2 To simplify notation, we slightly deviated from Hu and Batchelder (1994) and followed other authors (e.g., Klauer, 2010) by omitting $c_ {kji}$ in the model equation. This factor absorbs multiplicative constants in the *i*-th branch that terminates in category $C_{kj}$. If this factor is required for a specific model, it is possible to compensate for this simplification, for example, by fixing one of the model parameters to a constant or by adding an additional branch to the model as in case of the pair-clustering model.

3 Note that the correlation between *C* and *U* must be zero when *U* is a constant *u*.

4 Note that a non-zero correlation between *A* and *U* would introduce an additional violation of EAI so that results cannot be compared with those obtained for the three-parameter model.

5 Note that all these results necessarily refer to the three-parameter pair-clustering model, because the four-parameter version of this model is not testable (since $df = (4-1) + (2-1) - 4 = 0$).
