# Supplementary material for: On aggregation invariance of multinomial processing tree models
Source: Behav Res Methods. 2024 Oct 14;56(8):8677–94. doi: 10.3758/s13428-024-02497-y (PMC11525265; doi:10.3758/s13428-024-02497-y)
Supplement: Supplementary file 1 — (pdf 486 KB) [file 13428_2024_2497_MOESM1_ESM.pdf]

On Aggregation Invariance of Multinomial Processing Tree Models

Supplemental Material I: Additional Figures

Edgar Erdfelder, Julian Quevedo Pütter, & Martin Schnuerch

Bias in 3-Parameter Pair Clustering Model

The following plots visualize the bias in estimating c, r, and u in the 3-parameter pair clustering model based on aggregated data as a function of parameter variability ($\sigma_C = \sigma_R = \sigma_U = \sigma$) and correlations, sample size (in terms of No of participants and No of responses per person), and expected parameter values. Note that in all Figures, the expected value of U and the correlation between U and R are fixed as $E(U) = .50$ and $\rho_{RU} = 0$, respectively. In each of the following Figures 1–20, confidence bands (i.e., line widths) indicate the upper and lower 2.5% quantiles of simulation results, respectively, truncated at $+.25$ and $-.50$ when necessary to fit the ordinate scale.

Large Samples

Figures 1–10 visualize the bias for extremely large samples ($N = 1000$) with varying numbers of responses per participant. In **Figures 1–5**, the number of word pairs is fixed at $m_1 = 20$. Expected values of C and R vary as follows:

- Figure 1: $E(C) = E(R) = .20$
- Figure 2: $E(C) = .20$, $E(R) = .80$
- Figure 3: $E(C) = E(R) = .50$
- Figure 4: $E(C) = .80, E(R) = .20$
- Figure 5: $E(C) = E(R) = .80$

In **Figures 6–10**, the number of word pairs is fixed at $m_1 = 8$. Expected values of C and R vary as follows:

- Figure 6: $E(C) = E(R) = .20$
- Figure 7: $E(C) = .20$, $E(R) = .80$
- Figure 8: $E(C) = E(R) = .50$
- Figure 9: $E(C) = .80, E(R) = .20$
- Figure 10: $E(C) = E(R) = .80$

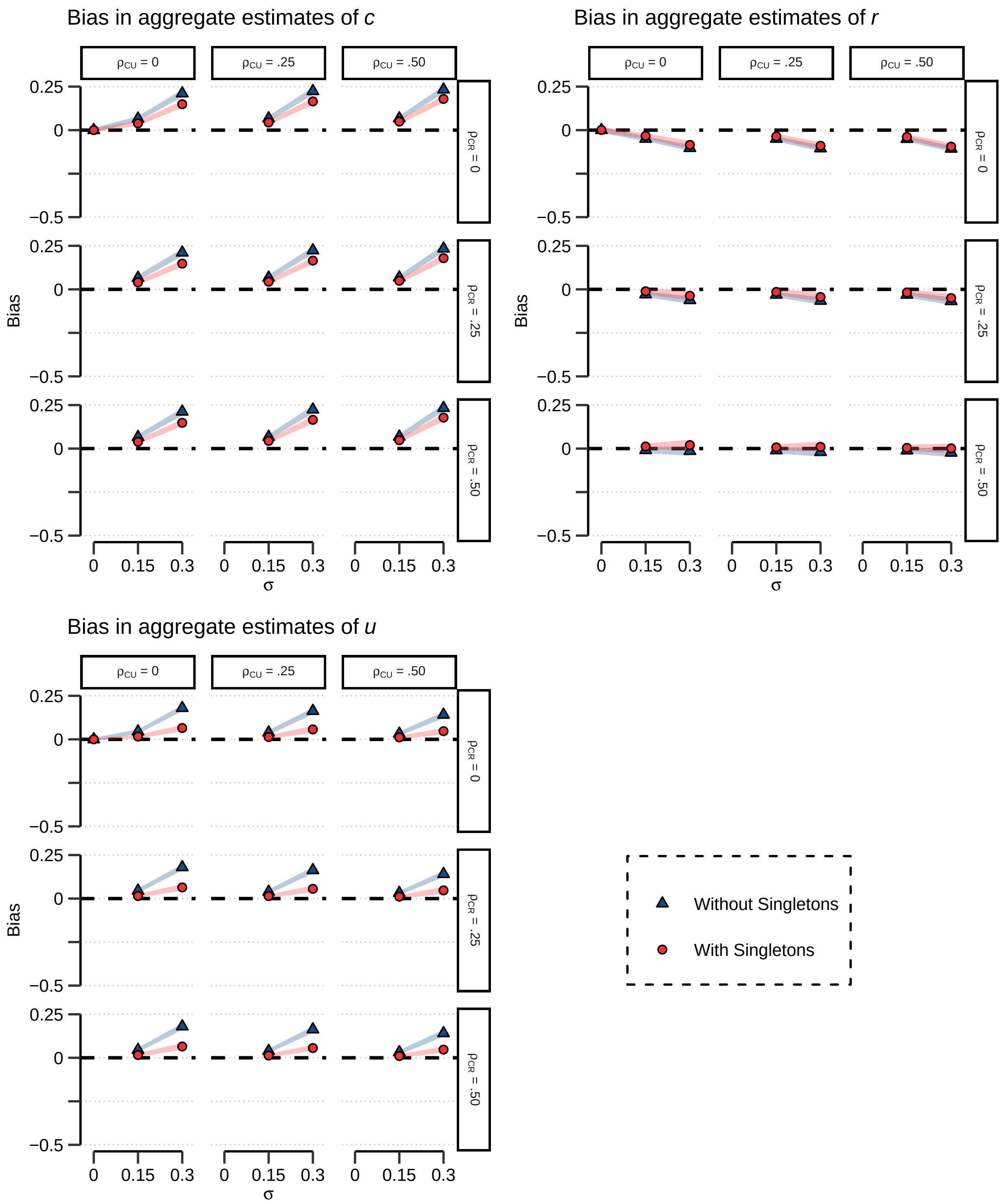

Figure 1: $N = 1000$, $m_1 = 20$, $E(C) = E(R) = .20$

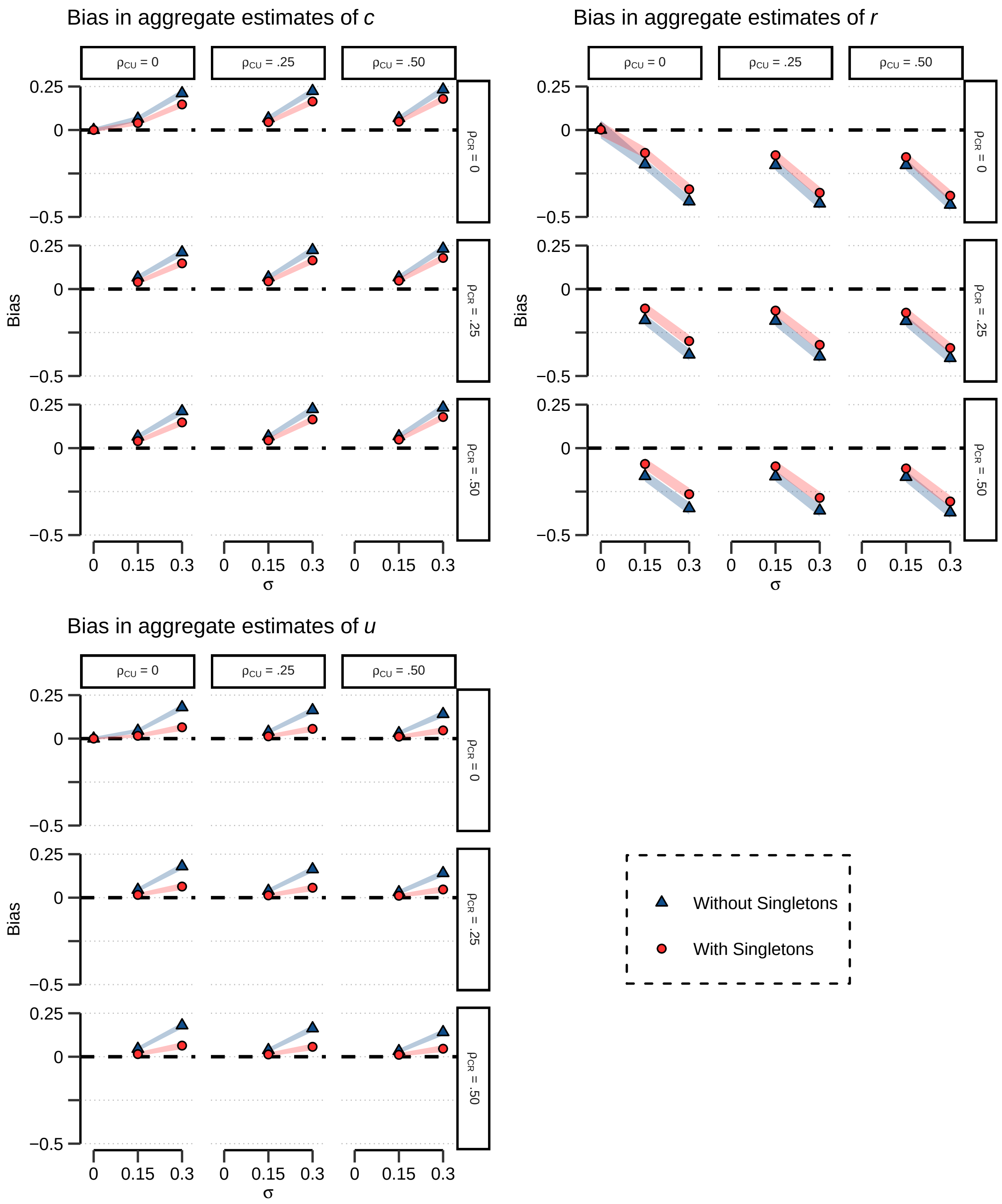

Figure 2: $N = 1000$, $m_1 = 20$, $E(C) = .20$, $E(R) = .80$

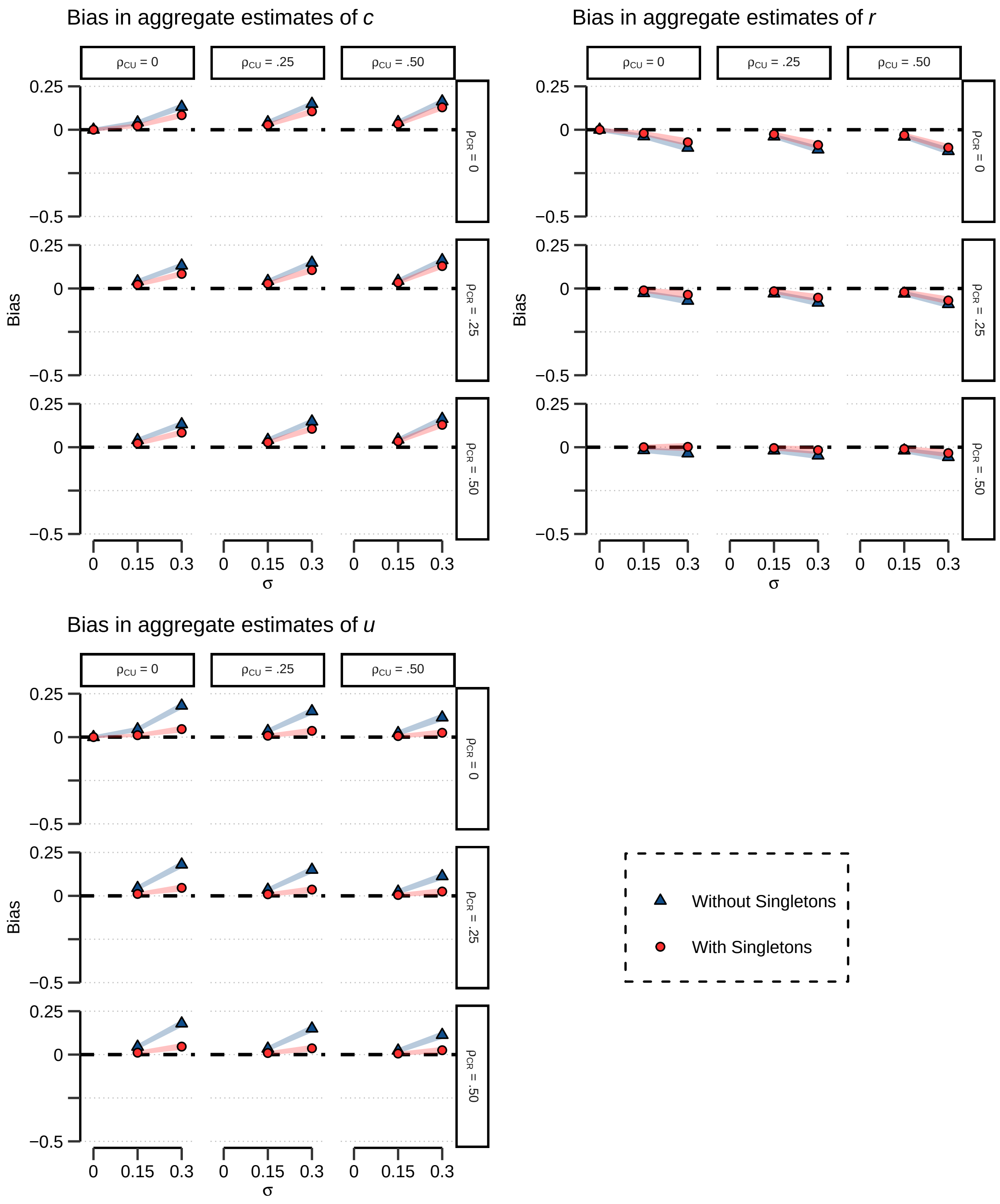

Figure 3: $N = 1000$, $m_1 = 20$, $E(C) = E(R) = .50$

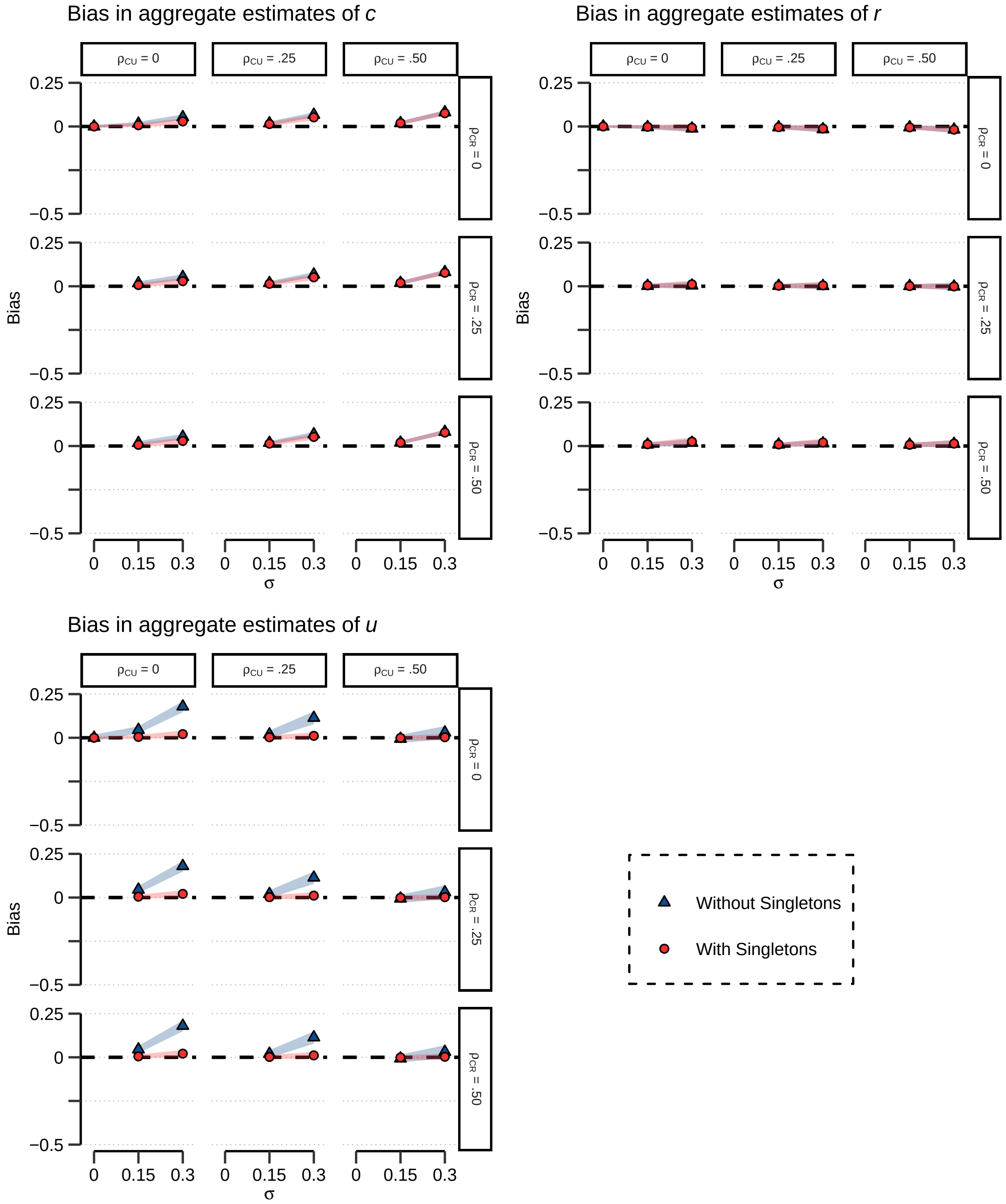

Figure 4: $N = 1000$, $m_1 = 20$, $E(C) = .80$, $E(R) = .20$

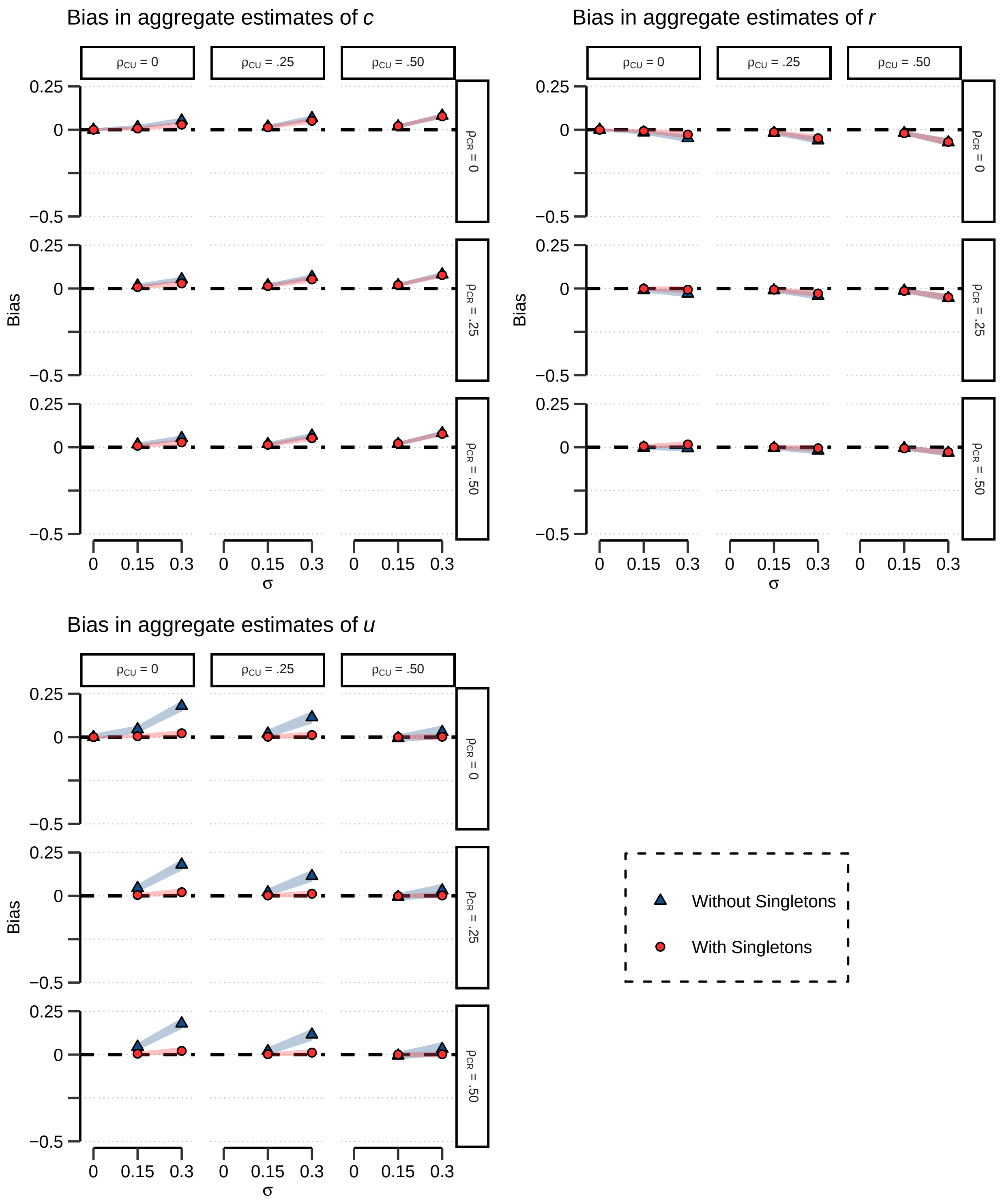

Figure 5: $N = 1000$, $m_1 = 20$, $E(C) = .80$, $E(R) = .80$

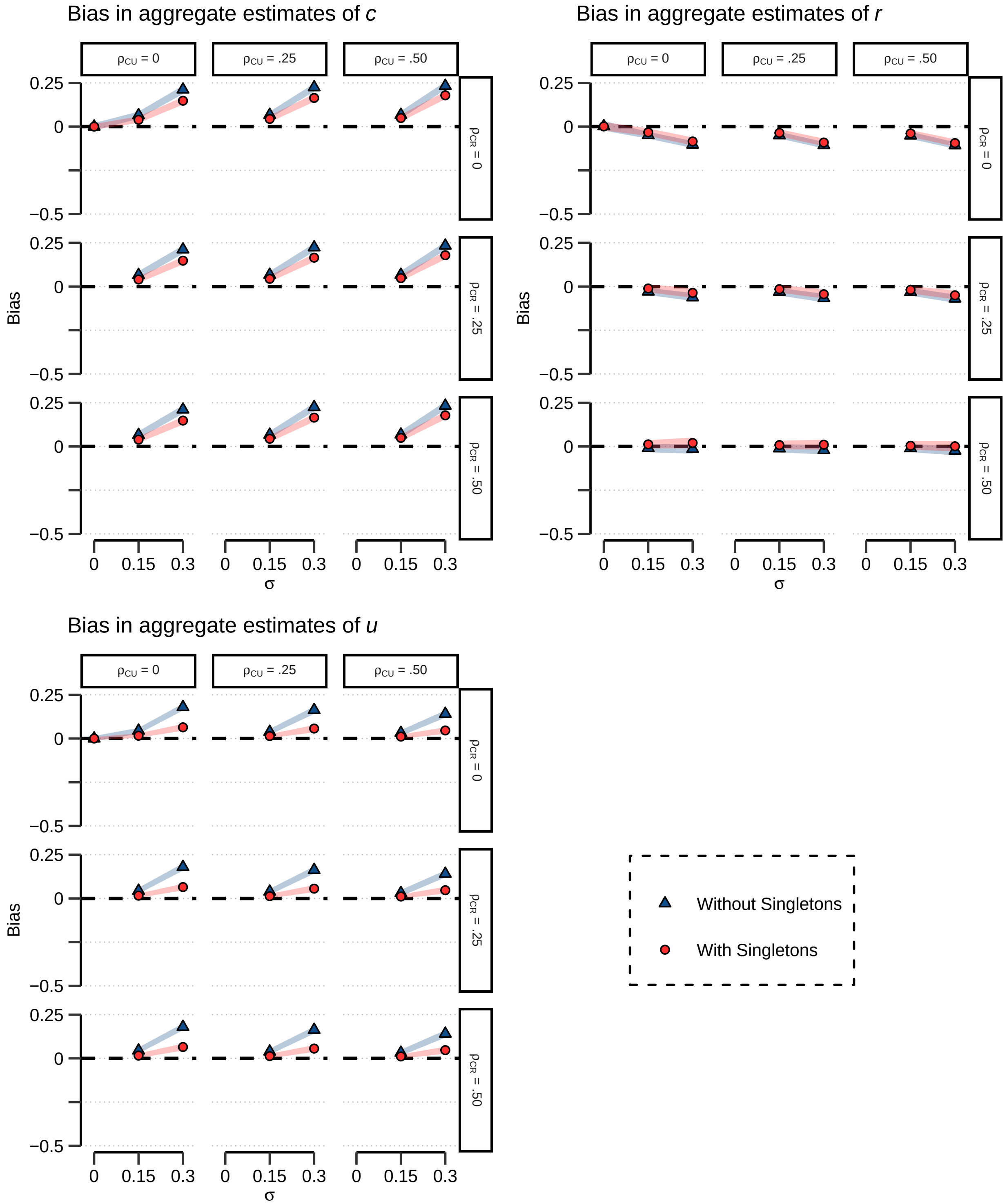

Figure 6: $N = 1000$, $m_1 = 8$, $E(C) = E(R) = .20$

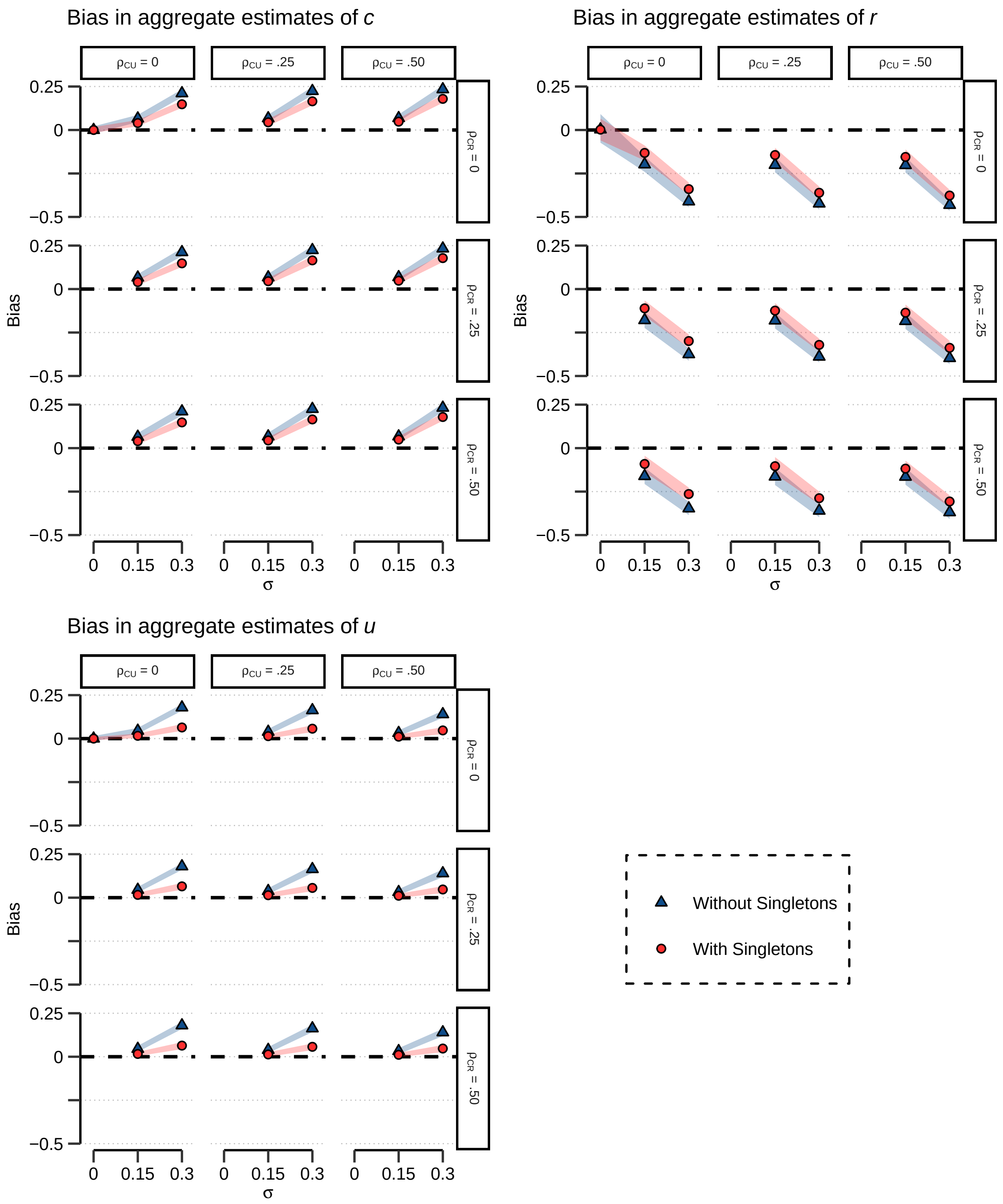

Figure 7: $N = 1000$, $m_1 = 8$, $E(C) = .20$, $E(R) = .80$

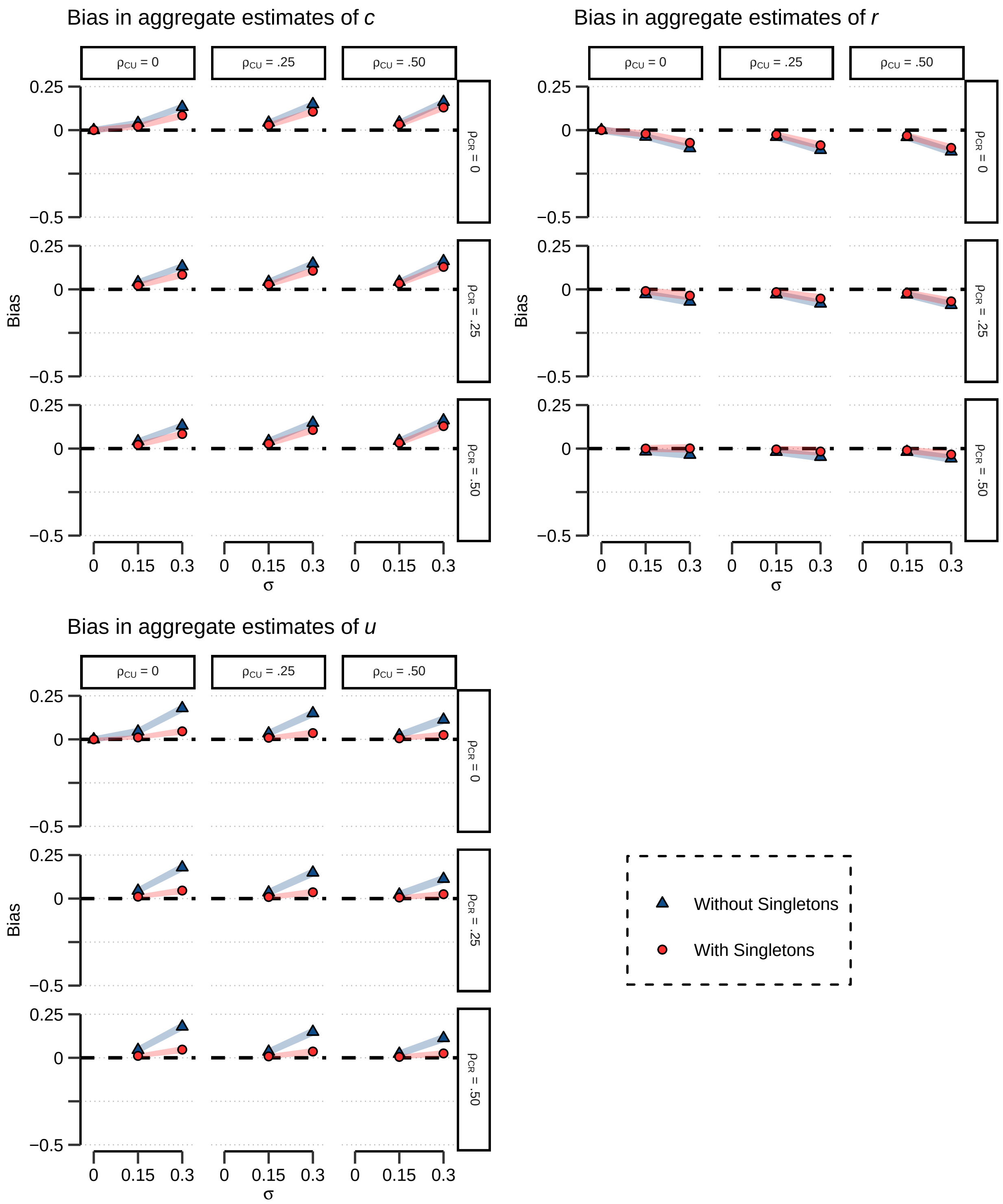

Figure 8: $N = 1000$, $m_1 = 8$, $E(C) = E(R) = .50$

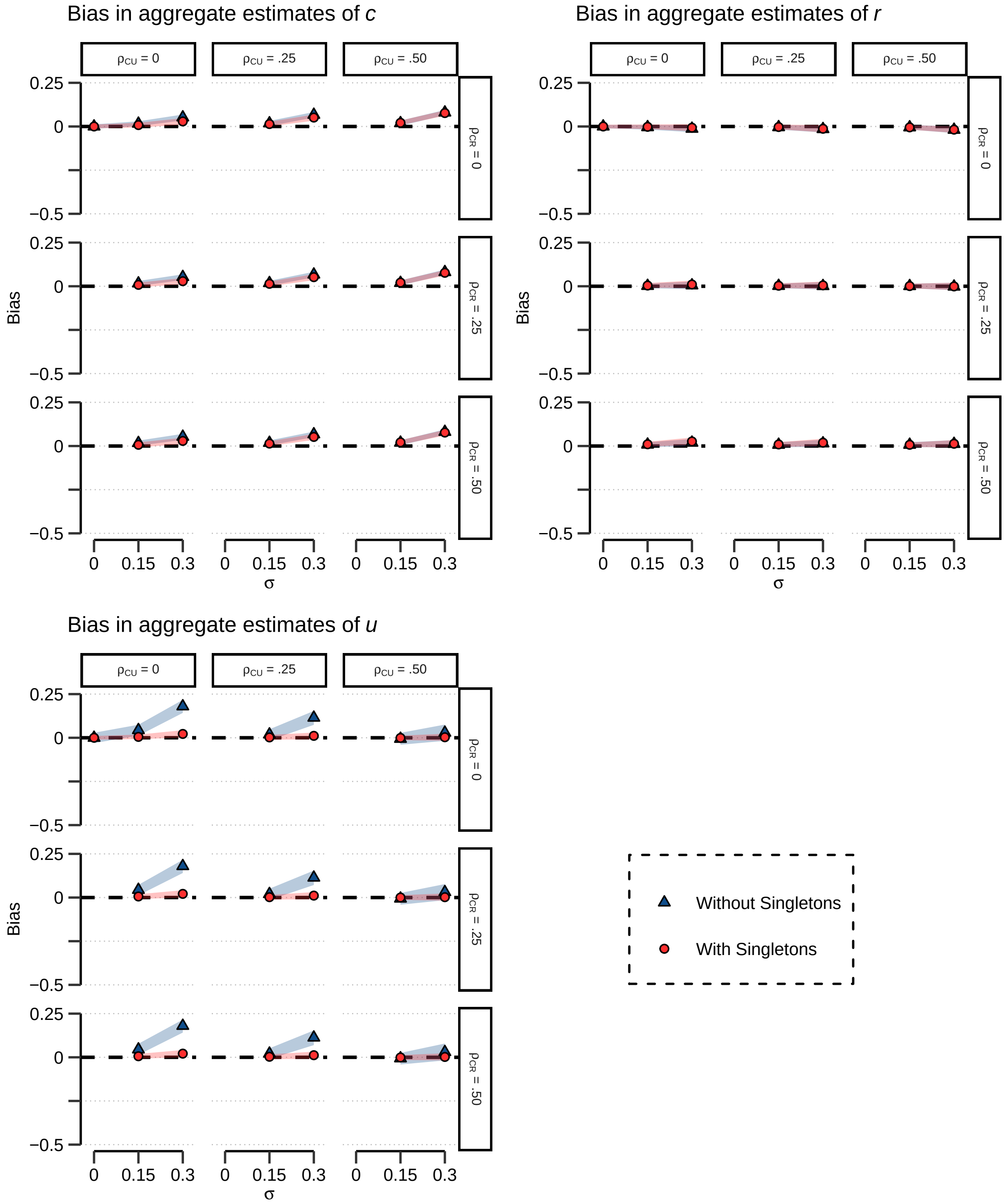

Figure 9: $N = 1000$, $m_1 = 8$, $E(C) = .80$, $E(R) = .20$

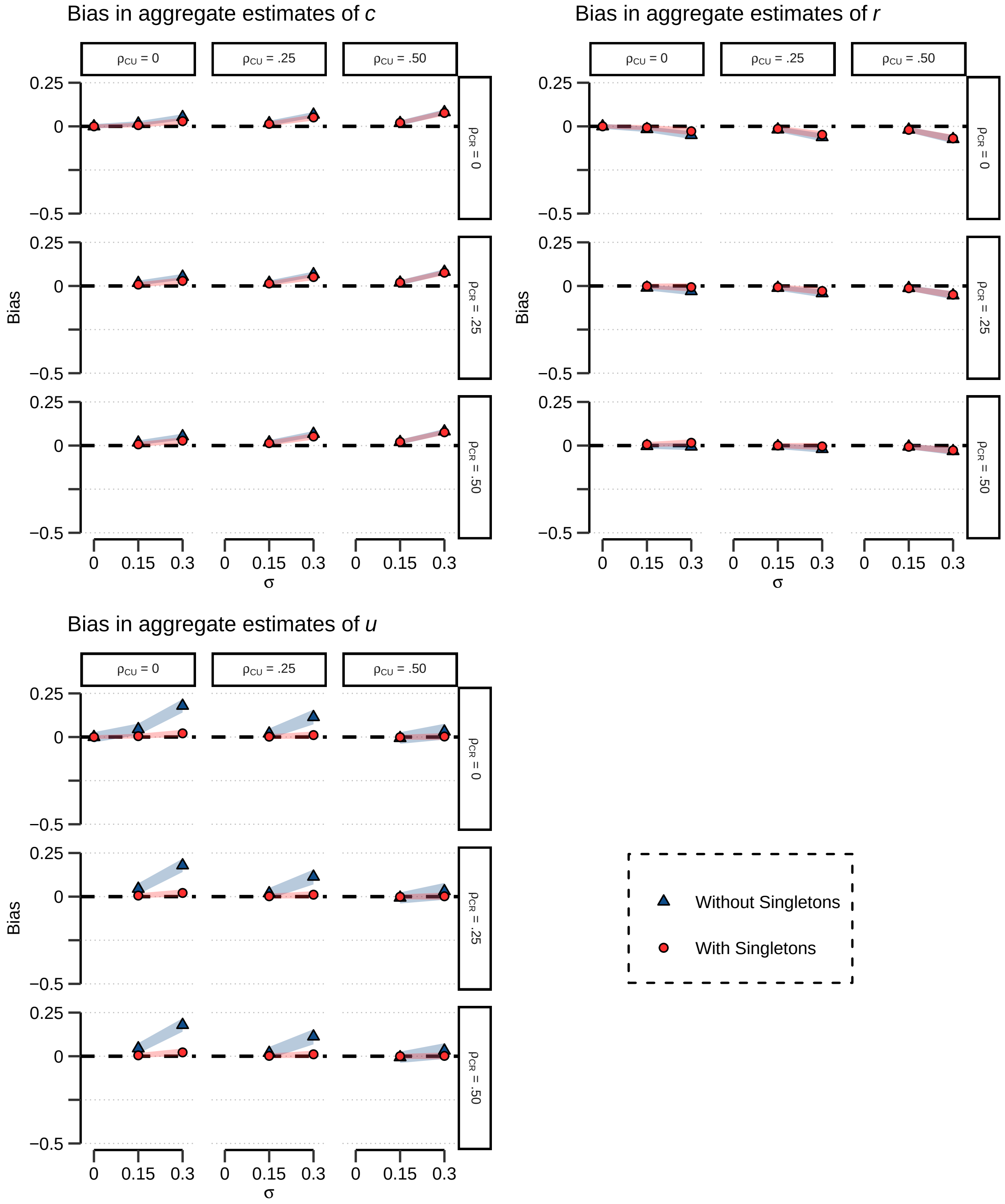

Figure 10: $N = 1000$, $m_1 = 8$, $E(C) = E(R) = .80$

Single Participants

Figures 11–20 visualize the bias for **single-participant settings** ($N = 1$) with varying numbers of responses per participant. In **Figures 11–15**, the number of word pairs is fixed at $m_1 = 20$. Expected values of C and R vary as follows:

- Figure 11: $E(C) = E(R) = .20$
- Figure 12: $E(C) = .20$, $E(R) = .80$
- Figure 13: $E(C) = E(R) = .50$
- Figure 14: $E(C) = .80, E(R) = .20$
- Figure 15: $E(C) = E(R) = .80$

In **Figures 16–20**, the number of word pairs is fixed at $m_1 = 8$. Expected values of C and R vary as follows:

- Figure 16: $E(C) = E(R) = .20$
- Figure 17: $E(C) = .20$, $E(R) = .80$
- Figure 18: $E(C) = E(R) = .50$
- Figure 19: $E(C) = .80, E(R) = .20$
- Figure 20: $E(C) = E(R) = .80$

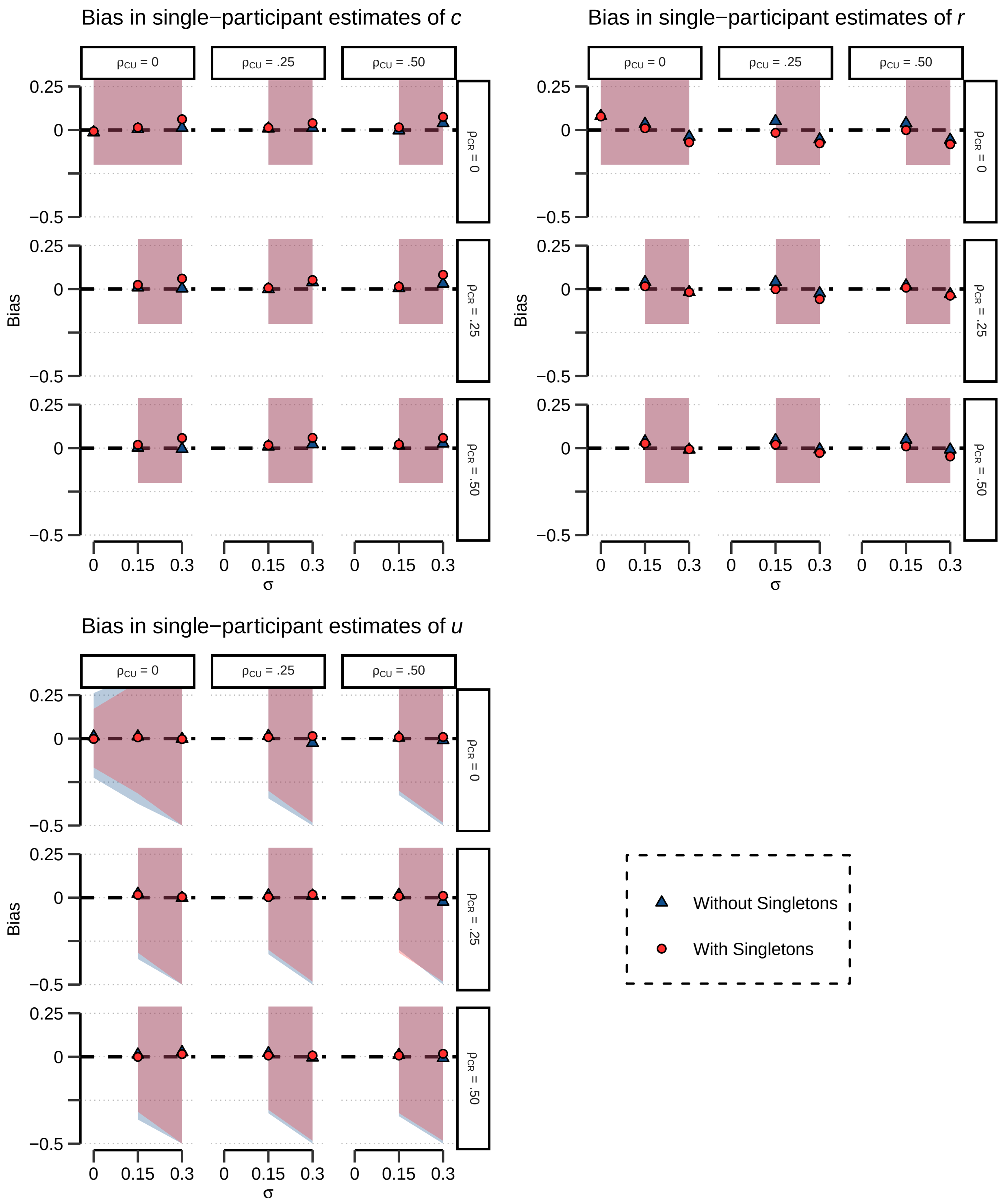

Figure 11: $N = 1$, $m_1 = 20$, $E(C) = E(R) = .20$

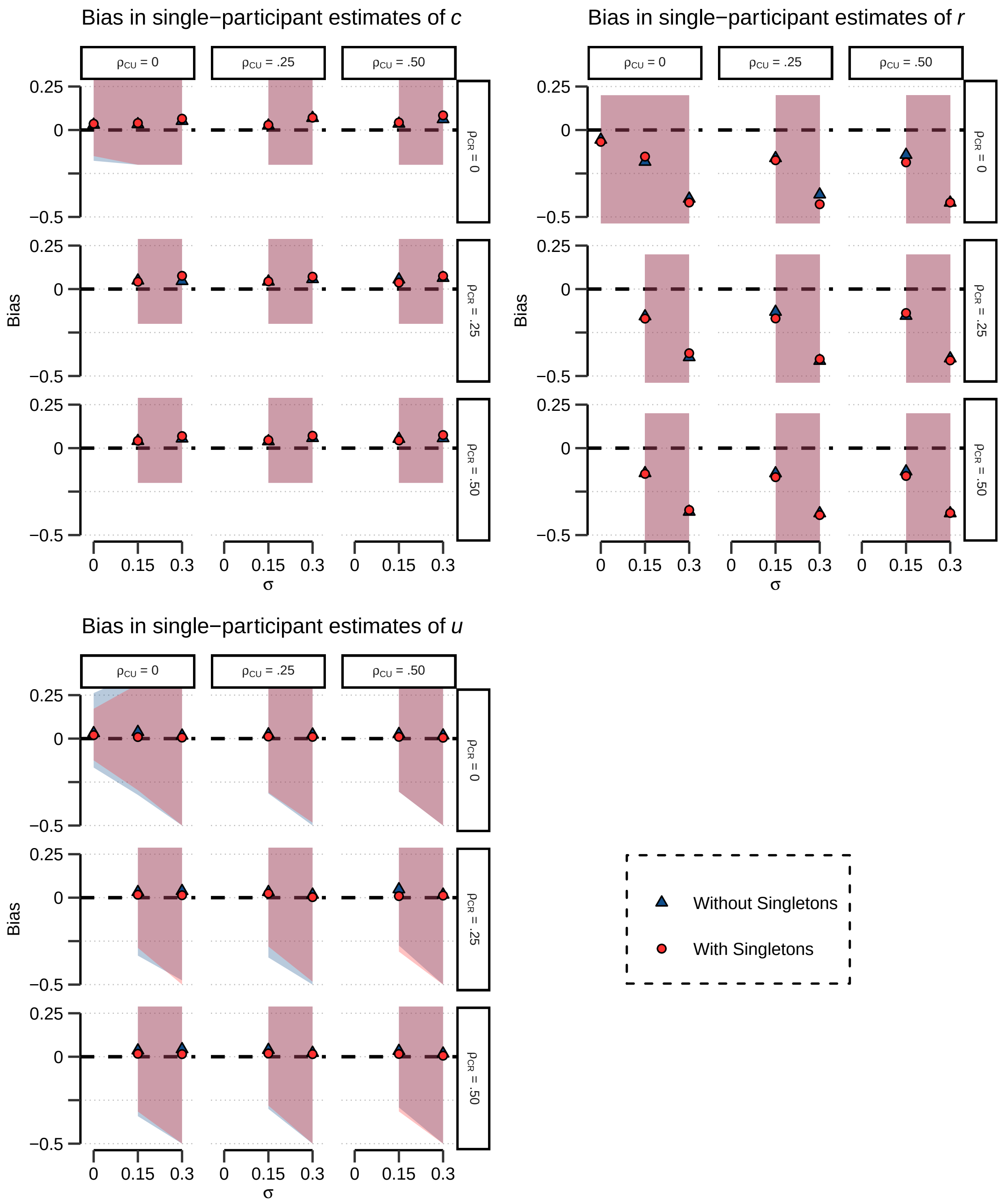

Figure 12: $N = 1$, $m_1 = 20$, $E(C) = .20$, $E(R) = .80$

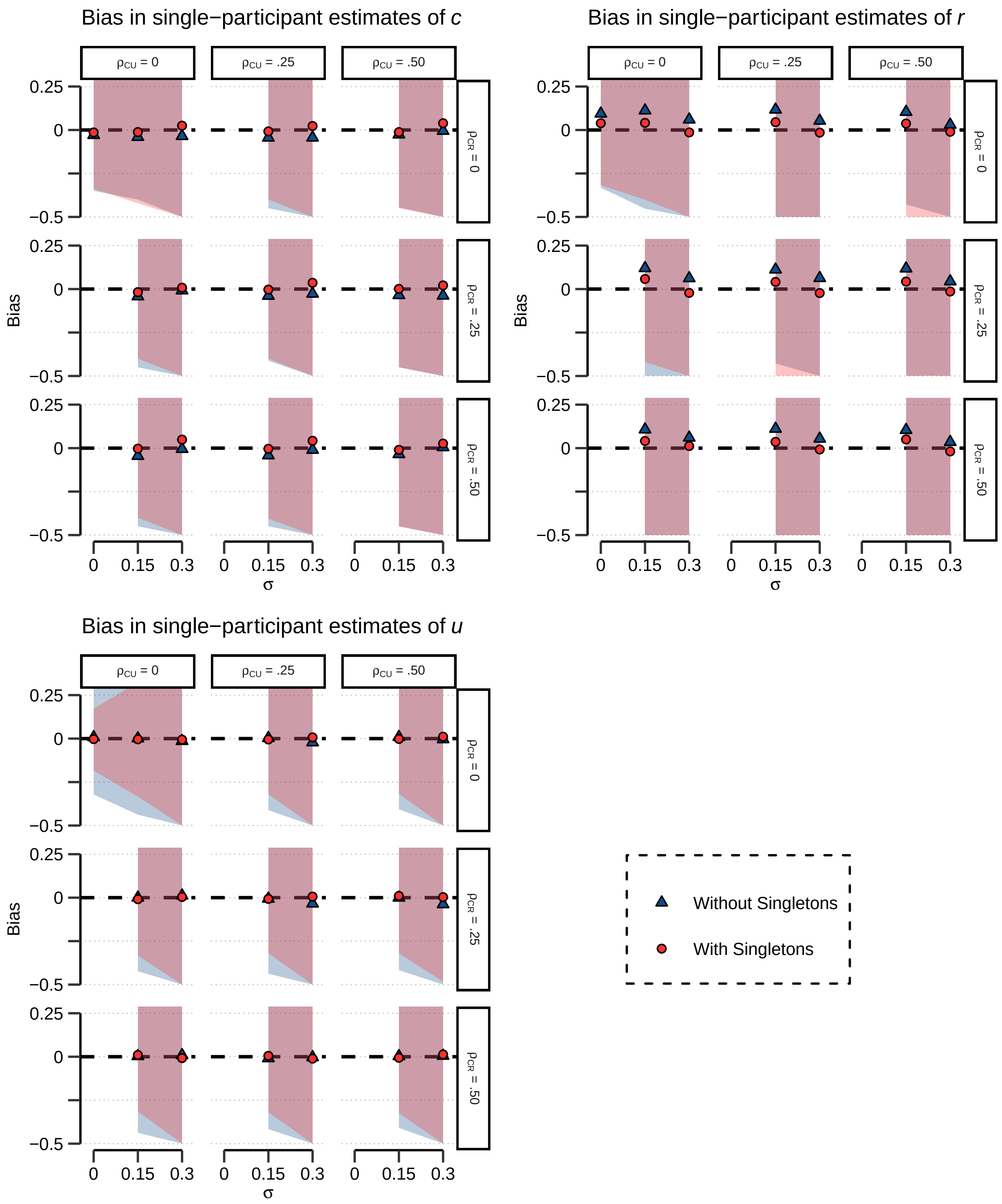

Figure 13: $N = 1$, $m_1 = 20$, $E(C) = E(R) = .50$

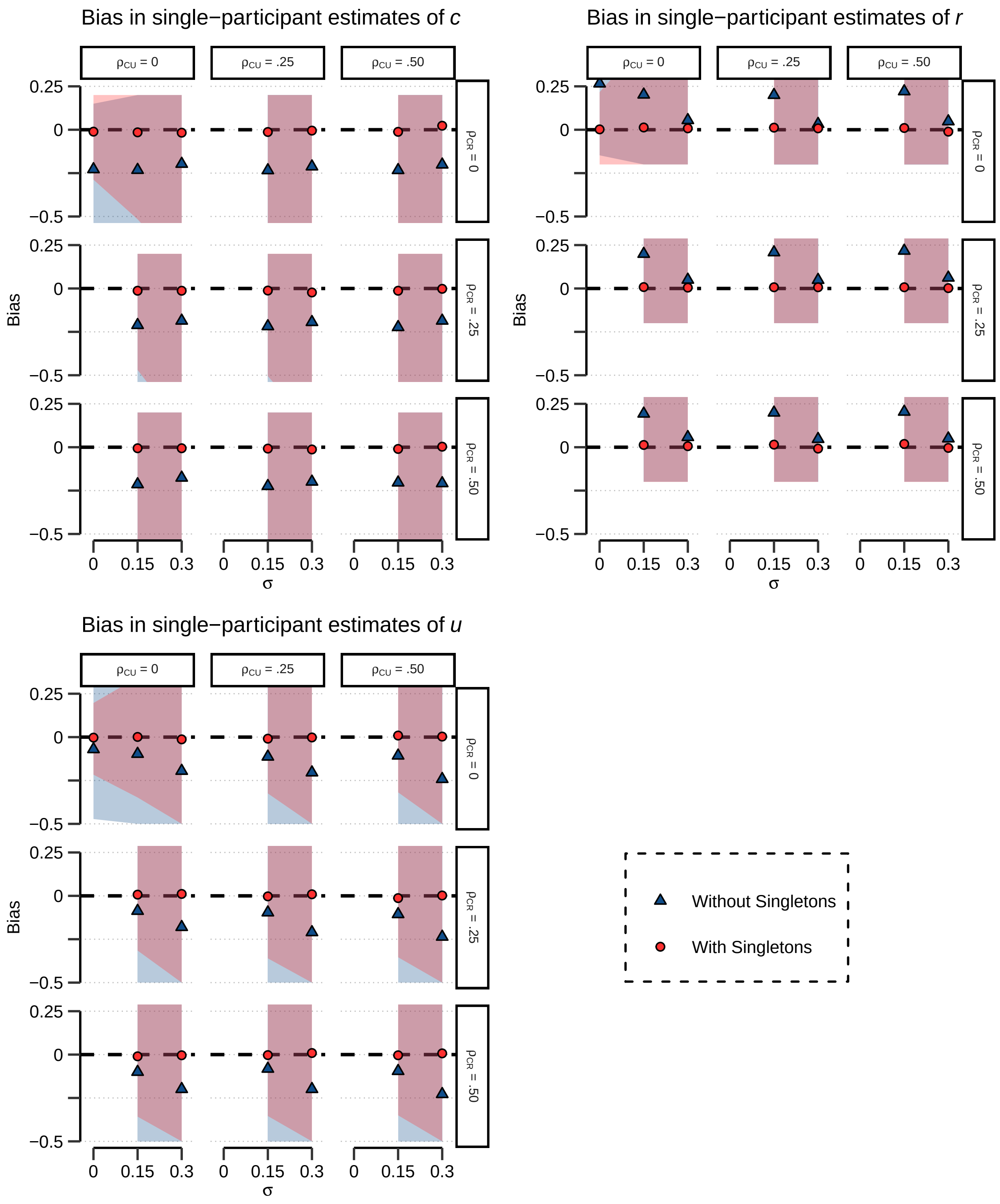

Figure 14: $N = 1$, $m_1 = 20$, $E(C) = .80$, $E(R) = .20$

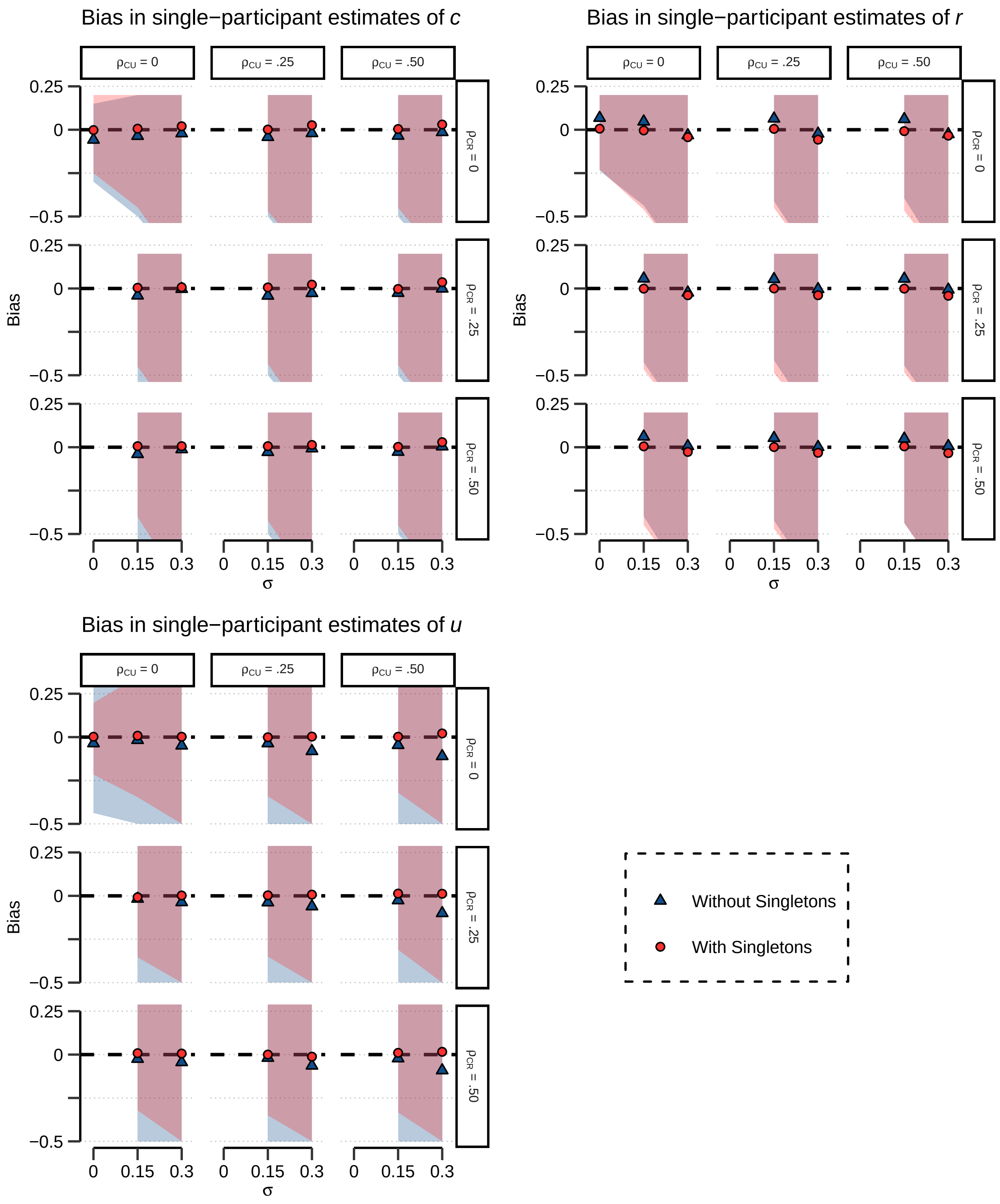

Figure 15: $N = 1$, $m_1 = 20$, $E(C) = .80$, $E(R) = .80$

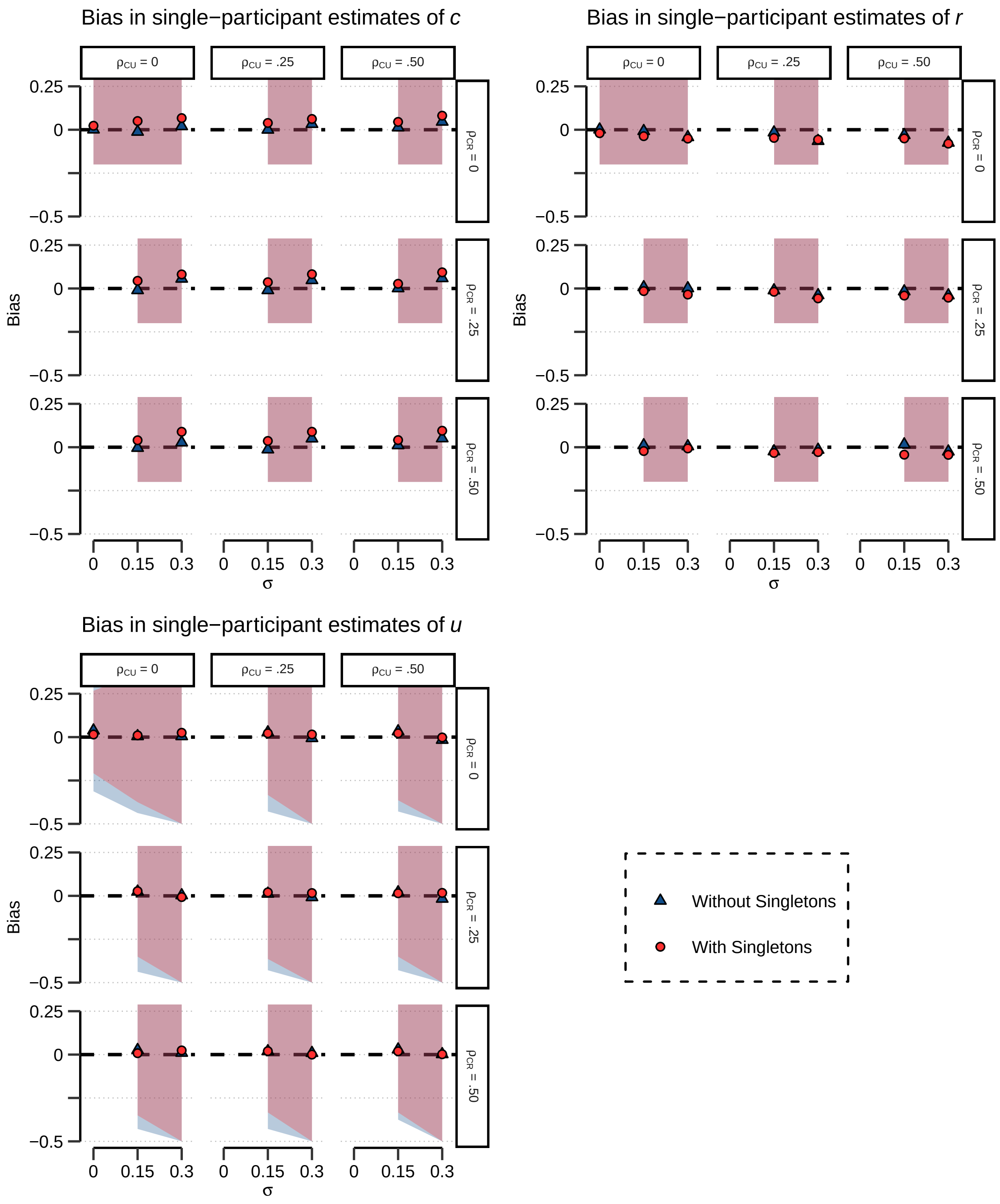

Figure 16: $N = 1$, $m_1 = 8$, $E(C) = E(R) = .20$

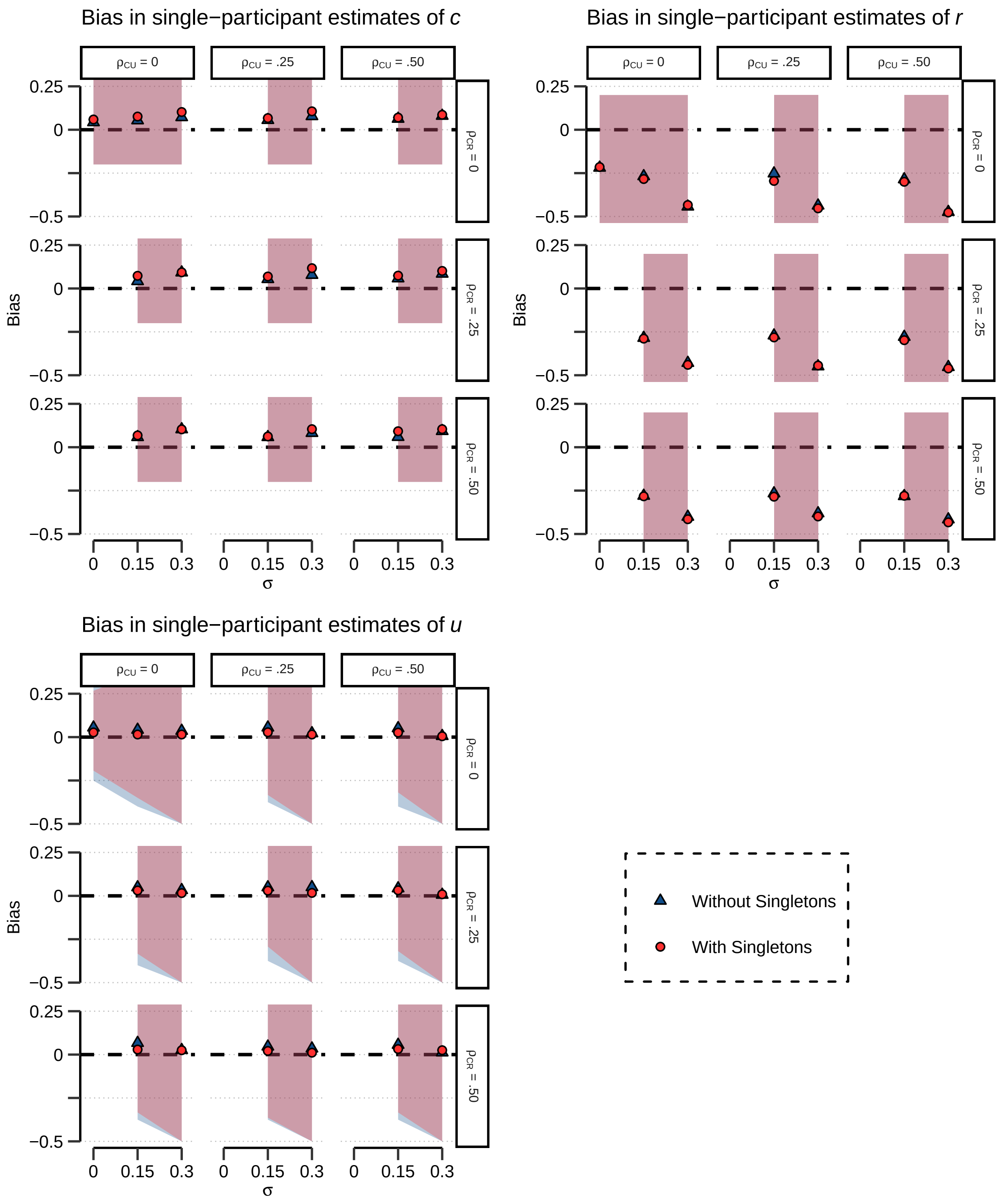

Figure 17: $N = 1$, $m_1 = 8$, $E(C) = .20$, $E(R) = .80$

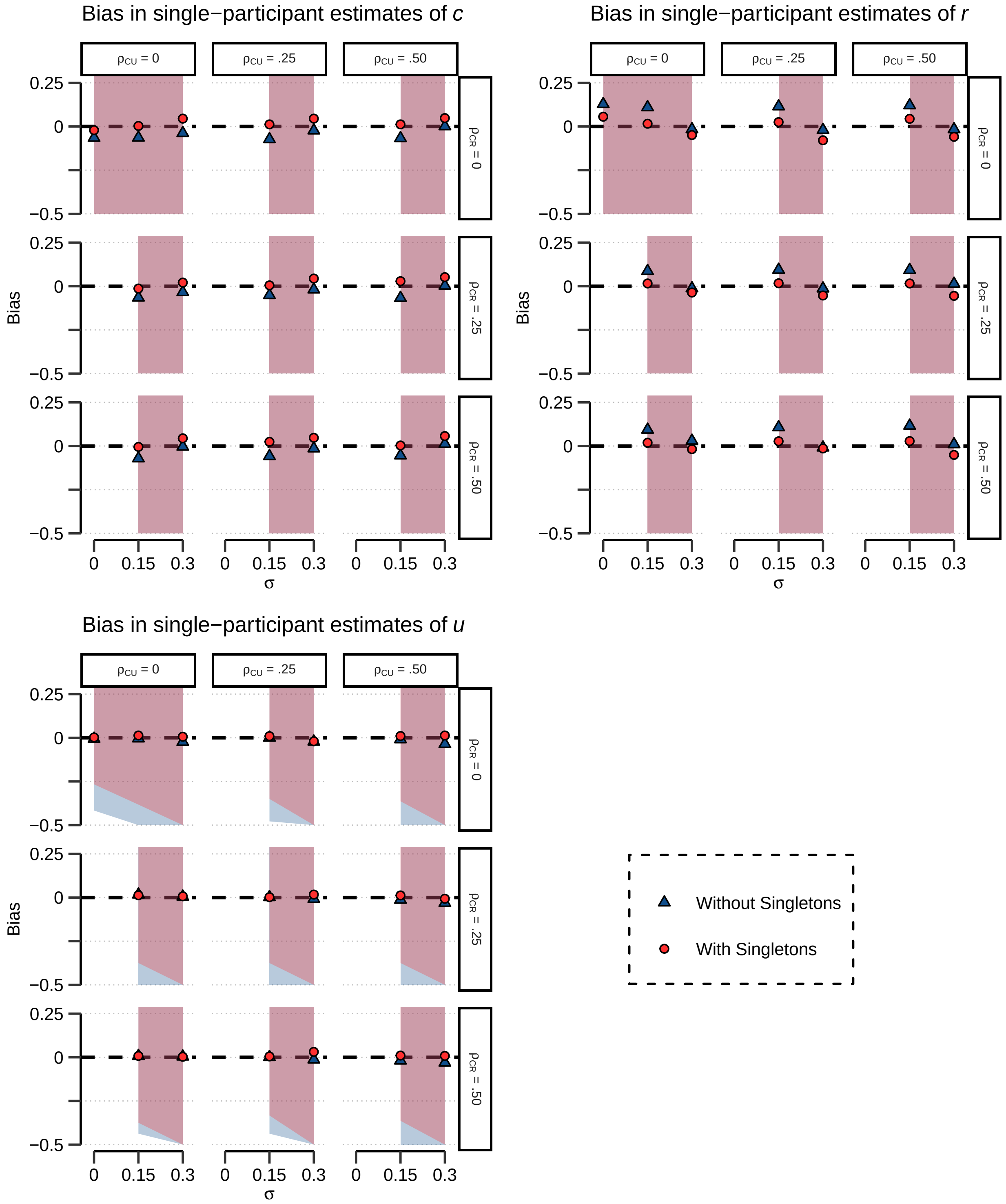

Figure 18: $N = 1$, $m_1 = 8$, $E(C) = E(R) = .50$

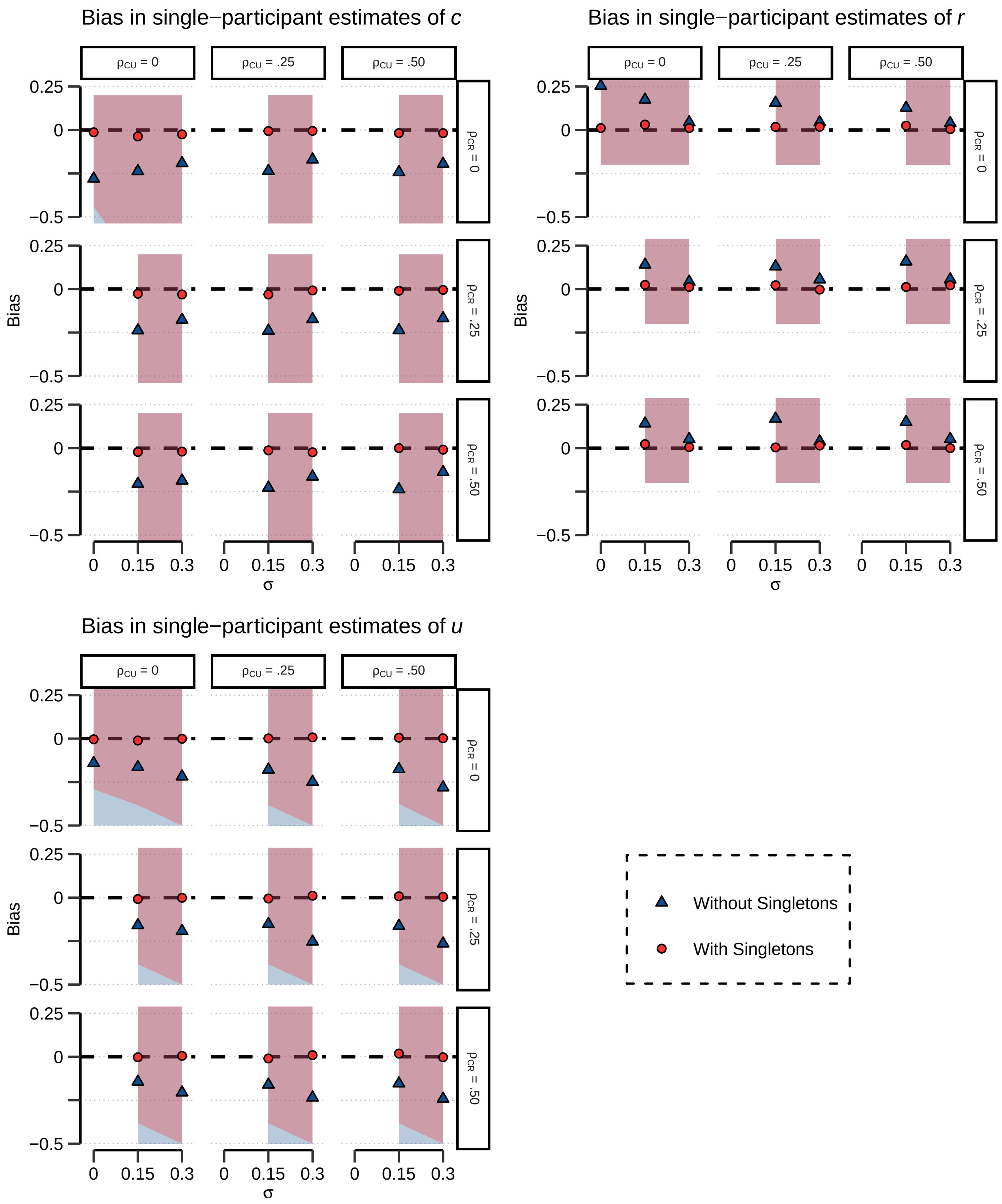

Figure 19: $N = 1$, $m_1 = 8$, $E(C) = .80$, $E(R) = .20$

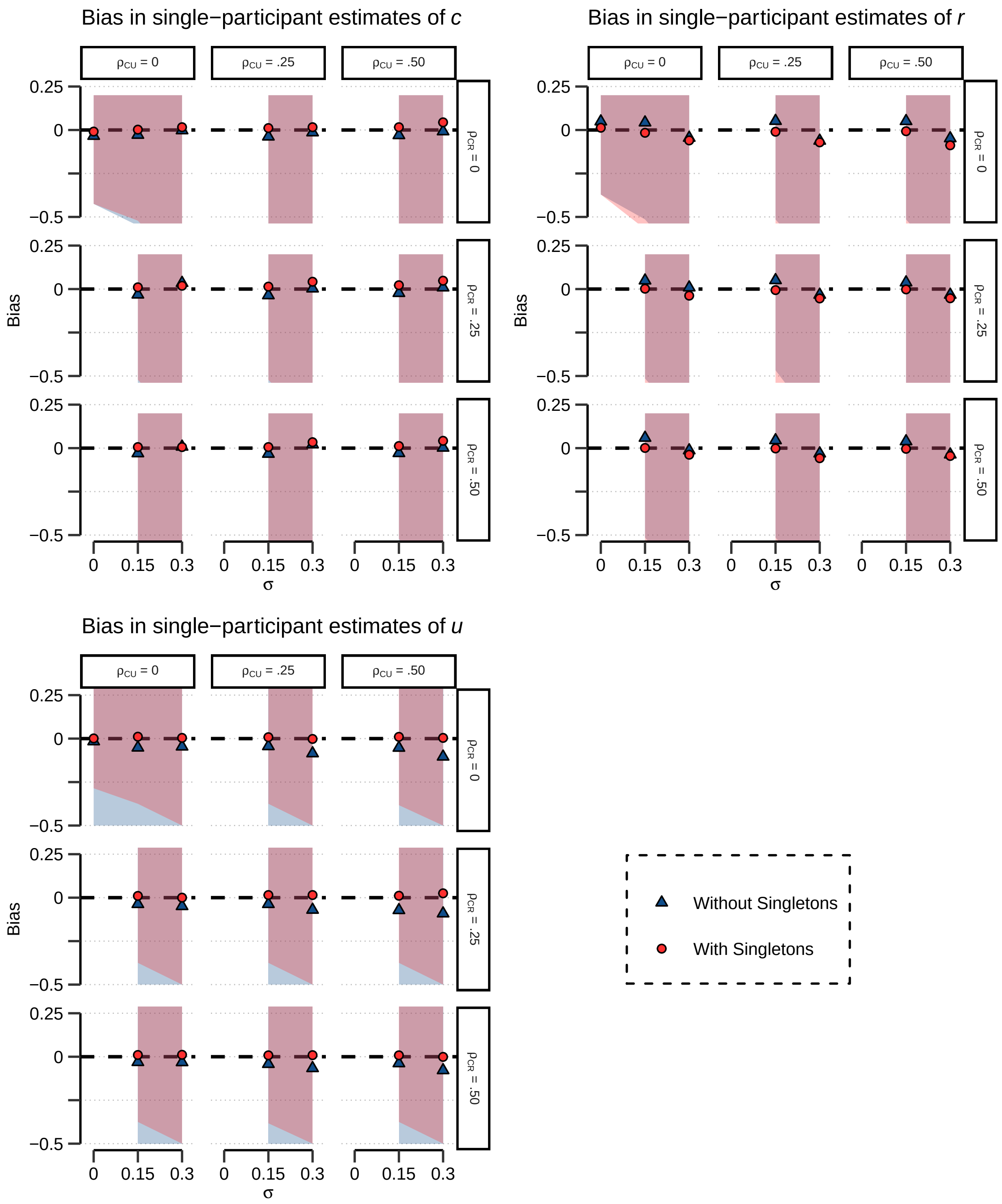

Figure 20: $N = 1$, $m_1 = 8$, $E(C) = .80$, $E(R) = .80$

Rejection Rates

Based on simulated data from the 3-parameter latent-trait version of the pair-clustering model, Figures 21–23 display observed rejection rates under the null hypothesis (at $\alpha = .05$) for three different goodness-of-fit test methods:

1. *Aggregate:* G^2 goodness-of-fit tests are performed for data aggregated across individuals.
2. *Sum:* G^2 goodness-of-fit tests are performed separately for each individual n and resulting $G^2_{(n)}$ statistics are added up, with $\sum_{n=1}^{N} G^2_{(n)}(\text{df})$ assumed to be $\chi^2(N \cdot \text{df})$-distributed.
3. *Parametric Bootstrap:* Based on individual parameter estimates, $k = 500$ data sets are simulated from the fitted model. For each bootstrap sample, the G^2 sum statistic is calculated to obtain the distribution and bootstrapped p-value for the observed G^2 sum statistic.

Results are shown as a function of method, ρ_{CU}, and sample size, based on 1,000 simulations per scenario. Note that ρ_{CR} had negligible effects on rejection rates and is thus not systematically manipulated in these plots. The number of items (i.e., word pairs and singletons) varies across figures. Expected values of all three parameters are fixed at $E(C) = E(R) = E(U) = .50$, and the correlations between R and U and between C and R are fixed at $\rho_{RU} = \rho_{CR} = 0$. Note that in all figures, confidence bands (i.e., line widths) denote 95% Clopper-Pearson confidence intervals, and dotted lines illustrate expected rejection rates for $\alpha = .05$.

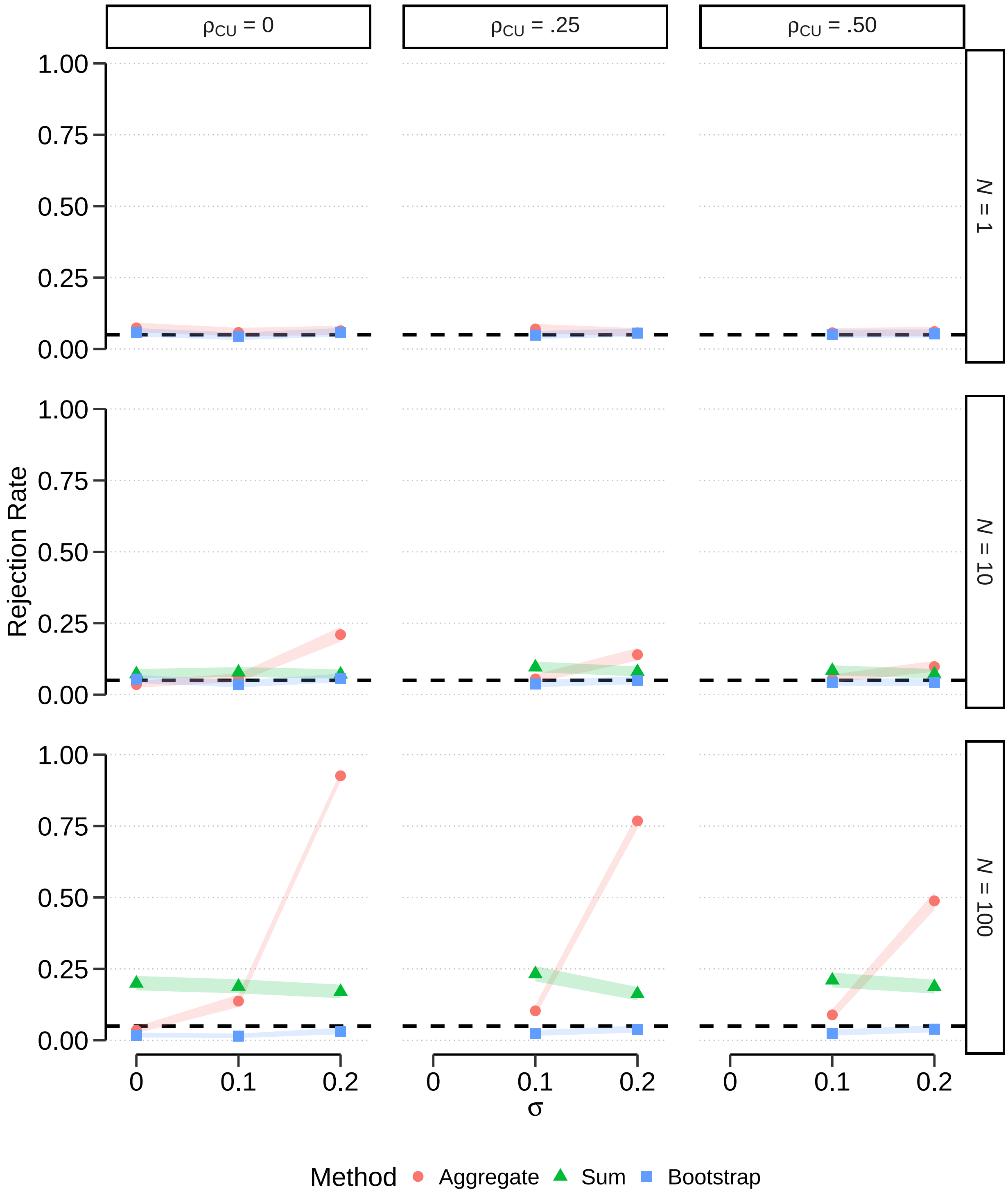

Figure 21: Rejection rate for $m_1 = m_2 = 20$ word pairs and singletons, respectively

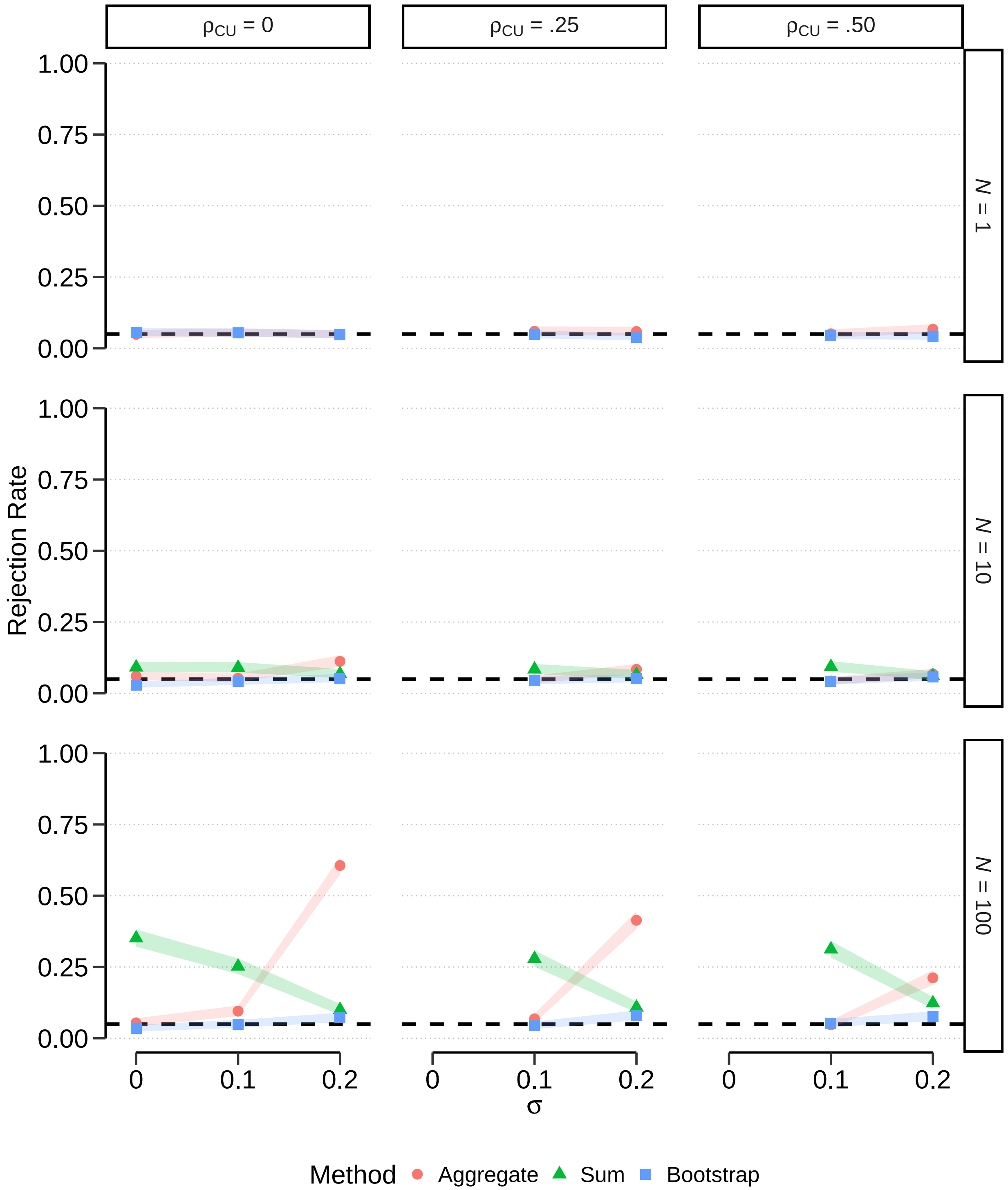

Figure 22: Rejection rate for $m_1 = m_2 = 8$ word pairs and singletons, respectively

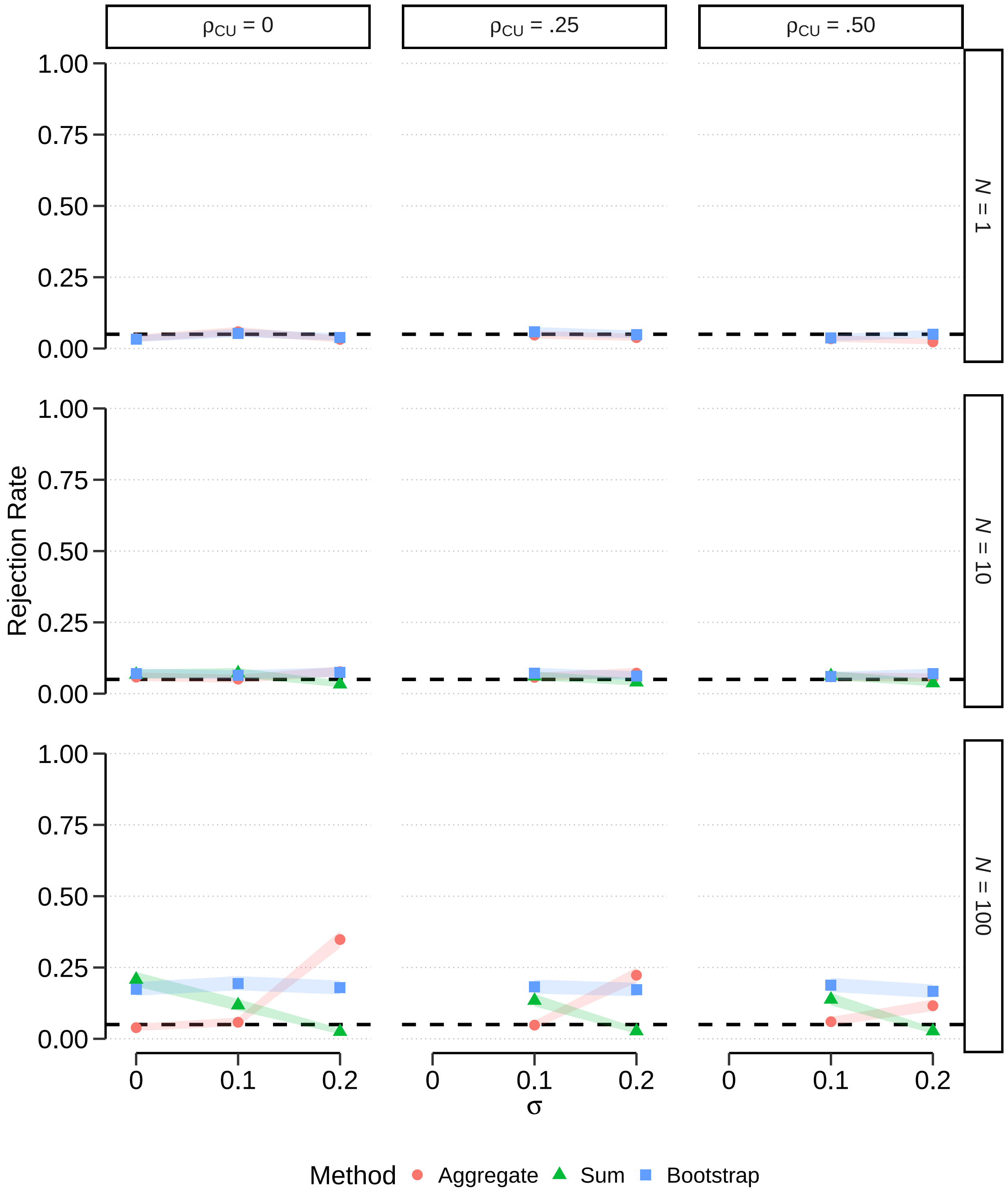

Figure 23: Rejection rate for $m_1 = m_2 = 4$ word pairs and singletons, respectively
