# Supplementary material for: On aggregation invariance of multinomial processing tree models
Source: Behav Res Methods. 2024 Oct 14;56(8):8677–94. doi: 10.3758/s13428-024-02497-y (PMC11525265; doi:10.3758/s13428-024-02497-y)
Supplement: Supplementary file 2 — (pdf 460 KB) [file 13428_2024_2497_MOESM2_ESM.pdf]

On Aggregation Invariance of Multinomial Processing Tree Models

Supplemental Material II: Full Table of Simulation Results (3-Parameter Model)

Edgar Erdfelder, Julian Quevedo Pütter, & Martin Schnuerch

Table 1

Full table of results of the Monte Carlo simulation for the 3-parameter pair-clustering model comprising 1,000 replications per parameter combination. Results display the mean bias in aggregate estimates of the model parameters c, r, and u.

												Mean Bias		
N	m_1	$\frac{m_2}{m_1}$	$E(C)$	$E(R)$	$E(U)$	σ_C	σ_R	σ_U	ρ_{CR}	ρ_{CU}	ρ_{RU}	c	r	u
1	4	0	0.2	0.2	0.5	0.00	0.00	0.00	0.00	0.00	0.00	0.001	-0.045	0.025
1	4	0	0.2	0.2	0.5	0.15	0.15	0.15	0.00	0.00	0.00	-0.008	-0.040	0.030
1	4	0	0.2	0.2	0.5	0.15	0.15	0.15	0.00	0.00	0.25	-0.017	-0.059	0.038
1	4	0	0.2	0.2	0.5	0.15	0.15	0.15	0.00	0.00	0.50	0.002	-0.063	0.030
1	4	0	0.2	0.2	0.5	0.15	0.15	0.15	0.00	0.25	0.00	-0.003	-0.053	0.034
1	4	0	0.2	0.2	0.5	0.15	0.15	0.15	0.00	0.25	0.25	-0.006	-0.050	0.031
1	4	0	0.2	0.2	0.5	0.15	0.15	0.15	0.00	0.25	0.50	0.002	-0.042	0.017
1	4	0	0.2	0.2	0.5	0.15	0.15	0.15	0.00	0.50	0.00	-0.018	-0.043	0.025
1	4	0	0.2	0.2	0.5	0.15	0.15	0.15	0.00	0.50	0.25	-0.002	-0.047	0.030
1	4	0	0.2	0.2	0.5	0.15	0.15	0.15	0.00	0.50	0.50	-0.014	-0.047	0.019
1	4	0	0.2	0.2	0.5	0.15	0.15	0.15	0.25	0.00	0.00	-0.011	-0.023	0.009
1	4	0	0.2	0.2	0.5	0.15	0.15	0.15	0.25	0.00	0.25	-0.006	-0.026	0.015
1	4	0	0.2	0.2	0.5	0.15	0.15	0.15	0.25	0.00	0.50	-0.016	-0.020	0.007
1	4	0	0.2	0.2	0.5	0.15	0.15	0.15	0.25	0.25	0.00	-0.008	-0.037	0.031
1	4	0	0.2	0.2	0.5	0.15	0.15	0.15	0.25	0.25	0.25	-0.001	-0.033	0.031
1	4	0	0.2	0.2	0.5	0.15	0.15	0.15	0.25	0.25	0.50	-0.003	-0.032	0.035
1	4	0	0.2	0.2	0.5	0.15	0.15	0.15	0.25	0.50	0.00	0.011	-0.038	0.028
1	4	0	0.2	0.2	0.5	0.15	0.15	0.15	0.25	0.50	0.25	-0.013	-0.042	0.017
1	4	0	0.2	0.2	0.5	0.15	0.15	0.15	0.25	0.50	0.50	0.001	-0.028	0.022
1	4	0	0.2	0.2	0.5	0.15	0.15	0.15	0.50	0.00	0.00	0.014	-0.024	0.028
1	4	0	0.2	0.2	0.5	0.15	0.15	0.15	0.50	0.00	0.25	0.002	-0.019	0.028
1	4	0	0.2	0.2	0.5	0.15	0.15	0.15	0.50	0.00	0.50	0.005	-0.026	0.043
1	4	0	0.2	0.2	0.5	0.15	0.15	0.15	0.50	0.25	0.00	-0.001	-0.010	0.013
1	4	0	0.2	0.2	0.5	0.15	0.15	0.15	0.50	0.25	0.25	0.008	-0.030	0.034
1	4	0	0.2	0.2	0.5	0.15	0.15	0.15	0.50	0.25	0.50	0.001	-0.028	0.016

(continued)

												Mean Bias		
N	m_1	$\frac{m_2}{m_1}$	$E(C)$	$E(R)$	$E(U)$	σ_C	σ_R	σ_U	ρ_{CR}	ρ_{CU}	ρ_{RU}	c	r	u
1	4	0	0.2	0.2	0.5	0.15	0.15	0.15	0.50	0.50	0.00	0.003	-0.032	0.023
1	4	0	0.2	0.2	0.5	0.15	0.15	0.15	0.50	0.50	0.25	-0.008	-0.009	0.019
1	4	0	0.2	0.2	0.5	0.15	0.15	0.15	0.50	0.50	0.50	0.005	-0.031	0.029
1	4	0	0.2	0.2	0.5	0.30	0.30	0.30	0.00	0.00	0.00	0.070	-0.043	0.000
1	4	0	0.2	0.2	0.5	0.30	0.30	0.30	0.00	0.00	0.25	0.046	-0.043	-0.002
1	4	0	0.2	0.2	0.5	0.30	0.30	0.30	0.00	0.00	0.50	0.063	-0.052	-0.009
1	4	0	0.2	0.2	0.5	0.30	0.30	0.30	0.00	0.25	0.00	0.065	-0.053	-0.016
1	4	0	0.2	0.2	0.5	0.30	0.30	0.30	0.00	0.25	0.25	0.059	-0.058	-0.008
1	4	0	0.2	0.2	0.5	0.30	0.30	0.30	0.00	0.25	0.50	0.042	-0.048	-0.029
1	4	0	0.2	0.2	0.5	0.30	0.30	0.30	0.00	0.50	0.00	0.081	-0.049	-0.025
1	4	0	0.2	0.2	0.5	0.30	0.30	0.30	0.00	0.50	0.25	0.074	-0.051	-0.024
1	4	0	0.2	0.2	0.5	0.30	0.30	0.30	0.00	0.50	0.50	0.061	-0.048	-0.006
1	4	0	0.2	0.2	0.5	0.30	0.30	0.30	0.25	0.00	0.00	0.060	-0.024	0.001
1	4	0	0.2	0.2	0.5	0.30	0.30	0.30	0.25	0.00	0.25	0.046	0.003	0.000
1	4	0	0.2	0.2	0.5	0.30	0.30	0.30	0.25	0.00	0.50	0.065	-0.007	-0.007
1	4	0	0.2	0.2	0.5	0.30	0.30	0.30	0.25	0.25	0.00	0.048	-0.022	-0.016
1	4	0	0.2	0.2	0.5	0.30	0.30	0.30	0.25	0.25	0.25	0.077	-0.032	0.004
1	4	0	0.2	0.2	0.5	0.30	0.30	0.30	0.25	0.25	0.50	0.074	-0.028	-0.018
1	4	0	0.2	0.2	0.5	0.30	0.30	0.30	0.25	0.50	0.00	0.068	-0.037	-0.008
1	4	0	0.2	0.2	0.5	0.30	0.30	0.30	0.25	0.50	0.25	0.049	-0.051	-0.015
1	4	0	0.2	0.2	0.5	0.30	0.30	0.30	0.25	0.50	0.50	0.063	-0.029	-0.026
1	4	0	0.2	0.2	0.5	0.30	0.30	0.30	0.50	0.00	0.00	0.079	-0.002	0.007
1	4	0	0.2	0.2	0.5	0.30	0.30	0.30	0.50	0.00	0.25	0.069	0.020	0.005
1	4	0	0.2	0.2	0.5	0.30	0.30	0.30	0.50	0.00	0.50	0.064	-0.008	-0.006
1	4	0	0.2	0.2	0.5	0.30	0.30	0.30	0.50	0.25	0.00	0.074	-0.018	-0.004
1	4	0	0.2	0.2	0.5	0.30	0.30	0.30	0.50	0.25	0.25	0.087	0.008	0.000
1	4	0	0.2	0.2	0.5	0.30	0.30	0.30	0.50	0.25	0.50	0.056	0.009	0.021
1	4	0	0.2	0.2	0.5	0.30	0.30	0.30	0.50	0.50	0.00	0.086	-0.006	-0.006
1	4	0	0.2	0.2	0.5	0.30	0.30	0.30	0.50	0.50	0.25	0.094	-0.005	-0.014
1	4	0	0.2	0.2	0.5	0.30	0.30	0.30	0.50	0.50	0.50	0.062	-0.026	-0.017
1	4	0	0.2	0.5	0.5	0.00	0.00	0.00	0.00	0.00	0.00	0.041	-0.170	0.060
1	4	0	0.2	0.5	0.5	0.15	0.15	0.15	0.00	0.00	0.00	0.026	-0.198	0.039
1	4	0	0.2	0.5	0.5	0.15	0.15	0.15	0.00	0.00	0.25	0.047	-0.185	0.028
1	4	0	0.2	0.5	0.5	0.15	0.15	0.15	0.00	0.00	0.50	0.026	-0.208	0.053
1	4	0	0.2	0.5	0.5	0.15	0.15	0.15	0.00	0.25	0.00	0.035	-0.190	0.040
1	4	0	0.2	0.5	0.5	0.15	0.15	0.15	0.00	0.25	0.25	0.023	-0.209	0.024
1	4	0	0.2	0.5	0.5	0.15	0.15	0.15	0.00	0.25	0.50	0.026	-0.199	0.037
1	4	0	0.2	0.5	0.5	0.15	0.15	0.15	0.00	0.50	0.00	0.017	-0.219	0.040
1	4	0	0.2	0.5	0.5	0.15	0.15	0.15	0.00	0.50	0.25	0.027	-0.217	0.014

(continued)

												Mean Bias		
N	m_1	$\frac{m_2}{m_1}$	$E(C)$	$E(R)$	$E(U)$	σ_C	σ_R	σ_U	ρ_{CR}	ρ_{CU}	ρ_{RU}	c	r	u
1	4	0	0.2	0.5	0.5	0.15	0.15	0.15	0.00	0.50	0.50	0.035	-0.222	0.029
1	4	0	0.2	0.5	0.5	0.15	0.15	0.15	0.25	0.00	0.00	0.035	-0.189	0.053
1	4	0	0.2	0.5	0.5	0.15	0.15	0.15	0.25	0.00	0.25	0.031	-0.220	0.041
1	4	0	0.2	0.5	0.5	0.15	0.15	0.15	0.25	0.00	0.50	0.032	-0.191	0.036
1	4	0	0.2	0.5	0.5	0.15	0.15	0.15	0.25	0.25	0.00	0.027	-0.197	0.037
1	4	0	0.2	0.5	0.5	0.15	0.15	0.15	0.25	0.25	0.25	0.027	-0.192	0.042
1	4	0	0.2	0.5	0.5	0.15	0.15	0.15	0.25	0.25	0.50	0.033	-0.211	0.038
1	4	0	0.2	0.5	0.5	0.15	0.15	0.15	0.25	0.50	0.00	0.035	-0.204	0.046
1	4	0	0.2	0.5	0.5	0.15	0.15	0.15	0.25	0.50	0.25	0.034	-0.198	0.032
1	4	0	0.2	0.5	0.5	0.15	0.15	0.15	0.25	0.50	0.50	0.047	-0.211	0.041
1	4	0	0.2	0.5	0.5	0.15	0.15	0.15	0.50	0.00	0.00	0.048	-0.181	0.047
1	4	0	0.2	0.5	0.5	0.15	0.15	0.15	0.50	0.00	0.25	0.034	-0.211	0.035
1	4	0	0.2	0.5	0.5	0.15	0.15	0.15	0.50	0.00	0.50	0.023	-0.187	0.035
1	4	0	0.2	0.5	0.5	0.15	0.15	0.15	0.50	0.25	0.00	0.039	-0.191	0.050
1	4	0	0.2	0.5	0.5	0.15	0.15	0.15	0.50	0.25	0.25	0.044	-0.189	0.052
1	4	0	0.2	0.5	0.5	0.15	0.15	0.15	0.50	0.25	0.50	0.029	-0.183	0.049
1	4	0	0.2	0.5	0.5	0.15	0.15	0.15	0.50	0.50	0.00	0.032	-0.207	0.042
1	4	0	0.2	0.5	0.5	0.15	0.15	0.15	0.50	0.50	0.25	0.044	-0.183	0.038
1	4	0	0.2	0.5	0.5	0.15	0.15	0.15	0.50	0.50	0.50	0.020	-0.190	0.017
1	4	0	0.2	0.5	0.5	0.30	0.30	0.30	0.00	0.00	0.00	0.088	-0.261	0.012
1	4	0	0.2	0.5	0.5	0.30	0.30	0.30	0.00	0.00	0.25	0.090	-0.250	0.014
1	4	0	0.2	0.5	0.5	0.30	0.30	0.30	0.00	0.00	0.50	0.064	-0.234	0.014
1	4	0	0.2	0.5	0.5	0.30	0.30	0.30	0.00	0.25	0.00	0.083	-0.267	0.014
1	4	0	0.2	0.5	0.5	0.30	0.30	0.30	0.00	0.25	0.25	0.082	-0.241	0.036
1	4	0	0.2	0.5	0.5	0.30	0.30	0.30	0.00	0.25	0.50	0.074	-0.237	-0.002
1	4	0	0.2	0.5	0.5	0.30	0.30	0.30	0.00	0.50	0.00	0.088	-0.262	-0.009
1	4	0	0.2	0.5	0.5	0.30	0.30	0.30	0.00	0.50	0.25	0.075	-0.265	0.002
1	4	0	0.2	0.5	0.5	0.30	0.30	0.30	0.00	0.50	0.50	0.082	-0.238	-0.002
1	4	0	0.2	0.5	0.5	0.30	0.30	0.30	0.25	0.00	0.00	0.087	-0.228	0.016
1	4	0	0.2	0.5	0.5	0.30	0.30	0.30	0.25	0.00	0.25	0.096	-0.217	0.043
1	4	0	0.2	0.5	0.5	0.30	0.30	0.30	0.25	0.00	0.50	0.078	-0.219	0.043
1	4	0	0.2	0.5	0.5	0.30	0.30	0.30	0.25	0.25	0.00	0.075	-0.241	0.000
1	4	0	0.2	0.5	0.5	0.30	0.30	0.30	0.25	0.25	0.25	0.099	-0.209	0.011
1	4	0	0.2	0.5	0.5	0.30	0.30	0.30	0.25	0.25	0.50	0.078	-0.237	0.007
1	4	0	0.2	0.5	0.5	0.30	0.30	0.30	0.25	0.50	0.00	0.097	-0.253	-0.019
1	4	0	0.2	0.5	0.5	0.30	0.30	0.30	0.25	0.50	0.25	0.099	-0.237	-0.001
1	4	0	0.2	0.5	0.5	0.30	0.30	0.30	0.25	0.50	0.50	0.092	-0.244	0.000
1	4	0	0.2	0.5	0.5	0.30	0.30	0.30	0.50	0.00	0.00	0.069	-0.216	0.030
1	4	0	0.2	0.5	0.5	0.30	0.30	0.30	0.50	0.00	0.25	0.077	-0.198	0.023

(continued)

												Mean Bias		
N	m_1	$\frac{m_2}{m_1}$	$E(C)$	$E(R)$	$E(U)$	σ_C	σ_R	σ_U	ρ_{CR}	ρ_{CU}	ρ_{RU}	c	r	u
1	4	0	0.2	0.5	0.5	0.30	0.30	0.30	0.50	0.00	0.50	0.092	-0.215	0.048
1	4	0	0.2	0.5	0.5	0.30	0.30	0.30	0.50	0.25	0.00	0.087	-0.225	0.021
1	4	0	0.2	0.5	0.5	0.30	0.30	0.30	0.50	0.25	0.25	0.098	-0.176	0.029
1	4	0	0.2	0.5	0.5	0.30	0.30	0.30	0.50	0.25	0.50	0.096	-0.220	0.010
1	4	0	0.2	0.5	0.5	0.30	0.30	0.30	0.50	0.50	0.00	0.114	-0.210	0.012
1	4	0	0.2	0.5	0.5	0.30	0.30	0.30	0.50	0.50	0.25	0.074	-0.212	0.007
1	4	0	0.2	0.5	0.5	0.30	0.30	0.30	0.50	0.50	0.50	0.101	-0.208	-0.004
1	4	0	0.2	0.8	0.5	0.00	0.00	0.00	0.00	0.00	0.00	0.056	-0.346	0.059
1	4	0	0.2	0.8	0.5	0.15	0.15	0.15	0.00	0.00	0.00	0.048	-0.392	0.044
1	4	0	0.2	0.8	0.5	0.15	0.15	0.15	0.00	0.00	0.25	0.072	-0.375	0.057
1	4	0	0.2	0.8	0.5	0.15	0.15	0.15	0.00	0.00	0.50	0.063	-0.374	0.063
1	4	0	0.2	0.8	0.5	0.15	0.15	0.15	0.00	0.25	0.00	0.057	-0.388	0.059
1	4	0	0.2	0.8	0.5	0.15	0.15	0.15	0.00	0.25	0.25	0.063	-0.386	0.057
1	4	0	0.2	0.8	0.5	0.15	0.15	0.15	0.00	0.25	0.50	0.066	-0.363	0.048
1	4	0	0.2	0.8	0.5	0.15	0.15	0.15	0.00	0.50	0.00	0.058	-0.369	0.054
1	4	0	0.2	0.8	0.5	0.15	0.15	0.15	0.00	0.50	0.25	0.064	-0.370	0.051
1	4	0	0.2	0.8	0.5	0.15	0.15	0.15	0.00	0.50	0.50	0.067	-0.378	0.074
1	4	0	0.2	0.8	0.5	0.15	0.15	0.15	0.25	0.00	0.00	0.052	-0.364	0.064
1	4	0	0.2	0.8	0.5	0.15	0.15	0.15	0.25	0.00	0.25	0.069	-0.391	0.064
1	4	0	0.2	0.8	0.5	0.15	0.15	0.15	0.25	0.00	0.50	0.062	-0.415	0.076
1	4	0	0.2	0.8	0.5	0.15	0.15	0.15	0.25	0.25	0.00	0.064	-0.359	0.056
1	4	0	0.2	0.8	0.5	0.15	0.15	0.15	0.25	0.25	0.25	0.061	-0.379	0.050
1	4	0	0.2	0.8	0.5	0.15	0.15	0.15	0.25	0.25	0.50	0.081	-0.358	0.079
1	4	0	0.2	0.8	0.5	0.15	0.15	0.15	0.25	0.50	0.00	0.063	-0.382	0.050
1	4	0	0.2	0.8	0.5	0.15	0.15	0.15	0.25	0.50	0.25	0.058	-0.391	0.059
1	4	0	0.2	0.8	0.5	0.15	0.15	0.15	0.25	0.50	0.50	0.050	-0.372	0.045
1	4	0	0.2	0.8	0.5	0.15	0.15	0.15	0.50	0.00	0.00	0.074	-0.376	0.056
1	4	0	0.2	0.8	0.5	0.15	0.15	0.15	0.50	0.00	0.25	0.075	-0.351	0.068
1	4	0	0.2	0.8	0.5	0.15	0.15	0.15	0.50	0.00	0.50	0.057	-0.373	0.056
1	4	0	0.2	0.8	0.5	0.15	0.15	0.15	0.50	0.25	0.00	0.061	-0.372	0.057
1	4	0	0.2	0.8	0.5	0.15	0.15	0.15	0.50	0.25	0.25	0.071	-0.366	0.055
1	4	0	0.2	0.8	0.5	0.15	0.15	0.15	0.50	0.25	0.50	0.052	-0.368	0.057
1	4	0	0.2	0.8	0.5	0.15	0.15	0.15	0.50	0.50	0.00	0.084	-0.366	0.053
1	4	0	0.2	0.8	0.5	0.15	0.15	0.15	0.50	0.50	0.25	0.051	-0.394	0.054
1	4	0	0.2	0.8	0.5	0.15	0.15	0.15	0.50	0.50	0.50	0.075	-0.358	0.053
1	4	0	0.2	0.8	0.5	0.30	0.30	0.30	0.00	0.00	0.00	0.110	-0.454	0.039
1	4	0	0.2	0.8	0.5	0.30	0.30	0.30	0.00	0.00	0.25	0.091	-0.468	0.026
1	4	0	0.2	0.8	0.5	0.30	0.30	0.30	0.00	0.00	0.50	0.093	-0.471	0.021
1	4	0	0.2	0.8	0.5	0.30	0.30	0.30	0.00	0.25	0.00	0.092	-0.473	0.009

(continued)

												Mean Bias		
N	m_1	$\frac{m_2}{m_1}$	$E(C)$	$E(R)$	$E(U)$	σ_C	σ_R	σ_U	ρ_{CR}	ρ_{CU}	ρ_{RU}	c	r	u
1	4	0	0.2	0.8	0.5	0.30	0.30	0.30	0.00	0.25	0.25	0.101	-0.464	0.019
1	4	0	0.2	0.8	0.5	0.30	0.30	0.30	0.00	0.25	0.50	0.114	-0.460	0.002
1	4	0	0.2	0.8	0.5	0.30	0.30	0.30	0.00	0.50	0.00	0.117	-0.458	0.022
1	4	0	0.2	0.8	0.5	0.30	0.30	0.30	0.00	0.50	0.25	0.128	-0.475	0.011
1	4	0	0.2	0.8	0.5	0.30	0.30	0.30	0.00	0.50	0.50	0.098	-0.463	0.027
1	4	0	0.2	0.8	0.5	0.30	0.30	0.30	0.25	0.00	0.00	0.125	-0.430	0.042
1	4	0	0.2	0.8	0.5	0.30	0.30	0.30	0.25	0.00	0.25	0.088	-0.452	0.043
1	4	0	0.2	0.8	0.5	0.30	0.30	0.30	0.25	0.00	0.50	0.089	-0.427	0.061
1	4	0	0.2	0.8	0.5	0.30	0.30	0.30	0.25	0.25	0.00	0.103	-0.458	0.031
1	4	0	0.2	0.8	0.5	0.30	0.30	0.30	0.25	0.25	0.25	0.110	-0.451	0.021
1	4	0	0.2	0.8	0.5	0.30	0.30	0.30	0.25	0.25	0.50	0.117	-0.473	0.022
1	4	0	0.2	0.8	0.5	0.30	0.30	0.30	0.25	0.50	0.00	0.103	-0.470	0.010
1	4	0	0.2	0.8	0.5	0.30	0.30	0.30	0.25	0.50	0.25	0.135	-0.450	0.024
1	4	0	0.2	0.8	0.5	0.30	0.30	0.30	0.25	0.50	0.50	0.120	-0.450	0.014
1	4	0	0.2	0.8	0.5	0.30	0.30	0.30	0.50	0.00	0.00	0.094	-0.451	0.039
1	4	0	0.2	0.8	0.5	0.30	0.30	0.30	0.50	0.00	0.25	0.104	-0.413	0.061
1	4	0	0.2	0.8	0.5	0.30	0.30	0.30	0.50	0.00	0.50	0.103	-0.435	0.044
1	4	0	0.2	0.8	0.5	0.30	0.30	0.30	0.50	0.25	0.00	0.121	-0.423	0.035
1	4	0	0.2	0.8	0.5	0.30	0.30	0.30	0.50	0.25	0.25	0.099	-0.460	0.050
1	4	0	0.2	0.8	0.5	0.30	0.30	0.30	0.50	0.25	0.50	0.099	-0.438	0.023
1	4	0	0.2	0.8	0.5	0.30	0.30	0.30	0.50	0.50	0.00	0.129	-0.431	0.020
1	4	0	0.2	0.8	0.5	0.30	0.30	0.30	0.50	0.50	0.25	0.101	-0.466	0.014
1	4	0	0.2	0.8	0.5	0.30	0.30	0.30	0.50	0.50	0.50	0.133	-0.451	0.005
1	4	0	0.5	0.2	0.5	0.00	0.00	0.00	0.00	0.00	0.00	-0.166	0.047	-0.056
1	4	0	0.5	0.2	0.5	0.15	0.15	0.15	0.00	0.00	0.00	-0.133	0.019	-0.080
1	4	0	0.5	0.2	0.5	0.15	0.15	0.15	0.00	0.00	0.25	-0.146	0.059	-0.079
1	4	0	0.5	0.2	0.5	0.15	0.15	0.15	0.00	0.00	0.50	-0.147	0.047	-0.068
1	4	0	0.5	0.2	0.5	0.15	0.15	0.15	0.00	0.25	0.00	-0.149	0.039	-0.078
1	4	0	0.5	0.2	0.5	0.15	0.15	0.15	0.00	0.25	0.25	-0.148	0.049	-0.059
1	4	0	0.5	0.2	0.5	0.15	0.15	0.15	0.00	0.25	0.50	-0.128	0.024	-0.049
1	4	0	0.5	0.2	0.5	0.15	0.15	0.15	0.00	0.50	0.00	-0.121	0.046	-0.066
1	4	0	0.5	0.2	0.5	0.15	0.15	0.15	0.00	0.50	0.25	-0.142	0.039	-0.068
1	4	0	0.5	0.2	0.5	0.15	0.15	0.15	0.00	0.50	0.50	-0.144	0.048	-0.057
1	4	0	0.5	0.2	0.5	0.15	0.15	0.15	0.25	0.00	0.00	-0.145	0.048	-0.056
1	4	0	0.5	0.2	0.5	0.15	0.15	0.15	0.25	0.00	0.25	-0.133	0.048	-0.060
1	4	0	0.5	0.2	0.5	0.15	0.15	0.15	0.25	0.00	0.50	-0.131	0.049	-0.066
1	4	0	0.5	0.2	0.5	0.15	0.15	0.15	0.25	0.25	0.00	-0.127	0.032	-0.053
1	4	0	0.5	0.2	0.5	0.15	0.15	0.15	0.25	0.25	0.25	-0.132	0.056	-0.064
1	4	0	0.5	0.2	0.5	0.15	0.15	0.15	0.25	0.25	0.50	-0.140	0.046	-0.067

(continued)

												Mean Bias		
N	m_1	$\frac{m_2}{m_1}$	$E(C)$	$E(R)$	$E(U)$	σ_C	σ_R	σ_U	ρ_{CR}	ρ_{CU}	ρ_{RU}	c	r	u
1	4	0	0.5	0.2	0.5	0.15	0.15	0.15	0.25	0.50	0.00	-0.116	0.030	-0.039
1	4	0	0.5	0.2	0.5	0.15	0.15	0.15	0.25	0.50	0.25	-0.130	0.050	-0.077
1	4	0	0.5	0.2	0.5	0.15	0.15	0.15	0.25	0.50	0.50	-0.138	0.058	-0.062
1	4	0	0.5	0.2	0.5	0.15	0.15	0.15	0.50	0.00	0.00	-0.128	0.056	-0.043
1	4	0	0.5	0.2	0.5	0.15	0.15	0.15	0.50	0.00	0.25	-0.135	0.039	-0.069
1	4	0	0.5	0.2	0.5	0.15	0.15	0.15	0.50	0.00	0.50	-0.126	0.048	-0.057
1	4	0	0.5	0.2	0.5	0.15	0.15	0.15	0.50	0.25	0.00	-0.119	0.024	-0.059
1	4	0	0.5	0.2	0.5	0.15	0.15	0.15	0.50	0.25	0.25	-0.127	0.042	-0.077
1	4	0	0.5	0.2	0.5	0.15	0.15	0.15	0.50	0.25	0.50	-0.115	0.094	-0.041
1	4	0	0.5	0.2	0.5	0.15	0.15	0.15	0.50	0.50	0.00	-0.107	0.063	-0.080
1	4	0	0.5	0.2	0.5	0.15	0.15	0.15	0.50	0.50	0.25	-0.132	0.055	-0.067
1	4	0	0.5	0.2	0.5	0.15	0.15	0.15	0.50	0.50	0.50	-0.119	0.055	-0.060
1	4	0	0.5	0.2	0.5	0.30	0.30	0.30	0.00	0.00	0.00	-0.075	0.004	-0.092
1	4	0	0.5	0.2	0.5	0.30	0.30	0.30	0.00	0.00	0.25	-0.093	0.019	-0.060
1	4	0	0.5	0.2	0.5	0.30	0.30	0.30	0.00	0.00	0.50	-0.080	-0.002	-0.065
1	4	0	0.5	0.2	0.5	0.30	0.30	0.30	0.00	0.25	0.00	-0.078	-0.007	-0.086
1	4	0	0.5	0.2	0.5	0.30	0.30	0.30	0.00	0.25	0.25	-0.084	0.017	-0.087
1	4	0	0.5	0.2	0.5	0.30	0.30	0.30	0.00	0.25	0.50	-0.087	0.010	-0.097
1	4	0	0.5	0.2	0.5	0.30	0.30	0.30	0.00	0.50	0.00	-0.070	-0.027	-0.128
1	4	0	0.5	0.2	0.5	0.30	0.30	0.30	0.00	0.50	0.25	-0.058	0.009	-0.118
1	4	0	0.5	0.2	0.5	0.30	0.30	0.30	0.00	0.50	0.50	-0.065	-0.014	-0.111
1	4	0	0.5	0.2	0.5	0.30	0.30	0.30	0.25	0.00	0.00	-0.053	0.026	-0.095
1	4	0	0.5	0.2	0.5	0.30	0.30	0.30	0.25	0.00	0.25	-0.063	0.021	-0.087
1	4	0	0.5	0.2	0.5	0.30	0.30	0.30	0.25	0.00	0.50	-0.073	0.025	-0.073
1	4	0	0.5	0.2	0.5	0.30	0.30	0.30	0.25	0.25	0.00	-0.070	0.033	-0.083
1	4	0	0.5	0.2	0.5	0.30	0.30	0.30	0.25	0.25	0.25	-0.063	0.011	-0.093
1	4	0	0.5	0.2	0.5	0.30	0.30	0.30	0.25	0.25	0.50	-0.049	0.031	-0.094
1	4	0	0.5	0.2	0.5	0.30	0.30	0.30	0.25	0.50	0.00	-0.068	-0.009	-0.117
1	4	0	0.5	0.2	0.5	0.30	0.30	0.30	0.25	0.50	0.25	-0.041	0.017	-0.111
1	4	0	0.5	0.2	0.5	0.30	0.30	0.30	0.25	0.50	0.50	-0.041	0.013	-0.128
1	4	0	0.5	0.2	0.5	0.30	0.30	0.30	0.50	0.00	0.00	-0.058	0.030	-0.068
1	4	0	0.5	0.2	0.5	0.30	0.30	0.30	0.50	0.00	0.25	-0.057	0.057	-0.067
1	4	0	0.5	0.2	0.5	0.30	0.30	0.30	0.50	0.00	0.50	-0.070	0.064	-0.061
1	4	0	0.5	0.2	0.5	0.30	0.30	0.30	0.50	0.25	0.00	-0.048	0.025	-0.081
1	4	0	0.5	0.2	0.5	0.30	0.30	0.30	0.50	0.25	0.25	-0.033	0.030	-0.079
1	4	0	0.5	0.2	0.5	0.30	0.30	0.30	0.50	0.25	0.50	-0.047	0.048	-0.070
1	4	0	0.5	0.2	0.5	0.30	0.30	0.30	0.50	0.50	0.00	-0.031	0.021	-0.092
1	4	0	0.5	0.2	0.5	0.30	0.30	0.30	0.50	0.50	0.25	-0.024	0.017	-0.105
1	4	0	0.5	0.2	0.5	0.30	0.30	0.30	0.50	0.50	0.50	-0.051	0.039	-0.123

(continued)

												Mean Bias		
N	m_1	$\frac{m_2}{m_1}$	$E(C)$	$E(R)$	$E(U)$	σ_C	σ_R	σ_U	ρ_{CR}	ρ_{CU}	ρ_{RU}	c	r	u
1	4	0	0.5	0.5	0.5	0.00	0.00	0.00	0.00	0.00	0.00	-0.077	0.063	-0.010
1	4	0	0.5	0.5	0.5	0.15	0.15	0.15	0.00	0.00	0.00	-0.053	0.029	-0.038
1	4	0	0.5	0.5	0.5	0.15	0.15	0.15	0.00	0.00	0.25	-0.034	0.013	-0.030
1	4	0	0.5	0.5	0.5	0.15	0.15	0.15	0.00	0.00	0.50	-0.071	0.016	-0.016
1	4	0	0.5	0.5	0.5	0.15	0.15	0.15	0.00	0.25	0.00	-0.044	0.002	-0.009
1	4	0	0.5	0.5	0.5	0.15	0.15	0.15	0.00	0.25	0.25	-0.044	-0.018	-0.018
1	4	0	0.5	0.5	0.5	0.15	0.15	0.15	0.00	0.25	0.50	-0.077	0.026	-0.018
1	4	0	0.5	0.5	0.5	0.15	0.15	0.15	0.00	0.50	0.00	-0.058	0.004	-0.025
1	4	0	0.5	0.5	0.5	0.15	0.15	0.15	0.00	0.50	0.25	-0.055	0.033	-0.032
1	4	0	0.5	0.5	0.5	0.15	0.15	0.15	0.00	0.50	0.50	-0.065	0.008	-0.026
1	4	0	0.5	0.5	0.5	0.15	0.15	0.15	0.25	0.00	0.00	-0.046	0.031	-0.029
1	4	0	0.5	0.5	0.5	0.15	0.15	0.15	0.25	0.00	0.25	-0.052	0.013	-0.027
1	4	0	0.5	0.5	0.5	0.15	0.15	0.15	0.25	0.00	0.50	-0.041	0.005	-0.028
1	4	0	0.5	0.5	0.5	0.15	0.15	0.15	0.25	0.25	0.00	-0.035	0.005	-0.008
1	4	0	0.5	0.5	0.5	0.15	0.15	0.15	0.25	0.25	0.25	-0.045	-0.024	0.001
1	4	0	0.5	0.5	0.5	0.15	0.15	0.15	0.25	0.25	0.50	-0.051	-0.004	-0.029
1	4	0	0.5	0.5	0.5	0.15	0.15	0.15	0.25	0.50	0.00	-0.036	0.011	-0.020
1	4	0	0.5	0.5	0.5	0.15	0.15	0.15	0.25	0.50	0.25	-0.044	0.007	-0.006
1	4	0	0.5	0.5	0.5	0.15	0.15	0.15	0.25	0.50	0.50	-0.048	0.028	-0.034
1	4	0	0.5	0.5	0.5	0.15	0.15	0.15	0.50	0.00	0.00	-0.037	-0.010	-0.020
1	4	0	0.5	0.5	0.5	0.15	0.15	0.15	0.50	0.00	0.25	-0.048	0.006	-0.032
1	4	0	0.5	0.5	0.5	0.15	0.15	0.15	0.50	0.00	0.50	-0.071	0.013	-0.028
1	4	0	0.5	0.5	0.5	0.15	0.15	0.15	0.50	0.25	0.00	-0.035	0.003	-0.028
1	4	0	0.5	0.5	0.5	0.15	0.15	0.15	0.50	0.25	0.25	-0.057	0.010	-0.021
1	4	0	0.5	0.5	0.5	0.15	0.15	0.15	0.50	0.25	0.50	-0.063	0.022	-0.021
1	4	0	0.5	0.5	0.5	0.15	0.15	0.15	0.50	0.50	0.00	-0.049	0.020	-0.054
1	4	0	0.5	0.5	0.5	0.15	0.15	0.15	0.50	0.50	0.25	-0.059	0.027	-0.036
1	4	0	0.5	0.5	0.5	0.15	0.15	0.15	0.50	0.50	0.50	-0.048	0.023	-0.018
1	4	0	0.5	0.5	0.5	0.30	0.30	0.30	0.00	0.00	0.00	0.004	-0.072	-0.038
1	4	0	0.5	0.5	0.5	0.30	0.30	0.30	0.00	0.00	0.25	-0.020	-0.068	-0.035
1	4	0	0.5	0.5	0.5	0.30	0.30	0.30	0.00	0.00	0.50	-0.029	-0.067	-0.026
1	4	0	0.5	0.5	0.5	0.30	0.30	0.30	0.00	0.25	0.00	0.024	-0.077	-0.039
1	4	0	0.5	0.5	0.5	0.30	0.30	0.30	0.00	0.25	0.25	-0.014	-0.057	-0.040
1	4	0	0.5	0.5	0.5	0.30	0.30	0.30	0.00	0.25	0.50	0.025	-0.042	-0.058
1	4	0	0.5	0.5	0.5	0.30	0.30	0.30	0.00	0.50	0.00	-0.009	-0.087	-0.070
1	4	0	0.5	0.5	0.5	0.30	0.30	0.30	0.00	0.50	0.25	0.018	-0.079	-0.073
1	4	0	0.5	0.5	0.5	0.30	0.30	0.30	0.00	0.50	0.50	0.004	-0.085	-0.063
1	4	0	0.5	0.5	0.5	0.30	0.30	0.30	0.25	0.00	0.00	-0.002	-0.074	-0.035
1	4	0	0.5	0.5	0.5	0.30	0.30	0.30	0.25	0.00	0.25	0.006	-0.056	-0.040

(continued)

N	m_1	$\frac{m_2}{m_1}$	$E(C)$	$E(R)$	$E(U)$	σ_C	σ_R	σ_U	ρ_{CR}	ρ_{CU}	ρ_{RU}	Mean Bias		
												c	r	u
1	4	0	0.5	0.5	0.5	0.30	0.30	0.30	0.25	0.00	0.50	-0.012	-0.008	-0.015
1	4	0	0.5	0.5	0.5	0.30	0.30	0.30	0.25	0.25	0.00	0.007	-0.064	-0.038
1	4	0	0.5	0.5	0.5	0.30	0.30	0.30	0.25	0.25	0.25	0.022	-0.034	-0.041
1	4	0	0.5	0.5	0.5	0.30	0.30	0.30	0.25	0.25	0.50	0.023	-0.013	-0.062
1	4	0	0.5	0.5	0.5	0.30	0.30	0.30	0.25	0.50	0.00	0.029	-0.078	-0.059
1	4	0	0.5	0.5	0.5	0.30	0.30	0.30	0.25	0.50	0.25	0.034	-0.062	-0.053
1	4	0	0.5	0.5	0.5	0.30	0.30	0.30	0.25	0.50	0.50	0.009	-0.027	-0.042
1	4	0	0.5	0.5	0.5	0.30	0.30	0.30	0.50	0.00	0.00	0.002	-0.054	-0.036
1	4	0	0.5	0.5	0.5	0.30	0.30	0.30	0.50	0.00	0.25	-0.017	-0.027	-0.019
1	4	0	0.5	0.5	0.5	0.30	0.30	0.30	0.50	0.00	0.50	-0.002	-0.023	-0.036
1	4	0	0.5	0.5	0.5	0.30	0.30	0.30	0.50	0.25	0.00	0.023	-0.068	-0.024
1	4	0	0.5	0.5	0.5	0.30	0.30	0.30	0.50	0.25	0.25	0.020	-0.061	-0.048
1	4	0	0.5	0.5	0.5	0.30	0.30	0.30	0.50	0.25	0.50	0.002	-0.035	-0.063
1	4	0	0.5	0.5	0.5	0.30	0.30	0.30	0.50	0.50	0.00	0.026	-0.060	-0.044
1	4	0	0.5	0.5	0.5	0.30	0.30	0.30	0.50	0.50	0.25	0.033	-0.043	-0.069
1	4	0	0.5	0.5	0.5	0.30	0.30	0.30	0.50	0.50	0.50	0.032	-0.040	-0.050
1	4	0	0.5	0.8	0.5	0.00	0.00	0.00	0.00	0.00	0.00	0.003	-0.075	0.024
1	4	0	0.5	0.8	0.5	0.15	0.15	0.15	0.00	0.00	0.00	0.012	-0.085	0.025
1	4	0	0.5	0.8	0.5	0.15	0.15	0.15	0.00	0.00	0.25	0.023	-0.086	0.020
1	4	0	0.5	0.8	0.5	0.15	0.15	0.15	0.00	0.00	0.50	0.017	-0.091	0.040
1	4	0	0.5	0.8	0.5	0.15	0.15	0.15	0.00	0.25	0.00	0.024	-0.089	0.009
1	4	0	0.5	0.8	0.5	0.15	0.15	0.15	0.00	0.25	0.25	0.017	-0.072	0.030
1	4	0	0.5	0.8	0.5	0.15	0.15	0.15	0.00	0.25	0.50	0.034	-0.063	0.018
1	4	0	0.5	0.8	0.5	0.15	0.15	0.15	0.00	0.50	0.00	0.022	-0.071	0.031
1	4	0	0.5	0.8	0.5	0.15	0.15	0.15	0.00	0.50	0.25	0.024	-0.093	0.010
1	4	0	0.5	0.8	0.5	0.15	0.15	0.15	0.00	0.50	0.50	-0.008	-0.075	0.000
1	4	0	0.5	0.8	0.5	0.15	0.15	0.15	0.25	0.00	0.00	0.026	-0.086	0.026
1	4	0	0.5	0.8	0.5	0.15	0.15	0.15	0.25	0.00	0.25	0.013	-0.070	0.026
1	4	0	0.5	0.8	0.5	0.15	0.15	0.15	0.25	0.00	0.50	0.016	-0.053	0.038
1	4	0	0.5	0.8	0.5	0.15	0.15	0.15	0.25	0.25	0.00	0.036	-0.089	0.032
1	4	0	0.5	0.8	0.5	0.15	0.15	0.15	0.25	0.25	0.25	0.041	-0.076	0.033
1	4	0	0.5	0.8	0.5	0.15	0.15	0.15	0.25	0.25	0.50	0.029	-0.068	0.032
1	4	0	0.5	0.8	0.5	0.15	0.15	0.15	0.25	0.50	0.00	0.013	-0.093	0.023
1	4	0	0.5	0.8	0.5	0.15	0.15	0.15	0.25	0.50	0.25	0.016	-0.082	0.017
1	4	0	0.5	0.8	0.5	0.15	0.15	0.15	0.25	0.50	0.50	0.009	-0.082	0.025
1	4	0	0.5	0.8	0.5	0.15	0.15	0.15	0.50	0.00	0.00	0.028	-0.080	0.040
1	4	0	0.5	0.8	0.5	0.15	0.15	0.15	0.50	0.00	0.25	0.043	-0.052	0.030
1	4	0	0.5	0.8	0.5	0.15	0.15	0.15	0.50	0.00	0.50	0.046	-0.073	0.033
1	4	0	0.5	0.8	0.5	0.15	0.15	0.15	0.50	0.25	0.00	0.024	-0.067	0.025

(continued)

N	m_1	$\frac{m_2}{m_1}$	$E(C)$	$E(R)$	$E(U)$	σ_C	σ_R	σ_U	ρ_{CR}	ρ_{CU}	ρ_{RU}	Mean Bias c	Mean Bias r	Mean Bias u
1	4	0	0.5	0.8	0.5	0.15	0.15	0.15	0.50	0.25	0.25	0.018	-0.086	0.033
1	4	0	0.5	0.8	0.5	0.15	0.15	0.15	0.50	0.25	0.50	0.013	-0.084	0.033
1	4	0	0.5	0.8	0.5	0.15	0.15	0.15	0.50	0.50	0.00	0.036	-0.072	0.026
1	4	0	0.5	0.8	0.5	0.15	0.15	0.15	0.50	0.50	0.25	0.008	-0.096	0.012
1	4	0	0.5	0.8	0.5	0.15	0.15	0.15	0.50	0.50	0.50	0.005	-0.085	0.026
1	4	0	0.5	0.8	0.5	0.30	0.30	0.30	0.00	0.00	0.00	0.059	-0.185	0.030
1	4	0	0.5	0.8	0.5	0.30	0.30	0.30	0.00	0.00	0.25	0.079	-0.143	0.003
1	4	0	0.5	0.8	0.5	0.30	0.30	0.30	0.00	0.00	0.50	0.045	-0.164	0.012
1	4	0	0.5	0.8	0.5	0.30	0.30	0.30	0.00	0.25	0.00	0.053	-0.177	0.005
1	4	0	0.5	0.8	0.5	0.30	0.30	0.30	0.00	0.25	0.25	0.036	-0.179	-0.004
1	4	0	0.5	0.8	0.5	0.30	0.30	0.30	0.00	0.25	0.50	0.041	-0.174	-0.007
1	4	0	0.5	0.8	0.5	0.30	0.30	0.30	0.00	0.50	0.00	0.059	-0.190	-0.018
1	4	0	0.5	0.8	0.5	0.30	0.30	0.30	0.00	0.50	0.25	0.052	-0.192	-0.026
1	4	0	0.5	0.8	0.5	0.30	0.30	0.30	0.00	0.50	0.50	0.063	-0.179	-0.025
1	4	0	0.5	0.8	0.5	0.30	0.30	0.30	0.25	0.00	0.00	0.039	-0.176	0.029
1	4	0	0.5	0.8	0.5	0.30	0.30	0.30	0.25	0.00	0.25	0.048	-0.150	0.050
1	4	0	0.5	0.8	0.5	0.30	0.30	0.30	0.25	0.00	0.50	0.038	-0.158	0.026
1	4	0	0.5	0.8	0.5	0.30	0.30	0.30	0.25	0.25	0.00	0.068	-0.165	0.003
1	4	0	0.5	0.8	0.5	0.30	0.30	0.30	0.25	0.25	0.25	0.071	-0.161	0.004
1	4	0	0.5	0.8	0.5	0.30	0.30	0.30	0.25	0.25	0.50	0.056	-0.170	-0.001
1	4	0	0.5	0.8	0.5	0.30	0.30	0.30	0.25	0.50	0.00	0.074	-0.167	0.000
1	4	0	0.5	0.8	0.5	0.30	0.30	0.30	0.25	0.50	0.25	0.071	-0.168	-0.018
1	4	0	0.5	0.8	0.5	0.30	0.30	0.30	0.25	0.50	0.50	0.082	-0.158	-0.003
1	4	0	0.5	0.8	0.5	0.30	0.30	0.30	0.50	0.00	0.00	0.057	-0.133	0.035
1	4	0	0.5	0.8	0.5	0.30	0.30	0.30	0.50	0.00	0.25	0.048	-0.154	0.030
1	4	0	0.5	0.8	0.5	0.30	0.30	0.30	0.50	0.00	0.50	0.057	-0.141	0.015
1	4	0	0.5	0.8	0.5	0.30	0.30	0.30	0.50	0.25	0.00	0.090	-0.149	0.030
1	4	0	0.5	0.8	0.5	0.30	0.30	0.30	0.50	0.25	0.25	0.066	-0.157	0.025
1	4	0	0.5	0.8	0.5	0.30	0.30	0.30	0.50	0.25	0.50	0.062	-0.131	0.006
1	4	0	0.5	0.8	0.5	0.30	0.30	0.30	0.50	0.50	0.00	0.078	-0.165	0.003
1	4	0	0.5	0.8	0.5	0.30	0.30	0.30	0.50	0.50	0.25	0.064	-0.159	-0.019
1	4	0	0.5	0.8	0.5	0.30	0.30	0.30	0.50	0.50	0.50	0.045	-0.157	-0.001
1	4	0	0.8	0.2	0.5	0.00	0.00	0.00	0.00	0.00	0.00	-0.247	0.112	-0.212
1	4	0	0.8	0.2	0.5	0.15	0.15	0.15	0.00	0.00	0.00	-0.213	0.080	-0.215
1	4	0	0.8	0.2	0.5	0.15	0.15	0.15	0.00	0.00	0.25	-0.198	0.074	-0.202
1	4	0	0.8	0.2	0.5	0.15	0.15	0.15	0.00	0.00	0.50	-0.219	0.066	-0.213
1	4	0	0.8	0.2	0.5	0.15	0.15	0.15	0.00	0.25	0.00	-0.221	0.064	-0.233
1	4	0	0.8	0.2	0.5	0.15	0.15	0.15	0.00	0.25	0.25	-0.211	0.068	-0.208
1	4	0	0.8	0.2	0.5	0.15	0.15	0.15	0.00	0.25	0.50	-0.224	0.090	-0.211

(continued)

N	m_1	$\frac{m_2}{m_1}$	$E(C)$	$E(R)$	$E(U)$	σ_C	σ_R	σ_U	ρ_{CR}	ρ_{CU}	ρ_{RU}	Mean Bias c	Mean Bias r	Mean Bias u
1	4	0	0.8	0.2	0.5	0.15	0.15	0.15	0.00	0.50	0.00	-0.218	0.077	-0.218
1	4	0	0.8	0.2	0.5	0.15	0.15	0.15	0.00	0.50	0.25	-0.236	0.070	-0.225
1	4	0	0.8	0.2	0.5	0.15	0.15	0.15	0.00	0.50	0.50	-0.221	0.094	-0.231
1	4	0	0.8	0.2	0.5	0.15	0.15	0.15	0.25	0.00	0.00	-0.210	0.073	-0.200
1	4	0	0.8	0.2	0.5	0.15	0.15	0.15	0.25	0.00	0.25	-0.213	0.084	-0.203
1	4	0	0.8	0.2	0.5	0.15	0.15	0.15	0.25	0.00	0.50	-0.205	0.089	-0.201
1	4	0	0.8	0.2	0.5	0.15	0.15	0.15	0.25	0.25	0.00	-0.203	0.099	-0.209
1	4	0	0.8	0.2	0.5	0.15	0.15	0.15	0.25	0.25	0.25	-0.223	0.049	-0.212
1	4	0	0.8	0.2	0.5	0.15	0.15	0.15	0.25	0.25	0.50	-0.220	0.073	-0.242
1	4	0	0.8	0.2	0.5	0.15	0.15	0.15	0.25	0.50	0.00	-0.223	0.090	-0.233
1	4	0	0.8	0.2	0.5	0.15	0.15	0.15	0.25	0.50	0.25	-0.220	0.088	-0.239
1	4	0	0.8	0.2	0.5	0.15	0.15	0.15	0.25	0.50	0.50	-0.221	0.072	-0.215
1	4	0	0.8	0.2	0.5	0.15	0.15	0.15	0.50	0.00	0.00	-0.198	0.080	-0.208
1	4	0	0.8	0.2	0.5	0.15	0.15	0.15	0.50	0.00	0.25	-0.200	0.067	-0.171
1	4	0	0.8	0.2	0.5	0.15	0.15	0.15	0.50	0.00	0.50	-0.220	0.066	-0.216
1	4	0	0.8	0.2	0.5	0.15	0.15	0.15	0.50	0.25	0.00	-0.218	0.080	-0.201
1	4	0	0.8	0.2	0.5	0.15	0.15	0.15	0.50	0.25	0.25	-0.221	0.083	-0.220
1	4	0	0.8	0.2	0.5	0.15	0.15	0.15	0.50	0.25	0.50	-0.208	0.064	-0.209
1	4	0	0.8	0.2	0.5	0.15	0.15	0.15	0.50	0.50	0.00	-0.195	0.099	-0.215
1	4	0	0.8	0.2	0.5	0.15	0.15	0.15	0.50	0.50	0.25	-0.214	0.088	-0.220
1	4	0	0.8	0.2	0.5	0.15	0.15	0.15	0.50	0.50	0.50	-0.201	0.074	-0.220
1	4	0	0.8	0.2	0.5	0.30	0.30	0.30	0.00	0.00	0.00	-0.187	0.027	-0.241
1	4	0	0.8	0.2	0.5	0.30	0.30	0.30	0.00	0.00	0.25	-0.193	0.057	-0.230
1	4	0	0.8	0.2	0.5	0.30	0.30	0.30	0.00	0.00	0.50	-0.200	0.050	-0.231
1	4	0	0.8	0.2	0.5	0.30	0.30	0.30	0.00	0.25	0.00	-0.148	0.038	-0.270
1	4	0	0.8	0.2	0.5	0.30	0.30	0.30	0.00	0.25	0.25	-0.183	0.044	-0.251
1	4	0	0.8	0.2	0.5	0.30	0.30	0.30	0.00	0.25	0.50	-0.178	0.046	-0.263
1	4	0	0.8	0.2	0.5	0.30	0.30	0.30	0.00	0.50	0.00	-0.163	0.030	-0.275
1	4	0	0.8	0.2	0.5	0.30	0.30	0.30	0.00	0.50	0.25	-0.185	0.016	-0.289
1	4	0	0.8	0.2	0.5	0.30	0.30	0.30	0.00	0.50	0.50	-0.183	0.036	-0.294
1	4	0	0.8	0.2	0.5	0.30	0.30	0.30	0.25	0.00	0.00	-0.192	0.029	-0.231
1	4	0	0.8	0.2	0.5	0.30	0.30	0.30	0.25	0.00	0.25	-0.205	0.043	-0.239
1	4	0	0.8	0.2	0.5	0.30	0.30	0.30	0.25	0.00	0.50	-0.206	0.042	-0.218
1	4	0	0.8	0.2	0.5	0.30	0.30	0.30	0.25	0.25	0.00	-0.175	0.031	-0.252
1	4	0	0.8	0.2	0.5	0.30	0.30	0.30	0.25	0.25	0.25	-0.181	0.059	-0.269
1	4	0	0.8	0.2	0.5	0.30	0.30	0.30	0.25	0.25	0.50	-0.188	0.066	-0.272
1	4	0	0.8	0.2	0.5	0.30	0.30	0.30	0.25	0.50	0.00	-0.187	0.026	-0.282
1	4	0	0.8	0.2	0.5	0.30	0.30	0.30	0.25	0.50	0.25	-0.155	0.071	-0.275
1	4	0	0.8	0.2	0.5	0.30	0.30	0.30	0.25	0.50	0.50	-0.195	0.043	-0.285

(continued)

												Mean Bias		
N	m_1	$\frac{m_2}{m_1}$	$E(C)$	$E(R)$	$E(U)$	σ_C	σ_R	σ_U	ρ_{CR}	ρ_{CU}	ρ_{RU}	c	r	u
1	4	0	0.8	0.2	0.5	0.30	0.30	0.30	0.50	0.00	0.00	-0.170	0.051	-0.240
1	4	0	0.8	0.2	0.5	0.30	0.30	0.30	0.50	0.00	0.25	-0.196	0.032	-0.238
1	4	0	0.8	0.2	0.5	0.30	0.30	0.30	0.50	0.00	0.50	-0.173	0.064	-0.217
1	4	0	0.8	0.2	0.5	0.30	0.30	0.30	0.50	0.25	0.00	-0.178	0.054	-0.247
1	4	0	0.8	0.2	0.5	0.30	0.30	0.30	0.50	0.25	0.25	-0.170	0.043	-0.258
1	4	0	0.8	0.2	0.5	0.30	0.30	0.30	0.50	0.25	0.50	-0.159	0.057	-0.249
1	4	0	0.8	0.2	0.5	0.30	0.30	0.30	0.50	0.50	0.00	-0.162	0.038	-0.273
1	4	0	0.8	0.2	0.5	0.30	0.30	0.30	0.50	0.50	0.25	-0.187	0.055	-0.274
1	4	0	0.8	0.2	0.5	0.30	0.30	0.30	0.50	0.50	0.50	-0.162	0.051	-0.266
1	4	0	0.8	0.5	0.5	0.00	0.00	0.00	0.00	0.00	0.00	-0.100	0.108	-0.150
1	4	0	0.8	0.5	0.5	0.15	0.15	0.15	0.00	0.00	0.00	-0.102	0.084	-0.150
1	4	0	0.8	0.5	0.5	0.15	0.15	0.15	0.00	0.00	0.25	-0.093	0.072	-0.140
1	4	0	0.8	0.5	0.5	0.15	0.15	0.15	0.00	0.00	0.50	-0.106	0.068	-0.141
1	4	0	0.8	0.5	0.5	0.15	0.15	0.15	0.00	0.25	0.00	-0.091	0.059	-0.121
1	4	0	0.8	0.5	0.5	0.15	0.15	0.15	0.00	0.25	0.25	-0.088	0.064	-0.164
1	4	0	0.8	0.5	0.5	0.15	0.15	0.15	0.00	0.25	0.50	-0.098	0.062	-0.128
1	4	0	0.8	0.5	0.5	0.15	0.15	0.15	0.00	0.50	0.00	-0.114	0.080	-0.156
1	4	0	0.8	0.5	0.5	0.15	0.15	0.15	0.00	0.50	0.25	-0.099	0.066	-0.152
1	4	0	0.8	0.5	0.5	0.15	0.15	0.15	0.00	0.50	0.50	-0.113	0.062	-0.157
1	4	0	0.8	0.5	0.5	0.15	0.15	0.15	0.25	0.00	0.00	-0.092	0.058	-0.130
1	4	0	0.8	0.5	0.5	0.15	0.15	0.15	0.25	0.00	0.25	-0.113	0.071	-0.148
1	4	0	0.8	0.5	0.5	0.15	0.15	0.15	0.25	0.00	0.50	-0.094	0.075	-0.140
1	4	0	0.8	0.5	0.5	0.15	0.15	0.15	0.25	0.25	0.00	-0.115	0.065	-0.155
1	4	0	0.8	0.5	0.5	0.15	0.15	0.15	0.25	0.25	0.25	-0.093	0.085	-0.164
1	4	0	0.8	0.5	0.5	0.15	0.15	0.15	0.25	0.25	0.50	-0.102	0.072	-0.136
1	4	0	0.8	0.5	0.5	0.15	0.15	0.15	0.25	0.50	0.00	-0.092	0.082	-0.159
1	4	0	0.8	0.5	0.5	0.15	0.15	0.15	0.25	0.50	0.25	-0.099	0.070	-0.142
1	4	0	0.8	0.5	0.5	0.15	0.15	0.15	0.25	0.50	0.50	-0.099	0.059	-0.165
1	4	0	0.8	0.5	0.5	0.15	0.15	0.15	0.50	0.00	0.00	-0.098	0.099	-0.145
1	4	0	0.8	0.5	0.5	0.15	0.15	0.15	0.50	0.00	0.25	-0.109	0.090	-0.127
1	4	0	0.8	0.5	0.5	0.15	0.15	0.15	0.50	0.00	0.50	-0.099	0.070	-0.130
1	4	0	0.8	0.5	0.5	0.15	0.15	0.15	0.50	0.25	0.00	-0.096	0.077	-0.137
1	4	0	0.8	0.5	0.5	0.15	0.15	0.15	0.50	0.25	0.25	-0.083	0.078	-0.122
1	4	0	0.8	0.5	0.5	0.15	0.15	0.15	0.50	0.25	0.50	-0.099	0.089	-0.135
1	4	0	0.8	0.5	0.5	0.15	0.15	0.15	0.50	0.50	0.00	-0.093	0.090	-0.152
1	4	0	0.8	0.5	0.5	0.15	0.15	0.15	0.50	0.50	0.25	-0.117	0.083	-0.159
1	4	0	0.8	0.5	0.5	0.15	0.15	0.15	0.50	0.50	0.50	-0.098	0.092	-0.147
1	4	0	0.8	0.5	0.5	0.30	0.30	0.30	0.00	0.00	0.00	-0.086	-0.014	-0.149
1	4	0	0.8	0.5	0.5	0.30	0.30	0.30	0.00	0.00	0.25	-0.085	0.003	-0.163

(continued)

												Mean Bias		
N	m_1	$\frac{m_2}{m_1}$	$E(C)$	$E(R)$	$E(U)$	σ_C	σ_R	σ_U	ρ_{CR}	ρ_{CU}	ρ_{RU}	c	r	u
1	4	0	0.8	0.5	0.5	0.30	0.30	0.30	0.00	0.00	0.50	-0.057	0.003	-0.154
1	4	0	0.8	0.5	0.5	0.30	0.30	0.30	0.00	0.25	0.00	-0.065	-0.007	-0.168
1	4	0	0.8	0.5	0.5	0.30	0.30	0.30	0.00	0.25	0.25	-0.091	-0.005	-0.169
1	4	0	0.8	0.5	0.5	0.30	0.30	0.30	0.00	0.25	0.50	-0.075	-0.007	-0.186
1	4	0	0.8	0.5	0.5	0.30	0.30	0.30	0.00	0.50	0.00	-0.070	-0.002	-0.204
1	4	0	0.8	0.5	0.5	0.30	0.30	0.30	0.00	0.50	0.25	-0.040	0.027	-0.206
1	4	0	0.8	0.5	0.5	0.30	0.30	0.30	0.00	0.50	0.50	-0.065	-0.001	-0.215
1	4	0	0.8	0.5	0.5	0.30	0.30	0.30	0.25	0.00	0.00	-0.064	-0.016	-0.161
1	4	0	0.8	0.5	0.5	0.30	0.30	0.30	0.25	0.00	0.25	-0.065	0.012	-0.143
1	4	0	0.8	0.5	0.5	0.30	0.30	0.30	0.25	0.00	0.50	-0.092	0.022	-0.149
1	4	0	0.8	0.5	0.5	0.30	0.30	0.30	0.25	0.25	0.00	-0.063	-0.002	-0.174
1	4	0	0.8	0.5	0.5	0.30	0.30	0.30	0.25	0.25	0.25	-0.070	-0.015	-0.168
1	4	0	0.8	0.5	0.5	0.30	0.30	0.30	0.25	0.25	0.50	-0.059	0.023	-0.169
1	4	0	0.8	0.5	0.5	0.30	0.30	0.30	0.25	0.50	0.00	-0.067	-0.004	-0.201
1	4	0	0.8	0.5	0.5	0.30	0.30	0.30	0.25	0.50	0.25	-0.046	0.012	-0.186
1	4	0	0.8	0.5	0.5	0.30	0.30	0.30	0.25	0.50	0.50	-0.070	0.019	-0.196
1	4	0	0.8	0.5	0.5	0.30	0.30	0.30	0.50	0.00	0.00	-0.068	0.027	-0.138
1	4	0	0.8	0.5	0.5	0.30	0.30	0.30	0.50	0.00	0.25	-0.051	0.045	-0.143
1	4	0	0.8	0.5	0.5	0.30	0.30	0.30	0.50	0.00	0.50	-0.062	0.024	-0.158
1	4	0	0.8	0.5	0.5	0.30	0.30	0.30	0.50	0.25	0.00	-0.041	0.021	-0.172
1	4	0	0.8	0.5	0.5	0.30	0.30	0.30	0.50	0.25	0.25	-0.074	0.018	-0.167
1	4	0	0.8	0.5	0.5	0.30	0.30	0.30	0.50	0.25	0.50	-0.072	0.016	-0.176
1	4	0	0.8	0.5	0.5	0.30	0.30	0.30	0.50	0.50	0.00	-0.059	0.002	-0.200
1	4	0	0.8	0.5	0.5	0.30	0.30	0.30	0.50	0.50	0.25	-0.047	0.030	-0.201
1	4	0	0.8	0.5	0.5	0.30	0.30	0.30	0.50	0.50	0.50	-0.045	0.020	-0.192
1	4	0	0.8	0.8	0.5	0.00	0.00	0.00	0.00	0.00	0.00	-0.008	0.027	-0.045
1	4	0	0.8	0.8	0.5	0.15	0.15	0.15	0.00	0.00	0.00	-0.011	0.025	-0.034
1	4	0	0.8	0.8	0.5	0.15	0.15	0.15	0.00	0.00	0.25	-0.004	0.010	-0.053
1	4	0	0.8	0.8	0.5	0.15	0.15	0.15	0.00	0.00	0.50	-0.022	-0.001	-0.046
1	4	0	0.8	0.8	0.5	0.15	0.15	0.15	0.00	0.25	0.00	-0.011	0.009	-0.054
1	4	0	0.8	0.8	0.5	0.15	0.15	0.15	0.00	0.25	0.25	-0.005	0.004	-0.062
1	4	0	0.8	0.8	0.5	0.15	0.15	0.15	0.00	0.25	0.50	-0.010	0.025	-0.057
1	4	0	0.8	0.8	0.5	0.15	0.15	0.15	0.00	0.50	0.00	-0.009	0.012	-0.068
1	4	0	0.8	0.8	0.5	0.15	0.15	0.15	0.00	0.50	0.25	-0.009	0.007	-0.071
1	4	0	0.8	0.8	0.5	0.15	0.15	0.15	0.00	0.50	0.50	-0.006	0.005	-0.076
1	4	0	0.8	0.8	0.5	0.15	0.15	0.15	0.25	0.00	0.00	-0.020	0.008	-0.053
1	4	0	0.8	0.8	0.5	0.15	0.15	0.15	0.25	0.00	0.25	-0.036	0.007	-0.061
1	4	0	0.8	0.8	0.5	0.15	0.15	0.15	0.25	0.00	0.50	-0.013	0.023	-0.041
1	4	0	0.8	0.8	0.5	0.15	0.15	0.15	0.25	0.25	0.00	-0.028	0.001	-0.051

(continued)

												Mean Bias		
N	m_1	$\frac{m_2}{m_1}$	$E(C)$	$E(R)$	$E(U)$	σ_C	σ_R	σ_U	ρ_{CR}	ρ_{CU}	ρ_{RU}	c	r	u
1	4	0	0.8	0.8	0.5	0.15	0.15	0.15	0.25	0.25	0.25	-0.022	0.007	-0.053
1	4	0	0.8	0.8	0.5	0.15	0.15	0.15	0.25	0.25	0.50	0.000	0.019	-0.075
1	4	0	0.8	0.8	0.5	0.15	0.15	0.15	0.25	0.50	0.00	-0.010	0.015	-0.065
1	4	0	0.8	0.8	0.5	0.15	0.15	0.15	0.25	0.50	0.25	-0.014	0.014	-0.069
1	4	0	0.8	0.8	0.5	0.15	0.15	0.15	0.25	0.50	0.50	-0.014	0.015	-0.053
1	4	0	0.8	0.8	0.5	0.15	0.15	0.15	0.50	0.00	0.00	-0.023	0.012	-0.045
1	4	0	0.8	0.8	0.5	0.15	0.15	0.15	0.50	0.00	0.25	0.000	0.005	-0.059
1	4	0	0.8	0.8	0.5	0.15	0.15	0.15	0.50	0.00	0.50	-0.015	0.023	-0.024
1	4	0	0.8	0.8	0.5	0.15	0.15	0.15	0.50	0.25	0.00	-0.027	0.011	-0.052
1	4	0	0.8	0.8	0.5	0.15	0.15	0.15	0.50	0.25	0.25	-0.011	0.020	-0.058
1	4	0	0.8	0.8	0.5	0.15	0.15	0.15	0.50	0.25	0.50	-0.012	0.019	-0.067
1	4	0	0.8	0.8	0.5	0.15	0.15	0.15	0.50	0.50	0.00	-0.027	0.001	-0.039
1	4	0	0.8	0.8	0.5	0.15	0.15	0.15	0.50	0.50	0.25	-0.026	0.013	-0.057
1	4	0	0.8	0.8	0.5	0.15	0.15	0.15	0.50	0.50	0.50	-0.011	0.006	-0.073
1	4	0	0.8	0.8	0.5	0.30	0.30	0.30	0.00	0.00	0.00	-0.009	-0.060	-0.067
1	4	0	0.8	0.8	0.5	0.30	0.30	0.30	0.00	0.00	0.25	-0.005	-0.054	-0.048
1	4	0	0.8	0.8	0.5	0.30	0.30	0.30	0.00	0.00	0.50	-0.034	-0.056	-0.060
1	4	0	0.8	0.8	0.5	0.30	0.30	0.30	0.00	0.25	0.00	-0.003	-0.058	-0.077
1	4	0	0.8	0.8	0.5	0.30	0.30	0.30	0.00	0.25	0.25	0.011	-0.045	-0.091
1	4	0	0.8	0.8	0.5	0.30	0.30	0.30	0.00	0.25	0.50	-0.017	-0.094	-0.093
1	4	0	0.8	0.8	0.5	0.30	0.30	0.30	0.00	0.50	0.00	0.017	-0.068	-0.120
1	4	0	0.8	0.8	0.5	0.30	0.30	0.30	0.00	0.50	0.25	0.013	-0.080	-0.111
1	4	0	0.8	0.8	0.5	0.30	0.30	0.30	0.00	0.50	0.50	0.010	-0.082	-0.105
1	4	0	0.8	0.8	0.5	0.30	0.30	0.30	0.25	0.00	0.00	0.017	-0.054	-0.060
1	4	0	0.8	0.8	0.5	0.30	0.30	0.30	0.25	0.00	0.25	-0.008	-0.042	-0.060
1	4	0	0.8	0.8	0.5	0.30	0.30	0.30	0.25	0.00	0.50	-0.027	-0.052	-0.056
1	4	0	0.8	0.8	0.5	0.30	0.30	0.30	0.25	0.25	0.00	-0.014	-0.061	-0.074
1	4	0	0.8	0.8	0.5	0.30	0.30	0.30	0.25	0.25	0.25	0.013	-0.046	-0.096
1	4	0	0.8	0.8	0.5	0.30	0.30	0.30	0.25	0.25	0.50	0.019	-0.020	-0.084
1	4	0	0.8	0.8	0.5	0.30	0.30	0.30	0.25	0.50	0.00	0.011	-0.060	-0.086
1	4	0	0.8	0.8	0.5	0.30	0.30	0.30	0.25	0.50	0.25	-0.005	-0.087	-0.109
1	4	0	0.8	0.8	0.5	0.30	0.30	0.30	0.25	0.50	0.50	0.005	-0.071	-0.096
1	4	0	0.8	0.8	0.5	0.30	0.30	0.30	0.50	0.00	0.00	0.007	-0.039	-0.045
1	4	0	0.8	0.8	0.5	0.30	0.30	0.30	0.50	0.00	0.25	0.004	-0.037	-0.045
1	4	0	0.8	0.8	0.5	0.30	0.30	0.30	0.50	0.00	0.50	-0.025	-0.052	-0.044
1	4	0	0.8	0.8	0.5	0.30	0.30	0.30	0.50	0.25	0.00	0.010	-0.031	-0.070
1	4	0	0.8	0.8	0.5	0.30	0.30	0.30	0.50	0.25	0.25	0.013	-0.052	-0.076
1	4	0	0.8	0.8	0.5	0.30	0.30	0.30	0.50	0.25	0.50	0.000	-0.034	-0.073
1	4	0	0.8	0.8	0.5	0.30	0.30	0.30	0.50	0.50	0.00	0.010	-0.037	-0.077

(continued)

N	m_1	$\frac{m_2}{m_1}$	$E(C)$	$E(R)$	$E(U)$	σ_C	σ_R	σ_U	ρ_{CR}	ρ_{CU}	ρ_{RU}	Mean Bias c	r	u
1	4	0	0.8	0.8	0.5	0.30	0.30	0.30	0.50	0.50	0.25	0.024	-0.038	-0.099
1	4	0	0.8	0.8	0.5	0.30	0.30	0.30	0.50	0.50	0.50	0.025	-0.031	-0.097
1	4	1	0.2	0.2	0.5	0.00	0.00	0.00	0.00	0.00	0.00	0.023	-0.050	0.033
1	4	1	0.2	0.2	0.5	0.15	0.15	0.15	0.00	0.00	0.00	0.040	-0.064	0.024
1	4	1	0.2	0.2	0.5	0.15	0.15	0.15	0.00	0.00	0.25	0.051	-0.061	0.029
1	4	1	0.2	0.2	0.5	0.15	0.15	0.15	0.00	0.00	0.50	0.045	-0.077	0.025
1	4	1	0.2	0.2	0.5	0.15	0.15	0.15	0.00	0.25	0.00	0.056	-0.073	0.036
1	4	1	0.2	0.2	0.5	0.15	0.15	0.15	0.00	0.25	0.25	0.055	-0.058	0.026
1	4	1	0.2	0.2	0.5	0.15	0.15	0.15	0.00	0.25	0.50	0.042	-0.058	0.017
1	4	1	0.2	0.2	0.5	0.15	0.15	0.15	0.00	0.50	0.00	0.072	-0.088	0.030
1	4	1	0.2	0.2	0.5	0.15	0.15	0.15	0.00	0.50	0.25	0.078	-0.068	0.035
1	4	1	0.2	0.2	0.5	0.15	0.15	0.15	0.00	0.50	0.50	0.060	-0.079	0.016
1	4	1	0.2	0.2	0.5	0.15	0.15	0.15	0.25	0.00	0.00	0.057	-0.062	0.029
1	4	1	0.2	0.2	0.5	0.15	0.15	0.15	0.25	0.00	0.25	0.053	-0.072	0.023
1	4	1	0.2	0.2	0.5	0.15	0.15	0.15	0.25	0.00	0.50	0.062	-0.042	0.040
1	4	1	0.2	0.2	0.5	0.15	0.15	0.15	0.25	0.25	0.00	0.074	-0.040	0.030
1	4	1	0.2	0.2	0.5	0.15	0.15	0.15	0.25	0.25	0.25	0.055	-0.040	0.029
1	4	1	0.2	0.2	0.5	0.15	0.15	0.15	0.25	0.25	0.50	0.069	-0.065	0.019
1	4	1	0.2	0.2	0.5	0.15	0.15	0.15	0.25	0.50	0.00	0.057	-0.072	0.031
1	4	1	0.2	0.2	0.5	0.15	0.15	0.15	0.25	0.50	0.25	0.085	-0.062	0.042
1	4	1	0.2	0.2	0.5	0.15	0.15	0.15	0.25	0.50	0.50	0.073	-0.058	0.045
1	4	1	0.2	0.2	0.5	0.15	0.15	0.15	0.50	0.00	0.00	0.050	-0.050	0.027
1	4	1	0.2	0.2	0.5	0.15	0.15	0.15	0.50	0.00	0.25	0.061	-0.048	0.040
1	4	1	0.2	0.2	0.5	0.15	0.15	0.15	0.50	0.00	0.50	0.050	-0.051	0.021
1	4	1	0.2	0.2	0.5	0.15	0.15	0.15	0.50	0.25	0.00	0.061	-0.048	0.021
1	4	1	0.2	0.2	0.5	0.15	0.15	0.15	0.50	0.25	0.25	0.050	-0.059	0.009
1	4	1	0.2	0.2	0.5	0.15	0.15	0.15	0.50	0.25	0.50	0.061	-0.047	0.028
1	4	1	0.2	0.2	0.5	0.15	0.15	0.15	0.50	0.50	0.00	0.053	-0.065	0.038
1	4	1	0.2	0.2	0.5	0.15	0.15	0.15	0.50	0.50	0.25	0.067	-0.045	0.034
1	4	1	0.2	0.2	0.5	0.15	0.15	0.15	0.50	0.50	0.50	0.070	-0.038	0.026
1	4	1	0.2	0.2	0.5	0.30	0.30	0.30	0.00	0.00	0.00	0.066	-0.027	0.040
1	4	1	0.2	0.2	0.5	0.30	0.30	0.30	0.00	0.00	0.25	0.095	-0.039	0.005
1	4	1	0.2	0.2	0.5	0.30	0.30	0.30	0.00	0.00	0.50	0.085	-0.033	0.016
1	4	1	0.2	0.2	0.5	0.30	0.30	0.30	0.00	0.25	0.00	0.101	-0.043	0.049
1	4	1	0.2	0.2	0.5	0.30	0.30	0.30	0.00	0.25	0.25	0.107	-0.060	0.043
1	4	1	0.2	0.2	0.5	0.30	0.30	0.30	0.00	0.25	0.50	0.100	-0.038	0.004
1	4	1	0.2	0.2	0.5	0.30	0.30	0.30	0.00	0.50	0.00	0.121	-0.065	0.025
1	4	1	0.2	0.2	0.5	0.30	0.30	0.30	0.00	0.50	0.25	0.107	-0.057	0.007
1	4	1	0.2	0.2	0.5	0.30	0.30	0.30	0.00	0.50	0.50	0.128	-0.063	0.016

(continued)

												Mean Bias		
N	m_1	$\frac{m_2}{m_1}$	$E(C)$	$E(R)$	$E(U)$	σ_C	σ_R	σ_U	ρ_{CR}	ρ_{CU}	ρ_{RU}	c	r	u
1	4	1	0.2	0.2	0.5	0.30	0.30	0.30	0.25	0.00	0.00	0.104	-0.022	0.026
1	4	1	0.2	0.2	0.5	0.30	0.30	0.30	0.25	0.00	0.25	0.099	-0.006	0.007
1	4	1	0.2	0.2	0.5	0.30	0.30	0.30	0.25	0.00	0.50	0.083	-0.005	0.023
1	4	1	0.2	0.2	0.5	0.30	0.30	0.30	0.25	0.25	0.00	0.094	-0.023	0.022
1	4	1	0.2	0.2	0.5	0.30	0.30	0.30	0.25	0.25	0.25	0.122	-0.031	0.029
1	4	1	0.2	0.2	0.5	0.30	0.30	0.30	0.25	0.25	0.50	0.128	-0.032	0.022
1	4	1	0.2	0.2	0.5	0.30	0.30	0.30	0.25	0.50	0.00	0.098	-0.033	0.034
1	4	1	0.2	0.2	0.5	0.30	0.30	0.30	0.25	0.50	0.25	0.090	-0.028	0.029
1	4	1	0.2	0.2	0.5	0.30	0.30	0.30	0.25	0.50	0.50	0.109	-0.042	0.014
1	4	1	0.2	0.2	0.5	0.30	0.30	0.30	0.50	0.00	0.00	0.095	0.005	0.006
1	4	1	0.2	0.2	0.5	0.30	0.30	0.30	0.50	0.00	0.25	0.093	0.018	0.029
1	4	1	0.2	0.2	0.5	0.30	0.30	0.30	0.50	0.00	0.50	0.093	0.016	0.014
1	4	1	0.2	0.2	0.5	0.30	0.30	0.30	0.50	0.25	0.00	0.118	-0.008	0.021
1	4	1	0.2	0.2	0.5	0.30	0.30	0.30	0.50	0.25	0.25	0.108	-0.008	0.040
1	4	1	0.2	0.2	0.5	0.30	0.30	0.30	0.50	0.25	0.50	0.103	0.002	0.029
1	4	1	0.2	0.2	0.5	0.30	0.30	0.30	0.50	0.50	0.00	0.103	-0.037	0.025
1	4	1	0.2	0.2	0.5	0.30	0.30	0.30	0.50	0.50	0.25	0.111	0.009	0.027
1	4	1	0.2	0.2	0.5	0.30	0.30	0.30	0.50	0.50	0.50	0.131	0.011	0.026
1	4	1	0.2	0.5	0.5	0.00	0.00	0.00	0.00	0.00	0.00	0.071	-0.199	0.039
1	4	1	0.2	0.5	0.5	0.15	0.15	0.15	0.00	0.00	0.00	0.084	-0.217	0.029
1	4	1	0.2	0.5	0.5	0.15	0.15	0.15	0.00	0.00	0.25	0.082	-0.237	0.034
1	4	1	0.2	0.5	0.5	0.15	0.15	0.15	0.00	0.00	0.50	0.069	-0.219	0.025
1	4	1	0.2	0.5	0.5	0.15	0.15	0.15	0.00	0.25	0.00	0.076	-0.213	0.035
1	4	1	0.2	0.5	0.5	0.15	0.15	0.15	0.00	0.25	0.25	0.073	-0.241	0.010
1	4	1	0.2	0.5	0.5	0.15	0.15	0.15	0.00	0.25	0.50	0.075	-0.219	0.051
1	4	1	0.2	0.5	0.5	0.15	0.15	0.15	0.00	0.50	0.00	0.057	-0.247	0.032
1	4	1	0.2	0.5	0.5	0.15	0.15	0.15	0.00	0.50	0.25	0.079	-0.239	0.042
1	4	1	0.2	0.5	0.5	0.15	0.15	0.15	0.00	0.50	0.50	0.075	-0.225	0.031
1	4	1	0.2	0.5	0.5	0.15	0.15	0.15	0.25	0.00	0.00	0.088	-0.219	0.039
1	4	1	0.2	0.5	0.5	0.15	0.15	0.15	0.25	0.00	0.25	0.095	-0.195	0.022
1	4	1	0.2	0.5	0.5	0.15	0.15	0.15	0.25	0.00	0.50	0.065	-0.211	0.036
1	4	1	0.2	0.5	0.5	0.15	0.15	0.15	0.25	0.25	0.00	0.080	-0.216	0.033
1	4	1	0.2	0.5	0.5	0.15	0.15	0.15	0.25	0.25	0.25	0.067	-0.215	0.033
1	4	1	0.2	0.5	0.5	0.15	0.15	0.15	0.25	0.25	0.50	0.072	-0.237	0.031
1	4	1	0.2	0.5	0.5	0.15	0.15	0.15	0.25	0.50	0.00	0.078	-0.242	0.021
1	4	1	0.2	0.5	0.5	0.15	0.15	0.15	0.25	0.50	0.25	0.080	-0.207	0.028
1	4	1	0.2	0.5	0.5	0.15	0.15	0.15	0.25	0.50	0.50	0.085	-0.210	0.044
1	4	1	0.2	0.5	0.5	0.15	0.15	0.15	0.50	0.00	0.00	0.073	-0.185	0.031
1	4	1	0.2	0.5	0.5	0.15	0.15	0.15	0.50	0.00	0.25	0.071	-0.228	0.033

(continued)

												Mean Bias		
N	m_1	$\frac{m_2}{m_1}$	$E(C)$	$E(R)$	$E(U)$	σ_C	σ_R	σ_U	ρ_{CR}	ρ_{CU}	ρ_{RU}	c	r	u
1	4	1	0.2	0.5	0.5	0.15	0.15	0.15	0.50	0.00	0.50	0.075	-0.209	0.034
1	4	1	0.2	0.5	0.5	0.15	0.15	0.15	0.50	0.25	0.00	0.098	-0.218	0.038
1	4	1	0.2	0.5	0.5	0.15	0.15	0.15	0.50	0.25	0.25	0.059	-0.226	0.036
1	4	1	0.2	0.5	0.5	0.15	0.15	0.15	0.50	0.25	0.50	0.083	-0.223	0.034
1	4	1	0.2	0.5	0.5	0.15	0.15	0.15	0.50	0.50	0.00	0.075	-0.227	0.047
1	4	1	0.2	0.5	0.5	0.15	0.15	0.15	0.50	0.50	0.25	0.084	-0.209	0.035
1	4	1	0.2	0.5	0.5	0.15	0.15	0.15	0.50	0.50	0.50	0.069	-0.219	0.035
1	4	1	0.2	0.5	0.5	0.30	0.30	0.30	0.00	0.00	0.00	0.081	-0.266	0.010
1	4	1	0.2	0.5	0.5	0.30	0.30	0.30	0.00	0.00	0.25	0.120	-0.247	0.032
1	4	1	0.2	0.5	0.5	0.30	0.30	0.30	0.00	0.00	0.50	0.103	-0.248	0.004
1	4	1	0.2	0.5	0.5	0.30	0.30	0.30	0.00	0.25	0.00	0.116	-0.255	0.033
1	4	1	0.2	0.5	0.5	0.30	0.30	0.30	0.00	0.25	0.25	0.117	-0.242	0.042
1	4	1	0.2	0.5	0.5	0.30	0.30	0.30	0.00	0.25	0.50	0.111	-0.253	0.039
1	4	1	0.2	0.5	0.5	0.30	0.30	0.30	0.00	0.50	0.00	0.122	-0.266	0.025
1	4	1	0.2	0.5	0.5	0.30	0.30	0.30	0.00	0.50	0.25	0.118	-0.257	0.033
1	4	1	0.2	0.5	0.5	0.30	0.30	0.30	0.00	0.50	0.50	0.116	-0.260	0.031
1	4	1	0.2	0.5	0.5	0.30	0.30	0.30	0.25	0.00	0.00	0.104	-0.221	0.021
1	4	1	0.2	0.5	0.5	0.30	0.30	0.30	0.25	0.00	0.25	0.102	-0.227	0.026
1	4	1	0.2	0.5	0.5	0.30	0.30	0.30	0.25	0.00	0.50	0.107	-0.219	0.010
1	4	1	0.2	0.5	0.5	0.30	0.30	0.30	0.25	0.25	0.00	0.113	-0.247	0.038
1	4	1	0.2	0.5	0.5	0.30	0.30	0.30	0.25	0.25	0.25	0.119	-0.225	0.034
1	4	1	0.2	0.5	0.5	0.30	0.30	0.30	0.25	0.25	0.50	0.126	-0.216	0.030
1	4	1	0.2	0.5	0.5	0.30	0.30	0.30	0.25	0.50	0.00	0.132	-0.225	0.036
1	4	1	0.2	0.5	0.5	0.30	0.30	0.30	0.25	0.50	0.25	0.145	-0.214	0.045
1	4	1	0.2	0.5	0.5	0.30	0.30	0.30	0.25	0.50	0.50	0.125	-0.231	0.035
1	4	1	0.2	0.5	0.5	0.30	0.30	0.30	0.50	0.00	0.00	0.104	-0.207	0.039
1	4	1	0.2	0.5	0.5	0.30	0.30	0.30	0.50	0.00	0.25	0.096	-0.205	0.027
1	4	1	0.2	0.5	0.5	0.30	0.30	0.30	0.50	0.00	0.50	0.105	-0.189	0.035
1	4	1	0.2	0.5	0.5	0.30	0.30	0.30	0.50	0.25	0.00	0.134	-0.202	0.034
1	4	1	0.2	0.5	0.5	0.30	0.30	0.30	0.50	0.25	0.25	0.120	-0.218	0.023
1	4	1	0.2	0.5	0.5	0.30	0.30	0.30	0.50	0.25	0.50	0.103	-0.221	0.021
1	4	1	0.2	0.5	0.5	0.30	0.30	0.30	0.50	0.50	0.00	0.124	-0.227	0.050
1	4	1	0.2	0.5	0.5	0.30	0.30	0.30	0.50	0.50	0.25	0.117	-0.231	0.030
1	4	1	0.2	0.5	0.5	0.30	0.30	0.30	0.50	0.50	0.50	0.108	-0.194	0.035
1	4	1	0.2	0.8	0.5	0.00	0.00	0.00	0.00	0.00	0.00	0.068	-0.380	0.031
1	4	1	0.2	0.8	0.5	0.15	0.15	0.15	0.00	0.00	0.00	0.103	-0.391	0.039
1	4	1	0.2	0.8	0.5	0.15	0.15	0.15	0.00	0.00	0.25	0.088	-0.399	0.043
1	4	1	0.2	0.8	0.5	0.15	0.15	0.15	0.00	0.00	0.50	0.106	-0.380	0.040
1	4	1	0.2	0.8	0.5	0.15	0.15	0.15	0.00	0.25	0.00	0.102	-0.440	0.042

(continued)

N	m_1	$\frac{m_2}{m_1}$	$E(C)$	$E(R)$	$E(U)$	σ_C	σ_R	σ_U	ρ_{CR}	ρ_{CU}	ρ_{RU}	Mean Bias c	Mean Bias r	Mean Bias u
1	4	1	0.2	0.8	0.5	0.15	0.15	0.15	0.00	0.25	0.25	0.088	-0.417	0.043
1	4	1	0.2	0.8	0.5	0.15	0.15	0.15	0.00	0.25	0.50	0.100	-0.391	0.033
1	4	1	0.2	0.8	0.5	0.15	0.15	0.15	0.00	0.50	0.00	0.086	-0.396	0.040
1	4	1	0.2	0.8	0.5	0.15	0.15	0.15	0.00	0.50	0.25	0.095	-0.441	0.030
1	4	1	0.2	0.8	0.5	0.15	0.15	0.15	0.00	0.50	0.50	0.093	-0.402	0.038
1	4	1	0.2	0.8	0.5	0.15	0.15	0.15	0.25	0.00	0.00	0.077	-0.403	0.035
1	4	1	0.2	0.8	0.5	0.15	0.15	0.15	0.25	0.00	0.25	0.080	-0.400	0.033
1	4	1	0.2	0.8	0.5	0.15	0.15	0.15	0.25	0.00	0.50	0.097	-0.387	0.030
1	4	1	0.2	0.8	0.5	0.15	0.15	0.15	0.25	0.25	0.00	0.107	-0.382	0.045
1	4	1	0.2	0.8	0.5	0.15	0.15	0.15	0.25	0.25	0.25	0.101	-0.404	0.041
1	4	1	0.2	0.8	0.5	0.15	0.15	0.15	0.25	0.25	0.50	0.109	-0.379	0.034
1	4	1	0.2	0.8	0.5	0.15	0.15	0.15	0.25	0.50	0.00	0.097	-0.390	0.036
1	4	1	0.2	0.8	0.5	0.15	0.15	0.15	0.25	0.50	0.25	0.097	-0.405	0.038
1	4	1	0.2	0.8	0.5	0.15	0.15	0.15	0.25	0.50	0.50	0.112	-0.400	0.047
1	4	1	0.2	0.8	0.5	0.15	0.15	0.15	0.50	0.00	0.00	0.094	-0.391	0.036
1	4	1	0.2	0.8	0.5	0.15	0.15	0.15	0.50	0.00	0.25	0.096	-0.389	0.035
1	4	1	0.2	0.8	0.5	0.15	0.15	0.15	0.50	0.00	0.50	0.100	-0.405	0.037
1	4	1	0.2	0.8	0.5	0.15	0.15	0.15	0.50	0.25	0.00	0.089	-0.401	0.048
1	4	1	0.2	0.8	0.5	0.15	0.15	0.15	0.50	0.25	0.25	0.105	-0.379	0.034
1	4	1	0.2	0.8	0.5	0.15	0.15	0.15	0.50	0.25	0.50	0.091	-0.398	0.050
1	4	1	0.2	0.8	0.5	0.15	0.15	0.15	0.50	0.50	0.00	0.085	-0.385	0.031
1	4	1	0.2	0.8	0.5	0.15	0.15	0.15	0.50	0.50	0.25	0.101	-0.389	0.036
1	4	1	0.2	0.8	0.5	0.15	0.15	0.15	0.50	0.50	0.50	0.088	-0.398	0.039
1	4	1	0.2	0.8	0.5	0.30	0.30	0.30	0.00	0.00	0.00	0.105	-0.485	0.029
1	4	1	0.2	0.8	0.5	0.30	0.30	0.30	0.00	0.00	0.25	0.123	-0.452	0.039
1	4	1	0.2	0.8	0.5	0.30	0.30	0.30	0.00	0.00	0.50	0.107	-0.464	0.030
1	4	1	0.2	0.8	0.5	0.30	0.30	0.30	0.00	0.25	0.00	0.123	-0.490	0.019
1	4	1	0.2	0.8	0.5	0.30	0.30	0.30	0.00	0.25	0.25	0.130	-0.471	0.032
1	4	1	0.2	0.8	0.5	0.30	0.30	0.30	0.00	0.25	0.50	0.110	-0.459	0.028
1	4	1	0.2	0.8	0.5	0.30	0.30	0.30	0.00	0.50	0.00	0.119	-0.479	0.034
1	4	1	0.2	0.8	0.5	0.30	0.30	0.30	0.00	0.50	0.25	0.137	-0.462	0.023
1	4	1	0.2	0.8	0.5	0.30	0.30	0.30	0.00	0.50	0.50	0.128	-0.480	0.018
1	4	1	0.2	0.8	0.5	0.30	0.30	0.30	0.25	0.00	0.00	0.108	-0.446	0.035
1	4	1	0.2	0.8	0.5	0.30	0.30	0.30	0.25	0.00	0.25	0.111	-0.443	0.007
1	4	1	0.2	0.8	0.5	0.30	0.30	0.30	0.25	0.00	0.50	0.125	-0.436	0.049
1	4	1	0.2	0.8	0.5	0.30	0.30	0.30	0.25	0.25	0.00	0.136	-0.444	0.035
1	4	1	0.2	0.8	0.5	0.30	0.30	0.30	0.25	0.25	0.25	0.128	-0.438	0.023
1	4	1	0.2	0.8	0.5	0.30	0.30	0.30	0.25	0.25	0.50	0.128	-0.435	0.019
1	4	1	0.2	0.8	0.5	0.30	0.30	0.30	0.25	0.50	0.00	0.132	-0.463	0.031

(continued)

												Mean Bias		
N	m_1	$\frac{m_2}{m_1}$	$E(C)$	$E(R)$	$E(U)$	σ_C	σ_R	σ_U	ρ_{CR}	ρ_{CU}	ρ_{RU}	c	r	u
1	4	1	0.2	0.8	0.5	0.30	0.30	0.30	0.25	0.50	0.25	0.130	-0.460	0.037
1	4	1	0.2	0.8	0.5	0.30	0.30	0.30	0.25	0.50	0.50	0.138	-0.478	0.012
1	4	1	0.2	0.8	0.5	0.30	0.30	0.30	0.50	0.00	0.00	0.123	-0.436	0.021
1	4	1	0.2	0.8	0.5	0.30	0.30	0.30	0.50	0.00	0.25	0.143	-0.408	0.009
1	4	1	0.2	0.8	0.5	0.30	0.30	0.30	0.50	0.00	0.50	0.127	-0.421	0.021
1	4	1	0.2	0.8	0.5	0.30	0.30	0.30	0.50	0.25	0.00	0.147	-0.421	0.039
1	4	1	0.2	0.8	0.5	0.30	0.30	0.30	0.50	0.25	0.25	0.120	-0.443	0.022
1	4	1	0.2	0.8	0.5	0.30	0.30	0.30	0.50	0.25	0.50	0.128	-0.443	0.055
1	4	1	0.2	0.8	0.5	0.30	0.30	0.30	0.50	0.50	0.00	0.131	-0.438	0.045
1	4	1	0.2	0.8	0.5	0.30	0.30	0.30	0.50	0.50	0.25	0.147	-0.432	0.022
1	4	1	0.2	0.8	0.5	0.30	0.30	0.30	0.50	0.50	0.50	0.116	-0.442	0.033
1	4	1	0.5	0.2	0.5	0.00	0.00	0.00	0.00	0.00	0.00	-0.010	-0.002	0.016
1	4	1	0.5	0.2	0.5	0.15	0.15	0.15	0.00	0.00	0.00	0.004	0.002	0.015
1	4	1	0.5	0.2	0.5	0.15	0.15	0.15	0.00	0.00	0.25	-0.009	0.009	0.017
1	4	1	0.5	0.2	0.5	0.15	0.15	0.15	0.00	0.00	0.50	-0.005	-0.006	0.020
1	4	1	0.5	0.2	0.5	0.15	0.15	0.15	0.00	0.25	0.00	-0.012	-0.024	0.000
1	4	1	0.5	0.2	0.5	0.15	0.15	0.15	0.00	0.25	0.25	-0.014	0.021	0.001
1	4	1	0.5	0.2	0.5	0.15	0.15	0.15	0.00	0.25	0.50	-0.009	-0.010	0.017
1	4	1	0.5	0.2	0.5	0.15	0.15	0.15	0.00	0.50	0.00	0.002	0.004	0.007
1	4	1	0.5	0.2	0.5	0.15	0.15	0.15	0.00	0.50	0.25	-0.016	-0.007	0.007
1	4	1	0.5	0.2	0.5	0.15	0.15	0.15	0.00	0.50	0.50	0.004	-0.007	0.012
1	4	1	0.5	0.2	0.5	0.15	0.15	0.15	0.25	0.00	0.00	-0.013	0.002	0.004
1	4	1	0.5	0.2	0.5	0.15	0.15	0.15	0.25	0.00	0.25	0.001	-0.003	-0.001
1	4	1	0.5	0.2	0.5	0.15	0.15	0.15	0.25	0.00	0.50	-0.008	0.002	0.021
1	4	1	0.5	0.2	0.5	0.15	0.15	0.15	0.25	0.25	0.00	-0.011	-0.010	0.005
1	4	1	0.5	0.2	0.5	0.15	0.15	0.15	0.25	0.25	0.25	-0.008	0.006	0.009
1	4	1	0.5	0.2	0.5	0.15	0.15	0.15	0.25	0.25	0.50	-0.023	0.009	-0.005
1	4	1	0.5	0.2	0.5	0.15	0.15	0.15	0.25	0.50	0.00	-0.005	-0.006	0.022
1	4	1	0.5	0.2	0.5	0.15	0.15	0.15	0.25	0.50	0.25	-0.004	-0.013	-0.001
1	4	1	0.5	0.2	0.5	0.15	0.15	0.15	0.25	0.50	0.50	-0.015	-0.009	0.010
1	4	1	0.5	0.2	0.5	0.15	0.15	0.15	0.50	0.00	0.00	0.001	0.014	0.005
1	4	1	0.5	0.2	0.5	0.15	0.15	0.15	0.50	0.00	0.25	-0.003	0.023	0.009
1	4	1	0.5	0.2	0.5	0.15	0.15	0.15	0.50	0.00	0.50	-0.025	-0.012	0.012
1	4	1	0.5	0.2	0.5	0.15	0.15	0.15	0.50	0.25	0.00	-0.001	-0.009	0.000
1	4	1	0.5	0.2	0.5	0.15	0.15	0.15	0.50	0.25	0.25	-0.011	0.015	0.008
1	4	1	0.5	0.2	0.5	0.15	0.15	0.15	0.50	0.25	0.50	0.004	0.012	0.004
1	4	1	0.5	0.2	0.5	0.15	0.15	0.15	0.50	0.50	0.00	0.009	0.019	0.015
1	4	1	0.5	0.2	0.5	0.15	0.15	0.15	0.50	0.50	0.25	0.003	-0.004	0.019
1	4	1	0.5	0.2	0.5	0.15	0.15	0.15	0.50	0.50	0.50	-0.010	0.006	0.028

(continued)

												Mean Bias		
N	m_1	$\frac{m_2}{m_1}$	$E(C)$	$E(R)$	$E(U)$	σ_C	σ_R	σ_U	ρ_{CR}	ρ_{CU}	ρ_{RU}	c	r	u
1	4	1	0.5	0.2	0.5	0.30	0.30	0.30	0.00	0.00	0.00	0.020	-0.004	0.020
1	4	1	0.5	0.2	0.5	0.30	0.30	0.30	0.00	0.00	0.25	0.017	0.019	0.004
1	4	1	0.5	0.2	0.5	0.30	0.30	0.30	0.00	0.00	0.50	-0.021	0.020	0.021
1	4	1	0.5	0.2	0.5	0.30	0.30	0.30	0.00	0.25	0.00	0.031	-0.017	0.035
1	4	1	0.5	0.2	0.5	0.30	0.30	0.30	0.00	0.25	0.25	0.008	0.012	0.026
1	4	1	0.5	0.2	0.5	0.30	0.30	0.30	0.00	0.25	0.50	0.002	0.013	0.021
1	4	1	0.5	0.2	0.5	0.30	0.30	0.30	0.00	0.50	0.00	0.047	-0.022	0.026
1	4	1	0.5	0.2	0.5	0.30	0.30	0.30	0.00	0.50	0.25	0.040	-0.003	0.029
1	4	1	0.5	0.2	0.5	0.30	0.30	0.30	0.00	0.50	0.50	0.041	0.006	0.012
1	4	1	0.5	0.2	0.5	0.30	0.30	0.30	0.25	0.00	0.00	0.016	0.009	0.013
1	4	1	0.5	0.2	0.5	0.30	0.30	0.30	0.25	0.00	0.25	0.003	0.016	0.014
1	4	1	0.5	0.2	0.5	0.30	0.30	0.30	0.25	0.00	0.50	0.010	0.033	0.008
1	4	1	0.5	0.2	0.5	0.30	0.30	0.30	0.25	0.25	0.00	0.020	0.003	0.013
1	4	1	0.5	0.2	0.5	0.30	0.30	0.30	0.25	0.25	0.25	0.039	0.022	0.010
1	4	1	0.5	0.2	0.5	0.30	0.30	0.30	0.25	0.25	0.50	0.025	0.015	0.016
1	4	1	0.5	0.2	0.5	0.30	0.30	0.30	0.25	0.50	0.00	0.036	0.003	0.015
1	4	1	0.5	0.2	0.5	0.30	0.30	0.30	0.25	0.50	0.25	0.039	0.021	0.021
1	4	1	0.5	0.2	0.5	0.30	0.30	0.30	0.25	0.50	0.50	0.021	0.016	0.012
1	4	1	0.5	0.2	0.5	0.30	0.30	0.30	0.50	0.00	0.00	-0.001	0.042	0.015
1	4	1	0.5	0.2	0.5	0.30	0.30	0.30	0.50	0.00	0.25	0.001	0.047	0.023
1	4	1	0.5	0.2	0.5	0.30	0.30	0.30	0.50	0.00	0.50	0.023	0.060	0.025
1	4	1	0.5	0.2	0.5	0.30	0.30	0.30	0.50	0.25	0.00	0.051	0.010	0.003
1	4	1	0.5	0.2	0.5	0.30	0.30	0.30	0.50	0.25	0.25	0.019	0.024	0.011
1	4	1	0.5	0.2	0.5	0.30	0.30	0.30	0.50	0.25	0.50	0.022	0.035	0.031
1	4	1	0.5	0.2	0.5	0.30	0.30	0.30	0.50	0.50	0.00	0.035	0.002	0.005
1	4	1	0.5	0.2	0.5	0.30	0.30	0.30	0.50	0.50	0.25	0.051	0.015	0.026
1	4	1	0.5	0.2	0.5	0.30	0.30	0.30	0.50	0.50	0.50	0.046	0.011	-0.005
1	4	1	0.5	0.5	0.5	0.00	0.00	0.00	0.00	0.00	0.00	0.018	-0.036	0.014
1	4	1	0.5	0.5	0.5	0.15	0.15	0.15	0.00	0.00	0.00	0.025	-0.021	0.024
1	4	1	0.5	0.5	0.5	0.15	0.15	0.15	0.00	0.00	0.25	0.022	-0.047	0.032
1	4	1	0.5	0.5	0.5	0.15	0.15	0.15	0.00	0.00	0.50	0.031	-0.049	0.014
1	4	1	0.5	0.5	0.5	0.15	0.15	0.15	0.00	0.25	0.00	0.014	-0.047	0.011
1	4	1	0.5	0.5	0.5	0.15	0.15	0.15	0.00	0.25	0.25	0.011	-0.041	0.024
1	4	1	0.5	0.5	0.5	0.15	0.15	0.15	0.00	0.25	0.50	0.016	-0.066	0.012
1	4	1	0.5	0.5	0.5	0.15	0.15	0.15	0.00	0.50	0.00	0.038	-0.041	0.020
1	4	1	0.5	0.5	0.5	0.15	0.15	0.15	0.00	0.50	0.25	-0.001	-0.043	0.008
1	4	1	0.5	0.5	0.5	0.15	0.15	0.15	0.00	0.50	0.50	0.009	-0.041	0.015
1	4	1	0.5	0.5	0.5	0.15	0.15	0.15	0.25	0.00	0.00	0.032	-0.037	0.008
1	4	1	0.5	0.5	0.5	0.15	0.15	0.15	0.25	0.00	0.25	0.020	-0.036	0.021

(continued)

												Mean Bias		
N	m_1	$\frac{m_2}{m_1}$	$E(C)$	$E(R)$	$E(U)$	σ_C	σ_R	σ_U	ρ_{CR}	ρ_{CU}	ρ_{RU}	c	r	u
1	4	1	0.5	0.5	0.5	0.15	0.15	0.15	0.25	0.00	0.50	0.018	-0.054	0.008
1	4	1	0.5	0.5	0.5	0.15	0.15	0.15	0.25	0.25	0.00	0.030	-0.030	0.033
1	4	1	0.5	0.5	0.5	0.15	0.15	0.15	0.25	0.25	0.25	-0.001	-0.033	0.015
1	4	1	0.5	0.5	0.5	0.15	0.15	0.15	0.25	0.25	0.50	0.021	-0.034	0.023
1	4	1	0.5	0.5	0.5	0.15	0.15	0.15	0.25	0.50	0.00	0.028	-0.042	0.009
1	4	1	0.5	0.5	0.5	0.15	0.15	0.15	0.25	0.50	0.25	0.033	-0.038	0.015
1	4	1	0.5	0.5	0.5	0.15	0.15	0.15	0.25	0.50	0.50	0.021	-0.033	0.019
1	4	1	0.5	0.5	0.5	0.15	0.15	0.15	0.50	0.00	0.00	0.019	-0.032	0.006
1	4	1	0.5	0.5	0.5	0.15	0.15	0.15	0.50	0.00	0.25	0.013	-0.041	0.023
1	4	1	0.5	0.5	0.5	0.15	0.15	0.15	0.50	0.00	0.50	0.018	-0.061	0.013
1	4	1	0.5	0.5	0.5	0.15	0.15	0.15	0.50	0.25	0.00	0.035	-0.027	0.016
1	4	1	0.5	0.5	0.5	0.15	0.15	0.15	0.50	0.25	0.25	0.002	-0.063	0.015
1	4	1	0.5	0.5	0.5	0.15	0.15	0.15	0.50	0.25	0.50	0.021	-0.043	0.006
1	4	1	0.5	0.5	0.5	0.15	0.15	0.15	0.50	0.50	0.00	0.026	-0.028	0.008
1	4	1	0.5	0.5	0.5	0.15	0.15	0.15	0.50	0.50	0.25	0.027	-0.044	0.027
1	4	1	0.5	0.5	0.5	0.15	0.15	0.15	0.50	0.50	0.50	0.029	-0.028	0.026
1	4	1	0.5	0.5	0.5	0.30	0.30	0.30	0.00	0.00	0.00	0.052	-0.089	0.030
1	4	1	0.5	0.5	0.5	0.30	0.30	0.30	0.00	0.00	0.25	0.027	-0.078	0.000
1	4	1	0.5	0.5	0.5	0.30	0.30	0.30	0.00	0.00	0.50	0.025	-0.098	0.014
1	4	1	0.5	0.5	0.5	0.30	0.30	0.30	0.00	0.25	0.00	0.049	-0.070	0.021
1	4	1	0.5	0.5	0.5	0.30	0.30	0.30	0.00	0.25	0.25	0.037	-0.109	0.016
1	4	1	0.5	0.5	0.5	0.30	0.30	0.30	0.00	0.25	0.50	0.044	-0.085	0.016
1	4	1	0.5	0.5	0.5	0.30	0.30	0.30	0.00	0.50	0.00	0.065	-0.099	0.005
1	4	1	0.5	0.5	0.5	0.30	0.30	0.30	0.00	0.50	0.25	0.063	-0.117	0.013
1	4	1	0.5	0.5	0.5	0.30	0.30	0.30	0.00	0.50	0.50	0.056	-0.092	0.003
1	4	1	0.5	0.5	0.5	0.30	0.30	0.30	0.25	0.00	0.00	0.044	-0.074	0.021
1	4	1	0.5	0.5	0.5	0.30	0.30	0.30	0.25	0.00	0.25	0.043	-0.064	0.009
1	4	1	0.5	0.5	0.5	0.30	0.30	0.30	0.25	0.00	0.50	0.029	-0.064	0.002
1	4	1	0.5	0.5	0.5	0.30	0.30	0.30	0.25	0.25	0.00	0.034	-0.079	0.004
1	4	1	0.5	0.5	0.5	0.30	0.30	0.30	0.25	0.25	0.25	0.035	-0.053	0.005
1	4	1	0.5	0.5	0.5	0.30	0.30	0.30	0.25	0.25	0.50	0.036	-0.058	0.005
1	4	1	0.5	0.5	0.5	0.30	0.30	0.30	0.25	0.50	0.00	0.055	-0.092	0.007
1	4	1	0.5	0.5	0.5	0.30	0.30	0.30	0.25	0.50	0.25	0.091	-0.069	0.007
1	4	1	0.5	0.5	0.5	0.30	0.30	0.30	0.25	0.50	0.50	0.069	-0.079	0.030
1	4	1	0.5	0.5	0.5	0.30	0.30	0.30	0.50	0.00	0.00	0.039	-0.062	0.023
1	4	1	0.5	0.5	0.5	0.30	0.30	0.30	0.50	0.00	0.25	0.024	-0.063	0.020
1	4	1	0.5	0.5	0.5	0.30	0.30	0.30	0.50	0.00	0.50	0.044	-0.035	0.016
1	4	1	0.5	0.5	0.5	0.30	0.30	0.30	0.50	0.25	0.00	0.066	-0.044	0.010
1	4	1	0.5	0.5	0.5	0.30	0.30	0.30	0.50	0.25	0.25	0.044	-0.044	0.015

(continued)

												Mean Bias		
N	m_1	$\frac{m_2}{m_1}$	$E(C)$	$E(R)$	$E(U)$	σ_C	σ_R	σ_U	ρ_{CR}	ρ_{CU}	ρ_{RU}	c	r	u
1	4	1	0.5	0.5	0.5	0.30	0.30	0.30	0.50	0.25	0.50	0.036	-0.060	-0.003
1	4	1	0.5	0.5	0.5	0.30	0.30	0.30	0.50	0.50	0.00	0.058	-0.066	0.019
1	4	1	0.5	0.5	0.5	0.30	0.30	0.30	0.50	0.50	0.25	0.066	-0.074	0.015
1	4	1	0.5	0.5	0.5	0.30	0.30	0.30	0.50	0.50	0.50	0.069	-0.027	0.026
1	4	1	0.5	0.8	0.5	0.00	0.00	0.00	0.00	0.00	0.00	0.040	-0.094	0.026
1	4	1	0.5	0.8	0.5	0.15	0.15	0.15	0.00	0.00	0.00	0.049	-0.113	0.028
1	4	1	0.5	0.8	0.5	0.15	0.15	0.15	0.00	0.00	0.25	0.059	-0.137	0.027
1	4	1	0.5	0.8	0.5	0.15	0.15	0.15	0.00	0.00	0.50	0.061	-0.123	0.030
1	4	1	0.5	0.8	0.5	0.15	0.15	0.15	0.00	0.25	0.00	0.075	-0.098	0.024
1	4	1	0.5	0.8	0.5	0.15	0.15	0.15	0.00	0.25	0.25	0.040	-0.143	0.032
1	4	1	0.5	0.8	0.5	0.15	0.15	0.15	0.00	0.25	0.50	0.054	-0.119	0.039
1	4	1	0.5	0.8	0.5	0.15	0.15	0.15	0.00	0.50	0.00	0.043	-0.108	0.034
1	4	1	0.5	0.8	0.5	0.15	0.15	0.15	0.00	0.50	0.25	0.056	-0.129	0.033
1	4	1	0.5	0.8	0.5	0.15	0.15	0.15	0.00	0.50	0.50	0.050	-0.141	0.026
1	4	1	0.5	0.8	0.5	0.15	0.15	0.15	0.25	0.00	0.00	0.041	-0.137	0.029
1	4	1	0.5	0.8	0.5	0.15	0.15	0.15	0.25	0.00	0.25	0.057	-0.112	0.032
1	4	1	0.5	0.8	0.5	0.15	0.15	0.15	0.25	0.00	0.50	0.045	-0.128	0.021
1	4	1	0.5	0.8	0.5	0.15	0.15	0.15	0.25	0.25	0.00	0.060	-0.117	0.027
1	4	1	0.5	0.8	0.5	0.15	0.15	0.15	0.25	0.25	0.25	0.058	-0.103	0.025
1	4	1	0.5	0.8	0.5	0.15	0.15	0.15	0.25	0.25	0.50	0.037	-0.127	0.023
1	4	1	0.5	0.8	0.5	0.15	0.15	0.15	0.25	0.50	0.00	0.039	-0.114	0.028
1	4	1	0.5	0.8	0.5	0.15	0.15	0.15	0.25	0.50	0.25	0.072	-0.107	0.025
1	4	1	0.5	0.8	0.5	0.15	0.15	0.15	0.25	0.50	0.50	0.063	-0.107	0.030
1	4	1	0.5	0.8	0.5	0.15	0.15	0.15	0.50	0.00	0.00	0.067	-0.105	0.036
1	4	1	0.5	0.8	0.5	0.15	0.15	0.15	0.50	0.00	0.25	0.047	-0.121	0.021
1	4	1	0.5	0.8	0.5	0.15	0.15	0.15	0.50	0.00	0.50	0.059	-0.106	0.033
1	4	1	0.5	0.8	0.5	0.15	0.15	0.15	0.50	0.25	0.00	0.060	-0.114	0.033
1	4	1	0.5	0.8	0.5	0.15	0.15	0.15	0.50	0.25	0.25	0.056	-0.109	0.040
1	4	1	0.5	0.8	0.5	0.15	0.15	0.15	0.50	0.25	0.50	0.037	-0.131	0.027
1	4	1	0.5	0.8	0.5	0.15	0.15	0.15	0.50	0.50	0.00	0.042	-0.107	0.035
1	4	1	0.5	0.8	0.5	0.15	0.15	0.15	0.50	0.50	0.25	0.057	-0.123	0.027
1	4	1	0.5	0.8	0.5	0.15	0.15	0.15	0.50	0.50	0.50	0.047	-0.122	0.031
1	4	1	0.5	0.8	0.5	0.30	0.30	0.30	0.00	0.00	0.00	0.064	-0.207	0.020
1	4	1	0.5	0.8	0.5	0.30	0.30	0.30	0.00	0.00	0.25	0.082	-0.183	0.024
1	4	1	0.5	0.8	0.5	0.30	0.30	0.30	0.00	0.00	0.50	0.067	-0.160	0.023
1	4	1	0.5	0.8	0.5	0.30	0.30	0.30	0.00	0.25	0.00	0.093	-0.191	0.012
1	4	1	0.5	0.8	0.5	0.30	0.30	0.30	0.00	0.25	0.25	0.095	-0.174	0.022
1	4	1	0.5	0.8	0.5	0.30	0.30	0.30	0.00	0.25	0.50	0.078	-0.202	0.007
1	4	1	0.5	0.8	0.5	0.30	0.30	0.30	0.00	0.50	0.00	0.104	-0.192	0.012

(continued)

												Mean Bias		
N	m_1	$\frac{m_2}{m_1}$	$E(C)$	$E(R)$	$E(U)$	σ_C	σ_R	σ_U	ρ_{CR}	ρ_{CU}	ρ_{RU}	c	r	u
1	4	1	0.5	0.8	0.5	0.30	0.30	0.30	0.00	0.50	0.25	0.085	-0.186	0.044
1	4	1	0.5	0.8	0.5	0.30	0.30	0.30	0.00	0.50	0.50	0.086	-0.167	0.048
1	4	1	0.5	0.8	0.5	0.30	0.30	0.30	0.25	0.00	0.00	0.080	-0.170	0.027
1	4	1	0.5	0.8	0.5	0.30	0.30	0.30	0.25	0.00	0.25	0.065	-0.183	0.030
1	4	1	0.5	0.8	0.5	0.30	0.30	0.30	0.25	0.00	0.50	0.090	-0.121	0.020
1	4	1	0.5	0.8	0.5	0.30	0.30	0.30	0.25	0.25	0.00	0.083	-0.164	0.009
1	4	1	0.5	0.8	0.5	0.30	0.30	0.30	0.25	0.25	0.25	0.083	-0.186	0.018
1	4	1	0.5	0.8	0.5	0.30	0.30	0.30	0.25	0.25	0.50	0.062	-0.197	0.017
1	4	1	0.5	0.8	0.5	0.30	0.30	0.30	0.25	0.50	0.00	0.092	-0.174	0.027
1	4	1	0.5	0.8	0.5	0.30	0.30	0.30	0.25	0.50	0.25	0.096	-0.175	0.031
1	4	1	0.5	0.8	0.5	0.30	0.30	0.30	0.25	0.50	0.50	0.087	-0.185	0.027
1	4	1	0.5	0.8	0.5	0.30	0.30	0.30	0.50	0.00	0.00	0.093	-0.169	0.010
1	4	1	0.5	0.8	0.5	0.30	0.30	0.30	0.50	0.00	0.25	0.054	-0.165	0.023
1	4	1	0.5	0.8	0.5	0.30	0.30	0.30	0.50	0.00	0.50	0.057	-0.157	0.029
1	4	1	0.5	0.8	0.5	0.30	0.30	0.30	0.50	0.25	0.00	0.073	-0.168	0.020
1	4	1	0.5	0.8	0.5	0.30	0.30	0.30	0.50	0.25	0.25	0.086	-0.147	0.033
1	4	1	0.5	0.8	0.5	0.30	0.30	0.30	0.50	0.25	0.50	0.113	-0.154	0.000
1	4	1	0.5	0.8	0.5	0.30	0.30	0.30	0.50	0.50	0.00	0.083	-0.192	0.019
1	4	1	0.5	0.8	0.5	0.30	0.30	0.30	0.50	0.50	0.25	0.091	-0.166	0.022
1	4	1	0.5	0.8	0.5	0.30	0.30	0.30	0.50	0.50	0.50	0.087	-0.178	0.020
1	4	1	0.8	0.2	0.5	0.00	0.00	0.00	0.00	0.00	0.00	-0.054	0.020	0.000
1	4	1	0.8	0.2	0.5	0.15	0.15	0.15	0.00	0.00	0.00	-0.032	0.011	-0.010
1	4	1	0.8	0.2	0.5	0.15	0.15	0.15	0.00	0.00	0.25	-0.073	0.023	-0.008
1	4	1	0.8	0.2	0.5	0.15	0.15	0.15	0.00	0.00	0.50	-0.041	0.001	0.004
1	4	1	0.8	0.2	0.5	0.15	0.15	0.15	0.00	0.25	0.00	-0.038	0.024	-0.004
1	4	1	0.8	0.2	0.5	0.15	0.15	0.15	0.00	0.25	0.25	-0.030	0.021	-0.010
1	4	1	0.8	0.2	0.5	0.15	0.15	0.15	0.00	0.25	0.50	-0.041	0.033	0.015
1	4	1	0.8	0.2	0.5	0.15	0.15	0.15	0.00	0.50	0.00	-0.021	0.018	0.009
1	4	1	0.8	0.2	0.5	0.15	0.15	0.15	0.00	0.50	0.25	-0.030	0.026	0.012
1	4	1	0.8	0.2	0.5	0.15	0.15	0.15	0.00	0.50	0.50	-0.039	0.015	0.005
1	4	1	0.8	0.2	0.5	0.15	0.15	0.15	0.25	0.00	0.00	-0.035	0.013	-0.001
1	4	1	0.8	0.2	0.5	0.15	0.15	0.15	0.25	0.00	0.25	-0.038	0.014	0.014
1	4	1	0.8	0.2	0.5	0.15	0.15	0.15	0.25	0.00	0.50	-0.050	0.020	-0.018
1	4	1	0.8	0.2	0.5	0.15	0.15	0.15	0.25	0.25	0.00	-0.030	0.018	0.008
1	4	1	0.8	0.2	0.5	0.15	0.15	0.15	0.25	0.25	0.25	-0.043	0.026	0.004
1	4	1	0.8	0.2	0.5	0.15	0.15	0.15	0.25	0.25	0.50	-0.030	0.024	-0.011
1	4	1	0.8	0.2	0.5	0.15	0.15	0.15	0.25	0.50	0.00	-0.018	0.030	0.004
1	4	1	0.8	0.2	0.5	0.15	0.15	0.15	0.25	0.50	0.25	-0.047	0.018	-0.011
1	4	1	0.8	0.2	0.5	0.15	0.15	0.15	0.25	0.50	0.50	-0.036	-0.002	-0.004

(continued)

N	m_1	$\frac{m_2}{m_1}$	$E(C)$	$E(R)$	$E(U)$	σ_C	σ_R	σ_U	ρ_{CR}	ρ_{CU}	ρ_{RU}	Mean Bias c	Mean Bias r	Mean Bias u
1	4	1	0.8	0.2	0.5	0.15	0.15	0.15	0.50	0.00	0.00	-0.036	0.006	-0.008
1	4	1	0.8	0.2	0.5	0.15	0.15	0.15	0.50	0.00	0.25	-0.059	0.024	0.006
1	4	1	0.8	0.2	0.5	0.15	0.15	0.15	0.50	0.00	0.50	-0.032	0.019	-0.008
1	4	1	0.8	0.2	0.5	0.15	0.15	0.15	0.50	0.25	0.00	-0.028	0.004	-0.001
1	4	1	0.8	0.2	0.5	0.15	0.15	0.15	0.50	0.25	0.25	-0.040	0.012	0.009
1	4	1	0.8	0.2	0.5	0.15	0.15	0.15	0.50	0.25	0.50	-0.034	0.032	0.005
1	4	1	0.8	0.2	0.5	0.15	0.15	0.15	0.50	0.50	0.00	-0.022	0.008	-0.003
1	4	1	0.8	0.2	0.5	0.15	0.15	0.15	0.50	0.50	0.25	-0.021	0.011	-0.008
1	4	1	0.8	0.2	0.5	0.15	0.15	0.15	0.50	0.50	0.50	-0.049	0.030	0.000
1	4	1	0.8	0.2	0.5	0.30	0.30	0.30	0.00	0.00	0.00	-0.060	0.007	0.001
1	4	1	0.8	0.2	0.5	0.30	0.30	0.30	0.00	0.00	0.25	-0.044	0.022	0.011
1	4	1	0.8	0.2	0.5	0.30	0.30	0.30	0.00	0.00	0.50	-0.062	0.024	-0.003
1	4	1	0.8	0.2	0.5	0.30	0.30	0.30	0.00	0.25	0.00	-0.049	0.014	-0.006
1	4	1	0.8	0.2	0.5	0.30	0.30	0.30	0.00	0.25	0.25	-0.051	0.037	-0.008
1	4	1	0.8	0.2	0.5	0.30	0.30	0.30	0.00	0.25	0.50	-0.054	0.012	0.008
1	4	1	0.8	0.2	0.5	0.30	0.30	0.30	0.00	0.50	0.00	-0.001	-0.010	0.012
1	4	1	0.8	0.2	0.5	0.30	0.30	0.30	0.00	0.50	0.25	-0.036	0.036	0.008
1	4	1	0.8	0.2	0.5	0.30	0.30	0.30	0.00	0.50	0.50	-0.024	0.027	0.001
1	4	1	0.8	0.2	0.5	0.30	0.30	0.30	0.25	0.00	0.00	-0.043	0.030	0.015
1	4	1	0.8	0.2	0.5	0.30	0.30	0.30	0.25	0.00	0.25	-0.060	0.038	-0.017
1	4	1	0.8	0.2	0.5	0.30	0.30	0.30	0.25	0.00	0.50	-0.070	0.056	0.015
1	4	1	0.8	0.2	0.5	0.30	0.30	0.30	0.25	0.25	0.00	-0.053	0.034	0.009
1	4	1	0.8	0.2	0.5	0.30	0.30	0.30	0.25	0.25	0.25	-0.043	0.033	-0.004
1	4	1	0.8	0.2	0.5	0.30	0.30	0.30	0.25	0.25	0.50	-0.047	0.041	-0.002
1	4	1	0.8	0.2	0.5	0.30	0.30	0.30	0.25	0.50	0.00	-0.018	0.020	0.003
1	4	1	0.8	0.2	0.5	0.30	0.30	0.30	0.25	0.50	0.25	-0.028	0.044	0.023
1	4	1	0.8	0.2	0.5	0.30	0.30	0.30	0.25	0.50	0.50	-0.036	0.028	-0.015
1	4	1	0.8	0.2	0.5	0.30	0.30	0.30	0.50	0.00	0.00	-0.075	0.042	0.003
1	4	1	0.8	0.2	0.5	0.30	0.30	0.30	0.50	0.00	0.25	-0.070	0.031	0.013
1	4	1	0.8	0.2	0.5	0.30	0.30	0.30	0.50	0.00	0.50	-0.061	0.065	0.001
1	4	1	0.8	0.2	0.5	0.30	0.30	0.30	0.50	0.25	0.00	-0.032	0.047	0.016
1	4	1	0.8	0.2	0.5	0.30	0.30	0.30	0.50	0.25	0.25	-0.021	0.047	0.017
1	4	1	0.8	0.2	0.5	0.30	0.30	0.30	0.50	0.25	0.50	-0.033	0.044	0.009
1	4	1	0.8	0.2	0.5	0.30	0.30	0.30	0.50	0.50	0.00	-0.015	0.032	0.004
1	4	1	0.8	0.2	0.5	0.30	0.30	0.30	0.50	0.50	0.25	-0.013	0.024	0.004
1	4	1	0.8	0.2	0.5	0.30	0.30	0.30	0.50	0.50	0.50	-0.041	0.026	0.013
1	4	1	0.8	0.5	0.5	0.00	0.00	0.00	0.00	0.00	0.00	-0.009	0.011	0.017
1	4	1	0.8	0.5	0.5	0.15	0.15	0.15	0.00	0.00	0.00	-0.008	-0.009	0.017
1	4	1	0.8	0.5	0.5	0.15	0.15	0.15	0.00	0.00	0.25	-0.002	0.024	0.011

(continued)

												Mean Bias		
N	m_1	$\frac{m_2}{m_1}$	$E(C)$	$E(R)$	$E(U)$	σ_C	σ_R	σ_U	ρ_{CR}	ρ_{CU}	ρ_{RU}	c	r	u
1	4	1	0.8	0.5	0.5	0.15	0.15	0.15	0.00	0.00	0.50	-0.003	0.017	0.024
1	4	1	0.8	0.5	0.5	0.15	0.15	0.15	0.00	0.25	0.00	-0.011	0.023	0.014
1	4	1	0.8	0.5	0.5	0.15	0.15	0.15	0.00	0.25	0.25	-0.004	-0.002	0.011
1	4	1	0.8	0.5	0.5	0.15	0.15	0.15	0.00	0.25	0.50	-0.001	-0.008	0.027
1	4	1	0.8	0.5	0.5	0.15	0.15	0.15	0.00	0.50	0.00	-0.011	0.004	0.007
1	4	1	0.8	0.5	0.5	0.15	0.15	0.15	0.00	0.50	0.25	-0.007	0.017	0.000
1	4	1	0.8	0.5	0.5	0.15	0.15	0.15	0.00	0.50	0.50	-0.011	0.007	0.004
1	4	1	0.8	0.5	0.5	0.15	0.15	0.15	0.25	0.00	0.00	-0.002	0.015	0.018
1	4	1	0.8	0.5	0.5	0.15	0.15	0.15	0.25	0.00	0.25	0.001	0.032	0.004
1	4	1	0.8	0.5	0.5	0.15	0.15	0.15	0.25	0.00	0.50	-0.002	0.016	0.011
1	4	1	0.8	0.5	0.5	0.15	0.15	0.15	0.25	0.25	0.00	-0.011	0.018	0.023
1	4	1	0.8	0.5	0.5	0.15	0.15	0.15	0.25	0.25	0.25	-0.015	0.011	-0.003
1	4	1	0.8	0.5	0.5	0.15	0.15	0.15	0.25	0.25	0.50	-0.015	0.003	0.007
1	4	1	0.8	0.5	0.5	0.15	0.15	0.15	0.25	0.50	0.00	0.013	-0.003	0.004
1	4	1	0.8	0.5	0.5	0.15	0.15	0.15	0.25	0.50	0.25	0.000	-0.003	0.013
1	4	1	0.8	0.5	0.5	0.15	0.15	0.15	0.25	0.50	0.50	-0.008	-0.004	0.018
1	4	1	0.8	0.5	0.5	0.15	0.15	0.15	0.50	0.00	0.00	-0.012	0.021	-0.004
1	4	1	0.8	0.5	0.5	0.15	0.15	0.15	0.50	0.00	0.25	-0.001	0.012	0.005
1	4	1	0.8	0.5	0.5	0.15	0.15	0.15	0.50	0.00	0.50	-0.029	0.008	0.002
1	4	1	0.8	0.5	0.5	0.15	0.15	0.15	0.50	0.25	0.00	-0.017	0.013	0.003
1	4	1	0.8	0.5	0.5	0.15	0.15	0.15	0.50	0.25	0.25	-0.014	0.017	0.003
1	4	1	0.8	0.5	0.5	0.15	0.15	0.15	0.50	0.25	0.50	-0.004	0.013	0.019
1	4	1	0.8	0.5	0.5	0.15	0.15	0.15	0.50	0.50	0.00	-0.016	0.016	0.007
1	4	1	0.8	0.5	0.5	0.15	0.15	0.15	0.50	0.50	0.25	-0.005	0.000	0.004
1	4	1	0.8	0.5	0.5	0.15	0.15	0.15	0.50	0.50	0.50	-0.013	0.027	0.011
1	4	1	0.8	0.5	0.5	0.30	0.30	0.30	0.00	0.00	0.00	0.003	-0.017	0.004
1	4	1	0.8	0.5	0.5	0.30	0.30	0.30	0.00	0.00	0.25	-0.008	0.012	0.022
1	4	1	0.8	0.5	0.5	0.30	0.30	0.30	0.00	0.00	0.50	-0.043	-0.023	0.008
1	4	1	0.8	0.5	0.5	0.30	0.30	0.30	0.00	0.25	0.00	-0.004	-0.028	-0.020
1	4	1	0.8	0.5	0.5	0.30	0.30	0.30	0.00	0.25	0.25	-0.024	-0.019	0.013
1	4	1	0.8	0.5	0.5	0.30	0.30	0.30	0.00	0.25	0.50	-0.038	-0.023	0.001
1	4	1	0.8	0.5	0.5	0.30	0.30	0.30	0.00	0.50	0.00	0.030	0.000	0.004
1	4	1	0.8	0.5	0.5	0.30	0.30	0.30	0.00	0.50	0.25	-0.008	-0.024	0.010
1	4	1	0.8	0.5	0.5	0.30	0.30	0.30	0.00	0.50	0.50	-0.007	-0.017	0.014
1	4	1	0.8	0.5	0.5	0.30	0.30	0.30	0.25	0.00	0.00	-0.034	-0.003	0.002
1	4	1	0.8	0.5	0.5	0.30	0.30	0.30	0.25	0.00	0.25	-0.037	0.014	0.008
1	4	1	0.8	0.5	0.5	0.30	0.30	0.30	0.25	0.00	0.50	-0.029	-0.004	0.032
1	4	1	0.8	0.5	0.5	0.30	0.30	0.30	0.25	0.25	0.00	-0.002	-0.015	-0.009
1	4	1	0.8	0.5	0.5	0.30	0.30	0.30	0.25	0.25	0.25	0.004	-0.009	-0.009

(continued)

												Mean Bias		
N	m_1	$\frac{m_2}{m_1}$	$E(C)$	$E(R)$	$E(U)$	σ_C	σ_R	σ_U	ρ_{CR}	ρ_{CU}	ρ_{RU}	c	r	u
1	4	1	0.8	0.5	0.5	0.30	0.30	0.30	0.25	0.25	0.50	-0.006	0.005	0.013
1	4	1	0.8	0.5	0.5	0.30	0.30	0.30	0.25	0.50	0.00	0.023	-0.010	0.015
1	4	1	0.8	0.5	0.5	0.30	0.30	0.30	0.25	0.50	0.25	0.008	-0.037	-0.020
1	4	1	0.8	0.5	0.5	0.30	0.30	0.30	0.25	0.50	0.50	0.005	-0.002	0.005
1	4	1	0.8	0.5	0.5	0.30	0.30	0.30	0.50	0.00	0.00	-0.012	0.007	-0.004
1	4	1	0.8	0.5	0.5	0.30	0.30	0.30	0.50	0.00	0.25	-0.025	0.006	0.020
1	4	1	0.8	0.5	0.5	0.30	0.30	0.30	0.50	0.00	0.50	-0.023	0.009	0.005
1	4	1	0.8	0.5	0.5	0.30	0.30	0.30	0.50	0.25	0.00	-0.002	-0.002	-0.003
1	4	1	0.8	0.5	0.5	0.30	0.30	0.30	0.50	0.25	0.25	-0.005	0.004	0.001
1	4	1	0.8	0.5	0.5	0.30	0.30	0.30	0.50	0.25	0.50	-0.002	0.018	0.010
1	4	1	0.8	0.5	0.5	0.30	0.30	0.30	0.50	0.50	0.00	-0.006	-0.020	0.005
1	4	1	0.8	0.5	0.5	0.30	0.30	0.30	0.50	0.50	0.25	0.008	-0.001	0.025
1	4	1	0.8	0.5	0.5	0.30	0.30	0.30	0.50	0.50	0.50	-0.013	0.005	0.014
1	4	1	0.8	0.8	0.5	0.00	0.00	0.00	0.00	0.00	0.00	0.017	0.000	0.011
1	4	1	0.8	0.8	0.5	0.15	0.15	0.15	0.00	0.00	0.00	0.018	-0.030	0.008
1	4	1	0.8	0.8	0.5	0.15	0.15	0.15	0.00	0.00	0.25	0.021	-0.003	0.009
1	4	1	0.8	0.8	0.5	0.15	0.15	0.15	0.00	0.00	0.50	-0.002	-0.017	0.012
1	4	1	0.8	0.8	0.5	0.15	0.15	0.15	0.00	0.25	0.00	0.009	-0.028	0.004
1	4	1	0.8	0.8	0.5	0.15	0.15	0.15	0.00	0.25	0.25	0.026	-0.032	0.007
1	4	1	0.8	0.8	0.5	0.15	0.15	0.15	0.00	0.25	0.50	0.027	-0.021	0.005
1	4	1	0.8	0.8	0.5	0.15	0.15	0.15	0.00	0.50	0.00	0.015	-0.024	0.003
1	4	1	0.8	0.8	0.5	0.15	0.15	0.15	0.00	0.50	0.25	0.033	-0.018	0.035
1	4	1	0.8	0.8	0.5	0.15	0.15	0.15	0.00	0.50	0.50	0.015	-0.047	0.006
1	4	1	0.8	0.8	0.5	0.15	0.15	0.15	0.25	0.00	0.00	0.019	-0.021	0.019
1	4	1	0.8	0.8	0.5	0.15	0.15	0.15	0.25	0.00	0.25	0.020	-0.006	0.003
1	4	1	0.8	0.8	0.5	0.15	0.15	0.15	0.25	0.00	0.50	-0.003	-0.017	0.016
1	4	1	0.8	0.8	0.5	0.15	0.15	0.15	0.25	0.25	0.00	0.028	-0.007	0.021
1	4	1	0.8	0.8	0.5	0.15	0.15	0.15	0.25	0.25	0.25	0.035	-0.021	0.006
1	4	1	0.8	0.8	0.5	0.15	0.15	0.15	0.25	0.25	0.50	0.009	-0.004	0.015
1	4	1	0.8	0.8	0.5	0.15	0.15	0.15	0.25	0.50	0.00	0.033	-0.027	0.010
1	4	1	0.8	0.8	0.5	0.15	0.15	0.15	0.25	0.50	0.25	0.023	-0.024	0.009
1	4	1	0.8	0.8	0.5	0.15	0.15	0.15	0.25	0.50	0.50	0.019	-0.032	0.018
1	4	1	0.8	0.8	0.5	0.15	0.15	0.15	0.50	0.00	0.00	0.023	-0.029	0.016
1	4	1	0.8	0.8	0.5	0.15	0.15	0.15	0.50	0.00	0.25	0.019	-0.019	0.010
1	4	1	0.8	0.8	0.5	0.15	0.15	0.15	0.50	0.00	0.50	0.001	-0.020	0.022
1	4	1	0.8	0.8	0.5	0.15	0.15	0.15	0.50	0.25	0.00	0.023	-0.035	0.008
1	4	1	0.8	0.8	0.5	0.15	0.15	0.15	0.50	0.25	0.25	0.017	-0.026	0.016
1	4	1	0.8	0.8	0.5	0.15	0.15	0.15	0.50	0.25	0.50	0.036	-0.026	0.004
1	4	1	0.8	0.8	0.5	0.15	0.15	0.15	0.50	0.50	0.00	0.019	-0.002	0.007

(continued)

												Mean Bias		
N	m_1	$\frac{m_2}{m_1}$	$E(C)$	$E(R)$	$E(U)$	σ_C	σ_R	σ_U	ρ_{CR}	ρ_{CU}	ρ_{RU}	c	r	u
1	4	1	0.8	0.8	0.5	0.15	0.15	0.15	0.50	0.50	0.25	0.014	-0.019	0.014
1	4	1	0.8	0.8	0.5	0.15	0.15	0.15	0.50	0.50	0.50	0.030	-0.028	0.017
1	4	1	0.8	0.8	0.5	0.30	0.30	0.30	0.00	0.00	0.00	0.025	-0.065	0.005
1	4	1	0.8	0.8	0.5	0.30	0.30	0.30	0.00	0.00	0.25	0.026	-0.064	0.013
1	4	1	0.8	0.8	0.5	0.30	0.30	0.30	0.00	0.00	0.50	0.003	-0.063	0.027
1	4	1	0.8	0.8	0.5	0.30	0.30	0.30	0.00	0.25	0.00	0.048	-0.088	0.013
1	4	1	0.8	0.8	0.5	0.30	0.30	0.30	0.00	0.25	0.25	0.015	-0.079	-0.005
1	4	1	0.8	0.8	0.5	0.30	0.30	0.30	0.00	0.25	0.50	0.020	-0.060	0.010
1	4	1	0.8	0.8	0.5	0.30	0.30	0.30	0.00	0.50	0.00	0.043	-0.094	0.011
1	4	1	0.8	0.8	0.5	0.30	0.30	0.30	0.00	0.50	0.25	0.053	-0.061	0.012
1	4	1	0.8	0.8	0.5	0.30	0.30	0.30	0.00	0.50	0.50	0.014	-0.076	0.002
1	4	1	0.8	0.8	0.5	0.30	0.30	0.30	0.25	0.00	0.00	0.051	-0.047	0.011
1	4	1	0.8	0.8	0.5	0.30	0.30	0.30	0.25	0.00	0.25	0.022	-0.049	0.006
1	4	1	0.8	0.8	0.5	0.30	0.30	0.30	0.25	0.00	0.50	0.027	-0.046	-0.006
1	4	1	0.8	0.8	0.5	0.30	0.30	0.30	0.25	0.25	0.00	0.039	-0.076	0.024
1	4	1	0.8	0.8	0.5	0.30	0.30	0.30	0.25	0.25	0.25	0.016	-0.062	0.004
1	4	1	0.8	0.8	0.5	0.30	0.30	0.30	0.25	0.25	0.50	0.010	-0.054	0.017
1	4	1	0.8	0.8	0.5	0.30	0.30	0.30	0.25	0.50	0.00	0.055	-0.076	0.011
1	4	1	0.8	0.8	0.5	0.30	0.30	0.30	0.25	0.50	0.25	0.042	-0.084	0.011
1	4	1	0.8	0.8	0.5	0.30	0.30	0.30	0.25	0.50	0.50	0.033	-0.070	0.018
1	4	1	0.8	0.8	0.5	0.30	0.30	0.30	0.50	0.00	0.00	0.030	-0.036	0.013
1	4	1	0.8	0.8	0.5	0.30	0.30	0.30	0.50	0.00	0.25	0.007	-0.060	-0.008
1	4	1	0.8	0.8	0.5	0.30	0.30	0.30	0.50	0.00	0.50	-0.006	-0.037	0.035
1	4	1	0.8	0.8	0.5	0.30	0.30	0.30	0.50	0.25	0.00	0.034	-0.069	-0.004
1	4	1	0.8	0.8	0.5	0.30	0.30	0.30	0.50	0.25	0.25	0.007	-0.054	0.008
1	4	1	0.8	0.8	0.5	0.30	0.30	0.30	0.50	0.25	0.50	0.018	-0.046	-0.004
1	4	1	0.8	0.8	0.5	0.30	0.30	0.30	0.50	0.50	0.00	0.030	-0.051	0.025
1	4	1	0.8	0.8	0.5	0.30	0.30	0.30	0.50	0.50	0.25	0.051	-0.031	0.037
1	4	1	0.8	0.8	0.5	0.30	0.30	0.30	0.50	0.50	0.50	0.034	-0.046	0.007
1	8	0	0.2	0.2	0.5	0.00	0.00	0.00	0.00	0.00	0.00	0.001	0.001	0.038
1	8	0	0.2	0.2	0.5	0.15	0.15	0.15	0.00	0.00	0.00	-0.012	-0.008	0.005
1	8	0	0.2	0.2	0.5	0.15	0.15	0.15	0.00	0.00	0.25	0.002	-0.017	0.020
1	8	0	0.2	0.2	0.5	0.15	0.15	0.15	0.00	0.00	0.50	-0.012	-0.009	0.027
1	8	0	0.2	0.2	0.5	0.15	0.15	0.15	0.00	0.25	0.00	0.000	-0.016	0.027
1	8	0	0.2	0.2	0.5	0.15	0.15	0.15	0.00	0.25	0.25	0.000	-0.005	0.022
1	8	0	0.2	0.2	0.5	0.15	0.15	0.15	0.00	0.25	0.50	-0.002	-0.020	0.014
1	8	0	0.2	0.2	0.5	0.15	0.15	0.15	0.00	0.50	0.00	0.013	-0.031	0.033
1	8	0	0.2	0.2	0.5	0.15	0.15	0.15	0.00	0.50	0.25	0.001	-0.029	0.027
1	8	0	0.2	0.2	0.5	0.15	0.15	0.15	0.00	0.50	0.50	0.013	-0.027	0.037

(continued)

N	m_1	$\frac{m_2}{m_1}$	$E(C)$	$E(R)$	$E(U)$	σ_C	σ_R	σ_U	ρ_{CR}	ρ_{CU}	ρ_{RU}	Mean Bias c	Mean Bias r	Mean Bias u
1	8	0	0.2	0.2	0.5	0.15	0.15	0.15	0.25	0.00	0.00	-0.010	0.007	0.024
1	8	0	0.2	0.2	0.5	0.15	0.15	0.15	0.25	0.00	0.25	-0.003	0.001	0.011
1	8	0	0.2	0.2	0.5	0.15	0.15	0.15	0.25	0.00	0.50	0.002	0.000	0.036
1	8	0	0.2	0.2	0.5	0.15	0.15	0.15	0.25	0.25	0.00	-0.010	-0.011	0.012
1	8	0	0.2	0.2	0.5	0.15	0.15	0.15	0.25	0.25	0.25	-0.001	0.021	0.016
1	8	0	0.2	0.2	0.5	0.15	0.15	0.15	0.25	0.25	0.50	-0.007	0.000	0.017
1	8	0	0.2	0.2	0.5	0.15	0.15	0.15	0.25	0.50	0.00	0.001	-0.017	0.020
1	8	0	0.2	0.2	0.5	0.15	0.15	0.15	0.25	0.50	0.25	0.005	-0.017	0.026
1	8	0	0.2	0.2	0.5	0.15	0.15	0.15	0.25	0.50	0.50	0.001	-0.008	0.033
1	8	0	0.2	0.2	0.5	0.15	0.15	0.15	0.50	0.00	0.00	-0.004	0.011	0.026
1	8	0	0.2	0.2	0.5	0.15	0.15	0.15	0.50	0.00	0.25	-0.004	0.023	0.022
1	8	0	0.2	0.2	0.5	0.15	0.15	0.15	0.50	0.00	0.50	-0.007	0.013	0.021
1	8	0	0.2	0.2	0.5	0.15	0.15	0.15	0.50	0.25	0.00	-0.013	-0.024	0.019
1	8	0	0.2	0.2	0.5	0.15	0.15	0.15	0.50	0.25	0.25	0.000	0.026	0.045
1	8	0	0.2	0.2	0.5	0.15	0.15	0.15	0.50	0.25	0.50	0.013	0.018	0.034
1	8	0	0.2	0.2	0.5	0.15	0.15	0.15	0.50	0.50	0.00	0.011	0.016	0.029
1	8	0	0.2	0.2	0.5	0.15	0.15	0.15	0.50	0.50	0.25	0.007	0.007	0.025
1	8	0	0.2	0.2	0.5	0.15	0.15	0.15	0.50	0.50	0.50	0.010	0.004	0.027
1	8	0	0.2	0.2	0.5	0.30	0.30	0.30	0.00	0.00	0.00	0.020	-0.043	0.005
1	8	0	0.2	0.2	0.5	0.30	0.30	0.30	0.00	0.00	0.25	0.023	-0.049	0.015
1	8	0	0.2	0.2	0.5	0.30	0.30	0.30	0.00	0.00	0.50	0.022	-0.061	0.008
1	8	0	0.2	0.2	0.5	0.30	0.30	0.30	0.00	0.25	0.00	0.033	-0.065	-0.006
1	8	0	0.2	0.2	0.5	0.30	0.30	0.30	0.00	0.25	0.25	0.054	-0.046	-0.008
1	8	0	0.2	0.2	0.5	0.30	0.30	0.30	0.00	0.25	0.50	0.036	-0.061	-0.008
1	8	0	0.2	0.2	0.5	0.30	0.30	0.30	0.00	0.50	0.00	0.046	-0.075	-0.016
1	8	0	0.2	0.2	0.5	0.30	0.30	0.30	0.00	0.50	0.25	0.047	-0.071	-0.020
1	8	0	0.2	0.2	0.5	0.30	0.30	0.30	0.00	0.50	0.50	0.049	-0.062	-0.009
1	8	0	0.2	0.2	0.5	0.30	0.30	0.30	0.25	0.00	0.00	0.056	0.001	0.003
1	8	0	0.2	0.2	0.5	0.30	0.30	0.30	0.25	0.00	0.25	0.039	-0.017	0.012
1	8	0	0.2	0.2	0.5	0.30	0.30	0.30	0.25	0.00	0.50	0.015	-0.023	-0.007
1	8	0	0.2	0.2	0.5	0.30	0.30	0.30	0.25	0.25	0.00	0.048	-0.039	-0.008
1	8	0	0.2	0.2	0.5	0.30	0.30	0.30	0.25	0.25	0.25	0.032	-0.033	-0.002
1	8	0	0.2	0.2	0.5	0.30	0.30	0.30	0.25	0.25	0.50	0.020	-0.044	-0.013
1	8	0	0.2	0.2	0.5	0.30	0.30	0.30	0.25	0.50	0.00	0.059	-0.040	-0.017
1	8	0	0.2	0.2	0.5	0.30	0.30	0.30	0.25	0.50	0.25	0.037	-0.032	-0.016
1	8	0	0.2	0.2	0.5	0.30	0.30	0.30	0.25	0.50	0.50	0.046	-0.042	-0.010
1	8	0	0.2	0.2	0.5	0.30	0.30	0.30	0.50	0.00	0.00	0.027	0.005	0.010
1	8	0	0.2	0.2	0.5	0.30	0.30	0.30	0.50	0.00	0.25	0.033	0.017	0.011
1	8	0	0.2	0.2	0.5	0.30	0.30	0.30	0.50	0.00	0.50	0.018	0.007	0.030

(continued)

												Mean Bias		
N	m_1	$\frac{m_2}{m_1}$	$E(C)$	$E(R)$	$E(U)$	σ_C	σ_R	σ_U	ρ_{CR}	ρ_{CU}	ρ_{RU}	c	r	u
1	8	0	0.2	0.2	0.5	0.30	0.30	0.30	0.50	0.25	0.00	0.050	-0.015	0.009
1	8	0	0.2	0.2	0.5	0.30	0.30	0.30	0.50	0.25	0.25	0.047	-0.005	-0.008
1	8	0	0.2	0.2	0.5	0.30	0.30	0.30	0.50	0.25	0.50	0.056	-0.015	-0.010
1	8	0	0.2	0.2	0.5	0.30	0.30	0.30	0.50	0.50	0.00	0.051	-0.025	0.002
1	8	0	0.2	0.2	0.5	0.30	0.30	0.30	0.50	0.50	0.25	0.038	-0.021	0.012
1	8	0	0.2	0.2	0.5	0.30	0.30	0.30	0.50	0.50	0.50	0.064	-0.031	-0.017
1	8	0	0.2	0.5	0.5	0.00	0.00	0.00	0.00	0.00	0.00	0.027	-0.072	0.033
1	8	0	0.2	0.5	0.5	0.15	0.15	0.15	0.00	0.00	0.00	0.028	-0.129	0.033
1	8	0	0.2	0.5	0.5	0.15	0.15	0.15	0.00	0.00	0.25	0.012	-0.137	0.043
1	8	0	0.2	0.5	0.5	0.15	0.15	0.15	0.00	0.00	0.50	0.021	-0.111	0.049
1	8	0	0.2	0.5	0.5	0.15	0.15	0.15	0.00	0.25	0.00	0.021	-0.134	0.043
1	8	0	0.2	0.5	0.5	0.15	0.15	0.15	0.00	0.25	0.25	0.008	-0.129	0.023
1	8	0	0.2	0.5	0.5	0.15	0.15	0.15	0.00	0.25	0.50	0.026	-0.125	0.037
1	8	0	0.2	0.5	0.5	0.15	0.15	0.15	0.00	0.50	0.00	0.029	-0.133	0.035
1	8	0	0.2	0.5	0.5	0.15	0.15	0.15	0.00	0.50	0.25	0.022	-0.132	0.040
1	8	0	0.2	0.5	0.5	0.15	0.15	0.15	0.00	0.50	0.50	0.022	-0.147	0.030
1	8	0	0.2	0.5	0.5	0.15	0.15	0.15	0.25	0.00	0.00	0.021	-0.102	0.041
1	8	0	0.2	0.5	0.5	0.15	0.15	0.15	0.25	0.00	0.25	0.022	-0.118	0.025
1	8	0	0.2	0.5	0.5	0.15	0.15	0.15	0.25	0.00	0.50	0.036	-0.104	0.043
1	8	0	0.2	0.5	0.5	0.15	0.15	0.15	0.25	0.25	0.00	0.012	-0.120	0.029
1	8	0	0.2	0.5	0.5	0.15	0.15	0.15	0.25	0.25	0.25	0.024	-0.107	0.035
1	8	0	0.2	0.5	0.5	0.15	0.15	0.15	0.25	0.25	0.50	0.030	-0.121	0.027
1	8	0	0.2	0.5	0.5	0.15	0.15	0.15	0.25	0.50	0.00	0.033	-0.129	0.039
1	8	0	0.2	0.5	0.5	0.15	0.15	0.15	0.25	0.50	0.25	0.024	-0.138	0.034
1	8	0	0.2	0.5	0.5	0.15	0.15	0.15	0.25	0.50	0.50	0.023	-0.129	0.026
1	8	0	0.2	0.5	0.5	0.15	0.15	0.15	0.50	0.00	0.00	0.029	-0.097	0.036
1	8	0	0.2	0.5	0.5	0.15	0.15	0.15	0.50	0.00	0.25	0.023	-0.117	0.042
1	8	0	0.2	0.5	0.5	0.15	0.15	0.15	0.50	0.00	0.50	0.029	-0.098	0.035
1	8	0	0.2	0.5	0.5	0.15	0.15	0.15	0.50	0.25	0.00	0.045	-0.107	0.041
1	8	0	0.2	0.5	0.5	0.15	0.15	0.15	0.50	0.25	0.25	0.027	-0.121	0.035
1	8	0	0.2	0.5	0.5	0.15	0.15	0.15	0.50	0.25	0.50	0.023	-0.131	0.036
1	8	0	0.2	0.5	0.5	0.15	0.15	0.15	0.50	0.50	0.00	0.030	-0.112	0.024
1	8	0	0.2	0.5	0.5	0.15	0.15	0.15	0.50	0.50	0.25	0.038	-0.114	0.038
1	8	0	0.2	0.5	0.5	0.15	0.15	0.15	0.50	0.50	0.50	0.034	-0.134	0.030
1	8	0	0.2	0.5	0.5	0.30	0.30	0.30	0.00	0.00	0.00	0.049	-0.220	0.033
1	8	0	0.2	0.5	0.5	0.30	0.30	0.30	0.00	0.00	0.25	0.046	-0.242	0.002
1	8	0	0.2	0.5	0.5	0.30	0.30	0.30	0.00	0.00	0.50	0.055	-0.217	0.011
1	8	0	0.2	0.5	0.5	0.30	0.30	0.30	0.00	0.25	0.00	0.063	-0.230	0.014
1	8	0	0.2	0.5	0.5	0.30	0.30	0.30	0.00	0.25	0.25	0.056	-0.223	0.025

(continued)

N	m_1	$\frac{m_2}{m_1}$	$E(C)$	$E(R)$	$E(U)$	σ_C	σ_R	σ_U	ρ_{CR}	ρ_{CU}	ρ_{RU}	Mean Bias c	Mean Bias r	Mean Bias u
1	8	0	0.2	0.5	0.5	0.30	0.30	0.30	0.00	0.25	0.50	0.053	-0.208	0.008
1	8	0	0.2	0.5	0.5	0.30	0.30	0.30	0.00	0.50	0.00	0.079	-0.244	0.004
1	8	0	0.2	0.5	0.5	0.30	0.30	0.30	0.00	0.50	0.25	0.081	-0.242	-0.003
1	8	0	0.2	0.5	0.5	0.30	0.30	0.30	0.00	0.50	0.50	0.057	-0.245	-0.002
1	8	0	0.2	0.5	0.5	0.30	0.30	0.30	0.25	0.00	0.00	0.073	-0.202	0.030
1	8	0	0.2	0.5	0.5	0.30	0.30	0.30	0.25	0.00	0.25	0.068	-0.201	0.026
1	8	0	0.2	0.5	0.5	0.30	0.30	0.30	0.25	0.00	0.50	0.045	-0.219	0.033
1	8	0	0.2	0.5	0.5	0.30	0.30	0.30	0.25	0.25	0.00	0.072	-0.230	0.003
1	8	0	0.2	0.5	0.5	0.30	0.30	0.30	0.25	0.25	0.25	0.068	-0.214	0.021
1	8	0	0.2	0.5	0.5	0.30	0.30	0.30	0.25	0.25	0.50	0.055	-0.225	0.013
1	8	0	0.2	0.5	0.5	0.30	0.30	0.30	0.25	0.50	0.00	0.048	-0.246	-0.016
1	8	0	0.2	0.5	0.5	0.30	0.30	0.30	0.25	0.50	0.25	0.059	-0.220	0.011
1	8	0	0.2	0.5	0.5	0.30	0.30	0.30	0.25	0.50	0.50	0.075	-0.210	0.008
1	8	0	0.2	0.5	0.5	0.30	0.30	0.30	0.50	0.00	0.00	0.072	-0.187	0.034
1	8	0	0.2	0.5	0.5	0.30	0.30	0.30	0.50	0.00	0.25	0.056	-0.164	0.018
1	8	0	0.2	0.5	0.5	0.30	0.30	0.30	0.50	0.00	0.50	0.069	-0.168	0.028
1	8	0	0.2	0.5	0.5	0.30	0.30	0.30	0.50	0.25	0.00	0.080	-0.189	0.014
1	8	0	0.2	0.5	0.5	0.30	0.30	0.30	0.50	0.25	0.25	0.056	-0.200	0.017
1	8	0	0.2	0.5	0.5	0.30	0.30	0.30	0.50	0.25	0.50	0.064	-0.183	0.033
1	8	0	0.2	0.5	0.5	0.30	0.30	0.30	0.50	0.50	0.00	0.074	-0.192	0.024
1	8	0	0.2	0.5	0.5	0.30	0.30	0.30	0.50	0.50	0.25	0.088	-0.191	0.011
1	8	0	0.2	0.5	0.5	0.30	0.30	0.30	0.50	0.50	0.50	0.080	-0.175	0.017
1	8	0	0.2	0.8	0.5	0.00	0.00	0.00	0.00	0.00	0.00	0.043	-0.219	0.055
1	8	0	0.2	0.8	0.5	0.15	0.15	0.15	0.00	0.00	0.00	0.054	-0.268	0.042
1	8	0	0.2	0.8	0.5	0.15	0.15	0.15	0.00	0.00	0.25	0.058	-0.269	0.053
1	8	0	0.2	0.8	0.5	0.15	0.15	0.15	0.00	0.00	0.50	0.032	-0.288	0.045
1	8	0	0.2	0.8	0.5	0.15	0.15	0.15	0.00	0.25	0.00	0.056	-0.252	0.055
1	8	0	0.2	0.8	0.5	0.15	0.15	0.15	0.00	0.25	0.25	0.043	-0.284	0.045
1	8	0	0.2	0.8	0.5	0.15	0.15	0.15	0.00	0.25	0.50	0.043	-0.268	0.050
1	8	0	0.2	0.8	0.5	0.15	0.15	0.15	0.00	0.50	0.00	0.063	-0.286	0.051
1	8	0	0.2	0.8	0.5	0.15	0.15	0.15	0.00	0.50	0.25	0.049	-0.325	0.060
1	8	0	0.2	0.8	0.5	0.15	0.15	0.15	0.00	0.50	0.50	0.058	-0.273	0.052
1	8	0	0.2	0.8	0.5	0.15	0.15	0.15	0.25	0.00	0.00	0.042	-0.285	0.049
1	8	0	0.2	0.8	0.5	0.15	0.15	0.15	0.25	0.00	0.25	0.047	-0.292	0.062
1	8	0	0.2	0.8	0.5	0.15	0.15	0.15	0.25	0.00	0.50	0.042	-0.280	0.045
1	8	0	0.2	0.8	0.5	0.15	0.15	0.15	0.25	0.25	0.00	0.054	-0.271	0.049
1	8	0	0.2	0.8	0.5	0.15	0.15	0.15	0.25	0.25	0.25	0.059	-0.266	0.044
1	8	0	0.2	0.8	0.5	0.15	0.15	0.15	0.25	0.25	0.50	0.054	-0.287	0.044
1	8	0	0.2	0.8	0.5	0.15	0.15	0.15	0.25	0.50	0.00	0.058	-0.279	0.043

(continued)

N	m_1	$\frac{m_2}{m_1}$	$E(C)$	$E(R)$	$E(U)$	σ_C	σ_R	σ_U	ρ_{CR}	ρ_{CU}	ρ_{RU}	Mean Bias c	Mean Bias r	Mean Bias u
1	8	0	0.2	0.8	0.5	0.15	0.15	0.15	0.25	0.50	0.25	0.052	-0.280	0.045
1	8	0	0.2	0.8	0.5	0.15	0.15	0.15	0.25	0.50	0.50	0.048	-0.285	0.058
1	8	0	0.2	0.8	0.5	0.15	0.15	0.15	0.50	0.00	0.00	0.058	-0.280	0.067
1	8	0	0.2	0.8	0.5	0.15	0.15	0.15	0.50	0.00	0.25	0.046	-0.262	0.050
1	8	0	0.2	0.8	0.5	0.15	0.15	0.15	0.50	0.00	0.50	0.050	-0.248	0.048
1	8	0	0.2	0.8	0.5	0.15	0.15	0.15	0.50	0.25	0.00	0.058	-0.266	0.046
1	8	0	0.2	0.8	0.5	0.15	0.15	0.15	0.50	0.25	0.25	0.060	-0.270	0.043
1	8	0	0.2	0.8	0.5	0.15	0.15	0.15	0.50	0.25	0.50	0.052	-0.268	0.051
1	8	0	0.2	0.8	0.5	0.15	0.15	0.15	0.50	0.50	0.00	0.059	-0.282	0.056
1	8	0	0.2	0.8	0.5	0.15	0.15	0.15	0.50	0.50	0.25	0.071	-0.263	0.060
1	8	0	0.2	0.8	0.5	0.15	0.15	0.15	0.50	0.50	0.50	0.067	-0.252	0.064
1	8	0	0.2	0.8	0.5	0.30	0.30	0.30	0.00	0.00	0.00	0.072	-0.443	0.036
1	8	0	0.2	0.8	0.5	0.30	0.30	0.30	0.00	0.00	0.25	0.080	-0.412	0.037
1	8	0	0.2	0.8	0.5	0.30	0.30	0.30	0.00	0.00	0.50	0.071	-0.429	0.046
1	8	0	0.2	0.8	0.5	0.30	0.30	0.30	0.00	0.25	0.00	0.078	-0.437	0.022
1	8	0	0.2	0.8	0.5	0.30	0.30	0.30	0.00	0.25	0.25	0.085	-0.431	0.018
1	8	0	0.2	0.8	0.5	0.30	0.30	0.30	0.00	0.25	0.50	0.070	-0.424	0.046
1	8	0	0.2	0.8	0.5	0.30	0.30	0.30	0.00	0.50	0.00	0.080	-0.474	0.006
1	8	0	0.2	0.8	0.5	0.30	0.30	0.30	0.00	0.50	0.25	0.082	-0.441	0.028
1	8	0	0.2	0.8	0.5	0.30	0.30	0.30	0.00	0.50	0.50	0.081	-0.459	0.010
1	8	0	0.2	0.8	0.5	0.30	0.30	0.30	0.25	0.00	0.00	0.092	-0.429	0.033
1	8	0	0.2	0.8	0.5	0.30	0.30	0.30	0.25	0.00	0.25	0.088	-0.404	0.039
1	8	0	0.2	0.8	0.5	0.30	0.30	0.30	0.25	0.00	0.50	0.068	-0.406	0.043
1	8	0	0.2	0.8	0.5	0.30	0.30	0.30	0.25	0.25	0.00	0.078	-0.449	0.050
1	8	0	0.2	0.8	0.5	0.30	0.30	0.30	0.25	0.25	0.25	0.088	-0.381	0.044
1	8	0	0.2	0.8	0.5	0.30	0.30	0.30	0.25	0.25	0.50	0.082	-0.414	0.030
1	8	0	0.2	0.8	0.5	0.30	0.30	0.30	0.25	0.50	0.00	0.085	-0.453	0.006
1	8	0	0.2	0.8	0.5	0.30	0.30	0.30	0.25	0.50	0.25	0.103	-0.437	0.004
1	8	0	0.2	0.8	0.5	0.30	0.30	0.30	0.25	0.50	0.50	0.091	-0.406	0.038
1	8	0	0.2	0.8	0.5	0.30	0.30	0.30	0.50	0.00	0.00	0.103	-0.401	0.026
1	8	0	0.2	0.8	0.5	0.30	0.30	0.30	0.50	0.00	0.25	0.087	-0.393	0.053
1	8	0	0.2	0.8	0.5	0.30	0.30	0.30	0.50	0.00	0.50	0.095	-0.379	0.053
1	8	0	0.2	0.8	0.5	0.30	0.30	0.30	0.50	0.25	0.00	0.082	-0.381	0.035
1	8	0	0.2	0.8	0.5	0.30	0.30	0.30	0.50	0.25	0.25	0.096	-0.417	0.042
1	8	0	0.2	0.8	0.5	0.30	0.30	0.30	0.50	0.25	0.50	0.096	-0.414	0.007
1	8	0	0.2	0.8	0.5	0.30	0.30	0.30	0.50	0.50	0.00	0.093	-0.416	0.012
1	8	0	0.2	0.8	0.5	0.30	0.30	0.30	0.50	0.50	0.25	0.104	-0.412	0.024
1	8	0	0.2	0.8	0.5	0.30	0.30	0.30	0.50	0.50	0.50	0.076	-0.431	0.013
1	8	0	0.5	0.2	0.5	0.00	0.00	0.00	0.00	0.00	0.00	-0.141	0.167	-0.020

(continued)

N	m_1	$\frac{m_2}{m_1}$	$E(C)$	$E(R)$	$E(U)$	σ_C	σ_R	σ_U	ρ_{CR}	ρ_{CU}	ρ_{RU}	Mean Bias c	Mean Bias r	Mean Bias u
1	8	0	0.5	0.2	0.5	0.15	0.15	0.15	0.00	0.00	0.00	-0.133	0.105	-0.027
1	8	0	0.5	0.2	0.5	0.15	0.15	0.15	0.00	0.00	0.25	-0.131	0.098	-0.045
1	8	0	0.5	0.2	0.5	0.15	0.15	0.15	0.00	0.00	0.50	-0.135	0.098	-0.016
1	8	0	0.5	0.2	0.5	0.15	0.15	0.15	0.00	0.25	0.00	-0.144	0.119	-0.027
1	8	0	0.5	0.2	0.5	0.15	0.15	0.15	0.00	0.25	0.25	-0.124	0.108	-0.025
1	8	0	0.5	0.2	0.5	0.15	0.15	0.15	0.00	0.25	0.50	-0.134	0.100	-0.032
1	8	0	0.5	0.2	0.5	0.15	0.15	0.15	0.00	0.50	0.00	-0.128	0.114	-0.015
1	8	0	0.5	0.2	0.5	0.15	0.15	0.15	0.00	0.50	0.25	-0.130	0.111	-0.037
1	8	0	0.5	0.2	0.5	0.15	0.15	0.15	0.00	0.50	0.50	-0.128	0.087	-0.001
1	8	0	0.5	0.2	0.5	0.15	0.15	0.15	0.25	0.00	0.00	-0.143	0.126	-0.035
1	8	0	0.5	0.2	0.5	0.15	0.15	0.15	0.25	0.00	0.25	-0.131	0.128	-0.030
1	8	0	0.5	0.2	0.5	0.15	0.15	0.15	0.25	0.00	0.50	-0.138	0.129	-0.040
1	8	0	0.5	0.2	0.5	0.15	0.15	0.15	0.25	0.25	0.00	-0.136	0.123	-0.026
1	8	0	0.5	0.2	0.5	0.15	0.15	0.15	0.25	0.25	0.25	-0.126	0.119	-0.021
1	8	0	0.5	0.2	0.5	0.15	0.15	0.15	0.25	0.25	0.50	-0.143	0.118	-0.049
1	8	0	0.5	0.2	0.5	0.15	0.15	0.15	0.25	0.50	0.00	-0.124	0.109	-0.028
1	8	0	0.5	0.2	0.5	0.15	0.15	0.15	0.25	0.50	0.25	-0.116	0.083	-0.015
1	8	0	0.5	0.2	0.5	0.15	0.15	0.15	0.25	0.50	0.50	-0.138	0.104	-0.028
1	8	0	0.5	0.2	0.5	0.15	0.15	0.15	0.50	0.00	0.00	-0.114	0.106	-0.029
1	8	0	0.5	0.2	0.5	0.15	0.15	0.15	0.50	0.00	0.25	-0.139	0.097	-0.022
1	8	0	0.5	0.2	0.5	0.15	0.15	0.15	0.50	0.00	0.50	-0.155	0.105	-0.030
1	8	0	0.5	0.2	0.5	0.15	0.15	0.15	0.50	0.25	0.00	-0.137	0.111	-0.039
1	8	0	0.5	0.2	0.5	0.15	0.15	0.15	0.50	0.25	0.25	-0.108	0.115	-0.008
1	8	0	0.5	0.2	0.5	0.15	0.15	0.15	0.50	0.25	0.50	-0.129	0.130	-0.021
1	8	0	0.5	0.2	0.5	0.15	0.15	0.15	0.50	0.50	0.00	-0.120	0.105	-0.020
1	8	0	0.5	0.2	0.5	0.15	0.15	0.15	0.50	0.50	0.25	-0.127	0.114	-0.025
1	8	0	0.5	0.2	0.5	0.15	0.15	0.15	0.50	0.50	0.50	-0.118	0.109	-0.014
1	8	0	0.5	0.2	0.5	0.30	0.30	0.30	0.00	0.00	0.00	-0.107	0.021	-0.041
1	8	0	0.5	0.2	0.5	0.30	0.30	0.30	0.00	0.00	0.25	-0.090	0.023	-0.056
1	8	0	0.5	0.2	0.5	0.30	0.30	0.30	0.00	0.00	0.50	-0.096	0.038	-0.061
1	8	0	0.5	0.2	0.5	0.30	0.30	0.30	0.00	0.25	0.00	-0.078	0.005	-0.065
1	8	0	0.5	0.2	0.5	0.30	0.30	0.30	0.00	0.25	0.25	-0.080	0.025	-0.052
1	8	0	0.5	0.2	0.5	0.30	0.30	0.30	0.00	0.25	0.50	-0.095	0.011	-0.040
1	8	0	0.5	0.2	0.5	0.30	0.30	0.30	0.00	0.50	0.00	-0.090	0.012	-0.085
1	8	0	0.5	0.2	0.5	0.30	0.30	0.30	0.00	0.50	0.25	-0.089	0.000	-0.070
1	8	0	0.5	0.2	0.5	0.30	0.30	0.30	0.00	0.50	0.50	-0.080	0.025	-0.081
1	8	0	0.5	0.2	0.5	0.30	0.30	0.30	0.25	0.00	0.00	-0.086	0.035	-0.032
1	8	0	0.5	0.2	0.5	0.30	0.30	0.30	0.25	0.00	0.25	-0.085	0.043	-0.031
1	8	0	0.5	0.2	0.5	0.30	0.30	0.30	0.25	0.00	0.50	-0.095	0.046	-0.043

(continued)

N	m_1	$\frac{m_2}{m_1}$	$E(C)$	$E(R)$	$E(U)$	σ_C	σ_R	σ_U	ρ_{CR}	ρ_{CU}	ρ_{RU}	Mean Bias c	Mean Bias r	Mean Bias u
1	8	0	0.5	0.2	0.5	0.30	0.30	0.30	0.25	0.25	0.00	-0.067	0.042	-0.030
1	8	0	0.5	0.2	0.5	0.30	0.30	0.30	0.25	0.25	0.25	-0.094	0.035	-0.066
1	8	0	0.5	0.2	0.5	0.30	0.30	0.30	0.25	0.25	0.50	-0.097	0.048	-0.067
1	8	0	0.5	0.2	0.5	0.30	0.30	0.30	0.25	0.50	0.00	-0.041	0.030	-0.041
1	8	0	0.5	0.2	0.5	0.30	0.30	0.30	0.25	0.50	0.25	-0.067	0.017	-0.066
1	8	0	0.5	0.2	0.5	0.30	0.30	0.30	0.25	0.50	0.50	-0.104	0.035	-0.076
1	8	0	0.5	0.2	0.5	0.30	0.30	0.30	0.50	0.00	0.00	-0.091	0.060	-0.043
1	8	0	0.5	0.2	0.5	0.30	0.30	0.30	0.50	0.00	0.25	-0.091	0.049	-0.043
1	8	0	0.5	0.2	0.5	0.30	0.30	0.30	0.50	0.00	0.50	-0.076	0.056	-0.036
1	8	0	0.5	0.2	0.5	0.30	0.30	0.30	0.50	0.25	0.00	-0.059	0.042	-0.056
1	8	0	0.5	0.2	0.5	0.30	0.30	0.30	0.50	0.25	0.25	-0.060	0.038	-0.059
1	8	0	0.5	0.2	0.5	0.30	0.30	0.30	0.50	0.25	0.50	-0.077	0.028	-0.054
1	8	0	0.5	0.2	0.5	0.30	0.30	0.30	0.50	0.50	0.00	-0.060	0.033	-0.073
1	8	0	0.5	0.2	0.5	0.30	0.30	0.30	0.50	0.50	0.25	-0.071	0.042	-0.070
1	8	0	0.5	0.2	0.5	0.30	0.30	0.30	0.50	0.50	0.50	-0.077	0.032	-0.070
1	8	0	0.5	0.5	0.5	0.00	0.00	0.00	0.00	0.00	0.00	-0.065	0.128	-0.006
1	8	0	0.5	0.5	0.5	0.15	0.15	0.15	0.00	0.00	0.00	-0.064	0.110	-0.004
1	8	0	0.5	0.5	0.5	0.15	0.15	0.15	0.00	0.00	0.25	-0.067	0.090	0.006
1	8	0	0.5	0.5	0.5	0.15	0.15	0.15	0.00	0.00	0.50	-0.062	0.113	0.003
1	8	0	0.5	0.5	0.5	0.15	0.15	0.15	0.00	0.25	0.00	-0.073	0.115	0.000
1	8	0	0.5	0.5	0.5	0.15	0.15	0.15	0.00	0.25	0.25	-0.067	0.115	-0.002
1	8	0	0.5	0.5	0.5	0.15	0.15	0.15	0.00	0.25	0.50	-0.067	0.124	0.000
1	8	0	0.5	0.5	0.5	0.15	0.15	0.15	0.00	0.50	0.00	-0.067	0.121	-0.009
1	8	0	0.5	0.5	0.5	0.15	0.15	0.15	0.00	0.50	0.25	-0.063	0.106	-0.013
1	8	0	0.5	0.5	0.5	0.15	0.15	0.15	0.00	0.50	0.50	-0.073	0.112	0.000
1	8	0	0.5	0.5	0.5	0.15	0.15	0.15	0.25	0.00	0.00	-0.065	0.087	0.017
1	8	0	0.5	0.5	0.5	0.15	0.15	0.15	0.25	0.00	0.25	-0.082	0.108	0.000
1	8	0	0.5	0.5	0.5	0.15	0.15	0.15	0.25	0.00	0.50	-0.066	0.104	-0.007
1	8	0	0.5	0.5	0.5	0.15	0.15	0.15	0.25	0.25	0.00	-0.051	0.094	0.002
1	8	0	0.5	0.5	0.5	0.15	0.15	0.15	0.25	0.25	0.25	-0.055	0.113	0.002
1	8	0	0.5	0.5	0.5	0.15	0.15	0.15	0.25	0.25	0.50	-0.067	0.126	-0.002
1	8	0	0.5	0.5	0.5	0.15	0.15	0.15	0.25	0.50	0.00	-0.067	0.093	-0.013
1	8	0	0.5	0.5	0.5	0.15	0.15	0.15	0.25	0.50	0.25	-0.078	0.129	-0.021
1	8	0	0.5	0.5	0.5	0.15	0.15	0.15	0.25	0.50	0.50	-0.062	0.088	-0.005
1	8	0	0.5	0.5	0.5	0.15	0.15	0.15	0.50	0.00	0.00	-0.071	0.093	0.007
1	8	0	0.5	0.5	0.5	0.15	0.15	0.15	0.50	0.00	0.25	-0.068	0.100	0.006
1	8	0	0.5	0.5	0.5	0.15	0.15	0.15	0.50	0.00	0.50	-0.080	0.110	-0.011
1	8	0	0.5	0.5	0.5	0.15	0.15	0.15	0.50	0.25	0.00	-0.058	0.107	0.001
1	8	0	0.5	0.5	0.5	0.15	0.15	0.15	0.50	0.25	0.25	-0.054	0.120	-0.010

(continued)

												Mean Bias		
N	m_1	$\frac{m_2}{m_1}$	$E(C)$	$E(R)$	$E(U)$	σ_C	σ_R	σ_U	ρ_{CR}	ρ_{CU}	ρ_{RU}	c	r	u
1	8	0	0.5	0.5	0.5	0.15	0.15	0.15	0.50	0.25	0.50	-0.060	0.118	-0.015
1	8	0	0.5	0.5	0.5	0.15	0.15	0.15	0.50	0.50	0.00	-0.054	0.116	-0.019
1	8	0	0.5	0.5	0.5	0.15	0.15	0.15	0.50	0.50	0.25	-0.057	0.114	0.001
1	8	0	0.5	0.5	0.5	0.15	0.15	0.15	0.50	0.50	0.50	-0.064	0.128	-0.006
1	8	0	0.5	0.5	0.5	0.30	0.30	0.30	0.00	0.00	0.00	-0.038	-0.016	-0.024
1	8	0	0.5	0.5	0.5	0.30	0.30	0.30	0.00	0.00	0.25	-0.028	-0.010	-0.021
1	8	0	0.5	0.5	0.5	0.30	0.30	0.30	0.00	0.00	0.50	-0.037	0.010	-0.041
1	8	0	0.5	0.5	0.5	0.30	0.30	0.30	0.00	0.25	0.00	-0.023	-0.020	-0.022
1	8	0	0.5	0.5	0.5	0.30	0.30	0.30	0.00	0.25	0.25	-0.022	-0.025	-0.029
1	8	0	0.5	0.5	0.5	0.30	0.30	0.30	0.00	0.25	0.50	-0.022	0.001	-0.032
1	8	0	0.5	0.5	0.5	0.30	0.30	0.30	0.00	0.50	0.00	0.001	-0.016	-0.036
1	8	0	0.5	0.5	0.5	0.30	0.30	0.30	0.00	0.50	0.25	-0.010	0.005	-0.050
1	8	0	0.5	0.5	0.5	0.30	0.30	0.30	0.00	0.50	0.50	-0.003	0.016	-0.047
1	8	0	0.5	0.5	0.5	0.30	0.30	0.30	0.25	0.00	0.00	-0.034	-0.012	0.005
1	8	0	0.5	0.5	0.5	0.30	0.30	0.30	0.25	0.00	0.25	-0.030	0.014	-0.022
1	8	0	0.5	0.5	0.5	0.30	0.30	0.30	0.25	0.00	0.50	-0.022	0.019	-0.009
1	8	0	0.5	0.5	0.5	0.30	0.30	0.30	0.25	0.25	0.00	-0.019	-0.013	-0.008
1	8	0	0.5	0.5	0.5	0.30	0.30	0.30	0.25	0.25	0.25	-0.023	-0.017	-0.037
1	8	0	0.5	0.5	0.5	0.30	0.30	0.30	0.25	0.25	0.50	-0.023	0.001	-0.034
1	8	0	0.5	0.5	0.5	0.30	0.30	0.30	0.25	0.50	0.00	0.003	0.014	-0.031
1	8	0	0.5	0.5	0.5	0.30	0.30	0.30	0.25	0.50	0.25	-0.018	0.004	-0.042
1	8	0	0.5	0.5	0.5	0.30	0.30	0.30	0.25	0.50	0.50	-0.009	0.008	-0.038
1	8	0	0.5	0.5	0.5	0.30	0.30	0.30	0.50	0.00	0.00	-0.004	0.030	0.004
1	8	0	0.5	0.5	0.5	0.30	0.30	0.30	0.50	0.00	0.25	0.005	0.028	-0.007
1	8	0	0.5	0.5	0.5	0.30	0.30	0.30	0.50	0.00	0.50	-0.007	0.038	-0.014
1	8	0	0.5	0.5	0.5	0.30	0.30	0.30	0.50	0.25	0.00	-0.014	-0.009	-0.013
1	8	0	0.5	0.5	0.5	0.30	0.30	0.30	0.50	0.25	0.25	0.001	0.010	-0.008
1	8	0	0.5	0.5	0.5	0.30	0.30	0.30	0.50	0.25	0.50	-0.019	0.019	-0.018
1	8	0	0.5	0.5	0.5	0.30	0.30	0.30	0.50	0.50	0.00	0.012	0.010	-0.031
1	8	0	0.5	0.5	0.5	0.30	0.30	0.30	0.50	0.50	0.25	-0.002	0.015	-0.025
1	8	0	0.5	0.5	0.5	0.30	0.30	0.30	0.50	0.50	0.50	-0.004	0.034	-0.038
1	8	0	0.5	0.8	0.5	0.00	0.00	0.00	0.00	0.00	0.00	0.001	0.032	0.027
1	8	0	0.5	0.8	0.5	0.15	0.15	0.15	0.00	0.00	0.00	0.001	-0.001	0.025
1	8	0	0.5	0.8	0.5	0.15	0.15	0.15	0.00	0.00	0.25	-0.012	-0.008	0.025
1	8	0	0.5	0.8	0.5	0.15	0.15	0.15	0.00	0.00	0.50	0.002	0.000	0.041
1	8	0	0.5	0.8	0.5	0.15	0.15	0.15	0.00	0.25	0.00	0.005	0.016	0.043
1	8	0	0.5	0.8	0.5	0.15	0.15	0.15	0.00	0.25	0.25	0.006	0.005	0.024
1	8	0	0.5	0.8	0.5	0.15	0.15	0.15	0.00	0.25	0.50	-0.001	0.005	0.031
1	8	0	0.5	0.8	0.5	0.15	0.15	0.15	0.00	0.50	0.00	0.012	-0.005	0.034

(continued)

												Mean Bias		
N	m_1	$\frac{m_2}{m_1}$	$E(C)$	$E(R)$	$E(U)$	σ_C	σ_R	σ_U	ρ_{CR}	ρ_{CU}	ρ_{RU}	c	r	u
1	8	0	0.5	0.8	0.5	0.15	0.15	0.15	0.00	0.50	0.25	-0.007	-0.001	0.025
1	8	0	0.5	0.8	0.5	0.15	0.15	0.15	0.00	0.50	0.50	0.009	-0.001	0.043
1	8	0	0.5	0.8	0.5	0.15	0.15	0.15	0.25	0.00	0.00	-0.003	0.005	0.024
1	8	0	0.5	0.8	0.5	0.15	0.15	0.15	0.25	0.00	0.25	0.001	-0.003	0.042
1	8	0	0.5	0.8	0.5	0.15	0.15	0.15	0.25	0.00	0.50	0.000	0.023	0.025
1	8	0	0.5	0.8	0.5	0.15	0.15	0.15	0.25	0.25	0.00	-0.002	-0.020	0.035
1	8	0	0.5	0.8	0.5	0.15	0.15	0.15	0.25	0.25	0.25	0.004	0.005	0.033
1	8	0	0.5	0.8	0.5	0.15	0.15	0.15	0.25	0.25	0.50	-0.001	0.014	0.035
1	8	0	0.5	0.8	0.5	0.15	0.15	0.15	0.25	0.50	0.00	0.013	-0.012	0.027
1	8	0	0.5	0.8	0.5	0.15	0.15	0.15	0.25	0.50	0.25	0.002	-0.006	0.013
1	8	0	0.5	0.8	0.5	0.15	0.15	0.15	0.25	0.50	0.50	-0.001	0.005	0.028
1	8	0	0.5	0.8	0.5	0.15	0.15	0.15	0.50	0.00	0.00	0.007	-0.004	0.057
1	8	0	0.5	0.8	0.5	0.15	0.15	0.15	0.50	0.00	0.25	-0.001	0.001	0.055
1	8	0	0.5	0.8	0.5	0.15	0.15	0.15	0.50	0.00	0.50	0.001	-0.005	0.043
1	8	0	0.5	0.8	0.5	0.15	0.15	0.15	0.50	0.25	0.00	0.003	0.006	0.036
1	8	0	0.5	0.8	0.5	0.15	0.15	0.15	0.50	0.25	0.25	0.013	0.017	0.030
1	8	0	0.5	0.8	0.5	0.15	0.15	0.15	0.50	0.25	0.50	-0.011	0.017	0.027
1	8	0	0.5	0.8	0.5	0.15	0.15	0.15	0.50	0.50	0.00	0.003	-0.012	0.026
1	8	0	0.5	0.8	0.5	0.15	0.15	0.15	0.50	0.50	0.25	0.023	0.000	0.045
1	8	0	0.5	0.8	0.5	0.15	0.15	0.15	0.50	0.50	0.50	0.008	-0.004	0.036
1	8	0	0.5	0.8	0.5	0.30	0.30	0.30	0.00	0.00	0.00	0.023	-0.098	0.006
1	8	0	0.5	0.8	0.5	0.30	0.30	0.30	0.00	0.00	0.25	0.031	-0.105	0.024
1	8	0	0.5	0.8	0.5	0.30	0.30	0.30	0.00	0.00	0.50	0.031	-0.082	0.030
1	8	0	0.5	0.8	0.5	0.30	0.30	0.30	0.00	0.25	0.00	0.033	-0.121	0.025
1	8	0	0.5	0.8	0.5	0.30	0.30	0.30	0.00	0.25	0.25	0.037	-0.117	0.019
1	8	0	0.5	0.8	0.5	0.30	0.30	0.30	0.00	0.25	0.50	0.000	-0.125	0.009
1	8	0	0.5	0.8	0.5	0.30	0.30	0.30	0.00	0.50	0.00	0.045	-0.127	0.010
1	8	0	0.5	0.8	0.5	0.30	0.30	0.30	0.00	0.50	0.25	0.044	-0.125	-0.006
1	8	0	0.5	0.8	0.5	0.30	0.30	0.30	0.00	0.50	0.50	0.059	-0.090	0.006
1	8	0	0.5	0.8	0.5	0.30	0.30	0.30	0.25	0.00	0.00	0.048	-0.100	0.045
1	8	0	0.5	0.8	0.5	0.30	0.30	0.30	0.25	0.00	0.25	0.034	-0.089	0.039
1	8	0	0.5	0.8	0.5	0.30	0.30	0.30	0.25	0.00	0.50	0.029	-0.081	0.031
1	8	0	0.5	0.8	0.5	0.30	0.30	0.30	0.25	0.25	0.00	0.057	-0.103	0.020
1	8	0	0.5	0.8	0.5	0.30	0.30	0.30	0.25	0.25	0.25	0.047	-0.100	0.024
1	8	0	0.5	0.8	0.5	0.30	0.30	0.30	0.25	0.25	0.50	0.051	-0.086	0.019
1	8	0	0.5	0.8	0.5	0.30	0.30	0.30	0.25	0.50	0.00	0.055	-0.081	0.011
1	8	0	0.5	0.8	0.5	0.30	0.30	0.30	0.25	0.50	0.25	0.051	-0.113	-0.002
1	8	0	0.5	0.8	0.5	0.30	0.30	0.30	0.25	0.50	0.50	0.043	-0.097	0.002
1	8	0	0.5	0.8	0.5	0.30	0.30	0.30	0.50	0.00	0.00	0.052	-0.093	0.052

(continued)

												Mean Bias		
N	m_1	$\frac{m_2}{m_1}$	$E(C)$	$E(R)$	$E(U)$	σ_C	σ_R	σ_U	ρ_{CR}	ρ_{CU}	ρ_{RU}	c	r	u
1	8	0	0.5	0.8	0.5	0.30	0.30	0.30	0.50	0.00	0.25	0.022	-0.078	0.056
1	8	0	0.5	0.8	0.5	0.30	0.30	0.30	0.50	0.00	0.50	0.027	-0.068	0.034
1	8	0	0.5	0.8	0.5	0.30	0.30	0.30	0.50	0.25	0.00	0.085	-0.099	0.016
1	8	0	0.5	0.8	0.5	0.30	0.30	0.30	0.50	0.25	0.25	0.052	-0.092	0.007
1	8	0	0.5	0.8	0.5	0.30	0.30	0.30	0.50	0.25	0.50	0.048	-0.096	0.010
1	8	0	0.5	0.8	0.5	0.30	0.30	0.30	0.50	0.50	0.00	0.044	-0.103	-0.001
1	8	0	0.5	0.8	0.5	0.30	0.30	0.30	0.50	0.50	0.25	0.056	-0.079	0.006
1	8	0	0.5	0.8	0.5	0.30	0.30	0.30	0.50	0.50	0.50	0.054	-0.067	-0.002
1	8	0	0.8	0.2	0.5	0.00	0.00	0.00	0.00	0.00	0.00	-0.280	0.254	-0.141
1	8	0	0.8	0.2	0.5	0.15	0.15	0.15	0.00	0.00	0.00	-0.237	0.174	-0.164
1	8	0	0.8	0.2	0.5	0.15	0.15	0.15	0.00	0.00	0.25	-0.252	0.150	-0.171
1	8	0	0.8	0.2	0.5	0.15	0.15	0.15	0.00	0.00	0.50	-0.259	0.148	-0.176
1	8	0	0.8	0.2	0.5	0.15	0.15	0.15	0.00	0.25	0.00	-0.236	0.156	-0.179
1	8	0	0.8	0.2	0.5	0.15	0.15	0.15	0.00	0.25	0.25	-0.269	0.138	-0.175
1	8	0	0.8	0.2	0.5	0.15	0.15	0.15	0.00	0.25	0.50	-0.253	0.152	-0.178
1	8	0	0.8	0.2	0.5	0.15	0.15	0.15	0.00	0.50	0.00	-0.243	0.127	-0.176
1	8	0	0.8	0.2	0.5	0.15	0.15	0.15	0.00	0.50	0.25	-0.236	0.163	-0.178
1	8	0	0.8	0.2	0.5	0.15	0.15	0.15	0.00	0.50	0.50	-0.263	0.141	-0.181
1	8	0	0.8	0.2	0.5	0.15	0.15	0.15	0.25	0.00	0.00	-0.238	0.140	-0.159
1	8	0	0.8	0.2	0.5	0.15	0.15	0.15	0.25	0.00	0.25	-0.219	0.145	-0.160
1	8	0	0.8	0.2	0.5	0.15	0.15	0.15	0.25	0.00	0.50	-0.219	0.133	-0.164
1	8	0	0.8	0.2	0.5	0.15	0.15	0.15	0.25	0.25	0.00	-0.240	0.130	-0.152
1	8	0	0.8	0.2	0.5	0.15	0.15	0.15	0.25	0.25	0.25	-0.250	0.137	-0.187
1	8	0	0.8	0.2	0.5	0.15	0.15	0.15	0.25	0.25	0.50	-0.237	0.158	-0.169
1	8	0	0.8	0.2	0.5	0.15	0.15	0.15	0.25	0.50	0.00	-0.237	0.158	-0.163
1	8	0	0.8	0.2	0.5	0.15	0.15	0.15	0.25	0.50	0.25	-0.241	0.164	-0.185
1	8	0	0.8	0.2	0.5	0.15	0.15	0.15	0.25	0.50	0.50	-0.251	0.165	-0.167
1	8	0	0.8	0.2	0.5	0.15	0.15	0.15	0.50	0.00	0.00	-0.206	0.141	-0.144
1	8	0	0.8	0.2	0.5	0.15	0.15	0.15	0.50	0.00	0.25	-0.236	0.123	-0.154
1	8	0	0.8	0.2	0.5	0.15	0.15	0.15	0.50	0.00	0.50	-0.234	0.122	-0.160
1	8	0	0.8	0.2	0.5	0.15	0.15	0.15	0.50	0.25	0.00	-0.228	0.169	-0.161
1	8	0	0.8	0.2	0.5	0.15	0.15	0.15	0.50	0.25	0.25	-0.246	0.147	-0.177
1	8	0	0.8	0.2	0.5	0.15	0.15	0.15	0.50	0.25	0.50	-0.243	0.154	-0.177
1	8	0	0.8	0.2	0.5	0.15	0.15	0.15	0.50	0.50	0.00	-0.237	0.150	-0.154
1	8	0	0.8	0.2	0.5	0.15	0.15	0.15	0.50	0.50	0.25	-0.216	0.157	-0.164
1	8	0	0.8	0.2	0.5	0.15	0.15	0.15	0.50	0.50	0.50	-0.239	0.171	-0.193
1	8	0	0.8	0.2	0.5	0.30	0.30	0.30	0.00	0.00	0.00	-0.191	0.044	-0.218
1	8	0	0.8	0.2	0.5	0.30	0.30	0.30	0.00	0.00	0.25	-0.188	0.030	-0.198
1	8	0	0.8	0.2	0.5	0.30	0.30	0.30	0.00	0.00	0.50	-0.228	0.052	-0.211

(continued)

												Mean Bias		
N	m_1	$\frac{m_2}{m_1}$	$E(C)$	$E(R)$	$E(U)$	σ_C	σ_R	σ_U	ρ_{CR}	ρ_{CU}	ρ_{RU}	c	r	u
1	8	0	0.8	0.2	0.5	0.30	0.30	0.30	0.00	0.25	0.00	-0.170	0.044	-0.249
1	8	0	0.8	0.2	0.5	0.30	0.30	0.30	0.00	0.25	0.25	-0.202	0.030	-0.247
1	8	0	0.8	0.2	0.5	0.30	0.30	0.30	0.00	0.25	0.50	-0.202	0.053	-0.260
1	8	0	0.8	0.2	0.5	0.30	0.30	0.30	0.00	0.50	0.00	-0.195	0.039	-0.281
1	8	0	0.8	0.2	0.5	0.30	0.30	0.30	0.00	0.50	0.25	-0.176	0.033	-0.282
1	8	0	0.8	0.2	0.5	0.30	0.30	0.30	0.00	0.50	0.50	-0.183	0.020	-0.273
1	8	0	0.8	0.2	0.5	0.30	0.30	0.30	0.25	0.00	0.00	-0.177	0.042	-0.192
1	8	0	0.8	0.2	0.5	0.30	0.30	0.30	0.25	0.00	0.25	-0.211	0.040	-0.201
1	8	0	0.8	0.2	0.5	0.30	0.30	0.30	0.25	0.00	0.50	-0.202	0.043	-0.219
1	8	0	0.8	0.2	0.5	0.30	0.30	0.30	0.25	0.25	0.00	-0.173	0.055	-0.253
1	8	0	0.8	0.2	0.5	0.30	0.30	0.30	0.25	0.25	0.25	-0.168	0.037	-0.231
1	8	0	0.8	0.2	0.5	0.30	0.30	0.30	0.25	0.25	0.50	-0.214	0.055	-0.244
1	8	0	0.8	0.2	0.5	0.30	0.30	0.30	0.25	0.50	0.00	-0.168	0.055	-0.264
1	8	0	0.8	0.2	0.5	0.30	0.30	0.30	0.25	0.50	0.25	-0.173	0.055	-0.266
1	8	0	0.8	0.2	0.5	0.30	0.30	0.30	0.25	0.50	0.50	-0.192	0.054	-0.268
1	8	0	0.8	0.2	0.5	0.30	0.30	0.30	0.50	0.00	0.00	-0.187	0.052	-0.206
1	8	0	0.8	0.2	0.5	0.30	0.30	0.30	0.50	0.00	0.25	-0.145	0.045	-0.185
1	8	0	0.8	0.2	0.5	0.30	0.30	0.30	0.50	0.00	0.50	-0.175	0.073	-0.213
1	8	0	0.8	0.2	0.5	0.30	0.30	0.30	0.50	0.25	0.00	-0.164	0.038	-0.235
1	8	0	0.8	0.2	0.5	0.30	0.30	0.30	0.50	0.25	0.25	-0.172	0.074	-0.243
1	8	0	0.8	0.2	0.5	0.30	0.30	0.30	0.50	0.25	0.50	-0.177	0.029	-0.219
1	8	0	0.8	0.2	0.5	0.30	0.30	0.30	0.50	0.50	0.00	-0.138	0.052	-0.242
1	8	0	0.8	0.2	0.5	0.30	0.30	0.30	0.50	0.50	0.25	-0.178	0.033	-0.269
1	8	0	0.8	0.2	0.5	0.30	0.30	0.30	0.50	0.50	0.50	-0.155	0.051	-0.265
1	8	0	0.8	0.5	0.5	0.00	0.00	0.00	0.00	0.00	0.00	-0.164	0.202	-0.107
1	8	0	0.8	0.5	0.5	0.15	0.15	0.15	0.00	0.00	0.00	-0.113	0.135	-0.096
1	8	0	0.8	0.5	0.5	0.15	0.15	0.15	0.00	0.00	0.25	-0.123	0.151	-0.110
1	8	0	0.8	0.5	0.5	0.15	0.15	0.15	0.00	0.00	0.50	-0.134	0.171	-0.136
1	8	0	0.8	0.5	0.5	0.15	0.15	0.15	0.00	0.25	0.00	-0.156	0.165	-0.137
1	8	0	0.8	0.5	0.5	0.15	0.15	0.15	0.00	0.25	0.25	-0.134	0.150	-0.123
1	8	0	0.8	0.5	0.5	0.15	0.15	0.15	0.00	0.25	0.50	-0.119	0.138	-0.099
1	8	0	0.8	0.5	0.5	0.15	0.15	0.15	0.00	0.50	0.00	-0.123	0.153	-0.126
1	8	0	0.8	0.5	0.5	0.15	0.15	0.15	0.00	0.50	0.25	-0.123	0.150	-0.134
1	8	0	0.8	0.5	0.5	0.15	0.15	0.15	0.00	0.50	0.50	-0.145	0.156	-0.128
1	8	0	0.8	0.5	0.5	0.15	0.15	0.15	0.25	0.00	0.00	-0.125	0.156	-0.114
1	8	0	0.8	0.5	0.5	0.15	0.15	0.15	0.25	0.00	0.25	-0.114	0.138	-0.102
1	8	0	0.8	0.5	0.5	0.15	0.15	0.15	0.25	0.00	0.50	-0.122	0.141	-0.111
1	8	0	0.8	0.5	0.5	0.15	0.15	0.15	0.25	0.25	0.00	-0.130	0.151	-0.117
1	8	0	0.8	0.5	0.5	0.15	0.15	0.15	0.25	0.25	0.25	-0.131	0.149	-0.117

(continued)

												Mean Bias		
N	m_1	$\frac{m_2}{m_1}$	$E(C)$	$E(R)$	$E(U)$	σ_C	σ_R	σ_U	ρ_{CR}	ρ_{CU}	ρ_{RU}	c	r	u
1	8	0	0.8	0.5	0.5	0.15	0.15	0.15	0.25	0.25	0.50	-0.137	0.150	-0.106
1	8	0	0.8	0.5	0.5	0.15	0.15	0.15	0.25	0.50	0.00	-0.123	0.134	-0.127
1	8	0	0.8	0.5	0.5	0.15	0.15	0.15	0.25	0.50	0.25	-0.132	0.156	-0.129
1	8	0	0.8	0.5	0.5	0.15	0.15	0.15	0.25	0.50	0.50	-0.139	0.150	-0.135
1	8	0	0.8	0.5	0.5	0.15	0.15	0.15	0.50	0.00	0.00	-0.123	0.150	-0.107
1	8	0	0.8	0.5	0.5	0.15	0.15	0.15	0.50	0.00	0.25	-0.121	0.137	-0.114
1	8	0	0.8	0.5	0.5	0.15	0.15	0.15	0.50	0.00	0.50	-0.134	0.161	-0.117
1	8	0	0.8	0.5	0.5	0.15	0.15	0.15	0.50	0.25	0.00	-0.123	0.156	-0.136
1	8	0	0.8	0.5	0.5	0.15	0.15	0.15	0.50	0.25	0.25	-0.130	0.172	-0.124
1	8	0	0.8	0.5	0.5	0.15	0.15	0.15	0.50	0.25	0.50	-0.130	0.157	-0.130
1	8	0	0.8	0.5	0.5	0.15	0.15	0.15	0.50	0.50	0.00	-0.137	0.156	-0.135
1	8	0	0.8	0.5	0.5	0.15	0.15	0.15	0.50	0.50	0.25	-0.132	0.163	-0.122
1	8	0	0.8	0.5	0.5	0.15	0.15	0.15	0.50	0.50	0.50	-0.130	0.161	-0.121
1	8	0	0.8	0.5	0.5	0.30	0.30	0.30	0.00	0.00	0.00	-0.083	0.032	-0.153
1	8	0	0.8	0.5	0.5	0.30	0.30	0.30	0.00	0.00	0.25	-0.087	0.027	-0.144
1	8	0	0.8	0.5	0.5	0.30	0.30	0.30	0.00	0.00	0.50	-0.073	0.054	-0.145
1	8	0	0.8	0.5	0.5	0.30	0.30	0.30	0.00	0.25	0.00	-0.080	0.004	-0.179
1	8	0	0.8	0.5	0.5	0.30	0.30	0.30	0.00	0.25	0.25	-0.071	0.031	-0.171
1	8	0	0.8	0.5	0.5	0.30	0.30	0.30	0.00	0.25	0.50	-0.093	0.014	-0.169
1	8	0	0.8	0.5	0.5	0.30	0.30	0.30	0.00	0.50	0.00	-0.084	0.003	-0.180
1	8	0	0.8	0.5	0.5	0.30	0.30	0.30	0.00	0.50	0.25	-0.078	0.043	-0.210
1	8	0	0.8	0.5	0.5	0.30	0.30	0.30	0.00	0.50	0.50	-0.082	0.037	-0.189
1	8	0	0.8	0.5	0.5	0.30	0.30	0.30	0.25	0.00	0.00	-0.071	0.031	-0.126
1	8	0	0.8	0.5	0.5	0.30	0.30	0.30	0.25	0.00	0.25	-0.056	0.031	-0.129
1	8	0	0.8	0.5	0.5	0.30	0.30	0.30	0.25	0.00	0.50	-0.083	0.069	-0.108
1	8	0	0.8	0.5	0.5	0.30	0.30	0.30	0.25	0.25	0.00	-0.071	0.044	-0.157
1	8	0	0.8	0.5	0.5	0.30	0.30	0.30	0.25	0.25	0.25	-0.057	0.017	-0.169
1	8	0	0.8	0.5	0.5	0.30	0.30	0.30	0.25	0.25	0.50	-0.078	0.020	-0.166
1	8	0	0.8	0.5	0.5	0.30	0.30	0.30	0.25	0.50	0.00	-0.064	0.030	-0.209
1	8	0	0.8	0.5	0.5	0.30	0.30	0.30	0.25	0.50	0.25	-0.065	0.033	-0.185
1	8	0	0.8	0.5	0.5	0.30	0.30	0.30	0.25	0.50	0.50	-0.075	0.026	-0.200
1	8	0	0.8	0.5	0.5	0.30	0.30	0.30	0.50	0.00	0.00	-0.067	0.045	-0.124
1	8	0	0.8	0.5	0.5	0.30	0.30	0.30	0.50	0.00	0.25	-0.054	0.043	-0.134
1	8	0	0.8	0.5	0.5	0.30	0.30	0.30	0.50	0.00	0.50	-0.071	0.059	-0.125
1	8	0	0.8	0.5	0.5	0.30	0.30	0.30	0.50	0.25	0.00	-0.077	0.038	-0.142
1	8	0	0.8	0.5	0.5	0.30	0.30	0.30	0.50	0.25	0.25	-0.078	0.037	-0.144
1	8	0	0.8	0.5	0.5	0.30	0.30	0.30	0.50	0.25	0.50	-0.080	0.072	-0.139
1	8	0	0.8	0.5	0.5	0.30	0.30	0.30	0.50	0.50	0.00	-0.053	0.042	-0.170
1	8	0	0.8	0.5	0.5	0.30	0.30	0.30	0.50	0.50	0.25	-0.084	0.027	-0.178

(continued)

N	m_1	$\frac{m_2}{m_1}$	$E(C)$	$E(R)$	$E(U)$	σ_C	σ_R	σ_U	ρ_{CR}	ρ_{CU}	ρ_{RU}	Mean Bias		
												c	r	u
1	8	0	0.8	0.5	0.5	0.30	0.30	0.30	0.50	0.50	0.50	-0.059	0.071	-0.182
1	8	0	0.8	0.8	0.5	0.00	0.00	0.00	0.00	0.00	0.00	-0.034	0.049	-0.016
1	8	0	0.8	0.8	0.5	0.15	0.15	0.15	0.00	0.00	0.00	-0.028	0.043	-0.052
1	8	0	0.8	0.8	0.5	0.15	0.15	0.15	0.00	0.00	0.25	-0.047	0.042	-0.035
1	8	0	0.8	0.8	0.5	0.15	0.15	0.15	0.00	0.00	0.50	-0.034	0.049	-0.047
1	8	0	0.8	0.8	0.5	0.15	0.15	0.15	0.00	0.25	0.00	-0.038	0.052	-0.044
1	8	0	0.8	0.8	0.5	0.15	0.15	0.15	0.00	0.25	0.25	-0.024	0.047	-0.026
1	8	0	0.8	0.8	0.5	0.15	0.15	0.15	0.00	0.25	0.50	-0.048	0.039	-0.059
1	8	0	0.8	0.8	0.5	0.15	0.15	0.15	0.00	0.50	0.00	-0.030	0.051	-0.053
1	8	0	0.8	0.8	0.5	0.15	0.15	0.15	0.00	0.50	0.25	-0.034	0.035	-0.065
1	8	0	0.8	0.8	0.5	0.15	0.15	0.15	0.00	0.50	0.50	-0.038	0.044	-0.068
1	8	0	0.8	0.8	0.5	0.15	0.15	0.15	0.25	0.00	0.00	-0.032	0.049	-0.037
1	8	0	0.8	0.8	0.5	0.15	0.15	0.15	0.25	0.00	0.25	-0.032	0.049	-0.043
1	8	0	0.8	0.8	0.5	0.15	0.15	0.15	0.25	0.00	0.50	-0.041	0.059	-0.034
1	8	0	0.8	0.8	0.5	0.15	0.15	0.15	0.25	0.25	0.00	-0.036	0.051	-0.037
1	8	0	0.8	0.8	0.5	0.15	0.15	0.15	0.25	0.25	0.25	-0.031	0.060	-0.047
1	8	0	0.8	0.8	0.5	0.15	0.15	0.15	0.25	0.25	0.50	-0.041	0.049	-0.062
1	8	0	0.8	0.8	0.5	0.15	0.15	0.15	0.25	0.50	0.00	-0.023	0.038	-0.072
1	8	0	0.8	0.8	0.5	0.15	0.15	0.15	0.25	0.50	0.25	-0.038	0.053	-0.038
1	8	0	0.8	0.8	0.5	0.15	0.15	0.15	0.25	0.50	0.50	-0.026	0.036	-0.055
1	8	0	0.8	0.8	0.5	0.15	0.15	0.15	0.50	0.00	0.00	-0.030	0.059	-0.031
1	8	0	0.8	0.8	0.5	0.15	0.15	0.15	0.50	0.00	0.25	-0.032	0.045	-0.026
1	8	0	0.8	0.8	0.5	0.15	0.15	0.15	0.50	0.00	0.50	-0.029	0.051	-0.044
1	8	0	0.8	0.8	0.5	0.15	0.15	0.15	0.50	0.25	0.00	-0.033	0.045	-0.042
1	8	0	0.8	0.8	0.5	0.15	0.15	0.15	0.50	0.25	0.25	-0.039	0.053	-0.038
1	8	0	0.8	0.8	0.5	0.15	0.15	0.15	0.50	0.25	0.50	-0.042	0.052	-0.026
1	8	0	0.8	0.8	0.5	0.15	0.15	0.15	0.50	0.50	0.00	-0.029	0.039	-0.038
1	8	0	0.8	0.8	0.5	0.15	0.15	0.15	0.50	0.50	0.25	-0.034	0.044	-0.064
1	8	0	0.8	0.8	0.5	0.15	0.15	0.15	0.50	0.50	0.50	-0.027	0.055	-0.065
1	8	0	0.8	0.8	0.5	0.30	0.30	0.30	0.00	0.00	0.00	-0.002	-0.045	-0.046
1	8	0	0.8	0.8	0.5	0.30	0.30	0.30	0.00	0.00	0.25	-0.024	-0.051	-0.064
1	8	0	0.8	0.8	0.5	0.30	0.30	0.30	0.00	0.00	0.50	-0.015	-0.010	-0.060
1	8	0	0.8	0.8	0.5	0.30	0.30	0.30	0.00	0.25	0.00	-0.014	-0.062	-0.085
1	8	0	0.8	0.8	0.5	0.30	0.30	0.30	0.00	0.25	0.25	-0.006	-0.046	-0.075
1	8	0	0.8	0.8	0.5	0.30	0.30	0.30	0.00	0.25	0.50	-0.037	-0.043	-0.093
1	8	0	0.8	0.8	0.5	0.30	0.30	0.30	0.00	0.50	0.00	-0.008	-0.048	-0.104
1	8	0	0.8	0.8	0.5	0.30	0.30	0.30	0.00	0.50	0.25	0.002	-0.047	-0.102
1	8	0	0.8	0.8	0.5	0.30	0.30	0.30	0.00	0.50	0.50	-0.013	-0.034	-0.112
1	8	0	0.8	0.8	0.5	0.30	0.30	0.30	0.25	0.00	0.00	0.035	0.009	-0.049

(continued)

												Mean Bias		
N	m_1	$\frac{m_2}{m_1}$	$E(C)$	$E(R)$	$E(U)$	σ_C	σ_R	σ_U	ρ_{CR}	ρ_{CU}	ρ_{RU}	c	r	u
1	8	0	0.8	0.8	0.5	0.30	0.30	0.30	0.25	0.00	0.25	-0.019	-0.041	-0.046
1	8	0	0.8	0.8	0.5	0.30	0.30	0.30	0.25	0.00	0.50	-0.027	-0.027	-0.041
1	8	0	0.8	0.8	0.5	0.30	0.30	0.30	0.25	0.25	0.00	0.003	-0.033	-0.070
1	8	0	0.8	0.8	0.5	0.30	0.30	0.30	0.25	0.25	0.25	-0.033	-0.051	-0.083
1	8	0	0.8	0.8	0.5	0.30	0.30	0.30	0.25	0.25	0.50	-0.006	-0.023	-0.057
1	8	0	0.8	0.8	0.5	0.30	0.30	0.30	0.25	0.50	0.00	0.009	-0.033	-0.091
1	8	0	0.8	0.8	0.5	0.30	0.30	0.30	0.25	0.50	0.25	-0.013	-0.034	-0.098
1	8	0	0.8	0.8	0.5	0.30	0.30	0.30	0.25	0.50	0.50	-0.007	-0.040	-0.096
1	8	0	0.8	0.8	0.5	0.30	0.30	0.30	0.50	0.00	0.00	0.008	-0.013	-0.032
1	8	0	0.8	0.8	0.5	0.30	0.30	0.30	0.50	0.00	0.25	-0.010	-0.028	-0.034
1	8	0	0.8	0.8	0.5	0.30	0.30	0.30	0.50	0.00	0.50	-0.031	-0.035	-0.049
1	8	0	0.8	0.8	0.5	0.30	0.30	0.30	0.50	0.25	0.00	0.021	-0.031	-0.066
1	8	0	0.8	0.8	0.5	0.30	0.30	0.30	0.50	0.25	0.25	0.000	-0.022	-0.067
1	8	0	0.8	0.8	0.5	0.30	0.30	0.30	0.50	0.25	0.50	0.011	-0.007	-0.067
1	8	0	0.8	0.8	0.5	0.30	0.30	0.30	0.50	0.50	0.00	0.002	-0.037	-0.078
1	8	0	0.8	0.8	0.5	0.30	0.30	0.30	0.50	0.50	0.25	0.016	-0.021	-0.090
1	8	0	0.8	0.8	0.5	0.30	0.30	0.30	0.50	0.50	0.50	0.018	-0.003	-0.091
1	8	1	0.2	0.2	0.5	0.00	0.00	0.00	0.00	0.00	0.00	0.023	-0.020	0.015
1	8	1	0.2	0.2	0.5	0.15	0.15	0.15	0.00	0.00	0.00	0.050	-0.037	0.010
1	8	1	0.2	0.2	0.5	0.15	0.15	0.15	0.00	0.00	0.25	0.028	-0.043	0.006
1	8	1	0.2	0.2	0.5	0.15	0.15	0.15	0.00	0.00	0.50	0.031	-0.029	0.030
1	8	1	0.2	0.2	0.5	0.15	0.15	0.15	0.00	0.25	0.00	0.039	-0.047	0.022
1	8	1	0.2	0.2	0.5	0.15	0.15	0.15	0.00	0.25	0.25	0.021	-0.040	0.016
1	8	1	0.2	0.2	0.5	0.15	0.15	0.15	0.00	0.25	0.50	0.028	-0.046	0.022
1	8	1	0.2	0.2	0.5	0.15	0.15	0.15	0.00	0.50	0.00	0.045	-0.050	0.022
1	8	1	0.2	0.2	0.5	0.15	0.15	0.15	0.00	0.50	0.25	0.041	-0.030	0.025
1	8	1	0.2	0.2	0.5	0.15	0.15	0.15	0.00	0.50	0.50	0.039	-0.053	0.014
1	8	1	0.2	0.2	0.5	0.15	0.15	0.15	0.25	0.00	0.00	0.044	-0.015	0.027
1	8	1	0.2	0.2	0.5	0.15	0.15	0.15	0.25	0.00	0.25	0.026	-0.034	0.013
1	8	1	0.2	0.2	0.5	0.15	0.15	0.15	0.25	0.00	0.50	0.017	-0.027	0.019
1	8	1	0.2	0.2	0.5	0.15	0.15	0.15	0.25	0.25	0.00	0.036	-0.019	0.020
1	8	1	0.2	0.2	0.5	0.15	0.15	0.15	0.25	0.25	0.25	0.043	-0.034	0.013
1	8	1	0.2	0.2	0.5	0.15	0.15	0.15	0.25	0.25	0.50	0.030	-0.029	0.013
1	8	1	0.2	0.2	0.5	0.15	0.15	0.15	0.25	0.50	0.00	0.027	-0.041	0.015
1	8	1	0.2	0.2	0.5	0.15	0.15	0.15	0.25	0.50	0.25	0.037	-0.043	0.014
1	8	1	0.2	0.2	0.5	0.15	0.15	0.15	0.25	0.50	0.50	0.038	-0.039	0.020
1	8	1	0.2	0.2	0.5	0.15	0.15	0.15	0.50	0.00	0.00	0.040	-0.022	0.008
1	8	1	0.2	0.2	0.5	0.15	0.15	0.15	0.50	0.00	0.25	0.038	-0.014	0.008
1	8	1	0.2	0.2	0.5	0.15	0.15	0.15	0.50	0.00	0.50	0.026	-0.012	0.007

(continued)

												Mean Bias		
N	m_1	$\frac{m_2}{m_1}$	$E(C)$	$E(R)$	$E(U)$	σ_C	σ_R	σ_U	ρ_{CR}	ρ_{CU}	ρ_{RU}	c	r	u
1	8	1	0.2	0.2	0.5	0.15	0.15	0.15	0.50	0.25	0.00	0.036	-0.033	0.020
1	8	1	0.2	0.2	0.5	0.15	0.15	0.15	0.50	0.25	0.25	0.042	-0.003	0.008
1	8	1	0.2	0.2	0.5	0.15	0.15	0.15	0.50	0.25	0.50	0.026	0.005	0.017
1	8	1	0.2	0.2	0.5	0.15	0.15	0.15	0.50	0.50	0.00	0.041	-0.043	0.019
1	8	1	0.2	0.2	0.5	0.15	0.15	0.15	0.50	0.50	0.25	0.043	-0.030	0.014
1	8	1	0.2	0.2	0.5	0.15	0.15	0.15	0.50	0.50	0.50	0.025	-0.030	0.015
1	8	1	0.2	0.2	0.5	0.30	0.30	0.30	0.00	0.00	0.00	0.067	-0.051	0.025
1	8	1	0.2	0.2	0.5	0.30	0.30	0.30	0.00	0.00	0.25	0.084	-0.067	0.022
1	8	1	0.2	0.2	0.5	0.30	0.30	0.30	0.00	0.00	0.50	0.060	-0.056	0.025
1	8	1	0.2	0.2	0.5	0.30	0.30	0.30	0.00	0.25	0.00	0.062	-0.057	0.015
1	8	1	0.2	0.2	0.5	0.30	0.30	0.30	0.00	0.25	0.25	0.089	-0.063	0.004
1	8	1	0.2	0.2	0.5	0.30	0.30	0.30	0.00	0.25	0.50	0.062	-0.075	0.011
1	8	1	0.2	0.2	0.5	0.30	0.30	0.30	0.00	0.50	0.00	0.081	-0.080	-0.002
1	8	1	0.2	0.2	0.5	0.30	0.30	0.30	0.00	0.50	0.25	0.085	-0.057	0.042
1	8	1	0.2	0.2	0.5	0.30	0.30	0.30	0.00	0.50	0.50	0.079	-0.074	0.026
1	8	1	0.2	0.2	0.5	0.30	0.30	0.30	0.25	0.00	0.00	0.081	-0.035	-0.007
1	8	1	0.2	0.2	0.5	0.30	0.30	0.30	0.25	0.00	0.25	0.084	-0.041	0.011
1	8	1	0.2	0.2	0.5	0.30	0.30	0.30	0.25	0.00	0.50	0.071	-0.041	0.003
1	8	1	0.2	0.2	0.5	0.30	0.30	0.30	0.25	0.25	0.00	0.082	-0.057	0.016
1	8	1	0.2	0.2	0.5	0.30	0.30	0.30	0.25	0.25	0.25	0.098	-0.045	0.017
1	8	1	0.2	0.2	0.5	0.30	0.30	0.30	0.25	0.25	0.50	0.088	-0.040	0.015
1	8	1	0.2	0.2	0.5	0.30	0.30	0.30	0.25	0.50	0.00	0.093	-0.053	0.017
1	8	1	0.2	0.2	0.5	0.30	0.30	0.30	0.25	0.50	0.25	0.083	-0.044	0.026
1	8	1	0.2	0.2	0.5	0.30	0.30	0.30	0.25	0.50	0.50	0.103	-0.040	0.030
1	8	1	0.2	0.2	0.5	0.30	0.30	0.30	0.50	0.00	0.00	0.089	-0.007	0.024
1	8	1	0.2	0.2	0.5	0.30	0.30	0.30	0.50	0.00	0.25	0.068	-0.034	0.008
1	8	1	0.2	0.2	0.5	0.30	0.30	0.30	0.50	0.00	0.50	0.071	0.007	0.027
1	8	1	0.2	0.2	0.5	0.30	0.30	0.30	0.50	0.25	0.00	0.089	-0.028	0.000
1	8	1	0.2	0.2	0.5	0.30	0.30	0.30	0.50	0.25	0.25	0.089	-0.022	0.018
1	8	1	0.2	0.2	0.5	0.30	0.30	0.30	0.50	0.25	0.50	0.077	-0.028	0.023
1	8	1	0.2	0.2	0.5	0.30	0.30	0.30	0.50	0.50	0.00	0.095	-0.044	0.002
1	8	1	0.2	0.2	0.5	0.30	0.30	0.30	0.50	0.50	0.25	0.095	-0.009	0.018
1	8	1	0.2	0.2	0.5	0.30	0.30	0.30	0.50	0.50	0.50	0.102	-0.037	0.006
1	8	1	0.2	0.5	0.5	0.00	0.00	0.00	0.00	0.00	0.00	0.034	-0.091	0.025
1	8	1	0.2	0.5	0.5	0.15	0.15	0.15	0.00	0.00	0.00	0.048	-0.177	0.024
1	8	1	0.2	0.5	0.5	0.15	0.15	0.15	0.00	0.00	0.25	0.050	-0.160	0.027
1	8	1	0.2	0.5	0.5	0.15	0.15	0.15	0.00	0.00	0.50	0.053	-0.139	0.018
1	8	1	0.2	0.5	0.5	0.15	0.15	0.15	0.00	0.25	0.00	0.054	-0.167	0.028
1	8	1	0.2	0.5	0.5	0.15	0.15	0.15	0.00	0.25	0.25	0.050	-0.136	0.016

(continued)

												Mean Bias		
N	m_1	$\frac{m_2}{m_1}$	$E(C)$	$E(R)$	$E(U)$	σ_C	σ_R	σ_U	ρ_{CR}	ρ_{CU}	ρ_{RU}	c	r	u
1	8	1	0.2	0.5	0.5	0.15	0.15	0.15	0.00	0.25	0.50	0.064	-0.135	0.021
1	8	1	0.2	0.5	0.5	0.15	0.15	0.15	0.00	0.50	0.00	0.055	-0.133	0.027
1	8	1	0.2	0.5	0.5	0.15	0.15	0.15	0.00	0.50	0.25	0.052	-0.160	0.020
1	8	1	0.2	0.5	0.5	0.15	0.15	0.15	0.00	0.50	0.50	0.059	-0.139	0.021
1	8	1	0.2	0.5	0.5	0.15	0.15	0.15	0.25	0.00	0.00	0.050	-0.159	0.018
1	8	1	0.2	0.5	0.5	0.15	0.15	0.15	0.25	0.00	0.25	0.042	-0.154	0.014
1	8	1	0.2	0.5	0.5	0.15	0.15	0.15	0.25	0.00	0.50	0.052	-0.126	0.019
1	8	1	0.2	0.5	0.5	0.15	0.15	0.15	0.25	0.25	0.00	0.054	-0.158	0.020
1	8	1	0.2	0.5	0.5	0.15	0.15	0.15	0.25	0.25	0.25	0.037	-0.173	0.018
1	8	1	0.2	0.5	0.5	0.15	0.15	0.15	0.25	0.25	0.50	0.053	-0.126	0.030
1	8	1	0.2	0.5	0.5	0.15	0.15	0.15	0.25	0.50	0.00	0.057	-0.146	0.020
1	8	1	0.2	0.5	0.5	0.15	0.15	0.15	0.25	0.50	0.25	0.060	-0.149	0.019
1	8	1	0.2	0.5	0.5	0.15	0.15	0.15	0.25	0.50	0.50	0.053	-0.133	0.028
1	8	1	0.2	0.5	0.5	0.15	0.15	0.15	0.50	0.00	0.00	0.045	-0.160	0.015
1	8	1	0.2	0.5	0.5	0.15	0.15	0.15	0.50	0.00	0.25	0.045	-0.148	0.014
1	8	1	0.2	0.5	0.5	0.15	0.15	0.15	0.50	0.00	0.50	0.036	-0.146	0.022
1	8	1	0.2	0.5	0.5	0.15	0.15	0.15	0.50	0.25	0.00	0.050	-0.113	0.018
1	8	1	0.2	0.5	0.5	0.15	0.15	0.15	0.50	0.25	0.25	0.049	-0.154	0.017
1	8	1	0.2	0.5	0.5	0.15	0.15	0.15	0.50	0.25	0.50	0.058	-0.141	0.020
1	8	1	0.2	0.5	0.5	0.15	0.15	0.15	0.50	0.50	0.00	0.056	-0.151	0.021
1	8	1	0.2	0.5	0.5	0.15	0.15	0.15	0.50	0.50	0.25	0.056	-0.141	0.017
1	8	1	0.2	0.5	0.5	0.15	0.15	0.15	0.50	0.50	0.50	0.061	-0.123	0.027
1	8	1	0.2	0.5	0.5	0.30	0.30	0.30	0.00	0.00	0.00	0.078	-0.263	0.026
1	8	1	0.2	0.5	0.5	0.30	0.30	0.30	0.00	0.00	0.25	0.076	-0.260	0.029
1	8	1	0.2	0.5	0.5	0.30	0.30	0.30	0.00	0.00	0.50	0.097	-0.267	0.011
1	8	1	0.2	0.5	0.5	0.30	0.30	0.30	0.00	0.25	0.00	0.097	-0.264	0.011
1	8	1	0.2	0.5	0.5	0.30	0.30	0.30	0.00	0.25	0.25	0.091	-0.252	0.023
1	8	1	0.2	0.5	0.5	0.30	0.30	0.30	0.00	0.25	0.50	0.099	-0.263	-0.001
1	8	1	0.2	0.5	0.5	0.30	0.30	0.30	0.00	0.50	0.00	0.091	-0.253	0.020
1	8	1	0.2	0.5	0.5	0.30	0.30	0.30	0.00	0.50	0.25	0.091	-0.265	0.033
1	8	1	0.2	0.5	0.5	0.30	0.30	0.30	0.00	0.50	0.50	0.084	-0.269	0.013
1	8	1	0.2	0.5	0.5	0.30	0.30	0.30	0.25	0.00	0.00	0.062	-0.237	0.019
1	8	1	0.2	0.5	0.5	0.30	0.30	0.30	0.25	0.00	0.25	0.081	-0.213	0.032
1	8	1	0.2	0.5	0.5	0.30	0.30	0.30	0.25	0.00	0.50	0.091	-0.222	0.011
1	8	1	0.2	0.5	0.5	0.30	0.30	0.30	0.25	0.25	0.00	0.090	-0.230	0.023
1	8	1	0.2	0.5	0.5	0.30	0.30	0.30	0.25	0.25	0.25	0.090	-0.229	0.006
1	8	1	0.2	0.5	0.5	0.30	0.30	0.30	0.25	0.25	0.50	0.083	-0.226	0.018
1	8	1	0.2	0.5	0.5	0.30	0.30	0.30	0.25	0.50	0.00	0.099	-0.231	0.008
1	8	1	0.2	0.5	0.5	0.30	0.30	0.30	0.25	0.50	0.25	0.101	-0.235	0.008

(continued)

												Mean Bias		
N	m_1	$\frac{m_2}{m_1}$	$E(C)$	$E(R)$	$E(U)$	σ_C	σ_R	σ_U	ρ_{CR}	ρ_{CU}	ρ_{RU}	c	r	u
1	8	1	0.2	0.5	0.5	0.30	0.30	0.30	0.25	0.50	0.50	0.100	-0.246	0.012
1	8	1	0.2	0.5	0.5	0.30	0.30	0.30	0.50	0.00	0.00	0.088	-0.202	0.020
1	8	1	0.2	0.5	0.5	0.30	0.30	0.30	0.50	0.00	0.25	0.094	-0.187	0.024
1	8	1	0.2	0.5	0.5	0.30	0.30	0.30	0.50	0.00	0.50	0.085	-0.177	0.027
1	8	1	0.2	0.5	0.5	0.30	0.30	0.30	0.50	0.25	0.00	0.091	-0.206	0.014
1	8	1	0.2	0.5	0.5	0.30	0.30	0.30	0.50	0.25	0.25	0.079	-0.231	0.015
1	8	1	0.2	0.5	0.5	0.30	0.30	0.30	0.50	0.25	0.50	0.098	-0.195	0.010
1	8	1	0.2	0.5	0.5	0.30	0.30	0.30	0.50	0.50	0.00	0.109	-0.205	0.021
1	8	1	0.2	0.5	0.5	0.30	0.30	0.30	0.50	0.50	0.25	0.089	-0.227	0.020
1	8	1	0.2	0.5	0.5	0.30	0.30	0.30	0.50	0.50	0.50	0.086	-0.218	0.027
1	8	1	0.2	0.8	0.5	0.00	0.00	0.00	0.00	0.00	0.00	0.059	-0.215	0.027
1	8	1	0.2	0.8	0.5	0.15	0.15	0.15	0.00	0.00	0.00	0.076	-0.284	0.015
1	8	1	0.2	0.8	0.5	0.15	0.15	0.15	0.00	0.00	0.25	0.069	-0.280	0.026
1	8	1	0.2	0.8	0.5	0.15	0.15	0.15	0.00	0.00	0.50	0.053	-0.300	0.022
1	8	1	0.2	0.8	0.5	0.15	0.15	0.15	0.00	0.25	0.00	0.067	-0.295	0.028
1	8	1	0.2	0.8	0.5	0.15	0.15	0.15	0.00	0.25	0.25	0.059	-0.311	0.026
1	8	1	0.2	0.8	0.5	0.15	0.15	0.15	0.00	0.25	0.50	0.059	-0.303	0.028
1	8	1	0.2	0.8	0.5	0.15	0.15	0.15	0.00	0.50	0.00	0.070	-0.300	0.026
1	8	1	0.2	0.8	0.5	0.15	0.15	0.15	0.00	0.50	0.25	0.067	-0.299	0.025
1	8	1	0.2	0.8	0.5	0.15	0.15	0.15	0.00	0.50	0.50	0.067	-0.301	0.021
1	8	1	0.2	0.8	0.5	0.15	0.15	0.15	0.25	0.00	0.00	0.073	-0.289	0.031
1	8	1	0.2	0.8	0.5	0.15	0.15	0.15	0.25	0.00	0.25	0.067	-0.287	0.028
1	8	1	0.2	0.8	0.5	0.15	0.15	0.15	0.25	0.00	0.50	0.070	-0.272	0.017
1	8	1	0.2	0.8	0.5	0.15	0.15	0.15	0.25	0.25	0.00	0.069	-0.282	0.030
1	8	1	0.2	0.8	0.5	0.15	0.15	0.15	0.25	0.25	0.25	0.061	-0.299	0.026
1	8	1	0.2	0.8	0.5	0.15	0.15	0.15	0.25	0.25	0.50	0.064	-0.299	0.025
1	8	1	0.2	0.8	0.5	0.15	0.15	0.15	0.25	0.50	0.00	0.074	-0.298	0.032
1	8	1	0.2	0.8	0.5	0.15	0.15	0.15	0.25	0.50	0.25	0.073	-0.276	0.034
1	8	1	0.2	0.8	0.5	0.15	0.15	0.15	0.25	0.50	0.50	0.064	-0.267	0.025
1	8	1	0.2	0.8	0.5	0.15	0.15	0.15	0.50	0.00	0.00	0.068	-0.283	0.029
1	8	1	0.2	0.8	0.5	0.15	0.15	0.15	0.50	0.00	0.25	0.085	-0.280	0.040
1	8	1	0.2	0.8	0.5	0.15	0.15	0.15	0.50	0.00	0.50	0.082	-0.279	0.036
1	8	1	0.2	0.8	0.5	0.15	0.15	0.15	0.50	0.25	0.00	0.062	-0.285	0.021
1	8	1	0.2	0.8	0.5	0.15	0.15	0.15	0.50	0.25	0.25	0.083	-0.275	0.029
1	8	1	0.2	0.8	0.5	0.15	0.15	0.15	0.50	0.25	0.50	0.078	-0.275	0.023
1	8	1	0.2	0.8	0.5	0.15	0.15	0.15	0.50	0.50	0.00	0.092	-0.280	0.032
1	8	1	0.2	0.8	0.5	0.15	0.15	0.15	0.50	0.50	0.25	0.072	-0.276	0.028
1	8	1	0.2	0.8	0.5	0.15	0.15	0.15	0.50	0.50	0.50	0.076	-0.303	0.024
1	8	1	0.2	0.8	0.5	0.30	0.30	0.30	0.00	0.00	0.00	0.102	-0.434	0.015

(continued)

												Mean Bias		
N	m_1	$\frac{m_2}{m_1}$	$E(C)$	$E(R)$	$E(U)$	σ_C	σ_R	σ_U	ρ_{CR}	ρ_{CU}	ρ_{RU}	c	r	u
1	8	1	0.2	0.8	0.5	0.30	0.30	0.30	0.00	0.00	0.25	0.104	-0.429	0.028
1	8	1	0.2	0.8	0.5	0.30	0.30	0.30	0.00	0.00	0.50	0.103	-0.435	0.029
1	8	1	0.2	0.8	0.5	0.30	0.30	0.30	0.00	0.25	0.00	0.106	-0.453	0.015
1	8	1	0.2	0.8	0.5	0.30	0.30	0.30	0.00	0.25	0.25	0.089	-0.452	0.003
1	8	1	0.2	0.8	0.5	0.30	0.30	0.30	0.00	0.25	0.50	0.081	-0.461	0.026
1	8	1	0.2	0.8	0.5	0.30	0.30	0.30	0.00	0.50	0.00	0.086	-0.477	0.005
1	8	1	0.2	0.8	0.5	0.30	0.30	0.30	0.00	0.50	0.25	0.101	-0.450	0.022
1	8	1	0.2	0.8	0.5	0.30	0.30	0.30	0.00	0.50	0.50	0.119	-0.435	0.034
1	8	1	0.2	0.8	0.5	0.30	0.30	0.30	0.25	0.00	0.00	0.093	-0.440	0.016
1	8	1	0.2	0.8	0.5	0.30	0.30	0.30	0.25	0.00	0.25	0.096	-0.407	0.025
1	8	1	0.2	0.8	0.5	0.30	0.30	0.30	0.25	0.00	0.50	0.074	-0.437	0.030
1	8	1	0.2	0.8	0.5	0.30	0.30	0.30	0.25	0.25	0.00	0.117	-0.444	0.017
1	8	1	0.2	0.8	0.5	0.30	0.30	0.30	0.25	0.25	0.25	0.102	-0.436	0.008
1	8	1	0.2	0.8	0.5	0.30	0.30	0.30	0.25	0.25	0.50	0.104	-0.429	0.042
1	8	1	0.2	0.8	0.5	0.30	0.30	0.30	0.25	0.50	0.00	0.101	-0.461	0.009
1	8	1	0.2	0.8	0.5	0.30	0.30	0.30	0.25	0.50	0.25	0.104	-0.449	0.005
1	8	1	0.2	0.8	0.5	0.30	0.30	0.30	0.25	0.50	0.50	0.112	-0.434	0.022
1	8	1	0.2	0.8	0.5	0.30	0.30	0.30	0.50	0.00	0.00	0.103	-0.415	0.025
1	8	1	0.2	0.8	0.5	0.30	0.30	0.30	0.50	0.00	0.25	0.085	-0.435	0.012
1	8	1	0.2	0.8	0.5	0.30	0.30	0.30	0.50	0.00	0.50	0.101	-0.406	0.012
1	8	1	0.2	0.8	0.5	0.30	0.30	0.30	0.50	0.25	0.00	0.104	-0.399	0.011
1	8	1	0.2	0.8	0.5	0.30	0.30	0.30	0.50	0.25	0.25	0.087	-0.435	0.017
1	8	1	0.2	0.8	0.5	0.30	0.30	0.30	0.50	0.25	0.50	0.116	-0.397	0.016
1	8	1	0.2	0.8	0.5	0.30	0.30	0.30	0.50	0.50	0.00	0.104	-0.433	0.025
1	8	1	0.2	0.8	0.5	0.30	0.30	0.30	0.50	0.50	0.25	0.107	-0.408	0.029
1	8	1	0.2	0.8	0.5	0.30	0.30	0.30	0.50	0.50	0.50	0.116	-0.397	0.022
1	8	1	0.5	0.2	0.5	0.00	0.00	0.00	0.00	0.00	0.00	-0.027	0.051	-0.002
1	8	1	0.5	0.2	0.5	0.15	0.15	0.15	0.00	0.00	0.00	-0.017	0.039	0.004
1	8	1	0.5	0.2	0.5	0.15	0.15	0.15	0.00	0.00	0.25	-0.034	0.026	0.000
1	8	1	0.5	0.2	0.5	0.15	0.15	0.15	0.00	0.00	0.50	-0.025	0.024	0.018
1	8	1	0.5	0.2	0.5	0.15	0.15	0.15	0.00	0.25	0.00	-0.011	0.028	0.015
1	8	1	0.5	0.2	0.5	0.15	0.15	0.15	0.00	0.25	0.25	-0.013	0.031	0.004
1	8	1	0.5	0.2	0.5	0.15	0.15	0.15	0.00	0.25	0.50	-0.029	0.025	0.008
1	8	1	0.5	0.2	0.5	0.15	0.15	0.15	0.00	0.50	0.00	-0.013	0.015	0.009
1	8	1	0.5	0.2	0.5	0.15	0.15	0.15	0.00	0.50	0.25	-0.009	0.014	0.001
1	8	1	0.5	0.2	0.5	0.15	0.15	0.15	0.00	0.50	0.50	-0.019	0.030	-0.003
1	8	1	0.5	0.2	0.5	0.15	0.15	0.15	0.25	0.00	0.00	-0.018	0.054	0.000
1	8	1	0.5	0.2	0.5	0.15	0.15	0.15	0.25	0.00	0.25	-0.017	0.020	0.003
1	8	1	0.5	0.2	0.5	0.15	0.15	0.15	0.25	0.00	0.50	-0.023	0.030	0.001

(continued)

												Mean Bias		
N	m_1	$\frac{m_2}{m_1}$	$E(C)$	$E(R)$	$E(U)$	σ_C	σ_R	σ_U	ρ_{CR}	ρ_{CU}	ρ_{RU}	c	r	u
1	8	1	0.5	0.2	0.5	0.15	0.15	0.15	0.25	0.25	0.00	-0.011	0.021	0.005
1	8	1	0.5	0.2	0.5	0.15	0.15	0.15	0.25	0.25	0.25	-0.013	0.040	0.015
1	8	1	0.5	0.2	0.5	0.15	0.15	0.15	0.25	0.25	0.50	-0.027	0.011	-0.004
1	8	1	0.5	0.2	0.5	0.15	0.15	0.15	0.25	0.50	0.00	-0.019	0.031	0.009
1	8	1	0.5	0.2	0.5	0.15	0.15	0.15	0.25	0.50	0.25	-0.017	0.039	0.014
1	8	1	0.5	0.2	0.5	0.15	0.15	0.15	0.25	0.50	0.50	-0.017	0.031	0.007
1	8	1	0.5	0.2	0.5	0.15	0.15	0.15	0.50	0.00	0.00	-0.017	0.030	0.011
1	8	1	0.5	0.2	0.5	0.15	0.15	0.15	0.50	0.00	0.25	-0.005	0.019	0.004
1	8	1	0.5	0.2	0.5	0.15	0.15	0.15	0.50	0.00	0.50	-0.012	0.028	0.006
1	8	1	0.5	0.2	0.5	0.15	0.15	0.15	0.50	0.25	0.00	0.000	0.022	0.001
1	8	1	0.5	0.2	0.5	0.15	0.15	0.15	0.50	0.25	0.25	-0.009	0.044	-0.007
1	8	1	0.5	0.2	0.5	0.15	0.15	0.15	0.50	0.25	0.50	-0.012	0.024	-0.001
1	8	1	0.5	0.2	0.5	0.15	0.15	0.15	0.50	0.50	0.00	0.003	0.036	0.008
1	8	1	0.5	0.2	0.5	0.15	0.15	0.15	0.50	0.50	0.25	0.003	0.037	-0.003
1	8	1	0.5	0.2	0.5	0.15	0.15	0.15	0.50	0.50	0.50	-0.016	0.020	0.007
1	8	1	0.5	0.2	0.5	0.30	0.30	0.30	0.00	0.00	0.00	-0.007	-0.005	-0.011
1	8	1	0.5	0.2	0.5	0.30	0.30	0.30	0.00	0.00	0.25	0.024	-0.002	0.019
1	8	1	0.5	0.2	0.5	0.30	0.30	0.30	0.00	0.00	0.50	-0.009	0.002	0.012
1	8	1	0.5	0.2	0.5	0.30	0.30	0.30	0.00	0.25	0.00	0.012	-0.013	0.011
1	8	1	0.5	0.2	0.5	0.30	0.30	0.30	0.00	0.25	0.25	0.022	-0.021	0.005
1	8	1	0.5	0.2	0.5	0.30	0.30	0.30	0.00	0.25	0.50	0.005	-0.018	0.000
1	8	1	0.5	0.2	0.5	0.30	0.30	0.30	0.00	0.50	0.00	0.024	-0.022	0.024
1	8	1	0.5	0.2	0.5	0.30	0.30	0.30	0.00	0.50	0.25	0.031	-0.002	0.012
1	8	1	0.5	0.2	0.5	0.30	0.30	0.30	0.00	0.50	0.50	0.011	-0.013	0.009
1	8	1	0.5	0.2	0.5	0.30	0.30	0.30	0.25	0.00	0.00	0.020	0.001	0.028
1	8	1	0.5	0.2	0.5	0.30	0.30	0.30	0.25	0.00	0.25	0.017	0.014	0.002
1	8	1	0.5	0.2	0.5	0.30	0.30	0.30	0.25	0.00	0.50	0.002	0.022	0.015
1	8	1	0.5	0.2	0.5	0.30	0.30	0.30	0.25	0.25	0.00	0.025	-0.004	0.014
1	8	1	0.5	0.2	0.5	0.30	0.30	0.30	0.25	0.25	0.25	0.020	-0.007	-0.002
1	8	1	0.5	0.2	0.5	0.30	0.30	0.30	0.25	0.25	0.50	0.021	-0.020	-0.010
1	8	1	0.5	0.2	0.5	0.30	0.30	0.30	0.25	0.50	0.00	0.039	-0.006	0.014
1	8	1	0.5	0.2	0.5	0.30	0.30	0.30	0.25	0.50	0.25	0.040	0.000	0.040
1	8	1	0.5	0.2	0.5	0.30	0.30	0.30	0.25	0.50	0.50	0.025	-0.008	0.005
1	8	1	0.5	0.2	0.5	0.30	0.30	0.30	0.50	0.00	0.00	0.010	0.009	-0.002
1	8	1	0.5	0.2	0.5	0.30	0.30	0.30	0.50	0.00	0.25	0.015	0.013	0.008
1	8	1	0.5	0.2	0.5	0.30	0.30	0.30	0.50	0.00	0.50	-0.011	0.020	0.013
1	8	1	0.5	0.2	0.5	0.30	0.30	0.30	0.50	0.25	0.00	0.043	0.001	0.009
1	8	1	0.5	0.2	0.5	0.30	0.30	0.30	0.50	0.25	0.25	0.021	0.023	0.013
1	8	1	0.5	0.2	0.5	0.30	0.30	0.30	0.50	0.25	0.50	0.011	0.019	0.007

(continued)

N	m_1	$\frac{m_2}{m_1}$	$E(C)$	$E(R)$	$E(U)$	σ_C	σ_R	σ_U	ρ_{CR}	ρ_{CU}	ρ_{RU}	Mean Bias c	Mean Bias r	Mean Bias u
1	8	1	0.5	0.2	0.5	0.30	0.30	0.30	0.50	0.50	0.00	0.018	-0.001	0.013
1	8	1	0.5	0.2	0.5	0.30	0.30	0.30	0.50	0.50	0.25	0.033	0.031	0.005
1	8	1	0.5	0.2	0.5	0.30	0.30	0.30	0.50	0.50	0.50	0.028	0.006	0.002
1	8	1	0.5	0.5	0.5	0.00	0.00	0.00	0.00	0.00	0.00	-0.021	0.056	0.002
1	8	1	0.5	0.5	0.5	0.15	0.15	0.15	0.00	0.00	0.00	0.003	0.016	0.013
1	8	1	0.5	0.5	0.5	0.15	0.15	0.15	0.00	0.00	0.25	0.002	0.027	0.003
1	8	1	0.5	0.5	0.5	0.15	0.15	0.15	0.00	0.00	0.50	0.007	0.019	0.006
1	8	1	0.5	0.5	0.5	0.15	0.15	0.15	0.00	0.25	0.00	0.012	0.025	0.009
1	8	1	0.5	0.5	0.5	0.15	0.15	0.15	0.00	0.25	0.25	0.006	0.003	0.011
1	8	1	0.5	0.5	0.5	0.15	0.15	0.15	0.00	0.25	0.50	0.002	0.012	0.014
1	8	1	0.5	0.5	0.5	0.15	0.15	0.15	0.00	0.50	0.00	0.012	0.044	0.010
1	8	1	0.5	0.5	0.5	0.15	0.15	0.15	0.00	0.50	0.25	0.011	0.004	0.006
1	8	1	0.5	0.5	0.5	0.15	0.15	0.15	0.00	0.50	0.50	0.016	0.010	0.009
1	8	1	0.5	0.5	0.5	0.15	0.15	0.15	0.25	0.00	0.00	-0.013	0.016	0.013
1	8	1	0.5	0.5	0.5	0.15	0.15	0.15	0.25	0.00	0.25	0.000	0.052	0.013
1	8	1	0.5	0.5	0.5	0.15	0.15	0.15	0.25	0.00	0.50	0.003	0.049	0.018
1	8	1	0.5	0.5	0.5	0.15	0.15	0.15	0.25	0.25	0.00	0.005	0.017	0.002
1	8	1	0.5	0.5	0.5	0.15	0.15	0.15	0.25	0.25	0.25	0.006	0.020	0.014
1	8	1	0.5	0.5	0.5	0.15	0.15	0.15	0.25	0.25	0.50	0.000	0.029	0.006
1	8	1	0.5	0.5	0.5	0.15	0.15	0.15	0.25	0.50	0.00	0.029	0.016	0.012
1	8	1	0.5	0.5	0.5	0.15	0.15	0.15	0.25	0.50	0.25	0.011	0.021	0.016
1	8	1	0.5	0.5	0.5	0.15	0.15	0.15	0.25	0.50	0.50	0.006	0.038	0.001
1	8	1	0.5	0.5	0.5	0.15	0.15	0.15	0.50	0.00	0.00	-0.005	0.019	0.008
1	8	1	0.5	0.5	0.5	0.15	0.15	0.15	0.50	0.00	0.25	0.002	0.004	0.010
1	8	1	0.5	0.5	0.5	0.15	0.15	0.15	0.50	0.00	0.50	-0.003	0.034	0.004
1	8	1	0.5	0.5	0.5	0.15	0.15	0.15	0.50	0.25	0.00	0.024	0.027	0.005
1	8	1	0.5	0.5	0.5	0.15	0.15	0.15	0.50	0.25	0.25	0.006	0.010	-0.009
1	8	1	0.5	0.5	0.5	0.15	0.15	0.15	0.50	0.25	0.50	-0.003	0.031	0.018
1	8	1	0.5	0.5	0.5	0.15	0.15	0.15	0.50	0.50	0.00	0.003	0.028	0.010
1	8	1	0.5	0.5	0.5	0.15	0.15	0.15	0.50	0.50	0.25	0.003	0.029	0.014
1	8	1	0.5	0.5	0.5	0.15	0.15	0.15	0.50	0.50	0.50	0.016	0.014	0.013
1	8	1	0.5	0.5	0.5	0.30	0.30	0.30	0.00	0.00	0.00	0.045	-0.049	0.006
1	8	1	0.5	0.5	0.5	0.30	0.30	0.30	0.00	0.00	0.25	0.012	-0.072	-0.011
1	8	1	0.5	0.5	0.5	0.30	0.30	0.30	0.00	0.00	0.50	0.033	-0.042	0.023
1	8	1	0.5	0.5	0.5	0.30	0.30	0.30	0.00	0.25	0.00	0.045	-0.079	-0.020
1	8	1	0.5	0.5	0.5	0.30	0.30	0.30	0.00	0.25	0.25	0.033	-0.053	0.020
1	8	1	0.5	0.5	0.5	0.30	0.30	0.30	0.00	0.25	0.50	0.015	-0.051	0.018
1	8	1	0.5	0.5	0.5	0.30	0.30	0.30	0.00	0.50	0.00	0.048	-0.059	0.013
1	8	1	0.5	0.5	0.5	0.30	0.30	0.30	0.00	0.50	0.25	0.070	-0.066	0.030

(continued)

												Mean Bias		
N	m_1	$\frac{m_2}{m_1}$	$E(C)$	$E(R)$	$E(U)$	σ_C	σ_R	σ_U	ρ_{CR}	ρ_{CU}	ρ_{RU}	c	r	u
1	8	1	0.5	0.5	0.5	0.30	0.30	0.30	0.00	0.50	0.50	0.040	-0.073	0.009
1	8	1	0.5	0.5	0.5	0.30	0.30	0.30	0.25	0.00	0.00	0.021	-0.036	0.007
1	8	1	0.5	0.5	0.5	0.30	0.30	0.30	0.25	0.00	0.25	0.018	-0.028	0.010
1	8	1	0.5	0.5	0.5	0.30	0.30	0.30	0.25	0.00	0.50	0.040	-0.032	0.012
1	8	1	0.5	0.5	0.5	0.30	0.30	0.30	0.25	0.25	0.00	0.044	-0.053	0.017
1	8	1	0.5	0.5	0.5	0.30	0.30	0.30	0.25	0.25	0.25	0.020	-0.030	0.017
1	8	1	0.5	0.5	0.5	0.30	0.30	0.30	0.25	0.25	0.50	0.046	-0.048	-0.002
1	8	1	0.5	0.5	0.5	0.30	0.30	0.30	0.25	0.50	0.00	0.052	-0.055	-0.007
1	8	1	0.5	0.5	0.5	0.30	0.30	0.30	0.25	0.50	0.25	0.044	-0.064	0.010
1	8	1	0.5	0.5	0.5	0.30	0.30	0.30	0.25	0.50	0.50	0.049	-0.055	0.023
1	8	1	0.5	0.5	0.5	0.30	0.30	0.30	0.50	0.00	0.00	0.044	-0.018	0.003
1	8	1	0.5	0.5	0.5	0.30	0.30	0.30	0.50	0.00	0.25	-0.005	-0.042	-0.001
1	8	1	0.5	0.5	0.5	0.30	0.30	0.30	0.50	0.00	0.50	0.024	-0.038	0.008
1	8	1	0.5	0.5	0.5	0.30	0.30	0.30	0.50	0.25	0.00	0.047	-0.014	0.031
1	8	1	0.5	0.5	0.5	0.30	0.30	0.30	0.50	0.25	0.25	0.036	-0.055	-0.007
1	8	1	0.5	0.5	0.5	0.30	0.30	0.30	0.50	0.25	0.50	0.033	-0.033	0.009
1	8	1	0.5	0.5	0.5	0.30	0.30	0.30	0.50	0.50	0.00	0.057	-0.051	0.008
1	8	1	0.5	0.5	0.5	0.30	0.30	0.30	0.50	0.50	0.25	0.035	-0.030	0.009
1	8	1	0.5	0.5	0.5	0.30	0.30	0.30	0.50	0.50	0.50	0.047	-0.038	0.014
1	8	1	0.5	0.8	0.5	0.00	0.00	0.00	0.00	0.00	0.00	0.024	-0.022	0.018
1	8	1	0.5	0.8	0.5	0.15	0.15	0.15	0.00	0.00	0.00	0.028	-0.046	0.024
1	8	1	0.5	0.8	0.5	0.15	0.15	0.15	0.00	0.00	0.25	0.040	-0.048	0.021
1	8	1	0.5	0.8	0.5	0.15	0.15	0.15	0.00	0.00	0.50	0.035	-0.043	0.019
1	8	1	0.5	0.8	0.5	0.15	0.15	0.15	0.00	0.25	0.00	0.043	-0.047	0.023
1	8	1	0.5	0.8	0.5	0.15	0.15	0.15	0.00	0.25	0.25	0.033	-0.047	0.019
1	8	1	0.5	0.8	0.5	0.15	0.15	0.15	0.00	0.25	0.50	0.018	-0.046	0.014
1	8	1	0.5	0.8	0.5	0.15	0.15	0.15	0.00	0.50	0.00	0.035	-0.065	0.009
1	8	1	0.5	0.8	0.5	0.15	0.15	0.15	0.00	0.50	0.25	0.036	-0.051	0.014
1	8	1	0.5	0.8	0.5	0.15	0.15	0.15	0.00	0.50	0.50	0.054	-0.043	0.019
1	8	1	0.5	0.8	0.5	0.15	0.15	0.15	0.25	0.00	0.00	0.014	-0.042	0.011
1	8	1	0.5	0.8	0.5	0.15	0.15	0.15	0.25	0.00	0.25	0.042	-0.056	0.012
1	8	1	0.5	0.8	0.5	0.15	0.15	0.15	0.25	0.00	0.50	0.016	-0.033	0.012
1	8	1	0.5	0.8	0.5	0.15	0.15	0.15	0.25	0.25	0.00	0.026	-0.040	0.012
1	8	1	0.5	0.8	0.5	0.15	0.15	0.15	0.25	0.25	0.25	0.034	-0.034	0.017
1	8	1	0.5	0.8	0.5	0.15	0.15	0.15	0.25	0.25	0.50	0.033	-0.048	0.022
1	8	1	0.5	0.8	0.5	0.15	0.15	0.15	0.25	0.50	0.00	0.043	-0.059	0.016
1	8	1	0.5	0.8	0.5	0.15	0.15	0.15	0.25	0.50	0.25	0.048	-0.024	0.029
1	8	1	0.5	0.8	0.5	0.15	0.15	0.15	0.25	0.50	0.50	0.033	-0.048	0.019
1	8	1	0.5	0.8	0.5	0.15	0.15	0.15	0.50	0.00	0.00	0.020	-0.026	0.017

(continued)

												Mean Bias		
N	m_1	$\frac{m_2}{m_1}$	$E(C)$	$E(R)$	$E(U)$	σ_C	σ_R	σ_U	ρ_{CR}	ρ_{CU}	ρ_{RU}	c	r	u
1	8	1	0.5	0.8	0.5	0.15	0.15	0.15	0.50	0.00	0.25	0.033	-0.025	0.023
1	8	1	0.5	0.8	0.5	0.15	0.15	0.15	0.50	0.00	0.50	0.028	-0.030	0.015
1	8	1	0.5	0.8	0.5	0.15	0.15	0.15	0.50	0.25	0.00	0.034	-0.048	0.021
1	8	1	0.5	0.8	0.5	0.15	0.15	0.15	0.50	0.25	0.25	0.038	-0.033	0.027
1	8	1	0.5	0.8	0.5	0.15	0.15	0.15	0.50	0.25	0.50	0.035	-0.059	0.021
1	8	1	0.5	0.8	0.5	0.15	0.15	0.15	0.50	0.50	0.00	0.040	-0.032	0.007
1	8	1	0.5	0.8	0.5	0.15	0.15	0.15	0.50	0.50	0.25	0.038	-0.049	0.016
1	8	1	0.5	0.8	0.5	0.15	0.15	0.15	0.50	0.50	0.50	0.029	-0.037	0.019
1	8	1	0.5	0.8	0.5	0.30	0.30	0.30	0.00	0.00	0.00	0.073	-0.130	0.008
1	8	1	0.5	0.8	0.5	0.30	0.30	0.30	0.00	0.00	0.25	0.061	-0.135	0.030
1	8	1	0.5	0.8	0.5	0.30	0.30	0.30	0.00	0.00	0.50	0.047	-0.121	0.013
1	8	1	0.5	0.8	0.5	0.30	0.30	0.30	0.00	0.25	0.00	0.062	-0.140	0.016
1	8	1	0.5	0.8	0.5	0.30	0.30	0.30	0.00	0.25	0.25	0.066	-0.136	0.029
1	8	1	0.5	0.8	0.5	0.30	0.30	0.30	0.00	0.25	0.50	0.071	-0.125	0.021
1	8	1	0.5	0.8	0.5	0.30	0.30	0.30	0.00	0.50	0.00	0.092	-0.144	0.021
1	8	1	0.5	0.8	0.5	0.30	0.30	0.30	0.00	0.50	0.25	0.086	-0.136	0.025
1	8	1	0.5	0.8	0.5	0.30	0.30	0.30	0.00	0.50	0.50	0.064	-0.144	0.022
1	8	1	0.5	0.8	0.5	0.30	0.30	0.30	0.25	0.00	0.00	0.065	-0.135	0.007
1	8	1	0.5	0.8	0.5	0.30	0.30	0.30	0.25	0.00	0.25	0.063	-0.147	0.007
1	8	1	0.5	0.8	0.5	0.30	0.30	0.30	0.25	0.00	0.50	0.056	-0.105	0.021
1	8	1	0.5	0.8	0.5	0.30	0.30	0.30	0.25	0.25	0.00	0.059	-0.127	0.028
1	8	1	0.5	0.8	0.5	0.30	0.30	0.30	0.25	0.25	0.25	0.052	-0.127	0.014
1	8	1	0.5	0.8	0.5	0.30	0.30	0.30	0.25	0.25	0.50	0.057	-0.136	0.004
1	8	1	0.5	0.8	0.5	0.30	0.30	0.30	0.25	0.50	0.00	0.072	-0.152	0.002
1	8	1	0.5	0.8	0.5	0.30	0.30	0.30	0.25	0.50	0.25	0.067	-0.152	0.008
1	8	1	0.5	0.8	0.5	0.30	0.30	0.30	0.25	0.50	0.50	0.063	-0.161	-0.004
1	8	1	0.5	0.8	0.5	0.30	0.30	0.30	0.50	0.00	0.00	0.048	-0.127	0.014
1	8	1	0.5	0.8	0.5	0.30	0.30	0.30	0.50	0.00	0.25	0.053	-0.124	0.020
1	8	1	0.5	0.8	0.5	0.30	0.30	0.30	0.50	0.00	0.50	0.070	-0.122	0.023
1	8	1	0.5	0.8	0.5	0.30	0.30	0.30	0.50	0.25	0.00	0.076	-0.117	0.015
1	8	1	0.5	0.8	0.5	0.30	0.30	0.30	0.50	0.25	0.25	0.080	-0.075	0.021
1	8	1	0.5	0.8	0.5	0.30	0.30	0.30	0.50	0.25	0.50	0.068	-0.110	0.012
1	8	1	0.5	0.8	0.5	0.30	0.30	0.30	0.50	0.50	0.00	0.072	-0.114	0.023
1	8	1	0.5	0.8	0.5	0.30	0.30	0.30	0.50	0.50	0.25	0.070	-0.120	0.013
1	8	1	0.5	0.8	0.5	0.30	0.30	0.30	0.50	0.50	0.50	0.066	-0.103	0.014
1	8	1	0.8	0.2	0.5	0.00	0.00	0.00	0.00	0.00	0.00	-0.013	0.011	-0.004
1	8	1	0.8	0.2	0.5	0.15	0.15	0.15	0.00	0.00	0.00	-0.037	0.031	-0.011
1	8	1	0.8	0.2	0.5	0.15	0.15	0.15	0.00	0.00	0.25	-0.031	0.014	-0.007
1	8	1	0.8	0.2	0.5	0.15	0.15	0.15	0.00	0.00	0.50	-0.030	0.028	-0.002

(continued)

												Mean Bias		
N	m_1	$\frac{m_2}{m_1}$	$E(C)$	$E(R)$	$E(U)$	σ_C	σ_R	σ_U	ρ_{CR}	ρ_{CU}	ρ_{RU}	c	r	u
1	8	1	0.8	0.2	0.5	0.15	0.15	0.15	0.00	0.25	0.00	-0.006	0.018	0.001
1	8	1	0.8	0.2	0.5	0.15	0.15	0.15	0.00	0.25	0.25	-0.027	0.016	-0.001
1	8	1	0.8	0.2	0.5	0.15	0.15	0.15	0.00	0.25	0.50	-0.028	0.019	-0.010
1	8	1	0.8	0.2	0.5	0.15	0.15	0.15	0.00	0.50	0.00	-0.018	0.025	0.005
1	8	1	0.8	0.2	0.5	0.15	0.15	0.15	0.00	0.50	0.25	-0.027	0.019	-0.005
1	8	1	0.8	0.2	0.5	0.15	0.15	0.15	0.00	0.50	0.50	-0.012	0.009	-0.011
1	8	1	0.8	0.2	0.5	0.15	0.15	0.15	0.25	0.00	0.00	-0.027	0.024	-0.008
1	8	1	0.8	0.2	0.5	0.15	0.15	0.15	0.25	0.00	0.25	-0.026	0.019	-0.003
1	8	1	0.8	0.2	0.5	0.15	0.15	0.15	0.25	0.00	0.50	-0.018	0.018	-0.010
1	8	1	0.8	0.2	0.5	0.15	0.15	0.15	0.25	0.25	0.00	-0.031	0.022	-0.005
1	8	1	0.8	0.2	0.5	0.15	0.15	0.15	0.25	0.25	0.25	-0.027	0.029	0.007
1	8	1	0.8	0.2	0.5	0.15	0.15	0.15	0.25	0.25	0.50	-0.031	0.016	-0.004
1	8	1	0.8	0.2	0.5	0.15	0.15	0.15	0.25	0.50	0.00	-0.010	0.012	0.008
1	8	1	0.8	0.2	0.5	0.15	0.15	0.15	0.25	0.50	0.25	-0.016	0.013	0.006
1	8	1	0.8	0.2	0.5	0.15	0.15	0.15	0.25	0.50	0.50	-0.020	0.021	-0.001
1	8	1	0.8	0.2	0.5	0.15	0.15	0.15	0.50	0.00	0.00	-0.022	0.023	-0.003
1	8	1	0.8	0.2	0.5	0.15	0.15	0.15	0.50	0.00	0.25	-0.021	0.014	-0.001
1	8	1	0.8	0.2	0.5	0.15	0.15	0.15	0.50	0.00	0.50	-0.026	0.014	-0.002
1	8	1	0.8	0.2	0.5	0.15	0.15	0.15	0.50	0.25	0.00	-0.013	0.004	-0.010
1	8	1	0.8	0.2	0.5	0.15	0.15	0.15	0.50	0.25	0.25	-0.022	0.020	0.006
1	8	1	0.8	0.2	0.5	0.15	0.15	0.15	0.50	0.25	0.50	-0.018	0.013	-0.004
1	8	1	0.8	0.2	0.5	0.15	0.15	0.15	0.50	0.50	0.00	0.000	0.018	0.018
1	8	1	0.8	0.2	0.5	0.15	0.15	0.15	0.50	0.50	0.25	-0.022	0.012	0.005
1	8	1	0.8	0.2	0.5	0.15	0.15	0.15	0.50	0.50	0.50	-0.028	0.010	-0.007
1	8	1	0.8	0.2	0.5	0.30	0.30	0.30	0.00	0.00	0.00	-0.025	0.011	-0.001
1	8	1	0.8	0.2	0.5	0.30	0.30	0.30	0.00	0.00	0.25	-0.023	-0.011	0.007
1	8	1	0.8	0.2	0.5	0.30	0.30	0.30	0.00	0.00	0.50	-0.033	0.001	0.023
1	8	1	0.8	0.2	0.5	0.30	0.30	0.30	0.00	0.25	0.00	-0.005	0.018	0.007
1	8	1	0.8	0.2	0.5	0.30	0.30	0.30	0.00	0.25	0.25	-0.036	0.016	-0.003
1	8	1	0.8	0.2	0.5	0.30	0.30	0.30	0.00	0.25	0.50	-0.022	-0.007	0.025
1	8	1	0.8	0.2	0.5	0.30	0.30	0.30	0.00	0.50	0.00	-0.018	0.005	0.002
1	8	1	0.8	0.2	0.5	0.30	0.30	0.30	0.00	0.50	0.25	-0.016	-0.007	-0.003
1	8	1	0.8	0.2	0.5	0.30	0.30	0.30	0.00	0.50	0.50	-0.023	0.001	0.005
1	8	1	0.8	0.2	0.5	0.30	0.30	0.30	0.25	0.00	0.00	-0.031	0.012	-0.001
1	8	1	0.8	0.2	0.5	0.30	0.30	0.30	0.25	0.00	0.25	-0.038	0.020	0.006
1	8	1	0.8	0.2	0.5	0.30	0.30	0.30	0.25	0.00	0.50	-0.030	0.018	0.002
1	8	1	0.8	0.2	0.5	0.30	0.30	0.30	0.25	0.25	0.00	-0.008	-0.003	0.011
1	8	1	0.8	0.2	0.5	0.30	0.30	0.30	0.25	0.25	0.25	-0.032	0.003	0.012
1	8	1	0.8	0.2	0.5	0.30	0.30	0.30	0.25	0.25	0.50	-0.019	0.033	-0.011

(continued)

												Mean Bias		
N	m_1	$\frac{m_2}{m_1}$	$E(C)$	$E(R)$	$E(U)$	σ_C	σ_R	σ_U	ρ_{CR}	ρ_{CU}	ρ_{RU}	c	r	u
1	8	1	0.8	0.2	0.5	0.30	0.30	0.30	0.25	0.50	0.00	-0.005	0.022	0.005
1	8	1	0.8	0.2	0.5	0.30	0.30	0.30	0.25	0.50	0.25	-0.017	0.019	0.002
1	8	1	0.8	0.2	0.5	0.30	0.30	0.30	0.25	0.50	0.50	-0.028	0.023	0.003
1	8	1	0.8	0.2	0.5	0.30	0.30	0.30	0.50	0.00	0.00	-0.020	0.006	0.005
1	8	1	0.8	0.2	0.5	0.30	0.30	0.30	0.50	0.00	0.25	-0.046	0.007	0.004
1	8	1	0.8	0.2	0.5	0.30	0.30	0.30	0.50	0.00	0.50	-0.020	0.034	0.032
1	8	1	0.8	0.2	0.5	0.30	0.30	0.30	0.50	0.25	0.00	-0.024	0.015	0.009
1	8	1	0.8	0.2	0.5	0.30	0.30	0.30	0.50	0.25	0.25	-0.012	0.020	-0.001
1	8	1	0.8	0.2	0.5	0.30	0.30	0.30	0.50	0.25	0.50	-0.024	0.022	0.005
1	8	1	0.8	0.2	0.5	0.30	0.30	0.30	0.50	0.50	0.00	-0.009	0.000	-0.003
1	8	1	0.8	0.2	0.5	0.30	0.30	0.30	0.50	0.50	0.25	-0.006	0.023	0.004
1	8	1	0.8	0.2	0.5	0.30	0.30	0.30	0.50	0.50	0.50	-0.027	0.031	-0.004
1	8	1	0.8	0.5	0.5	0.00	0.00	0.00	0.00	0.00	0.00	-0.019	0.022	0.002
1	8	1	0.8	0.5	0.5	0.15	0.15	0.15	0.00	0.00	0.00	-0.005	0.027	0.007
1	8	1	0.8	0.5	0.5	0.15	0.15	0.15	0.00	0.00	0.25	-0.016	-0.001	-0.010
1	8	1	0.8	0.5	0.5	0.15	0.15	0.15	0.00	0.00	0.50	-0.003	0.013	0.007
1	8	1	0.8	0.5	0.5	0.15	0.15	0.15	0.00	0.25	0.00	-0.017	0.016	-0.006
1	8	1	0.8	0.5	0.5	0.15	0.15	0.15	0.00	0.25	0.25	-0.017	0.017	0.010
1	8	1	0.8	0.5	0.5	0.15	0.15	0.15	0.00	0.25	0.50	-0.015	0.034	0.004
1	8	1	0.8	0.5	0.5	0.15	0.15	0.15	0.00	0.50	0.00	0.002	0.028	0.004
1	8	1	0.8	0.5	0.5	0.15	0.15	0.15	0.00	0.50	0.25	-0.018	0.028	-0.001
1	8	1	0.8	0.5	0.5	0.15	0.15	0.15	0.00	0.50	0.50	-0.014	0.025	-0.004
1	8	1	0.8	0.5	0.5	0.15	0.15	0.15	0.25	0.00	0.00	0.010	0.006	-0.012
1	8	1	0.8	0.5	0.5	0.15	0.15	0.15	0.25	0.00	0.25	-0.010	0.029	0.006
1	8	1	0.8	0.5	0.5	0.15	0.15	0.15	0.25	0.00	0.50	-0.001	0.018	0.011
1	8	1	0.8	0.5	0.5	0.15	0.15	0.15	0.25	0.25	0.00	-0.007	0.012	0.006
1	8	1	0.8	0.5	0.5	0.15	0.15	0.15	0.25	0.25	0.25	-0.016	0.020	-0.012
1	8	1	0.8	0.5	0.5	0.15	0.15	0.15	0.25	0.25	0.50	-0.014	0.025	0.011
1	8	1	0.8	0.5	0.5	0.15	0.15	0.15	0.25	0.50	0.00	-0.011	0.022	0.001
1	8	1	0.8	0.5	0.5	0.15	0.15	0.15	0.25	0.50	0.25	-0.012	0.019	-0.005
1	8	1	0.8	0.5	0.5	0.15	0.15	0.15	0.25	0.50	0.50	0.006	0.035	0.002
1	8	1	0.8	0.5	0.5	0.15	0.15	0.15	0.50	0.00	0.00	0.001	0.034	0.008
1	8	1	0.8	0.5	0.5	0.15	0.15	0.15	0.50	0.00	0.25	-0.012	0.021	0.004
1	8	1	0.8	0.5	0.5	0.15	0.15	0.15	0.50	0.00	0.50	-0.017	0.013	-0.014
1	8	1	0.8	0.5	0.5	0.15	0.15	0.15	0.50	0.25	0.00	0.012	0.031	0.006
1	8	1	0.8	0.5	0.5	0.15	0.15	0.15	0.50	0.25	0.25	-0.014	0.026	0.015
1	8	1	0.8	0.5	0.5	0.15	0.15	0.15	0.50	0.25	0.50	-0.015	0.015	0.007
1	8	1	0.8	0.5	0.5	0.15	0.15	0.15	0.50	0.50	0.00	-0.004	0.026	0.008
1	8	1	0.8	0.5	0.5	0.15	0.15	0.15	0.50	0.50	0.25	-0.003	0.031	0.013

(continued)

												Mean Bias		
N	m_1	$\frac{m_2}{m_1}$	$E(C)$	$E(R)$	$E(U)$	σ_C	σ_R	σ_U	ρ_{CR}	ρ_{CU}	ρ_{RU}	c	r	u
1	8	1	0.8	0.5	0.5	0.15	0.15	0.15	0.50	0.50	0.50	-0.004	0.033	0.001
1	8	1	0.8	0.5	0.5	0.30	0.30	0.30	0.00	0.00	0.00	-0.011	-0.013	0.000
1	8	1	0.8	0.5	0.5	0.30	0.30	0.30	0.00	0.00	0.25	0.010	-0.025	0.006
1	8	1	0.8	0.5	0.5	0.30	0.30	0.30	0.00	0.00	0.50	-0.005	-0.014	0.017
1	8	1	0.8	0.5	0.5	0.30	0.30	0.30	0.00	0.25	0.00	0.003	-0.018	-0.002
1	8	1	0.8	0.5	0.5	0.30	0.30	0.30	0.00	0.25	0.25	-0.007	-0.023	-0.004
1	8	1	0.8	0.5	0.5	0.30	0.30	0.30	0.00	0.25	0.50	0.005	-0.016	0.015
1	8	1	0.8	0.5	0.5	0.30	0.30	0.30	0.00	0.50	0.00	0.013	-0.043	-0.003
1	8	1	0.8	0.5	0.5	0.30	0.30	0.30	0.00	0.50	0.25	0.014	-0.050	0.006
1	8	1	0.8	0.5	0.5	0.30	0.30	0.30	0.00	0.50	0.50	0.029	-0.010	0.019
1	8	1	0.8	0.5	0.5	0.30	0.30	0.30	0.25	0.00	0.00	0.004	0.003	0.002
1	8	1	0.8	0.5	0.5	0.30	0.30	0.30	0.25	0.00	0.25	-0.003	0.011	-0.010
1	8	1	0.8	0.5	0.5	0.30	0.30	0.30	0.25	0.00	0.50	-0.030	-0.017	-0.004
1	8	1	0.8	0.5	0.5	0.30	0.30	0.30	0.25	0.25	0.00	0.019	-0.003	0.011
1	8	1	0.8	0.5	0.5	0.30	0.30	0.30	0.25	0.25	0.25	-0.003	-0.022	-0.002
1	8	1	0.8	0.5	0.5	0.30	0.30	0.30	0.25	0.25	0.50	-0.018	-0.002	-0.002
1	8	1	0.8	0.5	0.5	0.30	0.30	0.30	0.25	0.50	0.00	0.013	-0.004	0.000
1	8	1	0.8	0.5	0.5	0.30	0.30	0.30	0.25	0.50	0.25	0.014	-0.010	0.006
1	8	1	0.8	0.5	0.5	0.30	0.30	0.30	0.25	0.50	0.50	-0.006	-0.027	-0.006
1	8	1	0.8	0.5	0.5	0.30	0.30	0.30	0.50	0.00	0.00	-0.001	-0.001	0.031
1	8	1	0.8	0.5	0.5	0.30	0.30	0.30	0.50	0.00	0.25	0.001	0.025	0.008
1	8	1	0.8	0.5	0.5	0.30	0.30	0.30	0.50	0.00	0.50	-0.038	0.010	-0.018
1	8	1	0.8	0.5	0.5	0.30	0.30	0.30	0.50	0.25	0.00	0.003	-0.013	-0.009
1	8	1	0.8	0.5	0.5	0.30	0.30	0.30	0.50	0.25	0.25	0.011	-0.008	0.008
1	8	1	0.8	0.5	0.5	0.30	0.30	0.30	0.50	0.25	0.50	-0.017	-0.007	0.000
1	8	1	0.8	0.5	0.5	0.30	0.30	0.30	0.50	0.50	0.00	0.022	0.010	0.014
1	8	1	0.8	0.5	0.5	0.30	0.30	0.30	0.50	0.50	0.25	0.033	0.018	0.006
1	8	1	0.8	0.5	0.5	0.30	0.30	0.30	0.50	0.50	0.50	0.023	-0.010	0.010
1	8	1	0.8	0.8	0.5	0.00	0.00	0.00	0.00	0.00	0.00	-0.009	0.013	0.001
1	8	1	0.8	0.8	0.5	0.15	0.15	0.15	0.00	0.00	0.00	0.002	-0.016	0.011
1	8	1	0.8	0.8	0.5	0.15	0.15	0.15	0.00	0.00	0.25	0.005	0.003	0.009
1	8	1	0.8	0.8	0.5	0.15	0.15	0.15	0.00	0.00	0.50	0.009	-0.003	0.002
1	8	1	0.8	0.8	0.5	0.15	0.15	0.15	0.00	0.25	0.00	0.011	-0.010	0.008
1	8	1	0.8	0.8	0.5	0.15	0.15	0.15	0.00	0.25	0.25	0.032	-0.014	0.004
1	8	1	0.8	0.8	0.5	0.15	0.15	0.15	0.00	0.25	0.50	0.003	-0.001	0.006
1	8	1	0.8	0.8	0.5	0.15	0.15	0.15	0.00	0.50	0.00	0.016	-0.007	0.010
1	8	1	0.8	0.8	0.5	0.15	0.15	0.15	0.00	0.50	0.25	0.017	0.001	0.015
1	8	1	0.8	0.8	0.5	0.15	0.15	0.15	0.00	0.50	0.50	0.021	0.002	0.009
1	8	1	0.8	0.8	0.5	0.15	0.15	0.15	0.25	0.00	0.00	0.010	0.002	0.010

(continued)

N	m_1	$\frac{m_2}{m_1}$	$E(C)$	$E(R)$	$E(U)$	σ_C	σ_R	σ_U	ρ_{CR}	ρ_{CU}	ρ_{RU}	Mean Bias		
												c	r	u
1	8	1	0.8	0.8	0.5	0.15	0.15	0.15	0.25	0.00	0.25	0.011	-0.002	0.019
1	8	1	0.8	0.8	0.5	0.15	0.15	0.15	0.25	0.00	0.50	0.008	0.004	-0.003
1	8	1	0.8	0.8	0.5	0.15	0.15	0.15	0.25	0.25	0.00	0.014	-0.006	0.015
1	8	1	0.8	0.8	0.5	0.15	0.15	0.15	0.25	0.25	0.25	0.017	0.003	0.014
1	8	1	0.8	0.8	0.5	0.15	0.15	0.15	0.25	0.25	0.50	0.007	-0.009	0.008
1	8	1	0.8	0.8	0.5	0.15	0.15	0.15	0.25	0.50	0.00	0.022	-0.002	0.011
1	8	1	0.8	0.8	0.5	0.15	0.15	0.15	0.25	0.50	0.25	0.006	-0.002	0.008
1	8	1	0.8	0.8	0.5	0.15	0.15	0.15	0.25	0.50	0.50	0.025	-0.013	0.008
1	8	1	0.8	0.8	0.5	0.15	0.15	0.15	0.50	0.00	0.00	0.006	0.001	0.010
1	8	1	0.8	0.8	0.5	0.15	0.15	0.15	0.50	0.00	0.25	0.013	0.007	-0.010
1	8	1	0.8	0.8	0.5	0.15	0.15	0.15	0.50	0.00	0.50	0.009	0.014	0.015
1	8	1	0.8	0.8	0.5	0.15	0.15	0.15	0.50	0.25	0.00	0.006	-0.002	0.008
1	8	1	0.8	0.8	0.5	0.15	0.15	0.15	0.50	0.25	0.25	0.017	0.000	0.021
1	8	1	0.8	0.8	0.5	0.15	0.15	0.15	0.50	0.25	0.50	-0.007	-0.008	0.005
1	8	1	0.8	0.8	0.5	0.15	0.15	0.15	0.50	0.50	0.00	0.012	-0.004	0.008
1	8	1	0.8	0.8	0.5	0.15	0.15	0.15	0.50	0.50	0.25	0.022	0.002	0.007
1	8	1	0.8	0.8	0.5	0.15	0.15	0.15	0.50	0.50	0.50	-0.002	0.009	0.005
1	8	1	0.8	0.8	0.5	0.30	0.30	0.30	0.00	0.00	0.00	0.016	-0.060	0.004
1	8	1	0.8	0.8	0.5	0.30	0.30	0.30	0.00	0.00	0.25	0.006	-0.056	0.014
1	8	1	0.8	0.8	0.5	0.30	0.30	0.30	0.00	0.00	0.50	0.004	-0.056	-0.003
1	8	1	0.8	0.8	0.5	0.30	0.30	0.30	0.00	0.25	0.00	0.016	-0.071	-0.002
1	8	1	0.8	0.8	0.5	0.30	0.30	0.30	0.00	0.25	0.25	0.021	-0.062	0.010
1	8	1	0.8	0.8	0.5	0.30	0.30	0.30	0.00	0.25	0.50	0.019	-0.069	0.017
1	8	1	0.8	0.8	0.5	0.30	0.30	0.30	0.00	0.50	0.00	0.044	-0.088	0.004
1	8	1	0.8	0.8	0.5	0.30	0.30	0.30	0.00	0.50	0.25	0.046	-0.074	0.008
1	8	1	0.8	0.8	0.5	0.30	0.30	0.30	0.00	0.50	0.50	0.032	-0.062	0.017
1	8	1	0.8	0.8	0.5	0.30	0.30	0.30	0.25	0.00	0.00	0.019	-0.038	0.000
1	8	1	0.8	0.8	0.5	0.30	0.30	0.30	0.25	0.00	0.25	0.020	-0.050	-0.009
1	8	1	0.8	0.8	0.5	0.30	0.30	0.30	0.25	0.00	0.50	0.013	-0.045	-0.013
1	8	1	0.8	0.8	0.5	0.30	0.30	0.30	0.25	0.25	0.00	0.042	-0.054	0.015
1	8	1	0.8	0.8	0.5	0.30	0.30	0.30	0.25	0.25	0.25	0.044	-0.045	0.030
1	8	1	0.8	0.8	0.5	0.30	0.30	0.30	0.25	0.25	0.50	0.032	-0.031	0.012
1	8	1	0.8	0.8	0.5	0.30	0.30	0.30	0.25	0.50	0.00	0.048	-0.053	0.025
1	8	1	0.8	0.8	0.5	0.30	0.30	0.30	0.25	0.50	0.25	0.041	-0.056	0.002
1	8	1	0.8	0.8	0.5	0.30	0.30	0.30	0.25	0.50	0.50	0.024	-0.071	0.006
1	8	1	0.8	0.8	0.5	0.30	0.30	0.30	0.50	0.00	0.00	0.006	-0.038	0.011
1	8	1	0.8	0.8	0.5	0.30	0.30	0.30	0.50	0.00	0.25	0.017	-0.024	0.002
1	8	1	0.8	0.8	0.5	0.30	0.30	0.30	0.50	0.00	0.50	0.012	-0.026	0.004
1	8	1	0.8	0.8	0.5	0.30	0.30	0.30	0.50	0.25	0.00	0.034	-0.058	0.009

(continued)

												Mean Bias		
N	m_1	$\frac{m_2}{m_1}$	$E(C)$	$E(R)$	$E(U)$	σ_C	σ_R	σ_U	ρ_{CR}	ρ_{CU}	ρ_{RU}	c	r	u
1	8	1	0.8	0.8	0.5	0.30	0.30	0.30	0.50	0.25	0.25	0.014	-0.040	-0.002
1	8	1	0.8	0.8	0.5	0.30	0.30	0.30	0.50	0.25	0.50	0.008	-0.051	0.011
1	8	1	0.8	0.8	0.5	0.30	0.30	0.30	0.50	0.50	0.00	0.042	-0.045	-0.001
1	8	1	0.8	0.8	0.5	0.30	0.30	0.30	0.50	0.50	0.25	0.047	-0.038	0.020
1	8	1	0.8	0.8	0.5	0.30	0.30	0.30	0.50	0.50	0.50	0.030	-0.035	0.005
1	20	0	0.2	0.2	0.5	0.00	0.00	0.00	0.00	0.00	0.00	-0.014	0.080	0.012
1	20	0	0.2	0.2	0.5	0.15	0.15	0.15	0.00	0.00	0.00	0.005	0.035	0.012
1	20	0	0.2	0.2	0.5	0.15	0.15	0.15	0.00	0.00	0.25	-0.001	0.037	0.015
1	20	0	0.2	0.2	0.5	0.15	0.15	0.15	0.00	0.00	0.50	-0.015	0.042	0.012
1	20	0	0.2	0.2	0.5	0.15	0.15	0.15	0.00	0.25	0.00	0.008	0.051	0.015
1	20	0	0.2	0.2	0.5	0.15	0.15	0.15	0.00	0.25	0.25	-0.001	0.041	-0.001
1	20	0	0.2	0.2	0.5	0.15	0.15	0.15	0.00	0.25	0.50	-0.005	0.028	0.016
1	20	0	0.2	0.2	0.5	0.15	0.15	0.15	0.00	0.50	0.00	-0.003	0.038	0.005
1	20	0	0.2	0.2	0.5	0.15	0.15	0.15	0.00	0.50	0.25	0.000	0.021	0.000
1	20	0	0.2	0.2	0.5	0.15	0.15	0.15	0.00	0.50	0.50	0.011	0.020	0.020
1	20	0	0.2	0.2	0.5	0.15	0.15	0.15	0.25	0.00	0.00	0.009	0.040	0.022
1	20	0	0.2	0.2	0.5	0.15	0.15	0.15	0.25	0.00	0.25	0.005	0.051	0.024
1	20	0	0.2	0.2	0.5	0.15	0.15	0.15	0.25	0.00	0.50	-0.005	0.055	0.021
1	20	0	0.2	0.2	0.5	0.15	0.15	0.15	0.25	0.25	0.00	-0.001	0.041	0.013
1	20	0	0.2	0.2	0.5	0.15	0.15	0.15	0.25	0.25	0.25	0.001	0.035	0.013
1	20	0	0.2	0.2	0.5	0.15	0.15	0.15	0.25	0.25	0.50	0.006	0.041	0.018
1	20	0	0.2	0.2	0.5	0.15	0.15	0.15	0.25	0.50	0.00	0.005	0.020	0.016
1	20	0	0.2	0.2	0.5	0.15	0.15	0.15	0.25	0.50	0.25	0.013	0.018	0.011
1	20	0	0.2	0.2	0.5	0.15	0.15	0.15	0.25	0.50	0.50	0.011	0.023	0.022
1	20	0	0.2	0.2	0.5	0.15	0.15	0.15	0.50	0.00	0.00	0.002	0.038	0.012
1	20	0	0.2	0.2	0.5	0.15	0.15	0.15	0.50	0.00	0.25	-0.010	0.041	0.009
1	20	0	0.2	0.2	0.5	0.15	0.15	0.15	0.50	0.00	0.50	0.009	0.052	0.019
1	20	0	0.2	0.2	0.5	0.15	0.15	0.15	0.50	0.25	0.00	0.009	0.046	0.020
1	20	0	0.2	0.2	0.5	0.15	0.15	0.15	0.50	0.25	0.25	0.003	0.067	0.004
1	20	0	0.2	0.2	0.5	0.15	0.15	0.15	0.50	0.25	0.50	0.007	0.063	0.015
1	20	0	0.2	0.2	0.5	0.15	0.15	0.15	0.50	0.50	0.00	0.015	0.048	0.010
1	20	0	0.2	0.2	0.5	0.15	0.15	0.15	0.50	0.50	0.25	0.013	0.056	0.014
1	20	0	0.2	0.2	0.5	0.15	0.15	0.15	0.50	0.50	0.50	0.018	0.041	0.012
1	20	0	0.2	0.2	0.5	0.30	0.30	0.30	0.00	0.00	0.00	0.012	-0.039	-0.002
1	20	0	0.2	0.2	0.5	0.30	0.30	0.30	0.00	0.00	0.25	-0.002	-0.055	0.014
1	20	0	0.2	0.2	0.5	0.30	0.30	0.30	0.00	0.00	0.50	0.027	-0.031	-0.007
1	20	0	0.2	0.2	0.5	0.30	0.30	0.30	0.00	0.25	0.00	0.012	-0.054	-0.025
1	20	0	0.2	0.2	0.5	0.30	0.30	0.30	0.00	0.25	0.25	0.013	-0.042	0.024
1	20	0	0.2	0.2	0.5	0.30	0.30	0.30	0.00	0.25	0.50	0.017	-0.059	0.006

(continued)

												Mean Bias		
N	m_1	$\frac{m_2}{m_1}$	$E(C)$	$E(R)$	$E(U)$	σ_C	σ_R	σ_U	ρ_{CR}	ρ_{CU}	ρ_{RU}	c	r	u
1	20	0	0.2	0.2	0.5	0.30	0.30	0.30	0.00	0.50	0.00	0.039	-0.057	-0.009
1	20	0	0.2	0.2	0.5	0.30	0.30	0.30	0.00	0.50	0.25	0.013	-0.060	-0.013
1	20	0	0.2	0.2	0.5	0.30	0.30	0.30	0.00	0.50	0.50	0.040	-0.064	0.009
1	20	0	0.2	0.2	0.5	0.30	0.30	0.30	0.25	0.00	0.00	0.003	-0.018	-0.002
1	20	0	0.2	0.2	0.5	0.30	0.30	0.30	0.25	0.00	0.25	0.012	0.000	0.006
1	20	0	0.2	0.2	0.5	0.30	0.30	0.30	0.25	0.00	0.50	0.020	-0.003	0.034
1	20	0	0.2	0.2	0.5	0.30	0.30	0.30	0.25	0.25	0.00	0.039	-0.025	0.011
1	20	0	0.2	0.2	0.5	0.30	0.30	0.30	0.25	0.25	0.25	0.036	-0.024	-0.016
1	20	0	0.2	0.2	0.5	0.30	0.30	0.30	0.25	0.25	0.50	0.017	-0.046	-0.013
1	20	0	0.2	0.2	0.5	0.30	0.30	0.30	0.25	0.50	0.00	0.031	-0.030	-0.024
1	20	0	0.2	0.2	0.5	0.30	0.30	0.30	0.25	0.50	0.25	0.035	-0.029	-0.022
1	20	0	0.2	0.2	0.5	0.30	0.30	0.30	0.25	0.50	0.50	0.017	-0.036	0.009
1	20	0	0.2	0.2	0.5	0.30	0.30	0.30	0.50	0.00	0.00	-0.005	-0.009	0.026
1	20	0	0.2	0.2	0.5	0.30	0.30	0.30	0.50	0.00	0.25	0.009	0.011	0.023
1	20	0	0.2	0.2	0.5	0.30	0.30	0.30	0.50	0.00	0.50	0.009	0.017	0.010
1	20	0	0.2	0.2	0.5	0.30	0.30	0.30	0.50	0.25	0.00	0.022	-0.008	-0.005
1	20	0	0.2	0.2	0.5	0.30	0.30	0.30	0.50	0.25	0.25	0.027	0.006	0.006
1	20	0	0.2	0.2	0.5	0.30	0.30	0.30	0.50	0.25	0.50	0.021	-0.002	0.010
1	20	0	0.2	0.2	0.5	0.30	0.30	0.30	0.50	0.50	0.00	0.026	-0.009	-0.008
1	20	0	0.2	0.2	0.5	0.30	0.30	0.30	0.50	0.50	0.25	0.025	-0.016	0.007
1	20	0	0.2	0.2	0.5	0.30	0.30	0.30	0.50	0.50	0.50	0.031	0.006	-0.005
1	20	0	0.2	0.5	0.5	0.00	0.00	0.00	0.00	0.00	0.00	0.017	0.070	0.024
1	20	0	0.2	0.5	0.5	0.15	0.15	0.15	0.00	0.00	0.00	-0.002	-0.033	0.034
1	20	0	0.2	0.5	0.5	0.15	0.15	0.15	0.00	0.00	0.25	0.012	-0.011	0.023
1	20	0	0.2	0.5	0.5	0.15	0.15	0.15	0.00	0.00	0.50	0.017	-0.025	0.015
1	20	0	0.2	0.5	0.5	0.15	0.15	0.15	0.00	0.25	0.00	0.022	-0.036	0.019
1	20	0	0.2	0.5	0.5	0.15	0.15	0.15	0.00	0.25	0.25	0.026	-0.048	0.026
1	20	0	0.2	0.5	0.5	0.15	0.15	0.15	0.00	0.25	0.50	0.015	-0.019	0.009
1	20	0	0.2	0.5	0.5	0.15	0.15	0.15	0.00	0.50	0.00	0.021	-0.048	0.016
1	20	0	0.2	0.5	0.5	0.15	0.15	0.15	0.00	0.50	0.25	0.018	-0.049	0.023
1	20	0	0.2	0.5	0.5	0.15	0.15	0.15	0.00	0.50	0.50	0.024	-0.037	0.028
1	20	0	0.2	0.5	0.5	0.15	0.15	0.15	0.25	0.00	0.00	0.014	-0.013	0.016
1	20	0	0.2	0.5	0.5	0.15	0.15	0.15	0.25	0.00	0.25	0.014	-0.027	0.025
1	20	0	0.2	0.5	0.5	0.15	0.15	0.15	0.25	0.00	0.50	0.012	0.006	0.014
1	20	0	0.2	0.5	0.5	0.15	0.15	0.15	0.25	0.25	0.00	0.020	-0.026	0.029
1	20	0	0.2	0.5	0.5	0.15	0.15	0.15	0.25	0.25	0.25	0.027	-0.019	0.020
1	20	0	0.2	0.5	0.5	0.15	0.15	0.15	0.25	0.25	0.50	0.026	-0.008	0.024
1	20	0	0.2	0.5	0.5	0.15	0.15	0.15	0.25	0.50	0.00	0.017	-0.032	0.016
1	20	0	0.2	0.5	0.5	0.15	0.15	0.15	0.25	0.50	0.25	0.029	-0.029	0.030

(continued)

												Mean Bias		
N	m_1	$\frac{m_2}{m_1}$	$E(C)$	$E(R)$	$E(U)$	σ_C	σ_R	σ_U	ρ_{CR}	ρ_{CU}	ρ_{RU}	c	r	u
1	20	0	0.2	0.5	0.5	0.15	0.15	0.15	0.25	0.50	0.50	0.024	-0.010	0.016
1	20	0	0.2	0.5	0.5	0.15	0.15	0.15	0.50	0.00	0.00	0.028	-0.026	0.030
1	20	0	0.2	0.5	0.5	0.15	0.15	0.15	0.50	0.00	0.25	0.007	-0.016	0.016
1	20	0	0.2	0.5	0.5	0.15	0.15	0.15	0.50	0.00	0.50	0.012	0.025	0.020
1	20	0	0.2	0.5	0.5	0.15	0.15	0.15	0.50	0.25	0.00	0.022	-0.007	0.025
1	20	0	0.2	0.5	0.5	0.15	0.15	0.15	0.50	0.25	0.25	0.020	-0.017	0.017
1	20	0	0.2	0.5	0.5	0.15	0.15	0.15	0.50	0.25	0.50	0.035	-0.014	0.021
1	20	0	0.2	0.5	0.5	0.15	0.15	0.15	0.50	0.50	0.00	0.024	-0.025	0.026
1	20	0	0.2	0.5	0.5	0.15	0.15	0.15	0.50	0.50	0.25	0.020	-0.017	0.029
1	20	0	0.2	0.5	0.5	0.15	0.15	0.15	0.50	0.50	0.50	0.025	-0.037	0.025
1	20	0	0.2	0.5	0.5	0.30	0.30	0.30	0.00	0.00	0.00	0.026	-0.185	0.034
1	20	0	0.2	0.5	0.5	0.30	0.30	0.30	0.00	0.00	0.25	0.037	-0.173	0.017
1	20	0	0.2	0.5	0.5	0.30	0.30	0.30	0.00	0.00	0.50	0.030	-0.180	0.037
1	20	0	0.2	0.5	0.5	0.30	0.30	0.30	0.00	0.25	0.00	0.029	-0.221	0.018
1	20	0	0.2	0.5	0.5	0.30	0.30	0.30	0.00	0.25	0.25	0.023	-0.212	-0.001
1	20	0	0.2	0.5	0.5	0.30	0.30	0.30	0.00	0.25	0.50	0.029	-0.190	-0.001
1	20	0	0.2	0.5	0.5	0.30	0.30	0.30	0.00	0.50	0.00	0.041	-0.234	-0.014
1	20	0	0.2	0.5	0.5	0.30	0.30	0.30	0.00	0.50	0.25	0.053	-0.206	-0.010
1	20	0	0.2	0.5	0.5	0.30	0.30	0.30	0.00	0.50	0.50	0.037	-0.209	0.012
1	20	0	0.2	0.5	0.5	0.30	0.30	0.30	0.25	0.00	0.00	0.040	-0.154	0.026
1	20	0	0.2	0.5	0.5	0.30	0.30	0.30	0.25	0.00	0.25	0.030	-0.173	0.023
1	20	0	0.2	0.5	0.5	0.30	0.30	0.30	0.25	0.00	0.50	0.035	-0.149	0.029
1	20	0	0.2	0.5	0.5	0.30	0.30	0.30	0.25	0.25	0.00	0.053	-0.187	-0.002
1	20	0	0.2	0.5	0.5	0.30	0.30	0.30	0.25	0.25	0.25	0.048	-0.178	0.013
1	20	0	0.2	0.5	0.5	0.30	0.30	0.30	0.25	0.25	0.50	0.034	-0.178	0.004
1	20	0	0.2	0.5	0.5	0.30	0.30	0.30	0.25	0.50	0.00	0.037	-0.195	0.008
1	20	0	0.2	0.5	0.5	0.30	0.30	0.30	0.25	0.50	0.25	0.062	-0.188	0.005
1	20	0	0.2	0.5	0.5	0.30	0.30	0.30	0.25	0.50	0.50	0.055	-0.193	-0.005
1	20	0	0.2	0.5	0.5	0.30	0.30	0.30	0.50	0.00	0.00	0.032	-0.144	0.023
1	20	0	0.2	0.5	0.5	0.30	0.30	0.30	0.50	0.00	0.25	0.040	-0.155	0.010
1	20	0	0.2	0.5	0.5	0.30	0.30	0.30	0.50	0.00	0.50	0.049	-0.134	0.008
1	20	0	0.2	0.5	0.5	0.30	0.30	0.30	0.50	0.25	0.00	0.044	-0.171	0.008
1	20	0	0.2	0.5	0.5	0.30	0.30	0.30	0.50	0.25	0.25	0.047	-0.166	0.013
1	20	0	0.2	0.5	0.5	0.30	0.30	0.30	0.50	0.25	0.50	0.046	-0.165	0.002
1	20	0	0.2	0.5	0.5	0.30	0.30	0.30	0.50	0.50	0.00	0.060	-0.155	0.003
1	20	0	0.2	0.5	0.5	0.30	0.30	0.30	0.50	0.50	0.25	0.044	-0.177	0.015
1	20	0	0.2	0.5	0.5	0.30	0.30	0.30	0.50	0.50	0.50	0.047	-0.169	0.000
1	20	0	0.2	0.8	0.5	0.00	0.00	0.00	0.00	0.00	0.00	0.030	-0.056	0.031
1	20	0	0.2	0.8	0.5	0.15	0.15	0.15	0.00	0.00	0.00	0.033	-0.183	0.038

(continued)

N	m_1	$\frac{m_2}{m_1}$	$E(C)$	$E(R)$	$E(U)$	σ_C	σ_R	σ_U	ρ_{CR}	ρ_{CU}	ρ_{RU}	Mean Bias		
												c	r	u
1	20	0	0.2	0.8	0.5	0.15	0.15	0.15	0.00	0.00	0.25	0.045	-0.150	0.034
1	20	0	0.2	0.8	0.5	0.15	0.15	0.15	0.00	0.00	0.50	0.044	-0.145	0.030
1	20	0	0.2	0.8	0.5	0.15	0.15	0.15	0.00	0.25	0.00	0.025	-0.162	0.024
1	20	0	0.2	0.8	0.5	0.15	0.15	0.15	0.00	0.25	0.25	0.035	-0.175	0.027
1	20	0	0.2	0.8	0.5	0.15	0.15	0.15	0.00	0.25	0.50	0.028	-0.155	0.028
1	20	0	0.2	0.8	0.5	0.15	0.15	0.15	0.00	0.50	0.00	0.036	-0.143	0.026
1	20	0	0.2	0.8	0.5	0.15	0.15	0.15	0.00	0.50	0.25	0.040	-0.154	0.029
1	20	0	0.2	0.8	0.5	0.15	0.15	0.15	0.00	0.50	0.50	0.041	-0.163	0.027
1	20	0	0.2	0.8	0.5	0.15	0.15	0.15	0.25	0.00	0.00	0.049	-0.157	0.032
1	20	0	0.2	0.8	0.5	0.15	0.15	0.15	0.25	0.00	0.25	0.036	-0.140	0.027
1	20	0	0.2	0.8	0.5	0.15	0.15	0.15	0.25	0.00	0.50	0.035	-0.155	0.031
1	20	0	0.2	0.8	0.5	0.15	0.15	0.15	0.25	0.25	0.00	0.043	-0.131	0.032
1	20	0	0.2	0.8	0.5	0.15	0.15	0.15	0.25	0.25	0.25	0.041	-0.155	0.042
1	20	0	0.2	0.8	0.5	0.15	0.15	0.15	0.25	0.25	0.50	0.041	-0.131	0.032
1	20	0	0.2	0.8	0.5	0.15	0.15	0.15	0.25	0.50	0.00	0.055	-0.154	0.048
1	20	0	0.2	0.8	0.5	0.15	0.15	0.15	0.25	0.50	0.25	0.042	-0.145	0.036
1	20	0	0.2	0.8	0.5	0.15	0.15	0.15	0.25	0.50	0.50	0.039	-0.131	0.040
1	20	0	0.2	0.8	0.5	0.15	0.15	0.15	0.50	0.00	0.00	0.041	-0.144	0.036
1	20	0	0.2	0.8	0.5	0.15	0.15	0.15	0.50	0.00	0.25	0.034	-0.139	0.021
1	20	0	0.2	0.8	0.5	0.15	0.15	0.15	0.50	0.00	0.50	0.050	-0.136	0.034
1	20	0	0.2	0.8	0.5	0.15	0.15	0.15	0.50	0.25	0.00	0.039	-0.145	0.039
1	20	0	0.2	0.8	0.5	0.15	0.15	0.15	0.50	0.25	0.25	0.036	-0.165	0.043
1	20	0	0.2	0.8	0.5	0.15	0.15	0.15	0.50	0.25	0.50	0.054	-0.146	0.039
1	20	0	0.2	0.8	0.5	0.15	0.15	0.15	0.50	0.50	0.00	0.053	-0.134	0.033
1	20	0	0.2	0.8	0.5	0.15	0.15	0.15	0.50	0.50	0.25	0.063	-0.142	0.038
1	20	0	0.2	0.8	0.5	0.15	0.15	0.15	0.50	0.50	0.50	0.051	-0.165	0.039
1	20	0	0.2	0.8	0.5	0.30	0.30	0.30	0.00	0.00	0.00	0.052	-0.395	0.017
1	20	0	0.2	0.8	0.5	0.30	0.30	0.30	0.00	0.00	0.25	0.080	-0.357	0.022
1	20	0	0.2	0.8	0.5	0.30	0.30	0.30	0.00	0.00	0.50	0.022	-0.404	0.037
1	20	0	0.2	0.8	0.5	0.30	0.30	0.30	0.00	0.25	0.00	0.069	-0.371	0.023
1	20	0	0.2	0.8	0.5	0.30	0.30	0.30	0.00	0.25	0.25	0.051	-0.411	0.025
1	20	0	0.2	0.8	0.5	0.30	0.30	0.30	0.00	0.25	0.50	0.038	-0.383	0.012
1	20	0	0.2	0.8	0.5	0.30	0.30	0.30	0.00	0.50	0.00	0.062	-0.418	0.018
1	20	0	0.2	0.8	0.5	0.30	0.30	0.30	0.00	0.50	0.25	0.056	-0.425	0.010
1	20	0	0.2	0.8	0.5	0.30	0.30	0.30	0.00	0.50	0.50	0.049	-0.382	0.021
1	20	0	0.2	0.8	0.5	0.30	0.30	0.30	0.25	0.00	0.00	0.046	-0.392	0.038
1	20	0	0.2	0.8	0.5	0.30	0.30	0.30	0.25	0.00	0.25	0.049	-0.370	0.023
1	20	0	0.2	0.8	0.5	0.30	0.30	0.30	0.25	0.00	0.50	0.049	-0.363	0.039
1	20	0	0.2	0.8	0.5	0.30	0.30	0.30	0.25	0.25	0.00	0.057	-0.413	0.017

(continued)

												Mean Bias		
N	m_1	$\frac{m_2}{m_1}$	$E(C)$	$E(R)$	$E(U)$	σ_C	σ_R	σ_U	ρ_{CR}	ρ_{CU}	ρ_{RU}	c	r	u
1	20	0	0.2	0.8	0.5	0.30	0.30	0.30	0.25	0.25	0.25	0.051	-0.378	0.022
1	20	0	0.2	0.8	0.5	0.30	0.30	0.30	0.25	0.25	0.50	0.057	-0.378	0.025
1	20	0	0.2	0.8	0.5	0.30	0.30	0.30	0.25	0.50	0.00	0.064	-0.399	0.017
1	20	0	0.2	0.8	0.5	0.30	0.30	0.30	0.25	0.50	0.25	0.051	-0.382	0.015
1	20	0	0.2	0.8	0.5	0.30	0.30	0.30	0.25	0.50	0.50	0.069	-0.360	-0.004
1	20	0	0.2	0.8	0.5	0.30	0.30	0.30	0.50	0.00	0.00	0.055	-0.366	0.043
1	20	0	0.2	0.8	0.5	0.30	0.30	0.30	0.50	0.00	0.25	0.059	-0.357	0.029
1	20	0	0.2	0.8	0.5	0.30	0.30	0.30	0.50	0.00	0.50	0.046	-0.357	0.038
1	20	0	0.2	0.8	0.5	0.30	0.30	0.30	0.50	0.25	0.00	0.058	-0.375	0.022
1	20	0	0.2	0.8	0.5	0.30	0.30	0.30	0.50	0.25	0.25	0.062	-0.358	0.028
1	20	0	0.2	0.8	0.5	0.30	0.30	0.30	0.50	0.25	0.50	0.051	-0.365	0.013
1	20	0	0.2	0.8	0.5	0.30	0.30	0.30	0.50	0.50	0.00	0.057	-0.375	0.018
1	20	0	0.2	0.8	0.5	0.30	0.30	0.30	0.50	0.50	0.25	0.059	-0.387	0.029
1	20	0	0.2	0.8	0.5	0.30	0.30	0.30	0.50	0.50	0.50	0.036	-0.383	0.005
1	20	0	0.5	0.2	0.5	0.00	0.00	0.00	0.00	0.00	0.00	-0.073	0.121	-0.017
1	20	0	0.5	0.2	0.5	0.15	0.15	0.15	0.00	0.00	0.00	-0.085	0.129	-0.014
1	20	0	0.5	0.2	0.5	0.15	0.15	0.15	0.00	0.00	0.25	-0.083	0.137	-0.008
1	20	0	0.5	0.2	0.5	0.15	0.15	0.15	0.00	0.00	0.50	-0.102	0.113	-0.016
1	20	0	0.5	0.2	0.5	0.15	0.15	0.15	0.00	0.25	0.00	-0.075	0.126	-0.007
1	20	0	0.5	0.2	0.5	0.15	0.15	0.15	0.00	0.25	0.25	-0.086	0.122	-0.007
1	20	0	0.5	0.2	0.5	0.15	0.15	0.15	0.00	0.25	0.50	-0.087	0.125	-0.019
1	20	0	0.5	0.2	0.5	0.15	0.15	0.15	0.00	0.50	0.00	-0.069	0.105	-0.006
1	20	0	0.5	0.2	0.5	0.15	0.15	0.15	0.00	0.50	0.25	-0.065	0.106	-0.001
1	20	0	0.5	0.2	0.5	0.15	0.15	0.15	0.00	0.50	0.50	-0.070	0.119	-0.008
1	20	0	0.5	0.2	0.5	0.15	0.15	0.15	0.25	0.00	0.00	-0.082	0.132	-0.013
1	20	0	0.5	0.2	0.5	0.15	0.15	0.15	0.25	0.00	0.25	-0.083	0.124	-0.007
1	20	0	0.5	0.2	0.5	0.15	0.15	0.15	0.25	0.00	0.50	-0.097	0.133	-0.009
1	20	0	0.5	0.2	0.5	0.15	0.15	0.15	0.25	0.25	0.00	-0.074	0.132	-0.010
1	20	0	0.5	0.2	0.5	0.15	0.15	0.15	0.25	0.25	0.25	-0.091	0.144	-0.020
1	20	0	0.5	0.2	0.5	0.15	0.15	0.15	0.25	0.25	0.50	-0.074	0.113	0.002
1	20	0	0.5	0.2	0.5	0.15	0.15	0.15	0.25	0.50	0.00	-0.076	0.130	-0.015
1	20	0	0.5	0.2	0.5	0.15	0.15	0.15	0.25	0.50	0.25	-0.077	0.123	-0.024
1	20	0	0.5	0.2	0.5	0.15	0.15	0.15	0.25	0.50	0.50	-0.087	0.112	-0.013
1	20	0	0.5	0.2	0.5	0.15	0.15	0.15	0.50	0.00	0.00	-0.088	0.111	0.007
1	20	0	0.5	0.2	0.5	0.15	0.15	0.15	0.50	0.00	0.25	-0.090	0.126	-0.012
1	20	0	0.5	0.2	0.5	0.15	0.15	0.15	0.50	0.00	0.50	-0.106	0.090	-0.020
1	20	0	0.5	0.2	0.5	0.15	0.15	0.15	0.50	0.25	0.00	-0.073	0.111	-0.015
1	20	0	0.5	0.2	0.5	0.15	0.15	0.15	0.50	0.25	0.25	-0.091	0.108	-0.013
1	20	0	0.5	0.2	0.5	0.15	0.15	0.15	0.50	0.25	0.50	-0.071	0.108	-0.009

(continued)

												Mean Bias		
N	m_1	$\frac{m_2}{m_1}$	$E(C)$	$E(R)$	$E(U)$	σ_C	σ_R	σ_U	ρ_{CR}	ρ_{CU}	ρ_{RU}	c	r	u
1	20	0	0.5	0.2	0.5	0.15	0.15	0.15	0.50	0.50	0.00	-0.058	0.108	-0.001
1	20	0	0.5	0.2	0.5	0.15	0.15	0.15	0.50	0.50	0.25	-0.067	0.110	-0.009
1	20	0	0.5	0.2	0.5	0.15	0.15	0.15	0.50	0.50	0.50	-0.075	0.108	-0.017
1	20	0	0.5	0.2	0.5	0.30	0.30	0.30	0.00	0.00	0.00	-0.081	0.052	-0.047
1	20	0	0.5	0.2	0.5	0.30	0.30	0.30	0.00	0.00	0.25	-0.103	0.030	-0.028
1	20	0	0.5	0.2	0.5	0.30	0.30	0.30	0.00	0.00	0.50	-0.125	0.025	-0.038
1	20	0	0.5	0.2	0.5	0.30	0.30	0.30	0.00	0.25	0.00	-0.079	0.047	-0.030
1	20	0	0.5	0.2	0.5	0.30	0.30	0.30	0.00	0.25	0.25	-0.087	0.034	-0.034
1	20	0	0.5	0.2	0.5	0.30	0.30	0.30	0.00	0.25	0.50	-0.111	0.041	-0.012
1	20	0	0.5	0.2	0.5	0.30	0.30	0.30	0.00	0.50	0.00	-0.082	0.063	-0.033
1	20	0	0.5	0.2	0.5	0.30	0.30	0.30	0.00	0.50	0.25	-0.083	0.040	-0.041
1	20	0	0.5	0.2	0.5	0.30	0.30	0.30	0.00	0.50	0.50	-0.073	0.041	-0.042
1	20	0	0.5	0.2	0.5	0.30	0.30	0.30	0.25	0.00	0.00	-0.095	0.048	-0.025
1	20	0	0.5	0.2	0.5	0.30	0.30	0.30	0.25	0.00	0.25	-0.082	0.055	-0.017
1	20	0	0.5	0.2	0.5	0.30	0.30	0.30	0.25	0.00	0.50	-0.121	0.047	-0.036
1	20	0	0.5	0.2	0.5	0.30	0.30	0.30	0.25	0.25	0.00	-0.045	0.055	-0.015
1	20	0	0.5	0.2	0.5	0.30	0.30	0.30	0.25	0.25	0.25	-0.094	0.043	-0.017
1	20	0	0.5	0.2	0.5	0.30	0.30	0.30	0.25	0.25	0.50	-0.094	0.054	-0.024
1	20	0	0.5	0.2	0.5	0.30	0.30	0.30	0.25	0.50	0.00	-0.087	0.044	-0.037
1	20	0	0.5	0.2	0.5	0.30	0.30	0.30	0.25	0.50	0.25	-0.059	0.063	-0.042
1	20	0	0.5	0.2	0.5	0.30	0.30	0.30	0.25	0.50	0.50	-0.075	0.044	-0.029
1	20	0	0.5	0.2	0.5	0.30	0.30	0.30	0.50	0.00	0.00	-0.089	0.088	-0.020
1	20	0	0.5	0.2	0.5	0.30	0.30	0.30	0.50	0.00	0.25	-0.094	0.078	-0.012
1	20	0	0.5	0.2	0.5	0.30	0.30	0.30	0.50	0.00	0.50	-0.108	0.064	-0.013
1	20	0	0.5	0.2	0.5	0.30	0.30	0.30	0.50	0.25	0.00	-0.077	0.090	-0.017
1	20	0	0.5	0.2	0.5	0.30	0.30	0.30	0.50	0.25	0.25	-0.065	0.064	-0.007
1	20	0	0.5	0.2	0.5	0.30	0.30	0.30	0.50	0.25	0.50	-0.071	0.043	-0.022
1	20	0	0.5	0.2	0.5	0.30	0.30	0.30	0.50	0.50	0.00	-0.043	0.055	-0.021
1	20	0	0.5	0.2	0.5	0.30	0.30	0.30	0.50	0.50	0.25	-0.046	0.064	-0.020
1	20	0	0.5	0.2	0.5	0.30	0.30	0.30	0.50	0.50	0.50	-0.070	0.072	-0.037
1	20	0	0.5	0.5	0.5	0.00	0.00	0.00	0.00	0.00	0.00	-0.029	0.094	0.008
1	20	0	0.5	0.5	0.5	0.15	0.15	0.15	0.00	0.00	0.00	-0.040	0.112	0.001
1	20	0	0.5	0.5	0.5	0.15	0.15	0.15	0.00	0.00	0.25	-0.055	0.129	-0.005
1	20	0	0.5	0.5	0.5	0.15	0.15	0.15	0.00	0.00	0.50	-0.054	0.128	0.003
1	20	0	0.5	0.5	0.5	0.15	0.15	0.15	0.00	0.25	0.00	-0.044	0.117	0.003
1	20	0	0.5	0.5	0.5	0.15	0.15	0.15	0.00	0.25	0.25	-0.054	0.126	-0.012
1	20	0	0.5	0.5	0.5	0.15	0.15	0.15	0.00	0.25	0.50	-0.044	0.109	0.003
1	20	0	0.5	0.5	0.5	0.15	0.15	0.15	0.00	0.50	0.00	-0.026	0.104	0.009
1	20	0	0.5	0.5	0.5	0.15	0.15	0.15	0.00	0.50	0.25	-0.042	0.109	0.003

(continued)

												Mean Bias		
N	m_1	$\frac{m_2}{m_1}$	$E(C)$	$E(R)$	$E(U)$	σ_C	σ_R	σ_U	ρ_{CR}	ρ_{CU}	ρ_{RU}	c	r	u
1	20	0	0.5	0.5	0.5	0.15	0.15	0.15	0.00	0.50	0.50	-0.040	0.103	-0.001
1	20	0	0.5	0.5	0.5	0.15	0.15	0.15	0.25	0.00	0.00	-0.042	0.120	0.000
1	20	0	0.5	0.5	0.5	0.15	0.15	0.15	0.25	0.00	0.25	-0.054	0.113	0.000
1	20	0	0.5	0.5	0.5	0.15	0.15	0.15	0.25	0.00	0.50	-0.050	0.121	-0.009
1	20	0	0.5	0.5	0.5	0.15	0.15	0.15	0.25	0.25	0.00	-0.039	0.112	-0.006
1	20	0	0.5	0.5	0.5	0.15	0.15	0.15	0.25	0.25	0.25	-0.045	0.121	-0.008
1	20	0	0.5	0.5	0.5	0.15	0.15	0.15	0.25	0.25	0.50	-0.050	0.119	0.008
1	20	0	0.5	0.5	0.5	0.15	0.15	0.15	0.25	0.50	0.00	-0.035	0.118	0.000
1	20	0	0.5	0.5	0.5	0.15	0.15	0.15	0.25	0.50	0.25	-0.029	0.096	0.006
1	20	0	0.5	0.5	0.5	0.15	0.15	0.15	0.25	0.50	0.50	-0.046	0.136	-0.012
1	20	0	0.5	0.5	0.5	0.15	0.15	0.15	0.50	0.00	0.00	-0.045	0.107	0.003
1	20	0	0.5	0.5	0.5	0.15	0.15	0.15	0.50	0.00	0.25	-0.046	0.125	-0.005
1	20	0	0.5	0.5	0.5	0.15	0.15	0.15	0.50	0.00	0.50	-0.046	0.127	-0.001
1	20	0	0.5	0.5	0.5	0.15	0.15	0.15	0.50	0.25	0.00	-0.042	0.111	-0.009
1	20	0	0.5	0.5	0.5	0.15	0.15	0.15	0.50	0.25	0.25	-0.047	0.119	-0.013
1	20	0	0.5	0.5	0.5	0.15	0.15	0.15	0.50	0.25	0.50	-0.043	0.114	0.001
1	20	0	0.5	0.5	0.5	0.15	0.15	0.15	0.50	0.50	0.00	-0.035	0.104	0.003
1	20	0	0.5	0.5	0.5	0.15	0.15	0.15	0.50	0.50	0.25	-0.043	0.113	-0.005
1	20	0	0.5	0.5	0.5	0.15	0.15	0.15	0.50	0.50	0.50	-0.040	0.128	0.000
1	20	0	0.5	0.5	0.5	0.30	0.30	0.30	0.00	0.00	0.00	-0.035	0.060	-0.014
1	20	0	0.5	0.5	0.5	0.30	0.30	0.30	0.00	0.00	0.25	-0.044	0.053	-0.006
1	20	0	0.5	0.5	0.5	0.30	0.30	0.30	0.00	0.00	0.50	-0.064	0.069	0.012
1	20	0	0.5	0.5	0.5	0.30	0.30	0.30	0.00	0.25	0.00	-0.044	0.053	-0.022
1	20	0	0.5	0.5	0.5	0.30	0.30	0.30	0.00	0.25	0.25	-0.030	0.056	-0.019
1	20	0	0.5	0.5	0.5	0.30	0.30	0.30	0.00	0.25	0.50	-0.059	0.026	-0.040
1	20	0	0.5	0.5	0.5	0.30	0.30	0.30	0.00	0.50	0.00	-0.005	0.030	-0.004
1	20	0	0.5	0.5	0.5	0.30	0.30	0.30	0.00	0.50	0.25	-0.029	0.030	-0.040
1	20	0	0.5	0.5	0.5	0.30	0.30	0.30	0.00	0.50	0.50	-0.043	0.048	-0.039
1	20	0	0.5	0.5	0.5	0.30	0.30	0.30	0.25	0.00	0.00	-0.008	0.062	0.012
1	20	0	0.5	0.5	0.5	0.30	0.30	0.30	0.25	0.00	0.25	-0.039	0.069	0.005
1	20	0	0.5	0.5	0.5	0.30	0.30	0.30	0.25	0.00	0.50	-0.048	0.077	0.022
1	20	0	0.5	0.5	0.5	0.30	0.30	0.30	0.25	0.25	0.00	-0.027	0.063	-0.034
1	20	0	0.5	0.5	0.5	0.30	0.30	0.30	0.25	0.25	0.25	-0.034	0.066	-0.013
1	20	0	0.5	0.5	0.5	0.30	0.30	0.30	0.25	0.25	0.50	-0.049	0.091	-0.015
1	20	0	0.5	0.5	0.5	0.30	0.30	0.30	0.25	0.50	0.00	-0.038	0.044	-0.038
1	20	0	0.5	0.5	0.5	0.30	0.30	0.30	0.25	0.50	0.25	0.011	0.055	-0.013
1	20	0	0.5	0.5	0.5	0.30	0.30	0.30	0.25	0.50	0.50	-0.028	0.057	-0.014
1	20	0	0.5	0.5	0.5	0.30	0.30	0.30	0.50	0.00	0.00	-0.005	0.060	0.009
1	20	0	0.5	0.5	0.5	0.30	0.30	0.30	0.50	0.00	0.25	-0.023	0.066	-0.009

(continued)

N	m_1	$\frac{m_2}{m_1}$	$E(C)$	$E(R)$	$E(U)$	σ_C	σ_R	σ_U	ρ_{CR}	ρ_{CU}	ρ_{RU}	Mean Bias c	Mean Bias r	Mean Bias u
1	20	0	0.5	0.5	0.5	0.30	0.30	0.30	0.50	0.00	0.50	-0.040	0.091	0.013
1	20	0	0.5	0.5	0.5	0.30	0.30	0.30	0.50	0.25	0.00	-0.010	0.054	-0.003
1	20	0	0.5	0.5	0.5	0.30	0.30	0.30	0.50	0.25	0.25	-0.038	0.051	-0.014
1	20	0	0.5	0.5	0.5	0.30	0.30	0.30	0.50	0.25	0.50	-0.037	0.088	-0.005
1	20	0	0.5	0.5	0.5	0.30	0.30	0.30	0.50	0.50	0.00	0.005	0.035	0.005
1	20	0	0.5	0.5	0.5	0.30	0.30	0.30	0.50	0.50	0.25	0.004	0.069	-0.003
1	20	0	0.5	0.5	0.5	0.30	0.30	0.30	0.50	0.50	0.50	-0.028	0.057	-0.016
1	20	0	0.5	0.8	0.5	0.00	0.00	0.00	0.00	0.00	0.00	0.001	0.030	0.023
1	20	0	0.5	0.8	0.5	0.15	0.15	0.15	0.00	0.00	0.00	0.002	0.019	0.040
1	20	0	0.5	0.8	0.5	0.15	0.15	0.15	0.00	0.00	0.25	-0.009	0.021	0.024
1	20	0	0.5	0.8	0.5	0.15	0.15	0.15	0.00	0.00	0.50	-0.009	0.028	0.011
1	20	0	0.5	0.8	0.5	0.15	0.15	0.15	0.00	0.25	0.00	-0.003	0.014	0.023
1	20	0	0.5	0.8	0.5	0.15	0.15	0.15	0.00	0.25	0.25	0.001	0.036	0.026
1	20	0	0.5	0.8	0.5	0.15	0.15	0.15	0.00	0.25	0.50	0.006	0.023	0.029
1	20	0	0.5	0.8	0.5	0.15	0.15	0.15	0.00	0.50	0.00	0.013	0.001	0.033
1	20	0	0.5	0.8	0.5	0.15	0.15	0.15	0.00	0.50	0.25	-0.004	0.025	0.021
1	20	0	0.5	0.8	0.5	0.15	0.15	0.15	0.00	0.50	0.50	0.004	0.019	0.025
1	20	0	0.5	0.8	0.5	0.15	0.15	0.15	0.25	0.00	0.00	-0.006	0.021	0.030
1	20	0	0.5	0.8	0.5	0.15	0.15	0.15	0.25	0.00	0.25	-0.004	0.022	0.027
1	20	0	0.5	0.8	0.5	0.15	0.15	0.15	0.25	0.00	0.50	-0.004	0.032	0.019
1	20	0	0.5	0.8	0.5	0.15	0.15	0.15	0.25	0.25	0.00	0.004	0.009	0.032
1	20	0	0.5	0.8	0.5	0.15	0.15	0.15	0.25	0.25	0.25	-0.002	0.024	0.018
1	20	0	0.5	0.8	0.5	0.15	0.15	0.15	0.25	0.25	0.50	0.013	0.023	0.025
1	20	0	0.5	0.8	0.5	0.15	0.15	0.15	0.25	0.50	0.00	0.011	0.001	0.025
1	20	0	0.5	0.8	0.5	0.15	0.15	0.15	0.25	0.50	0.25	0.006	0.005	0.034
1	20	0	0.5	0.8	0.5	0.15	0.15	0.15	0.25	0.50	0.50	-0.003	0.027	0.016
1	20	0	0.5	0.8	0.5	0.15	0.15	0.15	0.50	0.00	0.00	0.011	0.030	0.032
1	20	0	0.5	0.8	0.5	0.15	0.15	0.15	0.50	0.00	0.25	0.008	0.027	0.032
1	20	0	0.5	0.8	0.5	0.15	0.15	0.15	0.50	0.00	0.50	0.001	0.027	0.017
1	20	0	0.5	0.8	0.5	0.15	0.15	0.15	0.50	0.25	0.00	0.012	0.029	0.022
1	20	0	0.5	0.8	0.5	0.15	0.15	0.15	0.50	0.25	0.25	0.001	0.030	0.020
1	20	0	0.5	0.8	0.5	0.15	0.15	0.15	0.50	0.25	0.50	-0.007	0.037	0.019
1	20	0	0.5	0.8	0.5	0.15	0.15	0.15	0.50	0.50	0.00	0.014	0.006	0.036
1	20	0	0.5	0.8	0.5	0.15	0.15	0.15	0.50	0.50	0.25	0.001	0.009	0.016
1	20	0	0.5	0.8	0.5	0.15	0.15	0.15	0.50	0.50	0.50	0.002	0.027	0.021
1	20	0	0.5	0.8	0.5	0.30	0.30	0.30	0.00	0.00	0.00	-0.019	-0.056	0.021
1	20	0	0.5	0.8	0.5	0.30	0.30	0.30	0.00	0.00	0.25	0.010	-0.060	0.017
1	20	0	0.5	0.8	0.5	0.30	0.30	0.30	0.00	0.00	0.50	-0.014	-0.050	0.021
1	20	0	0.5	0.8	0.5	0.30	0.30	0.30	0.00	0.25	0.00	0.037	-0.052	0.021

(continued)

												Mean Bias		
N	m_1	$\frac{m_2}{m_1}$	$E(C)$	$E(R)$	$E(U)$	σ_C	σ_R	σ_U	ρ_{CR}	ρ_{CU}	ρ_{RU}	c	r	u
1	20	0	0.5	0.8	0.5	0.30	0.30	0.30	0.00	0.25	0.25	0.019	-0.066	0.021
1	20	0	0.5	0.8	0.5	0.30	0.30	0.30	0.00	0.25	0.50	0.021	-0.037	0.027
1	20	0	0.5	0.8	0.5	0.30	0.30	0.30	0.00	0.50	0.00	0.026	-0.081	0.019
1	20	0	0.5	0.8	0.5	0.30	0.30	0.30	0.00	0.50	0.25	0.011	-0.049	0.004
1	20	0	0.5	0.8	0.5	0.30	0.30	0.30	0.00	0.50	0.50	0.009	-0.042	-0.003
1	20	0	0.5	0.8	0.5	0.30	0.30	0.30	0.25	0.00	0.00	0.024	-0.049	0.027
1	20	0	0.5	0.8	0.5	0.30	0.30	0.30	0.25	0.00	0.25	0.011	-0.043	0.023
1	20	0	0.5	0.8	0.5	0.30	0.30	0.30	0.25	0.00	0.50	0.016	-0.042	0.022
1	20	0	0.5	0.8	0.5	0.30	0.30	0.30	0.25	0.25	0.00	0.040	-0.058	0.022
1	20	0	0.5	0.8	0.5	0.30	0.30	0.30	0.25	0.25	0.25	0.005	-0.049	0.022
1	20	0	0.5	0.8	0.5	0.30	0.30	0.30	0.25	0.25	0.50	0.017	-0.049	0.018
1	20	0	0.5	0.8	0.5	0.30	0.30	0.30	0.25	0.50	0.00	0.035	-0.056	0.013
1	20	0	0.5	0.8	0.5	0.30	0.30	0.30	0.25	0.50	0.25	0.024	-0.074	0.032
1	20	0	0.5	0.8	0.5	0.30	0.30	0.30	0.25	0.50	0.50	0.023	-0.045	0.013
1	20	0	0.5	0.8	0.5	0.30	0.30	0.30	0.50	0.00	0.00	0.046	-0.020	0.038
1	20	0	0.5	0.8	0.5	0.30	0.30	0.30	0.50	0.00	0.25	0.023	-0.041	0.037
1	20	0	0.5	0.8	0.5	0.30	0.30	0.30	0.50	0.00	0.50	0.024	-0.023	0.047
1	20	0	0.5	0.8	0.5	0.30	0.30	0.30	0.50	0.25	0.00	0.030	-0.058	0.034
1	20	0	0.5	0.8	0.5	0.30	0.30	0.30	0.50	0.25	0.25	0.025	-0.026	0.040
1	20	0	0.5	0.8	0.5	0.30	0.30	0.30	0.50	0.25	0.50	0.036	-0.041	0.014
1	20	0	0.5	0.8	0.5	0.30	0.30	0.30	0.50	0.50	0.00	0.039	-0.060	0.021
1	20	0	0.5	0.8	0.5	0.30	0.30	0.30	0.50	0.50	0.25	0.031	-0.055	0.001
1	20	0	0.5	0.8	0.5	0.30	0.30	0.30	0.50	0.50	0.50	0.035	-0.033	0.021
1	20	0	0.8	0.2	0.5	0.00	0.00	0.00	0.00	0.00	0.00	-0.228	0.266	-0.070
1	20	0	0.8	0.2	0.5	0.15	0.15	0.15	0.00	0.00	0.00	-0.232	0.202	-0.097
1	20	0	0.8	0.2	0.5	0.15	0.15	0.15	0.00	0.00	0.25	-0.235	0.225	-0.110
1	20	0	0.8	0.2	0.5	0.15	0.15	0.15	0.00	0.00	0.50	-0.243	0.186	-0.106
1	20	0	0.8	0.2	0.5	0.15	0.15	0.15	0.00	0.25	0.00	-0.234	0.200	-0.113
1	20	0	0.8	0.2	0.5	0.15	0.15	0.15	0.00	0.25	0.25	-0.248	0.213	-0.113
1	20	0	0.8	0.2	0.5	0.15	0.15	0.15	0.00	0.25	0.50	-0.249	0.228	-0.116
1	20	0	0.8	0.2	0.5	0.15	0.15	0.15	0.00	0.50	0.00	-0.233	0.221	-0.107
1	20	0	0.8	0.2	0.5	0.15	0.15	0.15	0.00	0.50	0.25	-0.240	0.220	-0.107
1	20	0	0.8	0.2	0.5	0.15	0.15	0.15	0.00	0.50	0.50	-0.252	0.223	-0.126
1	20	0	0.8	0.2	0.5	0.15	0.15	0.15	0.25	0.00	0.00	-0.211	0.199	-0.087
1	20	0	0.8	0.2	0.5	0.15	0.15	0.15	0.25	0.00	0.25	-0.220	0.209	-0.102
1	20	0	0.8	0.2	0.5	0.15	0.15	0.15	0.25	0.00	0.50	-0.219	0.199	-0.094
1	20	0	0.8	0.2	0.5	0.15	0.15	0.15	0.25	0.25	0.00	-0.218	0.208	-0.096
1	20	0	0.8	0.2	0.5	0.15	0.15	0.15	0.25	0.25	0.25	-0.223	0.222	-0.107
1	20	0	0.8	0.2	0.5	0.15	0.15	0.15	0.25	0.25	0.50	-0.219	0.182	-0.092

(continued)

N	m_1	$\frac{m_2}{m_1}$	$E(C)$	$E(R)$	$E(U)$	σ_C	σ_R	σ_U	ρ_{CR}	ρ_{CU}	ρ_{RU}	Mean Bias c	r	u
1	20	0	0.8	0.2	0.5	0.15	0.15	0.15	0.25	0.50	0.00	-0.223	0.217	-0.106
1	20	0	0.8	0.2	0.5	0.15	0.15	0.15	0.25	0.50	0.25	-0.222	0.191	-0.098
1	20	0	0.8	0.2	0.5	0.15	0.15	0.15	0.25	0.50	0.50	-0.242	0.213	-0.103
1	20	0	0.8	0.2	0.5	0.15	0.15	0.15	0.50	0.00	0.00	-0.214	0.193	-0.100
1	20	0	0.8	0.2	0.5	0.15	0.15	0.15	0.50	0.00	0.25	-0.217	0.196	-0.097
1	20	0	0.8	0.2	0.5	0.15	0.15	0.15	0.50	0.00	0.50	-0.211	0.181	-0.064
1	20	0	0.8	0.2	0.5	0.15	0.15	0.15	0.50	0.25	0.00	-0.224	0.199	-0.082
1	20	0	0.8	0.2	0.5	0.15	0.15	0.15	0.50	0.25	0.25	-0.222	0.211	-0.107
1	20	0	0.8	0.2	0.5	0.15	0.15	0.15	0.50	0.25	0.50	-0.245	0.217	-0.127
1	20	0	0.8	0.2	0.5	0.15	0.15	0.15	0.50	0.50	0.00	-0.204	0.204	-0.095
1	20	0	0.8	0.2	0.5	0.15	0.15	0.15	0.50	0.50	0.25	-0.208	0.192	-0.085
1	20	0	0.8	0.2	0.5	0.15	0.15	0.15	0.50	0.50	0.50	-0.241	0.222	-0.120
1	20	0	0.8	0.2	0.5	0.30	0.30	0.30	0.00	0.00	0.00	-0.197	0.055	-0.195
1	20	0	0.8	0.2	0.5	0.30	0.30	0.30	0.00	0.00	0.25	-0.197	0.065	-0.191
1	20	0	0.8	0.2	0.5	0.30	0.30	0.30	0.00	0.00	0.50	-0.215	0.033	-0.192
1	20	0	0.8	0.2	0.5	0.30	0.30	0.30	0.00	0.25	0.00	-0.212	0.033	-0.204
1	20	0	0.8	0.2	0.5	0.30	0.30	0.30	0.00	0.25	0.25	-0.200	0.043	-0.205
1	20	0	0.8	0.2	0.5	0.30	0.30	0.30	0.00	0.25	0.50	-0.196	0.059	-0.209
1	20	0	0.8	0.2	0.5	0.30	0.30	0.30	0.00	0.50	0.00	-0.201	0.048	-0.242
1	20	0	0.8	0.2	0.5	0.30	0.30	0.30	0.00	0.50	0.25	-0.201	0.062	-0.244
1	20	0	0.8	0.2	0.5	0.30	0.30	0.30	0.00	0.50	0.50	-0.190	0.067	-0.239
1	20	0	0.8	0.2	0.5	0.30	0.30	0.30	0.25	0.00	0.00	-0.186	0.050	-0.180
1	20	0	0.8	0.2	0.5	0.30	0.30	0.30	0.25	0.00	0.25	-0.189	0.059	-0.180
1	20	0	0.8	0.2	0.5	0.30	0.30	0.30	0.25	0.00	0.50	-0.189	0.051	-0.193
1	20	0	0.8	0.2	0.5	0.30	0.30	0.30	0.25	0.25	0.00	-0.194	0.049	-0.210
1	20	0	0.8	0.2	0.5	0.30	0.30	0.30	0.25	0.25	0.25	-0.205	0.053	-0.194
1	20	0	0.8	0.2	0.5	0.30	0.30	0.30	0.25	0.25	0.50	-0.185	0.049	-0.215
1	20	0	0.8	0.2	0.5	0.30	0.30	0.30	0.25	0.50	0.00	-0.186	0.062	-0.236
1	20	0	0.8	0.2	0.5	0.30	0.30	0.30	0.25	0.50	0.25	-0.180	0.056	-0.233
1	20	0	0.8	0.2	0.5	0.30	0.30	0.30	0.25	0.50	0.50	-0.220	0.055	-0.237
1	20	0	0.8	0.2	0.5	0.30	0.30	0.30	0.50	0.00	0.00	-0.176	0.058	-0.199
1	20	0	0.8	0.2	0.5	0.30	0.30	0.30	0.50	0.00	0.25	-0.193	0.062	-0.167
1	20	0	0.8	0.2	0.5	0.30	0.30	0.30	0.50	0.00	0.50	-0.181	0.046	-0.165
1	20	0	0.8	0.2	0.5	0.30	0.30	0.30	0.50	0.25	0.00	-0.199	0.047	-0.199
1	20	0	0.8	0.2	0.5	0.30	0.30	0.30	0.50	0.25	0.25	-0.176	0.083	-0.197
1	20	0	0.8	0.2	0.5	0.30	0.30	0.30	0.50	0.25	0.50	-0.198	0.052	-0.189
1	20	0	0.8	0.2	0.5	0.30	0.30	0.30	0.50	0.50	0.00	-0.208	0.050	-0.228
1	20	0	0.8	0.2	0.5	0.30	0.30	0.30	0.50	0.50	0.25	-0.160	0.056	-0.242
1	20	0	0.8	0.2	0.5	0.30	0.30	0.30	0.50	0.50	0.50	-0.201	0.053	-0.225

(continued)

												Mean Bias		
N	m_1	$\frac{m_2}{m_1}$	$E(C)$	$E(R)$	$E(U)$	σ_C	σ_R	σ_U	ρ_{CR}	ρ_{CU}	ρ_{RU}	c	r	u
1	20	0	0.8	0.5	0.5	0.00	0.00	0.00	0.00	0.00	0.00	-0.140	0.169	-0.049
1	20	0	0.8	0.5	0.5	0.15	0.15	0.15	0.00	0.00	0.00	-0.139	0.158	-0.076
1	20	0	0.8	0.5	0.5	0.15	0.15	0.15	0.00	0.00	0.25	-0.138	0.164	-0.078
1	20	0	0.8	0.5	0.5	0.15	0.15	0.15	0.00	0.00	0.50	-0.128	0.156	-0.070
1	20	0	0.8	0.5	0.5	0.15	0.15	0.15	0.00	0.25	0.00	-0.141	0.172	-0.090
1	20	0	0.8	0.5	0.5	0.15	0.15	0.15	0.00	0.25	0.25	-0.148	0.170	-0.084
1	20	0	0.8	0.5	0.5	0.15	0.15	0.15	0.00	0.25	0.50	-0.140	0.177	-0.083
1	20	0	0.8	0.5	0.5	0.15	0.15	0.15	0.00	0.50	0.00	-0.142	0.167	-0.100
1	20	0	0.8	0.5	0.5	0.15	0.15	0.15	0.00	0.50	0.25	-0.128	0.163	-0.080
1	20	0	0.8	0.5	0.5	0.15	0.15	0.15	0.00	0.50	0.50	-0.128	0.178	-0.093
1	20	0	0.8	0.5	0.5	0.15	0.15	0.15	0.25	0.00	0.00	-0.128	0.168	-0.084
1	20	0	0.8	0.5	0.5	0.15	0.15	0.15	0.25	0.00	0.25	-0.140	0.179	-0.092
1	20	0	0.8	0.5	0.5	0.15	0.15	0.15	0.25	0.00	0.50	-0.138	0.192	-0.111
1	20	0	0.8	0.5	0.5	0.15	0.15	0.15	0.25	0.25	0.00	-0.127	0.161	-0.077
1	20	0	0.8	0.5	0.5	0.15	0.15	0.15	0.25	0.25	0.25	-0.134	0.171	-0.079
1	20	0	0.8	0.5	0.5	0.15	0.15	0.15	0.25	0.25	0.50	-0.138	0.183	-0.085
1	20	0	0.8	0.5	0.5	0.15	0.15	0.15	0.25	0.50	0.00	-0.128	0.171	-0.090
1	20	0	0.8	0.5	0.5	0.15	0.15	0.15	0.25	0.50	0.25	-0.131	0.174	-0.086
1	20	0	0.8	0.5	0.5	0.15	0.15	0.15	0.25	0.50	0.50	-0.125	0.187	-0.086
1	20	0	0.8	0.5	0.5	0.15	0.15	0.15	0.50	0.00	0.00	-0.123	0.160	-0.076
1	20	0	0.8	0.5	0.5	0.15	0.15	0.15	0.50	0.00	0.25	-0.135	0.169	-0.086
1	20	0	0.8	0.5	0.5	0.15	0.15	0.15	0.50	0.00	0.50	-0.145	0.181	-0.092
1	20	0	0.8	0.5	0.5	0.15	0.15	0.15	0.50	0.25	0.00	-0.114	0.159	-0.077
1	20	0	0.8	0.5	0.5	0.15	0.15	0.15	0.50	0.25	0.25	-0.123	0.176	-0.070
1	20	0	0.8	0.5	0.5	0.15	0.15	0.15	0.50	0.25	0.50	-0.129	0.168	-0.092
1	20	0	0.8	0.5	0.5	0.15	0.15	0.15	0.50	0.50	0.00	-0.124	0.174	-0.101
1	20	0	0.8	0.5	0.5	0.15	0.15	0.15	0.50	0.50	0.25	-0.141	0.184	-0.083
1	20	0	0.8	0.5	0.5	0.15	0.15	0.15	0.50	0.50	0.50	-0.123	0.155	-0.085
1	20	0	0.8	0.5	0.5	0.30	0.30	0.30	0.00	0.00	0.00	-0.058	0.052	-0.129
1	20	0	0.8	0.5	0.5	0.30	0.30	0.30	0.00	0.00	0.25	-0.101	0.056	-0.140
1	20	0	0.8	0.5	0.5	0.30	0.30	0.30	0.00	0.00	0.50	-0.103	0.060	-0.125
1	20	0	0.8	0.5	0.5	0.30	0.30	0.30	0.00	0.25	0.00	-0.081	0.053	-0.147
1	20	0	0.8	0.5	0.5	0.30	0.30	0.30	0.00	0.25	0.25	-0.090	0.062	-0.136
1	20	0	0.8	0.5	0.5	0.30	0.30	0.30	0.00	0.25	0.50	-0.083	0.071	-0.155
1	20	0	0.8	0.5	0.5	0.30	0.30	0.30	0.00	0.50	0.00	-0.066	0.081	-0.181
1	20	0	0.8	0.5	0.5	0.30	0.30	0.30	0.00	0.50	0.25	-0.096	0.059	-0.178
1	20	0	0.8	0.5	0.5	0.30	0.30	0.30	0.00	0.50	0.50	-0.102	0.064	-0.185
1	20	0	0.8	0.5	0.5	0.30	0.30	0.30	0.25	0.00	0.00	-0.094	0.068	-0.128
1	20	0	0.8	0.5	0.5	0.30	0.30	0.30	0.25	0.00	0.25	-0.089	0.064	-0.116

(continued)

N	m_1	$\frac{m_2}{m_1}$	$E(C)$	$E(R)$	$E(U)$	σ_C	σ_R	σ_U	ρ_{CR}	ρ_{CU}	ρ_{RU}	Mean Bias c	Mean Bias r	Mean Bias u
1	20	0	0.8	0.5	0.5	0.30	0.30	0.30	0.25	0.00	0.50	-0.102	0.062	-0.118
1	20	0	0.8	0.5	0.5	0.30	0.30	0.30	0.25	0.25	0.00	-0.082	0.075	-0.145
1	20	0	0.8	0.5	0.5	0.30	0.30	0.30	0.25	0.25	0.25	-0.094	0.085	-0.148
1	20	0	0.8	0.5	0.5	0.30	0.30	0.30	0.25	0.25	0.50	-0.098	0.068	-0.148
1	20	0	0.8	0.5	0.5	0.30	0.30	0.30	0.25	0.50	0.00	-0.081	0.073	-0.186
1	20	0	0.8	0.5	0.5	0.30	0.30	0.30	0.25	0.50	0.25	-0.074	0.048	-0.162
1	20	0	0.8	0.5	0.5	0.30	0.30	0.30	0.25	0.50	0.50	-0.080	0.069	-0.175
1	20	0	0.8	0.5	0.5	0.30	0.30	0.30	0.50	0.00	0.00	-0.081	0.081	-0.111
1	20	0	0.8	0.5	0.5	0.30	0.30	0.30	0.50	0.00	0.25	-0.068	0.070	-0.089
1	20	0	0.8	0.5	0.5	0.30	0.30	0.30	0.50	0.00	0.50	-0.078	0.064	-0.122
1	20	0	0.8	0.5	0.5	0.30	0.30	0.30	0.50	0.25	0.00	-0.069	0.078	-0.128
1	20	0	0.8	0.5	0.5	0.30	0.30	0.30	0.50	0.25	0.25	-0.076	0.081	-0.133
1	20	0	0.8	0.5	0.5	0.30	0.30	0.30	0.50	0.25	0.50	-0.088	0.081	-0.148
1	20	0	0.8	0.5	0.5	0.30	0.30	0.30	0.50	0.50	0.00	-0.073	0.097	-0.194
1	20	0	0.8	0.5	0.5	0.30	0.30	0.30	0.50	0.50	0.25	-0.071	0.072	-0.160
1	20	0	0.8	0.5	0.5	0.30	0.30	0.30	0.50	0.50	0.50	-0.094	0.077	-0.184
1	20	0	0.8	0.8	0.5	0.00	0.00	0.00	0.00	0.00	0.00	-0.058	0.068	-0.036
1	20	0	0.8	0.8	0.5	0.15	0.15	0.15	0.00	0.00	0.00	-0.036	0.047	-0.017
1	20	0	0.8	0.8	0.5	0.15	0.15	0.15	0.00	0.00	0.25	-0.038	0.058	-0.017
1	20	0	0.8	0.8	0.5	0.15	0.15	0.15	0.00	0.00	0.50	-0.040	0.064	-0.026
1	20	0	0.8	0.8	0.5	0.15	0.15	0.15	0.00	0.25	0.00	-0.042	0.064	-0.036
1	20	0	0.8	0.8	0.5	0.15	0.15	0.15	0.00	0.25	0.25	-0.042	0.063	-0.039
1	20	0	0.8	0.8	0.5	0.15	0.15	0.15	0.00	0.25	0.50	-0.056	0.056	-0.045
1	20	0	0.8	0.8	0.5	0.15	0.15	0.15	0.00	0.50	0.00	-0.035	0.061	-0.046
1	20	0	0.8	0.8	0.5	0.15	0.15	0.15	0.00	0.50	0.25	-0.050	0.064	-0.055
1	20	0	0.8	0.8	0.5	0.15	0.15	0.15	0.00	0.50	0.50	-0.050	0.057	-0.049
1	20	0	0.8	0.8	0.5	0.15	0.15	0.15	0.25	0.00	0.00	-0.041	0.057	-0.017
1	20	0	0.8	0.8	0.5	0.15	0.15	0.15	0.25	0.00	0.25	-0.048	0.056	-0.009
1	20	0	0.8	0.8	0.5	0.15	0.15	0.15	0.25	0.00	0.50	-0.036	0.075	-0.015
1	20	0	0.8	0.8	0.5	0.15	0.15	0.15	0.25	0.25	0.00	-0.042	0.054	-0.038
1	20	0	0.8	0.8	0.5	0.15	0.15	0.15	0.25	0.25	0.25	-0.033	0.052	-0.019
1	20	0	0.8	0.8	0.5	0.15	0.15	0.15	0.25	0.25	0.50	-0.041	0.063	-0.050
1	20	0	0.8	0.8	0.5	0.15	0.15	0.15	0.25	0.50	0.00	-0.026	0.056	-0.026
1	20	0	0.8	0.8	0.5	0.15	0.15	0.15	0.25	0.50	0.25	-0.040	0.058	-0.032
1	20	0	0.8	0.8	0.5	0.15	0.15	0.15	0.25	0.50	0.50	-0.048	0.067	-0.047
1	20	0	0.8	0.8	0.5	0.15	0.15	0.15	0.50	0.00	0.00	-0.040	0.061	-0.025
1	20	0	0.8	0.8	0.5	0.15	0.15	0.15	0.50	0.00	0.25	-0.041	0.066	-0.028
1	20	0	0.8	0.8	0.5	0.15	0.15	0.15	0.50	0.00	0.50	-0.039	0.061	-0.011
1	20	0	0.8	0.8	0.5	0.15	0.15	0.15	0.50	0.25	0.00	-0.028	0.053	-0.019

(continued)

N	m_1	$\frac{m_2}{m_1}$	$E(C)$	$E(R)$	$E(U)$	σ_C	σ_R	σ_U	ρ_{CR}	ρ_{CU}	ρ_{RU}	Mean Bias c	Mean Bias r	Mean Bias u
1	20	0	0.8	0.8	0.5	0.15	0.15	0.15	0.50	0.25	0.25	-0.050	0.062	-0.036
1	20	0	0.8	0.8	0.5	0.15	0.15	0.15	0.50	0.25	0.50	-0.052	0.061	-0.025
1	20	0	0.8	0.8	0.5	0.15	0.15	0.15	0.50	0.50	0.00	-0.027	0.049	-0.022
1	20	0	0.8	0.8	0.5	0.15	0.15	0.15	0.50	0.50	0.25	-0.034	0.054	-0.033
1	20	0	0.8	0.8	0.5	0.15	0.15	0.15	0.50	0.50	0.50	-0.063	0.069	-0.046
1	20	0	0.8	0.8	0.5	0.30	0.30	0.30	0.00	0.00	0.00	-0.021	-0.032	-0.049
1	20	0	0.8	0.8	0.5	0.30	0.30	0.30	0.00	0.00	0.25	-0.040	-0.012	-0.047
1	20	0	0.8	0.8	0.5	0.30	0.30	0.30	0.00	0.00	0.50	-0.063	-0.041	-0.043
1	20	0	0.8	0.8	0.5	0.30	0.30	0.30	0.00	0.25	0.00	-0.020	-0.023	-0.081
1	20	0	0.8	0.8	0.5	0.30	0.30	0.30	0.00	0.25	0.25	-0.026	-0.030	-0.070
1	20	0	0.8	0.8	0.5	0.30	0.30	0.30	0.00	0.25	0.50	-0.025	-0.026	-0.070
1	20	0	0.8	0.8	0.5	0.30	0.30	0.30	0.00	0.50	0.00	-0.014	-0.026	-0.110
1	20	0	0.8	0.8	0.5	0.30	0.30	0.30	0.00	0.50	0.25	-0.019	-0.031	-0.113
1	20	0	0.8	0.8	0.5	0.30	0.30	0.30	0.00	0.50	0.50	-0.031	-0.015	-0.113
1	20	0	0.8	0.8	0.5	0.30	0.30	0.30	0.25	0.00	0.00	-0.003	-0.024	-0.037
1	20	0	0.8	0.8	0.5	0.30	0.30	0.30	0.25	0.00	0.25	-0.012	0.025	-0.051
1	20	0	0.8	0.8	0.5	0.30	0.30	0.30	0.25	0.00	0.50	-0.007	0.002	-0.060
1	20	0	0.8	0.8	0.5	0.30	0.30	0.30	0.25	0.25	0.00	-0.027	-0.004	-0.061
1	20	0	0.8	0.8	0.5	0.30	0.30	0.30	0.25	0.25	0.25	-0.034	0.003	-0.070
1	20	0	0.8	0.8	0.5	0.30	0.30	0.30	0.25	0.25	0.50	-0.017	-0.002	-0.046
1	20	0	0.8	0.8	0.5	0.30	0.30	0.30	0.25	0.50	0.00	-0.001	-0.007	-0.100
1	20	0	0.8	0.8	0.5	0.30	0.30	0.30	0.25	0.50	0.25	-0.021	0.003	-0.096
1	20	0	0.8	0.8	0.5	0.30	0.30	0.30	0.25	0.50	0.50	-0.028	0.007	-0.098
1	20	0	0.8	0.8	0.5	0.30	0.30	0.30	0.50	0.00	0.00	-0.012	0.006	-0.044
1	20	0	0.8	0.8	0.5	0.30	0.30	0.30	0.50	0.00	0.25	-0.014	0.005	-0.031
1	20	0	0.8	0.8	0.5	0.30	0.30	0.30	0.50	0.00	0.50	-0.027	0.011	-0.041
1	20	0	0.8	0.8	0.5	0.30	0.30	0.30	0.50	0.25	0.00	-0.007	0.001	-0.064
1	20	0	0.8	0.8	0.5	0.30	0.30	0.30	0.50	0.25	0.25	-0.019	-0.002	-0.050
1	20	0	0.8	0.8	0.5	0.30	0.30	0.30	0.50	0.25	0.50	-0.003	0.014	-0.074
1	20	0	0.8	0.8	0.5	0.30	0.30	0.30	0.50	0.50	0.00	0.004	0.006	-0.091
1	20	0	0.8	0.8	0.5	0.30	0.30	0.30	0.50	0.50	0.25	-0.014	-0.001	-0.111
1	20	0	0.8	0.8	0.5	0.30	0.30	0.30	0.50	0.50	0.50	-0.010	0.028	-0.105
1	20	1	0.2	0.2	0.5	0.00	0.00	0.00	0.00	0.00	0.00	-0.007	0.078	-0.002
1	20	1	0.2	0.2	0.5	0.15	0.15	0.15	0.00	0.00	0.00	0.014	0.010	0.007
1	20	1	0.2	0.2	0.5	0.15	0.15	0.15	0.00	0.00	0.25	0.018	-0.016	-0.004
1	20	1	0.2	0.2	0.5	0.15	0.15	0.15	0.00	0.00	0.50	0.011	0.021	-0.005
1	20	1	0.2	0.2	0.5	0.15	0.15	0.15	0.00	0.25	0.00	0.013	-0.016	0.008
1	20	1	0.2	0.2	0.5	0.15	0.15	0.15	0.00	0.25	0.25	0.021	0.005	0.010
1	20	1	0.2	0.2	0.5	0.15	0.15	0.15	0.00	0.25	0.50	0.021	0.004	0.006

(continued)

												Mean Bias		
N	m_1	$\frac{m_2}{m_1}$	$E(C)$	$E(R)$	$E(U)$	σ_C	σ_R	σ_U	ρ_{CR}	ρ_{CU}	ρ_{RU}	c	r	u
1	20	1	0.2	0.2	0.5	0.15	0.15	0.15	0.00	0.50	0.00	0.015	-0.001	0.007
1	20	1	0.2	0.2	0.5	0.15	0.15	0.15	0.00	0.50	0.25	0.012	0.010	0.004
1	20	1	0.2	0.2	0.5	0.15	0.15	0.15	0.00	0.50	0.50	0.025	0.011	0.012
1	20	1	0.2	0.2	0.5	0.15	0.15	0.15	0.25	0.00	0.00	0.024	0.016	0.016
1	20	1	0.2	0.2	0.5	0.15	0.15	0.15	0.25	0.00	0.25	0.007	0.023	0.014
1	20	1	0.2	0.2	0.5	0.15	0.15	0.15	0.25	0.00	0.50	0.014	0.005	0.004
1	20	1	0.2	0.2	0.5	0.15	0.15	0.15	0.25	0.25	0.00	0.007	-0.001	0.003
1	20	1	0.2	0.2	0.5	0.15	0.15	0.15	0.25	0.25	0.25	0.020	0.022	0.014
1	20	1	0.2	0.2	0.5	0.15	0.15	0.15	0.25	0.25	0.50	0.011	0.008	0.003
1	20	1	0.2	0.2	0.5	0.15	0.15	0.15	0.25	0.50	0.00	0.014	0.008	0.008
1	20	1	0.2	0.2	0.5	0.15	0.15	0.15	0.25	0.50	0.25	0.018	0.004	0.006
1	20	1	0.2	0.2	0.5	0.15	0.15	0.15	0.25	0.50	0.50	0.017	-0.005	0.005
1	20	1	0.2	0.2	0.5	0.15	0.15	0.15	0.50	0.00	0.00	0.019	0.027	-0.001
1	20	1	0.2	0.2	0.5	0.15	0.15	0.15	0.50	0.00	0.25	0.015	0.017	0.008
1	20	1	0.2	0.2	0.5	0.15	0.15	0.15	0.50	0.00	0.50	0.012	0.006	0.002
1	20	1	0.2	0.2	0.5	0.15	0.15	0.15	0.50	0.25	0.00	0.017	0.020	0.007
1	20	1	0.2	0.2	0.5	0.15	0.15	0.15	0.50	0.25	0.25	0.018	0.009	0.011
1	20	1	0.2	0.2	0.5	0.15	0.15	0.15	0.50	0.25	0.50	0.014	0.008	0.000
1	20	1	0.2	0.2	0.5	0.15	0.15	0.15	0.50	0.50	0.00	0.021	0.010	0.007
1	20	1	0.2	0.2	0.5	0.15	0.15	0.15	0.50	0.50	0.25	0.014	0.008	-0.002
1	20	1	0.2	0.2	0.5	0.15	0.15	0.15	0.50	0.50	0.50	0.018	0.013	0.012
1	20	1	0.2	0.2	0.5	0.30	0.30	0.30	0.00	0.00	0.00	0.062	-0.071	-0.003
1	20	1	0.2	0.2	0.5	0.30	0.30	0.30	0.00	0.00	0.25	0.049	-0.062	0.015
1	20	1	0.2	0.2	0.5	0.30	0.30	0.30	0.00	0.00	0.50	0.065	-0.063	-0.003
1	20	1	0.2	0.2	0.5	0.30	0.30	0.30	0.00	0.25	0.00	0.039	-0.077	0.014
1	20	1	0.2	0.2	0.5	0.30	0.30	0.30	0.00	0.25	0.25	0.056	-0.080	-0.006
1	20	1	0.2	0.2	0.5	0.30	0.30	0.30	0.00	0.25	0.50	0.048	-0.053	0.029
1	20	1	0.2	0.2	0.5	0.30	0.30	0.30	0.00	0.50	0.00	0.075	-0.082	0.009
1	20	1	0.2	0.2	0.5	0.30	0.30	0.30	0.00	0.50	0.25	0.060	-0.088	-0.005
1	20	1	0.2	0.2	0.5	0.30	0.30	0.30	0.00	0.50	0.50	0.063	-0.084	0.009
1	20	1	0.2	0.2	0.5	0.30	0.30	0.30	0.25	0.00	0.00	0.060	-0.018	0.004
1	20	1	0.2	0.2	0.5	0.30	0.30	0.30	0.25	0.00	0.25	0.043	-0.047	0.015
1	20	1	0.2	0.2	0.5	0.30	0.30	0.30	0.25	0.00	0.50	0.040	-0.047	0.017
1	20	1	0.2	0.2	0.5	0.30	0.30	0.30	0.25	0.25	0.00	0.052	-0.058	0.018
1	20	1	0.2	0.2	0.5	0.30	0.30	0.30	0.25	0.25	0.25	0.064	-0.052	0.008
1	20	1	0.2	0.2	0.5	0.30	0.30	0.30	0.25	0.25	0.50	0.035	-0.059	0.009
1	20	1	0.2	0.2	0.5	0.30	0.30	0.30	0.25	0.50	0.00	0.082	-0.038	0.010
1	20	1	0.2	0.2	0.5	0.30	0.30	0.30	0.25	0.50	0.25	0.063	-0.044	0.009
1	20	1	0.2	0.2	0.5	0.30	0.30	0.30	0.25	0.50	0.50	0.065	-0.041	0.022

(continued)

												Mean Bias		
N	m_1	$\frac{m_2}{m_1}$	$E(C)$	$E(R)$	$E(U)$	σ_C	σ_R	σ_U	ρ_{CR}	ρ_{CU}	ρ_{RU}	c	r	u
1	20	1	0.2	0.2	0.5	0.30	0.30	0.30	0.50	0.00	0.00	0.058	-0.007	0.015
1	20	1	0.2	0.2	0.5	0.30	0.30	0.30	0.50	0.00	0.25	0.043	-0.030	0.007
1	20	1	0.2	0.2	0.5	0.30	0.30	0.30	0.50	0.00	0.50	0.031	-0.024	0.010
1	20	1	0.2	0.2	0.5	0.30	0.30	0.30	0.50	0.25	0.00	0.059	-0.028	0.007
1	20	1	0.2	0.2	0.5	0.30	0.30	0.30	0.50	0.25	0.25	0.060	-0.030	-0.026
1	20	1	0.2	0.2	0.5	0.30	0.30	0.30	0.50	0.25	0.50	0.054	-0.043	-0.003
1	20	1	0.2	0.2	0.5	0.30	0.30	0.30	0.50	0.50	0.00	0.058	-0.048	0.017
1	20	1	0.2	0.2	0.5	0.30	0.30	0.30	0.50	0.50	0.25	0.064	-0.030	0.009
1	20	1	0.2	0.2	0.5	0.30	0.30	0.30	0.50	0.50	0.50	0.050	-0.026	0.014
1	20	1	0.2	0.5	0.5	0.00	0.00	0.00	0.00	0.00	0.00	0.011	0.042	0.007
1	20	1	0.2	0.5	0.5	0.15	0.15	0.15	0.00	0.00	0.00	0.020	-0.033	0.012
1	20	1	0.2	0.5	0.5	0.15	0.15	0.15	0.00	0.00	0.25	0.012	-0.055	0.011
1	20	1	0.2	0.5	0.5	0.15	0.15	0.15	0.00	0.00	0.50	0.024	-0.042	0.012
1	20	1	0.2	0.5	0.5	0.15	0.15	0.15	0.00	0.25	0.00	0.025	-0.048	0.003
1	20	1	0.2	0.5	0.5	0.15	0.15	0.15	0.00	0.25	0.25	0.022	-0.050	0.002
1	20	1	0.2	0.5	0.5	0.15	0.15	0.15	0.00	0.25	0.50	0.026	-0.055	0.002
1	20	1	0.2	0.5	0.5	0.15	0.15	0.15	0.00	0.50	0.00	0.023	-0.063	0.009
1	20	1	0.2	0.5	0.5	0.15	0.15	0.15	0.00	0.50	0.25	0.020	-0.058	0.007
1	20	1	0.2	0.5	0.5	0.15	0.15	0.15	0.00	0.50	0.50	0.014	-0.056	0.005
1	20	1	0.2	0.5	0.5	0.15	0.15	0.15	0.25	0.00	0.00	0.023	-0.049	0.005
1	20	1	0.2	0.5	0.5	0.15	0.15	0.15	0.25	0.00	0.25	0.023	-0.045	0.018
1	20	1	0.2	0.5	0.5	0.15	0.15	0.15	0.25	0.00	0.50	0.012	-0.060	0.007
1	20	1	0.2	0.5	0.5	0.15	0.15	0.15	0.25	0.25	0.00	0.028	-0.062	0.017
1	20	1	0.2	0.5	0.5	0.15	0.15	0.15	0.25	0.25	0.25	0.015	-0.070	0.005
1	20	1	0.2	0.5	0.5	0.15	0.15	0.15	0.25	0.25	0.50	0.026	-0.046	0.007
1	20	1	0.2	0.5	0.5	0.15	0.15	0.15	0.25	0.50	0.00	0.024	-0.049	0.013
1	20	1	0.2	0.5	0.5	0.15	0.15	0.15	0.25	0.50	0.25	0.028	-0.051	0.010
1	20	1	0.2	0.5	0.5	0.15	0.15	0.15	0.25	0.50	0.50	0.032	-0.044	0.020
1	20	1	0.2	0.5	0.5	0.15	0.15	0.15	0.50	0.00	0.00	0.024	-0.039	0.015
1	20	1	0.2	0.5	0.5	0.15	0.15	0.15	0.50	0.00	0.25	0.013	-0.053	0.010
1	20	1	0.2	0.5	0.5	0.15	0.15	0.15	0.50	0.00	0.50	0.022	-0.064	0.010
1	20	1	0.2	0.5	0.5	0.15	0.15	0.15	0.50	0.25	0.00	0.029	-0.038	0.010
1	20	1	0.2	0.5	0.5	0.15	0.15	0.15	0.50	0.25	0.25	0.018	-0.042	0.007
1	20	1	0.2	0.5	0.5	0.15	0.15	0.15	0.50	0.25	0.50	0.027	-0.040	0.005
1	20	1	0.2	0.5	0.5	0.15	0.15	0.15	0.50	0.50	0.00	0.027	-0.028	-0.001
1	20	1	0.2	0.5	0.5	0.15	0.15	0.15	0.50	0.50	0.25	0.033	-0.062	0.009
1	20	1	0.2	0.5	0.5	0.15	0.15	0.15	0.50	0.50	0.50	0.030	-0.056	0.010
1	20	1	0.2	0.5	0.5	0.30	0.30	0.30	0.00	0.00	0.00	0.056	-0.218	0.018
1	20	1	0.2	0.5	0.5	0.30	0.30	0.30	0.00	0.00	0.25	0.042	-0.244	0.009

(continued)

N	m_1	$\frac{m_2}{m_1}$	$E(C)$	$E(R)$	$E(U)$	σ_C	σ_R	σ_U	ρ_{CR}	ρ_{CU}	ρ_{RU}	Mean Bias c	Mean Bias r	Mean Bias u
1	20	1	0.2	0.5	0.5	0.30	0.30	0.30	0.00	0.00	0.50	0.052	-0.233	0.011
1	20	1	0.2	0.5	0.5	0.30	0.30	0.30	0.00	0.25	0.00	0.073	-0.233	0.001
1	20	1	0.2	0.5	0.5	0.30	0.30	0.30	0.00	0.25	0.25	0.061	-0.201	0.023
1	20	1	0.2	0.5	0.5	0.30	0.30	0.30	0.00	0.25	0.50	0.058	-0.239	-0.004
1	20	1	0.2	0.5	0.5	0.30	0.30	0.30	0.00	0.50	0.00	0.063	-0.241	0.019
1	20	1	0.2	0.5	0.5	0.30	0.30	0.30	0.00	0.50	0.25	0.064	-0.237	0.000
1	20	1	0.2	0.5	0.5	0.30	0.30	0.30	0.00	0.50	0.50	0.063	-0.248	-0.001
1	20	1	0.2	0.5	0.5	0.30	0.30	0.30	0.25	0.00	0.00	0.068	-0.201	0.008
1	20	1	0.2	0.5	0.5	0.30	0.30	0.30	0.25	0.00	0.25	0.060	-0.195	0.004
1	20	1	0.2	0.5	0.5	0.30	0.30	0.30	0.25	0.00	0.50	0.046	-0.189	0.010
1	20	1	0.2	0.5	0.5	0.30	0.30	0.30	0.25	0.25	0.00	0.067	-0.219	-0.001
1	20	1	0.2	0.5	0.5	0.30	0.30	0.30	0.25	0.25	0.25	0.076	-0.210	0.007
1	20	1	0.2	0.5	0.5	0.30	0.30	0.30	0.25	0.25	0.50	0.059	-0.188	0.010
1	20	1	0.2	0.5	0.5	0.30	0.30	0.30	0.25	0.50	0.00	0.066	-0.220	0.006
1	20	1	0.2	0.5	0.5	0.30	0.30	0.30	0.25	0.50	0.25	0.062	-0.212	0.014
1	20	1	0.2	0.5	0.5	0.30	0.30	0.30	0.25	0.50	0.50	0.083	-0.203	0.015
1	20	1	0.2	0.5	0.5	0.30	0.30	0.30	0.50	0.00	0.00	0.066	-0.176	0.019
1	20	1	0.2	0.5	0.5	0.30	0.30	0.30	0.50	0.00	0.25	0.060	-0.169	0.008
1	20	1	0.2	0.5	0.5	0.30	0.30	0.30	0.50	0.00	0.50	0.041	-0.155	0.026
1	20	1	0.2	0.5	0.5	0.30	0.30	0.30	0.50	0.25	0.00	0.044	-0.193	0.009
1	20	1	0.2	0.5	0.5	0.30	0.30	0.30	0.50	0.25	0.25	0.078	-0.169	0.031
1	20	1	0.2	0.5	0.5	0.30	0.30	0.30	0.50	0.25	0.50	0.047	-0.185	0.007
1	20	1	0.2	0.5	0.5	0.30	0.30	0.30	0.50	0.50	0.00	0.073	-0.191	-0.002
1	20	1	0.2	0.5	0.5	0.30	0.30	0.30	0.50	0.50	0.25	0.061	-0.193	0.010
1	20	1	0.2	0.5	0.5	0.30	0.30	0.30	0.50	0.50	0.50	0.070	-0.185	-0.002
1	20	1	0.2	0.8	0.5	0.00	0.00	0.00	0.00	0.00	0.00	0.036	-0.068	0.020
1	20	1	0.2	0.8	0.5	0.15	0.15	0.15	0.00	0.00	0.00	0.040	-0.153	0.009
1	20	1	0.2	0.8	0.5	0.15	0.15	0.15	0.00	0.00	0.25	0.043	-0.135	0.018
1	20	1	0.2	0.8	0.5	0.15	0.15	0.15	0.00	0.00	0.50	0.032	-0.164	0.017
1	20	1	0.2	0.8	0.5	0.15	0.15	0.15	0.00	0.25	0.00	0.029	-0.174	0.012
1	20	1	0.2	0.8	0.5	0.15	0.15	0.15	0.00	0.25	0.25	0.033	-0.185	0.017
1	20	1	0.2	0.8	0.5	0.15	0.15	0.15	0.00	0.25	0.50	0.033	-0.155	0.024
1	20	1	0.2	0.8	0.5	0.15	0.15	0.15	0.00	0.50	0.00	0.043	-0.186	0.010
1	20	1	0.2	0.8	0.5	0.15	0.15	0.15	0.00	0.50	0.25	0.045	-0.159	0.018
1	20	1	0.2	0.8	0.5	0.15	0.15	0.15	0.00	0.50	0.50	0.036	-0.207	0.010
1	20	1	0.2	0.8	0.5	0.15	0.15	0.15	0.25	0.00	0.00	0.042	-0.170	0.017
1	20	1	0.2	0.8	0.5	0.15	0.15	0.15	0.25	0.00	0.25	0.040	-0.185	0.018
1	20	1	0.2	0.8	0.5	0.15	0.15	0.15	0.25	0.00	0.50	0.040	-0.153	0.022
1	20	1	0.2	0.8	0.5	0.15	0.15	0.15	0.25	0.25	0.00	0.044	-0.169	0.024

(continued)

												Mean Bias		
N	m_1	$\frac{m_2}{m_1}$	$E(C)$	$E(R)$	$E(U)$	σ_C	σ_R	σ_U	ρ_{CR}	ρ_{CU}	ρ_{RU}	c	r	u
1	20	1	0.2	0.8	0.5	0.15	0.15	0.15	0.25	0.25	0.25	0.051	-0.154	0.024
1	20	1	0.2	0.8	0.5	0.15	0.15	0.15	0.25	0.25	0.50	0.045	-0.152	0.015
1	20	1	0.2	0.8	0.5	0.15	0.15	0.15	0.25	0.50	0.00	0.037	-0.138	0.009
1	20	1	0.2	0.8	0.5	0.15	0.15	0.15	0.25	0.50	0.25	0.042	-0.173	0.011
1	20	1	0.2	0.8	0.5	0.15	0.15	0.15	0.25	0.50	0.50	0.040	-0.163	0.021
1	20	1	0.2	0.8	0.5	0.15	0.15	0.15	0.50	0.00	0.00	0.042	-0.148	0.017
1	20	1	0.2	0.8	0.5	0.15	0.15	0.15	0.50	0.00	0.25	0.049	-0.127	0.013
1	20	1	0.2	0.8	0.5	0.15	0.15	0.15	0.50	0.00	0.50	0.033	-0.149	0.015
1	20	1	0.2	0.8	0.5	0.15	0.15	0.15	0.50	0.25	0.00	0.046	-0.167	0.019
1	20	1	0.2	0.8	0.5	0.15	0.15	0.15	0.50	0.25	0.25	0.056	-0.151	0.025
1	20	1	0.2	0.8	0.5	0.15	0.15	0.15	0.50	0.25	0.50	0.039	-0.150	0.017
1	20	1	0.2	0.8	0.5	0.15	0.15	0.15	0.50	0.50	0.00	0.044	-0.160	0.016
1	20	1	0.2	0.8	0.5	0.15	0.15	0.15	0.50	0.50	0.25	0.044	-0.163	0.018
1	20	1	0.2	0.8	0.5	0.15	0.15	0.15	0.50	0.50	0.50	0.043	-0.156	0.016
1	20	1	0.2	0.8	0.5	0.30	0.30	0.30	0.00	0.00	0.00	0.065	-0.417	0.006
1	20	1	0.2	0.8	0.5	0.30	0.30	0.30	0.00	0.00	0.25	0.074	-0.398	0.010
1	20	1	0.2	0.8	0.5	0.30	0.30	0.30	0.00	0.00	0.50	0.087	-0.371	0.035
1	20	1	0.2	0.8	0.5	0.30	0.30	0.30	0.00	0.25	0.00	0.071	-0.427	0.010
1	20	1	0.2	0.8	0.5	0.30	0.30	0.30	0.00	0.25	0.25	0.068	-0.424	0.026
1	20	1	0.2	0.8	0.5	0.30	0.30	0.30	0.00	0.25	0.50	0.076	-0.398	0.008
1	20	1	0.2	0.8	0.5	0.30	0.30	0.30	0.00	0.50	0.00	0.084	-0.417	0.005
1	20	1	0.2	0.8	0.5	0.30	0.30	0.30	0.00	0.50	0.25	0.068	-0.411	0.013
1	20	1	0.2	0.8	0.5	0.30	0.30	0.30	0.00	0.50	0.50	0.080	-0.400	0.009
1	20	1	0.2	0.8	0.5	0.30	0.30	0.30	0.25	0.00	0.00	0.076	-0.369	0.014
1	20	1	0.2	0.8	0.5	0.30	0.30	0.30	0.25	0.00	0.25	0.063	-0.386	-0.004
1	20	1	0.2	0.8	0.5	0.30	0.30	0.30	0.25	0.00	0.50	0.074	-0.383	0.004
1	20	1	0.2	0.8	0.5	0.30	0.30	0.30	0.25	0.25	0.00	0.071	-0.403	0.003
1	20	1	0.2	0.8	0.5	0.30	0.30	0.30	0.25	0.25	0.25	0.082	-0.387	0.019
1	20	1	0.2	0.8	0.5	0.30	0.30	0.30	0.25	0.25	0.50	0.073	-0.385	0.014
1	20	1	0.2	0.8	0.5	0.30	0.30	0.30	0.25	0.50	0.00	0.075	-0.410	0.012
1	20	1	0.2	0.8	0.5	0.30	0.30	0.30	0.25	0.50	0.25	0.079	-0.405	0.009
1	20	1	0.2	0.8	0.5	0.30	0.30	0.30	0.25	0.50	0.50	0.066	-0.382	0.001
1	20	1	0.2	0.8	0.5	0.30	0.30	0.30	0.50	0.00	0.00	0.069	-0.356	0.015
1	20	1	0.2	0.8	0.5	0.30	0.30	0.30	0.50	0.00	0.25	0.069	-0.362	0.006
1	20	1	0.2	0.8	0.5	0.30	0.30	0.30	0.50	0.00	0.50	0.077	-0.371	0.007
1	20	1	0.2	0.8	0.5	0.30	0.30	0.30	0.50	0.25	0.00	0.071	-0.385	0.015
1	20	1	0.2	0.8	0.5	0.30	0.30	0.30	0.50	0.25	0.25	0.077	-0.345	0.018
1	20	1	0.2	0.8	0.5	0.30	0.30	0.30	0.50	0.25	0.50	0.087	-0.373	0.025
1	20	1	0.2	0.8	0.5	0.30	0.30	0.30	0.50	0.50	0.00	0.075	-0.373	0.006

(continued)

												Mean Bias		
N	m_1	$\frac{m_2}{m_1}$	$E(C)$	$E(R)$	$E(U)$	σ_C	σ_R	σ_U	ρ_{CR}	ρ_{CU}	ρ_{RU}	c	r	u
1	20	1	0.2	0.8	0.5	0.30	0.30	0.30	0.50	0.50	0.25	0.079	-0.376	0.010
1	20	1	0.2	0.8	0.5	0.30	0.30	0.30	0.50	0.50	0.50	0.088	-0.361	0.018
1	20	1	0.5	0.2	0.5	0.00	0.00	0.00	0.00	0.00	0.00	-0.008	0.027	0.000
1	20	1	0.5	0.2	0.5	0.15	0.15	0.15	0.00	0.00	0.00	-0.025	0.040	-0.008
1	20	1	0.5	0.2	0.5	0.15	0.15	0.15	0.00	0.00	0.25	-0.015	0.028	0.003
1	20	1	0.5	0.2	0.5	0.15	0.15	0.15	0.00	0.00	0.50	-0.007	0.014	0.000
1	20	1	0.5	0.2	0.5	0.15	0.15	0.15	0.00	0.25	0.00	-0.011	0.035	0.001
1	20	1	0.5	0.2	0.5	0.15	0.15	0.15	0.00	0.25	0.25	-0.007	0.022	-0.002
1	20	1	0.5	0.2	0.5	0.15	0.15	0.15	0.00	0.25	0.50	-0.015	0.040	0.001
1	20	1	0.5	0.2	0.5	0.15	0.15	0.15	0.00	0.50	0.00	-0.026	0.029	-0.008
1	20	1	0.5	0.2	0.5	0.15	0.15	0.15	0.00	0.50	0.25	-0.012	0.032	0.001
1	20	1	0.5	0.2	0.5	0.15	0.15	0.15	0.00	0.50	0.50	-0.021	0.055	-0.007
1	20	1	0.5	0.2	0.5	0.15	0.15	0.15	0.25	0.00	0.00	-0.018	0.039	-0.005
1	20	1	0.5	0.2	0.5	0.15	0.15	0.15	0.25	0.00	0.25	-0.018	0.023	-0.004
1	20	1	0.5	0.2	0.5	0.15	0.15	0.15	0.25	0.00	0.50	-0.012	0.025	0.006
1	20	1	0.5	0.2	0.5	0.15	0.15	0.15	0.25	0.25	0.00	-0.025	0.032	-0.001
1	20	1	0.5	0.2	0.5	0.15	0.15	0.15	0.25	0.25	0.25	-0.006	0.021	-0.007
1	20	1	0.5	0.2	0.5	0.15	0.15	0.15	0.25	0.25	0.50	-0.016	0.046	0.000
1	20	1	0.5	0.2	0.5	0.15	0.15	0.15	0.25	0.50	0.00	-0.014	0.026	0.001
1	20	1	0.5	0.2	0.5	0.15	0.15	0.15	0.25	0.50	0.25	-0.014	0.036	0.000
1	20	1	0.5	0.2	0.5	0.15	0.15	0.15	0.25	0.50	0.50	-0.005	0.034	0.002
1	20	1	0.5	0.2	0.5	0.15	0.15	0.15	0.50	0.00	0.00	-0.008	0.039	0.005
1	20	1	0.5	0.2	0.5	0.15	0.15	0.15	0.50	0.00	0.25	-0.014	0.029	0.005
1	20	1	0.5	0.2	0.5	0.15	0.15	0.15	0.50	0.00	0.50	-0.021	0.030	0.004
1	20	1	0.5	0.2	0.5	0.15	0.15	0.15	0.50	0.25	0.00	-0.007	0.022	0.001
1	20	1	0.5	0.2	0.5	0.15	0.15	0.15	0.50	0.25	0.25	-0.011	0.034	0.001
1	20	1	0.5	0.2	0.5	0.15	0.15	0.15	0.50	0.25	0.50	-0.018	0.022	0.008
1	20	1	0.5	0.2	0.5	0.15	0.15	0.15	0.50	0.50	0.00	-0.003	0.018	0.000
1	20	1	0.5	0.2	0.5	0.15	0.15	0.15	0.50	0.50	0.25	-0.021	0.033	0.003
1	20	1	0.5	0.2	0.5	0.15	0.15	0.15	0.50	0.50	0.50	-0.010	0.032	0.002
1	20	1	0.5	0.2	0.5	0.30	0.30	0.30	0.00	0.00	0.00	0.017	0.001	0.019
1	20	1	0.5	0.2	0.5	0.30	0.30	0.30	0.00	0.00	0.25	0.006	-0.017	-0.009
1	20	1	0.5	0.2	0.5	0.30	0.30	0.30	0.00	0.00	0.50	-0.008	0.005	0.000
1	20	1	0.5	0.2	0.5	0.30	0.30	0.30	0.00	0.25	0.00	0.008	0.008	0.004
1	20	1	0.5	0.2	0.5	0.30	0.30	0.30	0.00	0.25	0.25	0.005	0.009	0.003
1	20	1	0.5	0.2	0.5	0.30	0.30	0.30	0.00	0.25	0.50	0.002	-0.004	0.005
1	20	1	0.5	0.2	0.5	0.30	0.30	0.30	0.00	0.50	0.00	0.024	-0.027	-0.004
1	20	1	0.5	0.2	0.5	0.30	0.30	0.30	0.00	0.50	0.25	0.040	0.000	-0.001
1	20	1	0.5	0.2	0.5	0.30	0.30	0.30	0.00	0.50	0.50	0.013	-0.006	-0.002

(continued)

												Mean Bias		
N	m_1	$\frac{m_2}{m_1}$	$E(C)$	$E(R)$	$E(U)$	σ_C	σ_R	σ_U	ρ_{CR}	ρ_{CU}	ρ_{RU}	c	r	u
1	20	1	0.5	0.2	0.5	0.30	0.30	0.30	0.25	0.00	0.00	0.004	0.020	-0.003
1	20	1	0.5	0.2	0.5	0.30	0.30	0.30	0.25	0.00	0.25	-0.011	-0.003	-0.009
1	20	1	0.5	0.2	0.5	0.30	0.30	0.30	0.25	0.00	0.50	-0.015	0.004	-0.006
1	20	1	0.5	0.2	0.5	0.30	0.30	0.30	0.25	0.25	0.00	0.023	0.004	0.009
1	20	1	0.5	0.2	0.5	0.30	0.30	0.30	0.25	0.25	0.25	0.006	0.002	0.007
1	20	1	0.5	0.2	0.5	0.30	0.30	0.30	0.25	0.25	0.50	0.007	-0.007	0.013
1	20	1	0.5	0.2	0.5	0.30	0.30	0.30	0.25	0.50	0.00	0.036	0.039	0.013
1	20	1	0.5	0.2	0.5	0.30	0.30	0.30	0.25	0.50	0.25	0.020	0.006	-0.005
1	20	1	0.5	0.2	0.5	0.30	0.30	0.30	0.25	0.50	0.50	-0.003	0.016	-0.003
1	20	1	0.5	0.2	0.5	0.30	0.30	0.30	0.50	0.00	0.00	0.005	0.029	0.004
1	20	1	0.5	0.2	0.5	0.30	0.30	0.30	0.50	0.00	0.25	0.013	0.028	0.018
1	20	1	0.5	0.2	0.5	0.30	0.30	0.30	0.50	0.00	0.50	0.003	-0.002	-0.004
1	20	1	0.5	0.2	0.5	0.30	0.30	0.30	0.50	0.25	0.00	0.004	0.012	0.018
1	20	1	0.5	0.2	0.5	0.30	0.30	0.30	0.50	0.25	0.25	0.032	0.012	0.008
1	20	1	0.5	0.2	0.5	0.30	0.30	0.30	0.50	0.25	0.50	0.023	0.017	0.013
1	20	1	0.5	0.2	0.5	0.30	0.30	0.30	0.50	0.50	0.00	0.028	0.002	-0.009
1	20	1	0.5	0.2	0.5	0.30	0.30	0.30	0.50	0.50	0.25	0.021	0.012	-0.007
1	20	1	0.5	0.2	0.5	0.30	0.30	0.30	0.50	0.50	0.50	0.014	0.031	0.023
1	20	1	0.5	0.5	0.5	0.00	0.00	0.00	0.00	0.00	0.00	-0.014	0.039	-0.003
1	20	1	0.5	0.5	0.5	0.15	0.15	0.15	0.00	0.00	0.00	-0.012	0.041	-0.004
1	20	1	0.5	0.5	0.5	0.15	0.15	0.15	0.00	0.00	0.25	-0.008	0.049	0.009
1	20	1	0.5	0.5	0.5	0.15	0.15	0.15	0.00	0.00	0.50	-0.012	0.028	0.003
1	20	1	0.5	0.5	0.5	0.15	0.15	0.15	0.00	0.25	0.00	-0.008	0.045	-0.005
1	20	1	0.5	0.5	0.5	0.15	0.15	0.15	0.00	0.25	0.25	-0.010	0.054	-0.004
1	20	1	0.5	0.5	0.5	0.15	0.15	0.15	0.00	0.25	0.50	-0.007	0.038	-0.012
1	20	1	0.5	0.5	0.5	0.15	0.15	0.15	0.00	0.50	0.00	-0.012	0.037	-0.002
1	20	1	0.5	0.5	0.5	0.15	0.15	0.15	0.00	0.50	0.25	-0.003	0.034	0.003
1	20	1	0.5	0.5	0.5	0.15	0.15	0.15	0.00	0.50	0.50	-0.002	0.036	0.005
1	20	1	0.5	0.5	0.5	0.15	0.15	0.15	0.25	0.00	0.00	-0.018	0.058	-0.009
1	20	1	0.5	0.5	0.5	0.15	0.15	0.15	0.25	0.00	0.25	-0.010	0.044	-0.014
1	20	1	0.5	0.5	0.5	0.15	0.15	0.15	0.25	0.00	0.50	0.001	0.041	-0.001
1	20	1	0.5	0.5	0.5	0.15	0.15	0.15	0.25	0.25	0.00	-0.003	0.041	-0.007
1	20	1	0.5	0.5	0.5	0.15	0.15	0.15	0.25	0.25	0.25	0.000	0.039	0.011
1	20	1	0.5	0.5	0.5	0.15	0.15	0.15	0.25	0.25	0.50	-0.009	0.051	0.003
1	20	1	0.5	0.5	0.5	0.15	0.15	0.15	0.25	0.50	0.00	0.001	0.043	0.010
1	20	1	0.5	0.5	0.5	0.15	0.15	0.15	0.25	0.50	0.25	0.001	0.045	-0.002
1	20	1	0.5	0.5	0.5	0.15	0.15	0.15	0.25	0.50	0.50	0.000	0.054	0.014
1	20	1	0.5	0.5	0.5	0.15	0.15	0.15	0.50	0.00	0.00	-0.003	0.041	0.010
1	20	1	0.5	0.5	0.5	0.15	0.15	0.15	0.50	0.00	0.25	-0.008	0.044	0.007

(continued)

												Mean Bias		
N	m_1	$\frac{m_2}{m_1}$	$E(C)$	$E(R)$	$E(U)$	σ_C	σ_R	σ_U	ρ_{CR}	ρ_{CU}	ρ_{RU}	c	r	u
1	20	1	0.5	0.5	0.5	0.15	0.15	0.15	0.50	0.00	0.50	-0.005	0.049	0.004
1	20	1	0.5	0.5	0.5	0.15	0.15	0.15	0.50	0.25	0.00	-0.004	0.036	0.005
1	20	1	0.5	0.5	0.5	0.15	0.15	0.15	0.50	0.25	0.25	-0.004	0.050	0.007
1	20	1	0.5	0.5	0.5	0.15	0.15	0.15	0.50	0.25	0.50	0.000	0.050	0.000
1	20	1	0.5	0.5	0.5	0.15	0.15	0.15	0.50	0.50	0.00	-0.010	0.050	-0.006
1	20	1	0.5	0.5	0.5	0.15	0.15	0.15	0.50	0.50	0.25	-0.007	0.043	0.001
1	20	1	0.5	0.5	0.5	0.15	0.15	0.15	0.50	0.50	0.50	0.001	0.051	-0.006
1	20	1	0.5	0.5	0.5	0.30	0.30	0.30	0.00	0.00	0.00	0.025	-0.014	-0.006
1	20	1	0.5	0.5	0.5	0.30	0.30	0.30	0.00	0.00	0.25	0.007	-0.033	0.010
1	20	1	0.5	0.5	0.5	0.30	0.30	0.30	0.00	0.00	0.50	0.026	-0.014	0.020
1	20	1	0.5	0.5	0.5	0.30	0.30	0.30	0.00	0.25	0.00	0.023	-0.015	0.007
1	20	1	0.5	0.5	0.5	0.30	0.30	0.30	0.00	0.25	0.25	0.023	-0.022	-0.007
1	20	1	0.5	0.5	0.5	0.30	0.30	0.30	0.00	0.25	0.50	0.015	-0.008	0.002
1	20	1	0.5	0.5	0.5	0.30	0.30	0.30	0.00	0.50	0.00	0.039	-0.010	0.011
1	20	1	0.5	0.5	0.5	0.30	0.30	0.30	0.00	0.50	0.25	0.027	-0.035	0.011
1	20	1	0.5	0.5	0.5	0.30	0.30	0.30	0.00	0.50	0.50	0.020	-0.023	0.017
1	20	1	0.5	0.5	0.5	0.30	0.30	0.30	0.25	0.00	0.00	0.008	-0.022	0.004
1	20	1	0.5	0.5	0.5	0.30	0.30	0.30	0.25	0.00	0.25	0.017	-0.015	0.014
1	20	1	0.5	0.5	0.5	0.30	0.30	0.30	0.25	0.00	0.50	0.011	-0.014	0.001
1	20	1	0.5	0.5	0.5	0.30	0.30	0.30	0.25	0.25	0.00	0.036	-0.023	0.006
1	20	1	0.5	0.5	0.5	0.30	0.30	0.30	0.25	0.25	0.25	0.033	-0.027	0.001
1	20	1	0.5	0.5	0.5	0.30	0.30	0.30	0.25	0.25	0.50	0.031	0.003	0.015
1	20	1	0.5	0.5	0.5	0.30	0.30	0.30	0.25	0.50	0.00	0.021	-0.014	0.003
1	20	1	0.5	0.5	0.5	0.30	0.30	0.30	0.25	0.50	0.25	0.055	0.003	0.024
1	20	1	0.5	0.5	0.5	0.30	0.30	0.30	0.25	0.50	0.50	0.028	-0.020	-0.007
1	20	1	0.5	0.5	0.5	0.30	0.30	0.30	0.50	0.00	0.00	0.049	0.012	-0.008
1	20	1	0.5	0.5	0.5	0.30	0.30	0.30	0.50	0.00	0.25	0.013	0.051	0.010
1	20	1	0.5	0.5	0.5	0.30	0.30	0.30	0.50	0.00	0.50	0.011	0.000	-0.010
1	20	1	0.5	0.5	0.5	0.30	0.30	0.30	0.50	0.25	0.00	0.042	-0.008	-0.011
1	20	1	0.5	0.5	0.5	0.30	0.30	0.30	0.50	0.25	0.25	0.035	0.018	0.010
1	20	1	0.5	0.5	0.5	0.30	0.30	0.30	0.50	0.25	0.50	0.020	0.013	0.008
1	20	1	0.5	0.5	0.5	0.30	0.30	0.30	0.50	0.50	0.00	0.026	-0.019	0.013
1	20	1	0.5	0.5	0.5	0.30	0.30	0.30	0.50	0.50	0.25	0.044	0.006	0.016
1	20	1	0.5	0.5	0.5	0.30	0.30	0.30	0.50	0.50	0.50	0.046	0.015	0.000
1	20	1	0.5	0.8	0.5	0.00	0.00	0.00	0.00	0.00	0.00	0.004	0.005	0.009
1	20	1	0.5	0.8	0.5	0.15	0.15	0.15	0.00	0.00	0.00	0.021	-0.017	0.008
1	20	1	0.5	0.8	0.5	0.15	0.15	0.15	0.00	0.00	0.25	0.003	-0.005	-0.004
1	20	1	0.5	0.8	0.5	0.15	0.15	0.15	0.00	0.00	0.50	0.002	-0.004	0.009
1	20	1	0.5	0.8	0.5	0.15	0.15	0.15	0.00	0.25	0.00	0.031	-0.004	0.007

(continued)

												Mean Bias		
N	m_1	$\frac{m_2}{m_1}$	$E(C)$	$E(R)$	$E(U)$	σ_C	σ_R	σ_U	ρ_{CR}	ρ_{CU}	ρ_{RU}	c	r	u
1	20	1	0.5	0.8	0.5	0.15	0.15	0.15	0.00	0.25	0.25	0.009	-0.009	0.000
1	20	1	0.5	0.8	0.5	0.15	0.15	0.15	0.00	0.25	0.50	0.013	-0.003	0.009
1	20	1	0.5	0.8	0.5	0.15	0.15	0.15	0.00	0.50	0.00	0.021	-0.014	0.014
1	20	1	0.5	0.8	0.5	0.15	0.15	0.15	0.00	0.50	0.25	0.018	-0.010	0.007
1	20	1	0.5	0.8	0.5	0.15	0.15	0.15	0.00	0.50	0.50	0.018	-0.016	0.010
1	20	1	0.5	0.8	0.5	0.15	0.15	0.15	0.25	0.00	0.00	0.018	-0.001	0.016
1	20	1	0.5	0.8	0.5	0.15	0.15	0.15	0.25	0.00	0.25	0.018	-0.007	0.005
1	20	1	0.5	0.8	0.5	0.15	0.15	0.15	0.25	0.00	0.50	0.019	-0.004	0.009
1	20	1	0.5	0.8	0.5	0.15	0.15	0.15	0.25	0.25	0.00	0.021	-0.010	0.006
1	20	1	0.5	0.8	0.5	0.15	0.15	0.15	0.25	0.25	0.25	0.009	-0.004	0.001
1	20	1	0.5	0.8	0.5	0.15	0.15	0.15	0.25	0.25	0.50	0.006	0.005	0.007
1	20	1	0.5	0.8	0.5	0.15	0.15	0.15	0.25	0.50	0.00	0.019	-0.020	0.002
1	20	1	0.5	0.8	0.5	0.15	0.15	0.15	0.25	0.50	0.25	0.017	-0.011	0.004
1	20	1	0.5	0.8	0.5	0.15	0.15	0.15	0.25	0.50	0.50	0.010	-0.004	0.002
1	20	1	0.5	0.8	0.5	0.15	0.15	0.15	0.50	0.00	0.00	0.018	-0.006	0.006
1	20	1	0.5	0.8	0.5	0.15	0.15	0.15	0.50	0.00	0.25	0.019	-0.014	0.010
1	20	1	0.5	0.8	0.5	0.15	0.15	0.15	0.50	0.00	0.50	0.010	0.009	0.015
1	20	1	0.5	0.8	0.5	0.15	0.15	0.15	0.50	0.25	0.00	0.031	-0.005	0.012
1	20	1	0.5	0.8	0.5	0.15	0.15	0.15	0.50	0.25	0.25	0.016	-0.006	0.008
1	20	1	0.5	0.8	0.5	0.15	0.15	0.15	0.50	0.25	0.50	0.021	-0.006	0.013
1	20	1	0.5	0.8	0.5	0.15	0.15	0.15	0.50	0.50	0.00	0.027	-0.011	0.010
1	20	1	0.5	0.8	0.5	0.15	0.15	0.15	0.50	0.50	0.25	0.015	0.003	0.002
1	20	1	0.5	0.8	0.5	0.15	0.15	0.15	0.50	0.50	0.50	0.020	0.021	0.014
1	20	1	0.5	0.8	0.5	0.30	0.30	0.30	0.00	0.00	0.00	0.042	-0.097	-0.002
1	20	1	0.5	0.8	0.5	0.30	0.30	0.30	0.00	0.00	0.25	0.039	-0.072	0.004
1	20	1	0.5	0.8	0.5	0.30	0.30	0.30	0.00	0.00	0.50	0.019	-0.089	-0.011
1	20	1	0.5	0.8	0.5	0.30	0.30	0.30	0.00	0.25	0.00	0.058	-0.105	-0.010
1	20	1	0.5	0.8	0.5	0.30	0.30	0.30	0.00	0.25	0.25	0.063	-0.090	0.020
1	20	1	0.5	0.8	0.5	0.30	0.30	0.30	0.00	0.25	0.50	0.047	-0.076	0.016
1	20	1	0.5	0.8	0.5	0.30	0.30	0.30	0.00	0.50	0.00	0.047	-0.107	0.003
1	20	1	0.5	0.8	0.5	0.30	0.30	0.30	0.00	0.50	0.25	0.050	-0.110	0.017
1	20	1	0.5	0.8	0.5	0.30	0.30	0.30	0.00	0.50	0.50	0.046	-0.115	-0.003
1	20	1	0.5	0.8	0.5	0.30	0.30	0.30	0.25	0.00	0.00	0.034	-0.082	0.003
1	20	1	0.5	0.8	0.5	0.30	0.30	0.30	0.25	0.00	0.25	0.042	-0.069	0.014
1	20	1	0.5	0.8	0.5	0.30	0.30	0.30	0.25	0.00	0.50	0.054	-0.041	0.018
1	20	1	0.5	0.8	0.5	0.30	0.30	0.30	0.25	0.25	0.00	0.039	-0.100	0.009
1	20	1	0.5	0.8	0.5	0.30	0.30	0.30	0.25	0.25	0.25	0.040	-0.081	0.010
1	20	1	0.5	0.8	0.5	0.30	0.30	0.30	0.25	0.25	0.50	0.053	-0.051	0.012
1	20	1	0.5	0.8	0.5	0.30	0.30	0.30	0.25	0.50	0.00	0.041	-0.094	0.010

(continued)

												Mean Bias		
N	m_1	$\frac{m_2}{m_1}$	$E(C)$	$E(R)$	$E(U)$	σ_C	σ_R	σ_U	ρ_{CR}	ρ_{CU}	ρ_{RU}	c	r	u
1	20	1	0.5	0.8	0.5	0.30	0.30	0.30	0.25	0.50	0.25	0.051	-0.076	0.026
1	20	1	0.5	0.8	0.5	0.30	0.30	0.30	0.25	0.50	0.50	0.052	-0.080	0.015
1	20	1	0.5	0.8	0.5	0.30	0.30	0.30	0.50	0.00	0.00	0.047	-0.073	0.015
1	20	1	0.5	0.8	0.5	0.30	0.30	0.30	0.50	0.00	0.25	0.024	-0.086	-0.001
1	20	1	0.5	0.8	0.5	0.30	0.30	0.30	0.50	0.00	0.50	0.035	-0.051	0.001
1	20	1	0.5	0.8	0.5	0.30	0.30	0.30	0.50	0.25	0.00	0.041	-0.098	-0.006
1	20	1	0.5	0.8	0.5	0.30	0.30	0.30	0.50	0.25	0.25	0.044	-0.059	0.011
1	20	1	0.5	0.8	0.5	0.30	0.30	0.30	0.50	0.25	0.50	0.053	-0.051	0.016
1	20	1	0.5	0.8	0.5	0.30	0.30	0.30	0.50	0.50	0.00	0.059	-0.089	0.016
1	20	1	0.5	0.8	0.5	0.30	0.30	0.30	0.50	0.50	0.25	0.041	-0.061	0.013
1	20	1	0.5	0.8	0.5	0.30	0.30	0.30	0.50	0.50	0.50	0.038	-0.079	-0.001
1	20	1	0.8	0.2	0.5	0.00	0.00	0.00	0.00	0.00	0.00	-0.011	0.002	-0.003
1	20	1	0.8	0.2	0.5	0.15	0.15	0.15	0.00	0.00	0.00	-0.015	0.013	0.001
1	20	1	0.8	0.2	0.5	0.15	0.15	0.15	0.00	0.00	0.25	-0.010	0.024	0.001
1	20	1	0.8	0.2	0.5	0.15	0.15	0.15	0.00	0.00	0.50	-0.007	0.005	-0.001
1	20	1	0.8	0.2	0.5	0.15	0.15	0.15	0.00	0.25	0.00	-0.013	0.012	-0.009
1	20	1	0.8	0.2	0.5	0.15	0.15	0.15	0.00	0.25	0.25	-0.005	0.009	-0.008
1	20	1	0.8	0.2	0.5	0.15	0.15	0.15	0.00	0.25	0.50	-0.007	0.020	0.002
1	20	1	0.8	0.2	0.5	0.15	0.15	0.15	0.00	0.50	0.00	-0.012	0.010	0.009
1	20	1	0.8	0.2	0.5	0.15	0.15	0.15	0.00	0.50	0.25	-0.015	0.019	0.003
1	20	1	0.8	0.2	0.5	0.15	0.15	0.15	0.00	0.50	0.50	-0.006	0.001	0.001
1	20	1	0.8	0.2	0.5	0.15	0.15	0.15	0.25	0.00	0.00	-0.013	0.008	0.007
1	20	1	0.8	0.2	0.5	0.15	0.15	0.15	0.25	0.00	0.25	-0.015	0.020	0.008
1	20	1	0.8	0.2	0.5	0.15	0.15	0.15	0.25	0.00	0.50	-0.007	-0.005	-0.001
1	20	1	0.8	0.2	0.5	0.15	0.15	0.15	0.25	0.25	0.00	-0.012	0.007	-0.003
1	20	1	0.8	0.2	0.5	0.15	0.15	0.15	0.25	0.25	0.25	-0.009	0.017	0.017
1	20	1	0.8	0.2	0.5	0.15	0.15	0.15	0.25	0.25	0.50	-0.012	0.018	0.009
1	20	1	0.8	0.2	0.5	0.15	0.15	0.15	0.25	0.50	0.00	-0.013	0.007	-0.013
1	20	1	0.8	0.2	0.5	0.15	0.15	0.15	0.25	0.50	0.25	-0.008	0.013	-0.001
1	20	1	0.8	0.2	0.5	0.15	0.15	0.15	0.25	0.50	0.50	-0.018	0.013	0.000
1	20	1	0.8	0.2	0.5	0.15	0.15	0.15	0.50	0.00	0.00	-0.006	0.013	-0.010
1	20	1	0.8	0.2	0.5	0.15	0.15	0.15	0.50	0.00	0.25	-0.005	0.002	-0.011
1	20	1	0.8	0.2	0.5	0.15	0.15	0.15	0.50	0.00	0.50	-0.013	0.008	-0.007
1	20	1	0.8	0.2	0.5	0.15	0.15	0.15	0.50	0.25	0.00	-0.008	0.015	-0.003
1	20	1	0.8	0.2	0.5	0.15	0.15	0.15	0.50	0.25	0.25	-0.014	0.005	0.006
1	20	1	0.8	0.2	0.5	0.15	0.15	0.15	0.50	0.25	0.50	-0.017	0.008	-0.005
1	20	1	0.8	0.2	0.5	0.15	0.15	0.15	0.50	0.50	0.00	-0.010	0.019	-0.004
1	20	1	0.8	0.2	0.5	0.15	0.15	0.15	0.50	0.50	0.25	-0.010	0.009	0.005
1	20	1	0.8	0.2	0.5	0.15	0.15	0.15	0.50	0.50	0.50	-0.013	0.009	-0.001

(continued)

												Mean Bias		
N	m_1	$\frac{m_2}{m_1}$	$E(C)$	$E(R)$	$E(U)$	σ_C	σ_R	σ_U	ρ_{CR}	ρ_{CU}	ρ_{RU}	c	r	u
1	20	1	0.8	0.2	0.5	0.30	0.30	0.30	0.00	0.00	0.00	-0.017	0.008	-0.013
1	20	1	0.8	0.2	0.5	0.30	0.30	0.30	0.00	0.00	0.25	-0.032	-0.005	0.022
1	20	1	0.8	0.2	0.5	0.30	0.30	0.30	0.00	0.00	0.50	-0.019	0.005	-0.003
1	20	1	0.8	0.2	0.5	0.30	0.30	0.30	0.00	0.25	0.00	-0.005	0.008	-0.002
1	20	1	0.8	0.2	0.5	0.30	0.30	0.30	0.00	0.25	0.25	-0.003	-0.009	-0.011
1	20	1	0.8	0.2	0.5	0.30	0.30	0.30	0.00	0.25	0.50	-0.006	-0.007	-0.006
1	20	1	0.8	0.2	0.5	0.30	0.30	0.30	0.00	0.50	0.00	0.023	-0.011	0.003
1	20	1	0.8	0.2	0.5	0.30	0.30	0.30	0.00	0.50	0.25	-0.002	-0.001	0.004
1	20	1	0.8	0.2	0.5	0.30	0.30	0.30	0.00	0.50	0.50	0.004	-0.007	0.001
1	20	1	0.8	0.2	0.5	0.30	0.30	0.30	0.25	0.00	0.00	-0.013	0.005	0.011
1	20	1	0.8	0.2	0.5	0.30	0.30	0.30	0.25	0.00	0.25	-0.025	0.002	0.002
1	20	1	0.8	0.2	0.5	0.30	0.30	0.30	0.25	0.00	0.50	-0.013	-0.007	0.010
1	20	1	0.8	0.2	0.5	0.30	0.30	0.30	0.25	0.25	0.00	-0.023	0.007	0.009
1	20	1	0.8	0.2	0.5	0.30	0.30	0.30	0.25	0.25	0.25	-0.004	0.003	0.004
1	20	1	0.8	0.2	0.5	0.30	0.30	0.30	0.25	0.25	0.50	-0.023	0.001	0.003
1	20	1	0.8	0.2	0.5	0.30	0.30	0.30	0.25	0.50	0.00	-0.002	0.002	0.002
1	20	1	0.8	0.2	0.5	0.30	0.30	0.30	0.25	0.50	0.25	0.009	-0.010	0.006
1	20	1	0.8	0.2	0.5	0.30	0.30	0.30	0.25	0.50	0.50	0.007	0.013	0.009
1	20	1	0.8	0.2	0.5	0.30	0.30	0.30	0.50	0.00	0.00	-0.006	0.006	-0.004
1	20	1	0.8	0.2	0.5	0.30	0.30	0.30	0.50	0.00	0.25	-0.025	0.009	0.025
1	20	1	0.8	0.2	0.5	0.30	0.30	0.30	0.50	0.00	0.50	-0.021	0.017	0.017
1	20	1	0.8	0.2	0.5	0.30	0.30	0.30	0.50	0.25	0.00	-0.013	-0.008	0.009
1	20	1	0.8	0.2	0.5	0.30	0.30	0.30	0.50	0.25	0.25	-0.009	-0.009	-0.005
1	20	1	0.8	0.2	0.5	0.30	0.30	0.30	0.50	0.25	0.50	-0.016	0.008	0.011
1	20	1	0.8	0.2	0.5	0.30	0.30	0.30	0.50	0.50	0.00	0.003	-0.004	0.007
1	20	1	0.8	0.2	0.5	0.30	0.30	0.30	0.50	0.50	0.25	-0.012	0.010	-0.004
1	20	1	0.8	0.2	0.5	0.30	0.30	0.30	0.50	0.50	0.50	-0.010	0.015	-0.005
1	20	1	0.8	0.5	0.5	0.00	0.00	0.00	0.00	0.00	0.00	-0.011	0.013	-0.003
1	20	1	0.8	0.5	0.5	0.15	0.15	0.15	0.00	0.00	0.00	0.002	0.001	0.005
1	20	1	0.8	0.5	0.5	0.15	0.15	0.15	0.00	0.00	0.25	-0.009	0.019	0.000
1	20	1	0.8	0.5	0.5	0.15	0.15	0.15	0.00	0.00	0.50	-0.008	0.007	-0.012
1	20	1	0.8	0.5	0.5	0.15	0.15	0.15	0.00	0.25	0.00	-0.011	0.015	0.003
1	20	1	0.8	0.5	0.5	0.15	0.15	0.15	0.00	0.25	0.25	0.001	0.012	0.004
1	20	1	0.8	0.5	0.5	0.15	0.15	0.15	0.00	0.25	0.50	-0.015	0.019	-0.006
1	20	1	0.8	0.5	0.5	0.15	0.15	0.15	0.00	0.50	0.00	-0.002	0.027	-0.006
1	20	1	0.8	0.5	0.5	0.15	0.15	0.15	0.00	0.50	0.25	-0.011	0.026	0.002
1	20	1	0.8	0.5	0.5	0.15	0.15	0.15	0.00	0.50	0.50	-0.005	0.022	0.003
1	20	1	0.8	0.5	0.5	0.15	0.15	0.15	0.25	0.00	0.00	0.002	0.014	0.008
1	20	1	0.8	0.5	0.5	0.15	0.15	0.15	0.25	0.00	0.25	-0.006	0.006	0.003

(continued)

N	m_1	$\frac{m_2}{m_1}$	$E(C)$	$E(R)$	$E(U)$	σ_C	σ_R	σ_U	ρ_{CR}	ρ_{CU}	ρ_{RU}	Mean Bias		
												c	r	u
1	20	1	0.8	0.5	0.5	0.15	0.15	0.15	0.25	0.00	0.50	-0.011	0.013	-0.008
1	20	1	0.8	0.5	0.5	0.15	0.15	0.15	0.25	0.25	0.00	-0.008	0.019	0.002
1	20	1	0.8	0.5	0.5	0.15	0.15	0.15	0.25	0.25	0.25	-0.011	0.019	0.000
1	20	1	0.8	0.5	0.5	0.15	0.15	0.15	0.25	0.25	0.50	-0.003	0.022	0.003
1	20	1	0.8	0.5	0.5	0.15	0.15	0.15	0.25	0.50	0.00	-0.007	0.021	0.009
1	20	1	0.8	0.5	0.5	0.15	0.15	0.15	0.25	0.50	0.25	-0.006	0.035	0.005
1	20	1	0.8	0.5	0.5	0.15	0.15	0.15	0.25	0.50	0.50	-0.017	0.022	0.002
1	20	1	0.8	0.5	0.5	0.15	0.15	0.15	0.50	0.00	0.00	-0.014	0.023	0.005
1	20	1	0.8	0.5	0.5	0.15	0.15	0.15	0.50	0.00	0.25	-0.017	0.013	-0.010
1	20	1	0.8	0.5	0.5	0.15	0.15	0.15	0.50	0.00	0.50	-0.001	0.012	0.001
1	20	1	0.8	0.5	0.5	0.15	0.15	0.15	0.50	0.25	0.00	-0.019	0.003	-0.001
1	20	1	0.8	0.5	0.5	0.15	0.15	0.15	0.50	0.25	0.25	0.000	0.005	0.001
1	20	1	0.8	0.5	0.5	0.15	0.15	0.15	0.50	0.25	0.50	-0.007	0.009	0.000
1	20	1	0.8	0.5	0.5	0.15	0.15	0.15	0.50	0.50	0.00	-0.003	0.025	-0.001
1	20	1	0.8	0.5	0.5	0.15	0.15	0.15	0.50	0.50	0.25	-0.001	0.022	0.002
1	20	1	0.8	0.5	0.5	0.15	0.15	0.15	0.50	0.50	0.50	-0.007	0.018	0.001
1	20	1	0.8	0.5	0.5	0.30	0.30	0.30	0.00	0.00	0.00	0.002	-0.035	-0.001
1	20	1	0.8	0.5	0.5	0.30	0.30	0.30	0.00	0.00	0.25	-0.007	0.001	0.000
1	20	1	0.8	0.5	0.5	0.30	0.30	0.30	0.00	0.00	0.50	-0.002	-0.011	-0.008
1	20	1	0.8	0.5	0.5	0.30	0.30	0.30	0.00	0.25	0.00	-0.004	-0.014	-0.004
1	20	1	0.8	0.5	0.5	0.30	0.30	0.30	0.00	0.25	0.25	0.000	-0.008	-0.021
1	20	1	0.8	0.5	0.5	0.30	0.30	0.30	0.00	0.25	0.50	-0.004	-0.009	0.015
1	20	1	0.8	0.5	0.5	0.30	0.30	0.30	0.00	0.50	0.00	0.005	0.015	-0.016
1	20	1	0.8	0.5	0.5	0.30	0.30	0.30	0.00	0.50	0.25	0.029	-0.008	0.010
1	20	1	0.8	0.5	0.5	0.30	0.30	0.30	0.00	0.50	0.50	-0.006	-0.015	-0.004
1	20	1	0.8	0.5	0.5	0.30	0.30	0.30	0.25	0.00	0.00	-0.008	-0.008	-0.010
1	20	1	0.8	0.5	0.5	0.30	0.30	0.30	0.25	0.00	0.25	-0.023	-0.010	0.003
1	20	1	0.8	0.5	0.5	0.30	0.30	0.30	0.25	0.00	0.50	-0.009	-0.008	0.004
1	20	1	0.8	0.5	0.5	0.30	0.30	0.30	0.25	0.25	0.00	0.015	-0.006	0.008
1	20	1	0.8	0.5	0.5	0.30	0.30	0.30	0.25	0.25	0.25	0.011	0.002	-0.004
1	20	1	0.8	0.5	0.5	0.30	0.30	0.30	0.25	0.25	0.50	-0.007	-0.008	0.002
1	20	1	0.8	0.5	0.5	0.30	0.30	0.30	0.25	0.50	0.00	0.016	-0.008	0.002
1	20	1	0.8	0.5	0.5	0.30	0.30	0.30	0.25	0.50	0.25	0.023	0.001	0.008
1	20	1	0.8	0.5	0.5	0.30	0.30	0.30	0.25	0.50	0.50	-0.001	-0.005	-0.019
1	20	1	0.8	0.5	0.5	0.30	0.30	0.30	0.50	0.00	0.00	0.001	0.027	0.007
1	20	1	0.8	0.5	0.5	0.30	0.30	0.30	0.50	0.00	0.25	0.000	0.018	0.006
1	20	1	0.8	0.5	0.5	0.30	0.30	0.30	0.50	0.00	0.50	-0.012	0.003	0.026
1	20	1	0.8	0.5	0.5	0.30	0.30	0.30	0.50	0.25	0.00	0.027	0.018	-0.004
1	20	1	0.8	0.5	0.5	0.30	0.30	0.30	0.50	0.25	0.25	0.000	-0.006	0.008

(continued)

												Mean Bias		
N	m_1	$\frac{m_2}{m_1}$	$E(C)$	$E(R)$	$E(U)$	σ_C	σ_R	σ_U	ρ_{CR}	ρ_{CU}	ρ_{RU}	c	r	u
1	20	1	0.8	0.5	0.5	0.30	0.30	0.30	0.50	0.25	0.50	-0.003	0.005	-0.002
1	20	1	0.8	0.5	0.5	0.30	0.30	0.30	0.50	0.50	0.00	0.015	0.010	-0.010
1	20	1	0.8	0.5	0.5	0.30	0.30	0.30	0.50	0.50	0.25	0.005	0.001	-0.001
1	20	1	0.8	0.5	0.5	0.30	0.30	0.30	0.50	0.50	0.50	0.011	0.007	0.002
1	20	1	0.8	0.8	0.5	0.00	0.00	0.00	0.00	0.00	0.00	-0.002	0.006	0.002
1	20	1	0.8	0.8	0.5	0.15	0.15	0.15	0.00	0.00	0.00	0.006	-0.004	0.008
1	20	1	0.8	0.8	0.5	0.15	0.15	0.15	0.00	0.00	0.25	0.005	0.009	0.000
1	20	1	0.8	0.8	0.5	0.15	0.15	0.15	0.00	0.00	0.50	-0.002	-0.001	-0.001
1	20	1	0.8	0.8	0.5	0.15	0.15	0.15	0.00	0.25	0.00	0.001	0.005	-0.001
1	20	1	0.8	0.8	0.5	0.15	0.15	0.15	0.00	0.25	0.25	-0.003	0.009	0.003
1	20	1	0.8	0.8	0.5	0.15	0.15	0.15	0.00	0.25	0.50	0.000	-0.005	0.001
1	20	1	0.8	0.8	0.5	0.15	0.15	0.15	0.00	0.50	0.00	0.004	-0.008	0.002
1	20	1	0.8	0.8	0.5	0.15	0.15	0.15	0.00	0.50	0.25	-0.003	-0.013	0.002
1	20	1	0.8	0.8	0.5	0.15	0.15	0.15	0.00	0.50	0.50	0.001	0.007	0.005
1	20	1	0.8	0.8	0.5	0.15	0.15	0.15	0.25	0.00	0.00	0.004	-0.001	-0.008
1	20	1	0.8	0.8	0.5	0.15	0.15	0.15	0.25	0.00	0.25	-0.010	0.001	-0.001
1	20	1	0.8	0.8	0.5	0.15	0.15	0.15	0.25	0.00	0.50	-0.005	0.001	-0.002
1	20	1	0.8	0.8	0.5	0.15	0.15	0.15	0.25	0.25	0.00	0.006	0.000	0.003
1	20	1	0.8	0.8	0.5	0.15	0.15	0.15	0.25	0.25	0.25	0.005	0.004	0.003
1	20	1	0.8	0.8	0.5	0.15	0.15	0.15	0.25	0.25	0.50	-0.013	0.014	-0.006
1	20	1	0.8	0.8	0.5	0.15	0.15	0.15	0.25	0.50	0.00	-0.003	-0.001	0.013
1	20	1	0.8	0.8	0.5	0.15	0.15	0.15	0.25	0.50	0.25	0.006	0.007	0.007
1	20	1	0.8	0.8	0.5	0.15	0.15	0.15	0.25	0.50	0.50	-0.005	-0.001	0.000
1	20	1	0.8	0.8	0.5	0.15	0.15	0.15	0.50	0.00	0.00	0.006	0.005	0.008
1	20	1	0.8	0.8	0.5	0.15	0.15	0.15	0.50	0.00	0.25	0.004	0.005	0.001
1	20	1	0.8	0.8	0.5	0.15	0.15	0.15	0.50	0.00	0.50	-0.004	0.010	0.003
1	20	1	0.8	0.8	0.5	0.15	0.15	0.15	0.50	0.25	0.00	0.006	0.001	0.000
1	20	1	0.8	0.8	0.5	0.15	0.15	0.15	0.50	0.25	0.25	-0.008	0.010	0.015
1	20	1	0.8	0.8	0.5	0.15	0.15	0.15	0.50	0.25	0.50	0.005	0.012	0.002
1	20	1	0.8	0.8	0.5	0.15	0.15	0.15	0.50	0.50	0.00	0.002	0.005	0.010
1	20	1	0.8	0.8	0.5	0.15	0.15	0.15	0.50	0.50	0.25	0.008	0.011	0.004
1	20	1	0.8	0.8	0.5	0.15	0.15	0.15	0.50	0.50	0.50	0.005	0.013	0.001
1	20	1	0.8	0.8	0.5	0.30	0.30	0.30	0.00	0.00	0.00	0.020	-0.043	0.002
1	20	1	0.8	0.8	0.5	0.30	0.30	0.30	0.00	0.00	0.25	0.006	-0.029	0.011
1	20	1	0.8	0.8	0.5	0.30	0.30	0.30	0.00	0.00	0.50	-0.006	-0.034	0.011
1	20	1	0.8	0.8	0.5	0.30	0.30	0.30	0.00	0.25	0.00	0.026	-0.057	0.003
1	20	1	0.8	0.8	0.5	0.30	0.30	0.30	0.00	0.25	0.25	0.018	-0.049	0.021
1	20	1	0.8	0.8	0.5	0.30	0.30	0.30	0.00	0.25	0.50	0.016	-0.046	0.006
1	20	1	0.8	0.8	0.5	0.30	0.30	0.30	0.00	0.50	0.00	0.030	-0.034	0.021

(continued)

												Mean Bias		
N	m_1	$\frac{m_2}{m_1}$	$E(C)$	$E(R)$	$E(U)$	σ_C	σ_R	σ_U	ρ_{CR}	ρ_{CU}	ρ_{RU}	c	r	u
1	20	1	0.8	0.8	0.5	0.30	0.30	0.30	0.00	0.50	0.25	0.038	-0.057	0.009
1	20	1	0.8	0.8	0.5	0.30	0.30	0.30	0.00	0.50	0.50	0.028	-0.072	0.006
1	20	1	0.8	0.8	0.5	0.30	0.30	0.30	0.25	0.00	0.00	0.007	-0.039	0.002
1	20	1	0.8	0.8	0.5	0.30	0.30	0.30	0.25	0.00	0.25	-0.003	-0.036	0.003
1	20	1	0.8	0.8	0.5	0.30	0.30	0.30	0.25	0.00	0.50	0.001	-0.027	0.011
1	20	1	0.8	0.8	0.5	0.30	0.30	0.30	0.25	0.25	0.00	0.022	-0.039	0.007
1	20	1	0.8	0.8	0.5	0.30	0.30	0.30	0.25	0.25	0.25	0.009	-0.049	-0.008
1	20	1	0.8	0.8	0.5	0.30	0.30	0.30	0.25	0.25	0.50	0.013	-0.046	0.000
1	20	1	0.8	0.8	0.5	0.30	0.30	0.30	0.25	0.50	0.00	0.036	-0.042	0.012
1	20	1	0.8	0.8	0.5	0.30	0.30	0.30	0.25	0.50	0.25	0.025	-0.042	-0.010
1	20	1	0.8	0.8	0.5	0.30	0.30	0.30	0.25	0.50	0.50	0.022	-0.030	-0.003
1	20	1	0.8	0.8	0.5	0.30	0.30	0.30	0.50	0.00	0.00	0.006	-0.027	0.006
1	20	1	0.8	0.8	0.5	0.30	0.30	0.30	0.50	0.00	0.25	0.017	-0.021	-0.004
1	20	1	0.8	0.8	0.5	0.30	0.30	0.30	0.50	0.00	0.50	0.005	-0.021	0.005
1	20	1	0.8	0.8	0.5	0.30	0.30	0.30	0.50	0.25	0.00	0.013	-0.032	-0.012
1	20	1	0.8	0.8	0.5	0.30	0.30	0.30	0.50	0.25	0.25	0.014	-0.023	-0.009
1	20	1	0.8	0.8	0.5	0.30	0.30	0.30	0.50	0.25	0.50	0.012	-0.029	0.009
1	20	1	0.8	0.8	0.5	0.30	0.30	0.30	0.50	0.50	0.00	0.029	-0.034	0.016
1	20	1	0.8	0.8	0.5	0.30	0.30	0.30	0.50	0.50	0.25	0.038	-0.013	0.015
1	20	1	0.8	0.8	0.5	0.30	0.30	0.30	0.50	0.50	0.50	0.022	-0.021	0.011
10	4	0	0.2	0.2	0.5	0.00	0.00	0.00	0.00	0.00	0.00	-0.013	0.133	0.002
10	4	0	0.2	0.2	0.5	0.15	0.15	0.15	0.00	0.00	0.00	0.038	0.042	0.042
10	4	0	0.2	0.2	0.5	0.15	0.15	0.15	0.00	0.00	0.25	0.044	0.038	0.044
10	4	0	0.2	0.2	0.5	0.15	0.15	0.15	0.00	0.00	0.50	0.039	0.037	0.040
10	4	0	0.2	0.2	0.5	0.15	0.15	0.15	0.00	0.25	0.00	0.033	0.061	0.025
10	4	0	0.2	0.2	0.5	0.15	0.15	0.15	0.00	0.25	0.25	0.039	0.045	0.028
10	4	0	0.2	0.2	0.5	0.15	0.15	0.15	0.00	0.25	0.50	0.035	0.051	0.029
10	4	0	0.2	0.2	0.5	0.15	0.15	0.15	0.00	0.50	0.00	0.039	0.048	0.023
10	4	0	0.2	0.2	0.5	0.15	0.15	0.15	0.00	0.50	0.25	0.039	0.048	0.027
10	4	0	0.2	0.2	0.5	0.15	0.15	0.15	0.00	0.50	0.50	0.040	0.066	0.026
10	4	0	0.2	0.2	0.5	0.15	0.15	0.15	0.25	0.00	0.00	0.044	0.066	0.044
10	4	0	0.2	0.2	0.5	0.15	0.15	0.15	0.25	0.00	0.25	0.041	0.062	0.040
10	4	0	0.2	0.2	0.5	0.15	0.15	0.15	0.25	0.00	0.50	0.040	0.074	0.039
10	4	0	0.2	0.2	0.5	0.15	0.15	0.15	0.25	0.25	0.00	0.036	0.071	0.031
10	4	0	0.2	0.2	0.5	0.15	0.15	0.15	0.25	0.25	0.25	0.041	0.062	0.033
10	4	0	0.2	0.2	0.5	0.15	0.15	0.15	0.25	0.25	0.50	0.045	0.058	0.030
10	4	0	0.2	0.2	0.5	0.15	0.15	0.15	0.25	0.50	0.00	0.042	0.073	0.026
10	4	0	0.2	0.2	0.5	0.15	0.15	0.15	0.25	0.50	0.25	0.044	0.067	0.028
10	4	0	0.2	0.2	0.5	0.15	0.15	0.15	0.25	0.50	0.50	0.049	0.067	0.030

(continued)

												Mean Bias		
N	m_1	$\frac{m_2}{m_1}$	$E(C)$	$E(R)$	$E(U)$	σ_C	σ_R	σ_U	ρ_{CR}	ρ_{CU}	ρ_{RU}	c	r	u
10	4	0	0.2	0.2	0.5	0.15	0.15	0.15	0.50	0.00	0.00	0.049	0.080	0.049
10	4	0	0.2	0.2	0.5	0.15	0.15	0.15	0.50	0.00	0.25	0.029	0.106	0.027
10	4	0	0.2	0.2	0.5	0.15	0.15	0.15	0.50	0.00	0.50	0.032	0.097	0.036
10	4	0	0.2	0.2	0.5	0.15	0.15	0.15	0.50	0.25	0.00	0.052	0.075	0.035
10	4	0	0.2	0.2	0.5	0.15	0.15	0.15	0.50	0.25	0.25	0.044	0.083	0.033
10	4	0	0.2	0.2	0.5	0.15	0.15	0.15	0.50	0.25	0.50	0.041	0.097	0.034
10	4	0	0.2	0.2	0.5	0.15	0.15	0.15	0.50	0.50	0.00	0.045	0.094	0.027
10	4	0	0.2	0.2	0.5	0.15	0.15	0.15	0.50	0.50	0.25	0.035	0.115	0.021
10	4	0	0.2	0.2	0.5	0.15	0.15	0.15	0.50	0.50	0.50	0.049	0.089	0.034
10	4	0	0.2	0.2	0.5	0.30	0.30	0.30	0.00	0.00	0.00	0.183	-0.088	0.158
10	4	0	0.2	0.2	0.5	0.30	0.30	0.30	0.00	0.00	0.25	0.184	-0.093	0.160
10	4	0	0.2	0.2	0.5	0.30	0.30	0.30	0.00	0.00	0.50	0.183	-0.085	0.157
10	4	0	0.2	0.2	0.5	0.30	0.30	0.30	0.00	0.25	0.00	0.196	-0.084	0.150
10	4	0	0.2	0.2	0.5	0.30	0.30	0.30	0.00	0.25	0.25	0.195	-0.087	0.145
10	4	0	0.2	0.2	0.5	0.30	0.30	0.30	0.00	0.25	0.50	0.186	-0.091	0.145
10	4	0	0.2	0.2	0.5	0.30	0.30	0.30	0.00	0.50	0.00	0.199	-0.097	0.123
10	4	0	0.2	0.2	0.5	0.30	0.30	0.30	0.00	0.50	0.25	0.207	-0.082	0.122
10	4	0	0.2	0.2	0.5	0.30	0.30	0.30	0.00	0.50	0.50	0.211	-0.087	0.121
10	4	0	0.2	0.2	0.5	0.30	0.30	0.30	0.25	0.00	0.00	0.187	-0.046	0.167
10	4	0	0.2	0.2	0.5	0.30	0.30	0.30	0.25	0.00	0.25	0.186	-0.042	0.159
10	4	0	0.2	0.2	0.5	0.30	0.30	0.30	0.25	0.00	0.50	0.185	-0.045	0.157
10	4	0	0.2	0.2	0.5	0.30	0.30	0.30	0.25	0.25	0.00	0.201	-0.038	0.141
10	4	0	0.2	0.2	0.5	0.30	0.30	0.30	0.25	0.25	0.25	0.195	-0.041	0.139
10	4	0	0.2	0.2	0.5	0.30	0.30	0.30	0.25	0.25	0.50	0.196	-0.053	0.150
10	4	0	0.2	0.2	0.5	0.30	0.30	0.30	0.25	0.50	0.00	0.207	-0.034	0.121
10	4	0	0.2	0.2	0.5	0.30	0.30	0.30	0.25	0.50	0.25	0.205	-0.052	0.123
10	4	0	0.2	0.2	0.5	0.30	0.30	0.30	0.25	0.50	0.50	0.195	-0.054	0.123
10	4	0	0.2	0.2	0.5	0.30	0.30	0.30	0.50	0.00	0.00	0.190	0.016	0.168
10	4	0	0.2	0.2	0.5	0.30	0.30	0.30	0.50	0.00	0.25	0.186	0.017	0.160
10	4	0	0.2	0.2	0.5	0.30	0.30	0.30	0.50	0.00	0.50	0.184	0.004	0.160
10	4	0	0.2	0.2	0.5	0.30	0.30	0.30	0.50	0.25	0.00	0.196	0.011	0.142
10	4	0	0.2	0.2	0.5	0.30	0.30	0.30	0.50	0.25	0.25	0.195	0.007	0.145
10	4	0	0.2	0.2	0.5	0.30	0.30	0.30	0.50	0.25	0.50	0.200	0.006	0.147
10	4	0	0.2	0.2	0.5	0.30	0.30	0.30	0.50	0.50	0.00	0.209	0.000	0.125
10	4	0	0.2	0.2	0.5	0.30	0.30	0.30	0.50	0.50	0.25	0.203	0.000	0.124
10	4	0	0.2	0.2	0.5	0.30	0.30	0.30	0.50	0.50	0.50	0.201	0.006	0.125
10	4	0	0.2	0.5	0.5	0.00	0.00	0.00	0.00	0.00	0.00	0.005	0.103	0.011
10	4	0	0.2	0.5	0.5	0.15	0.15	0.15	0.00	0.00	0.00	0.047	-0.016	0.038
10	4	0	0.2	0.5	0.5	0.15	0.15	0.15	0.00	0.00	0.25	0.046	-0.010	0.044

(continued)

N	m_1	$\frac{m_2}{m_1}$	$E(C)$	$E(R)$	$E(U)$	σ_C	σ_R	σ_U	ρ_{CR}	ρ_{CU}	ρ_{RU}	Mean Bias c	Mean Bias r	Mean Bias u
10	4	0	0.2	0.5	0.5	0.15	0.15	0.15	0.00	0.00	0.50	0.045	0.003	0.042
10	4	0	0.2	0.5	0.5	0.15	0.15	0.15	0.00	0.25	0.00	0.055	-0.029	0.037
10	4	0	0.2	0.5	0.5	0.15	0.15	0.15	0.00	0.25	0.25	0.049	-0.004	0.040
10	4	0	0.2	0.5	0.5	0.15	0.15	0.15	0.00	0.25	0.50	0.048	-0.014	0.035
10	4	0	0.2	0.5	0.5	0.15	0.15	0.15	0.00	0.50	0.00	0.046	-0.002	0.030
10	4	0	0.2	0.5	0.5	0.15	0.15	0.15	0.00	0.50	0.25	0.050	-0.019	0.031
10	4	0	0.2	0.5	0.5	0.15	0.15	0.15	0.00	0.50	0.50	0.054	-0.007	0.026
10	4	0	0.2	0.5	0.5	0.15	0.15	0.15	0.25	0.00	0.00	0.042	0.019	0.036
10	4	0	0.2	0.5	0.5	0.15	0.15	0.15	0.25	0.00	0.25	0.045	0.010	0.038
10	4	0	0.2	0.5	0.5	0.15	0.15	0.15	0.25	0.00	0.50	0.050	0.002	0.046
10	4	0	0.2	0.5	0.5	0.15	0.15	0.15	0.25	0.25	0.00	0.052	0.000	0.036
10	4	0	0.2	0.5	0.5	0.15	0.15	0.15	0.25	0.25	0.25	0.052	0.008	0.036
10	4	0	0.2	0.5	0.5	0.15	0.15	0.15	0.25	0.25	0.50	0.048	0.001	0.036
10	4	0	0.2	0.5	0.5	0.15	0.15	0.15	0.25	0.50	0.00	0.046	0.027	0.029
10	4	0	0.2	0.5	0.5	0.15	0.15	0.15	0.25	0.50	0.25	0.049	0.000	0.031
10	4	0	0.2	0.5	0.5	0.15	0.15	0.15	0.25	0.50	0.50	0.056	0.011	0.036
10	4	0	0.2	0.5	0.5	0.15	0.15	0.15	0.50	0.00	0.00	0.051	0.038	0.040
10	4	0	0.2	0.5	0.5	0.15	0.15	0.15	0.50	0.00	0.25	0.054	0.013	0.049
10	4	0	0.2	0.5	0.5	0.15	0.15	0.15	0.50	0.00	0.50	0.051	0.028	0.042
10	4	0	0.2	0.5	0.5	0.15	0.15	0.15	0.50	0.25	0.00	0.049	0.034	0.036
10	4	0	0.2	0.5	0.5	0.15	0.15	0.15	0.50	0.25	0.25	0.052	0.012	0.043
10	4	0	0.2	0.5	0.5	0.15	0.15	0.15	0.50	0.25	0.50	0.053	0.027	0.035
10	4	0	0.2	0.5	0.5	0.15	0.15	0.15	0.50	0.50	0.00	0.050	0.037	0.036
10	4	0	0.2	0.5	0.5	0.15	0.15	0.15	0.50	0.50	0.25	0.055	0.029	0.028
10	4	0	0.2	0.5	0.5	0.15	0.15	0.15	0.50	0.50	0.50	0.054	0.030	0.034
10	4	0	0.2	0.5	0.5	0.30	0.30	0.30	0.00	0.00	0.00	0.181	-0.220	0.159
10	4	0	0.2	0.5	0.5	0.30	0.30	0.30	0.00	0.00	0.25	0.189	-0.223	0.165
10	4	0	0.2	0.5	0.5	0.30	0.30	0.30	0.00	0.00	0.50	0.187	-0.215	0.160
10	4	0	0.2	0.5	0.5	0.30	0.30	0.30	0.00	0.25	0.00	0.203	-0.232	0.140
10	4	0	0.2	0.5	0.5	0.30	0.30	0.30	0.00	0.25	0.25	0.201	-0.218	0.142
10	4	0	0.2	0.5	0.5	0.30	0.30	0.30	0.00	0.25	0.50	0.206	-0.214	0.142
10	4	0	0.2	0.5	0.5	0.30	0.30	0.30	0.00	0.50	0.00	0.206	-0.239	0.115
10	4	0	0.2	0.5	0.5	0.30	0.30	0.30	0.00	0.50	0.25	0.202	-0.237	0.122
10	4	0	0.2	0.5	0.5	0.30	0.30	0.30	0.00	0.50	0.50	0.208	-0.225	0.120
10	4	0	0.2	0.5	0.5	0.30	0.30	0.30	0.25	0.00	0.00	0.190	-0.185	0.162
10	4	0	0.2	0.5	0.5	0.30	0.30	0.30	0.25	0.00	0.25	0.187	-0.176	0.161
10	4	0	0.2	0.5	0.5	0.30	0.30	0.30	0.25	0.00	0.50	0.194	-0.185	0.168
10	4	0	0.2	0.5	0.5	0.30	0.30	0.30	0.25	0.25	0.00	0.195	-0.186	0.141
10	4	0	0.2	0.5	0.5	0.30	0.30	0.30	0.25	0.25	0.25	0.196	-0.184	0.149

(continued)

												Mean Bias		
N	m_1	$\frac{m_2}{m_1}$	$E(C)$	$E(R)$	$E(U)$	σ_C	σ_R	σ_U	ρ_{CR}	ρ_{CU}	ρ_{RU}	c	r	u
10	4	0	0.2	0.5	0.5	0.30	0.30	0.30	0.25	0.25	0.50	0.204	-0.183	0.148
10	4	0	0.2	0.5	0.5	0.30	0.30	0.30	0.25	0.50	0.00	0.203	-0.186	0.125
10	4	0	0.2	0.5	0.5	0.30	0.30	0.30	0.25	0.50	0.25	0.211	-0.194	0.124
10	4	0	0.2	0.5	0.5	0.30	0.30	0.30	0.25	0.50	0.50	0.211	-0.206	0.127
10	4	0	0.2	0.5	0.5	0.30	0.30	0.30	0.50	0.00	0.00	0.186	-0.135	0.153
10	4	0	0.2	0.5	0.5	0.30	0.30	0.30	0.50	0.00	0.25	0.190	-0.122	0.161
10	4	0	0.2	0.5	0.5	0.30	0.30	0.30	0.50	0.00	0.50	0.190	-0.125	0.161
10	4	0	0.2	0.5	0.5	0.30	0.30	0.30	0.50	0.25	0.00	0.205	-0.139	0.142
10	4	0	0.2	0.5	0.5	0.30	0.30	0.30	0.50	0.25	0.25	0.197	-0.146	0.146
10	4	0	0.2	0.5	0.5	0.30	0.30	0.30	0.50	0.25	0.50	0.194	-0.147	0.145
10	4	0	0.2	0.5	0.5	0.30	0.30	0.30	0.50	0.50	0.00	0.209	-0.142	0.119
10	4	0	0.2	0.5	0.5	0.30	0.30	0.30	0.50	0.50	0.25	0.216	-0.146	0.126
10	4	0	0.2	0.5	0.5	0.30	0.30	0.30	0.50	0.50	0.50	0.219	-0.146	0.127
10	4	0	0.2	0.8	0.5	0.00	0.00	0.00	0.00	0.00	0.00	0.024	-0.020	0.023
10	4	0	0.2	0.8	0.5	0.15	0.15	0.15	0.00	0.00	0.00	0.057	-0.126	0.051
10	4	0	0.2	0.8	0.5	0.15	0.15	0.15	0.00	0.00	0.25	0.059	-0.116	0.048
10	4	0	0.2	0.8	0.5	0.15	0.15	0.15	0.00	0.00	0.50	0.057	-0.116	0.049
10	4	0	0.2	0.8	0.5	0.15	0.15	0.15	0.00	0.25	0.00	0.063	-0.136	0.043
10	4	0	0.2	0.8	0.5	0.15	0.15	0.15	0.00	0.25	0.25	0.063	-0.117	0.041
10	4	0	0.2	0.8	0.5	0.15	0.15	0.15	0.00	0.25	0.50	0.061	-0.118	0.037
10	4	0	0.2	0.8	0.5	0.15	0.15	0.15	0.00	0.50	0.00	0.066	-0.126	0.038
10	4	0	0.2	0.8	0.5	0.15	0.15	0.15	0.00	0.50	0.25	0.065	-0.130	0.041
10	4	0	0.2	0.8	0.5	0.15	0.15	0.15	0.00	0.50	0.50	0.062	-0.129	0.038
10	4	0	0.2	0.8	0.5	0.15	0.15	0.15	0.25	0.00	0.00	0.066	-0.110	0.048
10	4	0	0.2	0.8	0.5	0.15	0.15	0.15	0.25	0.00	0.25	0.065	-0.113	0.048
10	4	0	0.2	0.8	0.5	0.15	0.15	0.15	0.25	0.00	0.50	0.059	-0.104	0.043
10	4	0	0.2	0.8	0.5	0.15	0.15	0.15	0.25	0.25	0.00	0.067	-0.115	0.046
10	4	0	0.2	0.8	0.5	0.15	0.15	0.15	0.25	0.25	0.25	0.071	-0.118	0.048
10	4	0	0.2	0.8	0.5	0.15	0.15	0.15	0.25	0.25	0.50	0.061	-0.106	0.040
10	4	0	0.2	0.8	0.5	0.15	0.15	0.15	0.25	0.50	0.00	0.068	-0.112	0.041
10	4	0	0.2	0.8	0.5	0.15	0.15	0.15	0.25	0.50	0.25	0.068	-0.116	0.039
10	4	0	0.2	0.8	0.5	0.15	0.15	0.15	0.25	0.50	0.50	0.068	-0.124	0.038
10	4	0	0.2	0.8	0.5	0.15	0.15	0.15	0.50	0.00	0.00	0.061	-0.100	0.053
10	4	0	0.2	0.8	0.5	0.15	0.15	0.15	0.50	0.00	0.25	0.072	-0.107	0.053
10	4	0	0.2	0.8	0.5	0.15	0.15	0.15	0.50	0.00	0.50	0.064	-0.102	0.050
10	4	0	0.2	0.8	0.5	0.15	0.15	0.15	0.50	0.25	0.00	0.062	-0.102	0.045
10	4	0	0.2	0.8	0.5	0.15	0.15	0.15	0.50	0.25	0.25	0.062	-0.095	0.045
10	4	0	0.2	0.8	0.5	0.15	0.15	0.15	0.50	0.25	0.50	0.067	-0.095	0.045
10	4	0	0.2	0.8	0.5	0.15	0.15	0.15	0.50	0.50	0.00	0.071	-0.108	0.036

(continued)

												Mean Bias		
N	m_1	$\frac{m_2}{m_1}$	$E(C)$	$E(R)$	$E(U)$	σ_C	σ_R	σ_U	ρ_{CR}	ρ_{CU}	ρ_{RU}	c	r	u
10	4	0	0.2	0.8	0.5	0.15	0.15	0.15	0.50	0.50	0.25	0.069	-0.117	0.038
10	4	0	0.2	0.8	0.5	0.15	0.15	0.15	0.50	0.50	0.50	0.059	-0.090	0.033
10	4	0	0.2	0.8	0.5	0.30	0.30	0.30	0.00	0.00	0.00	0.189	-0.371	0.160
10	4	0	0.2	0.8	0.5	0.30	0.30	0.30	0.00	0.00	0.25	0.198	-0.372	0.168
10	4	0	0.2	0.8	0.5	0.30	0.30	0.30	0.00	0.00	0.50	0.188	-0.379	0.171
10	4	0	0.2	0.8	0.5	0.30	0.30	0.30	0.00	0.25	0.00	0.203	-0.375	0.147
10	4	0	0.2	0.8	0.5	0.30	0.30	0.30	0.00	0.25	0.25	0.208	-0.406	0.146
10	4	0	0.2	0.8	0.5	0.30	0.30	0.30	0.00	0.25	0.50	0.202	-0.378	0.142
10	4	0	0.2	0.8	0.5	0.30	0.30	0.30	0.00	0.50	0.00	0.210	-0.393	0.123
10	4	0	0.2	0.8	0.5	0.30	0.30	0.30	0.00	0.50	0.25	0.198	-0.374	0.124
10	4	0	0.2	0.8	0.5	0.30	0.30	0.30	0.00	0.50	0.50	0.206	-0.366	0.119
10	4	0	0.2	0.8	0.5	0.30	0.30	0.30	0.25	0.00	0.00	0.195	-0.333	0.162
10	4	0	0.2	0.8	0.5	0.30	0.30	0.30	0.25	0.00	0.25	0.192	-0.320	0.159
10	4	0	0.2	0.8	0.5	0.30	0.30	0.30	0.25	0.00	0.50	0.192	-0.344	0.163
10	4	0	0.2	0.8	0.5	0.30	0.30	0.30	0.25	0.25	0.00	0.192	-0.348	0.142
10	4	0	0.2	0.8	0.5	0.30	0.30	0.30	0.25	0.25	0.25	0.191	-0.342	0.144
10	4	0	0.2	0.8	0.5	0.30	0.30	0.30	0.25	0.25	0.50	0.199	-0.345	0.149
10	4	0	0.2	0.8	0.5	0.30	0.30	0.30	0.25	0.50	0.00	0.213	-0.349	0.131
10	4	0	0.2	0.8	0.5	0.30	0.30	0.30	0.25	0.50	0.25	0.202	-0.340	0.125
10	4	0	0.2	0.8	0.5	0.30	0.30	0.30	0.25	0.50	0.50	0.202	-0.362	0.124
10	4	0	0.2	0.8	0.5	0.30	0.30	0.30	0.50	0.00	0.00	0.187	-0.320	0.167
10	4	0	0.2	0.8	0.5	0.30	0.30	0.30	0.50	0.00	0.25	0.188	-0.307	0.166
10	4	0	0.2	0.8	0.5	0.30	0.30	0.30	0.50	0.00	0.50	0.189	-0.300	0.162
10	4	0	0.2	0.8	0.5	0.30	0.30	0.30	0.50	0.25	0.00	0.201	-0.327	0.144
10	4	0	0.2	0.8	0.5	0.30	0.30	0.30	0.50	0.25	0.25	0.198	-0.315	0.141
10	4	0	0.2	0.8	0.5	0.30	0.30	0.30	0.50	0.25	0.50	0.203	-0.311	0.154
10	4	0	0.2	0.8	0.5	0.30	0.30	0.30	0.50	0.50	0.00	0.204	-0.322	0.120
10	4	0	0.2	0.8	0.5	0.30	0.30	0.30	0.50	0.50	0.25	0.213	-0.329	0.133
10	4	0	0.2	0.8	0.5	0.30	0.30	0.30	0.50	0.50	0.50	0.206	-0.324	0.125
10	4	0	0.5	0.2	0.5	0.00	0.00	0.00	0.00	0.00	0.00	-0.044	0.075	-0.015
10	4	0	0.5	0.2	0.5	0.15	0.15	0.15	0.00	0.00	0.00	0.006	0.026	0.036
10	4	0	0.5	0.2	0.5	0.15	0.15	0.15	0.00	0.00	0.25	0.001	0.034	0.029
10	4	0	0.5	0.2	0.5	0.15	0.15	0.15	0.00	0.00	0.50	-0.007	0.036	0.023
10	4	0	0.5	0.2	0.5	0.15	0.15	0.15	0.00	0.25	0.00	0.001	0.040	0.024
10	4	0	0.5	0.2	0.5	0.15	0.15	0.15	0.00	0.25	0.25	0.004	0.028	0.019
10	4	0	0.5	0.2	0.5	0.15	0.15	0.15	0.00	0.25	0.50	0.006	0.036	0.027
10	4	0	0.5	0.2	0.5	0.15	0.15	0.15	0.00	0.50	0.00	0.000	0.032	0.006
10	4	0	0.5	0.2	0.5	0.15	0.15	0.15	0.00	0.50	0.25	0.004	0.035	0.015
10	4	0	0.5	0.2	0.5	0.15	0.15	0.15	0.00	0.50	0.50	0.003	0.028	0.011

(continued)

N	m_1	$\frac{m_2}{m_1}$	$E(C)$	$E(R)$	$E(U)$	σ_C	σ_R	σ_U	ρ_{CR}	ρ_{CU}	ρ_{RU}	Mean Bias		
												c	r	u
10	4	0	0.5	0.2	0.5	0.15	0.15	0.15	0.25	0.00	0.00	0.006	0.045	0.028
10	4	0	0.5	0.2	0.5	0.15	0.15	0.15	0.25	0.00	0.25	-0.003	0.051	0.027
10	4	0	0.5	0.2	0.5	0.15	0.15	0.15	0.25	0.00	0.50	0.017	0.028	0.037
10	4	0	0.5	0.2	0.5	0.15	0.15	0.15	0.25	0.25	0.00	0.011	0.034	0.029
10	4	0	0.5	0.2	0.5	0.15	0.15	0.15	0.25	0.25	0.25	0.003	0.046	0.019
10	4	0	0.5	0.2	0.5	0.15	0.15	0.15	0.25	0.25	0.50	0.004	0.042	0.027
10	4	0	0.5	0.2	0.5	0.15	0.15	0.15	0.25	0.50	0.00	-0.004	0.056	0.011
10	4	0	0.5	0.2	0.5	0.15	0.15	0.15	0.25	0.50	0.25	0.002	0.052	0.016
10	4	0	0.5	0.2	0.5	0.15	0.15	0.15	0.25	0.50	0.50	-0.010	0.048	0.007
10	4	0	0.5	0.2	0.5	0.15	0.15	0.15	0.50	0.00	0.00	-0.001	0.055	0.023
10	4	0	0.5	0.2	0.5	0.15	0.15	0.15	0.50	0.00	0.25	0.007	0.056	0.035
10	4	0	0.5	0.2	0.5	0.15	0.15	0.15	0.50	0.00	0.50	0.000	0.059	0.032
10	4	0	0.5	0.2	0.5	0.15	0.15	0.15	0.50	0.25	0.00	0.007	0.050	0.021
10	4	0	0.5	0.2	0.5	0.15	0.15	0.15	0.50	0.25	0.25	0.008	0.057	0.029
10	4	0	0.5	0.2	0.5	0.15	0.15	0.15	0.50	0.25	0.50	0.009	0.048	0.032
10	4	0	0.5	0.2	0.5	0.15	0.15	0.15	0.50	0.50	0.00	-0.002	0.062	0.008
10	4	0	0.5	0.2	0.5	0.15	0.15	0.15	0.50	0.50	0.25	0.002	0.057	0.017
10	4	0	0.5	0.2	0.5	0.15	0.15	0.15	0.50	0.50	0.50	-0.007	0.066	0.006
10	4	0	0.5	0.2	0.5	0.30	0.30	0.30	0.00	0.00	0.00	0.100	-0.017	0.146
10	4	0	0.5	0.2	0.5	0.30	0.30	0.30	0.00	0.00	0.25	0.103	-0.023	0.158
10	4	0	0.5	0.2	0.5	0.30	0.30	0.30	0.00	0.00	0.50	0.103	-0.024	0.153
10	4	0	0.5	0.2	0.5	0.30	0.30	0.30	0.00	0.25	0.00	0.110	-0.023	0.120
10	4	0	0.5	0.2	0.5	0.30	0.30	0.30	0.00	0.25	0.25	0.115	-0.024	0.126
10	4	0	0.5	0.2	0.5	0.30	0.30	0.30	0.00	0.25	0.50	0.122	-0.022	0.127
10	4	0	0.5	0.2	0.5	0.30	0.30	0.30	0.00	0.50	0.00	0.123	-0.021	0.092
10	4	0	0.5	0.2	0.5	0.30	0.30	0.30	0.00	0.50	0.25	0.121	-0.019	0.092
10	4	0	0.5	0.2	0.5	0.30	0.30	0.30	0.00	0.50	0.50	0.125	-0.024	0.089
10	4	0	0.5	0.2	0.5	0.30	0.30	0.30	0.25	0.00	0.00	0.097	0.004	0.157
10	4	0	0.5	0.2	0.5	0.30	0.30	0.30	0.25	0.00	0.25	0.095	0.009	0.152
10	4	0	0.5	0.2	0.5	0.30	0.30	0.30	0.25	0.00	0.50	0.105	0.005	0.164
10	4	0	0.5	0.2	0.5	0.30	0.30	0.30	0.25	0.25	0.00	0.105	0.020	0.115
10	4	0	0.5	0.2	0.5	0.30	0.30	0.30	0.25	0.25	0.25	0.109	0.012	0.118
10	4	0	0.5	0.2	0.5	0.30	0.30	0.30	0.25	0.25	0.50	0.112	0.003	0.130
10	4	0	0.5	0.2	0.5	0.30	0.30	0.30	0.25	0.50	0.00	0.126	0.008	0.086
10	4	0	0.5	0.2	0.5	0.30	0.30	0.30	0.25	0.50	0.25	0.130	0.005	0.097
10	4	0	0.5	0.2	0.5	0.30	0.30	0.30	0.25	0.50	0.50	0.118	0.007	0.080
10	4	0	0.5	0.2	0.5	0.30	0.30	0.30	0.50	0.00	0.00	0.099	0.040	0.151
10	4	0	0.5	0.2	0.5	0.30	0.30	0.30	0.50	0.00	0.25	0.104	0.036	0.156
10	4	0	0.5	0.2	0.5	0.30	0.30	0.30	0.50	0.00	0.50	0.105	0.029	0.155

(continued)

												Mean Bias		
N	m_1	$\frac{m_2}{m_1}$	$E(C)$	$E(R)$	$E(U)$	σ_C	σ_R	σ_U	ρ_{CR}	ρ_{CU}	ρ_{RU}	c	r	u
10	4	0	0.5	0.2	0.5	0.30	0.30	0.30	0.50	0.25	0.00	0.118	0.040	0.123
10	4	0	0.5	0.2	0.5	0.30	0.30	0.30	0.50	0.25	0.25	0.118	0.038	0.131
10	4	0	0.5	0.2	0.5	0.30	0.30	0.30	0.50	0.25	0.50	0.117	0.034	0.125
10	4	0	0.5	0.2	0.5	0.30	0.30	0.30	0.50	0.50	0.00	0.114	0.048	0.091
10	4	0	0.5	0.2	0.5	0.30	0.30	0.30	0.50	0.50	0.25	0.125	0.040	0.092
10	4	0	0.5	0.2	0.5	0.30	0.30	0.30	0.50	0.50	0.50	0.125	0.038	0.090
10	4	0	0.5	0.5	0.5	0.00	0.00	0.00	0.00	0.00	0.00	-0.024	0.074	0.000
10	4	0	0.5	0.5	0.5	0.15	0.15	0.15	0.00	0.00	0.00	0.003	0.025	0.032
10	4	0	0.5	0.5	0.5	0.15	0.15	0.15	0.00	0.00	0.25	0.012	0.026	0.029
10	4	0	0.5	0.5	0.5	0.15	0.15	0.15	0.00	0.00	0.50	0.010	0.023	0.036
10	4	0	0.5	0.5	0.5	0.15	0.15	0.15	0.00	0.25	0.00	0.015	0.015	0.029
10	4	0	0.5	0.5	0.5	0.15	0.15	0.15	0.00	0.25	0.25	0.017	0.016	0.031
10	4	0	0.5	0.5	0.5	0.15	0.15	0.15	0.00	0.25	0.50	0.007	0.021	0.027
10	4	0	0.5	0.5	0.5	0.15	0.15	0.15	0.00	0.50	0.00	0.012	0.022	0.015
10	4	0	0.5	0.5	0.5	0.15	0.15	0.15	0.00	0.50	0.25	0.005	0.024	0.012
10	4	0	0.5	0.5	0.5	0.15	0.15	0.15	0.00	0.50	0.50	0.000	0.040	0.002
10	4	0	0.5	0.5	0.5	0.15	0.15	0.15	0.25	0.00	0.00	0.013	0.034	0.033
10	4	0	0.5	0.5	0.5	0.15	0.15	0.15	0.25	0.00	0.25	0.006	0.035	0.030
10	4	0	0.5	0.5	0.5	0.15	0.15	0.15	0.25	0.00	0.50	0.015	0.032	0.030
10	4	0	0.5	0.5	0.5	0.15	0.15	0.15	0.25	0.25	0.00	0.005	0.044	0.023
10	4	0	0.5	0.5	0.5	0.15	0.15	0.15	0.25	0.25	0.25	0.000	0.042	0.018
10	4	0	0.5	0.5	0.5	0.15	0.15	0.15	0.25	0.25	0.50	0.014	0.032	0.026
10	4	0	0.5	0.5	0.5	0.15	0.15	0.15	0.25	0.50	0.00	0.014	0.035	0.013
10	4	0	0.5	0.5	0.5	0.15	0.15	0.15	0.25	0.50	0.25	0.010	0.030	0.017
10	4	0	0.5	0.5	0.5	0.15	0.15	0.15	0.25	0.50	0.50	0.006	0.043	0.011
10	4	0	0.5	0.5	0.5	0.15	0.15	0.15	0.50	0.00	0.00	0.002	0.053	0.029
10	4	0	0.5	0.5	0.5	0.15	0.15	0.15	0.50	0.00	0.25	0.006	0.047	0.030
10	4	0	0.5	0.5	0.5	0.15	0.15	0.15	0.50	0.00	0.50	0.012	0.050	0.037
10	4	0	0.5	0.5	0.5	0.15	0.15	0.15	0.50	0.25	0.00	0.012	0.046	0.031
10	4	0	0.5	0.5	0.5	0.15	0.15	0.15	0.50	0.25	0.25	0.004	0.048	0.021
10	4	0	0.5	0.5	0.5	0.15	0.15	0.15	0.50	0.25	0.50	0.009	0.049	0.027
10	4	0	0.5	0.5	0.5	0.15	0.15	0.15	0.50	0.50	0.00	0.011	0.048	0.015
10	4	0	0.5	0.5	0.5	0.15	0.15	0.15	0.50	0.50	0.25	0.008	0.049	0.008
10	4	0	0.5	0.5	0.5	0.15	0.15	0.15	0.50	0.50	0.50	0.000	0.066	0.009
10	4	0	0.5	0.5	0.5	0.30	0.30	0.30	0.00	0.00	0.00	0.110	-0.070	0.155
10	4	0	0.5	0.5	0.5	0.30	0.30	0.30	0.00	0.00	0.25	0.103	-0.072	0.150
10	4	0	0.5	0.5	0.5	0.30	0.30	0.30	0.00	0.00	0.50	0.102	-0.076	0.158
10	4	0	0.5	0.5	0.5	0.30	0.30	0.30	0.00	0.25	0.00	0.120	-0.072	0.125
10	4	0	0.5	0.5	0.5	0.30	0.30	0.30	0.00	0.25	0.25	0.118	-0.085	0.114

(continued)

												Mean Bias		
N	m_1	$\frac{m_2}{m_1}$	$E(C)$	$E(R)$	$E(U)$	σ_C	σ_R	σ_U	ρ_{CR}	ρ_{CU}	ρ_{RU}	c	r	u
10	4	0	0.5	0.5	0.5	0.30	0.30	0.30	0.00	0.25	0.50	0.122	-0.088	0.127
10	4	0	0.5	0.5	0.5	0.30	0.30	0.30	0.00	0.50	0.00	0.122	-0.077	0.085
10	4	0	0.5	0.5	0.5	0.30	0.30	0.30	0.00	0.50	0.25	0.125	-0.082	0.090
10	4	0	0.5	0.5	0.5	0.30	0.30	0.30	0.00	0.50	0.50	0.126	-0.084	0.096
10	4	0	0.5	0.5	0.5	0.30	0.30	0.30	0.25	0.00	0.00	0.109	-0.042	0.157
10	4	0	0.5	0.5	0.5	0.30	0.30	0.30	0.25	0.00	0.25	0.111	-0.048	0.160
10	4	0	0.5	0.5	0.5	0.30	0.30	0.30	0.25	0.00	0.50	0.109	-0.042	0.152
10	4	0	0.5	0.5	0.5	0.30	0.30	0.30	0.25	0.25	0.00	0.123	-0.055	0.120
10	4	0	0.5	0.5	0.5	0.30	0.30	0.30	0.25	0.25	0.25	0.117	-0.049	0.125
10	4	0	0.5	0.5	0.5	0.30	0.30	0.30	0.25	0.25	0.50	0.111	-0.050	0.121
10	4	0	0.5	0.5	0.5	0.30	0.30	0.30	0.25	0.50	0.00	0.116	-0.050	0.078
10	4	0	0.5	0.5	0.5	0.30	0.30	0.30	0.25	0.50	0.25	0.127	-0.046	0.092
10	4	0	0.5	0.5	0.5	0.30	0.30	0.30	0.25	0.50	0.50	0.131	-0.047	0.087
10	4	0	0.5	0.5	0.5	0.30	0.30	0.30	0.50	0.00	0.00	0.101	-0.013	0.153
10	4	0	0.5	0.5	0.5	0.30	0.30	0.30	0.50	0.00	0.25	0.104	-0.007	0.152
10	4	0	0.5	0.5	0.5	0.30	0.30	0.30	0.50	0.00	0.50	0.108	-0.011	0.152
10	4	0	0.5	0.5	0.5	0.30	0.30	0.30	0.50	0.25	0.00	0.116	-0.016	0.123
10	4	0	0.5	0.5	0.5	0.30	0.30	0.30	0.50	0.25	0.25	0.113	-0.016	0.126
10	4	0	0.5	0.5	0.5	0.30	0.30	0.30	0.50	0.25	0.50	0.125	-0.022	0.131
10	4	0	0.5	0.5	0.5	0.30	0.30	0.30	0.50	0.50	0.00	0.127	-0.017	0.087
10	4	0	0.5	0.5	0.5	0.30	0.30	0.30	0.50	0.50	0.25	0.118	-0.020	0.093
10	4	0	0.5	0.5	0.5	0.30	0.30	0.30	0.50	0.50	0.50	0.128	-0.017	0.091
10	4	0	0.5	0.8	0.5	0.00	0.00	0.00	0.00	0.00	0.00	-0.005	0.025	0.013
10	4	0	0.5	0.8	0.5	0.15	0.15	0.15	0.00	0.00	0.00	0.023	-0.020	0.038
10	4	0	0.5	0.8	0.5	0.15	0.15	0.15	0.00	0.00	0.25	0.024	-0.017	0.043
10	4	0	0.5	0.8	0.5	0.15	0.15	0.15	0.00	0.00	0.50	0.018	-0.016	0.043
10	4	0	0.5	0.8	0.5	0.15	0.15	0.15	0.00	0.25	0.00	0.023	-0.018	0.034
10	4	0	0.5	0.8	0.5	0.15	0.15	0.15	0.00	0.25	0.25	0.024	-0.018	0.031
10	4	0	0.5	0.8	0.5	0.15	0.15	0.15	0.00	0.25	0.50	0.026	-0.007	0.030
10	4	0	0.5	0.8	0.5	0.15	0.15	0.15	0.00	0.50	0.00	0.026	-0.021	0.029
10	4	0	0.5	0.8	0.5	0.15	0.15	0.15	0.00	0.50	0.25	0.019	-0.011	0.018
10	4	0	0.5	0.8	0.5	0.15	0.15	0.15	0.00	0.50	0.50	0.027	-0.014	0.017
10	4	0	0.5	0.8	0.5	0.15	0.15	0.15	0.25	0.00	0.00	0.028	-0.009	0.039
10	4	0	0.5	0.8	0.5	0.15	0.15	0.15	0.25	0.00	0.25	0.025	-0.003	0.044
10	4	0	0.5	0.8	0.5	0.15	0.15	0.15	0.25	0.00	0.50	0.027	-0.012	0.042
10	4	0	0.5	0.8	0.5	0.15	0.15	0.15	0.25	0.25	0.00	0.025	-0.008	0.034
10	4	0	0.5	0.8	0.5	0.15	0.15	0.15	0.25	0.25	0.25	0.018	-0.010	0.033
10	4	0	0.5	0.8	0.5	0.15	0.15	0.15	0.25	0.25	0.50	0.021	-0.007	0.027
10	4	0	0.5	0.8	0.5	0.15	0.15	0.15	0.25	0.50	0.00	0.029	-0.019	0.027

(continued)

												Mean Bias		
N	m_1	$\frac{m_2}{m_1}$	$E(C)$	$E(R)$	$E(U)$	σ_C	σ_R	σ_U	ρ_{CR}	ρ_{CU}	ρ_{RU}	c	r	u
10	4	0	0.5	0.8	0.5	0.15	0.15	0.15	0.25	0.50	0.25	0.036	-0.012	0.024
10	4	0	0.5	0.8	0.5	0.15	0.15	0.15	0.25	0.50	0.50	0.029	-0.011	0.023
10	4	0	0.5	0.8	0.5	0.15	0.15	0.15	0.50	0.00	0.00	0.031	-0.006	0.043
10	4	0	0.5	0.8	0.5	0.15	0.15	0.15	0.50	0.00	0.25	0.022	-0.008	0.038
10	4	0	0.5	0.8	0.5	0.15	0.15	0.15	0.50	0.00	0.50	0.026	0.001	0.043
10	4	0	0.5	0.8	0.5	0.15	0.15	0.15	0.50	0.25	0.00	0.032	-0.013	0.039
10	4	0	0.5	0.8	0.5	0.15	0.15	0.15	0.50	0.25	0.25	0.036	-0.008	0.041
10	4	0	0.5	0.8	0.5	0.15	0.15	0.15	0.50	0.25	0.50	0.023	-0.005	0.039
10	4	0	0.5	0.8	0.5	0.15	0.15	0.15	0.50	0.50	0.00	0.032	-0.004	0.021
10	4	0	0.5	0.8	0.5	0.15	0.15	0.15	0.50	0.50	0.25	0.029	0.002	0.026
10	4	0	0.5	0.8	0.5	0.15	0.15	0.15	0.50	0.50	0.50	0.023	-0.008	0.022
10	4	0	0.5	0.8	0.5	0.30	0.30	0.30	0.00	0.00	0.00	0.114	-0.136	0.160
10	4	0	0.5	0.8	0.5	0.30	0.30	0.30	0.00	0.00	0.25	0.109	-0.129	0.162
10	4	0	0.5	0.8	0.5	0.30	0.30	0.30	0.00	0.00	0.50	0.108	-0.140	0.164
10	4	0	0.5	0.8	0.5	0.30	0.30	0.30	0.00	0.25	0.00	0.122	-0.150	0.129
10	4	0	0.5	0.8	0.5	0.30	0.30	0.30	0.00	0.25	0.25	0.121	-0.148	0.112
10	4	0	0.5	0.8	0.5	0.30	0.30	0.30	0.00	0.25	0.50	0.122	-0.150	0.127
10	4	0	0.5	0.8	0.5	0.30	0.30	0.30	0.00	0.50	0.00	0.138	-0.155	0.101
10	4	0	0.5	0.8	0.5	0.30	0.30	0.30	0.00	0.50	0.25	0.127	-0.154	0.081
10	4	0	0.5	0.8	0.5	0.30	0.30	0.30	0.00	0.50	0.50	0.130	-0.149	0.092
10	4	0	0.5	0.8	0.5	0.30	0.30	0.30	0.25	0.00	0.00	0.108	-0.107	0.155
10	4	0	0.5	0.8	0.5	0.30	0.30	0.30	0.25	0.00	0.25	0.106	-0.107	0.151
10	4	0	0.5	0.8	0.5	0.30	0.30	0.30	0.25	0.00	0.50	0.107	-0.101	0.163
10	4	0	0.5	0.8	0.5	0.30	0.30	0.30	0.25	0.25	0.00	0.122	-0.115	0.120
10	4	0	0.5	0.8	0.5	0.30	0.30	0.30	0.25	0.25	0.25	0.128	-0.136	0.134
10	4	0	0.5	0.8	0.5	0.30	0.30	0.30	0.25	0.25	0.50	0.128	-0.120	0.133
10	4	0	0.5	0.8	0.5	0.30	0.30	0.30	0.25	0.50	0.00	0.136	-0.126	0.080
10	4	0	0.5	0.8	0.5	0.30	0.30	0.30	0.25	0.50	0.25	0.126	-0.127	0.094
10	4	0	0.5	0.8	0.5	0.30	0.30	0.30	0.25	0.50	0.50	0.130	-0.134	0.081
10	4	0	0.5	0.8	0.5	0.30	0.30	0.30	0.50	0.00	0.00	0.111	-0.088	0.164
10	4	0	0.5	0.8	0.5	0.30	0.30	0.30	0.50	0.00	0.25	0.105	-0.078	0.162
10	4	0	0.5	0.8	0.5	0.30	0.30	0.30	0.50	0.00	0.50	0.116	-0.086	0.159
10	4	0	0.5	0.8	0.5	0.30	0.30	0.30	0.50	0.25	0.00	0.126	-0.091	0.122
10	4	0	0.5	0.8	0.5	0.30	0.30	0.30	0.50	0.25	0.25	0.125	-0.092	0.130
10	4	0	0.5	0.8	0.5	0.30	0.30	0.30	0.50	0.25	0.50	0.132	-0.090	0.132
10	4	0	0.5	0.8	0.5	0.30	0.30	0.30	0.50	0.50	0.00	0.138	-0.105	0.091
10	4	0	0.5	0.8	0.5	0.30	0.30	0.30	0.50	0.50	0.25	0.129	-0.099	0.091
10	4	0	0.5	0.8	0.5	0.30	0.30	0.30	0.50	0.50	0.50	0.139	-0.099	0.094
10	4	0	0.8	0.2	0.5	0.00	0.00	0.00	0.00	0.00	0.00	-0.107	0.107	-0.032

(continued)

N	m_1	$\frac{m_2}{m_1}$	$E(C)$	$E(R)$	$E(U)$	σ_C	σ_R	σ_U	ρ_{CR}	ρ_{CU}	ρ_{RU}	Mean Bias		
												c	r	u
10	4	0	0.8	0.2	0.5	0.15	0.15	0.15	0.00	0.00	0.00	-0.076	0.086	0.014
10	4	0	0.8	0.2	0.5	0.15	0.15	0.15	0.00	0.00	0.25	-0.091	0.108	0.006
10	4	0	0.8	0.2	0.5	0.15	0.15	0.15	0.00	0.00	0.50	-0.087	0.106	0.013
10	4	0	0.8	0.2	0.5	0.15	0.15	0.15	0.00	0.25	0.00	-0.110	0.133	-0.029
10	4	0	0.8	0.2	0.5	0.15	0.15	0.15	0.00	0.25	0.25	-0.104	0.124	-0.017
10	4	0	0.8	0.2	0.5	0.15	0.15	0.15	0.00	0.25	0.50	-0.102	0.129	-0.021
10	4	0	0.8	0.2	0.5	0.15	0.15	0.15	0.00	0.50	0.00	-0.115	0.145	-0.035
10	4	0	0.8	0.2	0.5	0.15	0.15	0.15	0.00	0.50	0.25	-0.115	0.137	-0.040
10	4	0	0.8	0.2	0.5	0.15	0.15	0.15	0.00	0.50	0.50	-0.111	0.139	-0.037
10	4	0	0.8	0.2	0.5	0.15	0.15	0.15	0.25	0.00	0.00	-0.100	0.120	0.000
10	4	0	0.8	0.2	0.5	0.15	0.15	0.15	0.25	0.00	0.25	-0.084	0.096	0.011
10	4	0	0.8	0.2	0.5	0.15	0.15	0.15	0.25	0.00	0.50	-0.081	0.108	0.009
10	4	0	0.8	0.2	0.5	0.15	0.15	0.15	0.25	0.25	0.00	-0.093	0.116	-0.006
10	4	0	0.8	0.2	0.5	0.15	0.15	0.15	0.25	0.25	0.25	-0.090	0.121	-0.011
10	4	0	0.8	0.2	0.5	0.15	0.15	0.15	0.25	0.25	0.50	-0.110	0.136	-0.019
10	4	0	0.8	0.2	0.5	0.15	0.15	0.15	0.25	0.50	0.00	-0.094	0.130	-0.019
10	4	0	0.8	0.2	0.5	0.15	0.15	0.15	0.25	0.50	0.25	-0.125	0.165	-0.050
10	4	0	0.8	0.2	0.5	0.15	0.15	0.15	0.25	0.50	0.50	-0.118	0.157	-0.042
10	4	0	0.8	0.2	0.5	0.15	0.15	0.15	0.50	0.00	0.00	-0.087	0.101	0.005
10	4	0	0.8	0.2	0.5	0.15	0.15	0.15	0.50	0.00	0.25	-0.099	0.116	0.001
10	4	0	0.8	0.2	0.5	0.15	0.15	0.15	0.50	0.00	0.50	-0.076	0.107	0.017
10	4	0	0.8	0.2	0.5	0.15	0.15	0.15	0.50	0.25	0.00	-0.110	0.148	-0.028
10	4	0	0.8	0.2	0.5	0.15	0.15	0.15	0.50	0.25	0.25	-0.098	0.132	-0.011
10	4	0	0.8	0.2	0.5	0.15	0.15	0.15	0.50	0.25	0.50	-0.086	0.115	-0.003
10	4	0	0.8	0.2	0.5	0.15	0.15	0.15	0.50	0.50	0.00	-0.110	0.140	-0.033
10	4	0	0.8	0.2	0.5	0.15	0.15	0.15	0.50	0.50	0.25	-0.113	0.153	-0.038
10	4	0	0.8	0.2	0.5	0.15	0.15	0.15	0.50	0.50	0.50	-0.109	0.145	-0.033
10	4	0	0.8	0.2	0.5	0.30	0.30	0.30	0.00	0.00	0.00	-0.066	0.106	0.091
10	4	0	0.8	0.2	0.5	0.30	0.30	0.30	0.00	0.00	0.25	-0.065	0.106	0.091
10	4	0	0.8	0.2	0.5	0.30	0.30	0.30	0.00	0.00	0.50	-0.066	0.095	0.098
10	4	0	0.8	0.2	0.5	0.30	0.30	0.30	0.00	0.25	0.00	-0.100	0.142	0.010
10	4	0	0.8	0.2	0.5	0.30	0.30	0.30	0.00	0.25	0.25	-0.100	0.142	0.022
10	4	0	0.8	0.2	0.5	0.30	0.30	0.30	0.00	0.25	0.50	-0.076	0.131	0.028
10	4	0	0.8	0.2	0.5	0.30	0.30	0.30	0.00	0.50	0.00	-0.155	0.213	-0.067
10	4	0	0.8	0.2	0.5	0.30	0.30	0.30	0.00	0.50	0.25	-0.142	0.196	-0.072
10	4	0	0.8	0.2	0.5	0.30	0.30	0.30	0.00	0.50	0.50	-0.136	0.198	-0.054
10	4	0	0.8	0.2	0.5	0.30	0.30	0.30	0.25	0.00	0.00	-0.056	0.109	0.082
10	4	0	0.8	0.2	0.5	0.30	0.30	0.30	0.25	0.00	0.25	-0.063	0.110	0.083
10	4	0	0.8	0.2	0.5	0.30	0.30	0.30	0.25	0.00	0.50	-0.062	0.116	0.085

(continued)

												Mean Bias		
N	m_1	$\frac{m_2}{m_1}$	$E(C)$	$E(R)$	$E(U)$	σ_C	σ_R	σ_U	ρ_{CR}	ρ_{CU}	ρ_{RU}	c	r	u
10	4	0	0.8	0.2	0.5	0.30	0.30	0.30	0.25	0.25	0.00	-0.085	0.149	0.023
10	4	0	0.8	0.2	0.5	0.30	0.30	0.30	0.25	0.25	0.25	-0.100	0.157	0.008
10	4	0	0.8	0.2	0.5	0.30	0.30	0.30	0.25	0.25	0.50	-0.101	0.156	0.000
10	4	0	0.8	0.2	0.5	0.30	0.30	0.30	0.25	0.50	0.00	-0.149	0.235	-0.073
10	4	0	0.8	0.2	0.5	0.30	0.30	0.30	0.25	0.50	0.25	-0.147	0.230	-0.070
10	4	0	0.8	0.2	0.5	0.30	0.30	0.30	0.25	0.50	0.50	-0.149	0.229	-0.073
10	4	0	0.8	0.2	0.5	0.30	0.30	0.30	0.50	0.00	0.00	-0.067	0.132	0.080
10	4	0	0.8	0.2	0.5	0.30	0.30	0.30	0.50	0.00	0.25	-0.059	0.126	0.088
10	4	0	0.8	0.2	0.5	0.30	0.30	0.30	0.50	0.00	0.50	-0.058	0.121	0.087
10	4	0	0.8	0.2	0.5	0.30	0.30	0.30	0.50	0.25	0.00	-0.081	0.150	0.027
10	4	0	0.8	0.2	0.5	0.30	0.30	0.30	0.50	0.25	0.25	-0.085	0.164	0.019
10	4	0	0.8	0.2	0.5	0.30	0.30	0.30	0.50	0.25	0.50	-0.085	0.163	0.024
10	4	0	0.8	0.2	0.5	0.30	0.30	0.30	0.50	0.50	0.00	-0.126	0.225	-0.063
10	4	0	0.8	0.2	0.5	0.30	0.30	0.30	0.50	0.50	0.25	-0.129	0.221	-0.057
10	4	0	0.8	0.2	0.5	0.30	0.30	0.30	0.50	0.50	0.50	-0.142	0.241	-0.081
10	4	0	0.8	0.5	0.5	0.00	0.00	0.00	0.00	0.00	0.00	-0.082	0.089	-0.039
10	4	0	0.8	0.5	0.5	0.15	0.15	0.15	0.00	0.00	0.00	-0.054	0.062	0.018
10	4	0	0.8	0.5	0.5	0.15	0.15	0.15	0.00	0.00	0.25	-0.057	0.072	0.004
10	4	0	0.8	0.5	0.5	0.15	0.15	0.15	0.00	0.00	0.50	-0.051	0.063	0.020
10	4	0	0.8	0.5	0.5	0.15	0.15	0.15	0.00	0.25	0.00	-0.055	0.070	-0.008
10	4	0	0.8	0.5	0.5	0.15	0.15	0.15	0.00	0.25	0.25	-0.074	0.080	-0.022
10	4	0	0.8	0.5	0.5	0.15	0.15	0.15	0.00	0.25	0.50	-0.061	0.077	-0.016
10	4	0	0.8	0.5	0.5	0.15	0.15	0.15	0.00	0.50	0.00	-0.072	0.097	-0.037
10	4	0	0.8	0.5	0.5	0.15	0.15	0.15	0.00	0.50	0.25	-0.070	0.092	-0.029
10	4	0	0.8	0.5	0.5	0.15	0.15	0.15	0.00	0.50	0.50	-0.070	0.091	-0.034
10	4	0	0.8	0.5	0.5	0.15	0.15	0.15	0.25	0.00	0.00	-0.057	0.079	0.003
10	4	0	0.8	0.5	0.5	0.15	0.15	0.15	0.25	0.00	0.25	-0.054	0.078	0.002
10	4	0	0.8	0.5	0.5	0.15	0.15	0.15	0.25	0.00	0.50	-0.045	0.066	0.021
10	4	0	0.8	0.5	0.5	0.15	0.15	0.15	0.25	0.25	0.00	-0.070	0.096	-0.021
10	4	0	0.8	0.5	0.5	0.15	0.15	0.15	0.25	0.25	0.25	-0.063	0.084	-0.022
10	4	0	0.8	0.5	0.5	0.15	0.15	0.15	0.25	0.25	0.50	-0.071	0.090	-0.020
10	4	0	0.8	0.5	0.5	0.15	0.15	0.15	0.25	0.50	0.00	-0.076	0.102	-0.053
10	4	0	0.8	0.5	0.5	0.15	0.15	0.15	0.25	0.50	0.25	-0.075	0.104	-0.034
10	4	0	0.8	0.5	0.5	0.15	0.15	0.15	0.25	0.50	0.50	-0.070	0.103	-0.034
10	4	0	0.8	0.5	0.5	0.15	0.15	0.15	0.50	0.00	0.00	-0.047	0.073	0.011
10	4	0	0.8	0.5	0.5	0.15	0.15	0.15	0.50	0.00	0.25	-0.057	0.085	0.003
10	4	0	0.8	0.5	0.5	0.15	0.15	0.15	0.50	0.00	0.50	-0.056	0.079	0.009
10	4	0	0.8	0.5	0.5	0.15	0.15	0.15	0.50	0.25	0.00	-0.056	0.076	-0.005
10	4	0	0.8	0.5	0.5	0.15	0.15	0.15	0.50	0.25	0.25	-0.061	0.090	-0.022

(continued)

												Mean Bias		
N	m_1	$\frac{m_2}{m_1}$	$E(C)$	$E(R)$	$E(U)$	σ_C	σ_R	σ_U	ρ_{CR}	ρ_{CU}	ρ_{RU}	c	r	u
10	4	0	0.8	0.5	0.5	0.15	0.15	0.15	0.50	0.25	0.50	-0.061	0.093	-0.008
10	4	0	0.8	0.5	0.5	0.15	0.15	0.15	0.50	0.50	0.00	-0.071	0.098	-0.032
10	4	0	0.8	0.5	0.5	0.15	0.15	0.15	0.50	0.50	0.25	-0.070	0.101	-0.043
10	4	0	0.8	0.5	0.5	0.15	0.15	0.15	0.50	0.50	0.50	-0.083	0.120	-0.059
10	4	0	0.8	0.5	0.5	0.30	0.30	0.30	0.00	0.00	0.00	-0.028	0.049	0.100
10	4	0	0.8	0.5	0.5	0.30	0.30	0.30	0.00	0.00	0.25	-0.033	0.055	0.084
10	4	0	0.8	0.5	0.5	0.30	0.30	0.30	0.00	0.00	0.50	-0.015	0.036	0.099
10	4	0	0.8	0.5	0.5	0.30	0.30	0.30	0.00	0.25	0.00	-0.051	0.086	0.021
10	4	0	0.8	0.5	0.5	0.30	0.30	0.30	0.00	0.25	0.25	-0.049	0.079	0.015
10	4	0	0.8	0.5	0.5	0.30	0.30	0.30	0.00	0.25	0.50	-0.044	0.080	0.004
10	4	0	0.8	0.5	0.5	0.30	0.30	0.30	0.00	0.50	0.00	-0.080	0.128	-0.077
10	4	0	0.8	0.5	0.5	0.30	0.30	0.30	0.00	0.50	0.25	-0.080	0.125	-0.070
10	4	0	0.8	0.5	0.5	0.30	0.30	0.30	0.00	0.50	0.50	-0.080	0.134	-0.068
10	4	0	0.8	0.5	0.5	0.30	0.30	0.30	0.25	0.00	0.00	-0.027	0.069	0.094
10	4	0	0.8	0.5	0.5	0.30	0.30	0.30	0.25	0.00	0.25	-0.026	0.072	0.087
10	4	0	0.8	0.5	0.5	0.30	0.30	0.30	0.25	0.00	0.50	-0.023	0.070	0.085
10	4	0	0.8	0.5	0.5	0.30	0.30	0.30	0.25	0.25	0.00	-0.036	0.087	0.021
10	4	0	0.8	0.5	0.5	0.30	0.30	0.30	0.25	0.25	0.25	-0.039	0.091	0.021
10	4	0	0.8	0.5	0.5	0.30	0.30	0.30	0.25	0.25	0.50	-0.037	0.098	0.011
10	4	0	0.8	0.5	0.5	0.30	0.30	0.30	0.25	0.50	0.00	-0.065	0.131	-0.062
10	4	0	0.8	0.5	0.5	0.30	0.30	0.30	0.25	0.50	0.25	-0.072	0.143	-0.072
10	4	0	0.8	0.5	0.5	0.30	0.30	0.30	0.25	0.50	0.50	-0.064	0.130	-0.073
10	4	0	0.8	0.5	0.5	0.30	0.30	0.30	0.50	0.00	0.00	-0.021	0.094	0.085
10	4	0	0.8	0.5	0.5	0.30	0.30	0.30	0.50	0.00	0.25	-0.027	0.089	0.083
10	4	0	0.8	0.5	0.5	0.30	0.30	0.30	0.50	0.00	0.50	-0.032	0.098	0.070
10	4	0	0.8	0.5	0.5	0.30	0.30	0.30	0.50	0.25	0.00	-0.029	0.095	0.035
10	4	0	0.8	0.5	0.5	0.30	0.30	0.30	0.50	0.25	0.25	-0.036	0.115	0.023
10	4	0	0.8	0.5	0.5	0.30	0.30	0.30	0.50	0.25	0.50	-0.033	0.113	0.010
10	4	0	0.8	0.5	0.5	0.30	0.30	0.30	0.50	0.50	0.00	-0.059	0.139	-0.061
10	4	0	0.8	0.5	0.5	0.30	0.30	0.30	0.50	0.50	0.25	-0.069	0.148	-0.079
10	4	0	0.8	0.5	0.5	0.30	0.30	0.30	0.50	0.50	0.50	-0.056	0.155	-0.060
10	4	0	0.8	0.8	0.5	0.00	0.00	0.00	0.00	0.00	0.00	-0.031	0.044	-0.013
10	4	0	0.8	0.8	0.5	0.15	0.15	0.15	0.00	0.00	0.00	-0.019	0.022	0.035
10	4	0	0.8	0.8	0.5	0.15	0.15	0.15	0.00	0.00	0.25	-0.022	0.028	0.012
10	4	0	0.8	0.8	0.5	0.15	0.15	0.15	0.00	0.00	0.50	-0.016	0.028	0.015
10	4	0	0.8	0.8	0.5	0.15	0.15	0.15	0.00	0.25	0.00	-0.022	0.029	-0.006
10	4	0	0.8	0.8	0.5	0.15	0.15	0.15	0.00	0.25	0.25	-0.020	0.031	-0.003
10	4	0	0.8	0.8	0.5	0.15	0.15	0.15	0.00	0.25	0.50	-0.027	0.038	-0.014
10	4	0	0.8	0.8	0.5	0.15	0.15	0.15	0.00	0.50	0.00	-0.029	0.041	-0.035

(continued)

												Mean Bias		
N	m_1	$\frac{m_2}{m_1}$	$E(C)$	$E(R)$	$E(U)$	σ_C	σ_R	σ_U	ρ_{CR}	ρ_{CU}	ρ_{RU}	c	r	u
10	4	0	0.8	0.8	0.5	0.15	0.15	0.15	0.00	0.50	0.25	-0.021	0.027	-0.008
10	4	0	0.8	0.8	0.5	0.15	0.15	0.15	0.00	0.50	0.50	-0.021	0.029	-0.029
10	4	0	0.8	0.8	0.5	0.15	0.15	0.15	0.25	0.00	0.00	-0.018	0.029	0.030
10	4	0	0.8	0.8	0.5	0.15	0.15	0.15	0.25	0.00	0.25	-0.018	0.038	0.013
10	4	0	0.8	0.8	0.5	0.15	0.15	0.15	0.25	0.00	0.50	-0.019	0.034	0.025
10	4	0	0.8	0.8	0.5	0.15	0.15	0.15	0.25	0.25	0.00	-0.019	0.033	-0.007
10	4	0	0.8	0.8	0.5	0.15	0.15	0.15	0.25	0.25	0.25	-0.025	0.040	-0.010
10	4	0	0.8	0.8	0.5	0.15	0.15	0.15	0.25	0.25	0.50	-0.022	0.036	-0.003
10	4	0	0.8	0.8	0.5	0.15	0.15	0.15	0.25	0.50	0.00	-0.023	0.036	-0.033
10	4	0	0.8	0.8	0.5	0.15	0.15	0.15	0.25	0.50	0.25	-0.020	0.040	-0.022
10	4	0	0.8	0.8	0.5	0.15	0.15	0.15	0.25	0.50	0.50	-0.023	0.042	-0.025
10	4	0	0.8	0.8	0.5	0.15	0.15	0.15	0.50	0.00	0.00	-0.015	0.036	0.011
10	4	0	0.8	0.8	0.5	0.15	0.15	0.15	0.50	0.00	0.25	-0.016	0.036	0.023
10	4	0	0.8	0.8	0.5	0.15	0.15	0.15	0.50	0.00	0.50	-0.020	0.043	0.016
10	4	0	0.8	0.8	0.5	0.15	0.15	0.15	0.50	0.25	0.00	-0.017	0.041	-0.004
10	4	0	0.8	0.8	0.5	0.15	0.15	0.15	0.50	0.25	0.25	-0.018	0.037	-0.001
10	4	0	0.8	0.8	0.5	0.15	0.15	0.15	0.50	0.25	0.50	-0.017	0.040	-0.001
10	4	0	0.8	0.8	0.5	0.15	0.15	0.15	0.50	0.50	0.00	-0.023	0.044	-0.027
10	4	0	0.8	0.8	0.5	0.15	0.15	0.15	0.50	0.50	0.25	-0.022	0.052	-0.034
10	4	0	0.8	0.8	0.5	0.15	0.15	0.15	0.50	0.50	0.50	-0.019	0.041	-0.022
10	4	0	0.8	0.8	0.5	0.30	0.30	0.30	0.00	0.00	0.00	0.007	-0.005	0.111
10	4	0	0.8	0.8	0.5	0.30	0.30	0.30	0.00	0.00	0.25	0.009	-0.001	0.096
10	4	0	0.8	0.8	0.5	0.30	0.30	0.30	0.00	0.00	0.50	0.008	-0.011	0.127
10	4	0	0.8	0.8	0.5	0.30	0.30	0.30	0.00	0.25	0.00	0.009	0.008	0.025
10	4	0	0.8	0.8	0.5	0.30	0.30	0.30	0.00	0.25	0.25	0.007	0.013	0.033
10	4	0	0.8	0.8	0.5	0.30	0.30	0.30	0.00	0.25	0.50	0.004	-0.001	0.037
10	4	0	0.8	0.8	0.5	0.30	0.30	0.30	0.00	0.50	0.00	-0.001	0.013	-0.056
10	4	0	0.8	0.8	0.5	0.30	0.30	0.30	0.00	0.50	0.25	-0.003	0.024	-0.059
10	4	0	0.8	0.8	0.5	0.30	0.30	0.30	0.00	0.50	0.50	0.001	0.011	-0.049
10	4	0	0.8	0.8	0.5	0.30	0.30	0.30	0.25	0.00	0.00	0.011	0.017	0.109
10	4	0	0.8	0.8	0.5	0.30	0.30	0.30	0.25	0.00	0.25	0.006	0.017	0.088
10	4	0	0.8	0.8	0.5	0.30	0.30	0.30	0.25	0.00	0.50	0.019	0.003	0.122
10	4	0	0.8	0.8	0.5	0.30	0.30	0.30	0.25	0.25	0.00	0.020	0.014	0.045
10	4	0	0.8	0.8	0.5	0.30	0.30	0.30	0.25	0.25	0.25	0.013	0.024	0.034
10	4	0	0.8	0.8	0.5	0.30	0.30	0.30	0.25	0.25	0.50	0.015	0.016	0.038
10	4	0	0.8	0.8	0.5	0.30	0.30	0.30	0.25	0.50	0.00	0.011	0.030	-0.046
10	4	0	0.8	0.8	0.5	0.30	0.30	0.30	0.25	0.50	0.25	0.018	0.019	-0.020
10	4	0	0.8	0.8	0.5	0.30	0.30	0.30	0.25	0.50	0.50	0.012	0.031	-0.047
10	4	0	0.8	0.8	0.5	0.30	0.30	0.30	0.50	0.00	0.00	0.018	0.026	0.118

(continued)

												Mean Bias		
N	m_1	$\frac{m_2}{m_1}$	$E(C)$	$E(R)$	$E(U)$	σ_C	σ_R	σ_U	ρ_{CR}	ρ_{CU}	ρ_{RU}	c	r	u
10	4	0	0.8	0.8	0.5	0.30	0.30	0.30	0.50	0.00	0.25	0.015	0.034	0.107
10	4	0	0.8	0.8	0.5	0.30	0.30	0.30	0.50	0.00	0.50	0.015	0.036	0.105
10	4	0	0.8	0.8	0.5	0.30	0.30	0.30	0.50	0.25	0.00	0.013	0.031	0.033
10	4	0	0.8	0.8	0.5	0.30	0.30	0.30	0.50	0.25	0.25	0.016	0.038	0.030
10	4	0	0.8	0.8	0.5	0.30	0.30	0.30	0.50	0.25	0.50	0.016	0.032	0.038
10	4	0	0.8	0.8	0.5	0.30	0.30	0.30	0.50	0.50	0.00	0.011	0.043	-0.047
10	4	0	0.8	0.8	0.5	0.30	0.30	0.30	0.50	0.50	0.25	0.018	0.040	-0.042
10	4	0	0.8	0.8	0.5	0.30	0.30	0.30	0.50	0.50	0.50	0.008	0.044	-0.043
10	4	1	0.2	0.2	0.5	0.00	0.00	0.00	0.00	0.00	0.00	-0.005	0.072	-0.001
10	4	1	0.2	0.2	0.5	0.15	0.15	0.15	0.00	0.00	0.00	0.027	0.037	0.014
10	4	1	0.2	0.2	0.5	0.15	0.15	0.15	0.00	0.00	0.25	0.021	0.029	0.014
10	4	1	0.2	0.2	0.5	0.15	0.15	0.15	0.00	0.00	0.50	0.029	0.014	0.017
10	4	1	0.2	0.2	0.5	0.15	0.15	0.15	0.00	0.25	0.00	0.028	0.026	0.011
10	4	1	0.2	0.2	0.5	0.15	0.15	0.15	0.00	0.25	0.25	0.034	0.020	0.013
10	4	1	0.2	0.2	0.5	0.15	0.15	0.15	0.00	0.25	0.50	0.034	0.022	0.009
10	4	1	0.2	0.2	0.5	0.15	0.15	0.15	0.00	0.50	0.00	0.033	0.027	0.006
10	4	1	0.2	0.2	0.5	0.15	0.15	0.15	0.00	0.50	0.25	0.038	0.016	0.012
10	4	1	0.2	0.2	0.5	0.15	0.15	0.15	0.00	0.50	0.50	0.035	0.018	0.009
10	4	1	0.2	0.2	0.5	0.15	0.15	0.15	0.25	0.00	0.00	0.035	0.036	0.014
10	4	1	0.2	0.2	0.5	0.15	0.15	0.15	0.25	0.00	0.25	0.024	0.051	0.013
10	4	1	0.2	0.2	0.5	0.15	0.15	0.15	0.25	0.00	0.50	0.030	0.037	0.018
10	4	1	0.2	0.2	0.5	0.15	0.15	0.15	0.25	0.25	0.00	0.029	0.048	0.012
10	4	1	0.2	0.2	0.5	0.15	0.15	0.15	0.25	0.25	0.25	0.038	0.046	0.014
10	4	1	0.2	0.2	0.5	0.15	0.15	0.15	0.25	0.25	0.50	0.037	0.043	0.009
10	4	1	0.2	0.2	0.5	0.15	0.15	0.15	0.25	0.50	0.00	0.041	0.041	0.014
10	4	1	0.2	0.2	0.5	0.15	0.15	0.15	0.25	0.50	0.25	0.038	0.039	0.011
10	4	1	0.2	0.2	0.5	0.15	0.15	0.15	0.25	0.50	0.50	0.037	0.042	0.009
10	4	1	0.2	0.2	0.5	0.15	0.15	0.15	0.50	0.00	0.00	0.037	0.069	0.016
10	4	1	0.2	0.2	0.5	0.15	0.15	0.15	0.50	0.00	0.25	0.032	0.074	0.014
10	4	1	0.2	0.2	0.5	0.15	0.15	0.15	0.50	0.00	0.50	0.031	0.080	0.021
10	4	1	0.2	0.2	0.5	0.15	0.15	0.15	0.50	0.25	0.00	0.038	0.076	0.014
10	4	1	0.2	0.2	0.5	0.15	0.15	0.15	0.50	0.25	0.25	0.034	0.062	0.014
10	4	1	0.2	0.2	0.5	0.15	0.15	0.15	0.50	0.25	0.50	0.035	0.071	0.011
10	4	1	0.2	0.2	0.5	0.15	0.15	0.15	0.50	0.50	0.00	0.033	0.067	0.013
10	4	1	0.2	0.2	0.5	0.15	0.15	0.15	0.50	0.50	0.25	0.038	0.058	0.010
10	4	1	0.2	0.2	0.5	0.15	0.15	0.15	0.50	0.50	0.50	0.040	0.060	0.007
10	4	1	0.2	0.2	0.5	0.30	0.30	0.30	0.00	0.00	0.00	0.126	-0.076	0.056
10	4	1	0.2	0.2	0.5	0.30	0.30	0.30	0.00	0.00	0.25	0.127	-0.076	0.050
10	4	1	0.2	0.2	0.5	0.30	0.30	0.30	0.00	0.00	0.50	0.124	-0.072	0.056

(continued)

												Mean Bias		
N	m_1	$\frac{m_2}{m_1}$	$E(C)$	$E(R)$	$E(U)$	σ_C	σ_R	σ_U	ρ_{CR}	ρ_{CU}	ρ_{RU}	c	r	u
10	4	1	0.2	0.2	0.5	0.30	0.30	0.30	0.00	0.25	0.00	0.151	-0.075	0.046
10	4	1	0.2	0.2	0.5	0.30	0.30	0.30	0.00	0.25	0.25	0.145	-0.073	0.051
10	4	1	0.2	0.2	0.5	0.30	0.30	0.30	0.00	0.25	0.50	0.148	-0.088	0.046
10	4	1	0.2	0.2	0.5	0.30	0.30	0.30	0.00	0.50	0.00	0.170	-0.084	0.043
10	4	1	0.2	0.2	0.5	0.30	0.30	0.30	0.00	0.50	0.25	0.160	-0.080	0.035
10	4	1	0.2	0.2	0.5	0.30	0.30	0.30	0.00	0.50	0.50	0.154	-0.085	0.040
10	4	1	0.2	0.2	0.5	0.30	0.30	0.30	0.25	0.00	0.00	0.130	-0.013	0.062
10	4	1	0.2	0.2	0.5	0.30	0.30	0.30	0.25	0.00	0.25	0.126	-0.022	0.054
10	4	1	0.2	0.2	0.5	0.30	0.30	0.30	0.25	0.00	0.50	0.136	-0.018	0.055
10	4	1	0.2	0.2	0.5	0.30	0.30	0.30	0.25	0.25	0.00	0.151	-0.028	0.048
10	4	1	0.2	0.2	0.5	0.30	0.30	0.30	0.25	0.25	0.25	0.150	-0.043	0.050
10	4	1	0.2	0.2	0.5	0.30	0.30	0.30	0.25	0.25	0.50	0.151	-0.027	0.046
10	4	1	0.2	0.2	0.5	0.30	0.30	0.30	0.25	0.50	0.00	0.161	-0.039	0.046
10	4	1	0.2	0.2	0.5	0.30	0.30	0.30	0.25	0.50	0.25	0.157	-0.034	0.031
10	4	1	0.2	0.2	0.5	0.30	0.30	0.30	0.25	0.50	0.50	0.167	-0.042	0.047
10	4	1	0.2	0.2	0.5	0.30	0.30	0.30	0.50	0.00	0.00	0.139	0.039	0.062
10	4	1	0.2	0.2	0.5	0.30	0.30	0.30	0.50	0.00	0.25	0.130	0.040	0.058
10	4	1	0.2	0.2	0.5	0.30	0.30	0.30	0.50	0.00	0.50	0.132	0.034	0.055
10	4	1	0.2	0.2	0.5	0.30	0.30	0.30	0.50	0.25	0.00	0.153	0.021	0.053
10	4	1	0.2	0.2	0.5	0.30	0.30	0.30	0.50	0.25	0.25	0.152	0.031	0.054
10	4	1	0.2	0.2	0.5	0.30	0.30	0.30	0.50	0.25	0.50	0.145	0.022	0.051
10	4	1	0.2	0.2	0.5	0.30	0.30	0.30	0.50	0.50	0.00	0.171	0.017	0.040
10	4	1	0.2	0.2	0.5	0.30	0.30	0.30	0.50	0.50	0.25	0.158	0.015	0.039
10	4	1	0.2	0.2	0.5	0.30	0.30	0.30	0.50	0.50	0.50	0.155	0.023	0.039
10	4	1	0.2	0.5	0.5	0.00	0.00	0.00	0.00	0.00	0.00	0.007	0.070	0.004
10	4	1	0.2	0.5	0.5	0.15	0.15	0.15	0.00	0.00	0.00	0.038	-0.003	0.020
10	4	1	0.2	0.5	0.5	0.15	0.15	0.15	0.00	0.00	0.25	0.035	-0.004	0.015
10	4	1	0.2	0.5	0.5	0.15	0.15	0.15	0.00	0.00	0.50	0.036	-0.012	0.017
10	4	1	0.2	0.5	0.5	0.15	0.15	0.15	0.00	0.25	0.00	0.043	-0.022	0.016
10	4	1	0.2	0.5	0.5	0.15	0.15	0.15	0.00	0.25	0.25	0.038	-0.019	0.015
10	4	1	0.2	0.5	0.5	0.15	0.15	0.15	0.00	0.25	0.50	0.035	-0.003	0.013
10	4	1	0.2	0.5	0.5	0.15	0.15	0.15	0.00	0.50	0.00	0.042	-0.021	0.009
10	4	1	0.2	0.5	0.5	0.15	0.15	0.15	0.00	0.50	0.25	0.044	-0.005	0.014
10	4	1	0.2	0.5	0.5	0.15	0.15	0.15	0.00	0.50	0.50	0.046	-0.020	0.013
10	4	1	0.2	0.5	0.5	0.15	0.15	0.15	0.25	0.00	0.00	0.038	0.016	0.018
10	4	1	0.2	0.5	0.5	0.15	0.15	0.15	0.25	0.00	0.25	0.042	0.009	0.018
10	4	1	0.2	0.5	0.5	0.15	0.15	0.15	0.25	0.00	0.50	0.040	0.024	0.013
10	4	1	0.2	0.5	0.5	0.15	0.15	0.15	0.25	0.25	0.00	0.047	0.007	0.020
10	4	1	0.2	0.5	0.5	0.15	0.15	0.15	0.25	0.25	0.25	0.030	0.027	0.007

(continued)

												Mean Bias		
N	m_1	$\frac{m_2}{m_1}$	$E(C)$	$E(R)$	$E(U)$	σ_C	σ_R	σ_U	ρ_{CR}	ρ_{CU}	ρ_{RU}	c	r	u
10	4	1	0.2	0.5	0.5	0.15	0.15	0.15	0.25	0.25	0.50	0.040	-0.003	0.015
10	4	1	0.2	0.5	0.5	0.15	0.15	0.15	0.25	0.50	0.00	0.044	0.003	0.011
10	4	1	0.2	0.5	0.5	0.15	0.15	0.15	0.25	0.50	0.25	0.047	-0.008	0.011
10	4	1	0.2	0.5	0.5	0.15	0.15	0.15	0.25	0.50	0.50	0.041	0.017	0.007
10	4	1	0.2	0.5	0.5	0.15	0.15	0.15	0.50	0.00	0.00	0.036	0.042	0.012
10	4	1	0.2	0.5	0.5	0.15	0.15	0.15	0.50	0.00	0.25	0.034	0.041	0.017
10	4	1	0.2	0.5	0.5	0.15	0.15	0.15	0.50	0.00	0.50	0.036	0.034	0.021
10	4	1	0.2	0.5	0.5	0.15	0.15	0.15	0.50	0.25	0.00	0.039	0.031	0.011
10	4	1	0.2	0.5	0.5	0.15	0.15	0.15	0.50	0.25	0.25	0.039	0.028	0.014
10	4	1	0.2	0.5	0.5	0.15	0.15	0.15	0.50	0.25	0.50	0.042	0.026	0.016
10	4	1	0.2	0.5	0.5	0.15	0.15	0.15	0.50	0.50	0.00	0.047	0.024	0.011
10	4	1	0.2	0.5	0.5	0.15	0.15	0.15	0.50	0.50	0.25	0.045	0.021	0.011
10	4	1	0.2	0.5	0.5	0.15	0.15	0.15	0.50	0.50	0.50	0.050	0.018	0.012
10	4	1	0.2	0.5	0.5	0.30	0.30	0.30	0.00	0.00	0.00	0.142	-0.189	0.058
10	4	1	0.2	0.5	0.5	0.30	0.30	0.30	0.00	0.00	0.25	0.133	-0.186	0.057
10	4	1	0.2	0.5	0.5	0.30	0.30	0.30	0.00	0.00	0.50	0.137	-0.187	0.056
10	4	1	0.2	0.5	0.5	0.30	0.30	0.30	0.00	0.25	0.00	0.145	-0.193	0.037
10	4	1	0.2	0.5	0.5	0.30	0.30	0.30	0.00	0.25	0.25	0.154	-0.208	0.054
10	4	1	0.2	0.5	0.5	0.30	0.30	0.30	0.00	0.25	0.50	0.148	-0.203	0.047
10	4	1	0.2	0.5	0.5	0.30	0.30	0.30	0.00	0.50	0.00	0.162	-0.219	0.044
10	4	1	0.2	0.5	0.5	0.30	0.30	0.30	0.00	0.50	0.25	0.165	-0.219	0.041
10	4	1	0.2	0.5	0.5	0.30	0.30	0.30	0.00	0.50	0.50	0.163	-0.204	0.049
10	4	1	0.2	0.5	0.5	0.30	0.30	0.30	0.25	0.00	0.00	0.134	-0.127	0.064
10	4	1	0.2	0.5	0.5	0.30	0.30	0.30	0.25	0.00	0.25	0.135	-0.141	0.063
10	4	1	0.2	0.5	0.5	0.30	0.30	0.30	0.25	0.00	0.50	0.132	-0.134	0.060
10	4	1	0.2	0.5	0.5	0.30	0.30	0.30	0.25	0.25	0.00	0.148	-0.142	0.047
10	4	1	0.2	0.5	0.5	0.30	0.30	0.30	0.25	0.25	0.25	0.153	-0.154	0.046
10	4	1	0.2	0.5	0.5	0.30	0.30	0.30	0.25	0.25	0.50	0.147	-0.155	0.052
10	4	1	0.2	0.5	0.5	0.30	0.30	0.30	0.25	0.50	0.00	0.162	-0.176	0.043
10	4	1	0.2	0.5	0.5	0.30	0.30	0.30	0.25	0.50	0.25	0.161	-0.156	0.034
10	4	1	0.2	0.5	0.5	0.30	0.30	0.30	0.25	0.50	0.50	0.167	-0.150	0.043
10	4	1	0.2	0.5	0.5	0.30	0.30	0.30	0.50	0.00	0.00	0.132	-0.071	0.058
10	4	1	0.2	0.5	0.5	0.30	0.30	0.30	0.50	0.00	0.25	0.126	-0.073	0.054
10	4	1	0.2	0.5	0.5	0.30	0.30	0.30	0.50	0.00	0.50	0.139	-0.088	0.062
10	4	1	0.2	0.5	0.5	0.30	0.30	0.30	0.50	0.25	0.00	0.149	-0.094	0.051
10	4	1	0.2	0.5	0.5	0.30	0.30	0.30	0.50	0.25	0.25	0.151	-0.094	0.054
10	4	1	0.2	0.5	0.5	0.30	0.30	0.30	0.50	0.25	0.50	0.151	-0.113	0.053
10	4	1	0.2	0.5	0.5	0.30	0.30	0.30	0.50	0.50	0.00	0.163	-0.126	0.042
10	4	1	0.2	0.5	0.5	0.30	0.30	0.30	0.50	0.50	0.25	0.163	-0.110	0.044

(continued)

												Mean Bias		
N	m_1	$\frac{m_2}{m_1}$	$E(C)$	$E(R)$	$E(U)$	σ_C	σ_R	σ_U	ρ_{CR}	ρ_{CU}	ρ_{RU}	c	r	u
10	4	1	0.2	0.5	0.5	0.30	0.30	0.30	0.50	0.50	0.50	0.162	-0.110	0.042
10	4	1	0.2	0.8	0.5	0.00	0.00	0.00	0.00	0.00	0.00	0.022	-0.021	0.009
10	4	1	0.2	0.8	0.5	0.15	0.15	0.15	0.00	0.00	0.00	0.052	-0.119	0.025
10	4	1	0.2	0.8	0.5	0.15	0.15	0.15	0.00	0.00	0.25	0.039	-0.080	0.022
10	4	1	0.2	0.8	0.5	0.15	0.15	0.15	0.00	0.00	0.50	0.048	-0.096	0.021
10	4	1	0.2	0.8	0.5	0.15	0.15	0.15	0.00	0.25	0.00	0.046	-0.098	0.012
10	4	1	0.2	0.8	0.5	0.15	0.15	0.15	0.00	0.25	0.25	0.051	-0.119	0.021
10	4	1	0.2	0.8	0.5	0.15	0.15	0.15	0.00	0.25	0.50	0.047	-0.109	0.017
10	4	1	0.2	0.8	0.5	0.15	0.15	0.15	0.00	0.50	0.00	0.054	-0.108	0.015
10	4	1	0.2	0.8	0.5	0.15	0.15	0.15	0.00	0.50	0.25	0.049	-0.109	0.016
10	4	1	0.2	0.8	0.5	0.15	0.15	0.15	0.00	0.50	0.50	0.050	-0.112	0.015
10	4	1	0.2	0.8	0.5	0.15	0.15	0.15	0.25	0.00	0.00	0.046	-0.093	0.015
10	4	1	0.2	0.8	0.5	0.15	0.15	0.15	0.25	0.00	0.25	0.047	-0.080	0.020
10	4	1	0.2	0.8	0.5	0.15	0.15	0.15	0.25	0.00	0.50	0.047	-0.081	0.021
10	4	1	0.2	0.8	0.5	0.15	0.15	0.15	0.25	0.25	0.00	0.053	-0.084	0.018
10	4	1	0.2	0.8	0.5	0.15	0.15	0.15	0.25	0.25	0.25	0.049	-0.091	0.016
10	4	1	0.2	0.8	0.5	0.15	0.15	0.15	0.25	0.25	0.50	0.048	-0.090	0.020
10	4	1	0.2	0.8	0.5	0.15	0.15	0.15	0.25	0.50	0.00	0.055	-0.107	0.016
10	4	1	0.2	0.8	0.5	0.15	0.15	0.15	0.25	0.50	0.25	0.056	-0.106	0.017
10	4	1	0.2	0.8	0.5	0.15	0.15	0.15	0.25	0.50	0.50	0.052	-0.097	0.015
10	4	1	0.2	0.8	0.5	0.15	0.15	0.15	0.50	0.00	0.00	0.049	-0.073	0.018
10	4	1	0.2	0.8	0.5	0.15	0.15	0.15	0.50	0.00	0.25	0.047	-0.064	0.019
10	4	1	0.2	0.8	0.5	0.15	0.15	0.15	0.50	0.00	0.50	0.046	-0.083	0.023
10	4	1	0.2	0.8	0.5	0.15	0.15	0.15	0.50	0.25	0.00	0.046	-0.071	0.017
10	4	1	0.2	0.8	0.5	0.15	0.15	0.15	0.50	0.25	0.25	0.051	-0.071	0.020
10	4	1	0.2	0.8	0.5	0.15	0.15	0.15	0.50	0.25	0.50	0.048	-0.073	0.018
10	4	1	0.2	0.8	0.5	0.15	0.15	0.15	0.50	0.50	0.00	0.052	-0.066	0.013
10	4	1	0.2	0.8	0.5	0.15	0.15	0.15	0.50	0.50	0.25	0.055	-0.091	0.010
10	4	1	0.2	0.8	0.5	0.15	0.15	0.15	0.50	0.50	0.50	0.053	-0.079	0.016
10	4	1	0.2	0.8	0.5	0.30	0.30	0.30	0.00	0.00	0.00	0.142	-0.316	0.051
10	4	1	0.2	0.8	0.5	0.30	0.30	0.30	0.00	0.00	0.25	0.136	-0.323	0.063
10	4	1	0.2	0.8	0.5	0.30	0.30	0.30	0.00	0.00	0.50	0.139	-0.329	0.058
10	4	1	0.2	0.8	0.5	0.30	0.30	0.30	0.00	0.25	0.00	0.144	-0.324	0.052
10	4	1	0.2	0.8	0.5	0.30	0.30	0.30	0.00	0.25	0.25	0.152	-0.333	0.046
10	4	1	0.2	0.8	0.5	0.30	0.30	0.30	0.00	0.25	0.50	0.149	-0.328	0.051
10	4	1	0.2	0.8	0.5	0.30	0.30	0.30	0.00	0.50	0.00	0.170	-0.345	0.049
10	4	1	0.2	0.8	0.5	0.30	0.30	0.30	0.00	0.50	0.25	0.170	-0.364	0.040
10	4	1	0.2	0.8	0.5	0.30	0.30	0.30	0.00	0.50	0.50	0.156	-0.361	0.042
10	4	1	0.2	0.8	0.5	0.30	0.30	0.30	0.25	0.00	0.00	0.134	-0.268	0.057

(continued)

N	m_1	$\frac{m_2}{m_1}$	$E(C)$	$E(R)$	$E(U)$	σ_C	σ_R	σ_U	ρ_{CR}	ρ_{CU}	ρ_{RU}	Mean Bias c	Mean Bias r	Mean Bias u
10	4	1	0.2	0.8	0.5	0.30	0.30	0.30	0.25	0.00	0.25	0.137	-0.265	0.056
10	4	1	0.2	0.8	0.5	0.30	0.30	0.30	0.25	0.00	0.50	0.138	-0.264	0.057
10	4	1	0.2	0.8	0.5	0.30	0.30	0.30	0.25	0.25	0.00	0.154	-0.289	0.051
10	4	1	0.2	0.8	0.5	0.30	0.30	0.30	0.25	0.25	0.25	0.148	-0.287	0.049
10	4	1	0.2	0.8	0.5	0.30	0.30	0.30	0.25	0.25	0.50	0.157	-0.286	0.056
10	4	1	0.2	0.8	0.5	0.30	0.30	0.30	0.25	0.50	0.00	0.166	-0.310	0.038
10	4	1	0.2	0.8	0.5	0.30	0.30	0.30	0.25	0.50	0.25	0.166	-0.319	0.045
10	4	1	0.2	0.8	0.5	0.30	0.30	0.30	0.25	0.50	0.50	0.169	-0.311	0.043
10	4	1	0.2	0.8	0.5	0.30	0.30	0.30	0.50	0.00	0.00	0.136	-0.225	0.056
10	4	1	0.2	0.8	0.5	0.30	0.30	0.30	0.50	0.00	0.25	0.143	-0.233	0.064
10	4	1	0.2	0.8	0.5	0.30	0.30	0.30	0.50	0.00	0.50	0.134	-0.231	0.062
10	4	1	0.2	0.8	0.5	0.30	0.30	0.30	0.50	0.25	0.00	0.147	-0.268	0.051
10	4	1	0.2	0.8	0.5	0.30	0.30	0.30	0.50	0.25	0.25	0.158	-0.250	0.046
10	4	1	0.2	0.8	0.5	0.30	0.30	0.30	0.50	0.25	0.50	0.149	-0.264	0.048
10	4	1	0.2	0.8	0.5	0.30	0.30	0.30	0.50	0.50	0.00	0.161	-0.284	0.041
10	4	1	0.2	0.8	0.5	0.30	0.30	0.30	0.50	0.50	0.25	0.169	-0.288	0.042
10	4	1	0.2	0.8	0.5	0.30	0.30	0.30	0.50	0.50	0.50	0.161	-0.288	0.044
10	4	1	0.5	0.2	0.5	0.00	0.00	0.00	0.00	0.00	0.00	-0.009	0.014	-0.005
10	4	1	0.5	0.2	0.5	0.15	0.15	0.15	0.00	0.00	0.00	0.014	0.004	0.013
10	4	1	0.5	0.2	0.5	0.15	0.15	0.15	0.00	0.00	0.25	0.013	0.005	0.011
10	4	1	0.5	0.2	0.5	0.15	0.15	0.15	0.00	0.00	0.50	0.023	-0.004	0.013
10	4	1	0.5	0.2	0.5	0.15	0.15	0.15	0.00	0.25	0.00	0.014	-0.005	0.007
10	4	1	0.5	0.2	0.5	0.15	0.15	0.15	0.00	0.25	0.25	0.022	-0.005	0.010
10	4	1	0.5	0.2	0.5	0.15	0.15	0.15	0.00	0.25	0.50	0.024	-0.004	0.011
10	4	1	0.5	0.2	0.5	0.15	0.15	0.15	0.00	0.50	0.00	0.028	-0.009	0.005
10	4	1	0.5	0.2	0.5	0.15	0.15	0.15	0.00	0.50	0.25	0.027	-0.004	0.006
10	4	1	0.5	0.2	0.5	0.15	0.15	0.15	0.00	0.50	0.50	0.021	-0.002	0.006
10	4	1	0.5	0.2	0.5	0.15	0.15	0.15	0.25	0.00	0.00	0.012	0.011	0.010
10	4	1	0.5	0.2	0.5	0.15	0.15	0.15	0.25	0.00	0.25	0.014	0.005	0.011
10	4	1	0.5	0.2	0.5	0.15	0.15	0.15	0.25	0.00	0.50	0.017	0.011	0.011
10	4	1	0.5	0.2	0.5	0.15	0.15	0.15	0.25	0.25	0.00	0.025	0.002	0.009
10	4	1	0.5	0.2	0.5	0.15	0.15	0.15	0.25	0.25	0.25	0.021	0.016	0.007
10	4	1	0.5	0.2	0.5	0.15	0.15	0.15	0.25	0.25	0.50	0.015	0.008	0.005
10	4	1	0.5	0.2	0.5	0.15	0.15	0.15	0.25	0.50	0.00	0.012	0.017	0.001
10	4	1	0.5	0.2	0.5	0.15	0.15	0.15	0.25	0.50	0.25	0.029	0.006	0.005
10	4	1	0.5	0.2	0.5	0.15	0.15	0.15	0.25	0.50	0.50	0.023	0.006	0.005
10	4	1	0.5	0.2	0.5	0.15	0.15	0.15	0.50	0.00	0.00	0.012	0.017	0.009
10	4	1	0.5	0.2	0.5	0.15	0.15	0.15	0.50	0.00	0.25	0.015	0.016	0.007
10	4	1	0.5	0.2	0.5	0.15	0.15	0.15	0.50	0.00	0.50	0.012	0.022	0.010

(continued)

												Mean Bias		
N	m_1	$\frac{m_2}{m_1}$	$E(C)$	$E(R)$	$E(U)$	σ_C	σ_R	σ_U	ρ_{CR}	ρ_{CU}	ρ_{RU}	c	r	u
10	4	1	0.5	0.2	0.5	0.15	0.15	0.15	0.50	0.25	0.00	0.023	0.020	0.007
10	4	1	0.5	0.2	0.5	0.15	0.15	0.15	0.50	0.25	0.25	0.019	0.013	0.008
10	4	1	0.5	0.2	0.5	0.15	0.15	0.15	0.50	0.25	0.50	0.021	0.020	0.002
10	4	1	0.5	0.2	0.5	0.15	0.15	0.15	0.50	0.50	0.00	0.023	0.017	0.006
10	4	1	0.5	0.2	0.5	0.15	0.15	0.15	0.50	0.50	0.25	0.023	0.015	0.004
10	4	1	0.5	0.2	0.5	0.15	0.15	0.15	0.50	0.50	0.50	0.027	0.020	0.008
10	4	1	0.5	0.2	0.5	0.30	0.30	0.30	0.00	0.00	0.00	0.073	-0.022	0.034
10	4	1	0.5	0.2	0.5	0.30	0.30	0.30	0.00	0.00	0.25	0.075	-0.024	0.045
10	4	1	0.5	0.2	0.5	0.30	0.30	0.30	0.00	0.00	0.50	0.074	-0.023	0.039
10	4	1	0.5	0.2	0.5	0.30	0.30	0.30	0.00	0.25	0.00	0.099	-0.026	0.033
10	4	1	0.5	0.2	0.5	0.30	0.30	0.30	0.00	0.25	0.25	0.093	-0.031	0.030
10	4	1	0.5	0.2	0.5	0.30	0.30	0.30	0.00	0.25	0.50	0.091	-0.034	0.031
10	4	1	0.5	0.2	0.5	0.30	0.30	0.30	0.00	0.50	0.00	0.117	-0.036	0.027
10	4	1	0.5	0.2	0.5	0.30	0.30	0.30	0.00	0.50	0.25	0.125	-0.034	0.021
10	4	1	0.5	0.2	0.5	0.30	0.30	0.30	0.00	0.50	0.50	0.121	-0.040	0.014
10	4	1	0.5	0.2	0.5	0.30	0.30	0.30	0.25	0.00	0.00	0.071	0.008	0.034
10	4	1	0.5	0.2	0.5	0.30	0.30	0.30	0.25	0.00	0.25	0.079	0.004	0.035
10	4	1	0.5	0.2	0.5	0.30	0.30	0.30	0.25	0.00	0.50	0.080	0.003	0.034
10	4	1	0.5	0.2	0.5	0.30	0.30	0.30	0.25	0.25	0.00	0.097	-0.002	0.033
10	4	1	0.5	0.2	0.5	0.30	0.30	0.30	0.25	0.25	0.25	0.101	0.000	0.027
10	4	1	0.5	0.2	0.5	0.30	0.30	0.30	0.25	0.25	0.50	0.095	0.000	0.030
10	4	1	0.5	0.2	0.5	0.30	0.30	0.30	0.25	0.50	0.00	0.113	-0.006	0.023
10	4	1	0.5	0.2	0.5	0.30	0.30	0.30	0.25	0.50	0.25	0.115	-0.004	0.024
10	4	1	0.5	0.2	0.5	0.30	0.30	0.30	0.25	0.50	0.50	0.117	-0.009	0.021
10	4	1	0.5	0.2	0.5	0.30	0.30	0.30	0.50	0.00	0.00	0.070	0.042	0.043
10	4	1	0.5	0.2	0.5	0.30	0.30	0.30	0.50	0.00	0.25	0.074	0.036	0.045
10	4	1	0.5	0.2	0.5	0.30	0.30	0.30	0.50	0.00	0.50	0.075	0.036	0.040
10	4	1	0.5	0.2	0.5	0.30	0.30	0.30	0.50	0.25	0.00	0.097	0.025	0.034
10	4	1	0.5	0.2	0.5	0.30	0.30	0.30	0.50	0.25	0.25	0.097	0.027	0.028
10	4	1	0.5	0.2	0.5	0.30	0.30	0.30	0.50	0.25	0.50	0.103	0.028	0.028
10	4	1	0.5	0.2	0.5	0.30	0.30	0.30	0.50	0.50	0.00	0.114	0.030	0.020
10	4	1	0.5	0.2	0.5	0.30	0.30	0.30	0.50	0.50	0.25	0.113	0.024	0.018
10	4	1	0.5	0.2	0.5	0.30	0.30	0.30	0.50	0.50	0.50	0.121	0.019	0.018
10	4	1	0.5	0.5	0.5	0.00	0.00	0.00	0.00	0.00	0.00	-0.008	0.028	-0.001
10	4	1	0.5	0.5	0.5	0.15	0.15	0.15	0.00	0.00	0.00	0.013	-0.001	0.012
10	4	1	0.5	0.5	0.5	0.15	0.15	0.15	0.00	0.00	0.25	0.021	-0.006	0.011
10	4	1	0.5	0.5	0.5	0.15	0.15	0.15	0.00	0.00	0.50	0.012	-0.002	0.009
10	4	1	0.5	0.5	0.5	0.15	0.15	0.15	0.00	0.25	0.00	0.021	-0.002	0.007
10	4	1	0.5	0.5	0.5	0.15	0.15	0.15	0.00	0.25	0.25	0.024	-0.008	0.011

(continued)

												Mean Bias		
N	m_1	$\frac{m_2}{m_1}$	$E(C)$	$E(R)$	$E(U)$	σ_C	σ_R	σ_U	ρ_{CR}	ρ_{CU}	ρ_{RU}	c	r	u
10	4	1	0.5	0.5	0.5	0.15	0.15	0.15	0.00	0.25	0.50	0.013	-0.011	0.007
10	4	1	0.5	0.5	0.5	0.15	0.15	0.15	0.00	0.50	0.00	0.019	0.000	0.005
10	4	1	0.5	0.5	0.5	0.15	0.15	0.15	0.00	0.50	0.25	0.022	-0.011	0.003
10	4	1	0.5	0.5	0.5	0.15	0.15	0.15	0.00	0.50	0.50	0.028	-0.012	0.006
10	4	1	0.5	0.5	0.5	0.15	0.15	0.15	0.25	0.00	0.00	0.016	0.002	0.009
10	4	1	0.5	0.5	0.5	0.15	0.15	0.15	0.25	0.00	0.25	0.009	0.015	0.006
10	4	1	0.5	0.5	0.5	0.15	0.15	0.15	0.25	0.00	0.50	0.005	0.016	0.012
10	4	1	0.5	0.5	0.5	0.15	0.15	0.15	0.25	0.25	0.00	0.021	0.002	0.010
10	4	1	0.5	0.5	0.5	0.15	0.15	0.15	0.25	0.25	0.25	0.013	0.009	0.006
10	4	1	0.5	0.5	0.5	0.15	0.15	0.15	0.25	0.25	0.50	0.025	0.003	0.008
10	4	1	0.5	0.5	0.5	0.15	0.15	0.15	0.25	0.50	0.00	0.020	0.002	0.005
10	4	1	0.5	0.5	0.5	0.15	0.15	0.15	0.25	0.50	0.25	0.024	0.002	0.007
10	4	1	0.5	0.5	0.5	0.15	0.15	0.15	0.25	0.50	0.50	0.015	0.003	0.003
10	4	1	0.5	0.5	0.5	0.15	0.15	0.15	0.50	0.00	0.00	0.017	0.016	0.010
10	4	1	0.5	0.5	0.5	0.15	0.15	0.15	0.50	0.00	0.25	0.016	0.029	0.011
10	4	1	0.5	0.5	0.5	0.15	0.15	0.15	0.50	0.00	0.50	0.004	0.026	0.008
10	4	1	0.5	0.5	0.5	0.15	0.15	0.15	0.50	0.25	0.00	0.024	0.009	0.005
10	4	1	0.5	0.5	0.5	0.15	0.15	0.15	0.50	0.25	0.25	0.020	0.022	0.008
10	4	1	0.5	0.5	0.5	0.15	0.15	0.15	0.50	0.25	0.50	0.024	0.010	0.007
10	4	1	0.5	0.5	0.5	0.15	0.15	0.15	0.50	0.50	0.00	0.023	0.020	0.001
10	4	1	0.5	0.5	0.5	0.15	0.15	0.15	0.50	0.50	0.25	0.019	0.000	0.003
10	4	1	0.5	0.5	0.5	0.15	0.15	0.15	0.50	0.50	0.50	0.017	0.015	0.008
10	4	1	0.5	0.5	0.5	0.30	0.30	0.30	0.00	0.00	0.00	0.069	-0.065	0.044
10	4	1	0.5	0.5	0.5	0.30	0.30	0.30	0.00	0.00	0.25	0.075	-0.049	0.042
10	4	1	0.5	0.5	0.5	0.30	0.30	0.30	0.00	0.00	0.50	0.073	-0.054	0.035
10	4	1	0.5	0.5	0.5	0.30	0.30	0.30	0.00	0.25	0.00	0.096	-0.076	0.031
10	4	1	0.5	0.5	0.5	0.30	0.30	0.30	0.00	0.25	0.25	0.089	-0.074	0.028
10	4	1	0.5	0.5	0.5	0.30	0.30	0.30	0.00	0.25	0.50	0.093	-0.080	0.029
10	4	1	0.5	0.5	0.5	0.30	0.30	0.30	0.00	0.50	0.00	0.122	-0.089	0.029
10	4	1	0.5	0.5	0.5	0.30	0.30	0.30	0.00	0.50	0.25	0.125	-0.093	0.024
10	4	1	0.5	0.5	0.5	0.30	0.30	0.30	0.00	0.50	0.50	0.115	-0.090	0.018
10	4	1	0.5	0.5	0.5	0.30	0.30	0.30	0.25	0.00	0.00	0.077	-0.024	0.043
10	4	1	0.5	0.5	0.5	0.30	0.30	0.30	0.25	0.00	0.25	0.073	-0.026	0.036
10	4	1	0.5	0.5	0.5	0.30	0.30	0.30	0.25	0.00	0.50	0.074	-0.023	0.039
10	4	1	0.5	0.5	0.5	0.30	0.30	0.30	0.25	0.25	0.00	0.098	-0.046	0.030
10	4	1	0.5	0.5	0.5	0.30	0.30	0.30	0.25	0.25	0.25	0.089	-0.037	0.033
10	4	1	0.5	0.5	0.5	0.30	0.30	0.30	0.25	0.25	0.50	0.096	-0.042	0.031
10	4	1	0.5	0.5	0.5	0.30	0.30	0.30	0.25	0.50	0.00	0.118	-0.056	0.020
10	4	1	0.5	0.5	0.5	0.30	0.30	0.30	0.25	0.50	0.25	0.119	-0.056	0.024

(continued)

												Mean Bias		
N	m_1	$\frac{m_2}{m_1}$	$E(C)$	$E(R)$	$E(U)$	σ_C	σ_R	σ_U	ρ_{CR}	ρ_{CU}	ρ_{RU}	c	r	u
10	4	1	0.5	0.5	0.5	0.30	0.30	0.30	0.25	0.50	0.50	0.112	-0.066	0.019
10	4	1	0.5	0.5	0.5	0.30	0.30	0.30	0.50	0.00	0.00	0.073	0.019	0.040
10	4	1	0.5	0.5	0.5	0.30	0.30	0.30	0.50	0.00	0.25	0.075	0.015	0.044
10	4	1	0.5	0.5	0.5	0.30	0.30	0.30	0.50	0.00	0.50	0.073	0.012	0.039
10	4	1	0.5	0.5	0.5	0.30	0.30	0.30	0.50	0.25	0.00	0.099	-0.008	0.028
10	4	1	0.5	0.5	0.5	0.30	0.30	0.30	0.50	0.25	0.25	0.095	-0.003	0.029
10	4	1	0.5	0.5	0.5	0.30	0.30	0.30	0.50	0.25	0.50	0.094	-0.009	0.027
10	4	1	0.5	0.5	0.5	0.30	0.30	0.30	0.50	0.50	0.00	0.115	-0.021	0.022
10	4	1	0.5	0.5	0.5	0.30	0.30	0.30	0.50	0.50	0.25	0.116	-0.026	0.020
10	4	1	0.5	0.5	0.5	0.30	0.30	0.30	0.50	0.50	0.50	0.116	-0.021	0.020
10	4	1	0.5	0.8	0.5	0.00	0.00	0.00	0.00	0.00	0.00	0.000	0.019	-0.002
10	4	1	0.5	0.8	0.5	0.15	0.15	0.15	0.00	0.00	0.00	0.016	-0.014	0.010
10	4	1	0.5	0.8	0.5	0.15	0.15	0.15	0.00	0.00	0.25	0.023	-0.012	0.013
10	4	1	0.5	0.8	0.5	0.15	0.15	0.15	0.00	0.00	0.50	0.020	-0.026	0.012
10	4	1	0.5	0.8	0.5	0.15	0.15	0.15	0.00	0.25	0.00	0.018	-0.011	0.011
10	4	1	0.5	0.8	0.5	0.15	0.15	0.15	0.00	0.25	0.25	0.026	-0.021	0.013
10	4	1	0.5	0.8	0.5	0.15	0.15	0.15	0.00	0.25	0.50	0.023	-0.026	0.006
10	4	1	0.5	0.8	0.5	0.15	0.15	0.15	0.00	0.50	0.00	0.028	-0.029	0.006
10	4	1	0.5	0.8	0.5	0.15	0.15	0.15	0.00	0.50	0.25	0.026	-0.026	0.005
10	4	1	0.5	0.8	0.5	0.15	0.15	0.15	0.00	0.50	0.50	0.024	-0.022	0.005
10	4	1	0.5	0.8	0.5	0.15	0.15	0.15	0.25	0.00	0.00	0.019	-0.008	0.010
10	4	1	0.5	0.8	0.5	0.15	0.15	0.15	0.25	0.00	0.25	0.018	-0.005	0.009
10	4	1	0.5	0.8	0.5	0.15	0.15	0.15	0.25	0.00	0.50	0.018	-0.002	0.010
10	4	1	0.5	0.8	0.5	0.15	0.15	0.15	0.25	0.25	0.00	0.023	-0.017	0.011
10	4	1	0.5	0.8	0.5	0.15	0.15	0.15	0.25	0.25	0.25	0.028	-0.017	0.010
10	4	1	0.5	0.8	0.5	0.15	0.15	0.15	0.25	0.25	0.50	0.023	-0.014	0.013
10	4	1	0.5	0.8	0.5	0.15	0.15	0.15	0.25	0.50	0.00	0.028	-0.025	0.005
10	4	1	0.5	0.8	0.5	0.15	0.15	0.15	0.25	0.50	0.25	0.027	-0.021	0.006
10	4	1	0.5	0.8	0.5	0.15	0.15	0.15	0.25	0.50	0.50	0.026	-0.013	0.005
10	4	1	0.5	0.8	0.5	0.15	0.15	0.15	0.50	0.00	0.00	0.022	0.001	0.014
10	4	1	0.5	0.8	0.5	0.15	0.15	0.15	0.50	0.00	0.25	0.022	0.003	0.013
10	4	1	0.5	0.8	0.5	0.15	0.15	0.15	0.50	0.00	0.50	0.018	-0.004	0.010
10	4	1	0.5	0.8	0.5	0.15	0.15	0.15	0.50	0.25	0.00	0.019	-0.004	0.006
10	4	1	0.5	0.8	0.5	0.15	0.15	0.15	0.50	0.25	0.25	0.021	0.005	0.006
10	4	1	0.5	0.8	0.5	0.15	0.15	0.15	0.50	0.25	0.50	0.023	-0.007	0.009
10	4	1	0.5	0.8	0.5	0.15	0.15	0.15	0.50	0.50	0.00	0.026	-0.021	0.006
10	4	1	0.5	0.8	0.5	0.15	0.15	0.15	0.50	0.50	0.25	0.034	-0.010	0.007
10	4	1	0.5	0.8	0.5	0.15	0.15	0.15	0.50	0.50	0.50	0.032	-0.016	0.004
10	4	1	0.5	0.8	0.5	0.30	0.30	0.30	0.00	0.00	0.00	0.072	-0.101	0.045

(continued)

N	m_1	$\frac{m_2}{m_1}$	$E(C)$	$E(R)$	$E(U)$	σ_C	σ_R	σ_U	ρ_{CR}	ρ_{CU}	ρ_{RU}	Mean Bias c	Mean Bias r	Mean Bias u
10	4	1	0.5	0.8	0.5	0.30	0.30	0.30	0.00	0.00	0.25	0.070	-0.105	0.040
10	4	1	0.5	0.8	0.5	0.30	0.30	0.30	0.00	0.00	0.50	0.074	-0.101	0.035
10	4	1	0.5	0.8	0.5	0.30	0.30	0.30	0.00	0.25	0.00	0.101	-0.118	0.033
10	4	1	0.5	0.8	0.5	0.30	0.30	0.30	0.00	0.25	0.25	0.098	-0.119	0.034
10	4	1	0.5	0.8	0.5	0.30	0.30	0.30	0.00	0.25	0.50	0.097	-0.119	0.036
10	4	1	0.5	0.8	0.5	0.30	0.30	0.30	0.00	0.50	0.00	0.114	-0.142	0.015
10	4	1	0.5	0.8	0.5	0.30	0.30	0.30	0.00	0.50	0.25	0.123	-0.142	0.023
10	4	1	0.5	0.8	0.5	0.30	0.30	0.30	0.00	0.50	0.50	0.123	-0.150	0.022
10	4	1	0.5	0.8	0.5	0.30	0.30	0.30	0.25	0.00	0.00	0.074	-0.067	0.041
10	4	1	0.5	0.8	0.5	0.30	0.30	0.30	0.25	0.00	0.25	0.076	-0.062	0.041
10	4	1	0.5	0.8	0.5	0.30	0.30	0.30	0.25	0.00	0.50	0.079	-0.070	0.039
10	4	1	0.5	0.8	0.5	0.30	0.30	0.30	0.25	0.25	0.00	0.097	-0.101	0.034
10	4	1	0.5	0.8	0.5	0.30	0.30	0.30	0.25	0.25	0.25	0.093	-0.098	0.028
10	4	1	0.5	0.8	0.5	0.30	0.30	0.30	0.25	0.25	0.50	0.093	-0.098	0.033
10	4	1	0.5	0.8	0.5	0.30	0.30	0.30	0.25	0.50	0.00	0.123	-0.124	0.019
10	4	1	0.5	0.8	0.5	0.30	0.30	0.30	0.25	0.50	0.25	0.119	-0.117	0.022
10	4	1	0.5	0.8	0.5	0.30	0.30	0.30	0.25	0.50	0.50	0.119	-0.123	0.027
10	4	1	0.5	0.8	0.5	0.30	0.30	0.30	0.50	0.00	0.00	0.080	-0.042	0.054
10	4	1	0.5	0.8	0.5	0.30	0.30	0.30	0.50	0.00	0.25	0.077	-0.039	0.042
10	4	1	0.5	0.8	0.5	0.30	0.30	0.30	0.50	0.00	0.50	0.076	-0.034	0.039
10	4	1	0.5	0.8	0.5	0.30	0.30	0.30	0.50	0.25	0.00	0.095	-0.067	0.036
10	4	1	0.5	0.8	0.5	0.30	0.30	0.30	0.50	0.25	0.25	0.096	-0.064	0.033
10	4	1	0.5	0.8	0.5	0.30	0.30	0.30	0.50	0.25	0.50	0.098	-0.067	0.023
10	4	1	0.5	0.8	0.5	0.30	0.30	0.30	0.50	0.50	0.00	0.117	-0.088	0.024
10	4	1	0.5	0.8	0.5	0.30	0.30	0.30	0.50	0.50	0.25	0.117	-0.079	0.021
10	4	1	0.5	0.8	0.5	0.30	0.30	0.30	0.50	0.50	0.50	0.115	-0.093	0.019
10	4	1	0.8	0.2	0.5	0.00	0.00	0.00	0.00	0.00	0.00	0.000	0.004	0.001
10	4	1	0.8	0.2	0.5	0.15	0.15	0.15	0.00	0.00	0.00	0.000	-0.001	0.003
10	4	1	0.8	0.2	0.5	0.15	0.15	0.15	0.00	0.00	0.25	0.001	-0.002	0.003
10	4	1	0.8	0.2	0.5	0.15	0.15	0.15	0.00	0.00	0.50	0.009	0.002	0.010
10	4	1	0.8	0.2	0.5	0.15	0.15	0.15	0.00	0.25	0.00	0.007	0.000	0.003
10	4	1	0.8	0.2	0.5	0.15	0.15	0.15	0.00	0.25	0.25	0.009	-0.009	0.002
10	4	1	0.8	0.2	0.5	0.15	0.15	0.15	0.00	0.25	0.50	0.012	0.002	0.005
10	4	1	0.8	0.2	0.5	0.15	0.15	0.15	0.00	0.50	0.00	0.016	-0.008	0.001
10	4	1	0.8	0.2	0.5	0.15	0.15	0.15	0.00	0.50	0.25	0.016	-0.004	0.001
10	4	1	0.8	0.2	0.5	0.15	0.15	0.15	0.00	0.50	0.50	0.016	-0.003	-0.003
10	4	1	0.8	0.2	0.5	0.15	0.15	0.15	0.25	0.00	0.00	0.002	0.004	0.004
10	4	1	0.8	0.2	0.5	0.15	0.15	0.15	0.25	0.00	0.25	0.004	0.008	0.006
10	4	1	0.8	0.2	0.5	0.15	0.15	0.15	0.25	0.00	0.50	-0.001	0.007	0.005

(continued)

												Mean Bias		
N	m_1	$\frac{m_2}{m_1}$	$E(C)$	$E(R)$	$E(U)$	σ_C	σ_R	σ_U	ρ_{CR}	ρ_{CU}	ρ_{RU}	c	r	u
10	4	1	0.8	0.2	0.5	0.15	0.15	0.15	0.25	0.25	0.00	0.007	0.007	0.004
10	4	1	0.8	0.2	0.5	0.15	0.15	0.15	0.25	0.25	0.25	0.011	0.005	0.003
10	4	1	0.8	0.2	0.5	0.15	0.15	0.15	0.25	0.25	0.50	0.014	0.005	0.003
10	4	1	0.8	0.2	0.5	0.15	0.15	0.15	0.25	0.50	0.00	0.014	0.005	0.000
10	4	1	0.8	0.2	0.5	0.15	0.15	0.15	0.25	0.50	0.25	0.018	0.001	-0.002
10	4	1	0.8	0.2	0.5	0.15	0.15	0.15	0.25	0.50	0.50	0.022	0.002	0.000
10	4	1	0.8	0.2	0.5	0.15	0.15	0.15	0.50	0.00	0.00	0.006	0.014	0.005
10	4	1	0.8	0.2	0.5	0.15	0.15	0.15	0.50	0.00	0.25	0.000	0.015	-0.002
10	4	1	0.8	0.2	0.5	0.15	0.15	0.15	0.50	0.00	0.50	0.003	0.006	0.003
10	4	1	0.8	0.2	0.5	0.15	0.15	0.15	0.50	0.25	0.00	0.008	0.010	0.004
10	4	1	0.8	0.2	0.5	0.15	0.15	0.15	0.50	0.25	0.25	0.008	0.010	0.002
10	4	1	0.8	0.2	0.5	0.15	0.15	0.15	0.50	0.25	0.50	0.009	0.012	0.005
10	4	1	0.8	0.2	0.5	0.15	0.15	0.15	0.50	0.50	0.00	0.019	0.004	0.001
10	4	1	0.8	0.2	0.5	0.15	0.15	0.15	0.50	0.50	0.25	0.015	0.008	-0.003
10	4	1	0.8	0.2	0.5	0.15	0.15	0.15	0.50	0.50	0.50	0.015	0.011	0.000
10	4	1	0.8	0.2	0.5	0.30	0.30	0.30	0.00	0.00	0.00	0.033	-0.007	0.018
10	4	1	0.8	0.2	0.5	0.30	0.30	0.30	0.00	0.00	0.25	0.025	-0.006	0.018
10	4	1	0.8	0.2	0.5	0.30	0.30	0.30	0.00	0.00	0.50	0.021	-0.004	0.017
10	4	1	0.8	0.2	0.5	0.30	0.30	0.30	0.00	0.25	0.00	0.049	-0.011	0.014
10	4	1	0.8	0.2	0.5	0.30	0.30	0.30	0.00	0.25	0.25	0.052	-0.009	0.016
10	4	1	0.8	0.2	0.5	0.30	0.30	0.30	0.00	0.25	0.50	0.046	-0.015	0.006
10	4	1	0.8	0.2	0.5	0.30	0.30	0.30	0.00	0.50	0.00	0.075	-0.014	0.006
10	4	1	0.8	0.2	0.5	0.30	0.30	0.30	0.00	0.50	0.25	0.070	-0.012	0.004
10	4	1	0.8	0.2	0.5	0.30	0.30	0.30	0.00	0.50	0.50	0.067	-0.011	0.001
10	4	1	0.8	0.2	0.5	0.30	0.30	0.30	0.25	0.00	0.00	0.026	0.007	0.024
10	4	1	0.8	0.2	0.5	0.30	0.30	0.30	0.25	0.00	0.25	0.028	0.008	0.016
10	4	1	0.8	0.2	0.5	0.30	0.30	0.30	0.25	0.00	0.50	0.028	0.008	0.021
10	4	1	0.8	0.2	0.5	0.30	0.30	0.30	0.25	0.25	0.00	0.050	0.008	0.006
10	4	1	0.8	0.2	0.5	0.30	0.30	0.30	0.25	0.25	0.25	0.049	0.000	0.006
10	4	1	0.8	0.2	0.5	0.30	0.30	0.30	0.25	0.25	0.50	0.049	0.005	0.013
10	4	1	0.8	0.2	0.5	0.30	0.30	0.30	0.25	0.50	0.00	0.072	0.000	0.005
10	4	1	0.8	0.2	0.5	0.30	0.30	0.30	0.25	0.50	0.25	0.078	-0.001	0.006
10	4	1	0.8	0.2	0.5	0.30	0.30	0.30	0.25	0.50	0.50	0.068	-0.001	0.001
10	4	1	0.8	0.2	0.5	0.30	0.30	0.30	0.50	0.00	0.00	0.031	0.032	0.017
10	4	1	0.8	0.2	0.5	0.30	0.30	0.30	0.50	0.00	0.25	0.025	0.021	0.016
10	4	1	0.8	0.2	0.5	0.30	0.30	0.30	0.50	0.00	0.50	0.018	0.023	0.020
10	4	1	0.8	0.2	0.5	0.30	0.30	0.30	0.50	0.25	0.00	0.046	0.018	0.012
10	4	1	0.8	0.2	0.5	0.30	0.30	0.30	0.50	0.25	0.25	0.043	0.016	0.014
10	4	1	0.8	0.2	0.5	0.30	0.30	0.30	0.50	0.25	0.50	0.045	0.013	0.008

(continued)

												Mean Bias		
N	m_1	$\frac{m_2}{m_1}$	$E(C)$	$E(R)$	$E(U)$	σ_C	σ_R	σ_U	ρ_{CR}	ρ_{CU}	ρ_{RU}	c	r	u
10	4	1	0.8	0.2	0.5	0.30	0.30	0.30	0.50	0.50	0.00	0.068	0.017	-0.001
10	4	1	0.8	0.2	0.5	0.30	0.30	0.30	0.50	0.50	0.25	0.075	0.010	0.000
10	4	1	0.8	0.2	0.5	0.30	0.30	0.30	0.50	0.50	0.50	0.071	0.010	-0.002
10	4	1	0.8	0.5	0.5	0.00	0.00	0.00	0.00	0.00	0.00	0.003	0.004	0.001
10	4	1	0.8	0.5	0.5	0.15	0.15	0.15	0.00	0.00	0.00	0.005	-0.002	0.006
10	4	1	0.8	0.5	0.5	0.15	0.15	0.15	0.00	0.00	0.25	0.008	0.001	0.003
10	4	1	0.8	0.5	0.5	0.15	0.15	0.15	0.00	0.00	0.50	0.006	-0.001	0.007
10	4	1	0.8	0.5	0.5	0.15	0.15	0.15	0.00	0.25	0.00	0.006	-0.004	-0.003
10	4	1	0.8	0.5	0.5	0.15	0.15	0.15	0.00	0.25	0.25	0.008	0.000	-0.002
10	4	1	0.8	0.5	0.5	0.15	0.15	0.15	0.00	0.25	0.50	0.010	-0.006	0.002
10	4	1	0.8	0.5	0.5	0.15	0.15	0.15	0.00	0.50	0.00	0.023	-0.002	0.005
10	4	1	0.8	0.5	0.5	0.15	0.15	0.15	0.00	0.50	0.25	0.016	-0.005	0.002
10	4	1	0.8	0.5	0.5	0.15	0.15	0.15	0.00	0.50	0.50	0.012	-0.005	-0.006
10	4	1	0.8	0.5	0.5	0.15	0.15	0.15	0.25	0.00	0.00	0.007	0.003	0.007
10	4	1	0.8	0.5	0.5	0.15	0.15	0.15	0.25	0.00	0.25	0.004	0.006	0.003
10	4	1	0.8	0.5	0.5	0.15	0.15	0.15	0.25	0.00	0.50	0.001	0.006	0.000
10	4	1	0.8	0.5	0.5	0.15	0.15	0.15	0.25	0.25	0.00	0.006	0.004	-0.002
10	4	1	0.8	0.5	0.5	0.15	0.15	0.15	0.25	0.25	0.25	0.007	0.003	-0.006
10	4	1	0.8	0.5	0.5	0.15	0.15	0.15	0.25	0.25	0.50	0.013	-0.004	0.006
10	4	1	0.8	0.5	0.5	0.15	0.15	0.15	0.25	0.50	0.00	0.015	0.002	0.000
10	4	1	0.8	0.5	0.5	0.15	0.15	0.15	0.25	0.50	0.25	0.019	0.000	0.005
10	4	1	0.8	0.5	0.5	0.15	0.15	0.15	0.25	0.50	0.50	0.016	0.003	-0.002
10	4	1	0.8	0.5	0.5	0.15	0.15	0.15	0.50	0.00	0.00	0.001	0.015	0.004
10	4	1	0.8	0.5	0.5	0.15	0.15	0.15	0.50	0.00	0.25	0.006	0.017	0.006
10	4	1	0.8	0.5	0.5	0.15	0.15	0.15	0.50	0.00	0.50	0.006	0.013	0.002
10	4	1	0.8	0.5	0.5	0.15	0.15	0.15	0.50	0.25	0.00	0.015	0.007	0.000
10	4	1	0.8	0.5	0.5	0.15	0.15	0.15	0.50	0.25	0.25	0.009	0.007	0.005
10	4	1	0.8	0.5	0.5	0.15	0.15	0.15	0.50	0.25	0.50	0.010	0.010	0.005
10	4	1	0.8	0.5	0.5	0.15	0.15	0.15	0.50	0.50	0.00	0.022	0.004	-0.002
10	4	1	0.8	0.5	0.5	0.15	0.15	0.15	0.50	0.50	0.25	0.021	0.003	0.000
10	4	1	0.8	0.5	0.5	0.15	0.15	0.15	0.50	0.50	0.50	0.019	0.009	0.000
10	4	1	0.8	0.5	0.5	0.30	0.30	0.30	0.00	0.00	0.00	0.028	-0.016	0.020
10	4	1	0.8	0.5	0.5	0.30	0.30	0.30	0.00	0.00	0.25	0.027	-0.017	0.011
10	4	1	0.8	0.5	0.5	0.30	0.30	0.30	0.00	0.00	0.50	0.027	-0.008	0.018
10	4	1	0.8	0.5	0.5	0.30	0.30	0.30	0.00	0.25	0.00	0.046	-0.028	0.011
10	4	1	0.8	0.5	0.5	0.30	0.30	0.30	0.00	0.25	0.25	0.048	-0.027	0.008
10	4	1	0.8	0.5	0.5	0.30	0.30	0.30	0.00	0.25	0.50	0.042	-0.026	0.012
10	4	1	0.8	0.5	0.5	0.30	0.30	0.30	0.00	0.50	0.00	0.072	-0.037	0.004
10	4	1	0.8	0.5	0.5	0.30	0.30	0.30	0.00	0.50	0.25	0.071	-0.039	0.002

(continued)

N	m_1	$\frac{m_2}{m_1}$	$E(C)$	$E(R)$	$E(U)$	σ_C	σ_R	σ_U	ρ_{CR}	ρ_{CU}	ρ_{RU}	Mean Bias c	Mean Bias r	Mean Bias u
10	4	1	0.8	0.5	0.5	0.30	0.30	0.30	0.00	0.50	0.50	0.074	-0.030	0.009
10	4	1	0.8	0.5	0.5	0.30	0.30	0.30	0.25	0.00	0.00	0.031	-0.002	0.020
10	4	1	0.8	0.5	0.5	0.30	0.30	0.30	0.25	0.00	0.25	0.026	0.003	0.018
10	4	1	0.8	0.5	0.5	0.30	0.30	0.30	0.25	0.00	0.50	0.025	0.010	0.018
10	4	1	0.8	0.5	0.5	0.30	0.30	0.30	0.25	0.25	0.00	0.047	-0.003	0.008
10	4	1	0.8	0.5	0.5	0.30	0.30	0.30	0.25	0.25	0.25	0.049	-0.004	0.012
10	4	1	0.8	0.5	0.5	0.30	0.30	0.30	0.25	0.25	0.50	0.046	-0.002	0.009
10	4	1	0.8	0.5	0.5	0.30	0.30	0.30	0.25	0.50	0.00	0.075	-0.015	0.004
10	4	1	0.8	0.5	0.5	0.30	0.30	0.30	0.25	0.50	0.25	0.068	-0.016	0.001
10	4	1	0.8	0.5	0.5	0.30	0.30	0.30	0.25	0.50	0.50	0.076	-0.017	0.007
10	4	1	0.8	0.5	0.5	0.30	0.30	0.30	0.50	0.00	0.00	0.029	0.027	0.017
10	4	1	0.8	0.5	0.5	0.30	0.30	0.30	0.50	0.00	0.25	0.021	0.032	0.019
10	4	1	0.8	0.5	0.5	0.30	0.30	0.30	0.50	0.00	0.50	0.026	0.025	0.020
10	4	1	0.8	0.5	0.5	0.30	0.30	0.30	0.50	0.25	0.00	0.046	0.015	0.013
10	4	1	0.8	0.5	0.5	0.30	0.30	0.30	0.50	0.25	0.25	0.048	0.017	0.007
10	4	1	0.8	0.5	0.5	0.30	0.30	0.30	0.50	0.25	0.50	0.044	0.015	0.013
10	4	1	0.8	0.5	0.5	0.30	0.30	0.30	0.50	0.50	0.00	0.068	0.001	0.007
10	4	1	0.8	0.5	0.5	0.30	0.30	0.30	0.50	0.50	0.25	0.071	0.003	0.003
10	4	1	0.8	0.5	0.5	0.30	0.30	0.30	0.50	0.50	0.50	0.069	-0.005	0.001
10	4	1	0.8	0.8	0.5	0.00	0.00	0.00	0.00	0.00	0.00	-0.001	0.003	0.002
10	4	1	0.8	0.8	0.5	0.15	0.15	0.15	0.00	0.00	0.00	0.004	-0.005	0.004
10	4	1	0.8	0.8	0.5	0.15	0.15	0.15	0.00	0.00	0.25	0.006	0.002	0.006
10	4	1	0.8	0.8	0.5	0.15	0.15	0.15	0.00	0.00	0.50	0.003	0.007	0.005
10	4	1	0.8	0.8	0.5	0.15	0.15	0.15	0.00	0.25	0.00	0.005	-0.009	0.003
10	4	1	0.8	0.8	0.5	0.15	0.15	0.15	0.00	0.25	0.25	0.006	-0.005	0.002
10	4	1	0.8	0.8	0.5	0.15	0.15	0.15	0.00	0.25	0.50	0.011	-0.012	0.000
10	4	1	0.8	0.8	0.5	0.15	0.15	0.15	0.00	0.50	0.00	0.018	-0.017	0.003
10	4	1	0.8	0.8	0.5	0.15	0.15	0.15	0.00	0.50	0.25	0.022	-0.013	0.003
10	4	1	0.8	0.8	0.5	0.15	0.15	0.15	0.00	0.50	0.50	0.016	-0.010	0.003
10	4	1	0.8	0.8	0.5	0.15	0.15	0.15	0.25	0.00	0.00	0.003	0.004	0.003
10	4	1	0.8	0.8	0.5	0.15	0.15	0.15	0.25	0.00	0.25	0.006	0.003	0.004
10	4	1	0.8	0.8	0.5	0.15	0.15	0.15	0.25	0.00	0.50	0.007	0.007	0.006
10	4	1	0.8	0.8	0.5	0.15	0.15	0.15	0.25	0.25	0.00	0.013	-0.004	0.006
10	4	1	0.8	0.8	0.5	0.15	0.15	0.15	0.25	0.25	0.25	0.008	0.005	0.001
10	4	1	0.8	0.8	0.5	0.15	0.15	0.15	0.25	0.25	0.50	0.006	-0.004	0.002
10	4	1	0.8	0.8	0.5	0.15	0.15	0.15	0.25	0.50	0.00	0.020	-0.008	0.003
10	4	1	0.8	0.8	0.5	0.15	0.15	0.15	0.25	0.50	0.25	0.018	-0.002	-0.001
10	4	1	0.8	0.8	0.5	0.15	0.15	0.15	0.25	0.50	0.50	0.016	-0.007	-0.002
10	4	1	0.8	0.8	0.5	0.15	0.15	0.15	0.50	0.00	0.00	0.003	0.010	0.008

(continued)

N	m_1	$\frac{m_2}{m_1}$	$E(C)$	$E(R)$	$E(U)$	σ_C	σ_R	σ_U	ρ_{CR}	ρ_{CU}	ρ_{RU}	Mean Bias c	Mean Bias r	Mean Bias u
10	4	1	0.8	0.8	0.5	0.15	0.15	0.15	0.50	0.00	0.25	0.007	0.012	0.007
10	4	1	0.8	0.8	0.5	0.15	0.15	0.15	0.50	0.00	0.50	0.007	0.012	0.003
10	4	1	0.8	0.8	0.5	0.15	0.15	0.15	0.50	0.25	0.00	0.009	0.005	0.000
10	4	1	0.8	0.8	0.5	0.15	0.15	0.15	0.50	0.25	0.25	0.005	0.001	-0.001
10	4	1	0.8	0.8	0.5	0.15	0.15	0.15	0.50	0.25	0.50	0.010	0.006	0.006
10	4	1	0.8	0.8	0.5	0.15	0.15	0.15	0.50	0.50	0.00	0.021	-0.003	0.003
10	4	1	0.8	0.8	0.5	0.15	0.15	0.15	0.50	0.50	0.25	0.020	-0.004	-0.001
10	4	1	0.8	0.8	0.5	0.15	0.15	0.15	0.50	0.50	0.50	0.016	0.000	-0.002
10	4	1	0.8	0.8	0.5	0.30	0.30	0.30	0.00	0.00	0.00	0.023	-0.027	0.018
10	4	1	0.8	0.8	0.5	0.30	0.30	0.30	0.00	0.00	0.25	0.027	-0.021	0.020
10	4	1	0.8	0.8	0.5	0.30	0.30	0.30	0.00	0.00	0.50	0.031	-0.020	0.023
10	4	1	0.8	0.8	0.5	0.30	0.30	0.30	0.00	0.25	0.00	0.044	-0.043	0.009
10	4	1	0.8	0.8	0.5	0.30	0.30	0.30	0.00	0.25	0.25	0.053	-0.047	0.014
10	4	1	0.8	0.8	0.5	0.30	0.30	0.30	0.00	0.25	0.50	0.050	-0.042	0.009
10	4	1	0.8	0.8	0.5	0.30	0.30	0.30	0.00	0.50	0.00	0.075	-0.068	0.002
10	4	1	0.8	0.8	0.5	0.30	0.30	0.30	0.00	0.50	0.25	0.077	-0.057	0.010
10	4	1	0.8	0.8	0.5	0.30	0.30	0.30	0.00	0.50	0.50	0.068	-0.070	0.004
10	4	1	0.8	0.8	0.5	0.30	0.30	0.30	0.25	0.00	0.00	0.023	0.001	0.011
10	4	1	0.8	0.8	0.5	0.30	0.30	0.30	0.25	0.00	0.25	0.024	0.004	0.013
10	4	1	0.8	0.8	0.5	0.30	0.30	0.30	0.25	0.00	0.50	0.027	-0.004	0.017
10	4	1	0.8	0.8	0.5	0.30	0.30	0.30	0.25	0.25	0.00	0.048	-0.026	0.005
10	4	1	0.8	0.8	0.5	0.30	0.30	0.30	0.25	0.25	0.25	0.048	-0.023	0.014
10	4	1	0.8	0.8	0.5	0.30	0.30	0.30	0.25	0.25	0.50	0.048	-0.031	0.011
10	4	1	0.8	0.8	0.5	0.30	0.30	0.30	0.25	0.50	0.00	0.075	-0.046	-0.003
10	4	1	0.8	0.8	0.5	0.30	0.30	0.30	0.25	0.50	0.25	0.066	-0.049	0.001
10	4	1	0.8	0.8	0.5	0.30	0.30	0.30	0.25	0.50	0.50	0.071	-0.046	0.006
10	4	1	0.8	0.8	0.5	0.30	0.30	0.30	0.50	0.00	0.00	0.031	0.020	0.025
10	4	1	0.8	0.8	0.5	0.30	0.30	0.30	0.50	0.00	0.25	0.025	0.017	0.022
10	4	1	0.8	0.8	0.5	0.30	0.30	0.30	0.50	0.00	0.50	0.023	0.021	0.021
10	4	1	0.8	0.8	0.5	0.30	0.30	0.30	0.50	0.25	0.00	0.047	-0.007	0.008
10	4	1	0.8	0.8	0.5	0.30	0.30	0.30	0.50	0.25	0.25	0.048	-0.002	0.007
10	4	1	0.8	0.8	0.5	0.30	0.30	0.30	0.50	0.25	0.50	0.049	0.004	0.012
10	4	1	0.8	0.8	0.5	0.30	0.30	0.30	0.50	0.50	0.00	0.068	-0.016	0.005
10	4	1	0.8	0.8	0.5	0.30	0.30	0.30	0.50	0.50	0.25	0.068	-0.026	0.010
10	4	1	0.8	0.8	0.5	0.30	0.30	0.30	0.50	0.50	0.50	0.074	-0.021	0.006
10	8	0	0.2	0.2	0.5	0.00	0.00	0.00	0.00	0.00	0.00	-0.008	0.125	-0.002
10	8	0	0.2	0.2	0.5	0.15	0.15	0.15	0.00	0.00	0.00	0.041	0.026	0.034
10	8	0	0.2	0.2	0.5	0.15	0.15	0.15	0.00	0.00	0.25	0.047	0.013	0.042
10	8	0	0.2	0.2	0.5	0.15	0.15	0.15	0.00	0.00	0.50	0.040	0.016	0.031

(continued)

N	m_1	$\frac{m_2}{m_1}$	$E(C)$	$E(R)$	$E(U)$	σ_C	σ_R	σ_U	ρ_{CR}	ρ_{CU}	ρ_{RU}	Mean Bias		
												c	r	u
10	8	0	0.2	0.2	0.5	0.15	0.15	0.15	0.00	0.25	0.00	0.053	0.014	0.032
10	8	0	0.2	0.2	0.5	0.15	0.15	0.15	0.00	0.25	0.25	0.048	0.019	0.029
10	8	0	0.2	0.2	0.5	0.15	0.15	0.15	0.00	0.25	0.50	0.045	0.009	0.031
10	8	0	0.2	0.2	0.5	0.15	0.15	0.15	0.00	0.50	0.00	0.047	0.020	0.023
10	8	0	0.2	0.2	0.5	0.15	0.15	0.15	0.00	0.50	0.25	0.044	0.018	0.022
10	8	0	0.2	0.2	0.5	0.15	0.15	0.15	0.00	0.50	0.50	0.048	0.015	0.027
10	8	0	0.2	0.2	0.5	0.15	0.15	0.15	0.25	0.00	0.00	0.047	0.027	0.038
10	8	0	0.2	0.2	0.5	0.15	0.15	0.15	0.25	0.00	0.25	0.043	0.037	0.034
10	8	0	0.2	0.2	0.5	0.15	0.15	0.15	0.25	0.00	0.50	0.051	0.032	0.040
10	8	0	0.2	0.2	0.5	0.15	0.15	0.15	0.25	0.25	0.00	0.049	0.039	0.029
10	8	0	0.2	0.2	0.5	0.15	0.15	0.15	0.25	0.25	0.25	0.045	0.041	0.029
10	8	0	0.2	0.2	0.5	0.15	0.15	0.15	0.25	0.25	0.50	0.048	0.019	0.033
10	8	0	0.2	0.2	0.5	0.15	0.15	0.15	0.25	0.50	0.00	0.044	0.043	0.023
10	8	0	0.2	0.2	0.5	0.15	0.15	0.15	0.25	0.50	0.25	0.047	0.036	0.026
10	8	0	0.2	0.2	0.5	0.15	0.15	0.15	0.25	0.50	0.50	0.047	0.028	0.024
10	8	0	0.2	0.2	0.5	0.15	0.15	0.15	0.50	0.00	0.00	0.046	0.053	0.036
10	8	0	0.2	0.2	0.5	0.15	0.15	0.15	0.50	0.00	0.25	0.050	0.059	0.041
10	8	0	0.2	0.2	0.5	0.15	0.15	0.15	0.50	0.00	0.50	0.049	0.042	0.041
10	8	0	0.2	0.2	0.5	0.15	0.15	0.15	0.50	0.25	0.00	0.054	0.048	0.035
10	8	0	0.2	0.2	0.5	0.15	0.15	0.15	0.50	0.25	0.25	0.052	0.050	0.031
10	8	0	0.2	0.2	0.5	0.15	0.15	0.15	0.50	0.25	0.50	0.055	0.047	0.033
10	8	0	0.2	0.2	0.5	0.15	0.15	0.15	0.50	0.50	0.00	0.048	0.062	0.025
10	8	0	0.2	0.2	0.5	0.15	0.15	0.15	0.50	0.50	0.25	0.053	0.045	0.029
10	8	0	0.2	0.2	0.5	0.15	0.15	0.15	0.50	0.50	0.50	0.052	0.056	0.026
10	8	0	0.2	0.2	0.5	0.30	0.30	0.30	0.00	0.00	0.00	0.198	-0.097	0.161
10	8	0	0.2	0.2	0.5	0.30	0.30	0.30	0.00	0.00	0.25	0.189	-0.095	0.166
10	8	0	0.2	0.2	0.5	0.30	0.30	0.30	0.00	0.00	0.50	0.193	-0.092	0.163
10	8	0	0.2	0.2	0.5	0.30	0.30	0.30	0.00	0.25	0.00	0.209	-0.092	0.149
10	8	0	0.2	0.2	0.5	0.30	0.30	0.30	0.00	0.25	0.25	0.215	-0.099	0.149
10	8	0	0.2	0.2	0.5	0.30	0.30	0.30	0.00	0.25	0.50	0.210	-0.095	0.151
10	8	0	0.2	0.2	0.5	0.30	0.30	0.30	0.00	0.50	0.00	0.211	-0.105	0.126
10	8	0	0.2	0.2	0.5	0.30	0.30	0.30	0.00	0.50	0.25	0.216	-0.100	0.123
10	8	0	0.2	0.2	0.5	0.30	0.30	0.30	0.00	0.50	0.50	0.211	-0.092	0.124
10	8	0	0.2	0.2	0.5	0.30	0.30	0.30	0.25	0.00	0.00	0.192	-0.050	0.166
10	8	0	0.2	0.2	0.5	0.30	0.30	0.30	0.25	0.00	0.25	0.192	-0.057	0.162
10	8	0	0.2	0.2	0.5	0.30	0.30	0.30	0.25	0.00	0.50	0.193	-0.054	0.162
10	8	0	0.2	0.2	0.5	0.30	0.30	0.30	0.25	0.25	0.00	0.214	-0.054	0.148
10	8	0	0.2	0.2	0.5	0.30	0.30	0.30	0.25	0.25	0.25	0.202	-0.062	0.148
10	8	0	0.2	0.2	0.5	0.30	0.30	0.30	0.25	0.25	0.50	0.209	-0.056	0.145

(continued)

												Mean Bias		
N	m_1	$\frac{m_2}{m_1}$	$E(C)$	$E(R)$	$E(U)$	σ_C	σ_R	σ_U	ρ_{CR}	ρ_{CU}	ρ_{RU}	c	r	u
10	8	0	0.2	0.2	0.5	0.30	0.30	0.30	0.25	0.50	0.00	0.210	-0.060	0.123
10	8	0	0.2	0.2	0.5	0.30	0.30	0.30	0.25	0.50	0.25	0.212	-0.057	0.131
10	8	0	0.2	0.2	0.5	0.30	0.30	0.30	0.25	0.50	0.50	0.208	-0.062	0.122
10	8	0	0.2	0.2	0.5	0.30	0.30	0.30	0.50	0.00	0.00	0.198	-0.005	0.162
10	8	0	0.2	0.2	0.5	0.30	0.30	0.30	0.50	0.00	0.25	0.194	-0.003	0.160
10	8	0	0.2	0.2	0.5	0.30	0.30	0.30	0.50	0.00	0.50	0.191	-0.003	0.166
10	8	0	0.2	0.2	0.5	0.30	0.30	0.30	0.50	0.25	0.00	0.216	-0.016	0.146
10	8	0	0.2	0.2	0.5	0.30	0.30	0.30	0.50	0.25	0.25	0.196	-0.012	0.138
10	8	0	0.2	0.2	0.5	0.30	0.30	0.30	0.50	0.25	0.50	0.203	-0.005	0.144
10	8	0	0.2	0.2	0.5	0.30	0.30	0.30	0.50	0.50	0.00	0.210	-0.016	0.128
10	8	0	0.2	0.2	0.5	0.30	0.30	0.30	0.50	0.50	0.25	0.208	-0.011	0.128
10	8	0	0.2	0.2	0.5	0.30	0.30	0.30	0.50	0.50	0.50	0.207	-0.008	0.124
10	8	0	0.2	0.5	0.5	0.00	0.00	0.00	0.00	0.00	0.00	0.002	0.083	0.007
10	8	0	0.2	0.5	0.5	0.15	0.15	0.15	0.00	0.00	0.00	0.056	-0.052	0.042
10	8	0	0.2	0.5	0.5	0.15	0.15	0.15	0.00	0.00	0.25	0.053	-0.045	0.041
10	8	0	0.2	0.5	0.5	0.15	0.15	0.15	0.00	0.00	0.50	0.053	-0.044	0.038
10	8	0	0.2	0.5	0.5	0.15	0.15	0.15	0.00	0.25	0.00	0.054	-0.053	0.038
10	8	0	0.2	0.5	0.5	0.15	0.15	0.15	0.00	0.25	0.25	0.049	-0.036	0.030
10	8	0	0.2	0.5	0.5	0.15	0.15	0.15	0.00	0.25	0.50	0.047	-0.025	0.030
10	8	0	0.2	0.5	0.5	0.15	0.15	0.15	0.00	0.50	0.00	0.058	-0.042	0.029
10	8	0	0.2	0.5	0.5	0.15	0.15	0.15	0.00	0.50	0.25	0.057	-0.056	0.028
10	8	0	0.2	0.5	0.5	0.15	0.15	0.15	0.00	0.50	0.50	0.052	-0.031	0.025
10	8	0	0.2	0.5	0.5	0.15	0.15	0.15	0.25	0.00	0.00	0.046	-0.017	0.041
10	8	0	0.2	0.5	0.5	0.15	0.15	0.15	0.25	0.00	0.25	0.053	-0.029	0.041
10	8	0	0.2	0.5	0.5	0.15	0.15	0.15	0.25	0.00	0.50	0.049	-0.008	0.033
10	8	0	0.2	0.5	0.5	0.15	0.15	0.15	0.25	0.25	0.00	0.053	-0.019	0.037
10	8	0	0.2	0.5	0.5	0.15	0.15	0.15	0.25	0.25	0.25	0.057	-0.021	0.037
10	8	0	0.2	0.5	0.5	0.15	0.15	0.15	0.25	0.25	0.50	0.054	-0.017	0.034
10	8	0	0.2	0.5	0.5	0.15	0.15	0.15	0.25	0.50	0.00	0.044	-0.007	0.023
10	8	0	0.2	0.5	0.5	0.15	0.15	0.15	0.25	0.50	0.25	0.053	-0.019	0.027
10	8	0	0.2	0.5	0.5	0.15	0.15	0.15	0.25	0.50	0.50	0.052	-0.016	0.030
10	8	0	0.2	0.5	0.5	0.15	0.15	0.15	0.50	0.00	0.00	0.056	-0.014	0.042
10	8	0	0.2	0.5	0.5	0.15	0.15	0.15	0.50	0.00	0.25	0.047	-0.009	0.039
10	8	0	0.2	0.5	0.5	0.15	0.15	0.15	0.50	0.00	0.50	0.047	0.009	0.038
10	8	0	0.2	0.5	0.5	0.15	0.15	0.15	0.50	0.25	0.00	0.057	0.001	0.035
10	8	0	0.2	0.5	0.5	0.15	0.15	0.15	0.50	0.25	0.25	0.051	-0.004	0.031
10	8	0	0.2	0.5	0.5	0.15	0.15	0.15	0.50	0.25	0.50	0.049	0.001	0.033
10	8	0	0.2	0.5	0.5	0.15	0.15	0.15	0.50	0.50	0.00	0.056	-0.011	0.029
10	8	0	0.2	0.5	0.5	0.15	0.15	0.15	0.50	0.50	0.25	0.056	-0.026	0.032

(continued)

												Mean Bias		
N	m_1	$\frac{m_2}{m_1}$	$E(C)$	$E(R)$	$E(U)$	σ_C	σ_R	σ_U	ρ_{CR}	ρ_{CU}	ρ_{RU}	c	r	u
10	8	0	0.2	0.5	0.5	0.15	0.15	0.15	0.50	0.50	0.50	0.056	-0.006	0.026
10	8	0	0.2	0.5	0.5	0.30	0.30	0.30	0.00	0.00	0.00	0.194	-0.246	0.166
10	8	0	0.2	0.5	0.5	0.30	0.30	0.30	0.00	0.00	0.25	0.191	-0.238	0.161
10	8	0	0.2	0.5	0.5	0.30	0.30	0.30	0.00	0.00	0.50	0.188	-0.246	0.165
10	8	0	0.2	0.5	0.5	0.30	0.30	0.30	0.00	0.25	0.00	0.203	-0.243	0.150
10	8	0	0.2	0.5	0.5	0.30	0.30	0.30	0.00	0.25	0.25	0.211	-0.249	0.147
10	8	0	0.2	0.5	0.5	0.30	0.30	0.30	0.00	0.25	0.50	0.205	-0.247	0.145
10	8	0	0.2	0.5	0.5	0.30	0.30	0.30	0.00	0.50	0.00	0.210	-0.248	0.123
10	8	0	0.2	0.5	0.5	0.30	0.30	0.30	0.00	0.50	0.25	0.216	-0.247	0.121
10	8	0	0.2	0.5	0.5	0.30	0.30	0.30	0.00	0.50	0.50	0.217	-0.252	0.121
10	8	0	0.2	0.5	0.5	0.30	0.30	0.30	0.25	0.00	0.00	0.198	-0.200	0.163
10	8	0	0.2	0.5	0.5	0.30	0.30	0.30	0.25	0.00	0.25	0.189	-0.196	0.165
10	8	0	0.2	0.5	0.5	0.30	0.30	0.30	0.25	0.00	0.50	0.195	-0.200	0.167
10	8	0	0.2	0.5	0.5	0.30	0.30	0.30	0.25	0.25	0.00	0.209	-0.204	0.148
10	8	0	0.2	0.5	0.5	0.30	0.30	0.30	0.25	0.25	0.25	0.206	-0.191	0.147
10	8	0	0.2	0.5	0.5	0.30	0.30	0.30	0.25	0.25	0.50	0.200	-0.200	0.147
10	8	0	0.2	0.5	0.5	0.30	0.30	0.30	0.25	0.50	0.00	0.213	-0.202	0.120
10	8	0	0.2	0.5	0.5	0.30	0.30	0.30	0.25	0.50	0.25	0.212	-0.208	0.121
10	8	0	0.2	0.5	0.5	0.30	0.30	0.30	0.25	0.50	0.50	0.215	-0.205	0.125
10	8	0	0.2	0.5	0.5	0.30	0.30	0.30	0.50	0.00	0.00	0.192	-0.147	0.158
10	8	0	0.2	0.5	0.5	0.30	0.30	0.30	0.50	0.00	0.25	0.194	-0.143	0.166
10	8	0	0.2	0.5	0.5	0.30	0.30	0.30	0.50	0.00	0.50	0.192	-0.148	0.164
10	8	0	0.2	0.5	0.5	0.30	0.30	0.30	0.50	0.25	0.00	0.203	-0.153	0.144
10	8	0	0.2	0.5	0.5	0.30	0.30	0.30	0.50	0.25	0.25	0.208	-0.164	0.148
10	8	0	0.2	0.5	0.5	0.30	0.30	0.30	0.50	0.25	0.50	0.208	-0.163	0.150
10	8	0	0.2	0.5	0.5	0.30	0.30	0.30	0.50	0.50	0.00	0.218	-0.176	0.127
10	8	0	0.2	0.5	0.5	0.30	0.30	0.30	0.50	0.50	0.25	0.207	-0.158	0.125
10	8	0	0.2	0.5	0.5	0.30	0.30	0.30	0.50	0.50	0.50	0.207	-0.162	0.123
10	8	0	0.2	0.8	0.5	0.00	0.00	0.00	0.00	0.00	0.00	0.016	-0.008	0.016
10	8	0	0.2	0.8	0.5	0.15	0.15	0.15	0.00	0.00	0.00	0.051	-0.121	0.043
10	8	0	0.2	0.8	0.5	0.15	0.15	0.15	0.00	0.00	0.25	0.060	-0.134	0.045
10	8	0	0.2	0.8	0.5	0.15	0.15	0.15	0.00	0.00	0.50	0.060	-0.130	0.044
10	8	0	0.2	0.8	0.5	0.15	0.15	0.15	0.00	0.25	0.00	0.062	-0.135	0.040
10	8	0	0.2	0.8	0.5	0.15	0.15	0.15	0.00	0.25	0.25	0.059	-0.130	0.038
10	8	0	0.2	0.8	0.5	0.15	0.15	0.15	0.00	0.25	0.50	0.064	-0.140	0.041
10	8	0	0.2	0.8	0.5	0.15	0.15	0.15	0.00	0.50	0.00	0.062	-0.140	0.033
10	8	0	0.2	0.8	0.5	0.15	0.15	0.15	0.00	0.50	0.25	0.061	-0.134	0.034
10	8	0	0.2	0.8	0.5	0.15	0.15	0.15	0.00	0.50	0.50	0.061	-0.133	0.034
10	8	0	0.2	0.8	0.5	0.15	0.15	0.15	0.25	0.00	0.00	0.058	-0.116	0.042

(continued)

												Mean Bias		
N	m_1	$\frac{m_2}{m_1}$	$E(C)$	$E(R)$	$E(U)$	σ_C	σ_R	σ_U	ρ_{CR}	ρ_{CU}	ρ_{RU}	c	r	u
10	8	0	0.2	0.8	0.5	0.15	0.15	0.15	0.25	0.00	0.25	0.057	-0.119	0.045
10	8	0	0.2	0.8	0.5	0.15	0.15	0.15	0.25	0.00	0.50	0.055	-0.116	0.043
10	8	0	0.2	0.8	0.5	0.15	0.15	0.15	0.25	0.25	0.00	0.061	-0.121	0.040
10	8	0	0.2	0.8	0.5	0.15	0.15	0.15	0.25	0.25	0.25	0.060	-0.120	0.039
10	8	0	0.2	0.8	0.5	0.15	0.15	0.15	0.25	0.25	0.50	0.058	-0.114	0.037
10	8	0	0.2	0.8	0.5	0.15	0.15	0.15	0.25	0.50	0.00	0.058	-0.123	0.033
10	8	0	0.2	0.8	0.5	0.15	0.15	0.15	0.25	0.50	0.25	0.061	-0.116	0.029
10	8	0	0.2	0.8	0.5	0.15	0.15	0.15	0.25	0.50	0.50	0.063	-0.132	0.034
10	8	0	0.2	0.8	0.5	0.15	0.15	0.15	0.50	0.00	0.00	0.058	-0.106	0.044
10	8	0	0.2	0.8	0.5	0.15	0.15	0.15	0.50	0.00	0.25	0.055	-0.097	0.043
10	8	0	0.2	0.8	0.5	0.15	0.15	0.15	0.50	0.00	0.50	0.053	-0.095	0.041
10	8	0	0.2	0.8	0.5	0.15	0.15	0.15	0.50	0.25	0.00	0.058	-0.104	0.036
10	8	0	0.2	0.8	0.5	0.15	0.15	0.15	0.50	0.25	0.25	0.064	-0.105	0.038
10	8	0	0.2	0.8	0.5	0.15	0.15	0.15	0.50	0.25	0.50	0.062	-0.096	0.041
10	8	0	0.2	0.8	0.5	0.15	0.15	0.15	0.50	0.50	0.00	0.061	-0.117	0.036
10	8	0	0.2	0.8	0.5	0.15	0.15	0.15	0.50	0.50	0.25	0.064	-0.108	0.035
10	8	0	0.2	0.8	0.5	0.15	0.15	0.15	0.50	0.50	0.50	0.064	-0.110	0.034
10	8	0	0.2	0.8	0.5	0.30	0.30	0.30	0.00	0.00	0.00	0.199	-0.380	0.165
10	8	0	0.2	0.8	0.5	0.30	0.30	0.30	0.00	0.00	0.25	0.199	-0.388	0.168
10	8	0	0.2	0.8	0.5	0.30	0.30	0.30	0.00	0.00	0.50	0.199	-0.389	0.162
10	8	0	0.2	0.8	0.5	0.30	0.30	0.30	0.00	0.25	0.00	0.208	-0.397	0.145
10	8	0	0.2	0.8	0.5	0.30	0.30	0.30	0.00	0.25	0.25	0.208	-0.398	0.142
10	8	0	0.2	0.8	0.5	0.30	0.30	0.30	0.00	0.25	0.50	0.208	-0.397	0.148
10	8	0	0.2	0.8	0.5	0.30	0.30	0.30	0.00	0.50	0.00	0.217	-0.405	0.125
10	8	0	0.2	0.8	0.5	0.30	0.30	0.30	0.00	0.50	0.25	0.205	-0.402	0.124
10	8	0	0.2	0.8	0.5	0.30	0.30	0.30	0.00	0.50	0.50	0.212	-0.407	0.123
10	8	0	0.2	0.8	0.5	0.30	0.30	0.30	0.25	0.00	0.00	0.197	-0.368	0.170
10	8	0	0.2	0.8	0.5	0.30	0.30	0.30	0.25	0.00	0.25	0.190	-0.350	0.161
10	8	0	0.2	0.8	0.5	0.30	0.30	0.30	0.25	0.00	0.50	0.197	-0.350	0.162
10	8	0	0.2	0.8	0.5	0.30	0.30	0.30	0.25	0.25	0.00	0.203	-0.361	0.139
10	8	0	0.2	0.8	0.5	0.30	0.30	0.30	0.25	0.25	0.25	0.202	-0.366	0.149
10	8	0	0.2	0.8	0.5	0.30	0.30	0.30	0.25	0.25	0.50	0.203	-0.365	0.143
10	8	0	0.2	0.8	0.5	0.30	0.30	0.30	0.25	0.50	0.00	0.207	-0.364	0.116
10	8	0	0.2	0.8	0.5	0.30	0.30	0.30	0.25	0.50	0.25	0.203	-0.363	0.127
10	8	0	0.2	0.8	0.5	0.30	0.30	0.30	0.25	0.50	0.50	0.219	-0.381	0.124
10	8	0	0.2	0.8	0.5	0.30	0.30	0.30	0.50	0.00	0.00	0.202	-0.329	0.158
10	8	0	0.2	0.8	0.5	0.30	0.30	0.30	0.50	0.00	0.25	0.199	-0.329	0.168
10	8	0	0.2	0.8	0.5	0.30	0.30	0.30	0.50	0.00	0.50	0.198	-0.326	0.163
10	8	0	0.2	0.8	0.5	0.30	0.30	0.30	0.50	0.25	0.00	0.208	-0.342	0.152

(continued)

N	m_1	$\frac{m_2}{m_1}$	$E(C)$	$E(R)$	$E(U)$	σ_C	σ_R	σ_U	ρ_{CR}	ρ_{CU}	ρ_{RU}	Mean Bias c	Mean Bias r	Mean Bias u
10	8	0	0.2	0.8	0.5	0.30	0.30	0.30	0.50	0.25	0.25	0.211	-0.335	0.148
10	8	0	0.2	0.8	0.5	0.30	0.30	0.30	0.50	0.25	0.50	0.202	-0.340	0.147
10	8	0	0.2	0.8	0.5	0.30	0.30	0.30	0.50	0.50	0.00	0.211	-0.347	0.128
10	8	0	0.2	0.8	0.5	0.30	0.30	0.30	0.50	0.50	0.25	0.212	-0.344	0.123
10	8	0	0.2	0.8	0.5	0.30	0.30	0.30	0.50	0.50	0.50	0.216	-0.335	0.124
10	8	0	0.5	0.2	0.5	0.00	0.00	0.00	0.00	0.00	0.00	-0.023	0.029	-0.009
10	8	0	0.5	0.2	0.5	0.15	0.15	0.15	0.00	0.00	0.00	0.030	0.000	0.041
10	8	0	0.5	0.2	0.5	0.15	0.15	0.15	0.00	0.00	0.25	0.016	0.008	0.030
10	8	0	0.5	0.2	0.5	0.15	0.15	0.15	0.00	0.00	0.50	0.023	0.004	0.040
10	8	0	0.5	0.2	0.5	0.15	0.15	0.15	0.00	0.25	0.00	0.018	0.006	0.024
10	8	0	0.5	0.2	0.5	0.15	0.15	0.15	0.00	0.25	0.25	0.020	-0.002	0.023
10	8	0	0.5	0.2	0.5	0.15	0.15	0.15	0.00	0.25	0.50	0.022	0.002	0.023
10	8	0	0.5	0.2	0.5	0.15	0.15	0.15	0.00	0.50	0.00	0.024	0.001	0.017
10	8	0	0.5	0.2	0.5	0.15	0.15	0.15	0.00	0.50	0.25	0.019	0.002	0.013
10	8	0	0.5	0.2	0.5	0.15	0.15	0.15	0.00	0.50	0.50	0.024	0.004	0.011
10	8	0	0.5	0.2	0.5	0.15	0.15	0.15	0.25	0.00	0.00	0.024	0.005	0.039
10	8	0	0.5	0.2	0.5	0.15	0.15	0.15	0.25	0.00	0.25	0.016	0.006	0.031
10	8	0	0.5	0.2	0.5	0.15	0.15	0.15	0.25	0.00	0.50	0.019	0.011	0.038
10	8	0	0.5	0.2	0.5	0.15	0.15	0.15	0.25	0.25	0.00	0.020	0.012	0.025
10	8	0	0.5	0.2	0.5	0.15	0.15	0.15	0.25	0.25	0.25	0.018	0.017	0.023
10	8	0	0.5	0.2	0.5	0.15	0.15	0.15	0.25	0.25	0.50	0.018	0.010	0.024
10	8	0	0.5	0.2	0.5	0.15	0.15	0.15	0.25	0.50	0.00	0.018	0.018	0.011
10	8	0	0.5	0.2	0.5	0.15	0.15	0.15	0.25	0.50	0.25	0.018	0.016	0.010
10	8	0	0.5	0.2	0.5	0.15	0.15	0.15	0.25	0.50	0.50	0.022	0.017	0.013
10	8	0	0.5	0.2	0.5	0.15	0.15	0.15	0.50	0.00	0.00	0.018	0.022	0.034
10	8	0	0.5	0.2	0.5	0.15	0.15	0.15	0.50	0.00	0.25	0.018	0.021	0.031
10	8	0	0.5	0.2	0.5	0.15	0.15	0.15	0.50	0.00	0.50	0.018	0.024	0.038
10	8	0	0.5	0.2	0.5	0.15	0.15	0.15	0.50	0.25	0.00	0.019	0.024	0.022
10	8	0	0.5	0.2	0.5	0.15	0.15	0.15	0.50	0.25	0.25	0.026	0.021	0.030
10	8	0	0.5	0.2	0.5	0.15	0.15	0.15	0.50	0.25	0.50	0.029	0.019	0.031
10	8	0	0.5	0.2	0.5	0.15	0.15	0.15	0.50	0.50	0.00	0.026	0.021	0.021
10	8	0	0.5	0.2	0.5	0.15	0.15	0.15	0.50	0.50	0.25	0.022	0.024	0.016
10	8	0	0.5	0.2	0.5	0.15	0.15	0.15	0.50	0.50	0.50	0.020	0.026	0.017
10	8	0	0.5	0.2	0.5	0.30	0.30	0.30	0.00	0.00	0.00	0.113	-0.041	0.161
10	8	0	0.5	0.2	0.5	0.30	0.30	0.30	0.00	0.00	0.25	0.119	-0.040	0.155
10	8	0	0.5	0.2	0.5	0.30	0.30	0.30	0.00	0.00	0.50	0.122	-0.037	0.156
10	8	0	0.5	0.2	0.5	0.30	0.30	0.30	0.00	0.25	0.00	0.127	-0.030	0.124
10	8	0	0.5	0.2	0.5	0.30	0.30	0.30	0.00	0.25	0.25	0.131	-0.038	0.135
10	8	0	0.5	0.2	0.5	0.30	0.30	0.30	0.00	0.25	0.50	0.124	-0.035	0.128

(continued)

												Mean Bias		
N	m_1	$\frac{m_2}{m_1}$	$E(C)$	$E(R)$	$E(U)$	σ_C	σ_R	σ_U	ρ_{CR}	ρ_{CU}	ρ_{RU}	c	r	u
10	8	0	0.5	0.2	0.5	0.30	0.30	0.30	0.00	0.50	0.00	0.135	-0.039	0.098
10	8	0	0.5	0.2	0.5	0.30	0.30	0.30	0.00	0.50	0.25	0.146	-0.043	0.096
10	8	0	0.5	0.2	0.5	0.30	0.30	0.30	0.00	0.50	0.50	0.142	-0.039	0.096
10	8	0	0.5	0.2	0.5	0.30	0.30	0.30	0.25	0.00	0.00	0.116	-0.002	0.157
10	8	0	0.5	0.2	0.5	0.30	0.30	0.30	0.25	0.00	0.25	0.109	-0.006	0.154
10	8	0	0.5	0.2	0.5	0.30	0.30	0.30	0.25	0.00	0.50	0.115	-0.006	0.163
10	8	0	0.5	0.2	0.5	0.30	0.30	0.30	0.25	0.25	0.00	0.124	-0.015	0.135
10	8	0	0.5	0.2	0.5	0.30	0.30	0.30	0.25	0.25	0.25	0.128	-0.012	0.133
10	8	0	0.5	0.2	0.5	0.30	0.30	0.30	0.25	0.25	0.50	0.134	-0.014	0.142
10	8	0	0.5	0.2	0.5	0.30	0.30	0.30	0.25	0.50	0.00	0.140	-0.012	0.098
10	8	0	0.5	0.2	0.5	0.30	0.30	0.30	0.25	0.50	0.25	0.139	-0.012	0.098
10	8	0	0.5	0.2	0.5	0.30	0.30	0.30	0.25	0.50	0.50	0.143	-0.006	0.105
10	8	0	0.5	0.2	0.5	0.30	0.30	0.30	0.50	0.00	0.00	0.121	0.022	0.161
10	8	0	0.5	0.2	0.5	0.30	0.30	0.30	0.50	0.00	0.25	0.110	0.028	0.159
10	8	0	0.5	0.2	0.5	0.30	0.30	0.30	0.50	0.00	0.50	0.114	0.025	0.160
10	8	0	0.5	0.2	0.5	0.30	0.30	0.30	0.50	0.25	0.00	0.124	0.020	0.128
10	8	0	0.5	0.2	0.5	0.30	0.30	0.30	0.50	0.25	0.25	0.128	0.013	0.127
10	8	0	0.5	0.2	0.5	0.30	0.30	0.30	0.50	0.25	0.50	0.126	0.024	0.126
10	8	0	0.5	0.2	0.5	0.30	0.30	0.30	0.50	0.50	0.00	0.144	0.021	0.102
10	8	0	0.5	0.2	0.5	0.30	0.30	0.30	0.50	0.50	0.25	0.142	0.017	0.103
10	8	0	0.5	0.2	0.5	0.30	0.30	0.30	0.50	0.50	0.50	0.141	0.020	0.095
10	8	0	0.5	0.5	0.5	0.00	0.00	0.00	0.00	0.00	0.00	-0.030	0.052	-0.011
10	8	0	0.5	0.5	0.5	0.15	0.15	0.15	0.00	0.00	0.00	0.019	0.001	0.033
10	8	0	0.5	0.5	0.5	0.15	0.15	0.15	0.00	0.00	0.25	0.018	-0.005	0.036
10	8	0	0.5	0.5	0.5	0.15	0.15	0.15	0.00	0.00	0.50	0.023	-0.010	0.037
10	8	0	0.5	0.5	0.5	0.15	0.15	0.15	0.00	0.25	0.00	0.026	-0.008	0.027
10	8	0	0.5	0.5	0.5	0.15	0.15	0.15	0.00	0.25	0.25	0.023	-0.005	0.025
10	8	0	0.5	0.5	0.5	0.15	0.15	0.15	0.00	0.25	0.50	0.022	-0.009	0.031
10	8	0	0.5	0.5	0.5	0.15	0.15	0.15	0.00	0.50	0.00	0.023	0.005	0.010
10	8	0	0.5	0.5	0.5	0.15	0.15	0.15	0.00	0.50	0.25	0.023	-0.008	0.015
10	8	0	0.5	0.5	0.5	0.15	0.15	0.15	0.00	0.50	0.50	0.024	-0.007	0.020
10	8	0	0.5	0.5	0.5	0.15	0.15	0.15	0.25	0.00	0.00	0.017	0.002	0.038
10	8	0	0.5	0.5	0.5	0.15	0.15	0.15	0.25	0.00	0.25	0.026	0.001	0.035
10	8	0	0.5	0.5	0.5	0.15	0.15	0.15	0.25	0.00	0.50	0.030	0.000	0.043
10	8	0	0.5	0.5	0.5	0.15	0.15	0.15	0.25	0.25	0.00	0.017	0.005	0.023
10	8	0	0.5	0.5	0.5	0.15	0.15	0.15	0.25	0.25	0.25	0.019	0.011	0.024
10	8	0	0.5	0.5	0.5	0.15	0.15	0.15	0.25	0.25	0.50	0.026	0.007	0.032
10	8	0	0.5	0.5	0.5	0.15	0.15	0.15	0.25	0.50	0.00	0.025	0.002	0.015
10	8	0	0.5	0.5	0.5	0.15	0.15	0.15	0.25	0.50	0.25	0.022	0.010	0.012

(continued)

												Mean Bias		
N	m_1	$\frac{m_2}{m_1}$	$E(C)$	$E(R)$	$E(U)$	σ_C	σ_R	σ_U	ρ_{CR}	ρ_{CU}	ρ_{RU}	c	r	u
10	8	0	0.5	0.5	0.5	0.15	0.15	0.15	0.25	0.50	0.50	0.024	0.003	0.017
10	8	0	0.5	0.5	0.5	0.15	0.15	0.15	0.50	0.00	0.00	0.014	0.021	0.033
10	8	0	0.5	0.5	0.5	0.15	0.15	0.15	0.50	0.00	0.25	0.026	0.011	0.042
10	8	0	0.5	0.5	0.5	0.15	0.15	0.15	0.50	0.00	0.50	0.022	0.018	0.036
10	8	0	0.5	0.5	0.5	0.15	0.15	0.15	0.50	0.25	0.00	0.022	0.011	0.030
10	8	0	0.5	0.5	0.5	0.15	0.15	0.15	0.50	0.25	0.25	0.020	0.018	0.023
10	8	0	0.5	0.5	0.5	0.15	0.15	0.15	0.50	0.25	0.50	0.022	0.016	0.022
10	8	0	0.5	0.5	0.5	0.15	0.15	0.15	0.50	0.50	0.00	0.026	0.015	0.018
10	8	0	0.5	0.5	0.5	0.15	0.15	0.15	0.50	0.50	0.25	0.025	0.015	0.017
10	8	0	0.5	0.5	0.5	0.15	0.15	0.15	0.50	0.50	0.50	0.019	0.020	0.012
10	8	0	0.5	0.5	0.5	0.30	0.30	0.30	0.00	0.00	0.00	0.114	-0.093	0.155
10	8	0	0.5	0.5	0.5	0.30	0.30	0.30	0.00	0.00	0.25	0.119	-0.090	0.161
10	8	0	0.5	0.5	0.5	0.30	0.30	0.30	0.00	0.00	0.50	0.122	-0.094	0.159
10	8	0	0.5	0.5	0.5	0.30	0.30	0.30	0.00	0.25	0.00	0.130	-0.099	0.129
10	8	0	0.5	0.5	0.5	0.30	0.30	0.30	0.00	0.25	0.25	0.130	-0.093	0.123
10	8	0	0.5	0.5	0.5	0.30	0.30	0.30	0.00	0.25	0.50	0.128	-0.097	0.124
10	8	0	0.5	0.5	0.5	0.30	0.30	0.30	0.00	0.50	0.00	0.138	-0.106	0.091
10	8	0	0.5	0.5	0.5	0.30	0.30	0.30	0.00	0.50	0.25	0.137	-0.099	0.087
10	8	0	0.5	0.5	0.5	0.30	0.30	0.30	0.00	0.50	0.50	0.142	-0.104	0.096
10	8	0	0.5	0.5	0.5	0.30	0.30	0.30	0.25	0.00	0.00	0.115	-0.049	0.166
10	8	0	0.5	0.5	0.5	0.30	0.30	0.30	0.25	0.00	0.25	0.110	-0.053	0.162
10	8	0	0.5	0.5	0.5	0.30	0.30	0.30	0.25	0.00	0.50	0.115	-0.052	0.156
10	8	0	0.5	0.5	0.5	0.30	0.30	0.30	0.25	0.25	0.00	0.131	-0.069	0.131
10	8	0	0.5	0.5	0.5	0.30	0.30	0.30	0.25	0.25	0.25	0.127	-0.064	0.128
10	8	0	0.5	0.5	0.5	0.30	0.30	0.30	0.25	0.25	0.50	0.123	-0.072	0.120
10	8	0	0.5	0.5	0.5	0.30	0.30	0.30	0.25	0.50	0.00	0.137	-0.074	0.092
10	8	0	0.5	0.5	0.5	0.30	0.30	0.30	0.25	0.50	0.25	0.137	-0.068	0.097
10	8	0	0.5	0.5	0.5	0.30	0.30	0.30	0.25	0.50	0.50	0.147	-0.067	0.102
10	8	0	0.5	0.5	0.5	0.30	0.30	0.30	0.50	0.00	0.00	0.120	-0.028	0.162
10	8	0	0.5	0.5	0.5	0.30	0.30	0.30	0.50	0.00	0.25	0.119	-0.030	0.163
10	8	0	0.5	0.5	0.5	0.30	0.30	0.30	0.50	0.00	0.50	0.117	-0.024	0.166
10	8	0	0.5	0.5	0.5	0.30	0.30	0.30	0.50	0.25	0.00	0.132	-0.030	0.128
10	8	0	0.5	0.5	0.5	0.30	0.30	0.30	0.50	0.25	0.25	0.120	-0.028	0.120
10	8	0	0.5	0.5	0.5	0.30	0.30	0.30	0.50	0.25	0.50	0.130	-0.036	0.134
10	8	0	0.5	0.5	0.5	0.30	0.30	0.30	0.50	0.50	0.00	0.140	-0.033	0.087
10	8	0	0.5	0.5	0.5	0.30	0.30	0.30	0.50	0.50	0.25	0.143	-0.038	0.094
10	8	0	0.5	0.5	0.5	0.30	0.30	0.30	0.50	0.50	0.50	0.139	-0.040	0.092
10	8	0	0.5	0.8	0.5	0.00	0.00	0.00	0.00	0.00	0.00	-0.003	0.025	0.001
10	8	0	0.5	0.8	0.5	0.15	0.15	0.15	0.00	0.00	0.00	0.030	-0.034	0.044

(continued)

												Mean Bias		
N	m_1	$\frac{m_2}{m_1}$	$E(C)$	$E(R)$	$E(U)$	σ_C	σ_R	σ_U	ρ_{CR}	ρ_{CU}	ρ_{RU}	c	r	u
10	8	0	0.5	0.8	0.5	0.15	0.15	0.15	0.00	0.00	0.25	0.025	-0.024	0.035
10	8	0	0.5	0.8	0.5	0.15	0.15	0.15	0.00	0.00	0.50	0.030	-0.030	0.038
10	8	0	0.5	0.8	0.5	0.15	0.15	0.15	0.00	0.25	0.00	0.027	-0.031	0.025
10	8	0	0.5	0.8	0.5	0.15	0.15	0.15	0.00	0.25	0.25	0.031	-0.030	0.034
10	8	0	0.5	0.8	0.5	0.15	0.15	0.15	0.00	0.25	0.50	0.029	-0.038	0.030
10	8	0	0.5	0.8	0.5	0.15	0.15	0.15	0.00	0.50	0.00	0.032	-0.034	0.025
10	8	0	0.5	0.8	0.5	0.15	0.15	0.15	0.00	0.50	0.25	0.031	-0.032	0.021
10	8	0	0.5	0.8	0.5	0.15	0.15	0.15	0.00	0.50	0.50	0.035	-0.032	0.022
10	8	0	0.5	0.8	0.5	0.15	0.15	0.15	0.25	0.00	0.00	0.030	-0.028	0.042
10	8	0	0.5	0.8	0.5	0.15	0.15	0.15	0.25	0.00	0.25	0.032	-0.026	0.046
10	8	0	0.5	0.8	0.5	0.15	0.15	0.15	0.25	0.00	0.50	0.031	-0.019	0.041
10	8	0	0.5	0.8	0.5	0.15	0.15	0.15	0.25	0.25	0.00	0.027	-0.022	0.028
10	8	0	0.5	0.8	0.5	0.15	0.15	0.15	0.25	0.25	0.25	0.031	-0.022	0.035
10	8	0	0.5	0.8	0.5	0.15	0.15	0.15	0.25	0.25	0.50	0.029	-0.018	0.026
10	8	0	0.5	0.8	0.5	0.15	0.15	0.15	0.25	0.50	0.00	0.030	-0.022	0.020
10	8	0	0.5	0.8	0.5	0.15	0.15	0.15	0.25	0.50	0.25	0.031	-0.022	0.019
10	8	0	0.5	0.8	0.5	0.15	0.15	0.15	0.25	0.50	0.50	0.028	-0.013	0.017
10	8	0	0.5	0.8	0.5	0.15	0.15	0.15	0.50	0.00	0.00	0.023	-0.010	0.035
10	8	0	0.5	0.8	0.5	0.15	0.15	0.15	0.50	0.00	0.25	0.029	-0.011	0.041
10	8	0	0.5	0.8	0.5	0.15	0.15	0.15	0.50	0.00	0.50	0.029	-0.014	0.041
10	8	0	0.5	0.8	0.5	0.15	0.15	0.15	0.50	0.25	0.00	0.031	-0.012	0.030
10	8	0	0.5	0.8	0.5	0.15	0.15	0.15	0.50	0.25	0.25	0.030	-0.013	0.028
10	8	0	0.5	0.8	0.5	0.15	0.15	0.15	0.50	0.25	0.50	0.026	-0.013	0.029
10	8	0	0.5	0.8	0.5	0.15	0.15	0.15	0.50	0.50	0.00	0.029	-0.009	0.016
10	8	0	0.5	0.8	0.5	0.15	0.15	0.15	0.50	0.50	0.25	0.030	-0.016	0.023
10	8	0	0.5	0.8	0.5	0.15	0.15	0.15	0.50	0.50	0.50	0.029	-0.007	0.016
10	8	0	0.5	0.8	0.5	0.30	0.30	0.30	0.00	0.00	0.00	0.120	-0.154	0.157
10	8	0	0.5	0.8	0.5	0.30	0.30	0.30	0.00	0.00	0.25	0.112	-0.149	0.161
10	8	0	0.5	0.8	0.5	0.30	0.30	0.30	0.00	0.00	0.50	0.118	-0.151	0.156
10	8	0	0.5	0.8	0.5	0.30	0.30	0.30	0.00	0.25	0.00	0.132	-0.161	0.136
10	8	0	0.5	0.8	0.5	0.30	0.30	0.30	0.00	0.25	0.25	0.132	-0.158	0.131
10	8	0	0.5	0.8	0.5	0.30	0.30	0.30	0.00	0.25	0.50	0.132	-0.167	0.136
10	8	0	0.5	0.8	0.5	0.30	0.30	0.30	0.00	0.50	0.00	0.148	-0.174	0.091
10	8	0	0.5	0.8	0.5	0.30	0.30	0.30	0.00	0.50	0.25	0.137	-0.166	0.090
10	8	0	0.5	0.8	0.5	0.30	0.30	0.30	0.00	0.50	0.50	0.141	-0.170	0.098
10	8	0	0.5	0.8	0.5	0.30	0.30	0.30	0.25	0.00	0.00	0.115	-0.122	0.165
10	8	0	0.5	0.8	0.5	0.30	0.30	0.30	0.25	0.00	0.25	0.113	-0.128	0.167
10	8	0	0.5	0.8	0.5	0.30	0.30	0.30	0.25	0.00	0.50	0.117	-0.115	0.157
10	8	0	0.5	0.8	0.5	0.30	0.30	0.30	0.25	0.25	0.00	0.133	-0.131	0.133

(continued)

												Mean Bias		
N	m_1	$\frac{m_2}{m_1}$	$E(C)$	$E(R)$	$E(U)$	σ_C	σ_R	σ_U	ρ_{CR}	ρ_{CU}	ρ_{RU}	c	r	u
10	8	0	0.5	0.8	0.5	0.30	0.30	0.30	0.25	0.25	0.25	0.130	-0.135	0.123
10	8	0	0.5	0.8	0.5	0.30	0.30	0.30	0.25	0.25	0.50	0.126	-0.130	0.139
10	8	0	0.5	0.8	0.5	0.30	0.30	0.30	0.25	0.50	0.00	0.138	-0.142	0.088
10	8	0	0.5	0.8	0.5	0.30	0.30	0.30	0.25	0.50	0.25	0.148	-0.152	0.100
10	8	0	0.5	0.8	0.5	0.30	0.30	0.30	0.25	0.50	0.50	0.143	-0.145	0.092
10	8	0	0.5	0.8	0.5	0.30	0.30	0.30	0.50	0.00	0.00	0.120	-0.094	0.160
10	8	0	0.5	0.8	0.5	0.30	0.30	0.30	0.50	0.00	0.25	0.115	-0.091	0.153
10	8	0	0.5	0.8	0.5	0.30	0.30	0.30	0.50	0.00	0.50	0.119	-0.094	0.162
10	8	0	0.5	0.8	0.5	0.30	0.30	0.30	0.50	0.25	0.00	0.132	-0.105	0.132
10	8	0	0.5	0.8	0.5	0.30	0.30	0.30	0.50	0.25	0.25	0.130	-0.106	0.133
10	8	0	0.5	0.8	0.5	0.30	0.30	0.30	0.50	0.25	0.50	0.134	-0.110	0.133
10	8	0	0.5	0.8	0.5	0.30	0.30	0.30	0.50	0.50	0.00	0.140	-0.120	0.088
10	8	0	0.5	0.8	0.5	0.30	0.30	0.30	0.50	0.50	0.25	0.142	-0.118	0.095
10	8	0	0.5	0.8	0.5	0.30	0.30	0.30	0.50	0.50	0.50	0.143	-0.114	0.096
10	8	0	0.8	0.2	0.5	0.00	0.00	0.00	0.00	0.00	0.00	-0.030	0.020	-0.015
10	8	0	0.8	0.2	0.5	0.15	0.15	0.15	0.00	0.00	0.00	-0.022	0.026	0.024
10	8	0	0.8	0.2	0.5	0.15	0.15	0.15	0.00	0.00	0.25	-0.020	0.025	0.029
10	8	0	0.8	0.2	0.5	0.15	0.15	0.15	0.00	0.00	0.50	-0.013	0.017	0.029
10	8	0	0.8	0.2	0.5	0.15	0.15	0.15	0.00	0.25	0.00	-0.025	0.028	-0.006
10	8	0	0.8	0.2	0.5	0.15	0.15	0.15	0.00	0.25	0.25	-0.022	0.032	0.002
10	8	0	0.8	0.2	0.5	0.15	0.15	0.15	0.00	0.25	0.50	-0.026	0.028	0.005
10	8	0	0.8	0.2	0.5	0.15	0.15	0.15	0.00	0.50	0.00	-0.032	0.039	-0.023
10	8	0	0.8	0.2	0.5	0.15	0.15	0.15	0.00	0.50	0.25	-0.026	0.029	-0.020
10	8	0	0.8	0.2	0.5	0.15	0.15	0.15	0.00	0.50	0.50	-0.027	0.031	-0.014
10	8	0	0.8	0.2	0.5	0.15	0.15	0.15	0.25	0.00	0.00	-0.016	0.024	0.029
10	8	0	0.8	0.2	0.5	0.15	0.15	0.15	0.25	0.00	0.25	-0.015	0.030	0.022
10	8	0	0.8	0.2	0.5	0.15	0.15	0.15	0.25	0.00	0.50	-0.019	0.021	0.026
10	8	0	0.8	0.2	0.5	0.15	0.15	0.15	0.25	0.25	0.00	-0.022	0.029	-0.002
10	8	0	0.8	0.2	0.5	0.15	0.15	0.15	0.25	0.25	0.25	-0.035	0.043	-0.007
10	8	0	0.8	0.2	0.5	0.15	0.15	0.15	0.25	0.25	0.50	-0.024	0.033	-0.007
10	8	0	0.8	0.2	0.5	0.15	0.15	0.15	0.25	0.50	0.00	-0.035	0.041	-0.030
10	8	0	0.8	0.2	0.5	0.15	0.15	0.15	0.25	0.50	0.25	-0.029	0.038	-0.027
10	8	0	0.8	0.2	0.5	0.15	0.15	0.15	0.25	0.50	0.50	-0.030	0.032	-0.032
10	8	0	0.8	0.2	0.5	0.15	0.15	0.15	0.50	0.00	0.00	-0.014	0.019	0.036
10	8	0	0.8	0.2	0.5	0.15	0.15	0.15	0.50	0.00	0.25	-0.021	0.032	0.017
10	8	0	0.8	0.2	0.5	0.15	0.15	0.15	0.50	0.00	0.50	-0.028	0.046	0.012
10	8	0	0.8	0.2	0.5	0.15	0.15	0.15	0.50	0.25	0.00	-0.024	0.041	0.004
10	8	0	0.8	0.2	0.5	0.15	0.15	0.15	0.50	0.25	0.25	-0.020	0.035	0.003
10	8	0	0.8	0.2	0.5	0.15	0.15	0.15	0.50	0.25	0.50	-0.033	0.045	-0.007

(continued)

												Mean Bias		
N	m_1	$\frac{m_2}{m_1}$	$E(C)$	$E(R)$	$E(U)$	σ_C	σ_R	σ_U	ρ_{CR}	ρ_{CU}	ρ_{RU}	c	r	u
10	8	0	0.8	0.2	0.5	0.15	0.15	0.15	0.50	0.50	0.00	-0.030	0.045	-0.025
10	8	0	0.8	0.2	0.5	0.15	0.15	0.15	0.50	0.50	0.25	-0.019	0.040	-0.015
10	8	0	0.8	0.2	0.5	0.15	0.15	0.15	0.50	0.50	0.50	-0.029	0.044	-0.019
10	8	0	0.8	0.2	0.5	0.30	0.30	0.30	0.00	0.00	0.00	-0.023	0.052	0.100
10	8	0	0.8	0.2	0.5	0.30	0.30	0.30	0.00	0.00	0.25	-0.003	0.036	0.123
10	8	0	0.8	0.2	0.5	0.30	0.30	0.30	0.00	0.00	0.50	-0.014	0.052	0.110
10	8	0	0.8	0.2	0.5	0.30	0.30	0.30	0.00	0.25	0.00	-0.043	0.080	0.036
10	8	0	0.8	0.2	0.5	0.30	0.30	0.30	0.00	0.25	0.25	-0.022	0.063	0.052
10	8	0	0.8	0.2	0.5	0.30	0.30	0.30	0.00	0.25	0.50	-0.028	0.068	0.050
10	8	0	0.8	0.2	0.5	0.30	0.30	0.30	0.00	0.50	0.00	-0.067	0.125	-0.030
10	8	0	0.8	0.2	0.5	0.30	0.30	0.30	0.00	0.50	0.25	-0.064	0.117	-0.031
10	8	0	0.8	0.2	0.5	0.30	0.30	0.30	0.00	0.50	0.50	-0.059	0.123	-0.022
10	8	0	0.8	0.2	0.5	0.30	0.30	0.30	0.25	0.00	0.00	-0.019	0.068	0.107
10	8	0	0.8	0.2	0.5	0.30	0.30	0.30	0.25	0.00	0.25	-0.015	0.054	0.113
10	8	0	0.8	0.2	0.5	0.30	0.30	0.30	0.25	0.00	0.50	-0.009	0.060	0.118
10	8	0	0.8	0.2	0.5	0.30	0.30	0.30	0.25	0.25	0.00	-0.033	0.086	0.040
10	8	0	0.8	0.2	0.5	0.30	0.30	0.30	0.25	0.25	0.25	-0.028	0.083	0.046
10	8	0	0.8	0.2	0.5	0.30	0.30	0.30	0.25	0.25	0.50	-0.021	0.078	0.059
10	8	0	0.8	0.2	0.5	0.30	0.30	0.30	0.25	0.50	0.00	-0.066	0.143	-0.045
10	8	0	0.8	0.2	0.5	0.30	0.30	0.30	0.25	0.50	0.25	-0.058	0.136	-0.028
10	8	0	0.8	0.2	0.5	0.30	0.30	0.30	0.25	0.50	0.50	-0.065	0.140	-0.047
10	8	0	0.8	0.2	0.5	0.30	0.30	0.30	0.50	0.00	0.00	-0.020	0.079	0.105
10	8	0	0.8	0.2	0.5	0.30	0.30	0.30	0.50	0.00	0.25	-0.008	0.074	0.115
10	8	0	0.8	0.2	0.5	0.30	0.30	0.30	0.50	0.00	0.50	-0.009	0.067	0.121
10	8	0	0.8	0.2	0.5	0.30	0.30	0.30	0.50	0.25	0.00	-0.027	0.101	0.037
10	8	0	0.8	0.2	0.5	0.30	0.30	0.30	0.50	0.25	0.25	-0.027	0.107	0.048
10	8	0	0.8	0.2	0.5	0.30	0.30	0.30	0.50	0.25	0.50	-0.032	0.101	0.038
10	8	0	0.8	0.2	0.5	0.30	0.30	0.30	0.50	0.50	0.00	-0.060	0.147	-0.043
10	8	0	0.8	0.2	0.5	0.30	0.30	0.30	0.50	0.50	0.25	-0.061	0.154	-0.052
10	8	0	0.8	0.2	0.5	0.30	0.30	0.30	0.50	0.50	0.50	-0.062	0.151	-0.039
10	8	0	0.8	0.5	0.5	0.00	0.00	0.00	0.00	0.00	0.00	-0.030	0.031	-0.015
10	8	0	0.8	0.5	0.5	0.15	0.15	0.15	0.00	0.00	0.00	-0.015	0.019	0.025
10	8	0	0.8	0.5	0.5	0.15	0.15	0.15	0.00	0.00	0.25	-0.019	0.018	0.020
10	8	0	0.8	0.5	0.5	0.15	0.15	0.15	0.00	0.00	0.50	-0.014	0.020	0.019
10	8	0	0.8	0.5	0.5	0.15	0.15	0.15	0.00	0.25	0.00	-0.021	0.027	-0.007
10	8	0	0.8	0.5	0.5	0.15	0.15	0.15	0.00	0.25	0.25	-0.017	0.029	-0.011
10	8	0	0.8	0.5	0.5	0.15	0.15	0.15	0.00	0.25	0.50	-0.018	0.027	-0.001
10	8	0	0.8	0.5	0.5	0.15	0.15	0.15	0.00	0.50	0.00	-0.026	0.027	-0.027
10	8	0	0.8	0.5	0.5	0.15	0.15	0.15	0.00	0.50	0.25	-0.022	0.027	-0.021

(continued)

												Mean Bias		
N	m_1	$\frac{m_2}{m_1}$	$E(C)$	$E(R)$	$E(U)$	σ_C	σ_R	σ_U	ρ_{CR}	ρ_{CU}	ρ_{RU}	c	r	u
10	8	0	0.8	0.5	0.5	0.15	0.15	0.15	0.00	0.50	0.50	-0.018	0.022	-0.024
10	8	0	0.8	0.5	0.5	0.15	0.15	0.15	0.25	0.00	0.00	-0.012	0.025	0.026
10	8	0	0.8	0.5	0.5	0.15	0.15	0.15	0.25	0.00	0.25	-0.008	0.019	0.026
10	8	0	0.8	0.5	0.5	0.15	0.15	0.15	0.25	0.00	0.50	-0.012	0.023	0.024
10	8	0	0.8	0.5	0.5	0.15	0.15	0.15	0.25	0.25	0.00	-0.017	0.033	0.000
10	8	0	0.8	0.5	0.5	0.15	0.15	0.15	0.25	0.25	0.25	-0.012	0.023	0.001
10	8	0	0.8	0.5	0.5	0.15	0.15	0.15	0.25	0.25	0.50	-0.018	0.023	-0.001
10	8	0	0.8	0.5	0.5	0.15	0.15	0.15	0.25	0.50	0.00	-0.024	0.042	-0.030
10	8	0	0.8	0.5	0.5	0.15	0.15	0.15	0.25	0.50	0.25	-0.022	0.037	-0.028
10	8	0	0.8	0.5	0.5	0.15	0.15	0.15	0.25	0.50	0.50	-0.020	0.029	-0.029
10	8	0	0.8	0.5	0.5	0.15	0.15	0.15	0.50	0.00	0.00	-0.012	0.027	0.017
10	8	0	0.8	0.5	0.5	0.15	0.15	0.15	0.50	0.00	0.25	-0.014	0.028	0.018
10	8	0	0.8	0.5	0.5	0.15	0.15	0.15	0.50	0.00	0.50	-0.015	0.031	0.025
10	8	0	0.8	0.5	0.5	0.15	0.15	0.15	0.50	0.25	0.00	-0.017	0.037	-0.004
10	8	0	0.8	0.5	0.5	0.15	0.15	0.15	0.50	0.25	0.25	-0.013	0.041	0.001
10	8	0	0.8	0.5	0.5	0.15	0.15	0.15	0.50	0.25	0.50	-0.013	0.036	0.000
10	8	0	0.8	0.5	0.5	0.15	0.15	0.15	0.50	0.50	0.00	-0.021	0.044	-0.016
10	8	0	0.8	0.5	0.5	0.15	0.15	0.15	0.50	0.50	0.25	-0.018	0.041	-0.022
10	8	0	0.8	0.5	0.5	0.15	0.15	0.15	0.50	0.50	0.50	-0.015	0.036	-0.020
10	8	0	0.8	0.5	0.5	0.30	0.30	0.30	0.00	0.00	0.00	0.001	0.018	0.118
10	8	0	0.8	0.5	0.5	0.30	0.30	0.30	0.00	0.00	0.25	0.008	0.018	0.112
10	8	0	0.8	0.5	0.5	0.30	0.30	0.30	0.00	0.00	0.50	-0.009	0.030	0.094
10	8	0	0.8	0.5	0.5	0.30	0.30	0.30	0.00	0.25	0.00	-0.005	0.028	0.053
10	8	0	0.8	0.5	0.5	0.30	0.30	0.30	0.00	0.25	0.25	0.004	0.024	0.056
10	8	0	0.8	0.5	0.5	0.30	0.30	0.30	0.00	0.25	0.50	-0.004	0.033	0.039
10	8	0	0.8	0.5	0.5	0.30	0.30	0.30	0.00	0.50	0.00	-0.032	0.074	-0.059
10	8	0	0.8	0.5	0.5	0.30	0.30	0.30	0.00	0.50	0.25	-0.026	0.062	-0.033
10	8	0	0.8	0.5	0.5	0.30	0.30	0.30	0.00	0.50	0.50	-0.028	0.064	-0.040
10	8	0	0.8	0.5	0.5	0.30	0.30	0.30	0.25	0.00	0.00	0.005	0.028	0.118
10	8	0	0.8	0.5	0.5	0.30	0.30	0.30	0.25	0.00	0.25	0.008	0.034	0.107
10	8	0	0.8	0.5	0.5	0.30	0.30	0.30	0.25	0.00	0.50	0.008	0.038	0.127
10	8	0	0.8	0.5	0.5	0.30	0.30	0.30	0.25	0.25	0.00	0.002	0.049	0.047
10	8	0	0.8	0.5	0.5	0.30	0.30	0.30	0.25	0.25	0.25	0.011	0.034	0.063
10	8	0	0.8	0.5	0.5	0.30	0.30	0.30	0.25	0.25	0.50	0.001	0.045	0.048
10	8	0	0.8	0.5	0.5	0.30	0.30	0.30	0.25	0.50	0.00	-0.021	0.076	-0.047
10	8	0	0.8	0.5	0.5	0.30	0.30	0.30	0.25	0.50	0.25	-0.031	0.081	-0.057
10	8	0	0.8	0.5	0.5	0.30	0.30	0.30	0.25	0.50	0.50	-0.018	0.078	-0.035
10	8	0	0.8	0.5	0.5	0.30	0.30	0.30	0.50	0.00	0.00	0.006	0.056	0.101
10	8	0	0.8	0.5	0.5	0.30	0.30	0.30	0.50	0.00	0.25	0.007	0.061	0.103

(continued)

N	m_1	$\frac{m_2}{m_1}$	$E(C)$	$E(R)$	$E(U)$	σ_C	σ_R	σ_U	ρ_{CR}	ρ_{CU}	ρ_{RU}	Mean Bias c	Mean Bias r	Mean Bias u
10	8	0	0.8	0.5	0.5	0.30	0.30	0.30	0.50	0.00	0.50	0.019	0.042	0.131
10	8	0	0.8	0.5	0.5	0.30	0.30	0.30	0.50	0.25	0.00	0.002	0.062	0.046
10	8	0	0.8	0.5	0.5	0.30	0.30	0.30	0.50	0.25	0.25	-0.002	0.073	0.037
10	8	0	0.8	0.5	0.5	0.30	0.30	0.30	0.50	0.25	0.50	0.003	0.062	0.043
10	8	0	0.8	0.5	0.5	0.30	0.30	0.30	0.50	0.50	0.00	-0.012	0.092	-0.044
10	8	0	0.8	0.5	0.5	0.30	0.30	0.30	0.50	0.50	0.25	-0.011	0.087	-0.038
10	8	0	0.8	0.5	0.5	0.30	0.30	0.30	0.50	0.50	0.50	-0.019	0.096	-0.042
10	8	0	0.8	0.8	0.5	0.00	0.00	0.00	0.00	0.00	0.00	-0.017	0.026	-0.007
10	8	0	0.8	0.8	0.5	0.15	0.15	0.15	0.00	0.00	0.00	-0.005	0.010	0.024
10	8	0	0.8	0.8	0.5	0.15	0.15	0.15	0.00	0.00	0.25	-0.002	0.005	0.033
10	8	0	0.8	0.8	0.5	0.15	0.15	0.15	0.00	0.00	0.50	0.001	0.005	0.039
10	8	0	0.8	0.8	0.5	0.15	0.15	0.15	0.00	0.25	0.00	-0.003	0.014	-0.001
10	8	0	0.8	0.8	0.5	0.15	0.15	0.15	0.00	0.25	0.25	-0.001	0.008	0.011
10	8	0	0.8	0.8	0.5	0.15	0.15	0.15	0.00	0.25	0.50	-0.008	0.006	0.006
10	8	0	0.8	0.8	0.5	0.15	0.15	0.15	0.00	0.50	0.00	-0.006	0.012	-0.018
10	8	0	0.8	0.8	0.5	0.15	0.15	0.15	0.00	0.50	0.25	-0.009	0.007	-0.022
10	8	0	0.8	0.8	0.5	0.15	0.15	0.15	0.00	0.50	0.50	-0.004	0.014	-0.024
10	8	0	0.8	0.8	0.5	0.15	0.15	0.15	0.25	0.00	0.00	-0.001	0.012	0.031
10	8	0	0.8	0.8	0.5	0.15	0.15	0.15	0.25	0.00	0.25	-0.005	0.014	0.022
10	8	0	0.8	0.8	0.5	0.15	0.15	0.15	0.25	0.00	0.50	-0.002	0.019	0.025
10	8	0	0.8	0.8	0.5	0.15	0.15	0.15	0.25	0.25	0.00	-0.008	0.017	-0.002
10	8	0	0.8	0.8	0.5	0.15	0.15	0.15	0.25	0.25	0.25	-0.004	0.016	0.006
10	8	0	0.8	0.8	0.5	0.15	0.15	0.15	0.25	0.25	0.50	-0.005	0.016	0.006
10	8	0	0.8	0.8	0.5	0.15	0.15	0.15	0.25	0.50	0.00	-0.007	0.021	-0.022
10	8	0	0.8	0.8	0.5	0.15	0.15	0.15	0.25	0.50	0.25	-0.004	0.021	-0.019
10	8	0	0.8	0.8	0.5	0.15	0.15	0.15	0.25	0.50	0.50	-0.008	0.023	-0.028
10	8	0	0.8	0.8	0.5	0.15	0.15	0.15	0.50	0.00	0.00	-0.005	0.021	0.024
10	8	0	0.8	0.8	0.5	0.15	0.15	0.15	0.50	0.00	0.25	-0.001	0.020	0.025
10	8	0	0.8	0.8	0.5	0.15	0.15	0.15	0.50	0.00	0.50	-0.009	0.023	0.025
10	8	0	0.8	0.8	0.5	0.15	0.15	0.15	0.50	0.25	0.00	-0.001	0.025	0.007
10	8	0	0.8	0.8	0.5	0.15	0.15	0.15	0.50	0.25	0.25	-0.007	0.021	0.002
10	8	0	0.8	0.8	0.5	0.15	0.15	0.15	0.50	0.25	0.50	-0.001	0.018	0.006
10	8	0	0.8	0.8	0.5	0.15	0.15	0.15	0.50	0.50	0.00	-0.005	0.021	-0.020
10	8	0	0.8	0.8	0.5	0.15	0.15	0.15	0.50	0.50	0.25	-0.001	0.020	-0.010
10	8	0	0.8	0.8	0.5	0.15	0.15	0.15	0.50	0.50	0.50	-0.005	0.019	-0.016
10	8	0	0.8	0.8	0.5	0.30	0.30	0.30	0.00	0.00	0.00	0.022	-0.017	0.108
10	8	0	0.8	0.8	0.5	0.30	0.30	0.30	0.00	0.00	0.25	0.024	-0.019	0.116
10	8	0	0.8	0.8	0.5	0.30	0.30	0.30	0.00	0.00	0.50	0.026	-0.019	0.111
10	8	0	0.8	0.8	0.5	0.30	0.30	0.30	0.00	0.25	0.00	0.025	-0.018	0.049

(continued)

N	m_1	$\frac{m_2}{m_1}$	$E(C)$	$E(R)$	$E(U)$	σ_C	σ_R	σ_U	ρ_{CR}	ρ_{CU}	ρ_{RU}	Mean Bias c	Mean Bias r	Mean Bias u
10	8	0	0.8	0.8	0.5	0.30	0.30	0.30	0.00	0.25	0.25	0.028	-0.017	0.067
10	8	0	0.8	0.8	0.5	0.30	0.30	0.30	0.00	0.25	0.50	0.029	-0.024	0.061
10	8	0	0.8	0.8	0.5	0.30	0.30	0.30	0.00	0.50	0.00	0.023	-0.012	-0.021
10	8	0	0.8	0.8	0.5	0.30	0.30	0.30	0.00	0.50	0.25	0.025	-0.013	-0.036
10	8	0	0.8	0.8	0.5	0.30	0.30	0.30	0.00	0.50	0.50	0.021	-0.012	-0.021
10	8	0	0.8	0.8	0.5	0.30	0.30	0.30	0.25	0.00	0.00	0.030	-0.006	0.130
10	8	0	0.8	0.8	0.5	0.30	0.30	0.30	0.25	0.00	0.25	0.023	-0.001	0.128
10	8	0	0.8	0.8	0.5	0.30	0.30	0.30	0.25	0.00	0.50	0.024	0.000	0.107
10	8	0	0.8	0.8	0.5	0.30	0.30	0.30	0.25	0.25	0.00	0.035	-0.005	0.064
10	8	0	0.8	0.8	0.5	0.30	0.30	0.30	0.25	0.25	0.25	0.030	-0.007	0.034
10	8	0	0.8	0.8	0.5	0.30	0.30	0.30	0.25	0.25	0.50	0.025	0.005	0.042
10	8	0	0.8	0.8	0.5	0.30	0.30	0.30	0.25	0.50	0.00	0.031	0.003	-0.022
10	8	0	0.8	0.8	0.5	0.30	0.30	0.30	0.25	0.50	0.25	0.028	0.009	-0.038
10	8	0	0.8	0.8	0.5	0.30	0.30	0.30	0.25	0.50	0.50	0.021	0.010	-0.038
10	8	0	0.8	0.8	0.5	0.30	0.30	0.30	0.50	0.00	0.00	0.026	0.019	0.120
10	8	0	0.8	0.8	0.5	0.30	0.30	0.30	0.50	0.00	0.25	0.028	0.014	0.129
10	8	0	0.8	0.8	0.5	0.30	0.30	0.30	0.50	0.00	0.50	0.020	0.021	0.101
10	8	0	0.8	0.8	0.5	0.30	0.30	0.30	0.50	0.25	0.00	0.035	0.012	0.054
10	8	0	0.8	0.8	0.5	0.30	0.30	0.30	0.50	0.25	0.25	0.024	0.012	0.058
10	8	0	0.8	0.8	0.5	0.30	0.30	0.30	0.50	0.25	0.50	0.031	0.021	0.039
10	8	0	0.8	0.8	0.5	0.30	0.30	0.30	0.50	0.50	0.00	0.033	0.020	-0.030
10	8	0	0.8	0.8	0.5	0.30	0.30	0.30	0.50	0.50	0.25	0.031	0.019	-0.036
10	8	0	0.8	0.8	0.5	0.30	0.30	0.30	0.50	0.50	0.50	0.026	0.024	-0.024
10	8	1	0.2	0.2	0.5	0.00	0.00	0.00	0.00	0.00	0.00	-0.003	0.065	0.002
10	8	1	0.2	0.2	0.5	0.15	0.15	0.15	0.00	0.00	0.00	0.027	0.016	0.013
10	8	1	0.2	0.2	0.5	0.15	0.15	0.15	0.00	0.00	0.25	0.033	-0.003	0.013
10	8	1	0.2	0.2	0.5	0.15	0.15	0.15	0.00	0.00	0.50	0.033	-0.002	0.016
10	8	1	0.2	0.2	0.5	0.15	0.15	0.15	0.00	0.25	0.00	0.034	0.006	0.008
10	8	1	0.2	0.2	0.5	0.15	0.15	0.15	0.00	0.25	0.25	0.040	0.001	0.017
10	8	1	0.2	0.2	0.5	0.15	0.15	0.15	0.00	0.25	0.50	0.037	-0.006	0.014
10	8	1	0.2	0.2	0.5	0.15	0.15	0.15	0.00	0.50	0.00	0.042	0.005	0.012
10	8	1	0.2	0.2	0.5	0.15	0.15	0.15	0.00	0.50	0.25	0.037	-0.004	0.009
10	8	1	0.2	0.2	0.5	0.15	0.15	0.15	0.00	0.50	0.50	0.045	-0.013	0.011
10	8	1	0.2	0.2	0.5	0.15	0.15	0.15	0.25	0.00	0.00	0.027	0.029	0.014
10	8	1	0.2	0.2	0.5	0.15	0.15	0.15	0.25	0.00	0.25	0.027	0.034	0.012
10	8	1	0.2	0.2	0.5	0.15	0.15	0.15	0.25	0.00	0.50	0.030	0.023	0.013
10	8	1	0.2	0.2	0.5	0.15	0.15	0.15	0.25	0.25	0.00	0.038	0.021	0.011
10	8	1	0.2	0.2	0.5	0.15	0.15	0.15	0.25	0.25	0.25	0.033	0.024	0.011
10	8	1	0.2	0.2	0.5	0.15	0.15	0.15	0.25	0.25	0.50	0.041	0.020	0.009

(continued)

N	m_1	$\frac{m_2}{m_1}$	$E(C)$	$E(R)$	$E(U)$	σ_C	σ_R	σ_U	ρ_{CR}	ρ_{CU}	ρ_{RU}	Mean Bias c	Mean Bias r	Mean Bias u
10	8	1	0.2	0.2	0.5	0.15	0.15	0.15	0.25	0.50	0.00	0.038	0.025	0.011
10	8	1	0.2	0.2	0.5	0.15	0.15	0.15	0.25	0.50	0.25	0.035	0.024	0.010
10	8	1	0.2	0.2	0.5	0.15	0.15	0.15	0.25	0.50	0.50	0.039	0.004	0.010
10	8	1	0.2	0.2	0.5	0.15	0.15	0.15	0.50	0.00	0.00	0.032	0.063	0.012
10	8	1	0.2	0.2	0.5	0.15	0.15	0.15	0.50	0.00	0.25	0.032	0.043	0.012
10	8	1	0.2	0.2	0.5	0.15	0.15	0.15	0.50	0.00	0.50	0.030	0.058	0.015
10	8	1	0.2	0.2	0.5	0.15	0.15	0.15	0.50	0.25	0.00	0.038	0.039	0.014
10	8	1	0.2	0.2	0.5	0.15	0.15	0.15	0.50	0.25	0.25	0.035	0.043	0.011
10	8	1	0.2	0.2	0.5	0.15	0.15	0.15	0.50	0.25	0.50	0.039	0.051	0.012
10	8	1	0.2	0.2	0.5	0.15	0.15	0.15	0.50	0.50	0.00	0.039	0.043	0.008
10	8	1	0.2	0.2	0.5	0.15	0.15	0.15	0.50	0.50	0.25	0.044	0.039	0.011
10	8	1	0.2	0.2	0.5	0.15	0.15	0.15	0.50	0.50	0.50	0.042	0.032	0.009
10	8	1	0.2	0.2	0.5	0.30	0.30	0.30	0.00	0.00	0.00	0.134	-0.076	0.060
10	8	1	0.2	0.2	0.5	0.30	0.30	0.30	0.00	0.00	0.25	0.132	-0.079	0.054
10	8	1	0.2	0.2	0.5	0.30	0.30	0.30	0.00	0.00	0.50	0.137	-0.090	0.053
10	8	1	0.2	0.2	0.5	0.30	0.30	0.30	0.00	0.25	0.00	0.152	-0.083	0.049
10	8	1	0.2	0.2	0.5	0.30	0.30	0.30	0.00	0.25	0.25	0.154	-0.081	0.050
10	8	1	0.2	0.2	0.5	0.30	0.30	0.30	0.00	0.25	0.50	0.154	-0.094	0.049
10	8	1	0.2	0.2	0.5	0.30	0.30	0.30	0.00	0.50	0.00	0.166	-0.085	0.036
10	8	1	0.2	0.2	0.5	0.30	0.30	0.30	0.00	0.50	0.25	0.159	-0.089	0.037
10	8	1	0.2	0.2	0.5	0.30	0.30	0.30	0.00	0.50	0.50	0.156	-0.091	0.051
10	8	1	0.2	0.2	0.5	0.30	0.30	0.30	0.25	0.00	0.00	0.132	-0.035	0.058
10	8	1	0.2	0.2	0.5	0.30	0.30	0.30	0.25	0.00	0.25	0.129	-0.032	0.048
10	8	1	0.2	0.2	0.5	0.30	0.30	0.30	0.25	0.00	0.50	0.130	-0.023	0.059
10	8	1	0.2	0.2	0.5	0.30	0.30	0.30	0.25	0.25	0.00	0.149	-0.035	0.045
10	8	1	0.2	0.2	0.5	0.30	0.30	0.30	0.25	0.25	0.25	0.143	-0.038	0.053
10	8	1	0.2	0.2	0.5	0.30	0.30	0.30	0.25	0.25	0.50	0.147	-0.044	0.054
10	8	1	0.2	0.2	0.5	0.30	0.30	0.30	0.25	0.50	0.00	0.170	-0.043	0.039
10	8	1	0.2	0.2	0.5	0.30	0.30	0.30	0.25	0.50	0.25	0.168	-0.046	0.042
10	8	1	0.2	0.2	0.5	0.30	0.30	0.30	0.25	0.50	0.50	0.171	-0.037	0.040
10	8	1	0.2	0.2	0.5	0.30	0.30	0.30	0.50	0.00	0.00	0.140	0.030	0.059
10	8	1	0.2	0.2	0.5	0.30	0.30	0.30	0.50	0.00	0.25	0.136	0.028	0.056
10	8	1	0.2	0.2	0.5	0.30	0.30	0.30	0.50	0.00	0.50	0.139	0.014	0.059
10	8	1	0.2	0.2	0.5	0.30	0.30	0.30	0.50	0.25	0.00	0.146	0.008	0.055
10	8	1	0.2	0.2	0.5	0.30	0.30	0.30	0.50	0.25	0.25	0.149	0.007	0.049
10	8	1	0.2	0.2	0.5	0.30	0.30	0.30	0.50	0.25	0.50	0.152	0.009	0.055
10	8	1	0.2	0.2	0.5	0.30	0.30	0.30	0.50	0.50	0.00	0.166	0.005	0.042
10	8	1	0.2	0.2	0.5	0.30	0.30	0.30	0.50	0.50	0.25	0.163	0.003	0.042
10	8	1	0.2	0.2	0.5	0.30	0.30	0.30	0.50	0.50	0.50	0.164	0.009	0.042

(continued)

												Mean Bias		
N	m_1	$\frac{m_2}{m_1}$	$E(C)$	$E(R)$	$E(U)$	σ_C	σ_R	σ_U	ρ_{CR}	ρ_{CU}	ρ_{RU}	c	r	u
10	8	1	0.2	0.5	0.5	0.00	0.00	0.00	0.00	0.00	0.00	0.000	0.070	0.002
10	8	1	0.2	0.5	0.5	0.15	0.15	0.15	0.00	0.00	0.00	0.037	-0.036	0.015
10	8	1	0.2	0.5	0.5	0.15	0.15	0.15	0.00	0.00	0.25	0.035	-0.029	0.015
10	8	1	0.2	0.5	0.5	0.15	0.15	0.15	0.00	0.00	0.50	0.036	-0.033	0.016
10	8	1	0.2	0.5	0.5	0.15	0.15	0.15	0.00	0.25	0.00	0.040	-0.041	0.016
10	8	1	0.2	0.5	0.5	0.15	0.15	0.15	0.00	0.25	0.25	0.037	-0.041	0.011
10	8	1	0.2	0.5	0.5	0.15	0.15	0.15	0.00	0.25	0.50	0.039	-0.030	0.009
10	8	1	0.2	0.5	0.5	0.15	0.15	0.15	0.00	0.50	0.00	0.040	-0.039	0.008
10	8	1	0.2	0.5	0.5	0.15	0.15	0.15	0.00	0.50	0.25	0.039	-0.036	0.010
10	8	1	0.2	0.5	0.5	0.15	0.15	0.15	0.00	0.50	0.50	0.045	-0.047	0.010
10	8	1	0.2	0.5	0.5	0.15	0.15	0.15	0.25	0.00	0.00	0.037	-0.010	0.018
10	8	1	0.2	0.5	0.5	0.15	0.15	0.15	0.25	0.00	0.25	0.036	-0.012	0.015
10	8	1	0.2	0.5	0.5	0.15	0.15	0.15	0.25	0.00	0.50	0.029	-0.002	0.014
10	8	1	0.2	0.5	0.5	0.15	0.15	0.15	0.25	0.25	0.00	0.042	-0.017	0.014
10	8	1	0.2	0.5	0.5	0.15	0.15	0.15	0.25	0.25	0.25	0.034	-0.008	0.014
10	8	1	0.2	0.5	0.5	0.15	0.15	0.15	0.25	0.25	0.50	0.031	-0.015	0.011
10	8	1	0.2	0.5	0.5	0.15	0.15	0.15	0.25	0.50	0.00	0.038	-0.026	0.010
10	8	1	0.2	0.5	0.5	0.15	0.15	0.15	0.25	0.50	0.25	0.041	-0.022	0.008
10	8	1	0.2	0.5	0.5	0.15	0.15	0.15	0.25	0.50	0.50	0.040	-0.012	0.009
10	8	1	0.2	0.5	0.5	0.15	0.15	0.15	0.50	0.00	0.00	0.035	0.000	0.015
10	8	1	0.2	0.5	0.5	0.15	0.15	0.15	0.50	0.00	0.25	0.031	0.018	0.012
10	8	1	0.2	0.5	0.5	0.15	0.15	0.15	0.50	0.00	0.50	0.041	0.008	0.015
10	8	1	0.2	0.5	0.5	0.15	0.15	0.15	0.50	0.25	0.00	0.038	0.016	0.010
10	8	1	0.2	0.5	0.5	0.15	0.15	0.15	0.50	0.25	0.25	0.034	0.018	0.011
10	8	1	0.2	0.5	0.5	0.15	0.15	0.15	0.50	0.25	0.50	0.037	0.005	0.013
10	8	1	0.2	0.5	0.5	0.15	0.15	0.15	0.50	0.50	0.00	0.041	0.009	0.004
10	8	1	0.2	0.5	0.5	0.15	0.15	0.15	0.50	0.50	0.25	0.038	-0.001	0.008
10	8	1	0.2	0.5	0.5	0.15	0.15	0.15	0.50	0.50	0.50	0.040	0.002	0.009
10	8	1	0.2	0.5	0.5	0.30	0.30	0.30	0.00	0.00	0.00	0.134	-0.202	0.059
10	8	1	0.2	0.5	0.5	0.30	0.30	0.30	0.00	0.00	0.25	0.139	-0.204	0.054
10	8	1	0.2	0.5	0.5	0.30	0.30	0.30	0.00	0.00	0.50	0.134	-0.200	0.057
10	8	1	0.2	0.5	0.5	0.30	0.30	0.30	0.00	0.25	0.00	0.148	-0.218	0.055
10	8	1	0.2	0.5	0.5	0.30	0.30	0.30	0.00	0.25	0.25	0.155	-0.218	0.047
10	8	1	0.2	0.5	0.5	0.30	0.30	0.30	0.00	0.25	0.50	0.149	-0.211	0.049
10	8	1	0.2	0.5	0.5	0.30	0.30	0.30	0.00	0.50	0.00	0.171	-0.223	0.045
10	8	1	0.2	0.5	0.5	0.30	0.30	0.30	0.00	0.50	0.25	0.169	-0.222	0.046
10	8	1	0.2	0.5	0.5	0.30	0.30	0.30	0.00	0.50	0.50	0.161	-0.233	0.037
10	8	1	0.2	0.5	0.5	0.30	0.30	0.30	0.25	0.00	0.00	0.140	-0.152	0.058
10	8	1	0.2	0.5	0.5	0.30	0.30	0.30	0.25	0.00	0.25	0.132	-0.150	0.065

(continued)

												Mean Bias		
N	m_1	$\frac{m_2}{m_1}$	$E(C)$	$E(R)$	$E(U)$	σ_C	σ_R	σ_U	ρ_{CR}	ρ_{CU}	ρ_{RU}	c	r	u
10	8	1	0.2	0.5	0.5	0.30	0.30	0.30	0.25	0.00	0.50	0.140	-0.157	0.059
10	8	1	0.2	0.5	0.5	0.30	0.30	0.30	0.25	0.25	0.00	0.151	-0.159	0.049
10	8	1	0.2	0.5	0.5	0.30	0.30	0.30	0.25	0.25	0.25	0.151	-0.163	0.048
10	8	1	0.2	0.5	0.5	0.30	0.30	0.30	0.25	0.25	0.50	0.150	-0.173	0.053
10	8	1	0.2	0.5	0.5	0.30	0.30	0.30	0.25	0.50	0.00	0.174	-0.176	0.043
10	8	1	0.2	0.5	0.5	0.30	0.30	0.30	0.25	0.50	0.25	0.164	-0.163	0.047
10	8	1	0.2	0.5	0.5	0.30	0.30	0.30	0.25	0.50	0.50	0.170	-0.178	0.040
10	8	1	0.2	0.5	0.5	0.30	0.30	0.30	0.50	0.00	0.00	0.125	-0.101	0.059
10	8	1	0.2	0.5	0.5	0.30	0.30	0.30	0.50	0.00	0.25	0.135	-0.100	0.059
10	8	1	0.2	0.5	0.5	0.30	0.30	0.30	0.50	0.00	0.50	0.140	-0.093	0.054
10	8	1	0.2	0.5	0.5	0.30	0.30	0.30	0.50	0.25	0.00	0.152	-0.116	0.047
10	8	1	0.2	0.5	0.5	0.30	0.30	0.30	0.50	0.25	0.25	0.154	-0.125	0.046
10	8	1	0.2	0.5	0.5	0.30	0.30	0.30	0.50	0.25	0.50	0.145	-0.124	0.046
10	8	1	0.2	0.5	0.5	0.30	0.30	0.30	0.50	0.50	0.00	0.161	-0.128	0.045
10	8	1	0.2	0.5	0.5	0.30	0.30	0.30	0.50	0.50	0.25	0.163	-0.115	0.043
10	8	1	0.2	0.5	0.5	0.30	0.30	0.30	0.50	0.50	0.50	0.170	-0.129	0.039
10	8	1	0.2	0.8	0.5	0.00	0.00	0.00	0.00	0.00	0.00	0.012	0.000	0.004
10	8	1	0.2	0.8	0.5	0.15	0.15	0.15	0.00	0.00	0.00	0.035	-0.092	0.019
10	8	1	0.2	0.8	0.5	0.15	0.15	0.15	0.00	0.00	0.25	0.039	-0.089	0.018
10	8	1	0.2	0.8	0.5	0.15	0.15	0.15	0.00	0.00	0.50	0.039	-0.102	0.014
10	8	1	0.2	0.8	0.5	0.15	0.15	0.15	0.00	0.25	0.00	0.045	-0.099	0.013
10	8	1	0.2	0.8	0.5	0.15	0.15	0.15	0.00	0.25	0.25	0.050	-0.114	0.014
10	8	1	0.2	0.8	0.5	0.15	0.15	0.15	0.00	0.25	0.50	0.046	-0.109	0.015
10	8	1	0.2	0.8	0.5	0.15	0.15	0.15	0.00	0.50	0.00	0.050	-0.115	0.015
10	8	1	0.2	0.8	0.5	0.15	0.15	0.15	0.00	0.50	0.25	0.042	-0.106	0.015
10	8	1	0.2	0.8	0.5	0.15	0.15	0.15	0.00	0.50	0.50	0.040	-0.109	0.013
10	8	1	0.2	0.8	0.5	0.15	0.15	0.15	0.25	0.00	0.00	0.041	-0.073	0.019
10	8	1	0.2	0.8	0.5	0.15	0.15	0.15	0.25	0.00	0.25	0.044	-0.083	0.015
10	8	1	0.2	0.8	0.5	0.15	0.15	0.15	0.25	0.00	0.50	0.040	-0.086	0.017
10	8	1	0.2	0.8	0.5	0.15	0.15	0.15	0.25	0.25	0.00	0.041	-0.089	0.013
10	8	1	0.2	0.8	0.5	0.15	0.15	0.15	0.25	0.25	0.25	0.043	-0.086	0.016
10	8	1	0.2	0.8	0.5	0.15	0.15	0.15	0.25	0.25	0.50	0.042	-0.093	0.011
10	8	1	0.2	0.8	0.5	0.15	0.15	0.15	0.25	0.50	0.00	0.049	-0.110	0.009
10	8	1	0.2	0.8	0.5	0.15	0.15	0.15	0.25	0.50	0.25	0.053	-0.108	0.013
10	8	1	0.2	0.8	0.5	0.15	0.15	0.15	0.25	0.50	0.50	0.054	-0.106	0.013
10	8	1	0.2	0.8	0.5	0.15	0.15	0.15	0.50	0.00	0.00	0.034	-0.054	0.014
10	8	1	0.2	0.8	0.5	0.15	0.15	0.15	0.50	0.00	0.25	0.038	-0.064	0.018
10	8	1	0.2	0.8	0.5	0.15	0.15	0.15	0.50	0.00	0.50	0.041	-0.066	0.018
10	8	1	0.2	0.8	0.5	0.15	0.15	0.15	0.50	0.25	0.00	0.048	-0.081	0.014

(continued)

												Mean Bias		
N	m_1	$\frac{m_2}{m_1}$	$E(C)$	$E(R)$	$E(U)$	σ_C	σ_R	σ_U	ρ_{CR}	ρ_{CU}	ρ_{RU}	c	r	u
10	8	1	0.2	0.8	0.5	0.15	0.15	0.15	0.50	0.25	0.25	0.043	-0.070	0.016
10	8	1	0.2	0.8	0.5	0.15	0.15	0.15	0.50	0.25	0.50	0.046	-0.074	0.015
10	8	1	0.2	0.8	0.5	0.15	0.15	0.15	0.50	0.50	0.00	0.049	-0.077	0.014
10	8	1	0.2	0.8	0.5	0.15	0.15	0.15	0.50	0.50	0.25	0.050	-0.083	0.011
10	8	1	0.2	0.8	0.5	0.15	0.15	0.15	0.50	0.50	0.50	0.048	-0.081	0.012
10	8	1	0.2	0.8	0.5	0.30	0.30	0.30	0.00	0.00	0.00	0.138	-0.324	0.056
10	8	1	0.2	0.8	0.5	0.30	0.30	0.30	0.00	0.00	0.25	0.140	-0.337	0.054
10	8	1	0.2	0.8	0.5	0.30	0.30	0.30	0.00	0.00	0.50	0.133	-0.336	0.054
10	8	1	0.2	0.8	0.5	0.30	0.30	0.30	0.00	0.25	0.00	0.153	-0.342	0.050
10	8	1	0.2	0.8	0.5	0.30	0.30	0.30	0.00	0.25	0.25	0.154	-0.336	0.049
10	8	1	0.2	0.8	0.5	0.30	0.30	0.30	0.00	0.25	0.50	0.144	-0.354	0.047
10	8	1	0.2	0.8	0.5	0.30	0.30	0.30	0.00	0.50	0.00	0.160	-0.355	0.045
10	8	1	0.2	0.8	0.5	0.30	0.30	0.30	0.00	0.50	0.25	0.168	-0.354	0.040
10	8	1	0.2	0.8	0.5	0.30	0.30	0.30	0.00	0.50	0.50	0.165	-0.366	0.041
10	8	1	0.2	0.8	0.5	0.30	0.30	0.30	0.25	0.00	0.00	0.132	-0.286	0.054
10	8	1	0.2	0.8	0.5	0.30	0.30	0.30	0.25	0.00	0.25	0.127	-0.287	0.058
10	8	1	0.2	0.8	0.5	0.30	0.30	0.30	0.25	0.00	0.50	0.134	-0.291	0.059
10	8	1	0.2	0.8	0.5	0.30	0.30	0.30	0.25	0.25	0.00	0.149	-0.300	0.051
10	8	1	0.2	0.8	0.5	0.30	0.30	0.30	0.25	0.25	0.25	0.154	-0.305	0.049
10	8	1	0.2	0.8	0.5	0.30	0.30	0.30	0.25	0.25	0.50	0.153	-0.294	0.058
10	8	1	0.2	0.8	0.5	0.30	0.30	0.30	0.25	0.50	0.00	0.164	-0.321	0.041
10	8	1	0.2	0.8	0.5	0.30	0.30	0.30	0.25	0.50	0.25	0.162	-0.320	0.043
10	8	1	0.2	0.8	0.5	0.30	0.30	0.30	0.25	0.50	0.50	0.173	-0.319	0.039
10	8	1	0.2	0.8	0.5	0.30	0.30	0.30	0.50	0.00	0.00	0.139	-0.247	0.060
10	8	1	0.2	0.8	0.5	0.30	0.30	0.30	0.50	0.00	0.25	0.135	-0.245	0.057
10	8	1	0.2	0.8	0.5	0.30	0.30	0.30	0.50	0.00	0.50	0.137	-0.240	0.056
10	8	1	0.2	0.8	0.5	0.30	0.30	0.30	0.50	0.25	0.00	0.151	-0.273	0.054
10	8	1	0.2	0.8	0.5	0.30	0.30	0.30	0.50	0.25	0.25	0.153	-0.284	0.052
10	8	1	0.2	0.8	0.5	0.30	0.30	0.30	0.50	0.25	0.50	0.148	-0.270	0.047
10	8	1	0.2	0.8	0.5	0.30	0.30	0.30	0.50	0.50	0.00	0.166	-0.293	0.042
10	8	1	0.2	0.8	0.5	0.30	0.30	0.30	0.50	0.50	0.25	0.166	-0.281	0.040
10	8	1	0.2	0.8	0.5	0.30	0.30	0.30	0.50	0.50	0.50	0.170	-0.300	0.044
10	8	1	0.5	0.2	0.5	0.00	0.00	0.00	0.00	0.00	0.00	-0.001	0.004	0.001
10	8	1	0.5	0.2	0.5	0.15	0.15	0.15	0.00	0.00	0.00	0.013	-0.002	0.009
10	8	1	0.5	0.2	0.5	0.15	0.15	0.15	0.00	0.00	0.25	0.013	-0.003	0.011
10	8	1	0.5	0.2	0.5	0.15	0.15	0.15	0.00	0.00	0.50	0.020	-0.006	0.010
10	8	1	0.5	0.2	0.5	0.15	0.15	0.15	0.00	0.25	0.00	0.028	-0.005	0.009
10	8	1	0.5	0.2	0.5	0.15	0.15	0.15	0.00	0.25	0.25	0.026	-0.010	0.007
10	8	1	0.5	0.2	0.5	0.15	0.15	0.15	0.00	0.25	0.50	0.021	-0.005	0.003

(continued)

N	m_1	$\frac{m_2}{m_1}$	$E(C)$	$E(R)$	$E(U)$	σ_C	σ_R	σ_U	ρ_{CR}	ρ_{CU}	ρ_{RU}	Mean Bias c	r	u
10	8	1	0.5	0.2	0.5	0.15	0.15	0.15	0.00	0.50	0.00	0.031	-0.012	0.004
10	8	1	0.5	0.2	0.5	0.15	0.15	0.15	0.00	0.50	0.25	0.025	-0.005	0.002
10	8	1	0.5	0.2	0.5	0.15	0.15	0.15	0.00	0.50	0.50	0.024	-0.005	0.003
10	8	1	0.5	0.2	0.5	0.15	0.15	0.15	0.25	0.00	0.00	0.012	0.006	0.008
10	8	1	0.5	0.2	0.5	0.15	0.15	0.15	0.25	0.00	0.25	0.018	0.009	0.009
10	8	1	0.5	0.2	0.5	0.15	0.15	0.15	0.25	0.00	0.50	0.011	0.007	0.010
10	8	1	0.5	0.2	0.5	0.15	0.15	0.15	0.25	0.25	0.00	0.021	0.006	0.004
10	8	1	0.5	0.2	0.5	0.15	0.15	0.15	0.25	0.25	0.25	0.019	0.004	0.007
10	8	1	0.5	0.2	0.5	0.15	0.15	0.15	0.25	0.25	0.50	0.021	0.006	0.006
10	8	1	0.5	0.2	0.5	0.15	0.15	0.15	0.25	0.50	0.00	0.023	0.007	0.002
10	8	1	0.5	0.2	0.5	0.15	0.15	0.15	0.25	0.50	0.25	0.026	0.004	0.006
10	8	1	0.5	0.2	0.5	0.15	0.15	0.15	0.25	0.50	0.50	0.029	0.004	0.004
10	8	1	0.5	0.2	0.5	0.15	0.15	0.15	0.50	0.00	0.00	0.016	0.016	0.015
10	8	1	0.5	0.2	0.5	0.15	0.15	0.15	0.50	0.00	0.25	0.013	0.018	0.009
10	8	1	0.5	0.2	0.5	0.15	0.15	0.15	0.50	0.00	0.50	0.016	0.015	0.008
10	8	1	0.5	0.2	0.5	0.15	0.15	0.15	0.50	0.25	0.00	0.025	0.014	0.010
10	8	1	0.5	0.2	0.5	0.15	0.15	0.15	0.50	0.25	0.25	0.026	0.005	0.008
10	8	1	0.5	0.2	0.5	0.15	0.15	0.15	0.50	0.25	0.50	0.022	0.016	0.009
10	8	1	0.5	0.2	0.5	0.15	0.15	0.15	0.50	0.50	0.00	0.030	0.012	0.004
10	8	1	0.5	0.2	0.5	0.15	0.15	0.15	0.50	0.50	0.25	0.028	0.011	0.002
10	8	1	0.5	0.2	0.5	0.15	0.15	0.15	0.50	0.50	0.50	0.025	0.011	0.002
10	8	1	0.5	0.2	0.5	0.30	0.30	0.30	0.00	0.00	0.00	0.081	-0.029	0.042
10	8	1	0.5	0.2	0.5	0.30	0.30	0.30	0.00	0.00	0.25	0.081	-0.023	0.036
10	8	1	0.5	0.2	0.5	0.30	0.30	0.30	0.00	0.00	0.50	0.075	-0.023	0.039
10	8	1	0.5	0.2	0.5	0.30	0.30	0.30	0.00	0.25	0.00	0.103	-0.026	0.035
10	8	1	0.5	0.2	0.5	0.30	0.30	0.30	0.00	0.25	0.25	0.104	-0.034	0.033
10	8	1	0.5	0.2	0.5	0.30	0.30	0.30	0.00	0.25	0.50	0.095	-0.037	0.023
10	8	1	0.5	0.2	0.5	0.30	0.30	0.30	0.00	0.50	0.00	0.121	-0.041	0.024
10	8	1	0.5	0.2	0.5	0.30	0.30	0.30	0.00	0.50	0.25	0.117	-0.038	0.019
10	8	1	0.5	0.2	0.5	0.30	0.30	0.30	0.00	0.50	0.50	0.119	-0.037	0.023
10	8	1	0.5	0.2	0.5	0.30	0.30	0.30	0.25	0.00	0.00	0.077	0.001	0.038
10	8	1	0.5	0.2	0.5	0.30	0.30	0.30	0.25	0.00	0.25	0.075	0.012	0.041
10	8	1	0.5	0.2	0.5	0.30	0.30	0.30	0.25	0.00	0.50	0.078	0.006	0.043
10	8	1	0.5	0.2	0.5	0.30	0.30	0.30	0.25	0.25	0.00	0.095	-0.001	0.033
10	8	1	0.5	0.2	0.5	0.30	0.30	0.30	0.25	0.25	0.25	0.100	-0.001	0.032
10	8	1	0.5	0.2	0.5	0.30	0.30	0.30	0.25	0.25	0.50	0.097	-0.001	0.033
10	8	1	0.5	0.2	0.5	0.30	0.30	0.30	0.25	0.50	0.00	0.122	-0.007	0.022
10	8	1	0.5	0.2	0.5	0.30	0.30	0.30	0.25	0.50	0.25	0.125	-0.010	0.025
10	8	1	0.5	0.2	0.5	0.30	0.30	0.30	0.25	0.50	0.50	0.118	-0.008	0.023

(continued)

N	m_1	$\frac{m_2}{m_1}$	$E(C)$	$E(R)$	$E(U)$	σ_C	σ_R	σ_U	ρ_{CR}	ρ_{CU}	ρ_{RU}	Mean Bias c	Mean Bias r	Mean Bias u
10	8	1	0.5	0.2	0.5	0.30	0.30	0.30	0.50	0.00	0.00	0.072	0.027	0.038
10	8	1	0.5	0.2	0.5	0.30	0.30	0.30	0.50	0.00	0.25	0.075	0.042	0.041
10	8	1	0.5	0.2	0.5	0.30	0.30	0.30	0.50	0.00	0.50	0.083	0.028	0.037
10	8	1	0.5	0.2	0.5	0.30	0.30	0.30	0.50	0.25	0.00	0.100	0.033	0.029
10	8	1	0.5	0.2	0.5	0.30	0.30	0.30	0.50	0.25	0.25	0.094	0.025	0.025
10	8	1	0.5	0.2	0.5	0.30	0.30	0.30	0.50	0.25	0.50	0.096	0.025	0.033
10	8	1	0.5	0.2	0.5	0.30	0.30	0.30	0.50	0.50	0.00	0.121	0.027	0.023
10	8	1	0.5	0.2	0.5	0.30	0.30	0.30	0.50	0.50	0.25	0.122	0.021	0.024
10	8	1	0.5	0.2	0.5	0.30	0.30	0.30	0.50	0.50	0.50	0.123	0.014	0.019
10	8	1	0.5	0.5	0.5	0.00	0.00	0.00	0.00	0.00	0.00	0.000	0.009	-0.002
10	8	1	0.5	0.5	0.5	0.15	0.15	0.15	0.00	0.00	0.00	0.014	-0.006	0.007
10	8	1	0.5	0.5	0.5	0.15	0.15	0.15	0.00	0.00	0.25	0.011	-0.005	0.008
10	8	1	0.5	0.5	0.5	0.15	0.15	0.15	0.00	0.00	0.50	0.017	-0.011	0.013
10	8	1	0.5	0.5	0.5	0.15	0.15	0.15	0.00	0.25	0.00	0.025	-0.016	0.007
10	8	1	0.5	0.5	0.5	0.15	0.15	0.15	0.00	0.25	0.25	0.025	-0.016	0.007
10	8	1	0.5	0.5	0.5	0.15	0.15	0.15	0.00	0.25	0.50	0.027	-0.015	0.008
10	8	1	0.5	0.5	0.5	0.15	0.15	0.15	0.00	0.50	0.00	0.034	-0.020	0.006
10	8	1	0.5	0.5	0.5	0.15	0.15	0.15	0.00	0.50	0.25	0.025	-0.021	0.006
10	8	1	0.5	0.5	0.5	0.15	0.15	0.15	0.00	0.50	0.50	0.026	-0.019	0.002
10	8	1	0.5	0.5	0.5	0.15	0.15	0.15	0.25	0.00	0.00	0.018	-0.001	0.010
10	8	1	0.5	0.5	0.5	0.15	0.15	0.15	0.25	0.00	0.25	0.015	0.002	0.012
10	8	1	0.5	0.5	0.5	0.15	0.15	0.15	0.25	0.00	0.50	0.016	-0.004	0.012
10	8	1	0.5	0.5	0.5	0.15	0.15	0.15	0.25	0.25	0.00	0.024	-0.006	0.008
10	8	1	0.5	0.5	0.5	0.15	0.15	0.15	0.25	0.25	0.25	0.017	-0.005	0.006
10	8	1	0.5	0.5	0.5	0.15	0.15	0.15	0.25	0.25	0.50	0.025	-0.007	0.009
10	8	1	0.5	0.5	0.5	0.15	0.15	0.15	0.25	0.50	0.00	0.023	-0.006	0.005
10	8	1	0.5	0.5	0.5	0.15	0.15	0.15	0.25	0.50	0.25	0.026	-0.007	0.004
10	8	1	0.5	0.5	0.5	0.15	0.15	0.15	0.25	0.50	0.50	0.031	-0.013	0.005
10	8	1	0.5	0.5	0.5	0.15	0.15	0.15	0.50	0.00	0.00	0.016	0.004	0.007
10	8	1	0.5	0.5	0.5	0.15	0.15	0.15	0.50	0.00	0.25	0.019	0.011	0.010
10	8	1	0.5	0.5	0.5	0.15	0.15	0.15	0.50	0.00	0.50	0.022	0.012	0.012
10	8	1	0.5	0.5	0.5	0.15	0.15	0.15	0.50	0.25	0.00	0.020	0.004	0.010
10	8	1	0.5	0.5	0.5	0.15	0.15	0.15	0.50	0.25	0.25	0.016	0.007	0.006
10	8	1	0.5	0.5	0.5	0.15	0.15	0.15	0.50	0.25	0.50	0.021	0.004	0.008
10	8	1	0.5	0.5	0.5	0.15	0.15	0.15	0.50	0.50	0.00	0.024	0.004	0.002
10	8	1	0.5	0.5	0.5	0.15	0.15	0.15	0.50	0.50	0.25	0.031	0.002	0.003
10	8	1	0.5	0.5	0.5	0.15	0.15	0.15	0.50	0.50	0.50	0.025	-0.003	0.004
10	8	1	0.5	0.5	0.5	0.30	0.30	0.30	0.00	0.00	0.00	0.079	-0.070	0.041
10	8	1	0.5	0.5	0.5	0.30	0.30	0.30	0.00	0.00	0.25	0.076	-0.068	0.040

(continued)

N	m_1	$\frac{m_2}{m_1}$	$E(C)$	$E(R)$	$E(U)$	σ_C	σ_R	σ_U	ρ_{CR}	ρ_{CU}	ρ_{RU}	Mean Bias c	Mean Bias r	Mean Bias u
10	8	1	0.5	0.5	0.5	0.30	0.30	0.30	0.00	0.00	0.50	0.070	-0.068	0.044
10	8	1	0.5	0.5	0.5	0.30	0.30	0.30	0.00	0.25	0.00	0.094	-0.085	0.033
10	8	1	0.5	0.5	0.5	0.30	0.30	0.30	0.00	0.25	0.25	0.095	-0.083	0.036
10	8	1	0.5	0.5	0.5	0.30	0.30	0.30	0.00	0.25	0.50	0.099	-0.084	0.028
10	8	1	0.5	0.5	0.5	0.30	0.30	0.30	0.00	0.50	0.00	0.116	-0.090	0.020
10	8	1	0.5	0.5	0.5	0.30	0.30	0.30	0.00	0.50	0.25	0.123	-0.100	0.016
10	8	1	0.5	0.5	0.5	0.30	0.30	0.30	0.00	0.50	0.50	0.119	-0.096	0.024
10	8	1	0.5	0.5	0.5	0.30	0.30	0.30	0.25	0.00	0.00	0.083	-0.021	0.035
10	8	1	0.5	0.5	0.5	0.30	0.30	0.30	0.25	0.00	0.25	0.074	-0.034	0.042
10	8	1	0.5	0.5	0.5	0.30	0.30	0.30	0.25	0.00	0.50	0.077	-0.027	0.045
10	8	1	0.5	0.5	0.5	0.30	0.30	0.30	0.25	0.25	0.00	0.098	-0.050	0.038
10	8	1	0.5	0.5	0.5	0.30	0.30	0.30	0.25	0.25	0.25	0.099	-0.051	0.032
10	8	1	0.5	0.5	0.5	0.30	0.30	0.30	0.25	0.25	0.50	0.095	-0.047	0.031
10	8	1	0.5	0.5	0.5	0.30	0.30	0.30	0.25	0.50	0.00	0.120	-0.066	0.027
10	8	1	0.5	0.5	0.5	0.30	0.30	0.30	0.25	0.50	0.25	0.117	-0.059	0.017
10	8	1	0.5	0.5	0.5	0.30	0.30	0.30	0.25	0.50	0.50	0.123	-0.056	0.019
10	8	1	0.5	0.5	0.5	0.30	0.30	0.30	0.50	0.00	0.00	0.081	0.009	0.031
10	8	1	0.5	0.5	0.5	0.30	0.30	0.30	0.50	0.00	0.25	0.078	0.008	0.041
10	8	1	0.5	0.5	0.5	0.30	0.30	0.30	0.50	0.00	0.50	0.074	0.011	0.042
10	8	1	0.5	0.5	0.5	0.30	0.30	0.30	0.50	0.25	0.00	0.098	-0.011	0.032
10	8	1	0.5	0.5	0.5	0.30	0.30	0.30	0.50	0.25	0.25	0.099	-0.018	0.038
10	8	1	0.5	0.5	0.5	0.30	0.30	0.30	0.50	0.25	0.50	0.101	-0.013	0.028
10	8	1	0.5	0.5	0.5	0.30	0.30	0.30	0.50	0.50	0.00	0.122	-0.022	0.017
10	8	1	0.5	0.5	0.5	0.30	0.30	0.30	0.50	0.50	0.25	0.126	-0.034	0.023
10	8	1	0.5	0.5	0.5	0.30	0.30	0.30	0.50	0.50	0.50	0.119	-0.037	0.016
10	8	1	0.5	0.8	0.5	0.00	0.00	0.00	0.00	0.00	0.00	-0.005	0.014	0.000
10	8	1	0.5	0.8	0.5	0.15	0.15	0.15	0.00	0.00	0.00	0.018	-0.016	0.010
10	8	1	0.5	0.8	0.5	0.15	0.15	0.15	0.00	0.00	0.25	0.013	-0.014	0.010
10	8	1	0.5	0.8	0.5	0.15	0.15	0.15	0.00	0.00	0.50	0.018	-0.026	0.008
10	8	1	0.5	0.8	0.5	0.15	0.15	0.15	0.00	0.25	0.00	0.021	-0.026	0.006
10	8	1	0.5	0.8	0.5	0.15	0.15	0.15	0.00	0.25	0.25	0.021	-0.024	0.009
10	8	1	0.5	0.8	0.5	0.15	0.15	0.15	0.00	0.25	0.50	0.024	-0.029	0.009
10	8	1	0.5	0.8	0.5	0.15	0.15	0.15	0.00	0.50	0.00	0.027	-0.034	0.003
10	8	1	0.5	0.8	0.5	0.15	0.15	0.15	0.00	0.50	0.25	0.030	-0.034	0.009
10	8	1	0.5	0.8	0.5	0.15	0.15	0.15	0.00	0.50	0.50	0.029	-0.031	0.007
10	8	1	0.5	0.8	0.5	0.15	0.15	0.15	0.25	0.00	0.00	0.020	-0.011	0.012
10	8	1	0.5	0.8	0.5	0.15	0.15	0.15	0.25	0.00	0.25	0.016	-0.008	0.008
10	8	1	0.5	0.8	0.5	0.15	0.15	0.15	0.25	0.00	0.50	0.020	-0.011	0.009
10	8	1	0.5	0.8	0.5	0.15	0.15	0.15	0.25	0.25	0.00	0.021	-0.018	0.002

(continued)

N	m_1	$\frac{m_2}{m_1}$	$E(C)$	$E(R)$	$E(U)$	σ_C	σ_R	σ_U	ρ_{CR}	ρ_{CU}	ρ_{RU}	Mean Bias c	Mean Bias r	Mean Bias u
10	8	1	0.5	0.8	0.5	0.15	0.15	0.15	0.25	0.25	0.25	0.022	-0.016	0.008
10	8	1	0.5	0.8	0.5	0.15	0.15	0.15	0.25	0.25	0.50	0.021	-0.018	0.012
10	8	1	0.5	0.8	0.5	0.15	0.15	0.15	0.25	0.50	0.00	0.030	-0.032	0.007
10	8	1	0.5	0.8	0.5	0.15	0.15	0.15	0.25	0.50	0.25	0.026	-0.024	0.003
10	8	1	0.5	0.8	0.5	0.15	0.15	0.15	0.25	0.50	0.50	0.033	-0.025	0.007
10	8	1	0.5	0.8	0.5	0.15	0.15	0.15	0.50	0.00	0.00	0.015	0.000	0.010
10	8	1	0.5	0.8	0.5	0.15	0.15	0.15	0.50	0.00	0.25	0.016	-0.002	0.012
10	8	1	0.5	0.8	0.5	0.15	0.15	0.15	0.50	0.00	0.50	0.021	-0.002	0.009
10	8	1	0.5	0.8	0.5	0.15	0.15	0.15	0.50	0.25	0.00	0.026	-0.009	0.010
10	8	1	0.5	0.8	0.5	0.15	0.15	0.15	0.50	0.25	0.25	0.025	-0.008	0.006
10	8	1	0.5	0.8	0.5	0.15	0.15	0.15	0.50	0.25	0.50	0.023	-0.010	0.010
10	8	1	0.5	0.8	0.5	0.15	0.15	0.15	0.50	0.50	0.00	0.030	-0.016	0.006
10	8	1	0.5	0.8	0.5	0.15	0.15	0.15	0.50	0.50	0.25	0.025	-0.010	0.002
10	8	1	0.5	0.8	0.5	0.15	0.15	0.15	0.50	0.50	0.50	0.026	-0.017	0.004
10	8	1	0.5	0.8	0.5	0.30	0.30	0.30	0.00	0.00	0.00	0.076	-0.104	0.039
10	8	1	0.5	0.8	0.5	0.30	0.30	0.30	0.00	0.00	0.25	0.082	-0.108	0.042
10	8	1	0.5	0.8	0.5	0.30	0.30	0.30	0.00	0.00	0.50	0.078	-0.106	0.043
10	8	1	0.5	0.8	0.5	0.30	0.30	0.30	0.00	0.25	0.00	0.093	-0.128	0.029
10	8	1	0.5	0.8	0.5	0.30	0.30	0.30	0.00	0.25	0.25	0.101	-0.131	0.036
10	8	1	0.5	0.8	0.5	0.30	0.30	0.30	0.00	0.25	0.50	0.102	-0.132	0.028
10	8	1	0.5	0.8	0.5	0.30	0.30	0.30	0.00	0.50	0.00	0.119	-0.155	0.026
10	8	1	0.5	0.8	0.5	0.30	0.30	0.30	0.00	0.50	0.25	0.120	-0.151	0.027
10	8	1	0.5	0.8	0.5	0.30	0.30	0.30	0.00	0.50	0.50	0.119	-0.158	0.022
10	8	1	0.5	0.8	0.5	0.30	0.30	0.30	0.25	0.00	0.00	0.077	-0.074	0.039
10	8	1	0.5	0.8	0.5	0.30	0.30	0.30	0.25	0.00	0.25	0.077	-0.078	0.038
10	8	1	0.5	0.8	0.5	0.30	0.30	0.30	0.25	0.00	0.50	0.076	-0.072	0.043
10	8	1	0.5	0.8	0.5	0.30	0.30	0.30	0.25	0.25	0.00	0.093	-0.107	0.029
10	8	1	0.5	0.8	0.5	0.30	0.30	0.30	0.25	0.25	0.25	0.100	-0.100	0.031
10	8	1	0.5	0.8	0.5	0.30	0.30	0.30	0.25	0.25	0.50	0.101	-0.099	0.026
10	8	1	0.5	0.8	0.5	0.30	0.30	0.30	0.25	0.50	0.00	0.123	-0.130	0.025
10	8	1	0.5	0.8	0.5	0.30	0.30	0.30	0.25	0.50	0.25	0.120	-0.124	0.021
10	8	1	0.5	0.8	0.5	0.30	0.30	0.30	0.25	0.50	0.50	0.122	-0.123	0.028
10	8	1	0.5	0.8	0.5	0.30	0.30	0.30	0.50	0.00	0.00	0.069	-0.053	0.038
10	8	1	0.5	0.8	0.5	0.30	0.30	0.30	0.50	0.00	0.25	0.080	-0.042	0.044
10	8	1	0.5	0.8	0.5	0.30	0.30	0.30	0.50	0.00	0.50	0.088	-0.040	0.040
10	8	1	0.5	0.8	0.5	0.30	0.30	0.30	0.50	0.25	0.00	0.100	-0.071	0.029
10	8	1	0.5	0.8	0.5	0.30	0.30	0.30	0.50	0.25	0.25	0.094	-0.076	0.033
10	8	1	0.5	0.8	0.5	0.30	0.30	0.30	0.50	0.25	0.50	0.097	-0.066	0.034
10	8	1	0.5	0.8	0.5	0.30	0.30	0.30	0.50	0.50	0.00	0.128	-0.093	0.023

(continued)

												Mean Bias		
N	m_1	$\frac{m_2}{m_1}$	$E(C)$	$E(R)$	$E(U)$	σ_C	σ_R	σ_U	ρ_{CR}	ρ_{CU}	ρ_{RU}	c	r	u
10	8	1	0.5	0.8	0.5	0.30	0.30	0.30	0.50	0.50	0.25	0.125	-0.097	0.023
10	8	1	0.5	0.8	0.5	0.30	0.30	0.30	0.50	0.50	0.50	0.122	-0.090	0.020
10	8	1	0.8	0.2	0.5	0.00	0.00	0.00	0.00	0.00	0.00	-0.003	0.000	-0.001
10	8	1	0.8	0.2	0.5	0.15	0.15	0.15	0.00	0.00	0.00	0.004	-0.001	-0.001
10	8	1	0.8	0.2	0.5	0.15	0.15	0.15	0.00	0.00	0.25	0.004	0.000	0.004
10	8	1	0.8	0.2	0.5	0.15	0.15	0.15	0.00	0.00	0.50	0.007	-0.003	0.002
10	8	1	0.8	0.2	0.5	0.15	0.15	0.15	0.00	0.25	0.00	0.010	-0.005	0.000
10	8	1	0.8	0.2	0.5	0.15	0.15	0.15	0.00	0.25	0.25	0.008	-0.007	0.002
10	8	1	0.8	0.2	0.5	0.15	0.15	0.15	0.00	0.25	0.50	0.007	-0.005	0.003
10	8	1	0.8	0.2	0.5	0.15	0.15	0.15	0.00	0.50	0.00	0.024	-0.005	-0.003
10	8	1	0.8	0.2	0.5	0.15	0.15	0.15	0.00	0.50	0.25	0.017	-0.003	0.002
10	8	1	0.8	0.2	0.5	0.15	0.15	0.15	0.00	0.50	0.50	0.019	-0.002	0.001
10	8	1	0.8	0.2	0.5	0.15	0.15	0.15	0.25	0.00	0.00	0.008	0.008	0.004
10	8	1	0.8	0.2	0.5	0.15	0.15	0.15	0.25	0.00	0.25	0.002	0.006	0.007
10	8	1	0.8	0.2	0.5	0.15	0.15	0.15	0.25	0.00	0.50	0.004	0.002	0.004
10	8	1	0.8	0.2	0.5	0.15	0.15	0.15	0.25	0.25	0.00	0.011	0.004	0.005
10	8	1	0.8	0.2	0.5	0.15	0.15	0.15	0.25	0.25	0.25	0.010	0.002	0.003
10	8	1	0.8	0.2	0.5	0.15	0.15	0.15	0.25	0.25	0.50	0.013	0.001	0.002
10	8	1	0.8	0.2	0.5	0.15	0.15	0.15	0.25	0.50	0.00	0.018	0.002	-0.001
10	8	1	0.8	0.2	0.5	0.15	0.15	0.15	0.25	0.50	0.25	0.010	-0.001	-0.002
10	8	1	0.8	0.2	0.5	0.15	0.15	0.15	0.25	0.50	0.50	0.014	0.004	-0.001
10	8	1	0.8	0.2	0.5	0.15	0.15	0.15	0.50	0.00	0.00	0.006	0.008	0.004
10	8	1	0.8	0.2	0.5	0.15	0.15	0.15	0.50	0.00	0.25	0.005	0.011	0.004
10	8	1	0.8	0.2	0.5	0.15	0.15	0.15	0.50	0.00	0.50	0.006	0.008	0.006
10	8	1	0.8	0.2	0.5	0.15	0.15	0.15	0.50	0.25	0.00	0.013	0.008	0.002
10	8	1	0.8	0.2	0.5	0.15	0.15	0.15	0.50	0.25	0.25	0.011	0.004	-0.002
10	8	1	0.8	0.2	0.5	0.15	0.15	0.15	0.50	0.25	0.50	0.009	0.011	0.002
10	8	1	0.8	0.2	0.5	0.15	0.15	0.15	0.50	0.50	0.00	0.015	0.008	0.002
10	8	1	0.8	0.2	0.5	0.15	0.15	0.15	0.50	0.50	0.25	0.016	0.008	-0.003
10	8	1	0.8	0.2	0.5	0.15	0.15	0.15	0.50	0.50	0.50	0.015	0.007	-0.006
10	8	1	0.8	0.2	0.5	0.30	0.30	0.30	0.00	0.00	0.00	0.025	-0.009	0.020
10	8	1	0.8	0.2	0.5	0.30	0.30	0.30	0.00	0.00	0.25	0.025	-0.005	0.026
10	8	1	0.8	0.2	0.5	0.30	0.30	0.30	0.00	0.00	0.50	0.029	-0.002	0.019
10	8	1	0.8	0.2	0.5	0.30	0.30	0.30	0.00	0.25	0.00	0.045	-0.011	0.014
10	8	1	0.8	0.2	0.5	0.30	0.30	0.30	0.00	0.25	0.25	0.050	-0.013	0.007
10	8	1	0.8	0.2	0.5	0.30	0.30	0.30	0.00	0.25	0.50	0.053	-0.017	0.010
10	8	1	0.8	0.2	0.5	0.30	0.30	0.30	0.00	0.50	0.00	0.075	-0.018	0.007
10	8	1	0.8	0.2	0.5	0.30	0.30	0.30	0.00	0.50	0.25	0.073	-0.014	0.006
10	8	1	0.8	0.2	0.5	0.30	0.30	0.30	0.00	0.50	0.50	0.073	-0.012	0.003

(continued)

N	m_1	$\frac{m_2}{m_1}$	$E(C)$	$E(R)$	$E(U)$	σ_C	σ_R	σ_U	ρ_{CR}	ρ_{CU}	ρ_{RU}	Mean Bias c	Mean Bias r	Mean Bias u
10	8	1	0.8	0.2	0.5	0.30	0.30	0.30	0.25	0.00	0.00	0.032	0.011	0.024
10	8	1	0.8	0.2	0.5	0.30	0.30	0.30	0.25	0.00	0.25	0.034	0.013	0.013
10	8	1	0.8	0.2	0.5	0.30	0.30	0.30	0.25	0.00	0.50	0.031	0.010	0.016
10	8	1	0.8	0.2	0.5	0.30	0.30	0.30	0.25	0.25	0.00	0.045	0.001	0.012
10	8	1	0.8	0.2	0.5	0.30	0.30	0.30	0.25	0.25	0.25	0.047	0.005	0.001
10	8	1	0.8	0.2	0.5	0.30	0.30	0.30	0.25	0.25	0.50	0.050	0.002	0.011
10	8	1	0.8	0.2	0.5	0.30	0.30	0.30	0.25	0.50	0.00	0.070	-0.007	-0.002
10	8	1	0.8	0.2	0.5	0.30	0.30	0.30	0.25	0.50	0.25	0.070	0.000	0.004
10	8	1	0.8	0.2	0.5	0.30	0.30	0.30	0.25	0.50	0.50	0.076	-0.001	-0.001
10	8	1	0.8	0.2	0.5	0.30	0.30	0.30	0.50	0.00	0.00	0.024	0.024	0.021
10	8	1	0.8	0.2	0.5	0.30	0.30	0.30	0.50	0.00	0.25	0.021	0.030	0.021
10	8	1	0.8	0.2	0.5	0.30	0.30	0.30	0.50	0.00	0.50	0.031	0.023	0.019
10	8	1	0.8	0.2	0.5	0.30	0.30	0.30	0.50	0.25	0.00	0.047	0.020	0.014
10	8	1	0.8	0.2	0.5	0.30	0.30	0.30	0.50	0.25	0.25	0.049	0.022	0.013
10	8	1	0.8	0.2	0.5	0.30	0.30	0.30	0.50	0.25	0.50	0.050	0.015	0.009
10	8	1	0.8	0.2	0.5	0.30	0.30	0.30	0.50	0.50	0.00	0.069	0.005	-0.004
10	8	1	0.8	0.2	0.5	0.30	0.30	0.30	0.50	0.50	0.25	0.073	0.017	0.002
10	8	1	0.8	0.2	0.5	0.30	0.30	0.30	0.50	0.50	0.50	0.074	0.016	0.002
10	8	1	0.8	0.5	0.5	0.00	0.00	0.00	0.00	0.00	0.00	0.001	0.005	-0.001
10	8	1	0.8	0.5	0.5	0.15	0.15	0.15	0.00	0.00	0.00	0.005	-0.004	0.005
10	8	1	0.8	0.5	0.5	0.15	0.15	0.15	0.00	0.00	0.25	0.006	-0.001	0.006
10	8	1	0.8	0.5	0.5	0.15	0.15	0.15	0.00	0.00	0.50	0.001	0.002	0.007
10	8	1	0.8	0.5	0.5	0.15	0.15	0.15	0.00	0.25	0.00	0.009	-0.002	0.004
10	8	1	0.8	0.5	0.5	0.15	0.15	0.15	0.00	0.25	0.25	0.013	-0.005	0.003
10	8	1	0.8	0.5	0.5	0.15	0.15	0.15	0.00	0.25	0.50	0.007	-0.009	0.000
10	8	1	0.8	0.5	0.5	0.15	0.15	0.15	0.00	0.50	0.00	0.014	-0.010	-0.004
10	8	1	0.8	0.5	0.5	0.15	0.15	0.15	0.00	0.50	0.25	0.018	-0.011	0.000
10	8	1	0.8	0.5	0.5	0.15	0.15	0.15	0.00	0.50	0.50	0.020	-0.013	-0.001
10	8	1	0.8	0.5	0.5	0.15	0.15	0.15	0.25	0.00	0.00	0.007	-0.003	0.003
10	8	1	0.8	0.5	0.5	0.15	0.15	0.15	0.25	0.00	0.25	0.004	0.006	0.002
10	8	1	0.8	0.5	0.5	0.15	0.15	0.15	0.25	0.00	0.50	0.008	0.005	0.007
10	8	1	0.8	0.5	0.5	0.15	0.15	0.15	0.25	0.25	0.00	0.005	0.001	0.001
10	8	1	0.8	0.5	0.5	0.15	0.15	0.15	0.25	0.25	0.25	0.010	-0.001	0.002
10	8	1	0.8	0.5	0.5	0.15	0.15	0.15	0.25	0.25	0.50	0.011	-0.001	0.002
10	8	1	0.8	0.5	0.5	0.15	0.15	0.15	0.25	0.50	0.00	0.016	-0.006	0.002
10	8	1	0.8	0.5	0.5	0.15	0.15	0.15	0.25	0.50	0.25	0.017	-0.004	-0.002
10	8	1	0.8	0.5	0.5	0.15	0.15	0.15	0.25	0.50	0.50	0.019	-0.008	-0.003
10	8	1	0.8	0.5	0.5	0.15	0.15	0.15	0.50	0.00	0.00	0.007	0.012	0.005
10	8	1	0.8	0.5	0.5	0.15	0.15	0.15	0.50	0.00	0.25	0.007	0.008	0.003

(continued)

												Mean Bias		
N	m_1	$\frac{m_2}{m_1}$	$E(C)$	$E(R)$	$E(U)$	σ_C	σ_R	σ_U	ρ_{CR}	ρ_{CU}	ρ_{RU}	c	r	u
10	8	1	0.8	0.5	0.5	0.15	0.15	0.15	0.50	0.00	0.50	0.007	0.010	0.006
10	8	1	0.8	0.5	0.5	0.15	0.15	0.15	0.50	0.25	0.00	0.013	0.007	0.000
10	8	1	0.8	0.5	0.5	0.15	0.15	0.15	0.50	0.25	0.25	0.010	0.008	-0.002
10	8	1	0.8	0.5	0.5	0.15	0.15	0.15	0.50	0.25	0.50	0.012	0.009	0.002
10	8	1	0.8	0.5	0.5	0.15	0.15	0.15	0.50	0.50	0.00	0.021	0.003	0.002
10	8	1	0.8	0.5	0.5	0.15	0.15	0.15	0.50	0.50	0.25	0.019	0.003	-0.002
10	8	1	0.8	0.5	0.5	0.15	0.15	0.15	0.50	0.50	0.50	0.019	0.007	0.002
10	8	1	0.8	0.5	0.5	0.30	0.30	0.30	0.00	0.00	0.00	0.024	-0.014	0.015
10	8	1	0.8	0.5	0.5	0.30	0.30	0.30	0.00	0.00	0.25	0.031	-0.015	0.017
10	8	1	0.8	0.5	0.5	0.30	0.30	0.30	0.00	0.00	0.50	0.027	-0.009	0.018
10	8	1	0.8	0.5	0.5	0.30	0.30	0.30	0.00	0.25	0.00	0.049	-0.030	0.015
10	8	1	0.8	0.5	0.5	0.30	0.30	0.30	0.00	0.25	0.25	0.049	-0.030	0.015
10	8	1	0.8	0.5	0.5	0.30	0.30	0.30	0.00	0.25	0.50	0.048	-0.023	0.014
10	8	1	0.8	0.5	0.5	0.30	0.30	0.30	0.00	0.50	0.00	0.074	-0.044	0.005
10	8	1	0.8	0.5	0.5	0.30	0.30	0.30	0.00	0.50	0.25	0.074	-0.036	0.003
10	8	1	0.8	0.5	0.5	0.30	0.30	0.30	0.00	0.50	0.50	0.069	-0.045	-0.004
10	8	1	0.8	0.5	0.5	0.30	0.30	0.30	0.25	0.00	0.00	0.030	0.011	0.020
10	8	1	0.8	0.5	0.5	0.30	0.30	0.30	0.25	0.00	0.25	0.028	0.006	0.021
10	8	1	0.8	0.5	0.5	0.30	0.30	0.30	0.25	0.00	0.50	0.027	0.000	0.018
10	8	1	0.8	0.5	0.5	0.30	0.30	0.30	0.25	0.25	0.00	0.047	-0.006	0.012
10	8	1	0.8	0.5	0.5	0.30	0.30	0.30	0.25	0.25	0.25	0.047	-0.004	0.014
10	8	1	0.8	0.5	0.5	0.30	0.30	0.30	0.25	0.25	0.50	0.048	-0.007	0.014
10	8	1	0.8	0.5	0.5	0.30	0.30	0.30	0.25	0.50	0.00	0.072	-0.022	0.005
10	8	1	0.8	0.5	0.5	0.30	0.30	0.30	0.25	0.50	0.25	0.070	-0.022	0.003
10	8	1	0.8	0.5	0.5	0.30	0.30	0.30	0.25	0.50	0.50	0.073	-0.025	0.005
10	8	1	0.8	0.5	0.5	0.30	0.30	0.30	0.50	0.00	0.00	0.029	0.033	0.020
10	8	1	0.8	0.5	0.5	0.30	0.30	0.30	0.50	0.00	0.25	0.023	0.027	0.021
10	8	1	0.8	0.5	0.5	0.30	0.30	0.30	0.50	0.00	0.50	0.024	0.018	0.016
10	8	1	0.8	0.5	0.5	0.30	0.30	0.30	0.50	0.25	0.00	0.048	0.011	0.012
10	8	1	0.8	0.5	0.5	0.30	0.30	0.30	0.50	0.25	0.25	0.051	0.012	0.006
10	8	1	0.8	0.5	0.5	0.30	0.30	0.30	0.50	0.25	0.50	0.046	0.014	0.007
10	8	1	0.8	0.5	0.5	0.30	0.30	0.30	0.50	0.50	0.00	0.070	-0.001	-0.005
10	8	1	0.8	0.5	0.5	0.30	0.30	0.30	0.50	0.50	0.25	0.073	-0.002	0.005
10	8	1	0.8	0.5	0.5	0.30	0.30	0.30	0.50	0.50	0.50	0.072	0.002	0.005
10	8	1	0.8	0.8	0.5	0.00	0.00	0.00	0.00	0.00	0.00	0.000	0.003	0.001
10	8	1	0.8	0.8	0.5	0.15	0.15	0.15	0.00	0.00	0.00	0.005	-0.004	0.003
10	8	1	0.8	0.8	0.5	0.15	0.15	0.15	0.00	0.00	0.25	0.003	-0.001	0.001
10	8	1	0.8	0.8	0.5	0.15	0.15	0.15	0.00	0.00	0.50	0.006	-0.004	0.005
10	8	1	0.8	0.8	0.5	0.15	0.15	0.15	0.00	0.25	0.00	0.009	-0.006	-0.001

(continued)

N	m_1	$\frac{m_2}{m_1}$	$E(C)$	$E(R)$	$E(U)$	σ_C	σ_R	σ_U	ρ_{CR}	ρ_{CU}	ρ_{RU}	Mean Bias c	Mean Bias r	Mean Bias u
10	8	1	0.8	0.8	0.5	0.15	0.15	0.15	0.00	0.25	0.25	0.013	-0.008	0.002
10	8	1	0.8	0.8	0.5	0.15	0.15	0.15	0.00	0.25	0.50	0.010	-0.012	0.004
10	8	1	0.8	0.8	0.5	0.15	0.15	0.15	0.00	0.50	0.00	0.020	-0.017	-0.001
10	8	1	0.8	0.8	0.5	0.15	0.15	0.15	0.00	0.50	0.25	0.014	-0.012	-0.002
10	8	1	0.8	0.8	0.5	0.15	0.15	0.15	0.00	0.50	0.50	0.016	-0.018	-0.003
10	8	1	0.8	0.8	0.5	0.15	0.15	0.15	0.25	0.00	0.00	0.005	0.002	0.005
10	8	1	0.8	0.8	0.5	0.15	0.15	0.15	0.25	0.00	0.25	0.004	0.003	0.006
10	8	1	0.8	0.8	0.5	0.15	0.15	0.15	0.25	0.00	0.50	0.004	0.001	0.001
10	8	1	0.8	0.8	0.5	0.15	0.15	0.15	0.25	0.25	0.00	0.014	-0.004	0.003
10	8	1	0.8	0.8	0.5	0.15	0.15	0.15	0.25	0.25	0.25	0.013	-0.005	0.003
10	8	1	0.8	0.8	0.5	0.15	0.15	0.15	0.25	0.25	0.50	0.014	-0.003	0.009
10	8	1	0.8	0.8	0.5	0.15	0.15	0.15	0.25	0.50	0.00	0.019	-0.008	0.002
10	8	1	0.8	0.8	0.5	0.15	0.15	0.15	0.25	0.50	0.25	0.021	-0.007	0.000
10	8	1	0.8	0.8	0.5	0.15	0.15	0.15	0.25	0.50	0.50	0.018	-0.011	0.001
10	8	1	0.8	0.8	0.5	0.15	0.15	0.15	0.50	0.00	0.00	0.007	0.012	0.004
10	8	1	0.8	0.8	0.5	0.15	0.15	0.15	0.50	0.00	0.25	0.003	0.008	0.004
10	8	1	0.8	0.8	0.5	0.15	0.15	0.15	0.50	0.00	0.50	0.006	0.004	0.004
10	8	1	0.8	0.8	0.5	0.15	0.15	0.15	0.50	0.25	0.00	0.011	0.000	0.004
10	8	1	0.8	0.8	0.5	0.15	0.15	0.15	0.50	0.25	0.25	0.014	0.001	0.001
10	8	1	0.8	0.8	0.5	0.15	0.15	0.15	0.50	0.25	0.50	0.010	0.004	0.002
10	8	1	0.8	0.8	0.5	0.15	0.15	0.15	0.50	0.50	0.00	0.020	-0.004	0.000
10	8	1	0.8	0.8	0.5	0.15	0.15	0.15	0.50	0.50	0.25	0.021	-0.003	0.001
10	8	1	0.8	0.8	0.5	0.15	0.15	0.15	0.50	0.50	0.50	0.018	-0.002	0.001
10	8	1	0.8	0.8	0.5	0.30	0.30	0.30	0.00	0.00	0.00	0.027	-0.024	0.017
10	8	1	0.8	0.8	0.5	0.30	0.30	0.30	0.00	0.00	0.25	0.027	-0.021	0.017
10	8	1	0.8	0.8	0.5	0.30	0.30	0.30	0.00	0.00	0.50	0.030	-0.026	0.018
10	8	1	0.8	0.8	0.5	0.30	0.30	0.30	0.00	0.25	0.00	0.049	-0.044	0.013
10	8	1	0.8	0.8	0.5	0.30	0.30	0.30	0.00	0.25	0.25	0.051	-0.046	0.009
10	8	1	0.8	0.8	0.5	0.30	0.30	0.30	0.00	0.25	0.50	0.052	-0.048	0.011
10	8	1	0.8	0.8	0.5	0.30	0.30	0.30	0.00	0.50	0.00	0.074	-0.064	0.002
10	8	1	0.8	0.8	0.5	0.30	0.30	0.30	0.00	0.50	0.25	0.072	-0.066	0.005
10	8	1	0.8	0.8	0.5	0.30	0.30	0.30	0.00	0.50	0.50	0.076	-0.060	0.008
10	8	1	0.8	0.8	0.5	0.30	0.30	0.30	0.25	0.00	0.00	0.026	-0.002	0.025
10	8	1	0.8	0.8	0.5	0.30	0.30	0.30	0.25	0.00	0.25	0.027	-0.006	0.021
10	8	1	0.8	0.8	0.5	0.30	0.30	0.30	0.25	0.00	0.50	0.029	-0.008	0.018
10	8	1	0.8	0.8	0.5	0.30	0.30	0.30	0.25	0.25	0.00	0.046	-0.022	0.010
10	8	1	0.8	0.8	0.5	0.30	0.30	0.30	0.25	0.25	0.25	0.048	-0.027	0.010
10	8	1	0.8	0.8	0.5	0.30	0.30	0.30	0.25	0.25	0.50	0.051	-0.030	0.011
10	8	1	0.8	0.8	0.5	0.30	0.30	0.30	0.25	0.50	0.00	0.076	-0.048	0.005

(continued)

												Mean Bias		
N	m_1	$\frac{m_2}{m_1}$	$E(C)$	$E(R)$	$E(U)$	σ_C	σ_R	σ_U	ρ_{CR}	ρ_{CU}	ρ_{RU}	c	r	u
10	8	1	0.8	0.8	0.5	0.30	0.30	0.30	0.25	0.50	0.25	0.071	-0.045	0.003
10	8	1	0.8	0.8	0.5	0.30	0.30	0.30	0.25	0.50	0.50	0.074	-0.048	0.002
10	8	1	0.8	0.8	0.5	0.30	0.30	0.30	0.50	0.00	0.00	0.026	0.016	0.014
10	8	1	0.8	0.8	0.5	0.30	0.30	0.30	0.50	0.00	0.25	0.028	0.018	0.019
10	8	1	0.8	0.8	0.5	0.30	0.30	0.30	0.50	0.00	0.50	0.029	0.016	0.020
10	8	1	0.8	0.8	0.5	0.30	0.30	0.30	0.50	0.25	0.00	0.049	-0.005	0.011
10	8	1	0.8	0.8	0.5	0.30	0.30	0.30	0.50	0.25	0.25	0.050	-0.009	0.005
10	8	1	0.8	0.8	0.5	0.30	0.30	0.30	0.50	0.25	0.50	0.050	-0.006	0.011
10	8	1	0.8	0.8	0.5	0.30	0.30	0.30	0.50	0.50	0.00	0.071	-0.026	-0.002
10	8	1	0.8	0.8	0.5	0.30	0.30	0.30	0.50	0.50	0.25	0.073	-0.029	0.005
10	8	1	0.8	0.8	0.5	0.30	0.30	0.30	0.50	0.50	0.50	0.074	-0.028	-0.002
10	20	0	0.2	0.2	0.5	0.00	0.00	0.00	0.00	0.00	0.00	-0.007	0.051	0.001
10	20	0	0.2	0.2	0.5	0.15	0.15	0.15	0.00	0.00	0.00	0.052	-0.025	0.039
10	20	0	0.2	0.2	0.5	0.15	0.15	0.15	0.00	0.00	0.25	0.050	-0.026	0.037
10	20	0	0.2	0.2	0.5	0.15	0.15	0.15	0.00	0.00	0.50	0.051	-0.026	0.038
10	20	0	0.2	0.2	0.5	0.15	0.15	0.15	0.00	0.25	0.00	0.054	-0.021	0.033
10	20	0	0.2	0.2	0.5	0.15	0.15	0.15	0.00	0.25	0.25	0.063	-0.033	0.034
10	20	0	0.2	0.2	0.5	0.15	0.15	0.15	0.00	0.25	0.50	0.059	-0.023	0.036
10	20	0	0.2	0.2	0.5	0.15	0.15	0.15	0.00	0.50	0.00	0.053	-0.026	0.024
10	20	0	0.2	0.2	0.5	0.15	0.15	0.15	0.00	0.50	0.25	0.057	-0.025	0.029
10	20	0	0.2	0.2	0.5	0.15	0.15	0.15	0.00	0.50	0.50	0.055	-0.030	0.027
10	20	0	0.2	0.2	0.5	0.15	0.15	0.15	0.25	0.00	0.00	0.055	-0.007	0.037
10	20	0	0.2	0.2	0.5	0.15	0.15	0.15	0.25	0.00	0.25	0.055	-0.007	0.036
10	20	0	0.2	0.2	0.5	0.15	0.15	0.15	0.25	0.00	0.50	0.053	-0.008	0.039
10	20	0	0.2	0.2	0.5	0.15	0.15	0.15	0.25	0.25	0.00	0.055	-0.009	0.033
10	20	0	0.2	0.2	0.5	0.15	0.15	0.15	0.25	0.25	0.25	0.053	-0.004	0.033
10	20	0	0.2	0.2	0.5	0.15	0.15	0.15	0.25	0.25	0.50	0.052	-0.003	0.030
10	20	0	0.2	0.2	0.5	0.15	0.15	0.15	0.25	0.50	0.00	0.058	-0.007	0.031
10	20	0	0.2	0.2	0.5	0.15	0.15	0.15	0.25	0.50	0.25	0.056	-0.004	0.025
10	20	0	0.2	0.2	0.5	0.15	0.15	0.15	0.25	0.50	0.50	0.062	-0.013	0.028
10	20	0	0.2	0.2	0.5	0.15	0.15	0.15	0.50	0.00	0.00	0.052	0.016	0.037
10	20	0	0.2	0.2	0.5	0.15	0.15	0.15	0.50	0.00	0.25	0.053	0.016	0.038
10	20	0	0.2	0.2	0.5	0.15	0.15	0.15	0.50	0.00	0.50	0.051	0.012	0.042
10	20	0	0.2	0.2	0.5	0.15	0.15	0.15	0.50	0.25	0.00	0.054	0.021	0.035
10	20	0	0.2	0.2	0.5	0.15	0.15	0.15	0.50	0.25	0.25	0.057	0.014	0.035
10	20	0	0.2	0.2	0.5	0.15	0.15	0.15	0.50	0.25	0.50	0.056	0.014	0.038
10	20	0	0.2	0.2	0.5	0.15	0.15	0.15	0.50	0.50	0.00	0.057	0.020	0.028
10	20	0	0.2	0.2	0.5	0.15	0.15	0.15	0.50	0.50	0.25	0.055	0.011	0.025
10	20	0	0.2	0.2	0.5	0.15	0.15	0.15	0.50	0.50	0.50	0.058	0.018	0.029

(continued)

												Mean Bias		
N	m_1	$\frac{m_2}{m_1}$	$E(C)$	$E(R)$	$E(U)$	σ_C	σ_R	σ_U	ρ_{CR}	ρ_{CU}	ρ_{RU}	c	r	u
10	20	0	0.2	0.2	0.5	0.30	0.30	0.30	0.00	0.00	0.00	0.197	-0.099	0.163
10	20	0	0.2	0.2	0.5	0.30	0.30	0.30	0.00	0.00	0.25	0.204	-0.105	0.167
10	20	0	0.2	0.2	0.5	0.30	0.30	0.30	0.00	0.00	0.50	0.202	-0.102	0.164
10	20	0	0.2	0.2	0.5	0.30	0.30	0.30	0.00	0.25	0.00	0.207	-0.100	0.148
10	20	0	0.2	0.2	0.5	0.30	0.30	0.30	0.00	0.25	0.25	0.215	-0.104	0.154
10	20	0	0.2	0.2	0.5	0.30	0.30	0.30	0.00	0.25	0.50	0.213	-0.101	0.148
10	20	0	0.2	0.2	0.5	0.30	0.30	0.30	0.00	0.50	0.00	0.219	-0.102	0.131
10	20	0	0.2	0.2	0.5	0.30	0.30	0.30	0.00	0.50	0.25	0.218	-0.105	0.122
10	20	0	0.2	0.2	0.5	0.30	0.30	0.30	0.00	0.50	0.50	0.216	-0.101	0.127
10	20	0	0.2	0.2	0.5	0.30	0.30	0.30	0.25	0.00	0.00	0.201	-0.055	0.164
10	20	0	0.2	0.2	0.5	0.30	0.30	0.30	0.25	0.00	0.25	0.196	-0.059	0.161
10	20	0	0.2	0.2	0.5	0.30	0.30	0.30	0.25	0.00	0.50	0.199	-0.059	0.164
10	20	0	0.2	0.2	0.5	0.30	0.30	0.30	0.25	0.25	0.00	0.208	-0.063	0.144
10	20	0	0.2	0.2	0.5	0.30	0.30	0.30	0.25	0.25	0.25	0.208	-0.060	0.147
10	20	0	0.2	0.2	0.5	0.30	0.30	0.30	0.25	0.25	0.50	0.211	-0.062	0.146
10	20	0	0.2	0.2	0.5	0.30	0.30	0.30	0.25	0.50	0.00	0.218	-0.066	0.127
10	20	0	0.2	0.2	0.5	0.30	0.30	0.30	0.25	0.50	0.25	0.218	-0.065	0.120
10	20	0	0.2	0.2	0.5	0.30	0.30	0.30	0.25	0.50	0.50	0.216	-0.061	0.122
10	20	0	0.2	0.2	0.5	0.30	0.30	0.30	0.50	0.00	0.00	0.191	-0.011	0.165
10	20	0	0.2	0.2	0.5	0.30	0.30	0.30	0.50	0.00	0.25	0.207	-0.015	0.163
10	20	0	0.2	0.2	0.5	0.30	0.30	0.30	0.50	0.00	0.50	0.199	-0.016	0.161
10	20	0	0.2	0.2	0.5	0.30	0.30	0.30	0.50	0.25	0.00	0.203	-0.017	0.149
10	20	0	0.2	0.2	0.5	0.30	0.30	0.30	0.50	0.25	0.25	0.217	-0.023	0.146
10	20	0	0.2	0.2	0.5	0.30	0.30	0.30	0.50	0.25	0.50	0.215	-0.011	0.150
10	20	0	0.2	0.2	0.5	0.30	0.30	0.30	0.50	0.50	0.00	0.222	-0.020	0.129
10	20	0	0.2	0.2	0.5	0.30	0.30	0.30	0.50	0.50	0.25	0.217	-0.017	0.134
10	20	0	0.2	0.2	0.5	0.30	0.30	0.30	0.50	0.50	0.50	0.212	-0.024	0.126
10	20	0	0.2	0.5	0.5	0.00	0.00	0.00	0.00	0.00	0.00	-0.004	0.064	0.001
10	20	0	0.2	0.5	0.5	0.15	0.15	0.15	0.00	0.00	0.00	0.053	-0.083	0.041
10	20	0	0.2	0.5	0.5	0.15	0.15	0.15	0.00	0.00	0.25	0.053	-0.084	0.039
10	20	0	0.2	0.5	0.5	0.15	0.15	0.15	0.00	0.00	0.50	0.051	-0.082	0.038
10	20	0	0.2	0.5	0.5	0.15	0.15	0.15	0.00	0.25	0.00	0.061	-0.081	0.036
10	20	0	0.2	0.5	0.5	0.15	0.15	0.15	0.00	0.25	0.25	0.057	-0.083	0.033
10	20	0	0.2	0.5	0.5	0.15	0.15	0.15	0.00	0.25	0.50	0.052	-0.072	0.034
10	20	0	0.2	0.5	0.5	0.15	0.15	0.15	0.00	0.50	0.00	0.057	-0.082	0.031
10	20	0	0.2	0.5	0.5	0.15	0.15	0.15	0.00	0.50	0.25	0.059	-0.084	0.033
10	20	0	0.2	0.5	0.5	0.15	0.15	0.15	0.00	0.50	0.50	0.058	-0.087	0.028
10	20	0	0.2	0.5	0.5	0.15	0.15	0.15	0.25	0.00	0.00	0.051	-0.054	0.033
10	20	0	0.2	0.5	0.5	0.15	0.15	0.15	0.25	0.00	0.25	0.056	-0.057	0.043

(continued)

												Mean Bias		
N	m_1	$\frac{m_2}{m_1}$	$E(C)$	$E(R)$	$E(U)$	σ_C	σ_R	σ_U	ρ_{CR}	ρ_{CU}	ρ_{RU}	c	r	u
10	20	0	0.2	0.5	0.5	0.15	0.15	0.15	0.25	0.00	0.50	0.051	-0.051	0.037
10	20	0	0.2	0.5	0.5	0.15	0.15	0.15	0.25	0.25	0.00	0.060	-0.066	0.036
10	20	0	0.2	0.5	0.5	0.15	0.15	0.15	0.25	0.25	0.25	0.059	-0.066	0.033
10	20	0	0.2	0.5	0.5	0.15	0.15	0.15	0.25	0.25	0.50	0.055	-0.062	0.032
10	20	0	0.2	0.5	0.5	0.15	0.15	0.15	0.25	0.50	0.00	0.059	-0.064	0.027
10	20	0	0.2	0.5	0.5	0.15	0.15	0.15	0.25	0.50	0.25	0.058	-0.061	0.025
10	20	0	0.2	0.5	0.5	0.15	0.15	0.15	0.25	0.50	0.50	0.056	-0.064	0.028
10	20	0	0.2	0.5	0.5	0.15	0.15	0.15	0.50	0.00	0.00	0.055	-0.036	0.041
10	20	0	0.2	0.5	0.5	0.15	0.15	0.15	0.50	0.00	0.25	0.055	-0.034	0.039
10	20	0	0.2	0.5	0.5	0.15	0.15	0.15	0.50	0.00	0.50	0.053	-0.038	0.038
10	20	0	0.2	0.5	0.5	0.15	0.15	0.15	0.50	0.25	0.00	0.054	-0.035	0.034
10	20	0	0.2	0.5	0.5	0.15	0.15	0.15	0.50	0.25	0.25	0.060	-0.037	0.040
10	20	0	0.2	0.5	0.5	0.15	0.15	0.15	0.50	0.25	0.50	0.054	-0.045	0.031
10	20	0	0.2	0.5	0.5	0.15	0.15	0.15	0.50	0.50	0.00	0.056	-0.052	0.027
10	20	0	0.2	0.5	0.5	0.15	0.15	0.15	0.50	0.50	0.25	0.061	-0.040	0.029
10	20	0	0.2	0.5	0.5	0.15	0.15	0.15	0.50	0.50	0.50	0.053	-0.035	0.023
10	20	0	0.2	0.5	0.5	0.30	0.30	0.30	0.00	0.00	0.00	0.200	-0.251	0.165
10	20	0	0.2	0.5	0.5	0.30	0.30	0.30	0.00	0.00	0.25	0.200	-0.247	0.166
10	20	0	0.2	0.5	0.5	0.30	0.30	0.30	0.00	0.00	0.50	0.199	-0.251	0.164
10	20	0	0.2	0.5	0.5	0.30	0.30	0.30	0.00	0.25	0.00	0.213	-0.254	0.144
10	20	0	0.2	0.5	0.5	0.30	0.30	0.30	0.00	0.25	0.25	0.208	-0.258	0.150
10	20	0	0.2	0.5	0.5	0.30	0.30	0.30	0.00	0.25	0.50	0.210	-0.266	0.148
10	20	0	0.2	0.5	0.5	0.30	0.30	0.30	0.00	0.50	0.00	0.215	-0.259	0.122
10	20	0	0.2	0.5	0.5	0.30	0.30	0.30	0.00	0.50	0.25	0.222	-0.259	0.123
10	20	0	0.2	0.5	0.5	0.30	0.30	0.30	0.00	0.50	0.50	0.220	-0.259	0.125
10	20	0	0.2	0.5	0.5	0.30	0.30	0.30	0.25	0.00	0.00	0.200	-0.212	0.166
10	20	0	0.2	0.5	0.5	0.30	0.30	0.30	0.25	0.00	0.25	0.204	-0.198	0.167
10	20	0	0.2	0.5	0.5	0.30	0.30	0.30	0.25	0.00	0.50	0.194	-0.202	0.164
10	20	0	0.2	0.5	0.5	0.30	0.30	0.30	0.25	0.25	0.00	0.209	-0.217	0.150
10	20	0	0.2	0.5	0.5	0.30	0.30	0.30	0.25	0.25	0.25	0.206	-0.217	0.145
10	20	0	0.2	0.5	0.5	0.30	0.30	0.30	0.25	0.25	0.50	0.213	-0.213	0.148
10	20	0	0.2	0.5	0.5	0.30	0.30	0.30	0.25	0.50	0.00	0.219	-0.222	0.127
10	20	0	0.2	0.5	0.5	0.30	0.30	0.30	0.25	0.50	0.25	0.214	-0.222	0.129
10	20	0	0.2	0.5	0.5	0.30	0.30	0.30	0.25	0.50	0.50	0.219	-0.220	0.128
10	20	0	0.2	0.5	0.5	0.30	0.30	0.30	0.50	0.00	0.00	0.198	-0.159	0.170
10	20	0	0.2	0.5	0.5	0.30	0.30	0.30	0.50	0.00	0.25	0.198	-0.157	0.166
10	20	0	0.2	0.5	0.5	0.30	0.30	0.30	0.50	0.00	0.50	0.204	-0.166	0.168
10	20	0	0.2	0.5	0.5	0.30	0.30	0.30	0.50	0.25	0.00	0.211	-0.175	0.147
10	20	0	0.2	0.5	0.5	0.30	0.30	0.30	0.50	0.25	0.25	0.214	-0.171	0.150

(continued)

N	m_1	$\frac{m_2}{m_1}$	$E(C)$	$E(R)$	$E(U)$	σ_C	σ_R	σ_U	ρ_{CR}	ρ_{CU}	ρ_{RU}	Mean Bias c	Mean Bias r	Mean Bias u
10	20	0	0.2	0.5	0.5	0.30	0.30	0.30	0.50	0.25	0.50	0.213	-0.168	0.144
10	20	0	0.2	0.5	0.5	0.30	0.30	0.30	0.50	0.50	0.00	0.212	-0.173	0.124
10	20	0	0.2	0.5	0.5	0.30	0.30	0.30	0.50	0.50	0.25	0.218	-0.174	0.126
10	20	0	0.2	0.5	0.5	0.30	0.30	0.30	0.50	0.50	0.50	0.223	-0.187	0.120
10	20	0	0.2	0.8	0.5	0.00	0.00	0.00	0.00	0.00	0.00	0.005	0.009	0.006
10	20	0	0.2	0.8	0.5	0.15	0.15	0.15	0.00	0.00	0.00	0.059	-0.158	0.041
10	20	0	0.2	0.8	0.5	0.15	0.15	0.15	0.00	0.00	0.25	0.056	-0.158	0.042
10	20	0	0.2	0.8	0.5	0.15	0.15	0.15	0.00	0.00	0.50	0.057	-0.159	0.043
10	20	0	0.2	0.8	0.5	0.15	0.15	0.15	0.00	0.25	0.00	0.059	-0.156	0.035
10	20	0	0.2	0.8	0.5	0.15	0.15	0.15	0.00	0.25	0.25	0.058	-0.154	0.033
10	20	0	0.2	0.8	0.5	0.15	0.15	0.15	0.00	0.25	0.50	0.059	-0.149	0.036
10	20	0	0.2	0.8	0.5	0.15	0.15	0.15	0.00	0.50	0.00	0.061	-0.167	0.029
10	20	0	0.2	0.8	0.5	0.15	0.15	0.15	0.00	0.50	0.25	0.059	-0.157	0.029
10	20	0	0.2	0.8	0.5	0.15	0.15	0.15	0.00	0.50	0.50	0.056	-0.154	0.027
10	20	0	0.2	0.8	0.5	0.15	0.15	0.15	0.25	0.00	0.00	0.060	-0.142	0.046
10	20	0	0.2	0.8	0.5	0.15	0.15	0.15	0.25	0.00	0.25	0.057	-0.122	0.040
10	20	0	0.2	0.8	0.5	0.15	0.15	0.15	0.25	0.00	0.50	0.059	-0.134	0.040
10	20	0	0.2	0.8	0.5	0.15	0.15	0.15	0.25	0.25	0.00	0.055	-0.127	0.035
10	20	0	0.2	0.8	0.5	0.15	0.15	0.15	0.25	0.25	0.25	0.064	-0.148	0.036
10	20	0	0.2	0.8	0.5	0.15	0.15	0.15	0.25	0.25	0.50	0.062	-0.129	0.037
10	20	0	0.2	0.8	0.5	0.15	0.15	0.15	0.25	0.50	0.00	0.060	-0.137	0.027
10	20	0	0.2	0.8	0.5	0.15	0.15	0.15	0.25	0.50	0.25	0.063	-0.137	0.031
10	20	0	0.2	0.8	0.5	0.15	0.15	0.15	0.25	0.50	0.50	0.062	-0.144	0.030
10	20	0	0.2	0.8	0.5	0.15	0.15	0.15	0.50	0.00	0.00	0.054	-0.114	0.039
10	20	0	0.2	0.8	0.5	0.15	0.15	0.15	0.50	0.00	0.25	0.058	-0.114	0.040
10	20	0	0.2	0.8	0.5	0.15	0.15	0.15	0.50	0.00	0.50	0.057	-0.121	0.039
10	20	0	0.2	0.8	0.5	0.15	0.15	0.15	0.50	0.25	0.00	0.058	-0.122	0.035
10	20	0	0.2	0.8	0.5	0.15	0.15	0.15	0.50	0.25	0.25	0.059	-0.124	0.036
10	20	0	0.2	0.8	0.5	0.15	0.15	0.15	0.50	0.25	0.50	0.060	-0.117	0.034
10	20	0	0.2	0.8	0.5	0.15	0.15	0.15	0.50	0.50	0.00	0.059	-0.124	0.028
10	20	0	0.2	0.8	0.5	0.15	0.15	0.15	0.50	0.50	0.25	0.060	-0.132	0.026
10	20	0	0.2	0.8	0.5	0.15	0.15	0.15	0.50	0.50	0.50	0.059	-0.121	0.026
10	20	0	0.2	0.8	0.5	0.30	0.30	0.30	0.00	0.00	0.00	0.198	-0.406	0.166
10	20	0	0.2	0.8	0.5	0.30	0.30	0.30	0.00	0.00	0.25	0.202	-0.406	0.165
10	20	0	0.2	0.8	0.5	0.30	0.30	0.30	0.00	0.00	0.50	0.204	-0.402	0.162
10	20	0	0.2	0.8	0.5	0.30	0.30	0.30	0.00	0.25	0.00	0.209	-0.417	0.146
10	20	0	0.2	0.8	0.5	0.30	0.30	0.30	0.00	0.25	0.25	0.211	-0.410	0.154
10	20	0	0.2	0.8	0.5	0.30	0.30	0.30	0.00	0.25	0.50	0.213	-0.413	0.146
10	20	0	0.2	0.8	0.5	0.30	0.30	0.30	0.00	0.50	0.00	0.219	-0.427	0.127

(continued)

												Mean Bias		
N	m_1	$\frac{m_2}{m_1}$	$E(C)$	$E(R)$	$E(U)$	σ_C	σ_R	σ_U	ρ_{CR}	ρ_{CU}	ρ_{RU}	c	r	u
10	20	0	0.2	0.8	0.5	0.30	0.30	0.30	0.00	0.50	0.25	0.213	-0.407	0.129
10	20	0	0.2	0.8	0.5	0.30	0.30	0.30	0.00	0.50	0.50	0.219	-0.419	0.126
10	20	0	0.2	0.8	0.5	0.30	0.30	0.30	0.25	0.00	0.00	0.199	-0.358	0.165
10	20	0	0.2	0.8	0.5	0.30	0.30	0.30	0.25	0.00	0.25	0.199	-0.364	0.166
10	20	0	0.2	0.8	0.5	0.30	0.30	0.30	0.25	0.00	0.50	0.200	-0.369	0.160
10	20	0	0.2	0.8	0.5	0.30	0.30	0.30	0.25	0.25	0.00	0.219	-0.374	0.153
10	20	0	0.2	0.8	0.5	0.30	0.30	0.30	0.25	0.25	0.25	0.205	-0.390	0.148
10	20	0	0.2	0.8	0.5	0.30	0.30	0.30	0.25	0.25	0.50	0.215	-0.383	0.149
10	20	0	0.2	0.8	0.5	0.30	0.30	0.30	0.25	0.50	0.00	0.216	-0.396	0.125
10	20	0	0.2	0.8	0.5	0.30	0.30	0.30	0.25	0.50	0.25	0.219	-0.380	0.122
10	20	0	0.2	0.8	0.5	0.30	0.30	0.30	0.25	0.50	0.50	0.224	-0.389	0.127
10	20	0	0.2	0.8	0.5	0.30	0.30	0.30	0.50	0.00	0.00	0.203	-0.334	0.164
10	20	0	0.2	0.8	0.5	0.30	0.30	0.30	0.50	0.00	0.25	0.196	-0.338	0.166
10	20	0	0.2	0.8	0.5	0.30	0.30	0.30	0.50	0.00	0.50	0.197	-0.343	0.165
10	20	0	0.2	0.8	0.5	0.30	0.30	0.30	0.50	0.25	0.00	0.206	-0.346	0.146
10	20	0	0.2	0.8	0.5	0.30	0.30	0.30	0.50	0.25	0.25	0.212	-0.354	0.150
10	20	0	0.2	0.8	0.5	0.30	0.30	0.30	0.50	0.25	0.50	0.211	-0.351	0.151
10	20	0	0.2	0.8	0.5	0.30	0.30	0.30	0.50	0.50	0.00	0.222	-0.356	0.127
10	20	0	0.2	0.8	0.5	0.30	0.30	0.30	0.50	0.50	0.25	0.217	-0.356	0.120
10	20	0	0.2	0.8	0.5	0.30	0.30	0.30	0.50	0.50	0.50	0.218	-0.350	0.133
10	20	0	0.5	0.2	0.5	0.00	0.00	0.00	0.00	0.00	0.00	-0.008	0.008	-0.003
10	20	0	0.5	0.2	0.5	0.15	0.15	0.15	0.00	0.00	0.00	0.033	-0.010	0.041
10	20	0	0.5	0.2	0.5	0.15	0.15	0.15	0.00	0.00	0.25	0.028	-0.014	0.037
10	20	0	0.5	0.2	0.5	0.15	0.15	0.15	0.00	0.00	0.50	0.029	-0.005	0.036
10	20	0	0.5	0.2	0.5	0.15	0.15	0.15	0.00	0.25	0.00	0.028	-0.009	0.023
10	20	0	0.5	0.2	0.5	0.15	0.15	0.15	0.00	0.25	0.25	0.034	-0.011	0.029
10	20	0	0.5	0.2	0.5	0.15	0.15	0.15	0.00	0.25	0.50	0.031	-0.009	0.032
10	20	0	0.5	0.2	0.5	0.15	0.15	0.15	0.00	0.50	0.00	0.033	-0.015	0.020
10	20	0	0.5	0.2	0.5	0.15	0.15	0.15	0.00	0.50	0.25	0.034	-0.014	0.017
10	20	0	0.5	0.2	0.5	0.15	0.15	0.15	0.00	0.50	0.50	0.036	-0.014	0.024
10	20	0	0.5	0.2	0.5	0.15	0.15	0.15	0.25	0.00	0.00	0.029	-0.004	0.033
10	20	0	0.5	0.2	0.5	0.15	0.15	0.15	0.25	0.00	0.25	0.033	-0.003	0.038
10	20	0	0.5	0.2	0.5	0.15	0.15	0.15	0.25	0.00	0.50	0.032	-0.001	0.039
10	20	0	0.5	0.2	0.5	0.15	0.15	0.15	0.25	0.25	0.00	0.030	-0.001	0.028
10	20	0	0.5	0.2	0.5	0.15	0.15	0.15	0.25	0.25	0.25	0.033	-0.002	0.031
10	20	0	0.5	0.2	0.5	0.15	0.15	0.15	0.25	0.25	0.50	0.036	-0.002	0.030
10	20	0	0.5	0.2	0.5	0.15	0.15	0.15	0.25	0.50	0.00	0.038	0.000	0.021
10	20	0	0.5	0.2	0.5	0.15	0.15	0.15	0.25	0.50	0.25	0.029	-0.004	0.016
10	20	0	0.5	0.2	0.5	0.15	0.15	0.15	0.25	0.50	0.50	0.033	-0.001	0.018

(continued)

												Mean Bias		
N	m_1	$\frac{m_2}{m_1}$	$E(C)$	$E(R)$	$E(U)$	σ_C	σ_R	σ_U	ρ_{CR}	ρ_{CU}	ρ_{RU}	c	r	u
10	20	0	0.5	0.2	0.5	0.15	0.15	0.15	0.50	0.00	0.00	0.034	0.006	0.038
10	20	0	0.5	0.2	0.5	0.15	0.15	0.15	0.50	0.00	0.25	0.033	0.004	0.042
10	20	0	0.5	0.2	0.5	0.15	0.15	0.15	0.50	0.00	0.50	0.029	0.010	0.039
10	20	0	0.5	0.2	0.5	0.15	0.15	0.15	0.50	0.25	0.00	0.033	0.011	0.028
10	20	0	0.5	0.2	0.5	0.15	0.15	0.15	0.50	0.25	0.25	0.029	0.006	0.027
10	20	0	0.5	0.2	0.5	0.15	0.15	0.15	0.50	0.25	0.50	0.033	0.007	0.027
10	20	0	0.5	0.2	0.5	0.15	0.15	0.15	0.50	0.50	0.00	0.032	0.010	0.017
10	20	0	0.5	0.2	0.5	0.15	0.15	0.15	0.50	0.50	0.25	0.033	0.007	0.020
10	20	0	0.5	0.2	0.5	0.15	0.15	0.15	0.50	0.50	0.50	0.035	0.011	0.019
10	20	0	0.5	0.2	0.5	0.30	0.30	0.30	0.00	0.00	0.00	0.124	-0.037	0.164
10	20	0	0.5	0.2	0.5	0.30	0.30	0.30	0.00	0.00	0.25	0.124	-0.036	0.162
10	20	0	0.5	0.2	0.5	0.30	0.30	0.30	0.00	0.00	0.50	0.127	-0.039	0.162
10	20	0	0.5	0.2	0.5	0.30	0.30	0.30	0.00	0.25	0.00	0.132	-0.044	0.128
10	20	0	0.5	0.2	0.5	0.30	0.30	0.30	0.00	0.25	0.25	0.128	-0.044	0.136
10	20	0	0.5	0.2	0.5	0.30	0.30	0.30	0.00	0.25	0.50	0.133	-0.036	0.134
10	20	0	0.5	0.2	0.5	0.30	0.30	0.30	0.00	0.50	0.00	0.151	-0.050	0.098
10	20	0	0.5	0.2	0.5	0.30	0.30	0.30	0.00	0.50	0.25	0.149	-0.041	0.096
10	20	0	0.5	0.2	0.5	0.30	0.30	0.30	0.00	0.50	0.50	0.145	-0.052	0.090
10	20	0	0.5	0.2	0.5	0.30	0.30	0.30	0.25	0.00	0.00	0.117	-0.014	0.161
10	20	0	0.5	0.2	0.5	0.30	0.30	0.30	0.25	0.00	0.25	0.118	-0.013	0.161
10	20	0	0.5	0.2	0.5	0.30	0.30	0.30	0.25	0.00	0.50	0.116	-0.012	0.159
10	20	0	0.5	0.2	0.5	0.30	0.30	0.30	0.25	0.25	0.00	0.133	-0.017	0.127
10	20	0	0.5	0.2	0.5	0.30	0.30	0.30	0.25	0.25	0.25	0.136	-0.019	0.125
10	20	0	0.5	0.2	0.5	0.30	0.30	0.30	0.25	0.25	0.50	0.137	-0.005	0.134
10	20	0	0.5	0.2	0.5	0.30	0.30	0.30	0.25	0.50	0.00	0.148	-0.020	0.098
10	20	0	0.5	0.2	0.5	0.30	0.30	0.30	0.25	0.50	0.25	0.146	-0.015	0.096
10	20	0	0.5	0.2	0.5	0.30	0.30	0.30	0.25	0.50	0.50	0.154	-0.021	0.092
10	20	0	0.5	0.2	0.5	0.30	0.30	0.30	0.50	0.00	0.00	0.118	0.016	0.163
10	20	0	0.5	0.2	0.5	0.30	0.30	0.30	0.50	0.00	0.25	0.119	0.012	0.166
10	20	0	0.5	0.2	0.5	0.30	0.30	0.30	0.50	0.00	0.50	0.118	0.021	0.165
10	20	0	0.5	0.2	0.5	0.30	0.30	0.30	0.50	0.25	0.00	0.133	0.009	0.131
10	20	0	0.5	0.2	0.5	0.30	0.30	0.30	0.50	0.25	0.25	0.136	0.012	0.134
10	20	0	0.5	0.2	0.5	0.30	0.30	0.30	0.50	0.25	0.50	0.136	0.011	0.137
10	20	0	0.5	0.2	0.5	0.30	0.30	0.30	0.50	0.50	0.00	0.154	0.004	0.100
10	20	0	0.5	0.2	0.5	0.30	0.30	0.30	0.50	0.50	0.25	0.149	0.008	0.096
10	20	0	0.5	0.2	0.5	0.30	0.30	0.30	0.50	0.50	0.50	0.147	0.016	0.098
10	20	0	0.5	0.5	0.5	0.00	0.00	0.00	0.00	0.00	0.00	-0.009	0.014	-0.005
10	20	0	0.5	0.5	0.5	0.15	0.15	0.15	0.00	0.00	0.00	0.031	-0.024	0.036
10	20	0	0.5	0.5	0.5	0.15	0.15	0.15	0.00	0.00	0.25	0.030	-0.024	0.037

(continued)

N	m_1	$\frac{m_2}{m_1}$	$E(C)$	$E(R)$	$E(U)$	σ_C	σ_R	σ_U	ρ_{CR}	ρ_{CU}	ρ_{RU}	Mean Bias c	Mean Bias r	Mean Bias u
10	20	0	0.5	0.5	0.5	0.15	0.15	0.15	0.00	0.00	0.50	0.031	-0.029	0.037
10	20	0	0.5	0.5	0.5	0.15	0.15	0.15	0.00	0.25	0.00	0.032	-0.023	0.031
10	20	0	0.5	0.5	0.5	0.15	0.15	0.15	0.00	0.25	0.25	0.032	-0.028	0.030
10	20	0	0.5	0.5	0.5	0.15	0.15	0.15	0.00	0.25	0.50	0.032	-0.026	0.029
10	20	0	0.5	0.5	0.5	0.15	0.15	0.15	0.00	0.50	0.00	0.035	-0.031	0.019
10	20	0	0.5	0.5	0.5	0.15	0.15	0.15	0.00	0.50	0.25	0.031	-0.022	0.018
10	20	0	0.5	0.5	0.5	0.15	0.15	0.15	0.00	0.50	0.50	0.034	-0.030	0.020
10	20	0	0.5	0.5	0.5	0.15	0.15	0.15	0.25	0.00	0.00	0.035	-0.018	0.040
10	20	0	0.5	0.5	0.5	0.15	0.15	0.15	0.25	0.00	0.25	0.034	-0.018	0.040
10	20	0	0.5	0.5	0.5	0.15	0.15	0.15	0.25	0.00	0.50	0.030	-0.020	0.035
10	20	0	0.5	0.5	0.5	0.15	0.15	0.15	0.25	0.25	0.00	0.037	-0.018	0.029
10	20	0	0.5	0.5	0.5	0.15	0.15	0.15	0.25	0.25	0.25	0.031	-0.017	0.029
10	20	0	0.5	0.5	0.5	0.15	0.15	0.15	0.25	0.25	0.50	0.035	-0.016	0.031
10	20	0	0.5	0.5	0.5	0.15	0.15	0.15	0.25	0.50	0.00	0.031	-0.017	0.016
10	20	0	0.5	0.5	0.5	0.15	0.15	0.15	0.25	0.50	0.25	0.034	-0.017	0.021
10	20	0	0.5	0.5	0.5	0.15	0.15	0.15	0.25	0.50	0.50	0.032	-0.019	0.018
10	20	0	0.5	0.5	0.5	0.15	0.15	0.15	0.50	0.00	0.00	0.030	-0.003	0.037
10	20	0	0.5	0.5	0.5	0.15	0.15	0.15	0.50	0.00	0.25	0.028	0.003	0.035
10	20	0	0.5	0.5	0.5	0.15	0.15	0.15	0.50	0.00	0.50	0.032	0.002	0.036
10	20	0	0.5	0.5	0.5	0.15	0.15	0.15	0.50	0.25	0.00	0.033	-0.005	0.026
10	20	0	0.5	0.5	0.5	0.15	0.15	0.15	0.50	0.25	0.25	0.033	-0.004	0.030
10	20	0	0.5	0.5	0.5	0.15	0.15	0.15	0.50	0.25	0.50	0.031	-0.004	0.028
10	20	0	0.5	0.5	0.5	0.15	0.15	0.15	0.50	0.50	0.00	0.032	-0.012	0.019
10	20	0	0.5	0.5	0.5	0.15	0.15	0.15	0.50	0.50	0.25	0.033	-0.008	0.019
10	20	0	0.5	0.5	0.5	0.15	0.15	0.15	0.50	0.50	0.50	0.032	-0.006	0.015
10	20	0	0.5	0.5	0.5	0.30	0.30	0.30	0.00	0.00	0.00	0.117	-0.100	0.159
10	20	0	0.5	0.5	0.5	0.30	0.30	0.30	0.00	0.00	0.25	0.123	-0.093	0.164
10	20	0	0.5	0.5	0.5	0.30	0.30	0.30	0.00	0.00	0.50	0.128	-0.093	0.159
10	20	0	0.5	0.5	0.5	0.30	0.30	0.30	0.00	0.25	0.00	0.133	-0.115	0.130
10	20	0	0.5	0.5	0.5	0.30	0.30	0.30	0.00	0.25	0.25	0.145	-0.101	0.133
10	20	0	0.5	0.5	0.5	0.30	0.30	0.30	0.00	0.25	0.50	0.131	-0.103	0.137
10	20	0	0.5	0.5	0.5	0.30	0.30	0.30	0.00	0.50	0.00	0.152	-0.114	0.099
10	20	0	0.5	0.5	0.5	0.30	0.30	0.30	0.00	0.50	0.25	0.151	-0.115	0.094
10	20	0	0.5	0.5	0.5	0.30	0.30	0.30	0.00	0.50	0.50	0.150	-0.115	0.099
10	20	0	0.5	0.5	0.5	0.30	0.30	0.30	0.25	0.00	0.00	0.125	-0.064	0.165
10	20	0	0.5	0.5	0.5	0.30	0.30	0.30	0.25	0.00	0.25	0.120	-0.067	0.160
10	20	0	0.5	0.5	0.5	0.30	0.30	0.30	0.25	0.00	0.50	0.121	-0.060	0.163
10	20	0	0.5	0.5	0.5	0.30	0.30	0.30	0.25	0.25	0.00	0.133	-0.078	0.136
10	20	0	0.5	0.5	0.5	0.30	0.30	0.30	0.25	0.25	0.25	0.133	-0.072	0.130

(continued)

N	m_1	$\frac{m_2}{m_1}$	$E(C)$	$E(R)$	$E(U)$	σ_C	σ_R	σ_U	ρ_{CR}	ρ_{CU}	ρ_{RU}	Mean Bias c	Mean Bias r	Mean Bias u
10	20	0	0.5	0.5	0.5	0.30	0.30	0.30	0.25	0.25	0.50	0.134	-0.079	0.133
10	20	0	0.5	0.5	0.5	0.30	0.30	0.30	0.25	0.50	0.00	0.145	-0.088	0.098
10	20	0	0.5	0.5	0.5	0.30	0.30	0.30	0.25	0.50	0.25	0.152	-0.080	0.094
10	20	0	0.5	0.5	0.5	0.30	0.30	0.30	0.25	0.50	0.50	0.143	-0.083	0.092
10	20	0	0.5	0.5	0.5	0.30	0.30	0.30	0.50	0.00	0.00	0.121	-0.031	0.160
10	20	0	0.5	0.5	0.5	0.30	0.30	0.30	0.50	0.00	0.25	0.125	-0.025	0.159
10	20	0	0.5	0.5	0.5	0.30	0.30	0.30	0.50	0.00	0.50	0.123	-0.028	0.163
10	20	0	0.5	0.5	0.5	0.30	0.30	0.30	0.50	0.25	0.00	0.135	-0.041	0.131
10	20	0	0.5	0.5	0.5	0.30	0.30	0.30	0.50	0.25	0.25	0.137	-0.037	0.132
10	20	0	0.5	0.5	0.5	0.30	0.30	0.30	0.50	0.25	0.50	0.132	-0.035	0.136
10	20	0	0.5	0.5	0.5	0.30	0.30	0.30	0.50	0.50	0.00	0.153	-0.043	0.093
10	20	0	0.5	0.5	0.5	0.30	0.30	0.30	0.50	0.50	0.25	0.154	-0.053	0.106
10	20	0	0.5	0.5	0.5	0.30	0.30	0.30	0.50	0.50	0.50	0.152	-0.050	0.094
10	20	0	0.5	0.8	0.5	0.00	0.00	0.00	0.00	0.00	0.00	-0.004	0.016	0.001
10	20	0	0.5	0.8	0.5	0.15	0.15	0.15	0.00	0.00	0.00	0.032	-0.045	0.038
10	20	0	0.5	0.8	0.5	0.15	0.15	0.15	0.00	0.00	0.25	0.034	-0.044	0.037
10	20	0	0.5	0.8	0.5	0.15	0.15	0.15	0.00	0.00	0.50	0.032	-0.043	0.041
10	20	0	0.5	0.8	0.5	0.15	0.15	0.15	0.00	0.25	0.00	0.030	-0.041	0.026
10	20	0	0.5	0.8	0.5	0.15	0.15	0.15	0.00	0.25	0.25	0.032	-0.047	0.029
10	20	0	0.5	0.8	0.5	0.15	0.15	0.15	0.00	0.25	0.50	0.034	-0.038	0.030
10	20	0	0.5	0.8	0.5	0.15	0.15	0.15	0.00	0.50	0.00	0.033	-0.049	0.021
10	20	0	0.5	0.8	0.5	0.15	0.15	0.15	0.00	0.50	0.25	0.035	-0.044	0.020
10	20	0	0.5	0.8	0.5	0.15	0.15	0.15	0.00	0.50	0.50	0.032	-0.048	0.019
10	20	0	0.5	0.8	0.5	0.15	0.15	0.15	0.25	0.00	0.00	0.033	-0.036	0.040
10	20	0	0.5	0.8	0.5	0.15	0.15	0.15	0.25	0.00	0.25	0.030	-0.028	0.038
10	20	0	0.5	0.8	0.5	0.15	0.15	0.15	0.25	0.00	0.50	0.033	-0.034	0.038
10	20	0	0.5	0.8	0.5	0.15	0.15	0.15	0.25	0.25	0.00	0.029	-0.031	0.029
10	20	0	0.5	0.8	0.5	0.15	0.15	0.15	0.25	0.25	0.25	0.035	-0.041	0.034
10	20	0	0.5	0.8	0.5	0.15	0.15	0.15	0.25	0.25	0.50	0.033	-0.030	0.027
10	20	0	0.5	0.8	0.5	0.15	0.15	0.15	0.25	0.50	0.00	0.034	-0.031	0.018
10	20	0	0.5	0.8	0.5	0.15	0.15	0.15	0.25	0.50	0.25	0.036	-0.034	0.021
10	20	0	0.5	0.8	0.5	0.15	0.15	0.15	0.25	0.50	0.50	0.035	-0.041	0.020
10	20	0	0.5	0.8	0.5	0.15	0.15	0.15	0.50	0.00	0.00	0.038	-0.025	0.042
10	20	0	0.5	0.8	0.5	0.15	0.15	0.15	0.50	0.00	0.25	0.031	-0.022	0.040
10	20	0	0.5	0.8	0.5	0.15	0.15	0.15	0.50	0.00	0.50	0.033	-0.026	0.039
10	20	0	0.5	0.8	0.5	0.15	0.15	0.15	0.50	0.25	0.00	0.031	-0.025	0.029
10	20	0	0.5	0.8	0.5	0.15	0.15	0.15	0.50	0.25	0.25	0.032	-0.027	0.028
10	20	0	0.5	0.8	0.5	0.15	0.15	0.15	0.50	0.25	0.50	0.034	-0.027	0.031
10	20	0	0.5	0.8	0.5	0.15	0.15	0.15	0.50	0.50	0.00	0.036	-0.024	0.019

(continued)

												Mean Bias		
N	m_1	$\frac{m_2}{m_1}$	$E(C)$	$E(R)$	$E(U)$	σ_C	σ_R	σ_U	ρ_{CR}	ρ_{CU}	ρ_{RU}	c	r	u
10	20	0	0.5	0.8	0.5	0.15	0.15	0.15	0.50	0.50	0.25	0.037	-0.028	0.023
10	20	0	0.5	0.8	0.5	0.15	0.15	0.15	0.50	0.50	0.50	0.030	-0.022	0.013
10	20	0	0.5	0.8	0.5	0.30	0.30	0.30	0.00	0.00	0.00	0.119	-0.160	0.158
10	20	0	0.5	0.8	0.5	0.30	0.30	0.30	0.00	0.00	0.25	0.120	-0.161	0.161
10	20	0	0.5	0.8	0.5	0.30	0.30	0.30	0.00	0.00	0.50	0.121	-0.146	0.159
10	20	0	0.5	0.8	0.5	0.30	0.30	0.30	0.00	0.25	0.00	0.138	-0.176	0.124
10	20	0	0.5	0.8	0.5	0.30	0.30	0.30	0.00	0.25	0.25	0.136	-0.169	0.136
10	20	0	0.5	0.8	0.5	0.30	0.30	0.30	0.00	0.25	0.50	0.134	-0.178	0.130
10	20	0	0.5	0.8	0.5	0.30	0.30	0.30	0.00	0.50	0.00	0.150	-0.186	0.101
10	20	0	0.5	0.8	0.5	0.30	0.30	0.30	0.00	0.50	0.25	0.155	-0.182	0.105
10	20	0	0.5	0.8	0.5	0.30	0.30	0.30	0.00	0.50	0.50	0.146	-0.181	0.108
10	20	0	0.5	0.8	0.5	0.30	0.30	0.30	0.25	0.00	0.00	0.119	-0.124	0.162
10	20	0	0.5	0.8	0.5	0.30	0.30	0.30	0.25	0.00	0.25	0.126	-0.125	0.168
10	20	0	0.5	0.8	0.5	0.30	0.30	0.30	0.25	0.00	0.50	0.120	-0.125	0.162
10	20	0	0.5	0.8	0.5	0.30	0.30	0.30	0.25	0.25	0.00	0.141	-0.146	0.127
10	20	0	0.5	0.8	0.5	0.30	0.30	0.30	0.25	0.25	0.25	0.141	-0.140	0.138
10	20	0	0.5	0.8	0.5	0.30	0.30	0.30	0.25	0.25	0.50	0.139	-0.145	0.135
10	20	0	0.5	0.8	0.5	0.30	0.30	0.30	0.25	0.50	0.00	0.150	-0.155	0.101
10	20	0	0.5	0.8	0.5	0.30	0.30	0.30	0.25	0.50	0.25	0.146	-0.154	0.091
10	20	0	0.5	0.8	0.5	0.30	0.30	0.30	0.25	0.50	0.50	0.148	-0.156	0.091
10	20	0	0.5	0.8	0.5	0.30	0.30	0.30	0.50	0.00	0.00	0.124	-0.103	0.159
10	20	0	0.5	0.8	0.5	0.30	0.30	0.30	0.50	0.00	0.25	0.115	-0.097	0.156
10	20	0	0.5	0.8	0.5	0.30	0.30	0.30	0.50	0.00	0.50	0.118	-0.094	0.163
10	20	0	0.5	0.8	0.5	0.30	0.30	0.30	0.50	0.25	0.00	0.133	-0.119	0.132
10	20	0	0.5	0.8	0.5	0.30	0.30	0.30	0.50	0.25	0.25	0.138	-0.111	0.135
10	20	0	0.5	0.8	0.5	0.30	0.30	0.30	0.50	0.25	0.50	0.133	-0.112	0.135
10	20	0	0.5	0.8	0.5	0.30	0.30	0.30	0.50	0.50	0.00	0.149	-0.125	0.094
10	20	0	0.5	0.8	0.5	0.30	0.30	0.30	0.50	0.50	0.25	0.152	-0.128	0.100
10	20	0	0.5	0.8	0.5	0.30	0.30	0.30	0.50	0.50	0.50	0.151	-0.128	0.096
10	20	0	0.8	0.2	0.5	0.00	0.00	0.00	0.00	0.00	0.00	-0.009	0.004	-0.003
10	20	0	0.8	0.2	0.5	0.15	0.15	0.15	0.00	0.00	0.00	0.005	-0.001	0.034
10	20	0	0.8	0.2	0.5	0.15	0.15	0.15	0.00	0.00	0.25	0.004	0.002	0.040
10	20	0	0.8	0.2	0.5	0.15	0.15	0.15	0.00	0.00	0.50	0.007	-0.002	0.035
10	20	0	0.8	0.2	0.5	0.15	0.15	0.15	0.00	0.25	0.00	0.008	-0.001	0.013
10	20	0	0.8	0.2	0.5	0.15	0.15	0.15	0.00	0.25	0.25	0.008	-0.002	0.014
10	20	0	0.8	0.2	0.5	0.15	0.15	0.15	0.00	0.25	0.50	0.006	0.000	0.008
10	20	0	0.8	0.2	0.5	0.15	0.15	0.15	0.00	0.50	0.00	0.007	0.003	-0.014
10	20	0	0.8	0.2	0.5	0.15	0.15	0.15	0.00	0.50	0.25	0.007	-0.002	-0.012
10	20	0	0.8	0.2	0.5	0.15	0.15	0.15	0.00	0.50	0.50	0.008	0.002	-0.016

(continued)

												Mean Bias		
N	m_1	$\frac{m_2}{m_1}$	$E(C)$	$E(R)$	$E(U)$	σ_C	σ_R	σ_U	ρ_{CR}	ρ_{CU}	ρ_{RU}	c	r	u
10	20	0	0.8	0.2	0.5	0.15	0.15	0.15	0.25	0.00	0.00	0.006	0.006	0.033
10	20	0	0.8	0.2	0.5	0.15	0.15	0.15	0.25	0.00	0.25	0.007	0.004	0.037
10	20	0	0.8	0.2	0.5	0.15	0.15	0.15	0.25	0.00	0.50	0.006	0.007	0.030
10	20	0	0.8	0.2	0.5	0.15	0.15	0.15	0.25	0.25	0.00	0.007	0.005	0.013
10	20	0	0.8	0.2	0.5	0.15	0.15	0.15	0.25	0.25	0.25	0.009	0.005	0.013
10	20	0	0.8	0.2	0.5	0.15	0.15	0.15	0.25	0.25	0.50	0.006	0.009	0.009
10	20	0	0.8	0.2	0.5	0.15	0.15	0.15	0.25	0.50	0.00	0.005	0.008	-0.018
10	20	0	0.8	0.2	0.5	0.15	0.15	0.15	0.25	0.50	0.25	0.008	0.006	-0.019
10	20	0	0.8	0.2	0.5	0.15	0.15	0.15	0.25	0.50	0.50	0.008	0.003	-0.013
10	20	0	0.8	0.2	0.5	0.15	0.15	0.15	0.50	0.00	0.00	0.004	0.010	0.035
10	20	0	0.8	0.2	0.5	0.15	0.15	0.15	0.50	0.00	0.25	0.006	0.011	0.030
10	20	0	0.8	0.2	0.5	0.15	0.15	0.15	0.50	0.00	0.50	0.007	0.012	0.034
10	20	0	0.8	0.2	0.5	0.15	0.15	0.15	0.50	0.25	0.00	0.010	0.009	0.008
10	20	0	0.8	0.2	0.5	0.15	0.15	0.15	0.50	0.25	0.25	0.003	0.010	0.008
10	20	0	0.8	0.2	0.5	0.15	0.15	0.15	0.50	0.25	0.50	0.009	0.009	0.012
10	20	0	0.8	0.2	0.5	0.15	0.15	0.15	0.50	0.50	0.00	0.007	0.009	-0.013
10	20	0	0.8	0.2	0.5	0.15	0.15	0.15	0.50	0.50	0.25	0.007	0.015	-0.017
10	20	0	0.8	0.2	0.5	0.15	0.15	0.15	0.50	0.50	0.50	0.005	0.009	-0.016
10	20	0	0.8	0.2	0.5	0.30	0.30	0.30	0.00	0.00	0.00	0.019	0.011	0.126
10	20	0	0.8	0.2	0.5	0.30	0.30	0.30	0.00	0.00	0.25	0.026	0.003	0.129
10	20	0	0.8	0.2	0.5	0.30	0.30	0.30	0.00	0.00	0.50	0.021	0.005	0.133
10	20	0	0.8	0.2	0.5	0.30	0.30	0.30	0.00	0.25	0.00	0.024	0.020	0.055
10	20	0	0.8	0.2	0.5	0.30	0.30	0.30	0.00	0.25	0.25	0.021	0.018	0.054
10	20	0	0.8	0.2	0.5	0.30	0.30	0.30	0.00	0.25	0.50	0.019	0.017	0.054
10	20	0	0.8	0.2	0.5	0.30	0.30	0.30	0.00	0.50	0.00	0.005	0.049	-0.016
10	20	0	0.8	0.2	0.5	0.30	0.30	0.30	0.00	0.50	0.25	0.009	0.041	-0.023
10	20	0	0.8	0.2	0.5	0.30	0.30	0.30	0.00	0.50	0.50	0.008	0.040	-0.019
10	20	0	0.8	0.2	0.5	0.30	0.30	0.30	0.25	0.00	0.00	0.027	0.020	0.130
10	20	0	0.8	0.2	0.5	0.30	0.30	0.30	0.25	0.00	0.25	0.022	0.019	0.123
10	20	0	0.8	0.2	0.5	0.30	0.30	0.30	0.25	0.00	0.50	0.028	0.022	0.126
10	20	0	0.8	0.2	0.5	0.30	0.30	0.30	0.25	0.25	0.00	0.027	0.029	0.063
10	20	0	0.8	0.2	0.5	0.30	0.30	0.30	0.25	0.25	0.25	0.026	0.039	0.067
10	20	0	0.8	0.2	0.5	0.30	0.30	0.30	0.25	0.25	0.50	0.028	0.029	0.056
10	20	0	0.8	0.2	0.5	0.30	0.30	0.30	0.25	0.50	0.00	0.007	0.061	-0.023
10	20	0	0.8	0.2	0.5	0.30	0.30	0.30	0.25	0.50	0.25	0.016	0.052	-0.014
10	20	0	0.8	0.2	0.5	0.30	0.30	0.30	0.25	0.50	0.50	0.017	0.057	-0.024
10	20	0	0.8	0.2	0.5	0.30	0.30	0.30	0.50	0.00	0.00	0.026	0.043	0.120
10	20	0	0.8	0.2	0.5	0.30	0.30	0.30	0.50	0.00	0.25	0.018	0.032	0.134
10	20	0	0.8	0.2	0.5	0.30	0.30	0.30	0.50	0.00	0.50	0.026	0.050	0.130

(continued)

												Mean Bias		
N	m_1	$\frac{m_2}{m_1}$	$E(C)$	$E(R)$	$E(U)$	σ_C	σ_R	σ_U	ρ_{CR}	ρ_{CU}	ρ_{RU}	c	r	u
10	20	0	0.8	0.2	0.5	0.30	0.30	0.30	0.50	0.25	0.00	0.026	0.039	0.054
10	20	0	0.8	0.2	0.5	0.30	0.30	0.30	0.50	0.25	0.25	0.036	0.045	0.071
10	20	0	0.8	0.2	0.5	0.30	0.30	0.30	0.50	0.25	0.50	0.028	0.048	0.063
10	20	0	0.8	0.2	0.5	0.30	0.30	0.30	0.50	0.50	0.00	0.018	0.065	-0.018
10	20	0	0.8	0.2	0.5	0.30	0.30	0.30	0.50	0.50	0.25	0.010	0.074	-0.034
10	20	0	0.8	0.2	0.5	0.30	0.30	0.30	0.50	0.50	0.50	0.020	0.060	-0.024
10	20	0	0.8	0.5	0.5	0.00	0.00	0.00	0.00	0.00	0.00	-0.009	0.010	-0.003
10	20	0	0.8	0.5	0.5	0.15	0.15	0.15	0.00	0.00	0.00	0.009	-0.007	0.031
10	20	0	0.8	0.5	0.5	0.15	0.15	0.15	0.00	0.00	0.25	0.007	-0.003	0.031
10	20	0	0.8	0.5	0.5	0.15	0.15	0.15	0.00	0.00	0.50	0.006	0.000	0.030
10	20	0	0.8	0.5	0.5	0.15	0.15	0.15	0.00	0.25	0.00	0.005	-0.001	0.005
10	20	0	0.8	0.5	0.5	0.15	0.15	0.15	0.00	0.25	0.25	0.008	-0.004	0.009
10	20	0	0.8	0.5	0.5	0.15	0.15	0.15	0.00	0.25	0.50	0.007	-0.004	0.008
10	20	0	0.8	0.5	0.5	0.15	0.15	0.15	0.00	0.50	0.00	0.007	-0.005	-0.010
10	20	0	0.8	0.5	0.5	0.15	0.15	0.15	0.00	0.50	0.25	0.005	0.003	-0.015
10	20	0	0.8	0.5	0.5	0.15	0.15	0.15	0.00	0.50	0.50	0.007	-0.003	-0.014
10	20	0	0.8	0.5	0.5	0.15	0.15	0.15	0.25	0.00	0.00	0.008	0.002	0.033
10	20	0	0.8	0.5	0.5	0.15	0.15	0.15	0.25	0.00	0.25	0.007	0.000	0.033
10	20	0	0.8	0.5	0.5	0.15	0.15	0.15	0.25	0.00	0.50	0.007	0.003	0.032
10	20	0	0.8	0.5	0.5	0.15	0.15	0.15	0.25	0.25	0.00	0.006	0.001	0.012
10	20	0	0.8	0.5	0.5	0.15	0.15	0.15	0.25	0.25	0.25	0.011	0.003	0.015
10	20	0	0.8	0.5	0.5	0.15	0.15	0.15	0.25	0.25	0.50	0.008	0.003	0.012
10	20	0	0.8	0.5	0.5	0.15	0.15	0.15	0.25	0.50	0.00	0.008	0.004	-0.015
10	20	0	0.8	0.5	0.5	0.15	0.15	0.15	0.25	0.50	0.25	0.006	0.002	-0.012
10	20	0	0.8	0.5	0.5	0.15	0.15	0.15	0.25	0.50	0.50	0.002	0.006	-0.016
10	20	0	0.8	0.5	0.5	0.15	0.15	0.15	0.50	0.00	0.00	0.007	0.008	0.032
10	20	0	0.8	0.5	0.5	0.15	0.15	0.15	0.50	0.00	0.25	0.006	0.012	0.031
10	20	0	0.8	0.5	0.5	0.15	0.15	0.15	0.50	0.00	0.50	0.008	0.012	0.036
10	20	0	0.8	0.5	0.5	0.15	0.15	0.15	0.50	0.25	0.00	0.006	0.010	0.015
10	20	0	0.8	0.5	0.5	0.15	0.15	0.15	0.50	0.25	0.25	0.008	0.012	0.014
10	20	0	0.8	0.5	0.5	0.15	0.15	0.15	0.50	0.25	0.50	0.007	0.008	0.011
10	20	0	0.8	0.5	0.5	0.15	0.15	0.15	0.50	0.50	0.00	0.006	0.010	-0.012
10	20	0	0.8	0.5	0.5	0.15	0.15	0.15	0.50	0.50	0.25	0.006	0.012	-0.016
10	20	0	0.8	0.5	0.5	0.15	0.15	0.15	0.50	0.50	0.50	0.012	0.009	-0.008
10	20	0	0.8	0.5	0.5	0.30	0.30	0.30	0.00	0.00	0.00	0.023	-0.006	0.125
10	20	0	0.8	0.5	0.5	0.30	0.30	0.30	0.00	0.00	0.25	0.029	-0.015	0.135
10	20	0	0.8	0.5	0.5	0.30	0.30	0.30	0.00	0.00	0.50	0.031	-0.012	0.113
10	20	0	0.8	0.5	0.5	0.30	0.30	0.30	0.00	0.25	0.00	0.024	-0.003	0.052
10	20	0	0.8	0.5	0.5	0.30	0.30	0.30	0.00	0.25	0.25	0.025	-0.010	0.059

(continued)

N	m_1	$\frac{m_2}{m_1}$	$E(C)$	$E(R)$	$E(U)$	σ_C	σ_R	σ_U	ρ_{CR}	ρ_{CU}	ρ_{RU}	Mean Bias c	Mean Bias r	Mean Bias u
10	20	0	0.8	0.5	0.5	0.30	0.30	0.30	0.00	0.25	0.50	0.028	-0.003	0.060
10	20	0	0.8	0.5	0.5	0.30	0.30	0.30	0.00	0.50	0.00	0.024	0.004	-0.032
10	20	0	0.8	0.5	0.5	0.30	0.30	0.30	0.00	0.50	0.25	0.034	-0.005	-0.023
10	20	0	0.8	0.5	0.5	0.30	0.30	0.30	0.00	0.50	0.50	0.023	0.002	-0.035
10	20	0	0.8	0.5	0.5	0.30	0.30	0.30	0.25	0.00	0.00	0.025	0.010	0.121
10	20	0	0.8	0.5	0.5	0.30	0.30	0.30	0.25	0.00	0.25	0.030	0.008	0.128
10	20	0	0.8	0.5	0.5	0.30	0.30	0.30	0.25	0.00	0.50	0.028	0.012	0.125
10	20	0	0.8	0.5	0.5	0.30	0.30	0.30	0.25	0.25	0.00	0.034	0.007	0.051
10	20	0	0.8	0.5	0.5	0.30	0.30	0.30	0.25	0.25	0.25	0.031	0.011	0.046
10	20	0	0.8	0.5	0.5	0.30	0.30	0.30	0.25	0.25	0.50	0.036	0.013	0.059
10	20	0	0.8	0.5	0.5	0.30	0.30	0.30	0.25	0.50	0.00	0.027	0.023	-0.032
10	20	0	0.8	0.5	0.5	0.30	0.30	0.30	0.25	0.50	0.25	0.032	0.019	-0.019
10	20	0	0.8	0.5	0.5	0.30	0.30	0.30	0.25	0.50	0.50	0.029	0.024	-0.019
10	20	0	0.8	0.5	0.5	0.30	0.30	0.30	0.50	0.00	0.00	0.032	0.023	0.125
10	20	0	0.8	0.5	0.5	0.30	0.30	0.30	0.50	0.00	0.25	0.028	0.021	0.128
10	20	0	0.8	0.5	0.5	0.30	0.30	0.30	0.50	0.00	0.50	0.027	0.026	0.123
10	20	0	0.8	0.5	0.5	0.30	0.30	0.30	0.50	0.25	0.00	0.031	0.032	0.057
10	20	0	0.8	0.5	0.5	0.30	0.30	0.30	0.50	0.25	0.25	0.036	0.026	0.062
10	20	0	0.8	0.5	0.5	0.30	0.30	0.30	0.50	0.25	0.50	0.037	0.032	0.059
10	20	0	0.8	0.5	0.5	0.30	0.30	0.30	0.50	0.50	0.00	0.033	0.042	-0.022
10	20	0	0.8	0.5	0.5	0.30	0.30	0.30	0.50	0.50	0.25	0.023	0.049	-0.042
10	20	0	0.8	0.5	0.5	0.30	0.30	0.30	0.50	0.50	0.50	0.030	0.042	-0.029
10	20	0	0.8	0.8	0.5	0.00	0.00	0.00	0.00	0.00	0.00	-0.005	0.009	-0.005
10	20	0	0.8	0.8	0.5	0.15	0.15	0.15	0.00	0.00	0.00	0.007	-0.005	0.031
10	20	0	0.8	0.8	0.5	0.15	0.15	0.15	0.00	0.00	0.25	0.006	-0.004	0.031
10	20	0	0.8	0.8	0.5	0.15	0.15	0.15	0.00	0.00	0.50	0.005	-0.005	0.031
10	20	0	0.8	0.8	0.5	0.15	0.15	0.15	0.00	0.25	0.00	0.008	-0.005	0.009
10	20	0	0.8	0.8	0.5	0.15	0.15	0.15	0.00	0.25	0.25	0.009	-0.008	0.006
10	20	0	0.8	0.8	0.5	0.15	0.15	0.15	0.00	0.25	0.50	0.009	-0.005	0.011
10	20	0	0.8	0.8	0.5	0.15	0.15	0.15	0.00	0.50	0.00	0.008	-0.006	-0.013
10	20	0	0.8	0.8	0.5	0.15	0.15	0.15	0.00	0.50	0.25	0.006	-0.003	-0.014
10	20	0	0.8	0.8	0.5	0.15	0.15	0.15	0.00	0.50	0.50	0.006	-0.004	-0.016
10	20	0	0.8	0.8	0.5	0.15	0.15	0.15	0.25	0.00	0.00	0.007	-0.001	0.034
10	20	0	0.8	0.8	0.5	0.15	0.15	0.15	0.25	0.00	0.25	0.005	-0.001	0.036
10	20	0	0.8	0.8	0.5	0.15	0.15	0.15	0.25	0.00	0.50	0.006	0.002	0.034
10	20	0	0.8	0.8	0.5	0.15	0.15	0.15	0.25	0.25	0.00	0.005	0.000	0.010
10	20	0	0.8	0.8	0.5	0.15	0.15	0.15	0.25	0.25	0.25	0.007	0.002	0.007
10	20	0	0.8	0.8	0.5	0.15	0.15	0.15	0.25	0.25	0.50	0.010	0.000	0.012
10	20	0	0.8	0.8	0.5	0.15	0.15	0.15	0.25	0.50	0.00	0.006	-0.001	-0.010

(continued)

												Mean Bias		
N	m_1	$\frac{m_2}{m_1}$	$E(C)$	$E(R)$	$E(U)$	σ_C	σ_R	σ_U	ρ_{CR}	ρ_{CU}	ρ_{RU}	c	r	u
10	20	0	0.8	0.8	0.5	0.15	0.15	0.15	0.25	0.50	0.25	0.008	0.002	-0.009
10	20	0	0.8	0.8	0.5	0.15	0.15	0.15	0.25	0.50	0.50	0.008	0.001	-0.014
10	20	0	0.8	0.8	0.5	0.15	0.15	0.15	0.50	0.00	0.00	0.006	0.003	0.026
10	20	0	0.8	0.8	0.5	0.15	0.15	0.15	0.50	0.00	0.25	0.008	0.009	0.033
10	20	0	0.8	0.8	0.5	0.15	0.15	0.15	0.50	0.00	0.50	0.005	0.011	0.033
10	20	0	0.8	0.8	0.5	0.15	0.15	0.15	0.50	0.25	0.00	0.008	0.004	0.011
10	20	0	0.8	0.8	0.5	0.15	0.15	0.15	0.50	0.25	0.25	0.006	0.006	0.015
10	20	0	0.8	0.8	0.5	0.15	0.15	0.15	0.50	0.25	0.50	0.008	0.003	0.009
10	20	0	0.8	0.8	0.5	0.15	0.15	0.15	0.50	0.50	0.00	0.009	0.005	-0.009
10	20	0	0.8	0.8	0.5	0.15	0.15	0.15	0.50	0.50	0.25	0.007	0.006	-0.014
10	20	0	0.8	0.8	0.5	0.15	0.15	0.15	0.50	0.50	0.50	0.009	0.005	-0.009
10	20	0	0.8	0.8	0.5	0.30	0.30	0.30	0.00	0.00	0.00	0.038	-0.030	0.131
10	20	0	0.8	0.8	0.5	0.30	0.30	0.30	0.00	0.00	0.25	0.034	-0.031	0.122
10	20	0	0.8	0.8	0.5	0.30	0.30	0.30	0.00	0.00	0.50	0.040	-0.035	0.127
10	20	0	0.8	0.8	0.5	0.30	0.30	0.30	0.00	0.25	0.00	0.042	-0.041	0.057
10	20	0	0.8	0.8	0.5	0.30	0.30	0.30	0.00	0.25	0.25	0.043	-0.040	0.060
10	20	0	0.8	0.8	0.5	0.30	0.30	0.30	0.00	0.25	0.50	0.041	-0.035	0.049
10	20	0	0.8	0.8	0.5	0.30	0.30	0.30	0.00	0.50	0.00	0.048	-0.040	-0.010
10	20	0	0.8	0.8	0.5	0.30	0.30	0.30	0.00	0.50	0.25	0.048	-0.037	-0.014
10	20	0	0.8	0.8	0.5	0.30	0.30	0.30	0.00	0.50	0.50	0.041	-0.035	-0.023
10	20	0	0.8	0.8	0.5	0.30	0.30	0.30	0.25	0.00	0.00	0.035	-0.017	0.118
10	20	0	0.8	0.8	0.5	0.30	0.30	0.30	0.25	0.00	0.25	0.031	-0.019	0.131
10	20	0	0.8	0.8	0.5	0.30	0.30	0.30	0.25	0.00	0.50	0.038	-0.015	0.126
10	20	0	0.8	0.8	0.5	0.30	0.30	0.30	0.25	0.25	0.00	0.041	-0.022	0.051
10	20	0	0.8	0.8	0.5	0.30	0.30	0.30	0.25	0.25	0.25	0.040	-0.023	0.051
10	20	0	0.8	0.8	0.5	0.30	0.30	0.30	0.25	0.25	0.50	0.046	-0.020	0.067
10	20	0	0.8	0.8	0.5	0.30	0.30	0.30	0.25	0.50	0.00	0.052	-0.019	-0.023
10	20	0	0.8	0.8	0.5	0.30	0.30	0.30	0.25	0.50	0.25	0.049	-0.023	-0.017
10	20	0	0.8	0.8	0.5	0.30	0.30	0.30	0.25	0.50	0.50	0.047	-0.017	-0.024
10	20	0	0.8	0.8	0.5	0.30	0.30	0.30	0.50	0.00	0.00	0.036	0.001	0.133
10	20	0	0.8	0.8	0.5	0.30	0.30	0.30	0.50	0.00	0.25	0.038	0.008	0.123
10	20	0	0.8	0.8	0.5	0.30	0.30	0.30	0.50	0.00	0.50	0.035	0.007	0.125
10	20	0	0.8	0.8	0.5	0.30	0.30	0.30	0.50	0.25	0.00	0.045	0.006	0.044
10	20	0	0.8	0.8	0.5	0.30	0.30	0.30	0.50	0.25	0.25	0.046	0.006	0.060
10	20	0	0.8	0.8	0.5	0.30	0.30	0.30	0.50	0.25	0.50	0.050	0.001	0.074
10	20	0	0.8	0.8	0.5	0.30	0.30	0.30	0.50	0.50	0.00	0.050	-0.007	-0.024
10	20	0	0.8	0.8	0.5	0.30	0.30	0.30	0.50	0.50	0.25	0.051	-0.005	-0.019
10	20	0	0.8	0.8	0.5	0.30	0.30	0.30	0.50	0.50	0.50	0.050	0.005	-0.022
10	20	1	0.2	0.2	0.5	0.00	0.00	0.00	0.00	0.00	0.00	0.001	0.019	0.000

(continued)

N	m_1	$\frac{m_2}{m_1}$	$E(C)$	$E(R)$	$E(U)$	σ_C	σ_R	σ_U	ρ_{CR}	ρ_{CU}	ρ_{RU}	Mean Bias c	r	u
10	20	1	0.2	0.2	0.5	0.15	0.15	0.15	0.00	0.00	0.00	0.033	-0.014	0.011
10	20	1	0.2	0.2	0.5	0.15	0.15	0.15	0.00	0.00	0.25	0.039	-0.026	0.015
10	20	1	0.2	0.2	0.5	0.15	0.15	0.15	0.00	0.00	0.50	0.031	-0.021	0.012
10	20	1	0.2	0.2	0.5	0.15	0.15	0.15	0.00	0.25	0.00	0.036	-0.019	0.013
10	20	1	0.2	0.2	0.5	0.15	0.15	0.15	0.00	0.25	0.25	0.040	-0.021	0.016
10	20	1	0.2	0.2	0.5	0.15	0.15	0.15	0.00	0.25	0.50	0.036	-0.023	0.011
10	20	1	0.2	0.2	0.5	0.15	0.15	0.15	0.00	0.50	0.00	0.042	-0.023	0.010
10	20	1	0.2	0.2	0.5	0.15	0.15	0.15	0.00	0.50	0.25	0.043	-0.024	0.010
10	20	1	0.2	0.2	0.5	0.15	0.15	0.15	0.00	0.50	0.50	0.046	-0.027	0.010
10	20	1	0.2	0.2	0.5	0.15	0.15	0.15	0.25	0.00	0.00	0.034	-0.001	0.016
10	20	1	0.2	0.2	0.5	0.15	0.15	0.15	0.25	0.00	0.25	0.032	0.005	0.015
10	20	1	0.2	0.2	0.5	0.15	0.15	0.15	0.25	0.00	0.50	0.039	-0.003	0.015
10	20	1	0.2	0.2	0.5	0.15	0.15	0.15	0.25	0.25	0.00	0.040	-0.003	0.013
10	20	1	0.2	0.2	0.5	0.15	0.15	0.15	0.25	0.25	0.25	0.038	-0.007	0.012
10	20	1	0.2	0.2	0.5	0.15	0.15	0.15	0.25	0.25	0.50	0.037	-0.008	0.010
10	20	1	0.2	0.2	0.5	0.15	0.15	0.15	0.25	0.50	0.00	0.042	-0.004	0.007
10	20	1	0.2	0.2	0.5	0.15	0.15	0.15	0.25	0.50	0.25	0.045	-0.007	0.011
10	20	1	0.2	0.2	0.5	0.15	0.15	0.15	0.25	0.50	0.50	0.046	-0.011	0.011
10	20	1	0.2	0.2	0.5	0.15	0.15	0.15	0.50	0.00	0.00	0.039	0.026	0.016
10	20	1	0.2	0.2	0.5	0.15	0.15	0.15	0.50	0.00	0.25	0.030	0.024	0.013
10	20	1	0.2	0.2	0.5	0.15	0.15	0.15	0.50	0.00	0.50	0.033	0.020	0.016
10	20	1	0.2	0.2	0.5	0.15	0.15	0.15	0.50	0.25	0.00	0.039	0.016	0.014
10	20	1	0.2	0.2	0.5	0.15	0.15	0.15	0.50	0.25	0.25	0.037	0.020	0.013
10	20	1	0.2	0.2	0.5	0.15	0.15	0.15	0.50	0.25	0.50	0.035	0.023	0.014
10	20	1	0.2	0.2	0.5	0.15	0.15	0.15	0.50	0.50	0.00	0.041	0.025	0.009
10	20	1	0.2	0.2	0.5	0.15	0.15	0.15	0.50	0.50	0.25	0.043	0.010	0.008
10	20	1	0.2	0.2	0.5	0.15	0.15	0.15	0.50	0.50	0.50	0.039	0.016	0.011
10	20	1	0.2	0.2	0.5	0.30	0.30	0.30	0.00	0.00	0.00	0.142	-0.081	0.053
10	20	1	0.2	0.2	0.5	0.30	0.30	0.30	0.00	0.00	0.25	0.135	-0.082	0.060
10	20	1	0.2	0.2	0.5	0.30	0.30	0.30	0.00	0.00	0.50	0.133	-0.086	0.056
10	20	1	0.2	0.2	0.5	0.30	0.30	0.30	0.00	0.25	0.00	0.150	-0.086	0.054
10	20	1	0.2	0.2	0.5	0.30	0.30	0.30	0.00	0.25	0.25	0.150	-0.088	0.050
10	20	1	0.2	0.2	0.5	0.30	0.30	0.30	0.00	0.25	0.50	0.154	-0.088	0.052
10	20	1	0.2	0.2	0.5	0.30	0.30	0.30	0.00	0.50	0.00	0.166	-0.088	0.045
10	20	1	0.2	0.2	0.5	0.30	0.30	0.30	0.00	0.50	0.25	0.168	-0.096	0.042
10	20	1	0.2	0.2	0.5	0.30	0.30	0.30	0.00	0.50	0.50	0.171	-0.097	0.041
10	20	1	0.2	0.2	0.5	0.30	0.30	0.30	0.25	0.00	0.00	0.136	-0.033	0.055
10	20	1	0.2	0.2	0.5	0.30	0.30	0.30	0.25	0.00	0.25	0.142	-0.040	0.056
10	20	1	0.2	0.2	0.5	0.30	0.30	0.30	0.25	0.00	0.50	0.142	-0.041	0.058

(continued)

												Mean Bias		
N	m_1	$\frac{m_2}{m_1}$	$E(C)$	$E(R)$	$E(U)$	σ_C	σ_R	σ_U	ρ_{CR}	ρ_{CU}	ρ_{RU}	c	r	u
10	20	1	0.2	0.2	0.5	0.30	0.30	0.30	0.25	0.25	0.00	0.157	-0.045	0.054
10	20	1	0.2	0.2	0.5	0.30	0.30	0.30	0.25	0.25	0.25	0.153	-0.039	0.047
10	20	1	0.2	0.2	0.5	0.30	0.30	0.30	0.25	0.25	0.50	0.156	-0.041	0.050
10	20	1	0.2	0.2	0.5	0.30	0.30	0.30	0.25	0.50	0.00	0.161	-0.052	0.043
10	20	1	0.2	0.2	0.5	0.30	0.30	0.30	0.25	0.50	0.25	0.164	-0.054	0.043
10	20	1	0.2	0.2	0.5	0.30	0.30	0.30	0.25	0.50	0.50	0.165	-0.050	0.044
10	20	1	0.2	0.2	0.5	0.30	0.30	0.30	0.50	0.00	0.00	0.138	0.021	0.054
10	20	1	0.2	0.2	0.5	0.30	0.30	0.30	0.50	0.00	0.25	0.141	0.025	0.059
10	20	1	0.2	0.2	0.5	0.30	0.30	0.30	0.50	0.00	0.50	0.135	0.021	0.058
10	20	1	0.2	0.2	0.5	0.30	0.30	0.30	0.50	0.25	0.00	0.154	0.016	0.049
10	20	1	0.2	0.2	0.5	0.30	0.30	0.30	0.50	0.25	0.25	0.154	0.007	0.045
10	20	1	0.2	0.2	0.5	0.30	0.30	0.30	0.50	0.25	0.50	0.160	0.017	0.053
10	20	1	0.2	0.2	0.5	0.30	0.30	0.30	0.50	0.50	0.00	0.172	-0.001	0.043
10	20	1	0.2	0.2	0.5	0.30	0.30	0.30	0.50	0.50	0.25	0.164	0.002	0.044
10	20	1	0.2	0.2	0.5	0.30	0.30	0.30	0.50	0.50	0.50	0.168	-0.001	0.044
10	20	1	0.2	0.5	0.5	0.00	0.00	0.00	0.00	0.00	0.00	0.001	0.024	0.002
10	20	1	0.2	0.5	0.5	0.15	0.15	0.15	0.00	0.00	0.00	0.042	-0.058	0.016
10	20	1	0.2	0.5	0.5	0.15	0.15	0.15	0.00	0.00	0.25	0.035	-0.057	0.015
10	20	1	0.2	0.5	0.5	0.15	0.15	0.15	0.00	0.00	0.50	0.035	-0.047	0.015
10	20	1	0.2	0.5	0.5	0.15	0.15	0.15	0.00	0.25	0.00	0.037	-0.054	0.010
10	20	1	0.2	0.5	0.5	0.15	0.15	0.15	0.00	0.25	0.25	0.041	-0.063	0.010
10	20	1	0.2	0.5	0.5	0.15	0.15	0.15	0.00	0.25	0.50	0.039	-0.063	0.010
10	20	1	0.2	0.5	0.5	0.15	0.15	0.15	0.00	0.50	0.00	0.043	-0.072	0.010
10	20	1	0.2	0.5	0.5	0.15	0.15	0.15	0.00	0.50	0.25	0.043	-0.067	0.010
10	20	1	0.2	0.5	0.5	0.15	0.15	0.15	0.00	0.50	0.50	0.043	-0.064	0.009
10	20	1	0.2	0.5	0.5	0.15	0.15	0.15	0.25	0.00	0.00	0.032	-0.036	0.015
10	20	1	0.2	0.5	0.5	0.15	0.15	0.15	0.25	0.00	0.25	0.034	-0.029	0.013
10	20	1	0.2	0.5	0.5	0.15	0.15	0.15	0.25	0.00	0.50	0.034	-0.036	0.011
10	20	1	0.2	0.5	0.5	0.15	0.15	0.15	0.25	0.25	0.00	0.041	-0.039	0.009
10	20	1	0.2	0.5	0.5	0.15	0.15	0.15	0.25	0.25	0.25	0.039	-0.049	0.012
10	20	1	0.2	0.5	0.5	0.15	0.15	0.15	0.25	0.25	0.50	0.042	-0.050	0.015
10	20	1	0.2	0.5	0.5	0.15	0.15	0.15	0.25	0.50	0.00	0.041	-0.051	0.009
10	20	1	0.2	0.5	0.5	0.15	0.15	0.15	0.25	0.50	0.25	0.043	-0.046	0.012
10	20	1	0.2	0.5	0.5	0.15	0.15	0.15	0.25	0.50	0.50	0.044	-0.047	0.010
10	20	1	0.2	0.5	0.5	0.15	0.15	0.15	0.50	0.00	0.00	0.037	-0.011	0.016
10	20	1	0.2	0.5	0.5	0.15	0.15	0.15	0.50	0.00	0.25	0.031	-0.005	0.016
10	20	1	0.2	0.5	0.5	0.15	0.15	0.15	0.50	0.00	0.50	0.033	-0.010	0.013
10	20	1	0.2	0.5	0.5	0.15	0.15	0.15	0.50	0.25	0.00	0.035	-0.019	0.013
10	20	1	0.2	0.5	0.5	0.15	0.15	0.15	0.50	0.25	0.25	0.040	-0.022	0.011

(continued)

												Mean Bias		
N	m_1	$\frac{m_2}{m_1}$	$E(C)$	$E(R)$	$E(U)$	σ_C	σ_R	σ_U	ρ_{CR}	ρ_{CU}	ρ_{RU}	c	r	u
10	20	1	0.2	0.5	0.5	0.15	0.15	0.15	0.50	0.25	0.50	0.037	-0.021	0.011
10	20	1	0.2	0.5	0.5	0.15	0.15	0.15	0.50	0.50	0.00	0.044	-0.032	0.009
10	20	1	0.2	0.5	0.5	0.15	0.15	0.15	0.50	0.50	0.25	0.042	-0.033	0.008
10	20	1	0.2	0.5	0.5	0.15	0.15	0.15	0.50	0.50	0.50	0.043	-0.024	0.010
10	20	1	0.2	0.5	0.5	0.30	0.30	0.30	0.00	0.00	0.00	0.134	-0.204	0.061
10	20	1	0.2	0.5	0.5	0.30	0.30	0.30	0.00	0.00	0.25	0.136	-0.207	0.059
10	20	1	0.2	0.5	0.5	0.30	0.30	0.30	0.00	0.00	0.50	0.129	-0.212	0.063
10	20	1	0.2	0.5	0.5	0.30	0.30	0.30	0.00	0.25	0.00	0.149	-0.231	0.049
10	20	1	0.2	0.5	0.5	0.30	0.30	0.30	0.00	0.25	0.25	0.157	-0.221	0.055
10	20	1	0.2	0.5	0.5	0.30	0.30	0.30	0.00	0.25	0.50	0.157	-0.223	0.053
10	20	1	0.2	0.5	0.5	0.30	0.30	0.30	0.00	0.50	0.00	0.162	-0.235	0.046
10	20	1	0.2	0.5	0.5	0.30	0.30	0.30	0.00	0.50	0.25	0.160	-0.229	0.046
10	20	1	0.2	0.5	0.5	0.30	0.30	0.30	0.00	0.50	0.50	0.170	-0.228	0.050
10	20	1	0.2	0.5	0.5	0.30	0.30	0.30	0.25	0.00	0.00	0.141	-0.159	0.054
10	20	1	0.2	0.5	0.5	0.30	0.30	0.30	0.25	0.00	0.25	0.136	-0.154	0.060
10	20	1	0.2	0.5	0.5	0.30	0.30	0.30	0.25	0.00	0.50	0.133	-0.159	0.060
10	20	1	0.2	0.5	0.5	0.30	0.30	0.30	0.25	0.25	0.00	0.151	-0.167	0.051
10	20	1	0.2	0.5	0.5	0.30	0.30	0.30	0.25	0.25	0.25	0.153	-0.174	0.049
10	20	1	0.2	0.5	0.5	0.30	0.30	0.30	0.25	0.25	0.50	0.151	-0.180	0.046
10	20	1	0.2	0.5	0.5	0.30	0.30	0.30	0.25	0.50	0.00	0.165	-0.180	0.046
10	20	1	0.2	0.5	0.5	0.30	0.30	0.30	0.25	0.50	0.25	0.160	-0.191	0.038
10	20	1	0.2	0.5	0.5	0.30	0.30	0.30	0.25	0.50	0.50	0.155	-0.199	0.039
10	20	1	0.2	0.5	0.5	0.30	0.30	0.30	0.50	0.00	0.00	0.142	-0.101	0.059
10	20	1	0.2	0.5	0.5	0.30	0.30	0.30	0.50	0.00	0.25	0.130	-0.103	0.057
10	20	1	0.2	0.5	0.5	0.30	0.30	0.30	0.50	0.00	0.50	0.133	-0.097	0.060
10	20	1	0.2	0.5	0.5	0.30	0.30	0.30	0.50	0.25	0.00	0.157	-0.121	0.052
10	20	1	0.2	0.5	0.5	0.30	0.30	0.30	0.50	0.25	0.25	0.148	-0.127	0.050
10	20	1	0.2	0.5	0.5	0.30	0.30	0.30	0.50	0.25	0.50	0.150	-0.128	0.045
10	20	1	0.2	0.5	0.5	0.30	0.30	0.30	0.50	0.50	0.00	0.166	-0.139	0.040
10	20	1	0.2	0.5	0.5	0.30	0.30	0.30	0.50	0.50	0.25	0.167	-0.134	0.042
10	20	1	0.2	0.5	0.5	0.30	0.30	0.30	0.50	0.50	0.50	0.167	-0.139	0.045
10	20	1	0.2	0.8	0.5	0.00	0.00	0.00	0.00	0.00	0.00	0.003	0.007	0.002
10	20	1	0.2	0.8	0.5	0.15	0.15	0.15	0.00	0.00	0.00	0.035	-0.100	0.013
10	20	1	0.2	0.8	0.5	0.15	0.15	0.15	0.00	0.00	0.25	0.038	-0.105	0.014
10	20	1	0.2	0.8	0.5	0.15	0.15	0.15	0.00	0.00	0.50	0.036	-0.103	0.016
10	20	1	0.2	0.8	0.5	0.15	0.15	0.15	0.00	0.25	0.00	0.042	-0.116	0.016
10	20	1	0.2	0.8	0.5	0.15	0.15	0.15	0.00	0.25	0.25	0.038	-0.109	0.011
10	20	1	0.2	0.8	0.5	0.15	0.15	0.15	0.00	0.25	0.50	0.039	-0.108	0.013
10	20	1	0.2	0.8	0.5	0.15	0.15	0.15	0.00	0.50	0.00	0.041	-0.125	0.009

(continued)

N	m_1	$\frac{m_2}{m_1}$	$E(C)$	$E(R)$	$E(U)$	σ_C	σ_R	σ_U	ρ_{CR}	ρ_{CU}	ρ_{RU}	Mean Bias c	Mean Bias r	Mean Bias u
10	20	1	0.2	0.8	0.5	0.15	0.15	0.15	0.00	0.50	0.25	0.042	-0.125	0.009
10	20	1	0.2	0.8	0.5	0.15	0.15	0.15	0.00	0.50	0.50	0.045	-0.134	0.011
10	20	1	0.2	0.8	0.5	0.15	0.15	0.15	0.25	0.00	0.00	0.034	-0.084	0.015
10	20	1	0.2	0.8	0.5	0.15	0.15	0.15	0.25	0.00	0.25	0.034	-0.082	0.011
10	20	1	0.2	0.8	0.5	0.15	0.15	0.15	0.25	0.00	0.50	0.035	-0.092	0.017
10	20	1	0.2	0.8	0.5	0.15	0.15	0.15	0.25	0.25	0.00	0.044	-0.098	0.015
10	20	1	0.2	0.8	0.5	0.15	0.15	0.15	0.25	0.25	0.25	0.041	-0.090	0.014
10	20	1	0.2	0.8	0.5	0.15	0.15	0.15	0.25	0.25	0.50	0.038	-0.095	0.011
10	20	1	0.2	0.8	0.5	0.15	0.15	0.15	0.25	0.50	0.00	0.047	-0.113	0.011
10	20	1	0.2	0.8	0.5	0.15	0.15	0.15	0.25	0.50	0.25	0.045	-0.098	0.009
10	20	1	0.2	0.8	0.5	0.15	0.15	0.15	0.25	0.50	0.50	0.047	-0.108	0.012
10	20	1	0.2	0.8	0.5	0.15	0.15	0.15	0.50	0.00	0.00	0.033	-0.051	0.016
10	20	1	0.2	0.8	0.5	0.15	0.15	0.15	0.50	0.00	0.25	0.035	-0.067	0.012
10	20	1	0.2	0.8	0.5	0.15	0.15	0.15	0.50	0.00	0.50	0.038	-0.075	0.016
10	20	1	0.2	0.8	0.5	0.15	0.15	0.15	0.50	0.25	0.00	0.040	-0.075	0.015
10	20	1	0.2	0.8	0.5	0.15	0.15	0.15	0.50	0.25	0.25	0.041	-0.071	0.012
10	20	1	0.2	0.8	0.5	0.15	0.15	0.15	0.50	0.25	0.50	0.041	-0.086	0.015
10	20	1	0.2	0.8	0.5	0.15	0.15	0.15	0.50	0.50	0.00	0.044	-0.090	0.012
10	20	1	0.2	0.8	0.5	0.15	0.15	0.15	0.50	0.50	0.25	0.045	-0.084	0.011
10	20	1	0.2	0.8	0.5	0.15	0.15	0.15	0.50	0.50	0.50	0.043	-0.089	0.011
10	20	1	0.2	0.8	0.5	0.30	0.30	0.30	0.00	0.00	0.00	0.136	-0.335	0.054
10	20	1	0.2	0.8	0.5	0.30	0.30	0.30	0.00	0.00	0.25	0.136	-0.347	0.054
10	20	1	0.2	0.8	0.5	0.30	0.30	0.30	0.00	0.00	0.50	0.140	-0.328	0.058
10	20	1	0.2	0.8	0.5	0.30	0.30	0.30	0.00	0.25	0.00	0.157	-0.356	0.050
10	20	1	0.2	0.8	0.5	0.30	0.30	0.30	0.00	0.25	0.25	0.156	-0.355	0.053
10	20	1	0.2	0.8	0.5	0.30	0.30	0.30	0.00	0.25	0.50	0.153	-0.358	0.052
10	20	1	0.2	0.8	0.5	0.30	0.30	0.30	0.00	0.50	0.00	0.166	-0.367	0.045
10	20	1	0.2	0.8	0.5	0.30	0.30	0.30	0.00	0.50	0.25	0.171	-0.378	0.043
10	20	1	0.2	0.8	0.5	0.30	0.30	0.30	0.00	0.50	0.50	0.172	-0.369	0.040
10	20	1	0.2	0.8	0.5	0.30	0.30	0.30	0.25	0.00	0.00	0.132	-0.290	0.065
10	20	1	0.2	0.8	0.5	0.30	0.30	0.30	0.25	0.00	0.25	0.135	-0.297	0.057
10	20	1	0.2	0.8	0.5	0.30	0.30	0.30	0.25	0.00	0.50	0.133	-0.297	0.060
10	20	1	0.2	0.8	0.5	0.30	0.30	0.30	0.25	0.25	0.00	0.147	-0.317	0.049
10	20	1	0.2	0.8	0.5	0.30	0.30	0.30	0.25	0.25	0.25	0.150	-0.310	0.049
10	20	1	0.2	0.8	0.5	0.30	0.30	0.30	0.25	0.25	0.50	0.151	-0.325	0.049
10	20	1	0.2	0.8	0.5	0.30	0.30	0.30	0.25	0.50	0.00	0.165	-0.334	0.042
10	20	1	0.2	0.8	0.5	0.30	0.30	0.30	0.25	0.50	0.25	0.164	-0.327	0.038
10	20	1	0.2	0.8	0.5	0.30	0.30	0.30	0.25	0.50	0.50	0.168	-0.333	0.040
10	20	1	0.2	0.8	0.5	0.30	0.30	0.30	0.50	0.00	0.00	0.136	-0.261	0.054

(continued)

N	m_1	$\frac{m_2}{m_1}$	$E(C)$	$E(R)$	$E(U)$	σ_C	σ_R	σ_U	ρ_{CR}	ρ_{CU}	ρ_{RU}	Mean Bias c	Mean Bias r	Mean Bias u
10	20	1	0.2	0.8	0.5	0.30	0.30	0.30	0.50	0.00	0.25	0.138	-0.251	0.059
10	20	1	0.2	0.8	0.5	0.30	0.30	0.30	0.50	0.00	0.50	0.136	-0.271	0.057
10	20	1	0.2	0.8	0.5	0.30	0.30	0.30	0.50	0.25	0.00	0.151	-0.279	0.053
10	20	1	0.2	0.8	0.5	0.30	0.30	0.30	0.50	0.25	0.25	0.156	-0.293	0.047
10	20	1	0.2	0.8	0.5	0.30	0.30	0.30	0.50	0.25	0.50	0.151	-0.285	0.051
10	20	1	0.2	0.8	0.5	0.30	0.30	0.30	0.50	0.50	0.00	0.169	-0.288	0.045
10	20	1	0.2	0.8	0.5	0.30	0.30	0.30	0.50	0.50	0.25	0.162	-0.295	0.047
10	20	1	0.2	0.8	0.5	0.30	0.30	0.30	0.50	0.50	0.50	0.173	-0.309	0.038
10	20	1	0.5	0.2	0.5	0.00	0.00	0.00	0.00	0.00	0.00	-0.003	0.001	0.001
10	20	1	0.5	0.2	0.5	0.15	0.15	0.15	0.00	0.00	0.00	0.022	-0.004	0.013
10	20	1	0.5	0.2	0.5	0.15	0.15	0.15	0.00	0.00	0.25	0.020	-0.007	0.006
10	20	1	0.5	0.2	0.5	0.15	0.15	0.15	0.00	0.00	0.50	0.021	-0.005	0.015
10	20	1	0.5	0.2	0.5	0.15	0.15	0.15	0.00	0.25	0.00	0.025	-0.008	0.008
10	20	1	0.5	0.2	0.5	0.15	0.15	0.15	0.00	0.25	0.25	0.025	-0.010	0.007
10	20	1	0.5	0.2	0.5	0.15	0.15	0.15	0.00	0.25	0.50	0.026	-0.009	0.008
10	20	1	0.5	0.2	0.5	0.15	0.15	0.15	0.00	0.50	0.00	0.027	-0.009	0.001
10	20	1	0.5	0.2	0.5	0.15	0.15	0.15	0.00	0.50	0.25	0.029	-0.012	0.004
10	20	1	0.5	0.2	0.5	0.15	0.15	0.15	0.00	0.50	0.50	0.034	-0.007	0.007
10	20	1	0.5	0.2	0.5	0.15	0.15	0.15	0.25	0.00	0.00	0.016	0.003	0.006
10	20	1	0.5	0.2	0.5	0.15	0.15	0.15	0.25	0.00	0.25	0.021	0.004	0.010
10	20	1	0.5	0.2	0.5	0.15	0.15	0.15	0.25	0.00	0.50	0.019	0.006	0.012
10	20	1	0.5	0.2	0.5	0.15	0.15	0.15	0.25	0.25	0.00	0.022	0.001	0.008
10	20	1	0.5	0.2	0.5	0.15	0.15	0.15	0.25	0.25	0.25	0.025	0.000	0.008
10	20	1	0.5	0.2	0.5	0.15	0.15	0.15	0.25	0.25	0.50	0.023	-0.001	0.006
10	20	1	0.5	0.2	0.5	0.15	0.15	0.15	0.25	0.50	0.00	0.027	0.001	0.005
10	20	1	0.5	0.2	0.5	0.15	0.15	0.15	0.25	0.50	0.25	0.028	-0.003	0.006
10	20	1	0.5	0.2	0.5	0.15	0.15	0.15	0.25	0.50	0.50	0.029	-0.005	0.006
10	20	1	0.5	0.2	0.5	0.15	0.15	0.15	0.50	0.00	0.00	0.020	0.017	0.009
10	20	1	0.5	0.2	0.5	0.15	0.15	0.15	0.50	0.00	0.25	0.019	0.015	0.011
10	20	1	0.5	0.2	0.5	0.15	0.15	0.15	0.50	0.00	0.50	0.020	0.014	0.012
10	20	1	0.5	0.2	0.5	0.15	0.15	0.15	0.50	0.25	0.00	0.022	0.010	0.008
10	20	1	0.5	0.2	0.5	0.15	0.15	0.15	0.50	0.25	0.25	0.024	0.011	0.007
10	20	1	0.5	0.2	0.5	0.15	0.15	0.15	0.50	0.25	0.50	0.024	0.011	0.010
10	20	1	0.5	0.2	0.5	0.15	0.15	0.15	0.50	0.50	0.00	0.030	0.011	0.005
10	20	1	0.5	0.2	0.5	0.15	0.15	0.15	0.50	0.50	0.25	0.025	0.007	0.005
10	20	1	0.5	0.2	0.5	0.15	0.15	0.15	0.50	0.50	0.50	0.030	0.006	0.002
10	20	1	0.5	0.2	0.5	0.30	0.30	0.30	0.00	0.00	0.00	0.080	-0.027	0.036
10	20	1	0.5	0.2	0.5	0.30	0.30	0.30	0.00	0.00	0.25	0.079	-0.027	0.043
10	20	1	0.5	0.2	0.5	0.30	0.30	0.30	0.00	0.00	0.50	0.072	-0.023	0.042

(continued)

N	m_1	$\frac{m_2}{m_1}$	$E(C)$	$E(R)$	$E(U)$	σ_C	σ_R	σ_U	ρ_{CR}	ρ_{CU}	ρ_{RU}	Mean Bias		
												c	r	u
10	20	1	0.5	0.2	0.5	0.30	0.30	0.30	0.00	0.25	0.00	0.095	-0.032	0.034
10	20	1	0.5	0.2	0.5	0.30	0.30	0.30	0.00	0.25	0.25	0.100	-0.033	0.032
10	20	1	0.5	0.2	0.5	0.30	0.30	0.30	0.00	0.25	0.50	0.099	-0.032	0.031
10	20	1	0.5	0.2	0.5	0.30	0.30	0.30	0.00	0.50	0.00	0.122	-0.037	0.021
10	20	1	0.5	0.2	0.5	0.30	0.30	0.30	0.00	0.50	0.25	0.118	-0.041	0.016
10	20	1	0.5	0.2	0.5	0.30	0.30	0.30	0.00	0.50	0.50	0.124	-0.038	0.020
10	20	1	0.5	0.2	0.5	0.30	0.30	0.30	0.25	0.00	0.00	0.082	0.012	0.044
10	20	1	0.5	0.2	0.5	0.30	0.30	0.30	0.25	0.00	0.25	0.073	0.003	0.042
10	20	1	0.5	0.2	0.5	0.30	0.30	0.30	0.25	0.00	0.50	0.081	0.003	0.039
10	20	1	0.5	0.2	0.5	0.30	0.30	0.30	0.25	0.25	0.00	0.097	-0.002	0.035
10	20	1	0.5	0.2	0.5	0.30	0.30	0.30	0.25	0.25	0.25	0.101	-0.005	0.028
10	20	1	0.5	0.2	0.5	0.30	0.30	0.30	0.25	0.25	0.50	0.095	-0.006	0.033
10	20	1	0.5	0.2	0.5	0.30	0.30	0.30	0.25	0.50	0.00	0.119	-0.016	0.022
10	20	1	0.5	0.2	0.5	0.30	0.30	0.30	0.25	0.50	0.25	0.121	-0.009	0.027
10	20	1	0.5	0.2	0.5	0.30	0.30	0.30	0.25	0.50	0.50	0.122	-0.013	0.027
10	20	1	0.5	0.2	0.5	0.30	0.30	0.30	0.50	0.00	0.00	0.083	0.032	0.041
10	20	1	0.5	0.2	0.5	0.30	0.30	0.30	0.50	0.00	0.25	0.078	0.035	0.040
10	20	1	0.5	0.2	0.5	0.30	0.30	0.30	0.50	0.00	0.50	0.075	0.032	0.037
10	20	1	0.5	0.2	0.5	0.30	0.30	0.30	0.50	0.25	0.00	0.105	0.031	0.030
10	20	1	0.5	0.2	0.5	0.30	0.30	0.30	0.50	0.25	0.25	0.096	0.020	0.033
10	20	1	0.5	0.2	0.5	0.30	0.30	0.30	0.50	0.25	0.50	0.099	0.020	0.025
10	20	1	0.5	0.2	0.5	0.30	0.30	0.30	0.50	0.50	0.00	0.119	0.012	0.020
10	20	1	0.5	0.2	0.5	0.30	0.30	0.30	0.50	0.50	0.25	0.123	0.016	0.020
10	20	1	0.5	0.2	0.5	0.30	0.30	0.30	0.50	0.50	0.50	0.120	0.020	0.026
10	20	1	0.5	0.5	0.5	0.00	0.00	0.00	0.00	0.00	0.00	-0.001	0.003	0.000
10	20	1	0.5	0.5	0.5	0.15	0.15	0.15	0.00	0.00	0.00	0.021	-0.015	0.007
10	20	1	0.5	0.5	0.5	0.15	0.15	0.15	0.00	0.00	0.25	0.017	-0.018	0.010
10	20	1	0.5	0.5	0.5	0.15	0.15	0.15	0.00	0.00	0.50	0.016	-0.015	0.011
10	20	1	0.5	0.5	0.5	0.15	0.15	0.15	0.00	0.25	0.00	0.026	-0.016	0.006
10	20	1	0.5	0.5	0.5	0.15	0.15	0.15	0.00	0.25	0.25	0.025	-0.025	0.006
10	20	1	0.5	0.5	0.5	0.15	0.15	0.15	0.00	0.25	0.50	0.025	-0.022	0.007
10	20	1	0.5	0.5	0.5	0.15	0.15	0.15	0.00	0.50	0.00	0.029	-0.023	0.004
10	20	1	0.5	0.5	0.5	0.15	0.15	0.15	0.00	0.50	0.25	0.031	-0.028	0.006
10	20	1	0.5	0.5	0.5	0.15	0.15	0.15	0.00	0.50	0.50	0.033	-0.025	0.006
10	20	1	0.5	0.5	0.5	0.15	0.15	0.15	0.25	0.00	0.00	0.019	-0.002	0.009
10	20	1	0.5	0.5	0.5	0.15	0.15	0.15	0.25	0.00	0.25	0.020	-0.004	0.012
10	20	1	0.5	0.5	0.5	0.15	0.15	0.15	0.25	0.00	0.50	0.020	-0.006	0.015
10	20	1	0.5	0.5	0.5	0.15	0.15	0.15	0.25	0.25	0.00	0.024	-0.014	0.007
10	20	1	0.5	0.5	0.5	0.15	0.15	0.15	0.25	0.25	0.25	0.021	-0.009	0.009

(continued)

N	m_1	$\frac{m_2}{m_1}$	$E(C)$	$E(R)$	$E(U)$	σ_C	σ_R	σ_U	ρ_{CR}	ρ_{CU}	ρ_{RU}	Mean Bias c	Mean Bias r	Mean Bias u
10	20	1	0.5	0.5	0.5	0.15	0.15	0.15	0.25	0.25	0.50	0.025	-0.012	0.009
10	20	1	0.5	0.5	0.5	0.15	0.15	0.15	0.25	0.50	0.00	0.032	-0.017	0.009
10	20	1	0.5	0.5	0.5	0.15	0.15	0.15	0.25	0.50	0.25	0.027	-0.022	0.005
10	20	1	0.5	0.5	0.5	0.15	0.15	0.15	0.25	0.50	0.50	0.028	-0.017	0.006
10	20	1	0.5	0.5	0.5	0.15	0.15	0.15	0.50	0.00	0.00	0.021	0.002	0.008
10	20	1	0.5	0.5	0.5	0.15	0.15	0.15	0.50	0.00	0.25	0.016	0.001	0.009
10	20	1	0.5	0.5	0.5	0.15	0.15	0.15	0.50	0.00	0.50	0.018	0.003	0.013
10	20	1	0.5	0.5	0.5	0.15	0.15	0.15	0.50	0.25	0.00	0.027	0.002	0.009
10	20	1	0.5	0.5	0.5	0.15	0.15	0.15	0.50	0.25	0.25	0.022	-0.004	0.009
10	20	1	0.5	0.5	0.5	0.15	0.15	0.15	0.50	0.25	0.50	0.024	0.001	0.008
10	20	1	0.5	0.5	0.5	0.15	0.15	0.15	0.50	0.50	0.00	0.029	-0.007	0.007
10	20	1	0.5	0.5	0.5	0.15	0.15	0.15	0.50	0.50	0.25	0.031	-0.003	0.008
10	20	1	0.5	0.5	0.5	0.15	0.15	0.15	0.50	0.50	0.50	0.029	-0.006	0.007
10	20	1	0.5	0.5	0.5	0.30	0.30	0.30	0.00	0.00	0.00	0.073	-0.062	0.037
10	20	1	0.5	0.5	0.5	0.30	0.30	0.30	0.00	0.00	0.25	0.077	-0.063	0.037
10	20	1	0.5	0.5	0.5	0.30	0.30	0.30	0.00	0.00	0.50	0.084	-0.070	0.037
10	20	1	0.5	0.5	0.5	0.30	0.30	0.30	0.00	0.25	0.00	0.104	-0.077	0.032
10	20	1	0.5	0.5	0.5	0.30	0.30	0.30	0.00	0.25	0.25	0.098	-0.082	0.033
10	20	1	0.5	0.5	0.5	0.30	0.30	0.30	0.00	0.25	0.50	0.098	-0.082	0.034
10	20	1	0.5	0.5	0.5	0.30	0.30	0.30	0.00	0.50	0.00	0.118	-0.098	0.022
10	20	1	0.5	0.5	0.5	0.30	0.30	0.30	0.00	0.50	0.25	0.125	-0.098	0.021
10	20	1	0.5	0.5	0.5	0.30	0.30	0.30	0.00	0.50	0.50	0.115	-0.101	0.018
10	20	1	0.5	0.5	0.5	0.30	0.30	0.30	0.25	0.00	0.00	0.083	-0.032	0.043
10	20	1	0.5	0.5	0.5	0.30	0.30	0.30	0.25	0.00	0.25	0.077	-0.023	0.048
10	20	1	0.5	0.5	0.5	0.30	0.30	0.30	0.25	0.00	0.50	0.076	-0.032	0.039
10	20	1	0.5	0.5	0.5	0.30	0.30	0.30	0.25	0.25	0.00	0.100	-0.050	0.029
10	20	1	0.5	0.5	0.5	0.30	0.30	0.30	0.25	0.25	0.25	0.101	-0.043	0.030
10	20	1	0.5	0.5	0.5	0.30	0.30	0.30	0.25	0.25	0.50	0.101	-0.046	0.037
10	20	1	0.5	0.5	0.5	0.30	0.30	0.30	0.25	0.50	0.00	0.119	-0.064	0.026
10	20	1	0.5	0.5	0.5	0.30	0.30	0.30	0.25	0.50	0.25	0.124	-0.056	0.025
10	20	1	0.5	0.5	0.5	0.30	0.30	0.30	0.25	0.50	0.50	0.122	-0.059	0.022
10	20	1	0.5	0.5	0.5	0.30	0.30	0.30	0.50	0.00	0.00	0.073	0.008	0.040
10	20	1	0.5	0.5	0.5	0.30	0.30	0.30	0.50	0.00	0.25	0.077	0.000	0.043
10	20	1	0.5	0.5	0.5	0.30	0.30	0.30	0.50	0.00	0.50	0.075	0.005	0.042
10	20	1	0.5	0.5	0.5	0.30	0.30	0.30	0.50	0.25	0.00	0.100	-0.009	0.028
10	20	1	0.5	0.5	0.5	0.30	0.30	0.30	0.50	0.25	0.25	0.100	-0.012	0.033
10	20	1	0.5	0.5	0.5	0.30	0.30	0.30	0.50	0.25	0.50	0.100	-0.014	0.029
10	20	1	0.5	0.5	0.5	0.30	0.30	0.30	0.50	0.50	0.00	0.124	-0.038	0.024
10	20	1	0.5	0.5	0.5	0.30	0.30	0.30	0.50	0.50	0.25	0.120	-0.025	0.022

(continued)

N	m_1	$\frac{m_2}{m_1}$	$E(C)$	$E(R)$	$E(U)$	σ_C	σ_R	σ_U	ρ_{CR}	ρ_{CU}	ρ_{RU}	Mean Bias c	r	u
10	20	1	0.5	0.5	0.5	0.30	0.30	0.30	0.50	0.50	0.50	0.121	-0.033	0.022
10	20	1	0.5	0.8	0.5	0.00	0.00	0.00	0.00	0.00	0.00	-0.003	0.009	-0.001
10	20	1	0.5	0.8	0.5	0.15	0.15	0.15	0.00	0.00	0.00	0.017	-0.027	0.007
10	20	1	0.5	0.8	0.5	0.15	0.15	0.15	0.00	0.00	0.25	0.022	-0.025	0.010
10	20	1	0.5	0.8	0.5	0.15	0.15	0.15	0.00	0.00	0.50	0.015	-0.020	0.010
10	20	1	0.5	0.8	0.5	0.15	0.15	0.15	0.00	0.25	0.00	0.021	-0.035	0.007
10	20	1	0.5	0.8	0.5	0.15	0.15	0.15	0.00	0.25	0.25	0.027	-0.034	0.009
10	20	1	0.5	0.8	0.5	0.15	0.15	0.15	0.00	0.25	0.50	0.025	-0.035	0.010
10	20	1	0.5	0.8	0.5	0.15	0.15	0.15	0.00	0.50	0.00	0.030	-0.043	0.003
10	20	1	0.5	0.8	0.5	0.15	0.15	0.15	0.00	0.50	0.25	0.029	-0.047	0.007
10	20	1	0.5	0.8	0.5	0.15	0.15	0.15	0.00	0.50	0.50	0.030	-0.042	0.008
10	20	1	0.5	0.8	0.5	0.15	0.15	0.15	0.25	0.00	0.00	0.020	-0.014	0.013
10	20	1	0.5	0.8	0.5	0.15	0.15	0.15	0.25	0.00	0.25	0.022	-0.018	0.013
10	20	1	0.5	0.8	0.5	0.15	0.15	0.15	0.25	0.00	0.50	0.021	-0.023	0.013
10	20	1	0.5	0.8	0.5	0.15	0.15	0.15	0.25	0.25	0.00	0.028	-0.024	0.009
10	20	1	0.5	0.8	0.5	0.15	0.15	0.15	0.25	0.25	0.25	0.026	-0.022	0.006
10	20	1	0.5	0.8	0.5	0.15	0.15	0.15	0.25	0.25	0.50	0.026	-0.024	0.006
10	20	1	0.5	0.8	0.5	0.15	0.15	0.15	0.25	0.50	0.00	0.031	-0.032	0.007
10	20	1	0.5	0.8	0.5	0.15	0.15	0.15	0.25	0.50	0.25	0.029	-0.031	0.007
10	20	1	0.5	0.8	0.5	0.15	0.15	0.15	0.25	0.50	0.50	0.029	-0.036	0.003
10	20	1	0.5	0.8	0.5	0.15	0.15	0.15	0.50	0.00	0.00	0.022	-0.008	0.010
10	20	1	0.5	0.8	0.5	0.15	0.15	0.15	0.50	0.00	0.25	0.018	-0.007	0.011
10	20	1	0.5	0.8	0.5	0.15	0.15	0.15	0.50	0.00	0.50	0.020	-0.012	0.011
10	20	1	0.5	0.8	0.5	0.15	0.15	0.15	0.50	0.25	0.00	0.022	-0.012	0.006
10	20	1	0.5	0.8	0.5	0.15	0.15	0.15	0.50	0.25	0.25	0.025	-0.016	0.008
10	20	1	0.5	0.8	0.5	0.15	0.15	0.15	0.50	0.25	0.50	0.026	-0.020	0.009
10	20	1	0.5	0.8	0.5	0.15	0.15	0.15	0.50	0.50	0.00	0.031	-0.022	0.008
10	20	1	0.5	0.8	0.5	0.15	0.15	0.15	0.50	0.50	0.25	0.031	-0.022	0.004
10	20	1	0.5	0.8	0.5	0.15	0.15	0.15	0.50	0.50	0.50	0.030	-0.022	0.006
10	20	1	0.5	0.8	0.5	0.30	0.30	0.30	0.00	0.00	0.00	0.077	-0.103	0.037
10	20	1	0.5	0.8	0.5	0.30	0.30	0.30	0.00	0.00	0.25	0.077	-0.102	0.037
10	20	1	0.5	0.8	0.5	0.30	0.30	0.30	0.00	0.00	0.50	0.074	-0.110	0.047
10	20	1	0.5	0.8	0.5	0.30	0.30	0.30	0.00	0.25	0.00	0.098	-0.130	0.032
10	20	1	0.5	0.8	0.5	0.30	0.30	0.30	0.00	0.25	0.25	0.100	-0.127	0.031
10	20	1	0.5	0.8	0.5	0.30	0.30	0.30	0.00	0.25	0.50	0.097	-0.128	0.036
10	20	1	0.5	0.8	0.5	0.30	0.30	0.30	0.00	0.50	0.00	0.121	-0.155	0.021
10	20	1	0.5	0.8	0.5	0.30	0.30	0.30	0.00	0.50	0.25	0.128	-0.160	0.025
10	20	1	0.5	0.8	0.5	0.30	0.30	0.30	0.00	0.50	0.50	0.121	-0.157	0.022
10	20	1	0.5	0.8	0.5	0.30	0.30	0.30	0.25	0.00	0.00	0.077	-0.085	0.041

(continued)

N	m_1	$\frac{m_2}{m_1}$	$E(C)$	$E(R)$	$E(U)$	σ_C	σ_R	σ_U	ρ_{CR}	ρ_{CU}	ρ_{RU}	Mean Bias c	r	u
10	20	1	0.5	0.8	0.5	0.30	0.30	0.30	0.25	0.00	0.25	0.080	-0.079	0.042
10	20	1	0.5	0.8	0.5	0.30	0.30	0.30	0.25	0.00	0.50	0.077	-0.074	0.040
10	20	1	0.5	0.8	0.5	0.30	0.30	0.30	0.25	0.25	0.00	0.097	-0.105	0.030
10	20	1	0.5	0.8	0.5	0.30	0.30	0.30	0.25	0.25	0.25	0.099	-0.103	0.030
10	20	1	0.5	0.8	0.5	0.30	0.30	0.30	0.25	0.25	0.50	0.097	-0.097	0.036
10	20	1	0.5	0.8	0.5	0.30	0.30	0.30	0.25	0.50	0.00	0.123	-0.124	0.028
10	20	1	0.5	0.8	0.5	0.30	0.30	0.30	0.25	0.50	0.25	0.123	-0.134	0.019
10	20	1	0.5	0.8	0.5	0.30	0.30	0.30	0.25	0.50	0.50	0.121	-0.138	0.019
10	20	1	0.5	0.8	0.5	0.30	0.30	0.30	0.50	0.00	0.00	0.079	-0.042	0.040
10	20	1	0.5	0.8	0.5	0.30	0.30	0.30	0.50	0.00	0.25	0.070	-0.053	0.049
10	20	1	0.5	0.8	0.5	0.30	0.30	0.30	0.50	0.00	0.50	0.077	-0.046	0.042
10	20	1	0.5	0.8	0.5	0.30	0.30	0.30	0.50	0.25	0.00	0.097	-0.070	0.031
10	20	1	0.5	0.8	0.5	0.30	0.30	0.30	0.50	0.25	0.25	0.099	-0.073	0.037
10	20	1	0.5	0.8	0.5	0.30	0.30	0.30	0.50	0.25	0.50	0.099	-0.080	0.030
10	20	1	0.5	0.8	0.5	0.30	0.30	0.30	0.50	0.50	0.00	0.122	-0.098	0.025
10	20	1	0.5	0.8	0.5	0.30	0.30	0.30	0.50	0.50	0.25	0.124	-0.103	0.022
10	20	1	0.5	0.8	0.5	0.30	0.30	0.30	0.50	0.50	0.50	0.124	-0.102	0.020
10	20	1	0.8	0.2	0.5	0.00	0.00	0.00	0.00	0.00	0.00	0.000	-0.001	0.002
10	20	1	0.8	0.2	0.5	0.15	0.15	0.15	0.00	0.00	0.00	0.005	-0.002	0.005
10	20	1	0.8	0.2	0.5	0.15	0.15	0.15	0.00	0.00	0.25	0.007	-0.002	0.007
10	20	1	0.8	0.2	0.5	0.15	0.15	0.15	0.00	0.00	0.50	0.009	-0.003	0.007
10	20	1	0.8	0.2	0.5	0.15	0.15	0.15	0.00	0.25	0.00	0.015	-0.004	0.001
10	20	1	0.8	0.2	0.5	0.15	0.15	0.15	0.00	0.25	0.25	0.014	-0.001	0.002
10	20	1	0.8	0.2	0.5	0.15	0.15	0.15	0.00	0.25	0.50	0.013	-0.002	0.004
10	20	1	0.8	0.2	0.5	0.15	0.15	0.15	0.00	0.50	0.00	0.019	-0.004	-0.002
10	20	1	0.8	0.2	0.5	0.15	0.15	0.15	0.00	0.50	0.25	0.014	-0.001	-0.001
10	20	1	0.8	0.2	0.5	0.15	0.15	0.15	0.00	0.50	0.50	0.019	-0.005	0.001
10	20	1	0.8	0.2	0.5	0.15	0.15	0.15	0.25	0.00	0.00	0.007	0.005	0.008
10	20	1	0.8	0.2	0.5	0.15	0.15	0.15	0.25	0.00	0.25	0.007	0.006	0.008
10	20	1	0.8	0.2	0.5	0.15	0.15	0.15	0.25	0.00	0.50	0.009	0.002	0.004
10	20	1	0.8	0.2	0.5	0.15	0.15	0.15	0.25	0.25	0.00	0.011	0.002	0.002
10	20	1	0.8	0.2	0.5	0.15	0.15	0.15	0.25	0.25	0.25	0.012	0.002	0.002
10	20	1	0.8	0.2	0.5	0.15	0.15	0.15	0.25	0.25	0.50	0.012	0.003	0.000
10	20	1	0.8	0.2	0.5	0.15	0.15	0.15	0.25	0.50	0.00	0.022	0.001	0.000
10	20	1	0.8	0.2	0.5	0.15	0.15	0.15	0.25	0.50	0.25	0.019	0.004	-0.002
10	20	1	0.8	0.2	0.5	0.15	0.15	0.15	0.25	0.50	0.50	0.019	0.001	0.000
10	20	1	0.8	0.2	0.5	0.15	0.15	0.15	0.50	0.00	0.00	0.001	0.007	0.006
10	20	1	0.8	0.2	0.5	0.15	0.15	0.15	0.50	0.00	0.25	0.006	0.008	0.006
10	20	1	0.8	0.2	0.5	0.15	0.15	0.15	0.50	0.00	0.50	0.008	0.012	0.005

(continued)

N	m_1	$\frac{m_2}{m_1}$	$E(C)$	$E(R)$	$E(U)$	σ_C	σ_R	σ_U	ρ_{CR}	ρ_{CU}	ρ_{RU}	Mean Bias		
												c	r	u
10	20	1	0.8	0.2	0.5	0.15	0.15	0.15	0.50	0.25	0.00	0.012	0.009	0.001
10	20	1	0.8	0.2	0.5	0.15	0.15	0.15	0.50	0.25	0.25	0.011	0.008	0.005
10	20	1	0.8	0.2	0.5	0.15	0.15	0.15	0.50	0.25	0.50	0.013	0.009	0.002
10	20	1	0.8	0.2	0.5	0.15	0.15	0.15	0.50	0.50	0.00	0.019	0.007	-0.004
10	20	1	0.8	0.2	0.5	0.15	0.15	0.15	0.50	0.50	0.25	0.020	0.006	0.002
10	20	1	0.8	0.2	0.5	0.15	0.15	0.15	0.50	0.50	0.50	0.018	0.006	-0.001
10	20	1	0.8	0.2	0.5	0.30	0.30	0.30	0.00	0.00	0.00	0.025	-0.004	0.022
10	20	1	0.8	0.2	0.5	0.30	0.30	0.30	0.00	0.00	0.25	0.027	-0.004	0.019
10	20	1	0.8	0.2	0.5	0.30	0.30	0.30	0.00	0.00	0.50	0.027	-0.003	0.015
10	20	1	0.8	0.2	0.5	0.30	0.30	0.30	0.00	0.25	0.00	0.048	-0.006	0.009
10	20	1	0.8	0.2	0.5	0.30	0.30	0.30	0.00	0.25	0.25	0.050	-0.009	0.000
10	20	1	0.8	0.2	0.5	0.30	0.30	0.30	0.00	0.25	0.50	0.053	-0.013	0.011
10	20	1	0.8	0.2	0.5	0.30	0.30	0.30	0.00	0.50	0.00	0.076	-0.016	0.002
10	20	1	0.8	0.2	0.5	0.30	0.30	0.30	0.00	0.50	0.25	0.073	-0.018	0.000
10	20	1	0.8	0.2	0.5	0.30	0.30	0.30	0.00	0.50	0.50	0.077	-0.015	0.006
10	20	1	0.8	0.2	0.5	0.30	0.30	0.30	0.25	0.00	0.00	0.029	0.011	0.015
10	20	1	0.8	0.2	0.5	0.30	0.30	0.30	0.25	0.00	0.25	0.022	0.007	0.020
10	20	1	0.8	0.2	0.5	0.30	0.30	0.30	0.25	0.00	0.50	0.028	0.013	0.015
10	20	1	0.8	0.2	0.5	0.30	0.30	0.30	0.25	0.25	0.00	0.047	0.007	0.008
10	20	1	0.8	0.2	0.5	0.30	0.30	0.30	0.25	0.25	0.25	0.049	0.000	0.005
10	20	1	0.8	0.2	0.5	0.30	0.30	0.30	0.25	0.25	0.50	0.052	0.003	0.009
10	20	1	0.8	0.2	0.5	0.30	0.30	0.30	0.25	0.50	0.00	0.074	0.000	0.003
10	20	1	0.8	0.2	0.5	0.30	0.30	0.30	0.25	0.50	0.25	0.073	-0.001	0.003
10	20	1	0.8	0.2	0.5	0.30	0.30	0.30	0.25	0.50	0.50	0.074	-0.003	-0.006
10	20	1	0.8	0.2	0.5	0.30	0.30	0.30	0.50	0.00	0.00	0.029	0.028	0.019
10	20	1	0.8	0.2	0.5	0.30	0.30	0.30	0.50	0.00	0.25	0.023	0.023	0.020
10	20	1	0.8	0.2	0.5	0.30	0.30	0.30	0.50	0.00	0.50	0.027	0.023	0.017
10	20	1	0.8	0.2	0.5	0.30	0.30	0.30	0.50	0.25	0.00	0.051	0.018	0.009
10	20	1	0.8	0.2	0.5	0.30	0.30	0.30	0.50	0.25	0.25	0.050	0.022	0.014
10	20	1	0.8	0.2	0.5	0.30	0.30	0.30	0.50	0.25	0.50	0.055	0.018	0.010
10	20	1	0.8	0.2	0.5	0.30	0.30	0.30	0.50	0.50	0.00	0.074	0.018	-0.001
10	20	1	0.8	0.2	0.5	0.30	0.30	0.30	0.50	0.50	0.25	0.069	0.009	0.002
10	20	1	0.8	0.2	0.5	0.30	0.30	0.30	0.50	0.50	0.50	0.072	0.010	0.000
10	20	1	0.8	0.5	0.5	0.00	0.00	0.00	0.00	0.00	0.00	-0.002	0.001	0.000
10	20	1	0.8	0.5	0.5	0.15	0.15	0.15	0.00	0.00	0.00	0.007	-0.004	0.004
10	20	1	0.8	0.5	0.5	0.15	0.15	0.15	0.00	0.00	0.25	0.008	-0.002	0.003
10	20	1	0.8	0.5	0.5	0.15	0.15	0.15	0.00	0.00	0.50	0.006	0.003	0.007
10	20	1	0.8	0.5	0.5	0.15	0.15	0.15	0.00	0.25	0.00	0.011	-0.011	0.001
10	20	1	0.8	0.5	0.5	0.15	0.15	0.15	0.00	0.25	0.25	0.013	-0.006	0.001

(continued)

N	m_1	$\frac{m_2}{m_1}$	$E(C)$	$E(R)$	$E(U)$	σ_C	σ_R	σ_U	ρ_{CR}	ρ_{CU}	ρ_{RU}	Mean Bias c	Mean Bias r	Mean Bias u
10	20	1	0.8	0.5	0.5	0.15	0.15	0.15	0.00	0.25	0.50	0.012	-0.010	0.002
10	20	1	0.8	0.5	0.5	0.15	0.15	0.15	0.00	0.50	0.00	0.016	-0.008	-0.005
10	20	1	0.8	0.5	0.5	0.15	0.15	0.15	0.00	0.50	0.25	0.015	-0.007	-0.002
10	20	1	0.8	0.5	0.5	0.15	0.15	0.15	0.00	0.50	0.50	0.018	-0.013	-0.003
10	20	1	0.8	0.5	0.5	0.15	0.15	0.15	0.25	0.00	0.00	0.008	0.006	0.009
10	20	1	0.8	0.5	0.5	0.15	0.15	0.15	0.25	0.00	0.25	0.006	0.003	0.004
10	20	1	0.8	0.5	0.5	0.15	0.15	0.15	0.25	0.00	0.50	0.006	0.005	0.006
10	20	1	0.8	0.5	0.5	0.15	0.15	0.15	0.25	0.25	0.00	0.009	-0.005	0.002
10	20	1	0.8	0.5	0.5	0.15	0.15	0.15	0.25	0.25	0.25	0.010	-0.002	0.005
10	20	1	0.8	0.5	0.5	0.15	0.15	0.15	0.25	0.25	0.50	0.012	-0.001	0.006
10	20	1	0.8	0.5	0.5	0.15	0.15	0.15	0.25	0.50	0.00	0.017	-0.006	0.001
10	20	1	0.8	0.5	0.5	0.15	0.15	0.15	0.25	0.50	0.25	0.020	-0.002	-0.003
10	20	1	0.8	0.5	0.5	0.15	0.15	0.15	0.25	0.50	0.50	0.020	-0.007	-0.002
10	20	1	0.8	0.5	0.5	0.15	0.15	0.15	0.50	0.00	0.00	0.005	0.008	0.008
10	20	1	0.8	0.5	0.5	0.15	0.15	0.15	0.50	0.00	0.25	0.008	0.012	0.006
10	20	1	0.8	0.5	0.5	0.15	0.15	0.15	0.50	0.00	0.50	0.005	0.012	0.007
10	20	1	0.8	0.5	0.5	0.15	0.15	0.15	0.50	0.25	0.00	0.012	0.004	0.001
10	20	1	0.8	0.5	0.5	0.15	0.15	0.15	0.50	0.25	0.25	0.012	0.006	0.005
10	20	1	0.8	0.5	0.5	0.15	0.15	0.15	0.50	0.25	0.50	0.012	0.005	0.001
10	20	1	0.8	0.5	0.5	0.15	0.15	0.15	0.50	0.50	0.00	0.021	0.000	0.004
10	20	1	0.8	0.5	0.5	0.15	0.15	0.15	0.50	0.50	0.25	0.017	0.001	0.000
10	20	1	0.8	0.5	0.5	0.15	0.15	0.15	0.50	0.50	0.50	0.017	0.002	0.003
10	20	1	0.8	0.5	0.5	0.30	0.30	0.30	0.00	0.00	0.00	0.028	-0.015	0.020
10	20	1	0.8	0.5	0.5	0.30	0.30	0.30	0.00	0.00	0.25	0.030	-0.023	0.015
10	20	1	0.8	0.5	0.5	0.30	0.30	0.30	0.00	0.00	0.50	0.026	-0.014	0.024
10	20	1	0.8	0.5	0.5	0.30	0.30	0.30	0.00	0.25	0.00	0.047	-0.032	0.008
10	20	1	0.8	0.5	0.5	0.30	0.30	0.30	0.00	0.25	0.25	0.047	-0.037	0.009
10	20	1	0.8	0.5	0.5	0.30	0.30	0.30	0.00	0.25	0.50	0.050	-0.029	0.012
10	20	1	0.8	0.5	0.5	0.30	0.30	0.30	0.00	0.50	0.00	0.075	-0.043	0.003
10	20	1	0.8	0.5	0.5	0.30	0.30	0.30	0.00	0.50	0.25	0.074	-0.045	0.004
10	20	1	0.8	0.5	0.5	0.30	0.30	0.30	0.00	0.50	0.50	0.071	-0.049	-0.005
10	20	1	0.8	0.5	0.5	0.30	0.30	0.30	0.25	0.00	0.00	0.025	0.009	0.018
10	20	1	0.8	0.5	0.5	0.30	0.30	0.30	0.25	0.00	0.25	0.025	0.015	0.017
10	20	1	0.8	0.5	0.5	0.30	0.30	0.30	0.25	0.00	0.50	0.030	0.008	0.018
10	20	1	0.8	0.5	0.5	0.30	0.30	0.30	0.25	0.25	0.00	0.050	-0.006	0.007
10	20	1	0.8	0.5	0.5	0.30	0.30	0.30	0.25	0.25	0.25	0.046	-0.015	0.008
10	20	1	0.8	0.5	0.5	0.30	0.30	0.30	0.25	0.25	0.50	0.047	-0.007	0.010
10	20	1	0.8	0.5	0.5	0.30	0.30	0.30	0.25	0.50	0.00	0.074	-0.025	0.003
10	20	1	0.8	0.5	0.5	0.30	0.30	0.30	0.25	0.50	0.25	0.072	-0.021	0.003

(continued)

												Mean Bias		
N	m_1	$\frac{m_2}{m_1}$	$E(C)$	$E(R)$	$E(U)$	σ_C	σ_R	σ_U	ρ_{CR}	ρ_{CU}	ρ_{RU}	c	r	u
10	20	1	0.8	0.5	0.5	0.30	0.30	0.30	0.25	0.50	0.50	0.072	-0.027	-0.005
10	20	1	0.8	0.5	0.5	0.30	0.30	0.30	0.50	0.00	0.00	0.025	0.025	0.015
10	20	1	0.8	0.5	0.5	0.30	0.30	0.30	0.50	0.00	0.25	0.027	0.035	0.019
10	20	1	0.8	0.5	0.5	0.30	0.30	0.30	0.50	0.00	0.50	0.027	0.033	0.020
10	20	1	0.8	0.5	0.5	0.30	0.30	0.30	0.50	0.25	0.00	0.053	0.014	0.011
10	20	1	0.8	0.5	0.5	0.30	0.30	0.30	0.50	0.25	0.25	0.047	0.018	0.010
10	20	1	0.8	0.5	0.5	0.30	0.30	0.30	0.50	0.25	0.50	0.045	0.013	0.012
10	20	1	0.8	0.5	0.5	0.30	0.30	0.30	0.50	0.50	0.00	0.073	-0.002	-0.002
10	20	1	0.8	0.5	0.5	0.30	0.30	0.30	0.50	0.50	0.25	0.077	-0.006	0.006
10	20	1	0.8	0.5	0.5	0.30	0.30	0.30	0.50	0.50	0.50	0.071	0.000	0.006
10	20	1	0.8	0.8	0.5	0.00	0.00	0.00	0.00	0.00	0.00	-0.002	0.001	-0.001
10	20	1	0.8	0.8	0.5	0.15	0.15	0.15	0.00	0.00	0.00	0.004	-0.004	0.004
10	20	1	0.8	0.8	0.5	0.15	0.15	0.15	0.00	0.00	0.25	0.007	-0.008	0.004
10	20	1	0.8	0.8	0.5	0.15	0.15	0.15	0.00	0.00	0.50	0.004	-0.006	0.003
10	20	1	0.8	0.8	0.5	0.15	0.15	0.15	0.00	0.25	0.00	0.013	-0.011	0.002
10	20	1	0.8	0.8	0.5	0.15	0.15	0.15	0.00	0.25	0.25	0.010	-0.011	0.001
10	20	1	0.8	0.8	0.5	0.15	0.15	0.15	0.00	0.25	0.50	0.011	-0.009	0.004
10	20	1	0.8	0.8	0.5	0.15	0.15	0.15	0.00	0.50	0.00	0.018	-0.016	0.002
10	20	1	0.8	0.8	0.5	0.15	0.15	0.15	0.00	0.50	0.25	0.019	-0.018	0.000
10	20	1	0.8	0.8	0.5	0.15	0.15	0.15	0.00	0.50	0.50	0.020	-0.015	0.002
10	20	1	0.8	0.8	0.5	0.15	0.15	0.15	0.25	0.00	0.00	0.005	-0.001	0.004
10	20	1	0.8	0.8	0.5	0.15	0.15	0.15	0.25	0.00	0.25	0.004	-0.002	0.002
10	20	1	0.8	0.8	0.5	0.15	0.15	0.15	0.25	0.00	0.50	0.010	0.001	0.003
10	20	1	0.8	0.8	0.5	0.15	0.15	0.15	0.25	0.25	0.00	0.013	-0.006	0.002
10	20	1	0.8	0.8	0.5	0.15	0.15	0.15	0.25	0.25	0.25	0.014	-0.008	-0.001
10	20	1	0.8	0.8	0.5	0.15	0.15	0.15	0.25	0.25	0.50	0.013	-0.003	0.000
10	20	1	0.8	0.8	0.5	0.15	0.15	0.15	0.25	0.50	0.00	0.020	-0.012	0.000
10	20	1	0.8	0.8	0.5	0.15	0.15	0.15	0.25	0.50	0.25	0.022	-0.012	0.003
10	20	1	0.8	0.8	0.5	0.15	0.15	0.15	0.25	0.50	0.50	0.020	-0.011	-0.001
10	20	1	0.8	0.8	0.5	0.15	0.15	0.15	0.50	0.00	0.00	0.006	0.007	0.004
10	20	1	0.8	0.8	0.5	0.15	0.15	0.15	0.50	0.00	0.25	0.004	0.006	0.005
10	20	1	0.8	0.8	0.5	0.15	0.15	0.15	0.50	0.00	0.50	0.007	0.008	0.008
10	20	1	0.8	0.8	0.5	0.15	0.15	0.15	0.50	0.25	0.00	0.012	0.001	0.002
10	20	1	0.8	0.8	0.5	0.15	0.15	0.15	0.50	0.25	0.25	0.015	0.002	0.001
10	20	1	0.8	0.8	0.5	0.15	0.15	0.15	0.50	0.25	0.50	0.014	0.004	0.002
10	20	1	0.8	0.8	0.5	0.15	0.15	0.15	0.50	0.50	0.00	0.021	-0.007	0.002
10	20	1	0.8	0.8	0.5	0.15	0.15	0.15	0.50	0.50	0.25	0.017	-0.006	-0.002
10	20	1	0.8	0.8	0.5	0.15	0.15	0.15	0.50	0.50	0.50	0.017	0.000	0.001
10	20	1	0.8	0.8	0.5	0.30	0.30	0.30	0.00	0.00	0.00	0.031	-0.025	0.020

(continued)

												Mean Bias		
N	m_1	$\frac{m_2}{m_1}$	$E(C)$	$E(R)$	$E(U)$	σ_C	σ_R	σ_U	ρ_{CR}	ρ_{CU}	ρ_{RU}	c	r	u
10	20	1	0.8	0.8	0.5	0.30	0.30	0.30	0.00	0.00	0.25	0.025	-0.032	0.020
10	20	1	0.8	0.8	0.5	0.30	0.30	0.30	0.00	0.00	0.50	0.025	-0.028	0.018
10	20	1	0.8	0.8	0.5	0.30	0.30	0.30	0.00	0.25	0.00	0.047	-0.050	0.011
10	20	1	0.8	0.8	0.5	0.30	0.30	0.30	0.00	0.25	0.25	0.055	-0.040	0.009
10	20	1	0.8	0.8	0.5	0.30	0.30	0.30	0.00	0.25	0.50	0.052	-0.044	0.012
10	20	1	0.8	0.8	0.5	0.30	0.30	0.30	0.00	0.50	0.00	0.072	-0.071	-0.003
10	20	1	0.8	0.8	0.5	0.30	0.30	0.30	0.00	0.50	0.25	0.072	-0.063	0.003
10	20	1	0.8	0.8	0.5	0.30	0.30	0.30	0.00	0.50	0.50	0.076	-0.069	0.000
10	20	1	0.8	0.8	0.5	0.30	0.30	0.30	0.25	0.00	0.00	0.025	-0.007	0.024
10	20	1	0.8	0.8	0.5	0.30	0.30	0.30	0.25	0.00	0.25	0.029	-0.004	0.022
10	20	1	0.8	0.8	0.5	0.30	0.30	0.30	0.25	0.00	0.50	0.024	-0.003	0.017
10	20	1	0.8	0.8	0.5	0.30	0.30	0.30	0.25	0.25	0.00	0.048	-0.028	0.009
10	20	1	0.8	0.8	0.5	0.30	0.30	0.30	0.25	0.25	0.25	0.046	-0.023	0.009
10	20	1	0.8	0.8	0.5	0.30	0.30	0.30	0.25	0.25	0.50	0.046	-0.032	0.010
10	20	1	0.8	0.8	0.5	0.30	0.30	0.30	0.25	0.50	0.00	0.073	-0.052	0.001
10	20	1	0.8	0.8	0.5	0.30	0.30	0.30	0.25	0.50	0.25	0.073	-0.047	-0.005
10	20	1	0.8	0.8	0.5	0.30	0.30	0.30	0.25	0.50	0.50	0.068	-0.053	-0.004
10	20	1	0.8	0.8	0.5	0.30	0.30	0.30	0.50	0.00	0.00	0.030	0.016	0.020
10	20	1	0.8	0.8	0.5	0.30	0.30	0.30	0.50	0.00	0.25	0.026	0.021	0.017
10	20	1	0.8	0.8	0.5	0.30	0.30	0.30	0.50	0.00	0.50	0.026	0.018	0.022
10	20	1	0.8	0.8	0.5	0.30	0.30	0.30	0.50	0.25	0.00	0.048	-0.007	0.010
10	20	1	0.8	0.8	0.5	0.30	0.30	0.30	0.50	0.25	0.25	0.049	-0.004	0.015
10	20	1	0.8	0.8	0.5	0.30	0.30	0.30	0.50	0.25	0.50	0.052	-0.004	0.009
10	20	1	0.8	0.8	0.5	0.30	0.30	0.30	0.50	0.50	0.00	0.073	-0.025	0.002
10	20	1	0.8	0.8	0.5	0.30	0.30	0.30	0.50	0.50	0.25	0.073	-0.025	0.001
10	20	1	0.8	0.8	0.5	0.30	0.30	0.30	0.50	0.50	0.50	0.072	-0.031	0.000
1000	4	0	0.2	0.2	0.5	0.00	0.00	0.00	0.00	0.00	0.00	0.000	0.002	0.000
1000	4	0	0.2	0.2	0.5	0.15	0.15	0.15	0.00	0.00	0.00	0.066	-0.049	0.045
1000	4	0	0.2	0.2	0.5	0.15	0.15	0.15	0.00	0.00	0.25	0.066	-0.049	0.045
1000	4	0	0.2	0.2	0.5	0.15	0.15	0.15	0.00	0.00	0.50	0.066	-0.050	0.045
1000	4	0	0.2	0.2	0.5	0.15	0.15	0.15	0.00	0.25	0.00	0.067	-0.050	0.038
1000	4	0	0.2	0.2	0.5	0.15	0.15	0.15	0.00	0.25	0.25	0.067	-0.050	0.038
1000	4	0	0.2	0.2	0.5	0.15	0.15	0.15	0.00	0.25	0.50	0.067	-0.050	0.039
1000	4	0	0.2	0.2	0.5	0.15	0.15	0.15	0.00	0.50	0.00	0.068	-0.051	0.032
1000	4	0	0.2	0.2	0.5	0.15	0.15	0.15	0.00	0.50	0.25	0.067	-0.050	0.032
1000	4	0	0.2	0.2	0.5	0.15	0.15	0.15	0.00	0.50	0.50	0.067	-0.050	0.031
1000	4	0	0.2	0.2	0.5	0.15	0.15	0.15	0.25	0.00	0.00	0.066	-0.030	0.045
1000	4	0	0.2	0.2	0.5	0.15	0.15	0.15	0.25	0.00	0.25	0.065	-0.028	0.044
1000	4	0	0.2	0.2	0.5	0.15	0.15	0.15	0.25	0.00	0.50	0.065	-0.029	0.045

(continued)

												Mean Bias		
N	m_1	$\frac{m_2}{m_1}$	$E(C)$	$E(R)$	$E(U)$	σ_C	σ_R	σ_U	ρ_{CR}	ρ_{CU}	ρ_{RU}	c	r	u
1000	4	0	0.2	0.2	0.5	0.15	0.15	0.15	0.25	0.25	0.00	0.066	-0.030	0.038
1000	4	0	0.2	0.2	0.5	0.15	0.15	0.15	0.25	0.25	0.25	0.067	-0.030	0.039
1000	4	0	0.2	0.2	0.5	0.15	0.15	0.15	0.25	0.25	0.50	0.067	-0.031	0.038
1000	4	0	0.2	0.2	0.5	0.15	0.15	0.15	0.25	0.50	0.00	0.067	-0.031	0.032
1000	4	0	0.2	0.2	0.5	0.15	0.15	0.15	0.25	0.50	0.25	0.067	-0.030	0.031
1000	4	0	0.2	0.2	0.5	0.15	0.15	0.15	0.25	0.50	0.50	0.067	-0.030	0.031
1000	4	0	0.2	0.2	0.5	0.15	0.15	0.15	0.50	0.00	0.00	0.066	-0.009	0.045
1000	4	0	0.2	0.2	0.5	0.15	0.15	0.15	0.50	0.00	0.25	0.066	-0.009	0.045
1000	4	0	0.2	0.2	0.5	0.15	0.15	0.15	0.50	0.00	0.50	0.065	-0.008	0.044
1000	4	0	0.2	0.2	0.5	0.15	0.15	0.15	0.50	0.25	0.00	0.067	-0.010	0.039
1000	4	0	0.2	0.2	0.5	0.15	0.15	0.15	0.50	0.25	0.25	0.067	-0.009	0.038
1000	4	0	0.2	0.2	0.5	0.15	0.15	0.15	0.50	0.25	0.50	0.067	-0.010	0.039
1000	4	0	0.2	0.2	0.5	0.15	0.15	0.15	0.50	0.50	0.00	0.068	-0.010	0.031
1000	4	0	0.2	0.2	0.5	0.15	0.15	0.15	0.50	0.50	0.25	0.067	-0.010	0.031
1000	4	0	0.2	0.2	0.5	0.15	0.15	0.15	0.50	0.50	0.50	0.067	-0.010	0.031
1000	4	0	0.2	0.2	0.5	0.30	0.30	0.30	0.00	0.00	0.00	0.211	-0.103	0.180
1000	4	0	0.2	0.2	0.5	0.30	0.30	0.30	0.00	0.00	0.25	0.212	-0.103	0.180
1000	4	0	0.2	0.2	0.5	0.30	0.30	0.30	0.00	0.00	0.50	0.212	-0.103	0.179
1000	4	0	0.2	0.2	0.5	0.30	0.30	0.30	0.00	0.25	0.00	0.224	-0.106	0.163
1000	4	0	0.2	0.2	0.5	0.30	0.30	0.30	0.00	0.25	0.25	0.224	-0.106	0.163
1000	4	0	0.2	0.2	0.5	0.30	0.30	0.30	0.00	0.25	0.50	0.223	-0.106	0.163
1000	4	0	0.2	0.2	0.5	0.30	0.30	0.30	0.00	0.50	0.00	0.233	-0.108	0.141
1000	4	0	0.2	0.2	0.5	0.30	0.30	0.30	0.00	0.50	0.25	0.233	-0.108	0.140
1000	4	0	0.2	0.2	0.5	0.30	0.30	0.30	0.00	0.50	0.50	0.233	-0.108	0.141
1000	4	0	0.2	0.2	0.5	0.30	0.30	0.30	0.25	0.00	0.00	0.212	-0.062	0.180
1000	4	0	0.2	0.2	0.5	0.30	0.30	0.30	0.25	0.00	0.25	0.211	-0.062	0.180
1000	4	0	0.2	0.2	0.5	0.30	0.30	0.30	0.25	0.00	0.50	0.211	-0.062	0.180
1000	4	0	0.2	0.2	0.5	0.30	0.30	0.30	0.25	0.25	0.00	0.224	-0.066	0.163
1000	4	0	0.2	0.2	0.5	0.30	0.30	0.30	0.25	0.25	0.25	0.225	-0.065	0.163
1000	4	0	0.2	0.2	0.5	0.30	0.30	0.30	0.25	0.25	0.50	0.223	-0.065	0.163
1000	4	0	0.2	0.2	0.5	0.30	0.30	0.30	0.25	0.50	0.00	0.233	-0.069	0.141
1000	4	0	0.2	0.2	0.5	0.30	0.30	0.30	0.25	0.50	0.25	0.232	-0.068	0.140
1000	4	0	0.2	0.2	0.5	0.30	0.30	0.30	0.25	0.50	0.50	0.232	-0.068	0.141
1000	4	0	0.2	0.2	0.5	0.30	0.30	0.30	0.50	0.00	0.00	0.213	-0.014	0.179
1000	4	0	0.2	0.2	0.5	0.30	0.30	0.30	0.50	0.00	0.25	0.212	-0.014	0.180
1000	4	0	0.2	0.2	0.5	0.30	0.30	0.30	0.50	0.00	0.50	0.212	-0.014	0.180
1000	4	0	0.2	0.2	0.5	0.30	0.30	0.30	0.50	0.25	0.00	0.224	-0.019	0.163
1000	4	0	0.2	0.2	0.5	0.30	0.30	0.30	0.50	0.25	0.25	0.224	-0.020	0.163
1000	4	0	0.2	0.2	0.5	0.30	0.30	0.30	0.50	0.25	0.50	0.224	-0.019	0.163

(continued)

												Mean Bias		
N	m_1	$\frac{m_2}{m_1}$	$E(C)$	$E(R)$	$E(U)$	σ_C	σ_R	σ_U	ρ_{CR}	ρ_{CU}	ρ_{RU}	c	r	u
1000	4	0	0.2	0.2	0.5	0.30	0.30	0.30	0.50	0.50	0.00	0.233	-0.023	0.141
1000	4	0	0.2	0.2	0.5	0.30	0.30	0.30	0.50	0.50	0.25	0.233	-0.024	0.140
1000	4	0	0.2	0.2	0.5	0.30	0.30	0.30	0.50	0.50	0.50	0.233	-0.023	0.140
1000	4	0	0.2	0.5	0.5	0.00	0.00	0.00	0.00	0.00	0.00	-0.001	0.004	0.000
1000	4	0	0.2	0.5	0.5	0.15	0.15	0.15	0.00	0.00	0.00	0.066	-0.122	0.044
1000	4	0	0.2	0.5	0.5	0.15	0.15	0.15	0.00	0.00	0.25	0.066	-0.123	0.045
1000	4	0	0.2	0.5	0.5	0.15	0.15	0.15	0.00	0.00	0.50	0.066	-0.123	0.045
1000	4	0	0.2	0.5	0.5	0.15	0.15	0.15	0.00	0.25	0.00	0.068	-0.125	0.039
1000	4	0	0.2	0.5	0.5	0.15	0.15	0.15	0.00	0.25	0.25	0.067	-0.124	0.038
1000	4	0	0.2	0.5	0.5	0.15	0.15	0.15	0.00	0.25	0.50	0.066	-0.126	0.039
1000	4	0	0.2	0.5	0.5	0.15	0.15	0.15	0.00	0.50	0.00	0.067	-0.125	0.032
1000	4	0	0.2	0.5	0.5	0.15	0.15	0.15	0.00	0.50	0.25	0.068	-0.126	0.031
1000	4	0	0.2	0.5	0.5	0.15	0.15	0.15	0.00	0.50	0.50	0.067	-0.124	0.031
1000	4	0	0.2	0.5	0.5	0.15	0.15	0.15	0.25	0.00	0.00	0.065	-0.103	0.045
1000	4	0	0.2	0.5	0.5	0.15	0.15	0.15	0.25	0.00	0.25	0.065	-0.104	0.044
1000	4	0	0.2	0.5	0.5	0.15	0.15	0.15	0.25	0.00	0.50	0.065	-0.101	0.044
1000	4	0	0.2	0.5	0.5	0.15	0.15	0.15	0.25	0.25	0.00	0.066	-0.104	0.038
1000	4	0	0.2	0.5	0.5	0.15	0.15	0.15	0.25	0.25	0.25	0.067	-0.105	0.039
1000	4	0	0.2	0.5	0.5	0.15	0.15	0.15	0.25	0.25	0.50	0.066	-0.104	0.038
1000	4	0	0.2	0.5	0.5	0.15	0.15	0.15	0.25	0.50	0.00	0.068	-0.106	0.032
1000	4	0	0.2	0.5	0.5	0.15	0.15	0.15	0.25	0.50	0.25	0.067	-0.105	0.031
1000	4	0	0.2	0.5	0.5	0.15	0.15	0.15	0.25	0.50	0.50	0.067	-0.104	0.031
1000	4	0	0.2	0.5	0.5	0.15	0.15	0.15	0.50	0.00	0.00	0.066	-0.083	0.045
1000	4	0	0.2	0.5	0.5	0.15	0.15	0.15	0.50	0.00	0.25	0.065	-0.083	0.044
1000	4	0	0.2	0.5	0.5	0.15	0.15	0.15	0.50	0.00	0.50	0.066	-0.082	0.045
1000	4	0	0.2	0.5	0.5	0.15	0.15	0.15	0.50	0.25	0.00	0.066	-0.084	0.038
1000	4	0	0.2	0.5	0.5	0.15	0.15	0.15	0.50	0.25	0.25	0.067	-0.084	0.038
1000	4	0	0.2	0.5	0.5	0.15	0.15	0.15	0.50	0.25	0.50	0.067	-0.086	0.039
1000	4	0	0.2	0.5	0.5	0.15	0.15	0.15	0.50	0.50	0.00	0.068	-0.086	0.031
1000	4	0	0.2	0.5	0.5	0.15	0.15	0.15	0.50	0.50	0.25	0.068	-0.085	0.032
1000	4	0	0.2	0.5	0.5	0.15	0.15	0.15	0.50	0.50	0.50	0.067	-0.086	0.032
1000	4	0	0.2	0.5	0.5	0.30	0.30	0.30	0.00	0.00	0.00	0.211	-0.257	0.179
1000	4	0	0.2	0.5	0.5	0.30	0.30	0.30	0.00	0.00	0.25	0.211	-0.256	0.180
1000	4	0	0.2	0.5	0.5	0.30	0.30	0.30	0.00	0.00	0.50	0.211	-0.257	0.179
1000	4	0	0.2	0.5	0.5	0.30	0.30	0.30	0.00	0.25	0.00	0.224	-0.265	0.163
1000	4	0	0.2	0.5	0.5	0.30	0.30	0.30	0.00	0.25	0.25	0.224	-0.264	0.163
1000	4	0	0.2	0.5	0.5	0.30	0.30	0.30	0.00	0.25	0.50	0.224	-0.264	0.163
1000	4	0	0.2	0.5	0.5	0.30	0.30	0.30	0.00	0.50	0.00	0.233	-0.269	0.141
1000	4	0	0.2	0.5	0.5	0.30	0.30	0.30	0.00	0.50	0.25	0.233	-0.269	0.141

(continued)

N	m_1	$\frac{m_2}{m_1}$	$E(C)$	$E(R)$	$E(U)$	σ_C	σ_R	σ_U	ρ_{CR}	ρ_{CU}	ρ_{RU}	Mean Bias c	r	u
1000	4	0	0.2	0.5	0.5	0.30	0.30	0.30	0.00	0.50	0.50	0.233	-0.269	0.142
1000	4	0	0.2	0.5	0.5	0.30	0.30	0.30	0.25	0.00	0.00	0.211	-0.212	0.180
1000	4	0	0.2	0.5	0.5	0.30	0.30	0.30	0.25	0.00	0.25	0.212	-0.212	0.181
1000	4	0	0.2	0.5	0.5	0.30	0.30	0.30	0.25	0.00	0.50	0.212	-0.213	0.180
1000	4	0	0.2	0.5	0.5	0.30	0.30	0.30	0.25	0.25	0.00	0.224	-0.220	0.162
1000	4	0	0.2	0.5	0.5	0.30	0.30	0.30	0.25	0.25	0.25	0.224	-0.221	0.164
1000	4	0	0.2	0.5	0.5	0.30	0.30	0.30	0.25	0.25	0.50	0.225	-0.222	0.163
1000	4	0	0.2	0.5	0.5	0.30	0.30	0.30	0.25	0.50	0.00	0.233	-0.227	0.140
1000	4	0	0.2	0.5	0.5	0.30	0.30	0.30	0.25	0.50	0.25	0.234	-0.226	0.140
1000	4	0	0.2	0.5	0.5	0.30	0.30	0.30	0.25	0.50	0.50	0.232	-0.228	0.141
1000	4	0	0.2	0.5	0.5	0.30	0.30	0.30	0.50	0.00	0.00	0.211	-0.167	0.179
1000	4	0	0.2	0.5	0.5	0.30	0.30	0.30	0.50	0.00	0.25	0.212	-0.167	0.179
1000	4	0	0.2	0.5	0.5	0.30	0.30	0.30	0.50	0.00	0.50	0.212	-0.167	0.180
1000	4	0	0.2	0.5	0.5	0.30	0.30	0.30	0.50	0.25	0.00	0.224	-0.178	0.163
1000	4	0	0.2	0.5	0.5	0.30	0.30	0.30	0.50	0.25	0.25	0.224	-0.177	0.163
1000	4	0	0.2	0.5	0.5	0.30	0.30	0.30	0.50	0.25	0.50	0.225	-0.177	0.163
1000	4	0	0.2	0.5	0.5	0.30	0.30	0.30	0.50	0.50	0.00	0.234	-0.185	0.141
1000	4	0	0.2	0.5	0.5	0.30	0.30	0.30	0.50	0.50	0.25	0.233	-0.184	0.141
1000	4	0	0.2	0.5	0.5	0.30	0.30	0.30	0.50	0.50	0.50	0.234	-0.184	0.140
1000	4	0	0.2	0.8	0.5	0.00	0.00	0.00	0.00	0.00	0.00	0.000	0.003	0.000
1000	4	0	0.2	0.8	0.5	0.15	0.15	0.15	0.00	0.00	0.00	0.065	-0.196	0.044
1000	4	0	0.2	0.8	0.5	0.15	0.15	0.15	0.00	0.00	0.25	0.066	-0.198	0.045
1000	4	0	0.2	0.8	0.5	0.15	0.15	0.15	0.00	0.00	0.50	0.065	-0.197	0.045
1000	4	0	0.2	0.8	0.5	0.15	0.15	0.15	0.00	0.25	0.00	0.067	-0.200	0.039
1000	4	0	0.2	0.8	0.5	0.15	0.15	0.15	0.00	0.25	0.25	0.067	-0.201	0.039
1000	4	0	0.2	0.8	0.5	0.15	0.15	0.15	0.00	0.25	0.50	0.067	-0.200	0.038
1000	4	0	0.2	0.8	0.5	0.15	0.15	0.15	0.00	0.50	0.00	0.067	-0.200	0.031
1000	4	0	0.2	0.8	0.5	0.15	0.15	0.15	0.00	0.50	0.25	0.067	-0.200	0.032
1000	4	0	0.2	0.8	0.5	0.15	0.15	0.15	0.00	0.50	0.50	0.067	-0.201	0.032
1000	4	0	0.2	0.8	0.5	0.15	0.15	0.15	0.25	0.00	0.00	0.065	-0.177	0.044
1000	4	0	0.2	0.8	0.5	0.15	0.15	0.15	0.25	0.00	0.25	0.065	-0.176	0.044
1000	4	0	0.2	0.8	0.5	0.15	0.15	0.15	0.25	0.00	0.50	0.065	-0.179	0.045
1000	4	0	0.2	0.8	0.5	0.15	0.15	0.15	0.25	0.25	0.00	0.067	-0.180	0.039
1000	4	0	0.2	0.8	0.5	0.15	0.15	0.15	0.25	0.25	0.25	0.067	-0.182	0.039
1000	4	0	0.2	0.8	0.5	0.15	0.15	0.15	0.25	0.25	0.50	0.067	-0.181	0.038
1000	4	0	0.2	0.8	0.5	0.15	0.15	0.15	0.25	0.50	0.00	0.067	-0.183	0.032
1000	4	0	0.2	0.8	0.5	0.15	0.15	0.15	0.25	0.50	0.25	0.067	-0.182	0.031
1000	4	0	0.2	0.8	0.5	0.15	0.15	0.15	0.25	0.50	0.50	0.068	-0.183	0.032
1000	4	0	0.2	0.8	0.5	0.15	0.15	0.15	0.50	0.00	0.00	0.065	-0.159	0.045

(continued)

N	m_1	$\frac{m_2}{m_1}$	$E(C)$	$E(R)$	$E(U)$	σ_C	σ_R	σ_U	ρ_{CR}	ρ_{CU}	ρ_{RU}	Mean Bias c	Mean Bias r	Mean Bias u
1000	4	0	0.2	0.8	0.5	0.15	0.15	0.15	0.50	0.00	0.25	0.065	-0.160	0.044
1000	4	0	0.2	0.8	0.5	0.15	0.15	0.15	0.50	0.00	0.50	0.066	-0.160	0.045
1000	4	0	0.2	0.8	0.5	0.15	0.15	0.15	0.50	0.25	0.00	0.067	-0.162	0.039
1000	4	0	0.2	0.8	0.5	0.15	0.15	0.15	0.50	0.25	0.25	0.067	-0.162	0.038
1000	4	0	0.2	0.8	0.5	0.15	0.15	0.15	0.50	0.25	0.50	0.067	-0.163	0.039
1000	4	0	0.2	0.8	0.5	0.15	0.15	0.15	0.50	0.50	0.00	0.068	-0.164	0.032
1000	4	0	0.2	0.8	0.5	0.15	0.15	0.15	0.50	0.50	0.25	0.068	-0.165	0.032
1000	4	0	0.2	0.8	0.5	0.15	0.15	0.15	0.50	0.50	0.50	0.068	-0.166	0.032
1000	4	0	0.2	0.8	0.5	0.30	0.30	0.30	0.00	0.00	0.00	0.212	-0.412	0.180
1000	4	0	0.2	0.8	0.5	0.30	0.30	0.30	0.00	0.00	0.25	0.211	-0.410	0.180
1000	4	0	0.2	0.8	0.5	0.30	0.30	0.30	0.00	0.00	0.50	0.211	-0.411	0.180
1000	4	0	0.2	0.8	0.5	0.30	0.30	0.30	0.00	0.25	0.00	0.224	-0.423	0.163
1000	4	0	0.2	0.8	0.5	0.30	0.30	0.30	0.00	0.25	0.25	0.224	-0.423	0.163
1000	4	0	0.2	0.8	0.5	0.30	0.30	0.30	0.00	0.25	0.50	0.224	-0.423	0.163
1000	4	0	0.2	0.8	0.5	0.30	0.30	0.30	0.00	0.50	0.00	0.233	-0.431	0.141
1000	4	0	0.2	0.8	0.5	0.30	0.30	0.30	0.00	0.50	0.25	0.234	-0.430	0.141
1000	4	0	0.2	0.8	0.5	0.30	0.30	0.30	0.00	0.50	0.50	0.233	-0.429	0.140
1000	4	0	0.2	0.8	0.5	0.30	0.30	0.30	0.25	0.00	0.00	0.211	-0.376	0.179
1000	4	0	0.2	0.8	0.5	0.30	0.30	0.30	0.25	0.00	0.25	0.212	-0.375	0.180
1000	4	0	0.2	0.8	0.5	0.30	0.30	0.30	0.25	0.00	0.50	0.212	-0.376	0.180
1000	4	0	0.2	0.8	0.5	0.30	0.30	0.30	0.25	0.25	0.00	0.224	-0.389	0.163
1000	4	0	0.2	0.8	0.5	0.30	0.30	0.30	0.25	0.25	0.25	0.224	-0.388	0.163
1000	4	0	0.2	0.8	0.5	0.30	0.30	0.30	0.25	0.25	0.50	0.223	-0.388	0.163
1000	4	0	0.2	0.8	0.5	0.30	0.30	0.30	0.25	0.50	0.00	0.233	-0.397	0.140
1000	4	0	0.2	0.8	0.5	0.30	0.30	0.30	0.25	0.50	0.25	0.233	-0.397	0.141
1000	4	0	0.2	0.8	0.5	0.30	0.30	0.30	0.25	0.50	0.50	0.233	-0.398	0.140
1000	4	0	0.2	0.8	0.5	0.30	0.30	0.30	0.50	0.00	0.00	0.211	-0.346	0.180
1000	4	0	0.2	0.8	0.5	0.30	0.30	0.30	0.50	0.00	0.25	0.212	-0.345	0.179
1000	4	0	0.2	0.8	0.5	0.30	0.30	0.30	0.50	0.00	0.50	0.212	-0.347	0.180
1000	4	0	0.2	0.8	0.5	0.30	0.30	0.30	0.50	0.25	0.00	0.225	-0.360	0.163
1000	4	0	0.2	0.8	0.5	0.30	0.30	0.30	0.50	0.25	0.25	0.225	-0.360	0.163
1000	4	0	0.2	0.8	0.5	0.30	0.30	0.30	0.50	0.25	0.50	0.225	-0.360	0.163
1000	4	0	0.2	0.8	0.5	0.30	0.30	0.30	0.50	0.50	0.00	0.233	-0.369	0.142
1000	4	0	0.2	0.8	0.5	0.30	0.30	0.30	0.50	0.50	0.25	0.234	-0.368	0.141
1000	4	0	0.2	0.8	0.5	0.30	0.30	0.30	0.50	0.50	0.50	0.233	-0.368	0.142
1000	4	0	0.5	0.2	0.5	0.00	0.00	0.00	0.00	0.00	0.00	0.000	0.000	0.000
1000	4	0	0.5	0.2	0.5	0.15	0.15	0.15	0.00	0.00	0.00	0.041	-0.015	0.045
1000	4	0	0.5	0.2	0.5	0.15	0.15	0.15	0.00	0.00	0.25	0.041	-0.015	0.046
1000	4	0	0.5	0.2	0.5	0.15	0.15	0.15	0.00	0.00	0.50	0.040	-0.015	0.044

(continued)

												Mean Bias		
N	m_1	$\frac{m_2}{m_1}$	$E(C)$	$E(R)$	$E(U)$	σ_C	σ_R	σ_U	ρ_{CR}	ρ_{CU}	ρ_{RU}	c	r	u
1000	4	0	0.5	0.2	0.5	0.15	0.15	0.15	0.00	0.25	0.00	0.043	-0.016	0.035
1000	4	0	0.5	0.2	0.5	0.15	0.15	0.15	0.00	0.25	0.25	0.042	-0.016	0.034
1000	4	0	0.5	0.2	0.5	0.15	0.15	0.15	0.00	0.25	0.50	0.043	-0.016	0.035
1000	4	0	0.5	0.2	0.5	0.15	0.15	0.15	0.00	0.50	0.00	0.044	-0.016	0.024
1000	4	0	0.5	0.2	0.5	0.15	0.15	0.15	0.00	0.50	0.25	0.044	-0.016	0.023
1000	4	0	0.5	0.2	0.5	0.15	0.15	0.15	0.00	0.50	0.50	0.043	-0.016	0.024
1000	4	0	0.5	0.2	0.5	0.15	0.15	0.15	0.25	0.00	0.00	0.041	-0.006	0.045
1000	4	0	0.5	0.2	0.5	0.15	0.15	0.15	0.25	0.00	0.25	0.042	-0.005	0.045
1000	4	0	0.5	0.2	0.5	0.15	0.15	0.15	0.25	0.00	0.50	0.041	-0.006	0.044
1000	4	0	0.5	0.2	0.5	0.15	0.15	0.15	0.25	0.25	0.00	0.042	-0.006	0.035
1000	4	0	0.5	0.2	0.5	0.15	0.15	0.15	0.25	0.25	0.25	0.042	-0.006	0.034
1000	4	0	0.5	0.2	0.5	0.15	0.15	0.15	0.25	0.25	0.50	0.042	-0.005	0.035
1000	4	0	0.5	0.2	0.5	0.15	0.15	0.15	0.25	0.50	0.00	0.044	-0.006	0.024
1000	4	0	0.5	0.2	0.5	0.15	0.15	0.15	0.25	0.50	0.25	0.044	-0.006	0.023
1000	4	0	0.5	0.2	0.5	0.15	0.15	0.15	0.25	0.50	0.50	0.043	-0.006	0.024
1000	4	0	0.5	0.2	0.5	0.15	0.15	0.15	0.50	0.00	0.00	0.041	0.005	0.045
1000	4	0	0.5	0.2	0.5	0.15	0.15	0.15	0.50	0.00	0.25	0.041	0.005	0.045
1000	4	0	0.5	0.2	0.5	0.15	0.15	0.15	0.50	0.00	0.50	0.040	0.005	0.045
1000	4	0	0.5	0.2	0.5	0.15	0.15	0.15	0.50	0.25	0.00	0.042	0.004	0.036
1000	4	0	0.5	0.2	0.5	0.15	0.15	0.15	0.50	0.25	0.25	0.042	0.005	0.034
1000	4	0	0.5	0.2	0.5	0.15	0.15	0.15	0.50	0.25	0.50	0.043	0.004	0.035
1000	4	0	0.5	0.2	0.5	0.15	0.15	0.15	0.50	0.50	0.00	0.044	0.003	0.024
1000	4	0	0.5	0.2	0.5	0.15	0.15	0.15	0.50	0.50	0.25	0.044	0.004	0.024
1000	4	0	0.5	0.2	0.5	0.15	0.15	0.15	0.50	0.50	0.50	0.044	0.004	0.023
1000	4	0	0.5	0.2	0.5	0.30	0.30	0.30	0.00	0.00	0.00	0.132	-0.041	0.180
1000	4	0	0.5	0.2	0.5	0.30	0.30	0.30	0.00	0.00	0.25	0.132	-0.042	0.180
1000	4	0	0.5	0.2	0.5	0.30	0.30	0.30	0.00	0.00	0.50	0.132	-0.042	0.180
1000	4	0	0.5	0.2	0.5	0.30	0.30	0.30	0.00	0.25	0.00	0.148	-0.046	0.149
1000	4	0	0.5	0.2	0.5	0.30	0.30	0.30	0.00	0.25	0.25	0.148	-0.046	0.150
1000	4	0	0.5	0.2	0.5	0.30	0.30	0.30	0.00	0.25	0.50	0.148	-0.046	0.150
1000	4	0	0.5	0.2	0.5	0.30	0.30	0.30	0.00	0.50	0.00	0.162	-0.049	0.112
1000	4	0	0.5	0.2	0.5	0.30	0.30	0.30	0.00	0.50	0.25	0.162	-0.049	0.112
1000	4	0	0.5	0.2	0.5	0.30	0.30	0.30	0.00	0.50	0.50	0.163	-0.048	0.114
1000	4	0	0.5	0.2	0.5	0.30	0.30	0.30	0.25	0.00	0.00	0.132	-0.013	0.181
1000	4	0	0.5	0.2	0.5	0.30	0.30	0.30	0.25	0.00	0.25	0.133	-0.014	0.180
1000	4	0	0.5	0.2	0.5	0.30	0.30	0.30	0.25	0.00	0.50	0.132	-0.013	0.180
1000	4	0	0.5	0.2	0.5	0.30	0.30	0.30	0.25	0.25	0.00	0.148	-0.018	0.149
1000	4	0	0.5	0.2	0.5	0.30	0.30	0.30	0.25	0.25	0.25	0.148	-0.017	0.150
1000	4	0	0.5	0.2	0.5	0.30	0.30	0.30	0.25	0.25	0.50	0.148	-0.017	0.150

(continued)

												Mean Bias		
N	m_1	$\frac{m_2}{m_1}$	$E(C)$	$E(R)$	$E(U)$	σ_C	σ_R	σ_U	ρ_{CR}	ρ_{CU}	ρ_{RU}	c	r	u
1000	4	0	0.5	0.2	0.5	0.30	0.30	0.30	0.25	0.50	0.00	0.162	-0.021	0.112
1000	4	0	0.5	0.2	0.5	0.30	0.30	0.30	0.25	0.50	0.25	0.162	-0.021	0.113
1000	4	0	0.5	0.2	0.5	0.30	0.30	0.30	0.25	0.50	0.50	0.163	-0.022	0.112
1000	4	0	0.5	0.2	0.5	0.30	0.30	0.30	0.50	0.00	0.00	0.132	0.016	0.180
1000	4	0	0.5	0.2	0.5	0.30	0.30	0.30	0.50	0.00	0.25	0.132	0.016	0.181
1000	4	0	0.5	0.2	0.5	0.30	0.30	0.30	0.50	0.00	0.50	0.132	0.016	0.179
1000	4	0	0.5	0.2	0.5	0.30	0.30	0.30	0.50	0.25	0.00	0.148	0.011	0.150
1000	4	0	0.5	0.2	0.5	0.30	0.30	0.30	0.50	0.25	0.25	0.149	0.011	0.151
1000	4	0	0.5	0.2	0.5	0.30	0.30	0.30	0.50	0.25	0.50	0.148	0.011	0.151
1000	4	0	0.5	0.2	0.5	0.30	0.30	0.30	0.50	0.50	0.00	0.162	0.007	0.112
1000	4	0	0.5	0.2	0.5	0.30	0.30	0.30	0.50	0.50	0.25	0.163	0.007	0.113
1000	4	0	0.5	0.2	0.5	0.30	0.30	0.30	0.50	0.50	0.50	0.163	0.006	0.113
1000	4	0	0.5	0.5	0.5	0.00	0.00	0.00	0.00	0.00	0.00	0.000	0.000	0.000
1000	4	0	0.5	0.5	0.5	0.15	0.15	0.15	0.00	0.00	0.00	0.041	-0.037	0.044
1000	4	0	0.5	0.5	0.5	0.15	0.15	0.15	0.00	0.00	0.25	0.041	-0.038	0.045
1000	4	0	0.5	0.5	0.5	0.15	0.15	0.15	0.00	0.00	0.50	0.041	-0.038	0.045
1000	4	0	0.5	0.5	0.5	0.15	0.15	0.15	0.00	0.25	0.00	0.043	-0.039	0.034
1000	4	0	0.5	0.5	0.5	0.15	0.15	0.15	0.00	0.25	0.25	0.042	-0.040	0.034
1000	4	0	0.5	0.5	0.5	0.15	0.15	0.15	0.00	0.25	0.50	0.042	-0.039	0.034
1000	4	0	0.5	0.5	0.5	0.15	0.15	0.15	0.00	0.50	0.00	0.044	-0.040	0.023
1000	4	0	0.5	0.5	0.5	0.15	0.15	0.15	0.00	0.50	0.25	0.045	-0.040	0.024
1000	4	0	0.5	0.5	0.5	0.15	0.15	0.15	0.00	0.50	0.50	0.043	-0.040	0.022
1000	4	0	0.5	0.5	0.5	0.15	0.15	0.15	0.25	0.00	0.00	0.042	-0.028	0.045
1000	4	0	0.5	0.5	0.5	0.15	0.15	0.15	0.25	0.00	0.25	0.041	-0.027	0.045
1000	4	0	0.5	0.5	0.5	0.15	0.15	0.15	0.25	0.00	0.50	0.041	-0.027	0.044
1000	4	0	0.5	0.5	0.5	0.15	0.15	0.15	0.25	0.25	0.00	0.042	-0.028	0.034
1000	4	0	0.5	0.5	0.5	0.15	0.15	0.15	0.25	0.25	0.25	0.043	-0.029	0.035
1000	4	0	0.5	0.5	0.5	0.15	0.15	0.15	0.25	0.25	0.50	0.042	-0.028	0.034
1000	4	0	0.5	0.5	0.5	0.15	0.15	0.15	0.25	0.50	0.00	0.043	-0.030	0.023
1000	4	0	0.5	0.5	0.5	0.15	0.15	0.15	0.25	0.50	0.25	0.044	-0.029	0.023
1000	4	0	0.5	0.5	0.5	0.15	0.15	0.15	0.25	0.50	0.50	0.043	-0.029	0.024
1000	4	0	0.5	0.5	0.5	0.15	0.15	0.15	0.50	0.00	0.00	0.040	-0.017	0.044
1000	4	0	0.5	0.5	0.5	0.15	0.15	0.15	0.50	0.00	0.25	0.041	-0.018	0.045
1000	4	0	0.5	0.5	0.5	0.15	0.15	0.15	0.50	0.00	0.50	0.041	-0.017	0.045
1000	4	0	0.5	0.5	0.5	0.15	0.15	0.15	0.50	0.25	0.00	0.043	-0.019	0.034
1000	4	0	0.5	0.5	0.5	0.15	0.15	0.15	0.50	0.25	0.25	0.042	-0.018	0.035
1000	4	0	0.5	0.5	0.5	0.15	0.15	0.15	0.50	0.25	0.50	0.043	-0.019	0.036
1000	4	0	0.5	0.5	0.5	0.15	0.15	0.15	0.50	0.50	0.00	0.043	-0.019	0.023
1000	4	0	0.5	0.5	0.5	0.15	0.15	0.15	0.50	0.50	0.25	0.043	-0.020	0.023

(continued)

												Mean Bias		
N	m_1	$\frac{m_2}{m_1}$	$E(C)$	$E(R)$	$E(U)$	σ_C	σ_R	σ_U	ρ_{CR}	ρ_{CU}	ρ_{RU}	c	r	u
1000	4	0	0.5	0.5	0.5	0.15	0.15	0.15	0.50	0.50	0.50	0.043	-0.019	0.023
1000	4	0	0.5	0.5	0.5	0.30	0.30	0.30	0.00	0.00	0.00	0.132	-0.104	0.180
1000	4	0	0.5	0.5	0.5	0.30	0.30	0.30	0.00	0.00	0.25	0.133	-0.104	0.180
1000	4	0	0.5	0.5	0.5	0.30	0.30	0.30	0.00	0.00	0.50	0.132	-0.104	0.180
1000	4	0	0.5	0.5	0.5	0.30	0.30	0.30	0.00	0.25	0.00	0.148	-0.114	0.150
1000	4	0	0.5	0.5	0.5	0.30	0.30	0.30	0.00	0.25	0.25	0.148	-0.114	0.149
1000	4	0	0.5	0.5	0.5	0.30	0.30	0.30	0.00	0.25	0.50	0.148	-0.114	0.150
1000	4	0	0.5	0.5	0.5	0.30	0.30	0.30	0.00	0.50	0.00	0.162	-0.123	0.112
1000	4	0	0.5	0.5	0.5	0.30	0.30	0.30	0.00	0.50	0.25	0.162	-0.123	0.113
1000	4	0	0.5	0.5	0.5	0.30	0.30	0.30	0.00	0.50	0.50	0.163	-0.122	0.112
1000	4	0	0.5	0.5	0.5	0.30	0.30	0.30	0.25	0.00	0.00	0.133	-0.071	0.180
1000	4	0	0.5	0.5	0.5	0.30	0.30	0.30	0.25	0.00	0.25	0.133	-0.071	0.180
1000	4	0	0.5	0.5	0.5	0.30	0.30	0.30	0.25	0.00	0.50	0.132	-0.072	0.180
1000	4	0	0.5	0.5	0.5	0.30	0.30	0.30	0.25	0.25	0.00	0.148	-0.082	0.149
1000	4	0	0.5	0.5	0.5	0.30	0.30	0.30	0.25	0.25	0.25	0.148	-0.082	0.149
1000	4	0	0.5	0.5	0.5	0.30	0.30	0.30	0.25	0.25	0.50	0.148	-0.081	0.149
1000	4	0	0.5	0.5	0.5	0.30	0.30	0.30	0.25	0.50	0.00	0.162	-0.090	0.113
1000	4	0	0.5	0.5	0.5	0.30	0.30	0.30	0.25	0.50	0.25	0.162	-0.090	0.112
1000	4	0	0.5	0.5	0.5	0.30	0.30	0.30	0.25	0.50	0.50	0.163	-0.091	0.113
1000	4	0	0.5	0.5	0.5	0.30	0.30	0.30	0.50	0.00	0.00	0.132	-0.035	0.180
1000	4	0	0.5	0.5	0.5	0.30	0.30	0.30	0.50	0.00	0.25	0.133	-0.036	0.180
1000	4	0	0.5	0.5	0.5	0.30	0.30	0.30	0.50	0.00	0.50	0.132	-0.036	0.180
1000	4	0	0.5	0.5	0.5	0.30	0.30	0.30	0.50	0.25	0.00	0.149	-0.048	0.150
1000	4	0	0.5	0.5	0.5	0.30	0.30	0.30	0.50	0.25	0.25	0.148	-0.047	0.150
1000	4	0	0.5	0.5	0.5	0.30	0.30	0.30	0.50	0.25	0.50	0.148	-0.048	0.150
1000	4	0	0.5	0.5	0.5	0.30	0.30	0.30	0.50	0.50	0.00	0.162	-0.057	0.112
1000	4	0	0.5	0.5	0.5	0.30	0.30	0.30	0.50	0.50	0.25	0.163	-0.058	0.113
1000	4	0	0.5	0.5	0.5	0.30	0.30	0.30	0.50	0.50	0.50	0.163	-0.057	0.113
1000	4	0	0.5	0.8	0.5	0.00	0.00	0.00	0.00	0.00	0.00	-0.001	0.001	0.000
1000	4	0	0.5	0.8	0.5	0.15	0.15	0.15	0.00	0.00	0.00	0.041	-0.061	0.045
1000	4	0	0.5	0.8	0.5	0.15	0.15	0.15	0.00	0.00	0.25	0.041	-0.061	0.045
1000	4	0	0.5	0.8	0.5	0.15	0.15	0.15	0.00	0.00	0.50	0.041	-0.060	0.044
1000	4	0	0.5	0.8	0.5	0.15	0.15	0.15	0.00	0.25	0.00	0.042	-0.062	0.034
1000	4	0	0.5	0.8	0.5	0.15	0.15	0.15	0.00	0.25	0.25	0.042	-0.062	0.034
1000	4	0	0.5	0.8	0.5	0.15	0.15	0.15	0.00	0.25	0.50	0.043	-0.063	0.035
1000	4	0	0.5	0.8	0.5	0.15	0.15	0.15	0.00	0.50	0.00	0.043	-0.064	0.023
1000	4	0	0.5	0.8	0.5	0.15	0.15	0.15	0.00	0.50	0.25	0.043	-0.064	0.023
1000	4	0	0.5	0.8	0.5	0.15	0.15	0.15	0.00	0.50	0.50	0.044	-0.065	0.024
1000	4	0	0.5	0.8	0.5	0.15	0.15	0.15	0.25	0.00	0.00	0.041	-0.050	0.045

(continued)

N	m_1	$\frac{m_2}{m_1}$	$E(C)$	$E(R)$	$E(U)$	σ_C	σ_R	σ_U	ρ_{CR}	ρ_{CU}	ρ_{RU}	Mean Bias c	r	u
1000	4	0	0.5	0.8	0.5	0.15	0.15	0.15	0.25	0.00	0.25	0.041	-0.050	0.044
1000	4	0	0.5	0.8	0.5	0.15	0.15	0.15	0.25	0.00	0.50	0.041	-0.051	0.045
1000	4	0	0.5	0.8	0.5	0.15	0.15	0.15	0.25	0.25	0.00	0.042	-0.052	0.034
1000	4	0	0.5	0.8	0.5	0.15	0.15	0.15	0.25	0.25	0.25	0.043	-0.053	0.035
1000	4	0	0.5	0.8	0.5	0.15	0.15	0.15	0.25	0.25	0.50	0.041	-0.052	0.034
1000	4	0	0.5	0.8	0.5	0.15	0.15	0.15	0.25	0.50	0.00	0.044	-0.054	0.023
1000	4	0	0.5	0.8	0.5	0.15	0.15	0.15	0.25	0.50	0.25	0.044	-0.055	0.024
1000	4	0	0.5	0.8	0.5	0.15	0.15	0.15	0.25	0.50	0.50	0.044	-0.055	0.023
1000	4	0	0.5	0.8	0.5	0.15	0.15	0.15	0.50	0.00	0.00	0.041	-0.040	0.044
1000	4	0	0.5	0.8	0.5	0.15	0.15	0.15	0.50	0.00	0.25	0.041	-0.040	0.045
1000	4	0	0.5	0.8	0.5	0.15	0.15	0.15	0.50	0.00	0.50	0.041	-0.040	0.045
1000	4	0	0.5	0.8	0.5	0.15	0.15	0.15	0.50	0.25	0.00	0.042	-0.042	0.033
1000	4	0	0.5	0.8	0.5	0.15	0.15	0.15	0.50	0.25	0.25	0.043	-0.043	0.035
1000	4	0	0.5	0.8	0.5	0.15	0.15	0.15	0.50	0.25	0.50	0.043	-0.042	0.034
1000	4	0	0.5	0.8	0.5	0.15	0.15	0.15	0.50	0.50	0.00	0.044	-0.044	0.023
1000	4	0	0.5	0.8	0.5	0.15	0.15	0.15	0.50	0.50	0.25	0.044	-0.045	0.023
1000	4	0	0.5	0.8	0.5	0.15	0.15	0.15	0.50	0.50	0.50	0.044	-0.045	0.024
1000	4	0	0.5	0.8	0.5	0.30	0.30	0.30	0.00	0.00	0.00	0.132	-0.167	0.180
1000	4	0	0.5	0.8	0.5	0.30	0.30	0.30	0.00	0.00	0.25	0.133	-0.166	0.179
1000	4	0	0.5	0.8	0.5	0.30	0.30	0.30	0.00	0.00	0.50	0.132	-0.167	0.179
1000	4	0	0.5	0.8	0.5	0.30	0.30	0.30	0.00	0.25	0.00	0.148	-0.182	0.150
1000	4	0	0.5	0.8	0.5	0.30	0.30	0.30	0.00	0.25	0.25	0.147	-0.183	0.149
1000	4	0	0.5	0.8	0.5	0.30	0.30	0.30	0.00	0.25	0.50	0.148	-0.183	0.150
1000	4	0	0.5	0.8	0.5	0.30	0.30	0.30	0.00	0.50	0.00	0.163	-0.197	0.112
1000	4	0	0.5	0.8	0.5	0.30	0.30	0.30	0.00	0.50	0.25	0.163	-0.197	0.114
1000	4	0	0.5	0.8	0.5	0.30	0.30	0.30	0.00	0.50	0.50	0.162	-0.195	0.113
1000	4	0	0.5	0.8	0.5	0.30	0.30	0.30	0.25	0.00	0.00	0.131	-0.139	0.179
1000	4	0	0.5	0.8	0.5	0.30	0.30	0.30	0.25	0.00	0.25	0.132	-0.138	0.180
1000	4	0	0.5	0.8	0.5	0.30	0.30	0.30	0.25	0.00	0.50	0.133	-0.138	0.180
1000	4	0	0.5	0.8	0.5	0.30	0.30	0.30	0.25	0.25	0.00	0.148	-0.154	0.149
1000	4	0	0.5	0.8	0.5	0.30	0.30	0.30	0.25	0.25	0.25	0.148	-0.154	0.150
1000	4	0	0.5	0.8	0.5	0.30	0.30	0.30	0.25	0.25	0.50	0.148	-0.155	0.150
1000	4	0	0.5	0.8	0.5	0.30	0.30	0.30	0.25	0.50	0.00	0.162	-0.168	0.113
1000	4	0	0.5	0.8	0.5	0.30	0.30	0.30	0.25	0.50	0.25	0.162	-0.168	0.113
1000	4	0	0.5	0.8	0.5	0.30	0.30	0.30	0.25	0.50	0.50	0.163	-0.167	0.113
1000	4	0	0.5	0.8	0.5	0.30	0.30	0.30	0.50	0.00	0.00	0.132	-0.109	0.179
1000	4	0	0.5	0.8	0.5	0.30	0.30	0.30	0.50	0.00	0.25	0.132	-0.109	0.179
1000	4	0	0.5	0.8	0.5	0.30	0.30	0.30	0.50	0.00	0.50	0.133	-0.110	0.180
1000	4	0	0.5	0.8	0.5	0.30	0.30	0.30	0.50	0.25	0.00	0.148	-0.125	0.149

(continued)

												Mean Bias		
N	m_1	$\frac{m_2}{m_1}$	$E(C)$	$E(R)$	$E(U)$	σ_C	σ_R	σ_U	ρ_{CR}	ρ_{CU}	ρ_{RU}	c	r	u
1000	4	0	0.5	0.8	0.5	0.30	0.30	0.30	0.50	0.25	0.25	0.148	-0.126	0.150
1000	4	0	0.5	0.8	0.5	0.30	0.30	0.30	0.50	0.25	0.50	0.148	-0.126	0.151
1000	4	0	0.5	0.8	0.5	0.30	0.30	0.30	0.50	0.50	0.00	0.163	-0.141	0.114
1000	4	0	0.5	0.8	0.5	0.30	0.30	0.30	0.50	0.50	0.25	0.162	-0.141	0.113
1000	4	0	0.5	0.8	0.5	0.30	0.30	0.30	0.50	0.50	0.50	0.163	-0.141	0.113
1000	4	0	0.8	0.2	0.5	0.00	0.00	0.00	0.00	0.00	0.00	0.000	0.000	0.001
1000	4	0	0.8	0.2	0.5	0.15	0.15	0.15	0.00	0.00	0.00	0.016	-0.004	0.045
1000	4	0	0.8	0.2	0.5	0.15	0.15	0.15	0.00	0.00	0.25	0.016	-0.004	0.044
1000	4	0	0.8	0.2	0.5	0.15	0.15	0.15	0.00	0.00	0.50	0.016	-0.004	0.045
1000	4	0	0.8	0.2	0.5	0.15	0.15	0.15	0.00	0.25	0.00	0.018	-0.004	0.020
1000	4	0	0.8	0.2	0.5	0.15	0.15	0.15	0.00	0.25	0.25	0.018	-0.004	0.019
1000	4	0	0.8	0.2	0.5	0.15	0.15	0.15	0.00	0.25	0.50	0.018	-0.005	0.019
1000	4	0	0.8	0.2	0.5	0.15	0.15	0.15	0.00	0.50	0.00	0.019	-0.005	-0.008
1000	4	0	0.8	0.2	0.5	0.15	0.15	0.15	0.00	0.50	0.25	0.019	-0.005	-0.006
1000	4	0	0.8	0.2	0.5	0.15	0.15	0.15	0.00	0.50	0.50	0.019	-0.004	-0.008
1000	4	0	0.8	0.2	0.5	0.15	0.15	0.15	0.25	0.00	0.00	0.016	0.002	0.046
1000	4	0	0.8	0.2	0.5	0.15	0.15	0.15	0.25	0.00	0.25	0.017	0.002	0.046
1000	4	0	0.8	0.2	0.5	0.15	0.15	0.15	0.25	0.00	0.50	0.016	0.002	0.045
1000	4	0	0.8	0.2	0.5	0.15	0.15	0.15	0.25	0.25	0.00	0.018	0.002	0.020
1000	4	0	0.8	0.2	0.5	0.15	0.15	0.15	0.25	0.25	0.25	0.018	0.002	0.021
1000	4	0	0.8	0.2	0.5	0.15	0.15	0.15	0.25	0.25	0.50	0.018	0.002	0.019
1000	4	0	0.8	0.2	0.5	0.15	0.15	0.15	0.25	0.50	0.00	0.018	0.001	-0.007
1000	4	0	0.8	0.2	0.5	0.15	0.15	0.15	0.25	0.50	0.25	0.019	0.002	-0.007
1000	4	0	0.8	0.2	0.5	0.15	0.15	0.15	0.25	0.50	0.50	0.018	0.002	-0.007
1000	4	0	0.8	0.2	0.5	0.15	0.15	0.15	0.50	0.00	0.00	0.017	0.008	0.045
1000	4	0	0.8	0.2	0.5	0.15	0.15	0.15	0.50	0.00	0.25	0.016	0.008	0.045
1000	4	0	0.8	0.2	0.5	0.15	0.15	0.15	0.50	0.00	0.50	0.016	0.008	0.045
1000	4	0	0.8	0.2	0.5	0.15	0.15	0.15	0.50	0.25	0.00	0.018	0.008	0.019
1000	4	0	0.8	0.2	0.5	0.15	0.15	0.15	0.50	0.25	0.25	0.018	0.008	0.019
1000	4	0	0.8	0.2	0.5	0.15	0.15	0.15	0.50	0.25	0.50	0.017	0.007	0.019
1000	4	0	0.8	0.2	0.5	0.15	0.15	0.15	0.50	0.50	0.00	0.019	0.007	-0.007
1000	4	0	0.8	0.2	0.5	0.15	0.15	0.15	0.50	0.50	0.25	0.019	0.007	-0.006
1000	4	0	0.8	0.2	0.5	0.15	0.15	0.15	0.50	0.50	0.50	0.019	0.007	-0.006
1000	4	0	0.8	0.2	0.5	0.30	0.30	0.30	0.00	0.00	0.00	0.053	-0.012	0.179
1000	4	0	0.8	0.2	0.5	0.30	0.30	0.30	0.00	0.00	0.25	0.053	-0.012	0.180
1000	4	0	0.8	0.2	0.5	0.30	0.30	0.30	0.00	0.00	0.50	0.053	-0.012	0.179
1000	4	0	0.8	0.2	0.5	0.30	0.30	0.30	0.00	0.25	0.00	0.067	-0.015	0.114
1000	4	0	0.8	0.2	0.5	0.30	0.30	0.30	0.00	0.25	0.25	0.067	-0.015	0.114
1000	4	0	0.8	0.2	0.5	0.30	0.30	0.30	0.00	0.25	0.50	0.067	-0.016	0.114

(continued)

												Mean Bias		
N	m_1	$\frac{m_2}{m_1}$	$E(C)$	$E(R)$	$E(U)$	σ_C	σ_R	σ_U	ρ_{CR}	ρ_{CU}	ρ_{RU}	c	r	u
1000	4	0	0.8	0.2	0.5	0.30	0.30	0.30	0.00	0.50	0.00	0.081	-0.018	0.030
1000	4	0	0.8	0.2	0.5	0.30	0.30	0.30	0.00	0.50	0.25	0.080	-0.018	0.031
1000	4	0	0.8	0.2	0.5	0.30	0.30	0.30	0.00	0.50	0.50	0.081	-0.018	0.029
1000	4	0	0.8	0.2	0.5	0.30	0.30	0.30	0.25	0.00	0.00	0.053	0.005	0.179
1000	4	0	0.8	0.2	0.5	0.30	0.30	0.30	0.25	0.00	0.25	0.053	0.005	0.180
1000	4	0	0.8	0.2	0.5	0.30	0.30	0.30	0.25	0.00	0.50	0.053	0.004	0.180
1000	4	0	0.8	0.2	0.5	0.30	0.30	0.30	0.25	0.25	0.00	0.067	0.001	0.115
1000	4	0	0.8	0.2	0.5	0.30	0.30	0.30	0.25	0.25	0.25	0.067	0.001	0.115
1000	4	0	0.8	0.2	0.5	0.30	0.30	0.30	0.25	0.25	0.50	0.067	0.002	0.114
1000	4	0	0.8	0.2	0.5	0.30	0.30	0.30	0.25	0.50	0.00	0.081	-0.002	0.031
1000	4	0	0.8	0.2	0.5	0.30	0.30	0.30	0.25	0.50	0.25	0.081	-0.001	0.030
1000	4	0	0.8	0.2	0.5	0.30	0.30	0.30	0.25	0.50	0.50	0.080	-0.002	0.030
1000	4	0	0.8	0.2	0.5	0.30	0.30	0.30	0.50	0.00	0.00	0.053	0.019	0.179
1000	4	0	0.8	0.2	0.5	0.30	0.30	0.30	0.50	0.00	0.25	0.053	0.019	0.180
1000	4	0	0.8	0.2	0.5	0.30	0.30	0.30	0.50	0.00	0.50	0.053	0.019	0.179
1000	4	0	0.8	0.2	0.5	0.30	0.30	0.30	0.50	0.25	0.00	0.067	0.016	0.113
1000	4	0	0.8	0.2	0.5	0.30	0.30	0.30	0.50	0.25	0.25	0.067	0.015	0.114
1000	4	0	0.8	0.2	0.5	0.30	0.30	0.30	0.50	0.25	0.50	0.067	0.015	0.114
1000	4	0	0.8	0.2	0.5	0.30	0.30	0.30	0.50	0.50	0.00	0.081	0.012	0.030
1000	4	0	0.8	0.2	0.5	0.30	0.30	0.30	0.50	0.50	0.25	0.081	0.012	0.029
1000	4	0	0.8	0.2	0.5	0.30	0.30	0.30	0.50	0.50	0.50	0.080	0.012	0.029
1000	4	0	0.8	0.5	0.5	0.00	0.00	0.00	0.00	0.00	0.00	0.000	0.001	-0.001
1000	4	0	0.8	0.5	0.5	0.15	0.15	0.15	0.00	0.00	0.00	0.016	-0.011	0.046
1000	4	0	0.8	0.5	0.5	0.15	0.15	0.15	0.00	0.00	0.25	0.016	-0.010	0.045
1000	4	0	0.8	0.5	0.5	0.15	0.15	0.15	0.00	0.00	0.50	0.017	-0.010	0.045
1000	4	0	0.8	0.5	0.5	0.15	0.15	0.15	0.00	0.25	0.00	0.018	-0.011	0.020
1000	4	0	0.8	0.5	0.5	0.15	0.15	0.15	0.00	0.25	0.25	0.018	-0.010	0.019
1000	4	0	0.8	0.5	0.5	0.15	0.15	0.15	0.00	0.25	0.50	0.018	-0.011	0.020
1000	4	0	0.8	0.5	0.5	0.15	0.15	0.15	0.00	0.50	0.00	0.019	-0.011	-0.006
1000	4	0	0.8	0.5	0.5	0.15	0.15	0.15	0.00	0.50	0.25	0.019	-0.011	-0.007
1000	4	0	0.8	0.5	0.5	0.15	0.15	0.15	0.00	0.50	0.50	0.018	-0.011	-0.007
1000	4	0	0.8	0.5	0.5	0.15	0.15	0.15	0.25	0.00	0.00	0.016	-0.004	0.045
1000	4	0	0.8	0.5	0.5	0.15	0.15	0.15	0.25	0.00	0.25	0.017	-0.003	0.045
1000	4	0	0.8	0.5	0.5	0.15	0.15	0.15	0.25	0.00	0.50	0.017	-0.004	0.045
1000	4	0	0.8	0.5	0.5	0.15	0.15	0.15	0.25	0.25	0.00	0.018	-0.004	0.022
1000	4	0	0.8	0.5	0.5	0.15	0.15	0.15	0.25	0.25	0.25	0.018	-0.004	0.019
1000	4	0	0.8	0.5	0.5	0.15	0.15	0.15	0.25	0.25	0.50	0.018	-0.004	0.020
1000	4	0	0.8	0.5	0.5	0.15	0.15	0.15	0.25	0.50	0.00	0.019	-0.005	-0.007
1000	4	0	0.8	0.5	0.5	0.15	0.15	0.15	0.25	0.50	0.25	0.019	-0.005	-0.007

(continued)

N	m_1	$\frac{m_2}{m_1}$	$E(C)$	$E(R)$	$E(U)$	σ_C	σ_R	σ_U	ρ_{CR}	ρ_{CU}	ρ_{RU}	Mean Bias c	Mean Bias r	Mean Bias u
1000	4	0	0.8	0.5	0.5	0.15	0.15	0.15	0.25	0.50	0.50	0.020	-0.005	-0.005
1000	4	0	0.8	0.5	0.5	0.15	0.15	0.15	0.50	0.00	0.00	0.017	0.004	0.045
1000	4	0	0.8	0.5	0.5	0.15	0.15	0.15	0.50	0.00	0.25	0.016	0.004	0.044
1000	4	0	0.8	0.5	0.5	0.15	0.15	0.15	0.50	0.00	0.50	0.017	0.003	0.045
1000	4	0	0.8	0.5	0.5	0.15	0.15	0.15	0.50	0.25	0.00	0.018	0.003	0.019
1000	4	0	0.8	0.5	0.5	0.15	0.15	0.15	0.50	0.25	0.25	0.018	0.003	0.020
1000	4	0	0.8	0.5	0.5	0.15	0.15	0.15	0.50	0.25	0.50	0.018	0.002	0.019
1000	4	0	0.8	0.5	0.5	0.15	0.15	0.15	0.50	0.50	0.00	0.019	0.002	-0.006
1000	4	0	0.8	0.5	0.5	0.15	0.15	0.15	0.50	0.50	0.25	0.019	0.002	-0.007
1000	4	0	0.8	0.5	0.5	0.15	0.15	0.15	0.50	0.50	0.50	0.019	0.001	-0.008
1000	4	0	0.8	0.5	0.5	0.30	0.30	0.30	0.00	0.00	0.00	0.053	-0.030	0.181
1000	4	0	0.8	0.5	0.5	0.30	0.30	0.30	0.00	0.00	0.25	0.053	-0.031	0.179
1000	4	0	0.8	0.5	0.5	0.30	0.30	0.30	0.00	0.00	0.50	0.052	-0.031	0.178
1000	4	0	0.8	0.5	0.5	0.30	0.30	0.30	0.00	0.25	0.00	0.068	-0.038	0.113
1000	4	0	0.8	0.5	0.5	0.30	0.30	0.30	0.00	0.25	0.25	0.067	-0.039	0.114
1000	4	0	0.8	0.5	0.5	0.30	0.30	0.30	0.00	0.25	0.50	0.067	-0.039	0.115
1000	4	0	0.8	0.5	0.5	0.30	0.30	0.30	0.00	0.50	0.00	0.081	-0.046	0.029
1000	4	0	0.8	0.5	0.5	0.30	0.30	0.30	0.00	0.50	0.25	0.081	-0.045	0.031
1000	4	0	0.8	0.5	0.5	0.30	0.30	0.30	0.00	0.50	0.50	0.080	-0.046	0.030
1000	4	0	0.8	0.5	0.5	0.30	0.30	0.30	0.25	0.00	0.00	0.053	-0.009	0.180
1000	4	0	0.8	0.5	0.5	0.30	0.30	0.30	0.25	0.00	0.25	0.053	-0.009	0.180
1000	4	0	0.8	0.5	0.5	0.30	0.30	0.30	0.25	0.00	0.50	0.053	-0.010	0.179
1000	4	0	0.8	0.5	0.5	0.30	0.30	0.30	0.25	0.25	0.00	0.067	-0.018	0.114
1000	4	0	0.8	0.5	0.5	0.30	0.30	0.30	0.25	0.25	0.25	0.067	-0.017	0.115
1000	4	0	0.8	0.5	0.5	0.30	0.30	0.30	0.25	0.25	0.50	0.067	-0.017	0.113
1000	4	0	0.8	0.5	0.5	0.30	0.30	0.30	0.25	0.50	0.00	0.081	-0.025	0.031
1000	4	0	0.8	0.5	0.5	0.30	0.30	0.30	0.25	0.50	0.25	0.081	-0.025	0.031
1000	4	0	0.8	0.5	0.5	0.30	0.30	0.30	0.25	0.50	0.50	0.080	-0.025	0.030
1000	4	0	0.8	0.5	0.5	0.30	0.30	0.30	0.50	0.00	0.00	0.053	0.012	0.181
1000	4	0	0.8	0.5	0.5	0.30	0.30	0.30	0.50	0.00	0.25	0.053	0.013	0.179
1000	4	0	0.8	0.5	0.5	0.30	0.30	0.30	0.50	0.00	0.50	0.053	0.013	0.180
1000	4	0	0.8	0.5	0.5	0.30	0.30	0.30	0.50	0.25	0.00	0.067	0.004	0.113
1000	4	0	0.8	0.5	0.5	0.30	0.30	0.30	0.50	0.25	0.25	0.067	0.004	0.115
1000	4	0	0.8	0.5	0.5	0.30	0.30	0.30	0.50	0.25	0.50	0.067	0.005	0.113
1000	4	0	0.8	0.5	0.5	0.30	0.30	0.30	0.50	0.50	0.00	0.081	-0.004	0.031
1000	4	0	0.8	0.5	0.5	0.30	0.30	0.30	0.50	0.50	0.25	0.081	-0.005	0.030
1000	4	0	0.8	0.5	0.5	0.30	0.30	0.30	0.50	0.50	0.50	0.081	-0.004	0.030
1000	4	0	0.8	0.8	0.5	0.00	0.00	0.00	0.00	0.00	0.00	0.000	0.000	0.001
1000	4	0	0.8	0.8	0.5	0.15	0.15	0.15	0.00	0.00	0.00	0.016	-0.016	0.044

(continued)

N	m_1	$\frac{m_2}{m_1}$	$E(C)$	$E(R)$	$E(U)$	σ_C	σ_R	σ_U	ρ_{CR}	ρ_{CU}	ρ_{RU}	Mean Bias c	Mean Bias r	Mean Bias u
1000	4	0	0.8	0.8	0.5	0.15	0.15	0.15	0.00	0.00	0.25	0.016	-0.016	0.044
1000	4	0	0.8	0.8	0.5	0.15	0.15	0.15	0.00	0.00	0.50	0.016	-0.015	0.044
1000	4	0	0.8	0.8	0.5	0.15	0.15	0.15	0.00	0.25	0.00	0.019	-0.017	0.020
1000	4	0	0.8	0.8	0.5	0.15	0.15	0.15	0.00	0.25	0.25	0.018	-0.017	0.020
1000	4	0	0.8	0.8	0.5	0.15	0.15	0.15	0.00	0.25	0.50	0.018	-0.017	0.019
1000	4	0	0.8	0.8	0.5	0.15	0.15	0.15	0.00	0.50	0.00	0.019	-0.018	-0.008
1000	4	0	0.8	0.8	0.5	0.15	0.15	0.15	0.00	0.50	0.25	0.019	-0.018	-0.007
1000	4	0	0.8	0.8	0.5	0.15	0.15	0.15	0.00	0.50	0.50	0.019	-0.019	-0.006
1000	4	0	0.8	0.8	0.5	0.15	0.15	0.15	0.25	0.00	0.00	0.016	-0.010	0.045
1000	4	0	0.8	0.8	0.5	0.15	0.15	0.15	0.25	0.00	0.25	0.016	-0.009	0.044
1000	4	0	0.8	0.8	0.5	0.15	0.15	0.15	0.25	0.00	0.50	0.016	-0.009	0.044
1000	4	0	0.8	0.8	0.5	0.15	0.15	0.15	0.25	0.25	0.00	0.018	-0.010	0.019
1000	4	0	0.8	0.8	0.5	0.15	0.15	0.15	0.25	0.25	0.25	0.018	-0.011	0.020
1000	4	0	0.8	0.8	0.5	0.15	0.15	0.15	0.25	0.25	0.50	0.018	-0.010	0.021
1000	4	0	0.8	0.8	0.5	0.15	0.15	0.15	0.25	0.50	0.00	0.019	-0.012	-0.006
1000	4	0	0.8	0.8	0.5	0.15	0.15	0.15	0.25	0.50	0.25	0.019	-0.012	-0.007
1000	4	0	0.8	0.8	0.5	0.15	0.15	0.15	0.25	0.50	0.50	0.019	-0.012	-0.007
1000	4	0	0.8	0.8	0.5	0.15	0.15	0.15	0.50	0.00	0.00	0.016	-0.002	0.046
1000	4	0	0.8	0.8	0.5	0.15	0.15	0.15	0.50	0.00	0.25	0.016	-0.003	0.045
1000	4	0	0.8	0.8	0.5	0.15	0.15	0.15	0.50	0.00	0.50	0.017	-0.003	0.045
1000	4	0	0.8	0.8	0.5	0.15	0.15	0.15	0.50	0.25	0.00	0.018	-0.004	0.020
1000	4	0	0.8	0.8	0.5	0.15	0.15	0.15	0.50	0.25	0.25	0.018	-0.004	0.021
1000	4	0	0.8	0.8	0.5	0.15	0.15	0.15	0.50	0.25	0.50	0.018	-0.004	0.019
1000	4	0	0.8	0.8	0.5	0.15	0.15	0.15	0.50	0.50	0.00	0.019	-0.005	-0.006
1000	4	0	0.8	0.8	0.5	0.15	0.15	0.15	0.50	0.50	0.25	0.019	-0.005	-0.007
1000	4	0	0.8	0.8	0.5	0.15	0.15	0.15	0.50	0.50	0.50	0.019	-0.005	-0.006
1000	4	0	0.8	0.8	0.5	0.30	0.30	0.30	0.00	0.00	0.00	0.053	-0.049	0.179
1000	4	0	0.8	0.8	0.5	0.30	0.30	0.30	0.00	0.00	0.25	0.053	-0.049	0.180
1000	4	0	0.8	0.8	0.5	0.30	0.30	0.30	0.00	0.00	0.50	0.053	-0.050	0.181
1000	4	0	0.8	0.8	0.5	0.30	0.30	0.30	0.00	0.25	0.00	0.067	-0.062	0.114
1000	4	0	0.8	0.8	0.5	0.30	0.30	0.30	0.00	0.25	0.25	0.067	-0.062	0.113
1000	4	0	0.8	0.8	0.5	0.30	0.30	0.30	0.00	0.25	0.50	0.067	-0.062	0.114
1000	4	0	0.8	0.8	0.5	0.30	0.30	0.30	0.00	0.50	0.00	0.081	-0.073	0.030
1000	4	0	0.8	0.8	0.5	0.30	0.30	0.30	0.00	0.50	0.25	0.081	-0.073	0.029
1000	4	0	0.8	0.8	0.5	0.30	0.30	0.30	0.00	0.50	0.50	0.081	-0.073	0.030
1000	4	0	0.8	0.8	0.5	0.30	0.30	0.30	0.25	0.00	0.00	0.053	-0.029	0.179
1000	4	0	0.8	0.8	0.5	0.30	0.30	0.30	0.25	0.00	0.25	0.053	-0.029	0.180
1000	4	0	0.8	0.8	0.5	0.30	0.30	0.30	0.25	0.00	0.50	0.052	-0.030	0.181
1000	4	0	0.8	0.8	0.5	0.30	0.30	0.30	0.25	0.25	0.00	0.067	-0.043	0.115

(continued)

N	m_1	$\frac{m_2}{m_1}$	$E(C)$	$E(R)$	$E(U)$	σ_C	σ_R	σ_U	ρ_{CR}	ρ_{CU}	ρ_{RU}	Mean Bias c	r	u
1000	4	0	0.8	0.8	0.5	0.30	0.30	0.30	0.25	0.25	0.25	0.067	-0.041	0.113
1000	4	0	0.8	0.8	0.5	0.30	0.30	0.30	0.25	0.25	0.50	0.067	-0.042	0.115
1000	4	0	0.8	0.8	0.5	0.30	0.30	0.30	0.25	0.50	0.00	0.080	-0.053	0.029
1000	4	0	0.8	0.8	0.5	0.30	0.30	0.30	0.25	0.50	0.25	0.081	-0.055	0.030
1000	4	0	0.8	0.8	0.5	0.30	0.30	0.30	0.25	0.50	0.50	0.081	-0.055	0.030
1000	4	0	0.8	0.8	0.5	0.30	0.30	0.30	0.50	0.00	0.00	0.053	-0.007	0.180
1000	4	0	0.8	0.8	0.5	0.30	0.30	0.30	0.50	0.00	0.25	0.052	-0.007	0.179
1000	4	0	0.8	0.8	0.5	0.30	0.30	0.30	0.50	0.00	0.50	0.053	-0.006	0.179
1000	4	0	0.8	0.8	0.5	0.30	0.30	0.30	0.50	0.25	0.00	0.067	-0.019	0.114
1000	4	0	0.8	0.8	0.5	0.30	0.30	0.30	0.50	0.25	0.25	0.067	-0.020	0.113
1000	4	0	0.8	0.8	0.5	0.30	0.30	0.30	0.50	0.25	0.50	0.067	-0.020	0.113
1000	4	0	0.8	0.8	0.5	0.30	0.30	0.30	0.50	0.50	0.00	0.081	-0.031	0.030
1000	4	0	0.8	0.8	0.5	0.30	0.30	0.30	0.50	0.50	0.25	0.080	-0.032	0.029
1000	4	0	0.8	0.8	0.5	0.30	0.30	0.30	0.50	0.50	0.50	0.081	-0.032	0.031
1000	4	1	0.2	0.2	0.5	0.00	0.00	0.00	0.00	0.00	0.00	0.000	0.000	0.000
1000	4	1	0.2	0.2	0.5	0.15	0.15	0.15	0.00	0.00	0.00	0.039	-0.033	0.016
1000	4	1	0.2	0.2	0.5	0.15	0.15	0.15	0.00	0.00	0.25	0.039	-0.033	0.016
1000	4	1	0.2	0.2	0.5	0.15	0.15	0.15	0.00	0.00	0.50	0.039	-0.033	0.016
1000	4	1	0.2	0.2	0.5	0.15	0.15	0.15	0.00	0.25	0.00	0.044	-0.036	0.014
1000	4	1	0.2	0.2	0.5	0.15	0.15	0.15	0.00	0.25	0.25	0.044	-0.036	0.013
1000	4	1	0.2	0.2	0.5	0.15	0.15	0.15	0.00	0.25	0.50	0.043	-0.036	0.013
1000	4	1	0.2	0.2	0.5	0.15	0.15	0.15	0.00	0.50	0.00	0.048	-0.039	0.011
1000	4	1	0.2	0.2	0.5	0.15	0.15	0.15	0.00	0.50	0.25	0.049	-0.039	0.011
1000	4	1	0.2	0.2	0.5	0.15	0.15	0.15	0.00	0.50	0.50	0.049	-0.039	0.011
1000	4	1	0.2	0.2	0.5	0.15	0.15	0.15	0.25	0.00	0.00	0.039	-0.010	0.016
1000	4	1	0.2	0.2	0.5	0.15	0.15	0.15	0.25	0.00	0.25	0.040	-0.012	0.016
1000	4	1	0.2	0.2	0.5	0.15	0.15	0.15	0.25	0.00	0.50	0.040	-0.011	0.016
1000	4	1	0.2	0.2	0.5	0.15	0.15	0.15	0.25	0.25	0.00	0.044	-0.014	0.013
1000	4	1	0.2	0.2	0.5	0.15	0.15	0.15	0.25	0.25	0.25	0.044	-0.014	0.013
1000	4	1	0.2	0.2	0.5	0.15	0.15	0.15	0.25	0.25	0.50	0.045	-0.015	0.013
1000	4	1	0.2	0.2	0.5	0.15	0.15	0.15	0.25	0.50	0.00	0.048	-0.018	0.011
1000	4	1	0.2	0.2	0.5	0.15	0.15	0.15	0.25	0.50	0.25	0.048	-0.018	0.011
1000	4	1	0.2	0.2	0.5	0.15	0.15	0.15	0.25	0.50	0.50	0.048	-0.017	0.011
1000	4	1	0.2	0.2	0.5	0.15	0.15	0.15	0.50	0.00	0.00	0.040	0.011	0.016
1000	4	1	0.2	0.2	0.5	0.15	0.15	0.15	0.50	0.00	0.25	0.039	0.012	0.016
1000	4	1	0.2	0.2	0.5	0.15	0.15	0.15	0.50	0.00	0.50	0.039	0.012	0.016
1000	4	1	0.2	0.2	0.5	0.15	0.15	0.15	0.50	0.25	0.00	0.045	0.008	0.014
1000	4	1	0.2	0.2	0.5	0.15	0.15	0.15	0.50	0.25	0.25	0.044	0.007	0.013
1000	4	1	0.2	0.2	0.5	0.15	0.15	0.15	0.50	0.25	0.50	0.045	0.007	0.014

(continued)

												Mean Bias		
N	m_1	$\frac{m_2}{m_1}$	$E(C)$	$E(R)$	$E(U)$	σ_C	σ_R	σ_U	ρ_{CR}	ρ_{CU}	ρ_{RU}	c	r	u
1000	4	1	0.2	0.2	0.5	0.15	0.15	0.15	0.50	0.50	0.00	0.049	0.004	0.011
1000	4	1	0.2	0.2	0.5	0.15	0.15	0.15	0.50	0.50	0.25	0.048	0.005	0.011
1000	4	1	0.2	0.2	0.5	0.15	0.15	0.15	0.50	0.50	0.50	0.048	0.004	0.010
1000	4	1	0.2	0.2	0.5	0.30	0.30	0.30	0.00	0.00	0.00	0.148	-0.086	0.064
1000	4	1	0.2	0.2	0.5	0.30	0.30	0.30	0.00	0.00	0.25	0.148	-0.085	0.065
1000	4	1	0.2	0.2	0.5	0.30	0.30	0.30	0.00	0.00	0.50	0.149	-0.085	0.064
1000	4	1	0.2	0.2	0.5	0.30	0.30	0.30	0.00	0.25	0.00	0.165	-0.090	0.056
1000	4	1	0.2	0.2	0.5	0.30	0.30	0.30	0.00	0.25	0.25	0.165	-0.090	0.056
1000	4	1	0.2	0.2	0.5	0.30	0.30	0.30	0.00	0.25	0.50	0.165	-0.090	0.056
1000	4	1	0.2	0.2	0.5	0.30	0.30	0.30	0.00	0.50	0.00	0.179	-0.094	0.047
1000	4	1	0.2	0.2	0.5	0.30	0.30	0.30	0.00	0.50	0.25	0.179	-0.094	0.047
1000	4	1	0.2	0.2	0.5	0.30	0.30	0.30	0.00	0.50	0.50	0.179	-0.094	0.047
1000	4	1	0.2	0.2	0.5	0.30	0.30	0.30	0.25	0.00	0.00	0.148	-0.036	0.064
1000	4	1	0.2	0.2	0.5	0.30	0.30	0.30	0.25	0.00	0.25	0.148	-0.036	0.064
1000	4	1	0.2	0.2	0.5	0.30	0.30	0.30	0.25	0.00	0.50	0.148	-0.037	0.064
1000	4	1	0.2	0.2	0.5	0.30	0.30	0.30	0.25	0.25	0.00	0.165	-0.045	0.056
1000	4	1	0.2	0.2	0.5	0.30	0.30	0.30	0.25	0.25	0.25	0.164	-0.045	0.057
1000	4	1	0.2	0.2	0.5	0.30	0.30	0.30	0.25	0.25	0.50	0.164	-0.044	0.056
1000	4	1	0.2	0.2	0.5	0.30	0.30	0.30	0.25	0.50	0.00	0.179	-0.049	0.047
1000	4	1	0.2	0.2	0.5	0.30	0.30	0.30	0.25	0.50	0.25	0.179	-0.050	0.047
1000	4	1	0.2	0.2	0.5	0.30	0.30	0.30	0.25	0.50	0.50	0.179	-0.050	0.047
1000	4	1	0.2	0.2	0.5	0.30	0.30	0.30	0.50	0.00	0.00	0.148	0.020	0.064
1000	4	1	0.2	0.2	0.5	0.30	0.30	0.30	0.50	0.00	0.25	0.148	0.019	0.064
1000	4	1	0.2	0.2	0.5	0.30	0.30	0.30	0.50	0.00	0.50	0.149	0.019	0.064
1000	4	1	0.2	0.2	0.5	0.30	0.30	0.30	0.50	0.25	0.00	0.165	0.009	0.056
1000	4	1	0.2	0.2	0.5	0.30	0.30	0.30	0.50	0.25	0.25	0.164	0.010	0.056
1000	4	1	0.2	0.2	0.5	0.30	0.30	0.30	0.50	0.25	0.50	0.165	0.009	0.056
1000	4	1	0.2	0.2	0.5	0.30	0.30	0.30	0.50	0.50	0.00	0.179	0.002	0.047
1000	4	1	0.2	0.2	0.5	0.30	0.30	0.30	0.50	0.50	0.25	0.180	0.001	0.047
1000	4	1	0.2	0.2	0.5	0.30	0.30	0.30	0.50	0.50	0.50	0.178	0.001	0.047
1000	4	1	0.2	0.5	0.5	0.00	0.00	0.00	0.00	0.00	0.00	0.000	0.002	0.000
1000	4	1	0.2	0.5	0.5	0.15	0.15	0.15	0.00	0.00	0.00	0.039	-0.082	0.016
1000	4	1	0.2	0.5	0.5	0.15	0.15	0.15	0.00	0.00	0.25	0.039	-0.082	0.016
1000	4	1	0.2	0.5	0.5	0.15	0.15	0.15	0.00	0.00	0.50	0.040	-0.082	0.016
1000	4	1	0.2	0.5	0.5	0.15	0.15	0.15	0.00	0.25	0.00	0.044	-0.090	0.013
1000	4	1	0.2	0.5	0.5	0.15	0.15	0.15	0.00	0.25	0.25	0.044	-0.091	0.013
1000	4	1	0.2	0.5	0.5	0.15	0.15	0.15	0.00	0.25	0.50	0.045	-0.090	0.013
1000	4	1	0.2	0.5	0.5	0.15	0.15	0.15	0.00	0.50	0.00	0.049	-0.097	0.011
1000	4	1	0.2	0.5	0.5	0.15	0.15	0.15	0.00	0.50	0.25	0.049	-0.098	0.011

(continued)

												Mean Bias		
N	m_1	$\frac{m_2}{m_1}$	$E(C)$	$E(R)$	$E(U)$	σ_C	σ_R	σ_U	ρ_{CR}	ρ_{CU}	ρ_{RU}	c	r	u
1000	4	1	0.2	0.5	0.5	0.15	0.15	0.15	0.00	0.50	0.50	0.049	-0.099	0.011
1000	4	1	0.2	0.5	0.5	0.15	0.15	0.15	0.25	0.00	0.00	0.040	-0.060	0.016
1000	4	1	0.2	0.5	0.5	0.15	0.15	0.15	0.25	0.00	0.25	0.040	-0.060	0.016
1000	4	1	0.2	0.5	0.5	0.15	0.15	0.15	0.25	0.00	0.50	0.039	-0.059	0.015
1000	4	1	0.2	0.5	0.5	0.15	0.15	0.15	0.25	0.25	0.00	0.044	-0.068	0.014
1000	4	1	0.2	0.5	0.5	0.15	0.15	0.15	0.25	0.25	0.25	0.044	-0.068	0.013
1000	4	1	0.2	0.5	0.5	0.15	0.15	0.15	0.25	0.25	0.50	0.045	-0.068	0.013
1000	4	1	0.2	0.5	0.5	0.15	0.15	0.15	0.25	0.50	0.00	0.048	-0.074	0.010
1000	4	1	0.2	0.5	0.5	0.15	0.15	0.15	0.25	0.50	0.25	0.049	-0.077	0.011
1000	4	1	0.2	0.5	0.5	0.15	0.15	0.15	0.25	0.50	0.50	0.049	-0.077	0.011
1000	4	1	0.2	0.5	0.5	0.15	0.15	0.15	0.50	0.00	0.00	0.039	-0.037	0.015
1000	4	1	0.2	0.5	0.5	0.15	0.15	0.15	0.50	0.00	0.25	0.040	-0.038	0.016
1000	4	1	0.2	0.5	0.5	0.15	0.15	0.15	0.50	0.00	0.50	0.039	-0.037	0.016
1000	4	1	0.2	0.5	0.5	0.15	0.15	0.15	0.50	0.25	0.00	0.044	-0.046	0.014
1000	4	1	0.2	0.5	0.5	0.15	0.15	0.15	0.50	0.25	0.25	0.044	-0.046	0.013
1000	4	1	0.2	0.5	0.5	0.15	0.15	0.15	0.50	0.25	0.50	0.044	-0.047	0.013
1000	4	1	0.2	0.5	0.5	0.15	0.15	0.15	0.50	0.50	0.00	0.049	-0.055	0.011
1000	4	1	0.2	0.5	0.5	0.15	0.15	0.15	0.50	0.50	0.25	0.048	-0.054	0.011
1000	4	1	0.2	0.5	0.5	0.15	0.15	0.15	0.50	0.50	0.50	0.048	-0.053	0.011
1000	4	1	0.2	0.5	0.5	0.30	0.30	0.30	0.00	0.00	0.00	0.148	-0.212	0.064
1000	4	1	0.2	0.5	0.5	0.30	0.30	0.30	0.00	0.00	0.25	0.148	-0.212	0.065
1000	4	1	0.2	0.5	0.5	0.30	0.30	0.30	0.00	0.00	0.50	0.149	-0.213	0.063
1000	4	1	0.2	0.5	0.5	0.30	0.30	0.30	0.00	0.25	0.00	0.165	-0.226	0.056
1000	4	1	0.2	0.5	0.5	0.30	0.30	0.30	0.00	0.25	0.25	0.165	-0.226	0.056
1000	4	1	0.2	0.5	0.5	0.30	0.30	0.30	0.00	0.25	0.50	0.164	-0.226	0.056
1000	4	1	0.2	0.5	0.5	0.30	0.30	0.30	0.00	0.50	0.00	0.179	-0.236	0.047
1000	4	1	0.2	0.5	0.5	0.30	0.30	0.30	0.00	0.50	0.25	0.179	-0.236	0.047
1000	4	1	0.2	0.5	0.5	0.30	0.30	0.30	0.00	0.50	0.50	0.179	-0.235	0.047
1000	4	1	0.2	0.5	0.5	0.30	0.30	0.30	0.25	0.00	0.00	0.149	-0.160	0.065
1000	4	1	0.2	0.5	0.5	0.30	0.30	0.30	0.25	0.00	0.25	0.148	-0.160	0.064
1000	4	1	0.2	0.5	0.5	0.30	0.30	0.30	0.25	0.00	0.50	0.148	-0.160	0.065
1000	4	1	0.2	0.5	0.5	0.30	0.30	0.30	0.25	0.25	0.00	0.164	-0.175	0.056
1000	4	1	0.2	0.5	0.5	0.30	0.30	0.30	0.25	0.25	0.25	0.164	-0.176	0.056
1000	4	1	0.2	0.5	0.5	0.30	0.30	0.30	0.25	0.25	0.50	0.165	-0.175	0.056
1000	4	1	0.2	0.5	0.5	0.30	0.30	0.30	0.25	0.50	0.00	0.179	-0.188	0.047
1000	4	1	0.2	0.5	0.5	0.30	0.30	0.30	0.25	0.50	0.25	0.179	-0.188	0.047
1000	4	1	0.2	0.5	0.5	0.30	0.30	0.30	0.25	0.50	0.50	0.178	-0.189	0.047
1000	4	1	0.2	0.5	0.5	0.30	0.30	0.30	0.50	0.00	0.00	0.148	-0.108	0.064
1000	4	1	0.2	0.5	0.5	0.30	0.30	0.30	0.50	0.00	0.25	0.149	-0.107	0.065

(continued)

N	m_1	$\frac{m_2}{m_1}$	$E(C)$	$E(R)$	$E(U)$	σ_C	σ_R	σ_U	ρ_{CR}	ρ_{CU}	ρ_{RU}	Mean Bias c	Mean Bias r	Mean Bias u
1000	4	1	0.2	0.5	0.5	0.30	0.30	0.30	0.50	0.00	0.50	0.148	-0.107	0.065
1000	4	1	0.2	0.5	0.5	0.30	0.30	0.30	0.50	0.25	0.00	0.165	-0.125	0.056
1000	4	1	0.2	0.5	0.5	0.30	0.30	0.30	0.50	0.25	0.25	0.165	-0.124	0.056
1000	4	1	0.2	0.5	0.5	0.30	0.30	0.30	0.50	0.25	0.50	0.164	-0.125	0.057
1000	4	1	0.2	0.5	0.5	0.30	0.30	0.30	0.50	0.50	0.00	0.179	-0.139	0.047
1000	4	1	0.2	0.5	0.5	0.30	0.30	0.30	0.50	0.50	0.25	0.178	-0.138	0.047
1000	4	1	0.2	0.5	0.5	0.30	0.30	0.30	0.50	0.50	0.50	0.179	-0.138	0.048
1000	4	1	0.2	0.8	0.5	0.00	0.00	0.00	0.00	0.00	0.00	0.000	0.002	0.000
1000	4	1	0.2	0.8	0.5	0.15	0.15	0.15	0.00	0.00	0.00	0.040	-0.131	0.016
1000	4	1	0.2	0.8	0.5	0.15	0.15	0.15	0.00	0.00	0.25	0.039	-0.132	0.016
1000	4	1	0.2	0.8	0.5	0.15	0.15	0.15	0.00	0.00	0.50	0.039	-0.131	0.016
1000	4	1	0.2	0.8	0.5	0.15	0.15	0.15	0.00	0.25	0.00	0.044	-0.145	0.014
1000	4	1	0.2	0.8	0.5	0.15	0.15	0.15	0.00	0.25	0.25	0.044	-0.144	0.013
1000	4	1	0.2	0.8	0.5	0.15	0.15	0.15	0.00	0.25	0.50	0.044	-0.143	0.013
1000	4	1	0.2	0.8	0.5	0.15	0.15	0.15	0.00	0.50	0.00	0.048	-0.155	0.011
1000	4	1	0.2	0.8	0.5	0.15	0.15	0.15	0.00	0.50	0.25	0.048	-0.153	0.011
1000	4	1	0.2	0.8	0.5	0.15	0.15	0.15	0.00	0.50	0.50	0.048	-0.155	0.011
1000	4	1	0.2	0.8	0.5	0.15	0.15	0.15	0.25	0.00	0.00	0.040	-0.112	0.016
1000	4	1	0.2	0.8	0.5	0.15	0.15	0.15	0.25	0.00	0.25	0.040	-0.110	0.016
1000	4	1	0.2	0.8	0.5	0.15	0.15	0.15	0.25	0.00	0.50	0.039	-0.110	0.016
1000	4	1	0.2	0.8	0.5	0.15	0.15	0.15	0.25	0.25	0.00	0.044	-0.124	0.013
1000	4	1	0.2	0.8	0.5	0.15	0.15	0.15	0.25	0.25	0.25	0.044	-0.122	0.014
1000	4	1	0.2	0.8	0.5	0.15	0.15	0.15	0.25	0.25	0.50	0.044	-0.124	0.014
1000	4	1	0.2	0.8	0.5	0.15	0.15	0.15	0.25	0.50	0.00	0.048	-0.133	0.011
1000	4	1	0.2	0.8	0.5	0.15	0.15	0.15	0.25	0.50	0.25	0.049	-0.137	0.011
1000	4	1	0.2	0.8	0.5	0.15	0.15	0.15	0.25	0.50	0.50	0.049	-0.135	0.011
1000	4	1	0.2	0.8	0.5	0.15	0.15	0.15	0.50	0.00	0.00	0.039	-0.090	0.015
1000	4	1	0.2	0.8	0.5	0.15	0.15	0.15	0.50	0.00	0.25	0.040	-0.090	0.016
1000	4	1	0.2	0.8	0.5	0.15	0.15	0.15	0.50	0.00	0.50	0.040	-0.091	0.016
1000	4	1	0.2	0.8	0.5	0.15	0.15	0.15	0.50	0.25	0.00	0.044	-0.106	0.014
1000	4	1	0.2	0.8	0.5	0.15	0.15	0.15	0.50	0.25	0.25	0.043	-0.102	0.013
1000	4	1	0.2	0.8	0.5	0.15	0.15	0.15	0.50	0.25	0.50	0.044	-0.103	0.014
1000	4	1	0.2	0.8	0.5	0.15	0.15	0.15	0.50	0.50	0.00	0.049	-0.118	0.011
1000	4	1	0.2	0.8	0.5	0.15	0.15	0.15	0.50	0.50	0.25	0.048	-0.115	0.011
1000	4	1	0.2	0.8	0.5	0.15	0.15	0.15	0.50	0.50	0.50	0.049	-0.115	0.011
1000	4	1	0.2	0.8	0.5	0.30	0.30	0.30	0.00	0.00	0.00	0.148	-0.340	0.064
1000	4	1	0.2	0.8	0.5	0.30	0.30	0.30	0.00	0.00	0.25	0.147	-0.340	0.065
1000	4	1	0.2	0.8	0.5	0.30	0.30	0.30	0.00	0.00	0.50	0.148	-0.340	0.064
1000	4	1	0.2	0.8	0.5	0.30	0.30	0.30	0.00	0.25	0.00	0.165	-0.361	0.056

(continued)

												Mean Bias		
N	m_1	$\frac{m_2}{m_1}$	$E(C)$	$E(R)$	$E(U)$	σ_C	σ_R	σ_U	ρ_{CR}	ρ_{CU}	ρ_{RU}	c	r	u
1000	4	1	0.2	0.8	0.5	0.30	0.30	0.30	0.00	0.25	0.25	0.165	-0.362	0.056
1000	4	1	0.2	0.8	0.5	0.30	0.30	0.30	0.00	0.25	0.50	0.165	-0.361	0.056
1000	4	1	0.2	0.8	0.5	0.30	0.30	0.30	0.00	0.50	0.00	0.179	-0.377	0.047
1000	4	1	0.2	0.8	0.5	0.30	0.30	0.30	0.00	0.50	0.25	0.178	-0.377	0.047
1000	4	1	0.2	0.8	0.5	0.30	0.30	0.30	0.00	0.50	0.50	0.178	-0.377	0.047
1000	4	1	0.2	0.8	0.5	0.30	0.30	0.30	0.25	0.00	0.00	0.148	-0.299	0.064
1000	4	1	0.2	0.8	0.5	0.30	0.30	0.30	0.25	0.00	0.25	0.148	-0.298	0.065
1000	4	1	0.2	0.8	0.5	0.30	0.30	0.30	0.25	0.00	0.50	0.147	-0.298	0.064
1000	4	1	0.2	0.8	0.5	0.30	0.30	0.30	0.25	0.25	0.00	0.164	-0.320	0.056
1000	4	1	0.2	0.8	0.5	0.30	0.30	0.30	0.25	0.25	0.25	0.165	-0.322	0.056
1000	4	1	0.2	0.8	0.5	0.30	0.30	0.30	0.25	0.25	0.50	0.164	-0.321	0.056
1000	4	1	0.2	0.8	0.5	0.30	0.30	0.30	0.25	0.50	0.00	0.178	-0.340	0.046
1000	4	1	0.2	0.8	0.5	0.30	0.30	0.30	0.25	0.50	0.25	0.179	-0.339	0.047
1000	4	1	0.2	0.8	0.5	0.30	0.30	0.30	0.25	0.50	0.50	0.179	-0.339	0.047
1000	4	1	0.2	0.8	0.5	0.30	0.30	0.30	0.50	0.00	0.00	0.148	-0.263	0.064
1000	4	1	0.2	0.8	0.5	0.30	0.30	0.30	0.50	0.00	0.25	0.148	-0.263	0.064
1000	4	1	0.2	0.8	0.5	0.30	0.30	0.30	0.50	0.00	0.50	0.148	-0.263	0.064
1000	4	1	0.2	0.8	0.5	0.30	0.30	0.30	0.50	0.25	0.00	0.165	-0.287	0.056
1000	4	1	0.2	0.8	0.5	0.30	0.30	0.30	0.50	0.25	0.25	0.164	-0.289	0.057
1000	4	1	0.2	0.8	0.5	0.30	0.30	0.30	0.50	0.25	0.50	0.165	-0.287	0.056
1000	4	1	0.2	0.8	0.5	0.30	0.30	0.30	0.50	0.50	0.00	0.179	-0.307	0.047
1000	4	1	0.2	0.8	0.5	0.30	0.30	0.30	0.50	0.50	0.25	0.178	-0.307	0.047
1000	4	1	0.2	0.8	0.5	0.30	0.30	0.30	0.50	0.50	0.50	0.179	-0.307	0.047
1000	4	1	0.5	0.2	0.5	0.00	0.00	0.00	0.00	0.00	0.00	0.000	0.000	0.000
1000	4	1	0.5	0.2	0.5	0.15	0.15	0.15	0.00	0.00	0.00	0.022	-0.009	0.011
1000	4	1	0.5	0.2	0.5	0.15	0.15	0.15	0.00	0.00	0.25	0.022	-0.009	0.011
1000	4	1	0.5	0.2	0.5	0.15	0.15	0.15	0.00	0.00	0.50	0.022	-0.009	0.011
1000	4	1	0.5	0.2	0.5	0.15	0.15	0.15	0.00	0.25	0.00	0.028	-0.010	0.008
1000	4	1	0.5	0.2	0.5	0.15	0.15	0.15	0.00	0.25	0.25	0.028	-0.011	0.009
1000	4	1	0.5	0.2	0.5	0.15	0.15	0.15	0.00	0.25	0.50	0.027	-0.011	0.008
1000	4	1	0.5	0.2	0.5	0.15	0.15	0.15	0.00	0.50	0.00	0.033	-0.012	0.006
1000	4	1	0.5	0.2	0.5	0.15	0.15	0.15	0.00	0.50	0.25	0.033	-0.013	0.005
1000	4	1	0.5	0.2	0.5	0.15	0.15	0.15	0.00	0.50	0.50	0.033	-0.012	0.006
1000	4	1	0.5	0.2	0.5	0.15	0.15	0.15	0.25	0.00	0.00	0.022	0.001	0.011
1000	4	1	0.5	0.2	0.5	0.15	0.15	0.15	0.25	0.00	0.25	0.022	0.002	0.011
1000	4	1	0.5	0.2	0.5	0.15	0.15	0.15	0.25	0.00	0.50	0.022	0.002	0.011
1000	4	1	0.5	0.2	0.5	0.15	0.15	0.15	0.25	0.25	0.00	0.028	0.000	0.009
1000	4	1	0.5	0.2	0.5	0.15	0.15	0.15	0.25	0.25	0.25	0.029	-0.001	0.009
1000	4	1	0.5	0.2	0.5	0.15	0.15	0.15	0.25	0.25	0.50	0.027	0.000	0.008

(continued)

N	m_1	$\frac{m_2}{m_1}$	$E(C)$	$E(R)$	$E(U)$	σ_C	σ_R	σ_U	ρ_{CR}	ρ_{CU}	ρ_{RU}	Mean Bias c	Mean Bias r	Mean Bias u
1000	4	1	0.5	0.2	0.5	0.15	0.15	0.15	0.25	0.50	0.00	0.034	-0.003	0.006
1000	4	1	0.5	0.2	0.5	0.15	0.15	0.15	0.25	0.50	0.25	0.033	-0.003	0.005
1000	4	1	0.5	0.2	0.5	0.15	0.15	0.15	0.25	0.50	0.50	0.033	-0.003	0.006
1000	4	1	0.5	0.2	0.5	0.15	0.15	0.15	0.50	0.00	0.00	0.022	0.012	0.012
1000	4	1	0.5	0.2	0.5	0.15	0.15	0.15	0.50	0.00	0.25	0.022	0.012	0.011
1000	4	1	0.5	0.2	0.5	0.15	0.15	0.15	0.50	0.00	0.50	0.022	0.012	0.011
1000	4	1	0.5	0.2	0.5	0.15	0.15	0.15	0.50	0.25	0.00	0.028	0.009	0.008
1000	4	1	0.5	0.2	0.5	0.15	0.15	0.15	0.50	0.25	0.25	0.028	0.010	0.009
1000	4	1	0.5	0.2	0.5	0.15	0.15	0.15	0.50	0.25	0.50	0.028	0.010	0.009
1000	4	1	0.5	0.2	0.5	0.15	0.15	0.15	0.50	0.50	0.00	0.033	0.007	0.006
1000	4	1	0.5	0.2	0.5	0.15	0.15	0.15	0.50	0.50	0.25	0.034	0.007	0.006
1000	4	1	0.5	0.2	0.5	0.15	0.15	0.15	0.50	0.50	0.50	0.033	0.007	0.005
1000	4	1	0.5	0.2	0.5	0.30	0.30	0.30	0.00	0.00	0.00	0.084	-0.029	0.046
1000	4	1	0.5	0.2	0.5	0.30	0.30	0.30	0.00	0.00	0.25	0.085	-0.029	0.046
1000	4	1	0.5	0.2	0.5	0.30	0.30	0.30	0.00	0.00	0.50	0.084	-0.029	0.046
1000	4	1	0.5	0.2	0.5	0.30	0.30	0.30	0.00	0.25	0.00	0.106	-0.035	0.036
1000	4	1	0.5	0.2	0.5	0.30	0.30	0.30	0.00	0.25	0.25	0.106	-0.035	0.035
1000	4	1	0.5	0.2	0.5	0.30	0.30	0.30	0.00	0.25	0.50	0.106	-0.035	0.035
1000	4	1	0.5	0.2	0.5	0.30	0.30	0.30	0.00	0.50	0.00	0.129	-0.041	0.025
1000	4	1	0.5	0.2	0.5	0.30	0.30	0.30	0.00	0.50	0.25	0.129	-0.041	0.024
1000	4	1	0.5	0.2	0.5	0.30	0.30	0.30	0.00	0.50	0.50	0.130	-0.041	0.025
1000	4	1	0.5	0.2	0.5	0.30	0.30	0.30	0.25	0.00	0.00	0.083	0.002	0.046
1000	4	1	0.5	0.2	0.5	0.30	0.30	0.30	0.25	0.00	0.25	0.084	0.002	0.046
1000	4	1	0.5	0.2	0.5	0.30	0.30	0.30	0.25	0.00	0.50	0.083	0.003	0.045
1000	4	1	0.5	0.2	0.5	0.30	0.30	0.30	0.25	0.25	0.00	0.106	-0.005	0.036
1000	4	1	0.5	0.2	0.5	0.30	0.30	0.30	0.25	0.25	0.25	0.106	-0.004	0.036
1000	4	1	0.5	0.2	0.5	0.30	0.30	0.30	0.25	0.25	0.50	0.107	-0.005	0.035
1000	4	1	0.5	0.2	0.5	0.30	0.30	0.30	0.25	0.50	0.00	0.129	-0.012	0.025
1000	4	1	0.5	0.2	0.5	0.30	0.30	0.30	0.25	0.50	0.25	0.130	-0.012	0.025
1000	4	1	0.5	0.2	0.5	0.30	0.30	0.30	0.25	0.50	0.50	0.129	-0.012	0.024
1000	4	1	0.5	0.2	0.5	0.30	0.30	0.30	0.50	0.00	0.00	0.084	0.034	0.046
1000	4	1	0.5	0.2	0.5	0.30	0.30	0.30	0.50	0.00	0.25	0.084	0.034	0.045
1000	4	1	0.5	0.2	0.5	0.30	0.30	0.30	0.50	0.00	0.50	0.084	0.035	0.046
1000	4	1	0.5	0.2	0.5	0.30	0.30	0.30	0.50	0.25	0.00	0.107	0.026	0.036
1000	4	1	0.5	0.2	0.5	0.30	0.30	0.30	0.50	0.25	0.25	0.106	0.026	0.036
1000	4	1	0.5	0.2	0.5	0.30	0.30	0.30	0.50	0.25	0.50	0.107	0.026	0.036
1000	4	1	0.5	0.2	0.5	0.30	0.30	0.30	0.50	0.50	0.00	0.130	0.017	0.026
1000	4	1	0.5	0.2	0.5	0.30	0.30	0.30	0.50	0.50	0.25	0.129	0.017	0.025
1000	4	1	0.5	0.2	0.5	0.30	0.30	0.30	0.50	0.50	0.50	0.129	0.017	0.025

(continued)

												Mean Bias		
N	m_1	$\frac{m_2}{m_1}$	$E(C)$	$E(R)$	$E(U)$	σ_C	σ_R	σ_U	ρ_{CR}	ρ_{CU}	ρ_{RU}	c	r	u
1000	4	1	0.5	0.5	0.5	0.00	0.00	0.00	0.00	0.00	0.00	0.000	0.000	0.000
1000	4	1	0.5	0.5	0.5	0.15	0.15	0.15	0.00	0.00	0.00	0.022	-0.021	0.011
1000	4	1	0.5	0.5	0.5	0.15	0.15	0.15	0.00	0.00	0.25	0.022	-0.021	0.011
1000	4	1	0.5	0.5	0.5	0.15	0.15	0.15	0.00	0.00	0.50	0.023	-0.021	0.011
1000	4	1	0.5	0.5	0.5	0.15	0.15	0.15	0.00	0.25	0.00	0.028	-0.027	0.009
1000	4	1	0.5	0.5	0.5	0.15	0.15	0.15	0.00	0.25	0.25	0.027	-0.026	0.009
1000	4	1	0.5	0.5	0.5	0.15	0.15	0.15	0.00	0.25	0.50	0.028	-0.026	0.008
1000	4	1	0.5	0.5	0.5	0.15	0.15	0.15	0.00	0.50	0.00	0.033	-0.031	0.006
1000	4	1	0.5	0.5	0.5	0.15	0.15	0.15	0.00	0.50	0.25	0.033	-0.031	0.006
1000	4	1	0.5	0.5	0.5	0.15	0.15	0.15	0.00	0.50	0.50	0.033	-0.031	0.006
1000	4	1	0.5	0.5	0.5	0.15	0.15	0.15	0.25	0.00	0.00	0.022	-0.010	0.011
1000	4	1	0.5	0.5	0.5	0.15	0.15	0.15	0.25	0.00	0.25	0.022	-0.010	0.011
1000	4	1	0.5	0.5	0.5	0.15	0.15	0.15	0.25	0.00	0.50	0.022	-0.009	0.011
1000	4	1	0.5	0.5	0.5	0.15	0.15	0.15	0.25	0.25	0.00	0.028	-0.015	0.009
1000	4	1	0.5	0.5	0.5	0.15	0.15	0.15	0.25	0.25	0.25	0.028	-0.016	0.009
1000	4	1	0.5	0.5	0.5	0.15	0.15	0.15	0.25	0.25	0.50	0.028	-0.016	0.009
1000	4	1	0.5	0.5	0.5	0.15	0.15	0.15	0.25	0.50	0.00	0.033	-0.021	0.006
1000	4	1	0.5	0.5	0.5	0.15	0.15	0.15	0.25	0.50	0.25	0.033	-0.021	0.006
1000	4	1	0.5	0.5	0.5	0.15	0.15	0.15	0.25	0.50	0.50	0.033	-0.021	0.005
1000	4	1	0.5	0.5	0.5	0.15	0.15	0.15	0.50	0.00	0.00	0.022	0.000	0.011
1000	4	1	0.5	0.5	0.5	0.15	0.15	0.15	0.50	0.00	0.25	0.022	0.000	0.011
1000	4	1	0.5	0.5	0.5	0.15	0.15	0.15	0.50	0.00	0.50	0.022	0.001	0.011
1000	4	1	0.5	0.5	0.5	0.15	0.15	0.15	0.50	0.25	0.00	0.028	-0.005	0.009
1000	4	1	0.5	0.5	0.5	0.15	0.15	0.15	0.50	0.25	0.25	0.028	-0.005	0.009
1000	4	1	0.5	0.5	0.5	0.15	0.15	0.15	0.50	0.25	0.50	0.028	-0.005	0.009
1000	4	1	0.5	0.5	0.5	0.15	0.15	0.15	0.50	0.50	0.00	0.034	-0.010	0.006
1000	4	1	0.5	0.5	0.5	0.15	0.15	0.15	0.50	0.50	0.25	0.033	-0.011	0.005
1000	4	1	0.5	0.5	0.5	0.15	0.15	0.15	0.50	0.50	0.50	0.033	-0.010	0.005
1000	4	1	0.5	0.5	0.5	0.30	0.30	0.30	0.00	0.00	0.00	0.085	-0.072	0.046
1000	4	1	0.5	0.5	0.5	0.30	0.30	0.30	0.00	0.00	0.25	0.083	-0.072	0.046
1000	4	1	0.5	0.5	0.5	0.30	0.30	0.30	0.00	0.00	0.50	0.084	-0.072	0.046
1000	4	1	0.5	0.5	0.5	0.30	0.30	0.30	0.00	0.25	0.00	0.106	-0.088	0.035
1000	4	1	0.5	0.5	0.5	0.30	0.30	0.30	0.00	0.25	0.25	0.107	-0.088	0.036
1000	4	1	0.5	0.5	0.5	0.30	0.30	0.30	0.00	0.25	0.50	0.107	-0.088	0.036
1000	4	1	0.5	0.5	0.5	0.30	0.30	0.30	0.00	0.50	0.00	0.129	-0.103	0.024
1000	4	1	0.5	0.5	0.5	0.30	0.30	0.30	0.00	0.50	0.25	0.130	-0.103	0.024
1000	4	1	0.5	0.5	0.5	0.30	0.30	0.30	0.00	0.50	0.50	0.130	-0.103	0.025
1000	4	1	0.5	0.5	0.5	0.30	0.30	0.30	0.25	0.00	0.00	0.084	-0.036	0.045
1000	4	1	0.5	0.5	0.5	0.30	0.30	0.30	0.25	0.00	0.25	0.084	-0.036	0.046

(continued)

												Mean Bias		
N	m_1	$\frac{m_2}{m_1}$	$E(C)$	$E(R)$	$E(U)$	σ_C	σ_R	σ_U	ρ_{CR}	ρ_{CU}	ρ_{RU}	c	r	u
1000	4	1	0.5	0.5	0.5	0.30	0.30	0.30	0.25	0.00	0.50	0.084	-0.036	0.046
1000	4	1	0.5	0.5	0.5	0.30	0.30	0.30	0.25	0.25	0.00	0.107	-0.052	0.035
1000	4	1	0.5	0.5	0.5	0.30	0.30	0.30	0.25	0.25	0.25	0.107	-0.053	0.035
1000	4	1	0.5	0.5	0.5	0.30	0.30	0.30	0.25	0.25	0.50	0.106	-0.052	0.036
1000	4	1	0.5	0.5	0.5	0.30	0.30	0.30	0.25	0.50	0.00	0.129	-0.069	0.025
1000	4	1	0.5	0.5	0.5	0.30	0.30	0.30	0.25	0.50	0.25	0.129	-0.068	0.025
1000	4	1	0.5	0.5	0.5	0.30	0.30	0.30	0.25	0.50	0.50	0.130	-0.069	0.024
1000	4	1	0.5	0.5	0.5	0.30	0.30	0.30	0.50	0.00	0.00	0.084	0.002	0.046
1000	4	1	0.5	0.5	0.5	0.30	0.30	0.30	0.50	0.00	0.25	0.084	0.002	0.046
1000	4	1	0.5	0.5	0.5	0.30	0.30	0.30	0.50	0.00	0.50	0.084	0.003	0.046
1000	4	1	0.5	0.5	0.5	0.30	0.30	0.30	0.50	0.25	0.00	0.107	-0.016	0.036
1000	4	1	0.5	0.5	0.5	0.30	0.30	0.30	0.50	0.25	0.25	0.106	-0.017	0.035
1000	4	1	0.5	0.5	0.5	0.30	0.30	0.30	0.50	0.25	0.50	0.106	-0.016	0.036
1000	4	1	0.5	0.5	0.5	0.30	0.30	0.30	0.50	0.50	0.00	0.129	-0.034	0.025
1000	4	1	0.5	0.5	0.5	0.30	0.30	0.30	0.50	0.50	0.25	0.129	-0.035	0.025
1000	4	1	0.5	0.5	0.5	0.30	0.30	0.30	0.50	0.50	0.50	0.130	-0.034	0.025
1000	4	1	0.5	0.8	0.5	0.00	0.00	0.00	0.00	0.00	0.00	0.000	0.001	0.000
1000	4	1	0.5	0.8	0.5	0.15	0.15	0.15	0.00	0.00	0.00	0.022	-0.034	0.011
1000	4	1	0.5	0.8	0.5	0.15	0.15	0.15	0.00	0.00	0.25	0.022	-0.034	0.012
1000	4	1	0.5	0.8	0.5	0.15	0.15	0.15	0.00	0.00	0.50	0.022	-0.034	0.011
1000	4	1	0.5	0.8	0.5	0.15	0.15	0.15	0.00	0.25	0.00	0.028	-0.042	0.008
1000	4	1	0.5	0.8	0.5	0.15	0.15	0.15	0.00	0.25	0.25	0.028	-0.042	0.009
1000	4	1	0.5	0.8	0.5	0.15	0.15	0.15	0.00	0.25	0.50	0.028	-0.042	0.008
1000	4	1	0.5	0.8	0.5	0.15	0.15	0.15	0.00	0.50	0.00	0.034	-0.050	0.006
1000	4	1	0.5	0.8	0.5	0.15	0.15	0.15	0.00	0.50	0.25	0.034	-0.050	0.006
1000	4	1	0.5	0.8	0.5	0.15	0.15	0.15	0.00	0.50	0.50	0.033	-0.049	0.006
1000	4	1	0.5	0.8	0.5	0.15	0.15	0.15	0.25	0.00	0.00	0.022	-0.024	0.012
1000	4	1	0.5	0.8	0.5	0.15	0.15	0.15	0.25	0.00	0.25	0.022	-0.024	0.011
1000	4	1	0.5	0.8	0.5	0.15	0.15	0.15	0.25	0.00	0.50	0.023	-0.023	0.011
1000	4	1	0.5	0.8	0.5	0.15	0.15	0.15	0.25	0.25	0.00	0.027	-0.032	0.009
1000	4	1	0.5	0.8	0.5	0.15	0.15	0.15	0.25	0.25	0.25	0.028	-0.032	0.009
1000	4	1	0.5	0.8	0.5	0.15	0.15	0.15	0.25	0.25	0.50	0.028	-0.032	0.008
1000	4	1	0.5	0.8	0.5	0.15	0.15	0.15	0.25	0.50	0.00	0.034	-0.040	0.006
1000	4	1	0.5	0.8	0.5	0.15	0.15	0.15	0.25	0.50	0.25	0.033	-0.039	0.005
1000	4	1	0.5	0.8	0.5	0.15	0.15	0.15	0.25	0.50	0.50	0.033	-0.039	0.006
1000	4	1	0.5	0.8	0.5	0.15	0.15	0.15	0.50	0.00	0.00	0.022	-0.012	0.011
1000	4	1	0.5	0.8	0.5	0.15	0.15	0.15	0.50	0.00	0.25	0.022	-0.014	0.011
1000	4	1	0.5	0.8	0.5	0.15	0.15	0.15	0.50	0.00	0.50	0.022	-0.013	0.011
1000	4	1	0.5	0.8	0.5	0.15	0.15	0.15	0.50	0.25	0.00	0.028	-0.021	0.008

(continued)

N	m_1	$\frac{m_2}{m_1}$	$E(C)$	$E(R)$	$E(U)$	σ_C	σ_R	σ_U	ρ_{CR}	ρ_{CU}	ρ_{RU}	Mean Bias		
												c	r	u
1000	4	1	0.5	0.8	0.5	0.15	0.15	0.15	0.50	0.25	0.25	0.027	-0.021	0.009
1000	4	1	0.5	0.8	0.5	0.15	0.15	0.15	0.50	0.25	0.50	0.028	-0.022	0.009
1000	4	1	0.5	0.8	0.5	0.15	0.15	0.15	0.50	0.50	0.00	0.033	-0.030	0.005
1000	4	1	0.5	0.8	0.5	0.15	0.15	0.15	0.50	0.50	0.25	0.033	-0.030	0.005
1000	4	1	0.5	0.8	0.5	0.15	0.15	0.15	0.50	0.50	0.50	0.033	-0.030	0.006
1000	4	1	0.5	0.8	0.5	0.30	0.30	0.30	0.00	0.00	0.00	0.084	-0.115	0.047
1000	4	1	0.5	0.8	0.5	0.30	0.30	0.30	0.00	0.00	0.25	0.084	-0.115	0.045
1000	4	1	0.5	0.8	0.5	0.30	0.30	0.30	0.00	0.00	0.50	0.084	-0.116	0.046
1000	4	1	0.5	0.8	0.5	0.30	0.30	0.30	0.00	0.25	0.00	0.107	-0.141	0.036
1000	4	1	0.5	0.8	0.5	0.30	0.30	0.30	0.00	0.25	0.25	0.107	-0.141	0.035
1000	4	1	0.5	0.8	0.5	0.30	0.30	0.30	0.00	0.25	0.50	0.106	-0.140	0.036
1000	4	1	0.5	0.8	0.5	0.30	0.30	0.30	0.00	0.50	0.00	0.129	-0.165	0.024
1000	4	1	0.5	0.8	0.5	0.30	0.30	0.30	0.00	0.50	0.25	0.129	-0.164	0.025
1000	4	1	0.5	0.8	0.5	0.30	0.30	0.30	0.00	0.50	0.50	0.130	-0.164	0.025
1000	4	1	0.5	0.8	0.5	0.30	0.30	0.30	0.25	0.00	0.00	0.085	-0.084	0.046
1000	4	1	0.5	0.8	0.5	0.30	0.30	0.30	0.25	0.00	0.25	0.084	-0.084	0.046
1000	4	1	0.5	0.8	0.5	0.30	0.30	0.30	0.25	0.00	0.50	0.085	-0.085	0.046
1000	4	1	0.5	0.8	0.5	0.30	0.30	0.30	0.25	0.25	0.00	0.106	-0.109	0.035
1000	4	1	0.5	0.8	0.5	0.30	0.30	0.30	0.25	0.25	0.25	0.107	-0.111	0.036
1000	4	1	0.5	0.8	0.5	0.30	0.30	0.30	0.25	0.25	0.50	0.106	-0.110	0.035
1000	4	1	0.5	0.8	0.5	0.30	0.30	0.30	0.25	0.50	0.00	0.130	-0.135	0.025
1000	4	1	0.5	0.8	0.5	0.30	0.30	0.30	0.25	0.50	0.25	0.130	-0.135	0.025
1000	4	1	0.5	0.8	0.5	0.30	0.30	0.30	0.25	0.50	0.50	0.129	-0.135	0.025
1000	4	1	0.5	0.8	0.5	0.30	0.30	0.30	0.50	0.00	0.00	0.084	-0.051	0.047
1000	4	1	0.5	0.8	0.5	0.30	0.30	0.30	0.50	0.00	0.25	0.084	-0.052	0.046
1000	4	1	0.5	0.8	0.5	0.30	0.30	0.30	0.50	0.00	0.50	0.084	-0.053	0.046
1000	4	1	0.5	0.8	0.5	0.30	0.30	0.30	0.50	0.25	0.00	0.106	-0.079	0.036
1000	4	1	0.5	0.8	0.5	0.30	0.30	0.30	0.50	0.25	0.25	0.106	-0.079	0.035
1000	4	1	0.5	0.8	0.5	0.30	0.30	0.30	0.50	0.25	0.50	0.106	-0.080	0.035
1000	4	1	0.5	0.8	0.5	0.30	0.30	0.30	0.50	0.50	0.00	0.130	-0.107	0.025
1000	4	1	0.5	0.8	0.5	0.30	0.30	0.30	0.50	0.50	0.25	0.130	-0.107	0.025
1000	4	1	0.5	0.8	0.5	0.30	0.30	0.30	0.50	0.50	0.50	0.130	-0.106	0.025
1000	4	1	0.8	0.2	0.5	0.00	0.00	0.00	0.00	0.00	0.00	0.000	0.001	0.000
1000	4	1	0.8	0.2	0.5	0.15	0.15	0.15	0.00	0.00	0.00	0.007	-0.002	0.005
1000	4	1	0.8	0.2	0.5	0.15	0.15	0.15	0.00	0.00	0.25	0.008	-0.002	0.006
1000	4	1	0.8	0.2	0.5	0.15	0.15	0.15	0.00	0.00	0.50	0.007	-0.002	0.005
1000	4	1	0.8	0.2	0.5	0.15	0.15	0.15	0.00	0.25	0.00	0.014	-0.003	0.002
1000	4	1	0.8	0.2	0.5	0.15	0.15	0.15	0.00	0.25	0.25	0.014	-0.003	0.003
1000	4	1	0.8	0.2	0.5	0.15	0.15	0.15	0.00	0.25	0.50	0.014	-0.004	0.003

(continued)

												Mean Bias		
N	m_1	$\frac{m_2}{m_1}$	$E(C)$	$E(R)$	$E(U)$	σ_C	σ_R	σ_U	ρ_{CR}	ρ_{CU}	ρ_{RU}	c	r	u
1000	4	1	0.8	0.2	0.5	0.15	0.15	0.15	0.00	0.50	0.00	0.020	-0.006	-0.001
1000	4	1	0.8	0.2	0.5	0.15	0.15	0.15	0.00	0.50	0.25	0.021	-0.005	-0.001
1000	4	1	0.8	0.2	0.5	0.15	0.15	0.15	0.00	0.50	0.50	0.021	-0.005	-0.001
1000	4	1	0.8	0.2	0.5	0.15	0.15	0.15	0.25	0.00	0.00	0.007	0.005	0.005
1000	4	1	0.8	0.2	0.5	0.15	0.15	0.15	0.25	0.00	0.25	0.007	0.005	0.005
1000	4	1	0.8	0.2	0.5	0.15	0.15	0.15	0.25	0.00	0.50	0.008	0.005	0.005
1000	4	1	0.8	0.2	0.5	0.15	0.15	0.15	0.25	0.25	0.00	0.014	0.003	0.002
1000	4	1	0.8	0.2	0.5	0.15	0.15	0.15	0.25	0.25	0.25	0.014	0.003	0.002
1000	4	1	0.8	0.2	0.5	0.15	0.15	0.15	0.25	0.25	0.50	0.013	0.002	0.002
1000	4	1	0.8	0.2	0.5	0.15	0.15	0.15	0.25	0.50	0.00	0.021	0.001	-0.001
1000	4	1	0.8	0.2	0.5	0.15	0.15	0.15	0.25	0.50	0.25	0.021	0.001	0.000
1000	4	1	0.8	0.2	0.5	0.15	0.15	0.15	0.25	0.50	0.50	0.020	0.001	-0.001
1000	4	1	0.8	0.2	0.5	0.15	0.15	0.15	0.50	0.00	0.00	0.007	0.010	0.005
1000	4	1	0.8	0.2	0.5	0.15	0.15	0.15	0.50	0.00	0.25	0.008	0.010	0.006
1000	4	1	0.8	0.2	0.5	0.15	0.15	0.15	0.50	0.00	0.50	0.007	0.011	0.005
1000	4	1	0.8	0.2	0.5	0.15	0.15	0.15	0.50	0.25	0.00	0.013	0.009	0.002
1000	4	1	0.8	0.2	0.5	0.15	0.15	0.15	0.50	0.25	0.25	0.014	0.009	0.002
1000	4	1	0.8	0.2	0.5	0.15	0.15	0.15	0.50	0.25	0.50	0.014	0.008	0.002
1000	4	1	0.8	0.2	0.5	0.15	0.15	0.15	0.50	0.50	0.00	0.020	0.007	-0.001
1000	4	1	0.8	0.2	0.5	0.15	0.15	0.15	0.50	0.50	0.25	0.021	0.007	-0.001
1000	4	1	0.8	0.2	0.5	0.15	0.15	0.15	0.50	0.50	0.50	0.021	0.007	-0.001
1000	4	1	0.8	0.2	0.5	0.30	0.30	0.30	0.00	0.00	0.00	0.028	-0.007	0.021
1000	4	1	0.8	0.2	0.5	0.30	0.30	0.30	0.00	0.00	0.25	0.029	-0.007	0.022
1000	4	1	0.8	0.2	0.5	0.30	0.30	0.30	0.00	0.00	0.50	0.029	-0.007	0.022
1000	4	1	0.8	0.2	0.5	0.30	0.30	0.30	0.00	0.25	0.00	0.051	-0.012	0.012
1000	4	1	0.8	0.2	0.5	0.30	0.30	0.30	0.00	0.25	0.25	0.052	-0.011	0.011
1000	4	1	0.8	0.2	0.5	0.30	0.30	0.30	0.00	0.25	0.50	0.052	-0.012	0.011
1000	4	1	0.8	0.2	0.5	0.30	0.30	0.30	0.00	0.50	0.00	0.077	-0.017	0.002
1000	4	1	0.8	0.2	0.5	0.30	0.30	0.30	0.00	0.50	0.25	0.077	-0.017	0.003
1000	4	1	0.8	0.2	0.5	0.30	0.30	0.30	0.00	0.50	0.50	0.077	-0.018	0.002
1000	4	1	0.8	0.2	0.5	0.30	0.30	0.30	0.25	0.00	0.00	0.029	0.010	0.022
1000	4	1	0.8	0.2	0.5	0.30	0.30	0.30	0.25	0.00	0.25	0.029	0.011	0.021
1000	4	1	0.8	0.2	0.5	0.30	0.30	0.30	0.25	0.00	0.50	0.029	0.011	0.021
1000	4	1	0.8	0.2	0.5	0.30	0.30	0.30	0.25	0.25	0.00	0.051	0.005	0.012
1000	4	1	0.8	0.2	0.5	0.30	0.30	0.30	0.25	0.25	0.25	0.051	0.005	0.011
1000	4	1	0.8	0.2	0.5	0.30	0.30	0.30	0.25	0.25	0.50	0.052	0.005	0.012
1000	4	1	0.8	0.2	0.5	0.30	0.30	0.30	0.25	0.50	0.00	0.077	-0.001	0.002
1000	4	1	0.8	0.2	0.5	0.30	0.30	0.30	0.25	0.50	0.25	0.077	-0.001	0.003
1000	4	1	0.8	0.2	0.5	0.30	0.30	0.30	0.25	0.50	0.50	0.077	-0.001	0.002

(continued)

N	m_1	$\frac{m_2}{m_1}$	$E(C)$	$E(R)$	$E(U)$	σ_C	σ_R	σ_U	ρ_{CR}	ρ_{CU}	ρ_{RU}	Mean Bias c	Mean Bias r	Mean Bias u
1000	4	1	0.8	0.2	0.5	0.30	0.30	0.30	0.50	0.00	0.00	0.029	0.025	0.022
1000	4	1	0.8	0.2	0.5	0.30	0.30	0.30	0.50	0.00	0.25	0.029	0.025	0.021
1000	4	1	0.8	0.2	0.5	0.30	0.30	0.30	0.50	0.00	0.50	0.028	0.026	0.022
1000	4	1	0.8	0.2	0.5	0.30	0.30	0.30	0.50	0.25	0.00	0.052	0.019	0.011
1000	4	1	0.8	0.2	0.5	0.30	0.30	0.30	0.50	0.25	0.25	0.051	0.020	0.011
1000	4	1	0.8	0.2	0.5	0.30	0.30	0.30	0.50	0.25	0.50	0.051	0.020	0.012
1000	4	1	0.8	0.2	0.5	0.30	0.30	0.30	0.50	0.50	0.00	0.077	0.013	0.002
1000	4	1	0.8	0.2	0.5	0.30	0.30	0.30	0.50	0.50	0.25	0.076	0.013	0.003
1000	4	1	0.8	0.2	0.5	0.30	0.30	0.30	0.50	0.50	0.50	0.076	0.013	0.003
1000	4	1	0.8	0.5	0.5	0.00	0.00	0.00	0.00	0.00	0.00	0.000	0.000	0.000
1000	4	1	0.8	0.5	0.5	0.15	0.15	0.15	0.00	0.00	0.00	0.008	-0.004	0.006
1000	4	1	0.8	0.5	0.5	0.15	0.15	0.15	0.00	0.00	0.25	0.007	-0.004	0.005
1000	4	1	0.8	0.5	0.5	0.15	0.15	0.15	0.00	0.00	0.50	0.008	-0.005	0.006
1000	4	1	0.8	0.5	0.5	0.15	0.15	0.15	0.00	0.25	0.00	0.014	-0.009	0.002
1000	4	1	0.8	0.5	0.5	0.15	0.15	0.15	0.00	0.25	0.25	0.014	-0.009	0.002
1000	4	1	0.8	0.5	0.5	0.15	0.15	0.15	0.00	0.25	0.50	0.014	-0.009	0.002
1000	4	1	0.8	0.5	0.5	0.15	0.15	0.15	0.00	0.50	0.00	0.020	-0.013	-0.001
1000	4	1	0.8	0.5	0.5	0.15	0.15	0.15	0.00	0.50	0.25	0.021	-0.013	0.000
1000	4	1	0.8	0.5	0.5	0.15	0.15	0.15	0.00	0.50	0.50	0.020	-0.012	0.000
1000	4	1	0.8	0.5	0.5	0.15	0.15	0.15	0.25	0.00	0.00	0.007	0.002	0.006
1000	4	1	0.8	0.5	0.5	0.15	0.15	0.15	0.25	0.00	0.25	0.007	0.002	0.005
1000	4	1	0.8	0.5	0.5	0.15	0.15	0.15	0.25	0.00	0.50	0.008	0.002	0.005
1000	4	1	0.8	0.5	0.5	0.15	0.15	0.15	0.25	0.25	0.00	0.014	-0.002	0.002
1000	4	1	0.8	0.5	0.5	0.15	0.15	0.15	0.25	0.25	0.25	0.014	-0.002	0.003
1000	4	1	0.8	0.5	0.5	0.15	0.15	0.15	0.25	0.25	0.50	0.014	-0.002	0.003
1000	4	1	0.8	0.5	0.5	0.15	0.15	0.15	0.25	0.50	0.00	0.021	-0.006	0.000
1000	4	1	0.8	0.5	0.5	0.15	0.15	0.15	0.25	0.50	0.25	0.020	-0.006	-0.001
1000	4	1	0.8	0.5	0.5	0.15	0.15	0.15	0.25	0.50	0.50	0.021	-0.006	-0.001
1000	4	1	0.8	0.5	0.5	0.15	0.15	0.15	0.50	0.00	0.00	0.007	0.008	0.006
1000	4	1	0.8	0.5	0.5	0.15	0.15	0.15	0.50	0.00	0.25	0.007	0.009	0.005
1000	4	1	0.8	0.5	0.5	0.15	0.15	0.15	0.50	0.00	0.50	0.007	0.009	0.006
1000	4	1	0.8	0.5	0.5	0.15	0.15	0.15	0.50	0.25	0.00	0.014	0.005	0.002
1000	4	1	0.8	0.5	0.5	0.15	0.15	0.15	0.50	0.25	0.25	0.014	0.005	0.002
1000	4	1	0.8	0.5	0.5	0.15	0.15	0.15	0.50	0.25	0.50	0.014	0.005	0.003
1000	4	1	0.8	0.5	0.5	0.15	0.15	0.15	0.50	0.50	0.00	0.021	0.001	-0.001
1000	4	1	0.8	0.5	0.5	0.15	0.15	0.15	0.50	0.50	0.25	0.021	0.001	-0.001
1000	4	1	0.8	0.5	0.5	0.15	0.15	0.15	0.50	0.50	0.50	0.021	0.001	0.000
1000	4	1	0.8	0.5	0.5	0.30	0.30	0.30	0.00	0.00	0.00	0.029	-0.017	0.021
1000	4	1	0.8	0.5	0.5	0.30	0.30	0.30	0.00	0.00	0.25	0.029	-0.017	0.021

(continued)

												Mean Bias		
N	m_1	$\frac{m_2}{m_1}$	$E(C)$	$E(R)$	$E(U)$	σ_C	σ_R	σ_U	ρ_{CR}	ρ_{CU}	ρ_{RU}	c	r	u
1000	4	1	0.8	0.5	0.5	0.30	0.30	0.30	0.00	0.00	0.50	0.028	-0.017	0.022
1000	4	1	0.8	0.5	0.5	0.30	0.30	0.30	0.00	0.25	0.00	0.051	-0.030	0.012
1000	4	1	0.8	0.5	0.5	0.30	0.30	0.30	0.00	0.25	0.25	0.051	-0.030	0.011
1000	4	1	0.8	0.5	0.5	0.30	0.30	0.30	0.00	0.25	0.50	0.051	-0.030	0.012
1000	4	1	0.8	0.5	0.5	0.30	0.30	0.30	0.00	0.50	0.00	0.077	-0.044	0.003
1000	4	1	0.8	0.5	0.5	0.30	0.30	0.30	0.00	0.50	0.25	0.076	-0.044	0.002
1000	4	1	0.8	0.5	0.5	0.30	0.30	0.30	0.00	0.50	0.50	0.077	-0.043	0.003
1000	4	1	0.8	0.5	0.5	0.30	0.30	0.30	0.25	0.00	0.00	0.029	0.004	0.021
1000	4	1	0.8	0.5	0.5	0.30	0.30	0.30	0.25	0.00	0.25	0.029	0.005	0.021
1000	4	1	0.8	0.5	0.5	0.30	0.30	0.30	0.25	0.00	0.50	0.029	0.004	0.021
1000	4	1	0.8	0.5	0.5	0.30	0.30	0.30	0.25	0.25	0.00	0.051	-0.008	0.011
1000	4	1	0.8	0.5	0.5	0.30	0.30	0.30	0.25	0.25	0.25	0.052	-0.008	0.011
1000	4	1	0.8	0.5	0.5	0.30	0.30	0.30	0.25	0.25	0.50	0.051	-0.009	0.011
1000	4	1	0.8	0.5	0.5	0.30	0.30	0.30	0.25	0.50	0.00	0.077	-0.023	0.003
1000	4	1	0.8	0.5	0.5	0.30	0.30	0.30	0.25	0.50	0.25	0.077	-0.023	0.002
1000	4	1	0.8	0.5	0.5	0.30	0.30	0.30	0.25	0.50	0.50	0.077	-0.023	0.003
1000	4	1	0.8	0.5	0.5	0.30	0.30	0.30	0.50	0.00	0.00	0.029	0.027	0.022
1000	4	1	0.8	0.5	0.5	0.30	0.30	0.30	0.50	0.00	0.25	0.029	0.027	0.021
1000	4	1	0.8	0.5	0.5	0.30	0.30	0.30	0.50	0.00	0.50	0.028	0.028	0.022
1000	4	1	0.8	0.5	0.5	0.30	0.30	0.30	0.50	0.25	0.00	0.051	0.013	0.012
1000	4	1	0.8	0.5	0.5	0.30	0.30	0.30	0.50	0.25	0.25	0.052	0.013	0.011
1000	4	1	0.8	0.5	0.5	0.30	0.30	0.30	0.50	0.25	0.50	0.051	0.013	0.012
1000	4	1	0.8	0.5	0.5	0.30	0.30	0.30	0.50	0.50	0.00	0.076	-0.001	0.002
1000	4	1	0.8	0.5	0.5	0.30	0.30	0.30	0.50	0.50	0.25	0.077	-0.002	0.003
1000	4	1	0.8	0.5	0.5	0.30	0.30	0.30	0.50	0.50	0.50	0.077	-0.002	0.003
1000	4	1	0.8	0.8	0.5	0.00	0.00	0.00	0.00	0.00	0.00	-0.001	0.000	0.000
1000	4	1	0.8	0.8	0.5	0.15	0.15	0.15	0.00	0.00	0.00	0.008	-0.008	0.006
1000	4	1	0.8	0.8	0.5	0.15	0.15	0.15	0.00	0.00	0.25	0.007	-0.007	0.006
1000	4	1	0.8	0.8	0.5	0.15	0.15	0.15	0.00	0.00	0.50	0.008	-0.007	0.006
1000	4	1	0.8	0.8	0.5	0.15	0.15	0.15	0.00	0.25	0.00	0.014	-0.013	0.002
1000	4	1	0.8	0.8	0.5	0.15	0.15	0.15	0.00	0.25	0.25	0.013	-0.014	0.002
1000	4	1	0.8	0.8	0.5	0.15	0.15	0.15	0.00	0.25	0.50	0.014	-0.014	0.002
1000	4	1	0.8	0.8	0.5	0.15	0.15	0.15	0.00	0.50	0.00	0.020	-0.020	-0.001
1000	4	1	0.8	0.8	0.5	0.15	0.15	0.15	0.00	0.50	0.25	0.021	-0.020	-0.001
1000	4	1	0.8	0.8	0.5	0.15	0.15	0.15	0.00	0.50	0.50	0.021	-0.020	-0.001
1000	4	1	0.8	0.8	0.5	0.15	0.15	0.15	0.25	0.00	0.00	0.008	0.000	0.006
1000	4	1	0.8	0.8	0.5	0.15	0.15	0.15	0.25	0.00	0.25	0.008	-0.001	0.005
1000	4	1	0.8	0.8	0.5	0.15	0.15	0.15	0.25	0.00	0.50	0.008	-0.001	0.005
1000	4	1	0.8	0.8	0.5	0.15	0.15	0.15	0.25	0.25	0.00	0.014	-0.007	0.002

(continued)

												Mean Bias		
N	m_1	$\frac{m_2}{m_1}$	$E(C)$	$E(R)$	$E(U)$	σ_C	σ_R	σ_U	ρ_{CR}	ρ_{CU}	ρ_{RU}	c	r	u
1000	4	1	0.8	0.8	0.5	0.15	0.15	0.15	0.25	0.25	0.25	0.014	-0.007	0.003
1000	4	1	0.8	0.8	0.5	0.15	0.15	0.15	0.25	0.25	0.50	0.014	-0.007	0.002
1000	4	1	0.8	0.8	0.5	0.15	0.15	0.15	0.25	0.50	0.00	0.021	-0.013	-0.001
1000	4	1	0.8	0.8	0.5	0.15	0.15	0.15	0.25	0.50	0.25	0.021	-0.014	0.000
1000	4	1	0.8	0.8	0.5	0.15	0.15	0.15	0.25	0.50	0.50	0.021	-0.014	0.000
1000	4	1	0.8	0.8	0.5	0.15	0.15	0.15	0.50	0.00	0.00	0.008	0.006	0.005
1000	4	1	0.8	0.8	0.5	0.15	0.15	0.15	0.50	0.00	0.25	0.007	0.006	0.005
1000	4	1	0.8	0.8	0.5	0.15	0.15	0.15	0.50	0.00	0.50	0.007	0.006	0.006
1000	4	1	0.8	0.8	0.5	0.15	0.15	0.15	0.50	0.25	0.00	0.014	0.000	0.002
1000	4	1	0.8	0.8	0.5	0.15	0.15	0.15	0.50	0.25	0.25	0.014	0.000	0.002
1000	4	1	0.8	0.8	0.5	0.15	0.15	0.15	0.50	0.25	0.50	0.014	0.000	0.002
1000	4	1	0.8	0.8	0.5	0.15	0.15	0.15	0.50	0.50	0.00	0.021	-0.006	-0.001
1000	4	1	0.8	0.8	0.5	0.15	0.15	0.15	0.50	0.50	0.25	0.021	-0.007	0.000
1000	4	1	0.8	0.8	0.5	0.15	0.15	0.15	0.50	0.50	0.50	0.020	-0.007	-0.001
1000	4	1	0.8	0.8	0.5	0.30	0.30	0.30	0.00	0.00	0.00	0.029	-0.028	0.022
1000	4	1	0.8	0.8	0.5	0.30	0.30	0.30	0.00	0.00	0.25	0.028	-0.028	0.021
1000	4	1	0.8	0.8	0.5	0.30	0.30	0.30	0.00	0.00	0.50	0.029	-0.027	0.021
1000	4	1	0.8	0.8	0.5	0.30	0.30	0.30	0.00	0.25	0.00	0.051	-0.048	0.011
1000	4	1	0.8	0.8	0.5	0.30	0.30	0.30	0.00	0.25	0.25	0.052	-0.048	0.012
1000	4	1	0.8	0.8	0.5	0.30	0.30	0.30	0.00	0.25	0.50	0.051	-0.048	0.012
1000	4	1	0.8	0.8	0.5	0.30	0.30	0.30	0.00	0.50	0.00	0.076	-0.070	0.002
1000	4	1	0.8	0.8	0.5	0.30	0.30	0.30	0.00	0.50	0.25	0.077	-0.070	0.003
1000	4	1	0.8	0.8	0.5	0.30	0.30	0.30	0.00	0.50	0.50	0.077	-0.070	0.003
1000	4	1	0.8	0.8	0.5	0.30	0.30	0.30	0.25	0.00	0.00	0.028	-0.007	0.021
1000	4	1	0.8	0.8	0.5	0.30	0.30	0.30	0.25	0.00	0.25	0.029	-0.007	0.021
1000	4	1	0.8	0.8	0.5	0.30	0.30	0.30	0.25	0.00	0.50	0.029	-0.007	0.022
1000	4	1	0.8	0.8	0.5	0.30	0.30	0.30	0.25	0.25	0.00	0.051	-0.028	0.012
1000	4	1	0.8	0.8	0.5	0.30	0.30	0.30	0.25	0.25	0.25	0.052	-0.029	0.012
1000	4	1	0.8	0.8	0.5	0.30	0.30	0.30	0.25	0.25	0.50	0.051	-0.028	0.011
1000	4	1	0.8	0.8	0.5	0.30	0.30	0.30	0.25	0.50	0.00	0.077	-0.051	0.003
1000	4	1	0.8	0.8	0.5	0.30	0.30	0.30	0.25	0.50	0.25	0.077	-0.051	0.002
1000	4	1	0.8	0.8	0.5	0.30	0.30	0.30	0.25	0.50	0.50	0.077	-0.051	0.002
1000	4	1	0.8	0.8	0.5	0.30	0.30	0.30	0.50	0.00	0.00	0.029	0.016	0.022
1000	4	1	0.8	0.8	0.5	0.30	0.30	0.30	0.50	0.00	0.25	0.028	0.017	0.022
1000	4	1	0.8	0.8	0.5	0.30	0.30	0.30	0.50	0.00	0.50	0.029	0.016	0.021
1000	4	1	0.8	0.8	0.5	0.30	0.30	0.30	0.50	0.25	0.00	0.052	-0.005	0.012
1000	4	1	0.8	0.8	0.5	0.30	0.30	0.30	0.50	0.25	0.25	0.051	-0.005	0.011
1000	4	1	0.8	0.8	0.5	0.30	0.30	0.30	0.50	0.25	0.50	0.052	-0.006	0.011
1000	4	1	0.8	0.8	0.5	0.30	0.30	0.30	0.50	0.50	0.00	0.076	-0.029	0.003

(continued)

												Mean Bias		
N	m_1	$\frac{m_2}{m_1}$	$E(C)$	$E(R)$	$E(U)$	σ_C	σ_R	σ_U	ρ_{CR}	ρ_{CU}	ρ_{RU}	c	r	u
1000	4	1	0.8	0.8	0.5	0.30	0.30	0.30	0.50	0.50	0.25	0.076	-0.028	0.003
1000	4	1	0.8	0.8	0.5	0.30	0.30	0.30	0.50	0.50	0.50	0.077	-0.028	0.003
1000	8	0	0.2	0.2	0.5	0.00	0.00	0.00	0.00	0.00	0.00	0.000	0.002	0.000
1000	8	0	0.2	0.2	0.5	0.15	0.15	0.15	0.00	0.00	0.00	0.065	-0.049	0.045
1000	8	0	0.2	0.2	0.5	0.15	0.15	0.15	0.00	0.00	0.25	0.066	-0.049	0.045
1000	8	0	0.2	0.2	0.5	0.15	0.15	0.15	0.00	0.00	0.50	0.066	-0.050	0.045
1000	8	0	0.2	0.2	0.5	0.15	0.15	0.15	0.00	0.25	0.00	0.067	-0.050	0.038
1000	8	0	0.2	0.2	0.5	0.15	0.15	0.15	0.00	0.25	0.25	0.067	-0.050	0.039
1000	8	0	0.2	0.2	0.5	0.15	0.15	0.15	0.00	0.25	0.50	0.067	-0.051	0.039
1000	8	0	0.2	0.2	0.5	0.15	0.15	0.15	0.00	0.50	0.00	0.067	-0.051	0.032
1000	8	0	0.2	0.2	0.5	0.15	0.15	0.15	0.00	0.50	0.25	0.067	-0.050	0.031
1000	8	0	0.2	0.2	0.5	0.15	0.15	0.15	0.00	0.50	0.50	0.067	-0.050	0.032
1000	8	0	0.2	0.2	0.5	0.15	0.15	0.15	0.25	0.00	0.00	0.066	-0.029	0.044
1000	8	0	0.2	0.2	0.5	0.15	0.15	0.15	0.25	0.00	0.25	0.066	-0.030	0.045
1000	8	0	0.2	0.2	0.5	0.15	0.15	0.15	0.25	0.00	0.50	0.065	-0.029	0.044
1000	8	0	0.2	0.2	0.5	0.15	0.15	0.15	0.25	0.25	0.00	0.067	-0.030	0.039
1000	8	0	0.2	0.2	0.5	0.15	0.15	0.15	0.25	0.25	0.25	0.067	-0.030	0.039
1000	8	0	0.2	0.2	0.5	0.15	0.15	0.15	0.25	0.25	0.50	0.068	-0.031	0.039
1000	8	0	0.2	0.2	0.5	0.15	0.15	0.15	0.25	0.50	0.00	0.067	-0.031	0.031
1000	8	0	0.2	0.2	0.5	0.15	0.15	0.15	0.25	0.50	0.25	0.068	-0.031	0.032
1000	8	0	0.2	0.2	0.5	0.15	0.15	0.15	0.25	0.50	0.50	0.067	-0.030	0.031
1000	8	0	0.2	0.2	0.5	0.15	0.15	0.15	0.50	0.00	0.00	0.066	-0.009	0.045
1000	8	0	0.2	0.2	0.5	0.15	0.15	0.15	0.50	0.00	0.25	0.066	-0.009	0.045
1000	8	0	0.2	0.2	0.5	0.15	0.15	0.15	0.50	0.00	0.50	0.066	-0.009	0.045
1000	8	0	0.2	0.2	0.5	0.15	0.15	0.15	0.50	0.25	0.00	0.067	-0.011	0.039
1000	8	0	0.2	0.2	0.5	0.15	0.15	0.15	0.50	0.25	0.25	0.067	-0.010	0.039
1000	8	0	0.2	0.2	0.5	0.15	0.15	0.15	0.50	0.25	0.50	0.067	-0.010	0.038
1000	8	0	0.2	0.2	0.5	0.15	0.15	0.15	0.50	0.50	0.00	0.068	-0.010	0.032
1000	8	0	0.2	0.2	0.5	0.15	0.15	0.15	0.50	0.50	0.25	0.068	-0.010	0.032
1000	8	0	0.2	0.2	0.5	0.15	0.15	0.15	0.50	0.50	0.50	0.067	-0.010	0.031
1000	8	0	0.2	0.2	0.5	0.30	0.30	0.30	0.00	0.00	0.00	0.212	-0.103	0.180
1000	8	0	0.2	0.2	0.5	0.30	0.30	0.30	0.00	0.00	0.25	0.212	-0.103	0.180
1000	8	0	0.2	0.2	0.5	0.30	0.30	0.30	0.00	0.00	0.50	0.212	-0.103	0.180
1000	8	0	0.2	0.2	0.5	0.30	0.30	0.30	0.00	0.25	0.00	0.225	-0.106	0.163
1000	8	0	0.2	0.2	0.5	0.30	0.30	0.30	0.00	0.25	0.25	0.224	-0.106	0.163
1000	8	0	0.2	0.2	0.5	0.30	0.30	0.30	0.00	0.25	0.50	0.224	-0.105	0.163
1000	8	0	0.2	0.2	0.5	0.30	0.30	0.30	0.00	0.50	0.00	0.233	-0.107	0.141
1000	8	0	0.2	0.2	0.5	0.30	0.30	0.30	0.00	0.50	0.25	0.233	-0.108	0.141
1000	8	0	0.2	0.2	0.5	0.30	0.30	0.30	0.00	0.50	0.50	0.233	-0.108	0.141

(continued)

N	m_1	$\frac{m_2}{m_1}$	$E(C)$	$E(R)$	$E(U)$	σ_C	σ_R	σ_U	ρ_{CR}	ρ_{CU}	ρ_{RU}	Mean Bias c	Mean Bias r	Mean Bias u
1000	8	0	0.2	0.2	0.5	0.30	0.30	0.30	0.25	0.00	0.00	0.212	-0.062	0.180
1000	8	0	0.2	0.2	0.5	0.30	0.30	0.30	0.25	0.00	0.25	0.211	-0.062	0.180
1000	8	0	0.2	0.2	0.5	0.30	0.30	0.30	0.25	0.00	0.50	0.212	-0.062	0.179
1000	8	0	0.2	0.2	0.5	0.30	0.30	0.30	0.25	0.25	0.00	0.224	-0.066	0.163
1000	8	0	0.2	0.2	0.5	0.30	0.30	0.30	0.25	0.25	0.25	0.224	-0.066	0.163
1000	8	0	0.2	0.2	0.5	0.30	0.30	0.30	0.25	0.25	0.50	0.224	-0.066	0.163
1000	8	0	0.2	0.2	0.5	0.30	0.30	0.30	0.25	0.50	0.00	0.234	-0.069	0.141
1000	8	0	0.2	0.2	0.5	0.30	0.30	0.30	0.25	0.50	0.25	0.233	-0.069	0.140
1000	8	0	0.2	0.2	0.5	0.30	0.30	0.30	0.25	0.50	0.50	0.233	-0.069	0.140
1000	8	0	0.2	0.2	0.5	0.30	0.30	0.30	0.50	0.00	0.00	0.211	-0.014	0.180
1000	8	0	0.2	0.2	0.5	0.30	0.30	0.30	0.50	0.00	0.25	0.211	-0.014	0.179
1000	8	0	0.2	0.2	0.5	0.30	0.30	0.30	0.50	0.00	0.50	0.212	-0.014	0.180
1000	8	0	0.2	0.2	0.5	0.30	0.30	0.30	0.50	0.25	0.00	0.225	-0.021	0.163
1000	8	0	0.2	0.2	0.5	0.30	0.30	0.30	0.50	0.25	0.25	0.225	-0.020	0.163
1000	8	0	0.2	0.2	0.5	0.30	0.30	0.30	0.50	0.25	0.50	0.224	-0.020	0.163
1000	8	0	0.2	0.2	0.5	0.30	0.30	0.30	0.50	0.50	0.00	0.233	-0.024	0.141
1000	8	0	0.2	0.2	0.5	0.30	0.30	0.30	0.50	0.50	0.25	0.233	-0.023	0.141
1000	8	0	0.2	0.2	0.5	0.30	0.30	0.30	0.50	0.50	0.50	0.234	-0.023	0.141
1000	8	0	0.2	0.5	0.5	0.00	0.00	0.00	0.00	0.00	0.00	0.000	0.002	0.000
1000	8	0	0.2	0.5	0.5	0.15	0.15	0.15	0.00	0.00	0.00	0.066	-0.123	0.045
1000	8	0	0.2	0.5	0.5	0.15	0.15	0.15	0.00	0.00	0.25	0.066	-0.123	0.044
1000	8	0	0.2	0.5	0.5	0.15	0.15	0.15	0.00	0.00	0.50	0.066	-0.125	0.045
1000	8	0	0.2	0.5	0.5	0.15	0.15	0.15	0.00	0.25	0.00	0.067	-0.124	0.038
1000	8	0	0.2	0.5	0.5	0.15	0.15	0.15	0.00	0.25	0.25	0.067	-0.126	0.039
1000	8	0	0.2	0.5	0.5	0.15	0.15	0.15	0.00	0.25	0.50	0.067	-0.126	0.039
1000	8	0	0.2	0.5	0.5	0.15	0.15	0.15	0.00	0.50	0.00	0.068	-0.127	0.031
1000	8	0	0.2	0.5	0.5	0.15	0.15	0.15	0.00	0.50	0.25	0.068	-0.126	0.032
1000	8	0	0.2	0.5	0.5	0.15	0.15	0.15	0.00	0.50	0.50	0.068	-0.127	0.032
1000	8	0	0.2	0.5	0.5	0.15	0.15	0.15	0.25	0.00	0.00	0.066	-0.103	0.045
1000	8	0	0.2	0.5	0.5	0.15	0.15	0.15	0.25	0.00	0.25	0.066	-0.104	0.045
1000	8	0	0.2	0.5	0.5	0.15	0.15	0.15	0.25	0.00	0.50	0.066	-0.104	0.046
1000	8	0	0.2	0.5	0.5	0.15	0.15	0.15	0.25	0.25	0.00	0.067	-0.105	0.038
1000	8	0	0.2	0.5	0.5	0.15	0.15	0.15	0.25	0.25	0.25	0.067	-0.105	0.038
1000	8	0	0.2	0.5	0.5	0.15	0.15	0.15	0.25	0.25	0.50	0.067	-0.105	0.038
1000	8	0	0.2	0.5	0.5	0.15	0.15	0.15	0.25	0.50	0.00	0.068	-0.106	0.031
1000	8	0	0.2	0.5	0.5	0.15	0.15	0.15	0.25	0.50	0.25	0.068	-0.107	0.032
1000	8	0	0.2	0.5	0.5	0.15	0.15	0.15	0.25	0.50	0.50	0.068	-0.107	0.032
1000	8	0	0.2	0.5	0.5	0.15	0.15	0.15	0.50	0.00	0.00	0.066	-0.083	0.045
1000	8	0	0.2	0.5	0.5	0.15	0.15	0.15	0.50	0.00	0.25	0.066	-0.084	0.045

(continued)

												Mean Bias		
N	m_1	$\frac{m_2}{m_1}$	$E(C)$	$E(R)$	$E(U)$	σ_C	σ_R	σ_U	ρ_{CR}	ρ_{CU}	ρ_{RU}	c	r	u
1000	8	0	0.2	0.5	0.5	0.15	0.15	0.15	0.50	0.00	0.50	0.065	-0.082	0.044
1000	8	0	0.2	0.5	0.5	0.15	0.15	0.15	0.50	0.25	0.00	0.067	-0.085	0.038
1000	8	0	0.2	0.5	0.5	0.15	0.15	0.15	0.50	0.25	0.25	0.067	-0.085	0.039
1000	8	0	0.2	0.5	0.5	0.15	0.15	0.15	0.50	0.25	0.50	0.067	-0.086	0.039
1000	8	0	0.2	0.5	0.5	0.15	0.15	0.15	0.50	0.50	0.00	0.067	-0.086	0.031
1000	8	0	0.2	0.5	0.5	0.15	0.15	0.15	0.50	0.50	0.25	0.067	-0.086	0.031
1000	8	0	0.2	0.5	0.5	0.15	0.15	0.15	0.50	0.50	0.50	0.068	-0.087	0.032
1000	8	0	0.2	0.5	0.5	0.30	0.30	0.30	0.00	0.00	0.00	0.212	-0.257	0.180
1000	8	0	0.2	0.5	0.5	0.30	0.30	0.30	0.00	0.00	0.25	0.212	-0.257	0.179
1000	8	0	0.2	0.5	0.5	0.30	0.30	0.30	0.00	0.00	0.50	0.212	-0.257	0.180
1000	8	0	0.2	0.5	0.5	0.30	0.30	0.30	0.00	0.25	0.00	0.224	-0.264	0.163
1000	8	0	0.2	0.5	0.5	0.30	0.30	0.30	0.00	0.25	0.25	0.224	-0.264	0.163
1000	8	0	0.2	0.5	0.5	0.30	0.30	0.30	0.00	0.25	0.50	0.224	-0.265	0.163
1000	8	0	0.2	0.5	0.5	0.30	0.30	0.30	0.00	0.50	0.00	0.233	-0.269	0.140
1000	8	0	0.2	0.5	0.5	0.30	0.30	0.30	0.00	0.50	0.25	0.233	-0.269	0.141
1000	8	0	0.2	0.5	0.5	0.30	0.30	0.30	0.00	0.50	0.50	0.233	-0.269	0.140
1000	8	0	0.2	0.5	0.5	0.30	0.30	0.30	0.25	0.00	0.00	0.212	-0.213	0.179
1000	8	0	0.2	0.5	0.5	0.30	0.30	0.30	0.25	0.00	0.25	0.212	-0.212	0.180
1000	8	0	0.2	0.5	0.5	0.30	0.30	0.30	0.25	0.00	0.50	0.211	-0.213	0.180
1000	8	0	0.2	0.5	0.5	0.30	0.30	0.30	0.25	0.25	0.00	0.224	-0.221	0.163
1000	8	0	0.2	0.5	0.5	0.30	0.30	0.30	0.25	0.25	0.25	0.225	-0.222	0.163
1000	8	0	0.2	0.5	0.5	0.30	0.30	0.30	0.25	0.25	0.50	0.224	-0.221	0.163
1000	8	0	0.2	0.5	0.5	0.30	0.30	0.30	0.25	0.50	0.00	0.233	-0.227	0.141
1000	8	0	0.2	0.5	0.5	0.30	0.30	0.30	0.25	0.50	0.25	0.233	-0.227	0.140
1000	8	0	0.2	0.5	0.5	0.30	0.30	0.30	0.25	0.50	0.50	0.233	-0.227	0.140
1000	8	0	0.2	0.5	0.5	0.30	0.30	0.30	0.50	0.00	0.00	0.212	-0.169	0.180
1000	8	0	0.2	0.5	0.5	0.30	0.30	0.30	0.50	0.00	0.25	0.212	-0.169	0.180
1000	8	0	0.2	0.5	0.5	0.30	0.30	0.30	0.50	0.00	0.50	0.212	-0.168	0.180
1000	8	0	0.2	0.5	0.5	0.30	0.30	0.30	0.50	0.25	0.00	0.224	-0.178	0.163
1000	8	0	0.2	0.5	0.5	0.30	0.30	0.30	0.50	0.25	0.25	0.224	-0.178	0.163
1000	8	0	0.2	0.5	0.5	0.30	0.30	0.30	0.50	0.25	0.50	0.224	-0.177	0.163
1000	8	0	0.2	0.5	0.5	0.30	0.30	0.30	0.50	0.50	0.00	0.233	-0.183	0.141
1000	8	0	0.2	0.5	0.5	0.30	0.30	0.30	0.50	0.50	0.25	0.233	-0.185	0.140
1000	8	0	0.2	0.5	0.5	0.30	0.30	0.30	0.50	0.50	0.50	0.232	-0.185	0.141
1000	8	0	0.2	0.8	0.5	0.00	0.00	0.00	0.00	0.00	0.00	0.000	0.003	0.000
1000	8	0	0.2	0.8	0.5	0.15	0.15	0.15	0.00	0.00	0.00	0.066	-0.198	0.045
1000	8	0	0.2	0.8	0.5	0.15	0.15	0.15	0.00	0.00	0.25	0.066	-0.198	0.045
1000	8	0	0.2	0.8	0.5	0.15	0.15	0.15	0.00	0.00	0.50	0.065	-0.196	0.044
1000	8	0	0.2	0.8	0.5	0.15	0.15	0.15	0.00	0.25	0.00	0.067	-0.201	0.039

(continued)

												Mean Bias		
N	m_1	$\frac{m_2}{m_1}$	$E(C)$	$E(R)$	$E(U)$	σ_C	σ_R	σ_U	ρ_{CR}	ρ_{CU}	ρ_{RU}	c	r	u
1000	8	0	0.2	0.8	0.5	0.15	0.15	0.15	0.00	0.25	0.25	0.067	-0.200	0.039
1000	8	0	0.2	0.8	0.5	0.15	0.15	0.15	0.00	0.25	0.50	0.067	-0.201	0.038
1000	8	0	0.2	0.8	0.5	0.15	0.15	0.15	0.00	0.50	0.00	0.068	-0.202	0.032
1000	8	0	0.2	0.8	0.5	0.15	0.15	0.15	0.00	0.50	0.25	0.068	-0.202	0.032
1000	8	0	0.2	0.8	0.5	0.15	0.15	0.15	0.00	0.50	0.50	0.067	-0.201	0.032
1000	8	0	0.2	0.8	0.5	0.15	0.15	0.15	0.25	0.00	0.00	0.066	-0.179	0.045
1000	8	0	0.2	0.8	0.5	0.15	0.15	0.15	0.25	0.00	0.25	0.066	-0.178	0.045
1000	8	0	0.2	0.8	0.5	0.15	0.15	0.15	0.25	0.00	0.50	0.065	-0.178	0.045
1000	8	0	0.2	0.8	0.5	0.15	0.15	0.15	0.25	0.25	0.00	0.067	-0.181	0.038
1000	8	0	0.2	0.8	0.5	0.15	0.15	0.15	0.25	0.25	0.25	0.067	-0.181	0.038
1000	8	0	0.2	0.8	0.5	0.15	0.15	0.15	0.25	0.25	0.50	0.067	-0.182	0.039
1000	8	0	0.2	0.8	0.5	0.15	0.15	0.15	0.25	0.50	0.00	0.068	-0.184	0.032
1000	8	0	0.2	0.8	0.5	0.15	0.15	0.15	0.25	0.50	0.25	0.068	-0.183	0.032
1000	8	0	0.2	0.8	0.5	0.15	0.15	0.15	0.25	0.50	0.50	0.068	-0.183	0.031
1000	8	0	0.2	0.8	0.5	0.15	0.15	0.15	0.50	0.00	0.00	0.065	-0.161	0.045
1000	8	0	0.2	0.8	0.5	0.15	0.15	0.15	0.50	0.00	0.25	0.065	-0.161	0.045
1000	8	0	0.2	0.8	0.5	0.15	0.15	0.15	0.50	0.00	0.50	0.066	-0.160	0.045
1000	8	0	0.2	0.8	0.5	0.15	0.15	0.15	0.50	0.25	0.00	0.067	-0.164	0.039
1000	8	0	0.2	0.8	0.5	0.15	0.15	0.15	0.50	0.25	0.25	0.068	-0.164	0.039
1000	8	0	0.2	0.8	0.5	0.15	0.15	0.15	0.50	0.25	0.50	0.067	-0.164	0.038
1000	8	0	0.2	0.8	0.5	0.15	0.15	0.15	0.50	0.50	0.00	0.067	-0.165	0.032
1000	8	0	0.2	0.8	0.5	0.15	0.15	0.15	0.50	0.50	0.25	0.068	-0.165	0.032
1000	8	0	0.2	0.8	0.5	0.15	0.15	0.15	0.50	0.50	0.50	0.067	-0.165	0.032
1000	8	0	0.2	0.8	0.5	0.30	0.30	0.30	0.00	0.00	0.00	0.211	-0.411	0.179
1000	8	0	0.2	0.8	0.5	0.30	0.30	0.30	0.00	0.00	0.25	0.211	-0.412	0.180
1000	8	0	0.2	0.8	0.5	0.30	0.30	0.30	0.00	0.00	0.50	0.211	-0.411	0.180
1000	8	0	0.2	0.8	0.5	0.30	0.30	0.30	0.00	0.25	0.00	0.224	-0.423	0.163
1000	8	0	0.2	0.8	0.5	0.30	0.30	0.30	0.00	0.25	0.25	0.225	-0.423	0.163
1000	8	0	0.2	0.8	0.5	0.30	0.30	0.30	0.00	0.25	0.50	0.225	-0.421	0.163
1000	8	0	0.2	0.8	0.5	0.30	0.30	0.30	0.00	0.50	0.00	0.234	-0.431	0.140
1000	8	0	0.2	0.8	0.5	0.30	0.30	0.30	0.00	0.50	0.25	0.233	-0.430	0.141
1000	8	0	0.2	0.8	0.5	0.30	0.30	0.30	0.00	0.50	0.50	0.233	-0.431	0.141
1000	8	0	0.2	0.8	0.5	0.30	0.30	0.30	0.25	0.00	0.00	0.212	-0.375	0.180
1000	8	0	0.2	0.8	0.5	0.30	0.30	0.30	0.25	0.00	0.25	0.212	-0.376	0.179
1000	8	0	0.2	0.8	0.5	0.30	0.30	0.30	0.25	0.00	0.50	0.211	-0.376	0.180
1000	8	0	0.2	0.8	0.5	0.30	0.30	0.30	0.25	0.25	0.00	0.224	-0.389	0.164
1000	8	0	0.2	0.8	0.5	0.30	0.30	0.30	0.25	0.25	0.25	0.224	-0.388	0.162
1000	8	0	0.2	0.8	0.5	0.30	0.30	0.30	0.25	0.25	0.50	0.225	-0.389	0.163
1000	8	0	0.2	0.8	0.5	0.30	0.30	0.30	0.25	0.50	0.00	0.233	-0.397	0.140

(continued)

N	m_1	$\frac{m_2}{m_1}$	$E(C)$	$E(R)$	$E(U)$	σ_C	σ_R	σ_U	ρ_{CR}	ρ_{CU}	ρ_{RU}	Mean Bias		
												c	r	u
1000	8	0	0.2	0.8	0.5	0.30	0.30	0.30	0.25	0.50	0.25	0.233	-0.397	0.141
1000	8	0	0.2	0.8	0.5	0.30	0.30	0.30	0.25	0.50	0.50	0.233	-0.398	0.141
1000	8	0	0.2	0.8	0.5	0.30	0.30	0.30	0.50	0.00	0.00	0.211	-0.347	0.180
1000	8	0	0.2	0.8	0.5	0.30	0.30	0.30	0.50	0.00	0.25	0.211	-0.346	0.180
1000	8	0	0.2	0.8	0.5	0.30	0.30	0.30	0.50	0.00	0.50	0.212	-0.347	0.180
1000	8	0	0.2	0.8	0.5	0.30	0.30	0.30	0.50	0.25	0.00	0.225	-0.360	0.163
1000	8	0	0.2	0.8	0.5	0.30	0.30	0.30	0.50	0.25	0.25	0.224	-0.360	0.163
1000	8	0	0.2	0.8	0.5	0.30	0.30	0.30	0.50	0.25	0.50	0.224	-0.360	0.163
1000	8	0	0.2	0.8	0.5	0.30	0.30	0.30	0.50	0.50	0.00	0.232	-0.369	0.140
1000	8	0	0.2	0.8	0.5	0.30	0.30	0.30	0.50	0.50	0.25	0.233	-0.369	0.141
1000	8	0	0.2	0.8	0.5	0.30	0.30	0.30	0.50	0.50	0.50	0.233	-0.369	0.141
1000	8	0	0.5	0.2	0.5	0.00	0.00	0.00	0.00	0.00	0.00	-0.001	0.001	-0.001
1000	8	0	0.5	0.2	0.5	0.15	0.15	0.15	0.00	0.00	0.00	0.041	-0.015	0.045
1000	8	0	0.5	0.2	0.5	0.15	0.15	0.15	0.00	0.00	0.25	0.041	-0.015	0.045
1000	8	0	0.5	0.2	0.5	0.15	0.15	0.15	0.00	0.00	0.50	0.041	-0.015	0.045
1000	8	0	0.5	0.2	0.5	0.15	0.15	0.15	0.00	0.25	0.00	0.042	-0.016	0.034
1000	8	0	0.5	0.2	0.5	0.15	0.15	0.15	0.00	0.25	0.25	0.042	-0.015	0.034
1000	8	0	0.5	0.2	0.5	0.15	0.15	0.15	0.00	0.25	0.50	0.043	-0.016	0.034
1000	8	0	0.5	0.2	0.5	0.15	0.15	0.15	0.00	0.50	0.00	0.044	-0.016	0.024
1000	8	0	0.5	0.2	0.5	0.15	0.15	0.15	0.00	0.50	0.25	0.044	-0.016	0.024
1000	8	0	0.5	0.2	0.5	0.15	0.15	0.15	0.00	0.50	0.50	0.044	-0.016	0.024
1000	8	0	0.5	0.2	0.5	0.15	0.15	0.15	0.25	0.00	0.00	0.041	-0.005	0.045
1000	8	0	0.5	0.2	0.5	0.15	0.15	0.15	0.25	0.00	0.25	0.041	-0.005	0.044
1000	8	0	0.5	0.2	0.5	0.15	0.15	0.15	0.25	0.00	0.50	0.042	-0.005	0.045
1000	8	0	0.5	0.2	0.5	0.15	0.15	0.15	0.25	0.25	0.00	0.043	-0.006	0.035
1000	8	0	0.5	0.2	0.5	0.15	0.15	0.15	0.25	0.25	0.25	0.043	-0.006	0.034
1000	8	0	0.5	0.2	0.5	0.15	0.15	0.15	0.25	0.25	0.50	0.042	-0.006	0.034
1000	8	0	0.5	0.2	0.5	0.15	0.15	0.15	0.25	0.50	0.00	0.043	-0.006	0.023
1000	8	0	0.5	0.2	0.5	0.15	0.15	0.15	0.25	0.50	0.25	0.043	-0.006	0.023
1000	8	0	0.5	0.2	0.5	0.15	0.15	0.15	0.25	0.50	0.50	0.044	-0.006	0.024
1000	8	0	0.5	0.2	0.5	0.15	0.15	0.15	0.50	0.00	0.00	0.041	0.005	0.044
1000	8	0	0.5	0.2	0.5	0.15	0.15	0.15	0.50	0.00	0.25	0.041	0.005	0.045
1000	8	0	0.5	0.2	0.5	0.15	0.15	0.15	0.50	0.00	0.50	0.041	0.005	0.045
1000	8	0	0.5	0.2	0.5	0.15	0.15	0.15	0.50	0.25	0.00	0.042	0.004	0.034
1000	8	0	0.5	0.2	0.5	0.15	0.15	0.15	0.50	0.25	0.25	0.043	0.004	0.035
1000	8	0	0.5	0.2	0.5	0.15	0.15	0.15	0.50	0.25	0.50	0.043	0.004	0.035
1000	8	0	0.5	0.2	0.5	0.15	0.15	0.15	0.50	0.50	0.00	0.043	0.004	0.023
1000	8	0	0.5	0.2	0.5	0.15	0.15	0.15	0.50	0.50	0.25	0.044	0.004	0.024
1000	8	0	0.5	0.2	0.5	0.15	0.15	0.15	0.50	0.50	0.50	0.045	0.004	0.024

(continued)

N	m_1	$\frac{m_2}{m_1}$	$E(C)$	$E(R)$	$E(U)$	σ_C	σ_R	σ_U	ρ_{CR}	ρ_{CU}	ρ_{RU}	Mean Bias c	r	u
1000	8	0	0.5	0.2	0.5	0.30	0.30	0.30	0.00	0.00	0.00	0.132	-0.042	0.179
1000	8	0	0.5	0.2	0.5	0.30	0.30	0.30	0.00	0.00	0.25	0.132	-0.042	0.179
1000	8	0	0.5	0.2	0.5	0.30	0.30	0.30	0.00	0.00	0.50	0.132	-0.042	0.180
1000	8	0	0.5	0.2	0.5	0.30	0.30	0.30	0.00	0.25	0.00	0.149	-0.046	0.150
1000	8	0	0.5	0.2	0.5	0.30	0.30	0.30	0.00	0.25	0.25	0.149	-0.046	0.150
1000	8	0	0.5	0.2	0.5	0.30	0.30	0.30	0.00	0.25	0.50	0.148	-0.046	0.150
1000	8	0	0.5	0.2	0.5	0.30	0.30	0.30	0.00	0.50	0.00	0.162	-0.049	0.113
1000	8	0	0.5	0.2	0.5	0.30	0.30	0.30	0.00	0.50	0.25	0.162	-0.049	0.112
1000	8	0	0.5	0.2	0.5	0.30	0.30	0.30	0.00	0.50	0.50	0.162	-0.048	0.113
1000	8	0	0.5	0.2	0.5	0.30	0.30	0.30	0.25	0.00	0.00	0.132	-0.013	0.179
1000	8	0	0.5	0.2	0.5	0.30	0.30	0.30	0.25	0.00	0.25	0.132	-0.013	0.180
1000	8	0	0.5	0.2	0.5	0.30	0.30	0.30	0.25	0.00	0.50	0.132	-0.012	0.179
1000	8	0	0.5	0.2	0.5	0.30	0.30	0.30	0.25	0.25	0.00	0.148	-0.018	0.150
1000	8	0	0.5	0.2	0.5	0.30	0.30	0.30	0.25	0.25	0.25	0.148	-0.017	0.150
1000	8	0	0.5	0.2	0.5	0.30	0.30	0.30	0.25	0.25	0.50	0.148	-0.017	0.150
1000	8	0	0.5	0.2	0.5	0.30	0.30	0.30	0.25	0.50	0.00	0.162	-0.022	0.114
1000	8	0	0.5	0.2	0.5	0.30	0.30	0.30	0.25	0.50	0.25	0.162	-0.021	0.113
1000	8	0	0.5	0.2	0.5	0.30	0.30	0.30	0.25	0.50	0.50	0.162	-0.021	0.113
1000	8	0	0.5	0.2	0.5	0.30	0.30	0.30	0.50	0.00	0.00	0.132	0.017	0.180
1000	8	0	0.5	0.2	0.5	0.30	0.30	0.30	0.50	0.00	0.25	0.132	0.016	0.179
1000	8	0	0.5	0.2	0.5	0.30	0.30	0.30	0.50	0.00	0.50	0.132	0.017	0.179
1000	8	0	0.5	0.2	0.5	0.30	0.30	0.30	0.50	0.25	0.00	0.148	0.011	0.149
1000	8	0	0.5	0.2	0.5	0.30	0.30	0.30	0.50	0.25	0.25	0.149	0.011	0.150
1000	8	0	0.5	0.2	0.5	0.30	0.30	0.30	0.50	0.25	0.50	0.148	0.012	0.150
1000	8	0	0.5	0.2	0.5	0.30	0.30	0.30	0.50	0.50	0.00	0.162	0.007	0.113
1000	8	0	0.5	0.2	0.5	0.30	0.30	0.30	0.50	0.50	0.25	0.163	0.007	0.113
1000	8	0	0.5	0.2	0.5	0.30	0.30	0.30	0.50	0.50	0.50	0.162	0.007	0.112
1000	8	0	0.5	0.5	0.5	0.00	0.00	0.00	0.00	0.00	0.00	0.000	0.000	0.000
1000	8	0	0.5	0.5	0.5	0.15	0.15	0.15	0.00	0.00	0.00	0.041	-0.038	0.045
1000	8	0	0.5	0.5	0.5	0.15	0.15	0.15	0.00	0.00	0.25	0.041	-0.038	0.045
1000	8	0	0.5	0.5	0.5	0.15	0.15	0.15	0.00	0.00	0.50	0.041	-0.038	0.045
1000	8	0	0.5	0.5	0.5	0.15	0.15	0.15	0.00	0.25	0.00	0.043	-0.039	0.034
1000	8	0	0.5	0.5	0.5	0.15	0.15	0.15	0.00	0.25	0.25	0.042	-0.039	0.034
1000	8	0	0.5	0.5	0.5	0.15	0.15	0.15	0.00	0.25	0.50	0.042	-0.040	0.034
1000	8	0	0.5	0.5	0.5	0.15	0.15	0.15	0.00	0.50	0.00	0.044	-0.040	0.023
1000	8	0	0.5	0.5	0.5	0.15	0.15	0.15	0.00	0.50	0.25	0.044	-0.040	0.022
1000	8	0	0.5	0.5	0.5	0.15	0.15	0.15	0.00	0.50	0.50	0.044	-0.041	0.023
1000	8	0	0.5	0.5	0.5	0.15	0.15	0.15	0.25	0.00	0.00	0.041	-0.028	0.045
1000	8	0	0.5	0.5	0.5	0.15	0.15	0.15	0.25	0.00	0.25	0.041	-0.027	0.045

(continued)

N	m_1	$\frac{m_2}{m_1}$	$E(C)$	$E(R)$	$E(U)$	σ_C	σ_R	σ_U	ρ_{CR}	ρ_{CU}	ρ_{RU}	Mean Bias c	Mean Bias r	Mean Bias u
1000	8	0	0.5	0.5	0.5	0.15	0.15	0.15	0.25	0.00	0.50	0.041	-0.027	0.045
1000	8	0	0.5	0.5	0.5	0.15	0.15	0.15	0.25	0.25	0.00	0.043	-0.029	0.035
1000	8	0	0.5	0.5	0.5	0.15	0.15	0.15	0.25	0.25	0.25	0.043	-0.029	0.034
1000	8	0	0.5	0.5	0.5	0.15	0.15	0.15	0.25	0.25	0.50	0.043	-0.029	0.035
1000	8	0	0.5	0.5	0.5	0.15	0.15	0.15	0.25	0.50	0.00	0.043	-0.030	0.023
1000	8	0	0.5	0.5	0.5	0.15	0.15	0.15	0.25	0.50	0.25	0.044	-0.030	0.023
1000	8	0	0.5	0.5	0.5	0.15	0.15	0.15	0.25	0.50	0.50	0.044	-0.030	0.023
1000	8	0	0.5	0.5	0.5	0.15	0.15	0.15	0.50	0.00	0.00	0.042	-0.017	0.045
1000	8	0	0.5	0.5	0.5	0.15	0.15	0.15	0.50	0.00	0.25	0.042	-0.017	0.045
1000	8	0	0.5	0.5	0.5	0.15	0.15	0.15	0.50	0.00	0.50	0.041	-0.018	0.045
1000	8	0	0.5	0.5	0.5	0.15	0.15	0.15	0.50	0.25	0.00	0.043	-0.019	0.035
1000	8	0	0.5	0.5	0.5	0.15	0.15	0.15	0.50	0.25	0.25	0.043	-0.019	0.034
1000	8	0	0.5	0.5	0.5	0.15	0.15	0.15	0.50	0.25	0.50	0.043	-0.018	0.034
1000	8	0	0.5	0.5	0.5	0.15	0.15	0.15	0.50	0.50	0.00	0.044	-0.019	0.023
1000	8	0	0.5	0.5	0.5	0.15	0.15	0.15	0.50	0.50	0.25	0.043	-0.020	0.024
1000	8	0	0.5	0.5	0.5	0.15	0.15	0.15	0.50	0.50	0.50	0.044	-0.019	0.024
1000	8	0	0.5	0.5	0.5	0.30	0.30	0.30	0.00	0.00	0.00	0.133	-0.104	0.179
1000	8	0	0.5	0.5	0.5	0.30	0.30	0.30	0.00	0.00	0.25	0.133	-0.105	0.180
1000	8	0	0.5	0.5	0.5	0.30	0.30	0.30	0.00	0.00	0.50	0.133	-0.105	0.180
1000	8	0	0.5	0.5	0.5	0.30	0.30	0.30	0.00	0.25	0.00	0.149	-0.114	0.150
1000	8	0	0.5	0.5	0.5	0.30	0.30	0.30	0.00	0.25	0.25	0.149	-0.114	0.150
1000	8	0	0.5	0.5	0.5	0.30	0.30	0.30	0.00	0.25	0.50	0.148	-0.114	0.150
1000	8	0	0.5	0.5	0.5	0.30	0.30	0.30	0.00	0.50	0.00	0.162	-0.123	0.113
1000	8	0	0.5	0.5	0.5	0.30	0.30	0.30	0.00	0.50	0.25	0.163	-0.122	0.113
1000	8	0	0.5	0.5	0.5	0.30	0.30	0.30	0.00	0.50	0.50	0.163	-0.123	0.113
1000	8	0	0.5	0.5	0.5	0.30	0.30	0.30	0.25	0.00	0.00	0.132	-0.071	0.179
1000	8	0	0.5	0.5	0.5	0.30	0.30	0.30	0.25	0.00	0.25	0.132	-0.071	0.180
1000	8	0	0.5	0.5	0.5	0.30	0.30	0.30	0.25	0.00	0.50	0.132	-0.071	0.180
1000	8	0	0.5	0.5	0.5	0.30	0.30	0.30	0.25	0.25	0.00	0.148	-0.082	0.149
1000	8	0	0.5	0.5	0.5	0.30	0.30	0.30	0.25	0.25	0.25	0.148	-0.081	0.150
1000	8	0	0.5	0.5	0.5	0.30	0.30	0.30	0.25	0.25	0.50	0.148	-0.081	0.150
1000	8	0	0.5	0.5	0.5	0.30	0.30	0.30	0.25	0.50	0.00	0.162	-0.090	0.113
1000	8	0	0.5	0.5	0.5	0.30	0.30	0.30	0.25	0.50	0.25	0.163	-0.090	0.113
1000	8	0	0.5	0.5	0.5	0.30	0.30	0.30	0.25	0.50	0.50	0.163	-0.091	0.113
1000	8	0	0.5	0.5	0.5	0.30	0.30	0.30	0.50	0.00	0.00	0.132	-0.036	0.179
1000	8	0	0.5	0.5	0.5	0.30	0.30	0.30	0.50	0.00	0.25	0.132	-0.037	0.180
1000	8	0	0.5	0.5	0.5	0.30	0.30	0.30	0.50	0.00	0.50	0.133	-0.036	0.181
1000	8	0	0.5	0.5	0.5	0.30	0.30	0.30	0.50	0.25	0.00	0.148	-0.048	0.149
1000	8	0	0.5	0.5	0.5	0.30	0.30	0.30	0.50	0.25	0.25	0.148	-0.048	0.150

(continued)

N	m_1	$\frac{m_2}{m_1}$	$E(C)$	$E(R)$	$E(U)$	σ_C	σ_R	σ_U	ρ_{CR}	ρ_{CU}	ρ_{RU}	Mean Bias c	r	u
1000	8	0	0.5	0.5	0.5	0.30	0.30	0.30	0.50	0.25	0.50	0.148	-0.048	0.149
1000	8	0	0.5	0.5	0.5	0.30	0.30	0.30	0.50	0.50	0.00	0.162	-0.057	0.113
1000	8	0	0.5	0.5	0.5	0.30	0.30	0.30	0.50	0.50	0.25	0.162	-0.058	0.113
1000	8	0	0.5	0.5	0.5	0.30	0.30	0.30	0.50	0.50	0.50	0.162	-0.058	0.113
1000	8	0	0.5	0.8	0.5	0.00	0.00	0.00	0.00	0.00	0.00	0.000	0.000	0.000
1000	8	0	0.5	0.8	0.5	0.15	0.15	0.15	0.00	0.00	0.00	0.041	-0.060	0.045
1000	8	0	0.5	0.8	0.5	0.15	0.15	0.15	0.00	0.00	0.25	0.041	-0.061	0.045
1000	8	0	0.5	0.8	0.5	0.15	0.15	0.15	0.00	0.00	0.50	0.041	-0.061	0.045
1000	8	0	0.5	0.8	0.5	0.15	0.15	0.15	0.00	0.25	0.00	0.042	-0.062	0.034
1000	8	0	0.5	0.8	0.5	0.15	0.15	0.15	0.00	0.25	0.25	0.043	-0.063	0.035
1000	8	0	0.5	0.8	0.5	0.15	0.15	0.15	0.00	0.25	0.50	0.042	-0.062	0.035
1000	8	0	0.5	0.8	0.5	0.15	0.15	0.15	0.00	0.50	0.00	0.044	-0.064	0.023
1000	8	0	0.5	0.8	0.5	0.15	0.15	0.15	0.00	0.50	0.25	0.043	-0.064	0.023
1000	8	0	0.5	0.8	0.5	0.15	0.15	0.15	0.00	0.50	0.50	0.044	-0.065	0.024
1000	8	0	0.5	0.8	0.5	0.15	0.15	0.15	0.25	0.00	0.00	0.041	-0.050	0.045
1000	8	0	0.5	0.8	0.5	0.15	0.15	0.15	0.25	0.00	0.25	0.041	-0.050	0.044
1000	8	0	0.5	0.8	0.5	0.15	0.15	0.15	0.25	0.00	0.50	0.041	-0.051	0.045
1000	8	0	0.5	0.8	0.5	0.15	0.15	0.15	0.25	0.25	0.00	0.042	-0.053	0.034
1000	8	0	0.5	0.8	0.5	0.15	0.15	0.15	0.25	0.25	0.25	0.042	-0.053	0.035
1000	8	0	0.5	0.8	0.5	0.15	0.15	0.15	0.25	0.25	0.50	0.042	-0.053	0.035
1000	8	0	0.5	0.8	0.5	0.15	0.15	0.15	0.25	0.50	0.00	0.044	-0.055	0.024
1000	8	0	0.5	0.8	0.5	0.15	0.15	0.15	0.25	0.50	0.25	0.044	-0.055	0.024
1000	8	0	0.5	0.8	0.5	0.15	0.15	0.15	0.25	0.50	0.50	0.043	-0.055	0.024
1000	8	0	0.5	0.8	0.5	0.15	0.15	0.15	0.50	0.00	0.00	0.041	-0.041	0.045
1000	8	0	0.5	0.8	0.5	0.15	0.15	0.15	0.50	0.00	0.25	0.041	-0.041	0.045
1000	8	0	0.5	0.8	0.5	0.15	0.15	0.15	0.50	0.00	0.50	0.042	-0.041	0.045
1000	8	0	0.5	0.8	0.5	0.15	0.15	0.15	0.50	0.25	0.00	0.043	-0.043	0.034
1000	8	0	0.5	0.8	0.5	0.15	0.15	0.15	0.50	0.25	0.25	0.043	-0.043	0.034
1000	8	0	0.5	0.8	0.5	0.15	0.15	0.15	0.50	0.25	0.50	0.043	-0.043	0.035
1000	8	0	0.5	0.8	0.5	0.15	0.15	0.15	0.50	0.50	0.00	0.044	-0.045	0.024
1000	8	0	0.5	0.8	0.5	0.15	0.15	0.15	0.50	0.50	0.25	0.044	-0.045	0.023
1000	8	0	0.5	0.8	0.5	0.15	0.15	0.15	0.50	0.50	0.50	0.044	-0.044	0.024
1000	8	0	0.5	0.8	0.5	0.30	0.30	0.30	0.00	0.00	0.00	0.133	-0.167	0.180
1000	8	0	0.5	0.8	0.5	0.30	0.30	0.30	0.00	0.00	0.25	0.133	-0.167	0.180
1000	8	0	0.5	0.8	0.5	0.30	0.30	0.30	0.00	0.00	0.50	0.132	-0.168	0.180
1000	8	0	0.5	0.8	0.5	0.30	0.30	0.30	0.00	0.25	0.00	0.148	-0.183	0.150
1000	8	0	0.5	0.8	0.5	0.30	0.30	0.30	0.00	0.25	0.25	0.148	-0.182	0.150
1000	8	0	0.5	0.8	0.5	0.30	0.30	0.30	0.00	0.25	0.50	0.148	-0.183	0.149
1000	8	0	0.5	0.8	0.5	0.30	0.30	0.30	0.00	0.50	0.00	0.163	-0.197	0.114

(continued)

N	m_1	$\frac{m_2}{m_1}$	$E(C)$	$E(R)$	$E(U)$	σ_C	σ_R	σ_U	ρ_{CR}	ρ_{CU}	ρ_{RU}	Mean Bias c	Mean Bias r	Mean Bias u
1000	8	0	0.5	0.8	0.5	0.30	0.30	0.30	0.00	0.50	0.25	0.162	-0.197	0.113
1000	8	0	0.5	0.8	0.5	0.30	0.30	0.30	0.00	0.50	0.50	0.162	-0.196	0.113
1000	8	0	0.5	0.8	0.5	0.30	0.30	0.30	0.25	0.00	0.00	0.132	-0.139	0.180
1000	8	0	0.5	0.8	0.5	0.30	0.30	0.30	0.25	0.00	0.25	0.133	-0.138	0.179
1000	8	0	0.5	0.8	0.5	0.30	0.30	0.30	0.25	0.00	0.50	0.133	-0.138	0.180
1000	8	0	0.5	0.8	0.5	0.30	0.30	0.30	0.25	0.25	0.00	0.148	-0.155	0.150
1000	8	0	0.5	0.8	0.5	0.30	0.30	0.30	0.25	0.25	0.25	0.149	-0.154	0.150
1000	8	0	0.5	0.8	0.5	0.30	0.30	0.30	0.25	0.25	0.50	0.148	-0.155	0.150
1000	8	0	0.5	0.8	0.5	0.30	0.30	0.30	0.25	0.50	0.00	0.163	-0.169	0.113
1000	8	0	0.5	0.8	0.5	0.30	0.30	0.30	0.25	0.50	0.25	0.162	-0.169	0.113
1000	8	0	0.5	0.8	0.5	0.30	0.30	0.30	0.25	0.50	0.50	0.163	-0.168	0.113
1000	8	0	0.5	0.8	0.5	0.30	0.30	0.30	0.50	0.00	0.00	0.132	-0.109	0.180
1000	8	0	0.5	0.8	0.5	0.30	0.30	0.30	0.50	0.00	0.25	0.132	-0.109	0.179
1000	8	0	0.5	0.8	0.5	0.30	0.30	0.30	0.50	0.00	0.50	0.133	-0.110	0.179
1000	8	0	0.5	0.8	0.5	0.30	0.30	0.30	0.50	0.25	0.00	0.148	-0.125	0.150
1000	8	0	0.5	0.8	0.5	0.30	0.30	0.30	0.50	0.25	0.25	0.148	-0.126	0.150
1000	8	0	0.5	0.8	0.5	0.30	0.30	0.30	0.50	0.25	0.50	0.148	-0.125	0.150
1000	8	0	0.5	0.8	0.5	0.30	0.30	0.30	0.50	0.50	0.00	0.162	-0.140	0.113
1000	8	0	0.5	0.8	0.5	0.30	0.30	0.30	0.50	0.50	0.25	0.163	-0.140	0.113
1000	8	0	0.5	0.8	0.5	0.30	0.30	0.30	0.50	0.50	0.50	0.163	-0.141	0.113
1000	8	0	0.8	0.2	0.5	0.00	0.00	0.00	0.00	0.00	0.00	0.000	0.000	0.000
1000	8	0	0.8	0.2	0.5	0.15	0.15	0.15	0.00	0.00	0.00	0.016	-0.004	0.044
1000	8	0	0.8	0.2	0.5	0.15	0.15	0.15	0.00	0.00	0.25	0.017	-0.004	0.045
1000	8	0	0.8	0.2	0.5	0.15	0.15	0.15	0.00	0.00	0.50	0.017	-0.004	0.045
1000	8	0	0.8	0.2	0.5	0.15	0.15	0.15	0.00	0.25	0.00	0.018	-0.004	0.019
1000	8	0	0.8	0.2	0.5	0.15	0.15	0.15	0.00	0.25	0.25	0.018	-0.005	0.020
1000	8	0	0.8	0.2	0.5	0.15	0.15	0.15	0.00	0.25	0.50	0.018	-0.005	0.020
1000	8	0	0.8	0.2	0.5	0.15	0.15	0.15	0.00	0.50	0.00	0.019	-0.005	-0.006
1000	8	0	0.8	0.2	0.5	0.15	0.15	0.15	0.00	0.50	0.25	0.019	-0.004	-0.007
1000	8	0	0.8	0.2	0.5	0.15	0.15	0.15	0.00	0.50	0.50	0.019	-0.005	-0.006
1000	8	0	0.8	0.2	0.5	0.15	0.15	0.15	0.25	0.00	0.00	0.016	0.002	0.044
1000	8	0	0.8	0.2	0.5	0.15	0.15	0.15	0.25	0.00	0.25	0.016	0.002	0.044
1000	8	0	0.8	0.2	0.5	0.15	0.15	0.15	0.25	0.00	0.50	0.016	0.002	0.045
1000	8	0	0.8	0.2	0.5	0.15	0.15	0.15	0.25	0.25	0.00	0.018	0.002	0.020
1000	8	0	0.8	0.2	0.5	0.15	0.15	0.15	0.25	0.25	0.25	0.018	0.001	0.020
1000	8	0	0.8	0.2	0.5	0.15	0.15	0.15	0.25	0.25	0.50	0.018	0.002	0.020
1000	8	0	0.8	0.2	0.5	0.15	0.15	0.15	0.25	0.50	0.00	0.019	0.001	-0.006
1000	8	0	0.8	0.2	0.5	0.15	0.15	0.15	0.25	0.50	0.25	0.019	0.002	-0.007
1000	8	0	0.8	0.2	0.5	0.15	0.15	0.15	0.25	0.50	0.50	0.019	0.001	-0.006

(continued)

												Mean Bias		
N	m_1	$\frac{m_2}{m_1}$	$E(C)$	$E(R)$	$E(U)$	σ_C	σ_R	σ_U	ρ_{CR}	ρ_{CU}	ρ_{RU}	c	r	u
1000	8	0	0.8	0.2	0.5	0.15	0.15	0.15	0.50	0.00	0.00	0.017	0.008	0.045
1000	8	0	0.8	0.2	0.5	0.15	0.15	0.15	0.50	0.00	0.25	0.017	0.008	0.045
1000	8	0	0.8	0.2	0.5	0.15	0.15	0.15	0.50	0.00	0.50	0.016	0.008	0.045
1000	8	0	0.8	0.2	0.5	0.15	0.15	0.15	0.50	0.25	0.00	0.018	0.007	0.021
1000	8	0	0.8	0.2	0.5	0.15	0.15	0.15	0.50	0.25	0.25	0.018	0.008	0.021
1000	8	0	0.8	0.2	0.5	0.15	0.15	0.15	0.50	0.25	0.50	0.018	0.008	0.019
1000	8	0	0.8	0.2	0.5	0.15	0.15	0.15	0.50	0.50	0.00	0.019	0.007	-0.006
1000	8	0	0.8	0.2	0.5	0.15	0.15	0.15	0.50	0.50	0.25	0.019	0.007	-0.006
1000	8	0	0.8	0.2	0.5	0.15	0.15	0.15	0.50	0.50	0.50	0.019	0.007	-0.007
1000	8	0	0.8	0.2	0.5	0.30	0.30	0.30	0.00	0.00	0.00	0.052	-0.013	0.180
1000	8	0	0.8	0.2	0.5	0.30	0.30	0.30	0.00	0.00	0.25	0.053	-0.012	0.180
1000	8	0	0.8	0.2	0.5	0.30	0.30	0.30	0.00	0.00	0.50	0.053	-0.013	0.179
1000	8	0	0.8	0.2	0.5	0.30	0.30	0.30	0.00	0.25	0.00	0.067	-0.015	0.115
1000	8	0	0.8	0.2	0.5	0.30	0.30	0.30	0.00	0.25	0.25	0.067	-0.016	0.114
1000	8	0	0.8	0.2	0.5	0.30	0.30	0.30	0.00	0.25	0.50	0.067	-0.015	0.114
1000	8	0	0.8	0.2	0.5	0.30	0.30	0.30	0.00	0.50	0.00	0.080	-0.019	0.029
1000	8	0	0.8	0.2	0.5	0.30	0.30	0.30	0.00	0.50	0.25	0.081	-0.018	0.031
1000	8	0	0.8	0.2	0.5	0.30	0.30	0.30	0.00	0.50	0.50	0.081	-0.019	0.030
1000	8	0	0.8	0.2	0.5	0.30	0.30	0.30	0.25	0.00	0.00	0.053	0.005	0.180
1000	8	0	0.8	0.2	0.5	0.30	0.30	0.30	0.25	0.00	0.25	0.053	0.005	0.179
1000	8	0	0.8	0.2	0.5	0.30	0.30	0.30	0.25	0.00	0.50	0.053	0.005	0.180
1000	8	0	0.8	0.2	0.5	0.30	0.30	0.30	0.25	0.25	0.00	0.067	0.001	0.114
1000	8	0	0.8	0.2	0.5	0.30	0.30	0.30	0.25	0.25	0.25	0.067	0.001	0.113
1000	8	0	0.8	0.2	0.5	0.30	0.30	0.30	0.25	0.25	0.50	0.067	0.002	0.115
1000	8	0	0.8	0.2	0.5	0.30	0.30	0.30	0.25	0.50	0.00	0.081	-0.002	0.031
1000	8	0	0.8	0.2	0.5	0.30	0.30	0.30	0.25	0.50	0.25	0.080	-0.001	0.030
1000	8	0	0.8	0.2	0.5	0.30	0.30	0.30	0.25	0.50	0.50	0.081	-0.001	0.029
1000	8	0	0.8	0.2	0.5	0.30	0.30	0.30	0.50	0.00	0.00	0.053	0.019	0.180
1000	8	0	0.8	0.2	0.5	0.30	0.30	0.30	0.50	0.00	0.25	0.053	0.018	0.180
1000	8	0	0.8	0.2	0.5	0.30	0.30	0.30	0.50	0.00	0.50	0.053	0.019	0.179
1000	8	0	0.8	0.2	0.5	0.30	0.30	0.30	0.50	0.25	0.00	0.067	0.015	0.113
1000	8	0	0.8	0.2	0.5	0.30	0.30	0.30	0.50	0.25	0.25	0.067	0.015	0.115
1000	8	0	0.8	0.2	0.5	0.30	0.30	0.30	0.50	0.25	0.50	0.067	0.015	0.115
1000	8	0	0.8	0.2	0.5	0.30	0.30	0.30	0.50	0.50	0.00	0.081	0.012	0.030
1000	8	0	0.8	0.2	0.5	0.30	0.30	0.30	0.50	0.50	0.25	0.081	0.012	0.031
1000	8	0	0.8	0.2	0.5	0.30	0.30	0.30	0.50	0.50	0.50	0.081	0.012	0.029
1000	8	0	0.8	0.5	0.5	0.00	0.00	0.00	0.00	0.00	0.00	0.000	0.000	-0.001
1000	8	0	0.8	0.5	0.5	0.15	0.15	0.15	0.00	0.00	0.00	0.016	-0.010	0.045
1000	8	0	0.8	0.5	0.5	0.15	0.15	0.15	0.00	0.00	0.25	0.017	-0.010	0.046

(continued)

												Mean Bias		
N	m_1	$\frac{m_2}{m_1}$	$E(C)$	$E(R)$	$E(U)$	σ_C	σ_R	σ_U	ρ_{CR}	ρ_{CU}	ρ_{RU}	c	r	u
1000	8	0	0.8	0.5	0.5	0.15	0.15	0.15	0.00	0.00	0.50	0.016	-0.010	0.045
1000	8	0	0.8	0.5	0.5	0.15	0.15	0.15	0.00	0.25	0.00	0.018	-0.011	0.020
1000	8	0	0.8	0.5	0.5	0.15	0.15	0.15	0.00	0.25	0.25	0.018	-0.011	0.020
1000	8	0	0.8	0.5	0.5	0.15	0.15	0.15	0.00	0.25	0.50	0.018	-0.011	0.020
1000	8	0	0.8	0.5	0.5	0.15	0.15	0.15	0.00	0.50	0.00	0.019	-0.012	-0.007
1000	8	0	0.8	0.5	0.5	0.15	0.15	0.15	0.00	0.50	0.25	0.018	-0.011	-0.008
1000	8	0	0.8	0.5	0.5	0.15	0.15	0.15	0.00	0.50	0.50	0.019	-0.011	-0.007
1000	8	0	0.8	0.5	0.5	0.15	0.15	0.15	0.25	0.00	0.00	0.017	-0.004	0.045
1000	8	0	0.8	0.5	0.5	0.15	0.15	0.15	0.25	0.00	0.25	0.016	-0.003	0.046
1000	8	0	0.8	0.5	0.5	0.15	0.15	0.15	0.25	0.00	0.50	0.016	-0.003	0.046
1000	8	0	0.8	0.5	0.5	0.15	0.15	0.15	0.25	0.25	0.00	0.018	-0.004	0.020
1000	8	0	0.8	0.5	0.5	0.15	0.15	0.15	0.25	0.25	0.25	0.018	-0.005	0.020
1000	8	0	0.8	0.5	0.5	0.15	0.15	0.15	0.25	0.25	0.50	0.017	-0.004	0.019
1000	8	0	0.8	0.5	0.5	0.15	0.15	0.15	0.25	0.50	0.00	0.019	-0.005	-0.007
1000	8	0	0.8	0.5	0.5	0.15	0.15	0.15	0.25	0.50	0.25	0.019	-0.005	-0.006
1000	8	0	0.8	0.5	0.5	0.15	0.15	0.15	0.25	0.50	0.50	0.019	-0.005	-0.007
1000	8	0	0.8	0.5	0.5	0.15	0.15	0.15	0.50	0.00	0.00	0.016	0.003	0.045
1000	8	0	0.8	0.5	0.5	0.15	0.15	0.15	0.50	0.00	0.25	0.016	0.003	0.046
1000	8	0	0.8	0.5	0.5	0.15	0.15	0.15	0.50	0.00	0.50	0.016	0.003	0.045
1000	8	0	0.8	0.5	0.5	0.15	0.15	0.15	0.50	0.25	0.00	0.018	0.002	0.020
1000	8	0	0.8	0.5	0.5	0.15	0.15	0.15	0.50	0.25	0.25	0.018	0.002	0.020
1000	8	0	0.8	0.5	0.5	0.15	0.15	0.15	0.50	0.25	0.50	0.018	0.002	0.019
1000	8	0	0.8	0.5	0.5	0.15	0.15	0.15	0.50	0.50	0.00	0.019	0.001	-0.006
1000	8	0	0.8	0.5	0.5	0.15	0.15	0.15	0.50	0.50	0.25	0.020	0.001	-0.006
1000	8	0	0.8	0.5	0.5	0.15	0.15	0.15	0.50	0.50	0.50	0.019	0.002	-0.006
1000	8	0	0.8	0.5	0.5	0.30	0.30	0.30	0.00	0.00	0.00	0.053	-0.031	0.179
1000	8	0	0.8	0.5	0.5	0.30	0.30	0.30	0.00	0.00	0.25	0.053	-0.031	0.180
1000	8	0	0.8	0.5	0.5	0.30	0.30	0.30	0.00	0.00	0.50	0.053	-0.031	0.180
1000	8	0	0.8	0.5	0.5	0.30	0.30	0.30	0.00	0.25	0.00	0.067	-0.038	0.114
1000	8	0	0.8	0.5	0.5	0.30	0.30	0.30	0.00	0.25	0.25	0.067	-0.039	0.114
1000	8	0	0.8	0.5	0.5	0.30	0.30	0.30	0.00	0.25	0.50	0.067	-0.038	0.113
1000	8	0	0.8	0.5	0.5	0.30	0.30	0.30	0.00	0.50	0.00	0.080	-0.045	0.030
1000	8	0	0.8	0.5	0.5	0.30	0.30	0.30	0.00	0.50	0.25	0.081	-0.046	0.030
1000	8	0	0.8	0.5	0.5	0.30	0.30	0.30	0.00	0.50	0.50	0.080	-0.046	0.030
1000	8	0	0.8	0.5	0.5	0.30	0.30	0.30	0.25	0.00	0.00	0.053	-0.009	0.180
1000	8	0	0.8	0.5	0.5	0.30	0.30	0.30	0.25	0.00	0.25	0.053	-0.009	0.179
1000	8	0	0.8	0.5	0.5	0.30	0.30	0.30	0.25	0.00	0.50	0.053	-0.010	0.178
1000	8	0	0.8	0.5	0.5	0.30	0.30	0.30	0.25	0.25	0.00	0.067	-0.018	0.115
1000	8	0	0.8	0.5	0.5	0.30	0.30	0.30	0.25	0.25	0.25	0.067	-0.017	0.115

(continued)

N	m_1	$\frac{m_2}{m_1}$	$E(C)$	$E(R)$	$E(U)$	σ_C	σ_R	σ_U	ρ_{CR}	ρ_{CU}	ρ_{RU}	Mean Bias c	Mean Bias r	Mean Bias u
1000	8	0	0.8	0.5	0.5	0.30	0.30	0.30	0.25	0.25	0.50	0.068	-0.017	0.114
1000	8	0	0.8	0.5	0.5	0.30	0.30	0.30	0.25	0.50	0.00	0.081	-0.024	0.031
1000	8	0	0.8	0.5	0.5	0.30	0.30	0.30	0.25	0.50	0.25	0.081	-0.025	0.030
1000	8	0	0.8	0.5	0.5	0.30	0.30	0.30	0.25	0.50	0.50	0.081	-0.026	0.031
1000	8	0	0.8	0.5	0.5	0.30	0.30	0.30	0.50	0.00	0.00	0.053	0.012	0.180
1000	8	0	0.8	0.5	0.5	0.30	0.30	0.30	0.50	0.00	0.25	0.053	0.012	0.180
1000	8	0	0.8	0.5	0.5	0.30	0.30	0.30	0.50	0.00	0.50	0.053	0.013	0.179
1000	8	0	0.8	0.5	0.5	0.30	0.30	0.30	0.50	0.25	0.00	0.067	0.004	0.115
1000	8	0	0.8	0.5	0.5	0.30	0.30	0.30	0.50	0.25	0.25	0.067	0.004	0.114
1000	8	0	0.8	0.5	0.5	0.30	0.30	0.30	0.50	0.25	0.50	0.067	0.003	0.114
1000	8	0	0.8	0.5	0.5	0.30	0.30	0.30	0.50	0.50	0.00	0.081	-0.004	0.031
1000	8	0	0.8	0.5	0.5	0.30	0.30	0.30	0.50	0.50	0.25	0.081	-0.004	0.031
1000	8	0	0.8	0.5	0.5	0.30	0.30	0.30	0.50	0.50	0.50	0.081	-0.005	0.030
1000	8	0	0.8	0.8	0.5	0.00	0.00	0.00	0.00	0.00	0.00	0.000	0.000	0.000
1000	8	0	0.8	0.8	0.5	0.15	0.15	0.15	0.00	0.00	0.00	0.016	-0.016	0.045
1000	8	0	0.8	0.8	0.5	0.15	0.15	0.15	0.00	0.00	0.25	0.016	-0.016	0.044
1000	8	0	0.8	0.8	0.5	0.15	0.15	0.15	0.00	0.00	0.50	0.016	-0.016	0.045
1000	8	0	0.8	0.8	0.5	0.15	0.15	0.15	0.00	0.25	0.00	0.018	-0.018	0.020
1000	8	0	0.8	0.8	0.5	0.15	0.15	0.15	0.00	0.25	0.25	0.018	-0.018	0.020
1000	8	0	0.8	0.8	0.5	0.15	0.15	0.15	0.00	0.25	0.50	0.018	-0.017	0.020
1000	8	0	0.8	0.8	0.5	0.15	0.15	0.15	0.00	0.50	0.00	0.019	-0.019	-0.006
1000	8	0	0.8	0.8	0.5	0.15	0.15	0.15	0.00	0.50	0.25	0.019	-0.019	-0.006
1000	8	0	0.8	0.8	0.5	0.15	0.15	0.15	0.00	0.50	0.50	0.019	-0.019	-0.007
1000	8	0	0.8	0.8	0.5	0.15	0.15	0.15	0.25	0.00	0.00	0.017	-0.010	0.045
1000	8	0	0.8	0.8	0.5	0.15	0.15	0.15	0.25	0.00	0.25	0.017	-0.010	0.044
1000	8	0	0.8	0.8	0.5	0.15	0.15	0.15	0.25	0.00	0.50	0.017	-0.009	0.045
1000	8	0	0.8	0.8	0.5	0.15	0.15	0.15	0.25	0.25	0.00	0.018	-0.011	0.019
1000	8	0	0.8	0.8	0.5	0.15	0.15	0.15	0.25	0.25	0.25	0.018	-0.011	0.020
1000	8	0	0.8	0.8	0.5	0.15	0.15	0.15	0.25	0.25	0.50	0.018	-0.011	0.019
1000	8	0	0.8	0.8	0.5	0.15	0.15	0.15	0.25	0.50	0.00	0.019	-0.012	-0.006
1000	8	0	0.8	0.8	0.5	0.15	0.15	0.15	0.25	0.50	0.25	0.019	-0.012	-0.006
1000	8	0	0.8	0.8	0.5	0.15	0.15	0.15	0.25	0.50	0.50	0.019	-0.013	-0.006
1000	8	0	0.8	0.8	0.5	0.15	0.15	0.15	0.50	0.00	0.00	0.017	-0.003	0.045
1000	8	0	0.8	0.8	0.5	0.15	0.15	0.15	0.50	0.00	0.25	0.017	-0.003	0.045
1000	8	0	0.8	0.8	0.5	0.15	0.15	0.15	0.50	0.00	0.50	0.016	-0.003	0.045
1000	8	0	0.8	0.8	0.5	0.15	0.15	0.15	0.50	0.25	0.00	0.018	-0.004	0.019
1000	8	0	0.8	0.8	0.5	0.15	0.15	0.15	0.50	0.25	0.25	0.018	-0.004	0.020
1000	8	0	0.8	0.8	0.5	0.15	0.15	0.15	0.50	0.25	0.50	0.018	-0.004	0.020
1000	8	0	0.8	0.8	0.5	0.15	0.15	0.15	0.50	0.50	0.00	0.020	-0.006	-0.006

(continued)

N	m_1	$\frac{m_2}{m_1}$	$E(C)$	$E(R)$	$E(U)$	σ_C	σ_R	σ_U	ρ_{CR}	ρ_{CU}	ρ_{RU}	Mean Bias		
												c	r	u
1000	8	0	0.8	0.8	0.5	0.15	0.15	0.15	0.50	0.50	0.25	0.019	-0.005	-0.007
1000	8	0	0.8	0.8	0.5	0.15	0.15	0.15	0.50	0.50	0.50	0.019	-0.005	-0.007
1000	8	0	0.8	0.8	0.5	0.30	0.30	0.30	0.00	0.00	0.00	0.053	-0.050	0.179
1000	8	0	0.8	0.8	0.5	0.30	0.30	0.30	0.00	0.00	0.25	0.053	-0.050	0.181
1000	8	0	0.8	0.8	0.5	0.30	0.30	0.30	0.00	0.00	0.50	0.053	-0.050	0.178
1000	8	0	0.8	0.8	0.5	0.30	0.30	0.30	0.00	0.25	0.00	0.067	-0.062	0.114
1000	8	0	0.8	0.8	0.5	0.30	0.30	0.30	0.00	0.25	0.25	0.067	-0.062	0.114
1000	8	0	0.8	0.8	0.5	0.30	0.30	0.30	0.00	0.25	0.50	0.067	-0.062	0.114
1000	8	0	0.8	0.8	0.5	0.30	0.30	0.30	0.00	0.50	0.00	0.081	-0.073	0.031
1000	8	0	0.8	0.8	0.5	0.30	0.30	0.30	0.00	0.50	0.25	0.080	-0.073	0.030
1000	8	0	0.8	0.8	0.5	0.30	0.30	0.30	0.00	0.50	0.50	0.081	-0.073	0.029
1000	8	0	0.8	0.8	0.5	0.30	0.30	0.30	0.25	0.00	0.00	0.053	-0.030	0.179
1000	8	0	0.8	0.8	0.5	0.30	0.30	0.30	0.25	0.00	0.25	0.053	-0.029	0.180
1000	8	0	0.8	0.8	0.5	0.30	0.30	0.30	0.25	0.00	0.50	0.053	-0.030	0.180
1000	8	0	0.8	0.8	0.5	0.30	0.30	0.30	0.25	0.25	0.00	0.067	-0.042	0.114
1000	8	0	0.8	0.8	0.5	0.30	0.30	0.30	0.25	0.25	0.25	0.067	-0.042	0.114
1000	8	0	0.8	0.8	0.5	0.30	0.30	0.30	0.25	0.25	0.50	0.067	-0.042	0.115
1000	8	0	0.8	0.8	0.5	0.30	0.30	0.30	0.25	0.50	0.00	0.081	-0.054	0.031
1000	8	0	0.8	0.8	0.5	0.30	0.30	0.30	0.25	0.50	0.25	0.081	-0.054	0.031
1000	8	0	0.8	0.8	0.5	0.30	0.30	0.30	0.25	0.50	0.50	0.081	-0.054	0.031
1000	8	0	0.8	0.8	0.5	0.30	0.30	0.30	0.50	0.00	0.00	0.053	-0.006	0.179
1000	8	0	0.8	0.8	0.5	0.30	0.30	0.30	0.50	0.00	0.25	0.053	-0.007	0.180
1000	8	0	0.8	0.8	0.5	0.30	0.30	0.30	0.50	0.00	0.50	0.053	-0.006	0.180
1000	8	0	0.8	0.8	0.5	0.30	0.30	0.30	0.50	0.25	0.00	0.067	-0.020	0.113
1000	8	0	0.8	0.8	0.5	0.30	0.30	0.30	0.50	0.25	0.25	0.067	-0.020	0.114
1000	8	0	0.8	0.8	0.5	0.30	0.30	0.30	0.50	0.25	0.50	0.068	-0.020	0.114
1000	8	0	0.8	0.8	0.5	0.30	0.30	0.30	0.50	0.50	0.00	0.081	-0.032	0.032
1000	8	0	0.8	0.8	0.5	0.30	0.30	0.30	0.50	0.50	0.25	0.081	-0.032	0.031
1000	8	0	0.8	0.8	0.5	0.30	0.30	0.30	0.50	0.50	0.50	0.081	-0.032	0.031
1000	8	1	0.2	0.2	0.5	0.00	0.00	0.00	0.00	0.00	0.00	0.000	0.001	0.000
1000	8	1	0.2	0.2	0.5	0.15	0.15	0.15	0.00	0.00	0.00	0.039	-0.033	0.016
1000	8	1	0.2	0.2	0.5	0.15	0.15	0.15	0.00	0.00	0.25	0.039	-0.033	0.016
1000	8	1	0.2	0.2	0.5	0.15	0.15	0.15	0.00	0.00	0.50	0.040	-0.033	0.016
1000	8	1	0.2	0.2	0.5	0.15	0.15	0.15	0.00	0.25	0.00	0.044	-0.036	0.014
1000	8	1	0.2	0.2	0.5	0.15	0.15	0.15	0.00	0.25	0.25	0.045	-0.036	0.013
1000	8	1	0.2	0.2	0.5	0.15	0.15	0.15	0.00	0.25	0.50	0.044	-0.035	0.013
1000	8	1	0.2	0.2	0.5	0.15	0.15	0.15	0.00	0.50	0.00	0.049	-0.039	0.011
1000	8	1	0.2	0.2	0.5	0.15	0.15	0.15	0.00	0.50	0.25	0.049	-0.039	0.011
1000	8	1	0.2	0.2	0.5	0.15	0.15	0.15	0.00	0.50	0.50	0.048	-0.039	0.011

(continued)

												Mean Bias		
N	m_1	$\frac{m_2}{m_1}$	$E(C)$	$E(R)$	$E(U)$	σ_C	σ_R	σ_U	ρ_{CR}	ρ_{CU}	ρ_{RU}	c	r	u
1000	8	1	0.2	0.2	0.5	0.15	0.15	0.15	0.25	0.00	0.00	0.040	-0.011	0.016
1000	8	1	0.2	0.2	0.5	0.15	0.15	0.15	0.25	0.00	0.25	0.040	-0.010	0.016
1000	8	1	0.2	0.2	0.5	0.15	0.15	0.15	0.25	0.00	0.50	0.040	-0.011	0.016
1000	8	1	0.2	0.2	0.5	0.15	0.15	0.15	0.25	0.25	0.00	0.044	-0.015	0.013
1000	8	1	0.2	0.2	0.5	0.15	0.15	0.15	0.25	0.25	0.25	0.044	-0.015	0.013
1000	8	1	0.2	0.2	0.5	0.15	0.15	0.15	0.25	0.25	0.50	0.044	-0.014	0.014
1000	8	1	0.2	0.2	0.5	0.15	0.15	0.15	0.25	0.50	0.00	0.048	-0.019	0.011
1000	8	1	0.2	0.2	0.5	0.15	0.15	0.15	0.25	0.50	0.25	0.049	-0.018	0.011
1000	8	1	0.2	0.2	0.5	0.15	0.15	0.15	0.25	0.50	0.50	0.049	-0.017	0.011
1000	8	1	0.2	0.2	0.5	0.15	0.15	0.15	0.50	0.00	0.00	0.039	0.012	0.016
1000	8	1	0.2	0.2	0.5	0.15	0.15	0.15	0.50	0.00	0.25	0.039	0.012	0.016
1000	8	1	0.2	0.2	0.5	0.15	0.15	0.15	0.50	0.00	0.50	0.040	0.011	0.016
1000	8	1	0.2	0.2	0.5	0.15	0.15	0.15	0.50	0.25	0.00	0.044	0.008	0.013
1000	8	1	0.2	0.2	0.5	0.15	0.15	0.15	0.50	0.25	0.25	0.044	0.008	0.014
1000	8	1	0.2	0.2	0.5	0.15	0.15	0.15	0.50	0.25	0.50	0.044	0.008	0.014
1000	8	1	0.2	0.2	0.5	0.15	0.15	0.15	0.50	0.50	0.00	0.049	0.004	0.011
1000	8	1	0.2	0.2	0.5	0.15	0.15	0.15	0.50	0.50	0.25	0.048	0.004	0.011
1000	8	1	0.2	0.2	0.5	0.15	0.15	0.15	0.50	0.50	0.50	0.049	0.004	0.011
1000	8	1	0.2	0.2	0.5	0.30	0.30	0.30	0.00	0.00	0.00	0.148	-0.085	0.064
1000	8	1	0.2	0.2	0.5	0.30	0.30	0.30	0.00	0.00	0.25	0.149	-0.086	0.064
1000	8	1	0.2	0.2	0.5	0.30	0.30	0.30	0.00	0.00	0.50	0.148	-0.085	0.065
1000	8	1	0.2	0.2	0.5	0.30	0.30	0.30	0.00	0.25	0.00	0.164	-0.091	0.057
1000	8	1	0.2	0.2	0.5	0.30	0.30	0.30	0.00	0.25	0.25	0.165	-0.091	0.056
1000	8	1	0.2	0.2	0.5	0.30	0.30	0.30	0.00	0.25	0.50	0.165	-0.090	0.056
1000	8	1	0.2	0.2	0.5	0.30	0.30	0.30	0.00	0.50	0.00	0.179	-0.094	0.046
1000	8	1	0.2	0.2	0.5	0.30	0.30	0.30	0.00	0.50	0.25	0.179	-0.094	0.048
1000	8	1	0.2	0.2	0.5	0.30	0.30	0.30	0.00	0.50	0.50	0.179	-0.094	0.047
1000	8	1	0.2	0.2	0.5	0.30	0.30	0.30	0.25	0.00	0.00	0.148	-0.036	0.065
1000	8	1	0.2	0.2	0.5	0.30	0.30	0.30	0.25	0.00	0.25	0.148	-0.035	0.065
1000	8	1	0.2	0.2	0.5	0.30	0.30	0.30	0.25	0.00	0.50	0.148	-0.037	0.064
1000	8	1	0.2	0.2	0.5	0.30	0.30	0.30	0.25	0.25	0.00	0.165	-0.044	0.056
1000	8	1	0.2	0.2	0.5	0.30	0.30	0.30	0.25	0.25	0.25	0.164	-0.044	0.057
1000	8	1	0.2	0.2	0.5	0.30	0.30	0.30	0.25	0.25	0.50	0.165	-0.044	0.056
1000	8	1	0.2	0.2	0.5	0.30	0.30	0.30	0.25	0.50	0.00	0.179	-0.050	0.047
1000	8	1	0.2	0.2	0.5	0.30	0.30	0.30	0.25	0.50	0.25	0.178	-0.049	0.048
1000	8	1	0.2	0.2	0.5	0.30	0.30	0.30	0.25	0.50	0.50	0.179	-0.050	0.047
1000	8	1	0.2	0.2	0.5	0.30	0.30	0.30	0.50	0.00	0.00	0.148	0.020	0.065
1000	8	1	0.2	0.2	0.5	0.30	0.30	0.30	0.50	0.00	0.25	0.148	0.019	0.064
1000	8	1	0.2	0.2	0.5	0.30	0.30	0.30	0.50	0.00	0.50	0.148	0.020	0.065

(continued)

												Mean Bias		
N	m_1	$\frac{m_2}{m_1}$	$E(C)$	$E(R)$	$E(U)$	σ_C	σ_R	σ_U	ρ_{CR}	ρ_{CU}	ρ_{RU}	c	r	u
1000	8	1	0.2	0.2	0.5	0.30	0.30	0.30	0.50	0.25	0.00	0.165	0.010	0.056
1000	8	1	0.2	0.2	0.5	0.30	0.30	0.30	0.50	0.25	0.25	0.166	0.010	0.056
1000	8	1	0.2	0.2	0.5	0.30	0.30	0.30	0.50	0.25	0.50	0.164	0.010	0.057
1000	8	1	0.2	0.2	0.5	0.30	0.30	0.30	0.50	0.50	0.00	0.178	0.001	0.047
1000	8	1	0.2	0.2	0.5	0.30	0.30	0.30	0.50	0.50	0.25	0.178	0.001	0.047
1000	8	1	0.2	0.2	0.5	0.30	0.30	0.30	0.50	0.50	0.50	0.179	0.002	0.046
1000	8	1	0.2	0.5	0.5	0.00	0.00	0.00	0.00	0.00	0.00	0.000	0.000	0.000
1000	8	1	0.2	0.5	0.5	0.15	0.15	0.15	0.00	0.00	0.00	0.039	-0.082	0.015
1000	8	1	0.2	0.5	0.5	0.15	0.15	0.15	0.00	0.00	0.25	0.040	-0.083	0.016
1000	8	1	0.2	0.5	0.5	0.15	0.15	0.15	0.00	0.00	0.50	0.039	-0.083	0.016
1000	8	1	0.2	0.5	0.5	0.15	0.15	0.15	0.00	0.25	0.00	0.044	-0.091	0.013
1000	8	1	0.2	0.5	0.5	0.15	0.15	0.15	0.00	0.25	0.25	0.045	-0.090	0.013
1000	8	1	0.2	0.5	0.5	0.15	0.15	0.15	0.00	0.25	0.50	0.044	-0.090	0.013
1000	8	1	0.2	0.5	0.5	0.15	0.15	0.15	0.00	0.50	0.00	0.049	-0.098	0.011
1000	8	1	0.2	0.5	0.5	0.15	0.15	0.15	0.00	0.50	0.25	0.049	-0.097	0.011
1000	8	1	0.2	0.5	0.5	0.15	0.15	0.15	0.00	0.50	0.50	0.049	-0.098	0.011
1000	8	1	0.2	0.5	0.5	0.15	0.15	0.15	0.25	0.00	0.00	0.039	-0.060	0.016
1000	8	1	0.2	0.5	0.5	0.15	0.15	0.15	0.25	0.00	0.25	0.039	-0.059	0.016
1000	8	1	0.2	0.5	0.5	0.15	0.15	0.15	0.25	0.00	0.50	0.040	-0.061	0.016
1000	8	1	0.2	0.5	0.5	0.15	0.15	0.15	0.25	0.25	0.00	0.044	-0.068	0.013
1000	8	1	0.2	0.5	0.5	0.15	0.15	0.15	0.25	0.25	0.25	0.044	-0.068	0.013
1000	8	1	0.2	0.5	0.5	0.15	0.15	0.15	0.25	0.25	0.50	0.044	-0.068	0.013
1000	8	1	0.2	0.5	0.5	0.15	0.15	0.15	0.25	0.50	0.00	0.048	-0.075	0.011
1000	8	1	0.2	0.5	0.5	0.15	0.15	0.15	0.25	0.50	0.25	0.049	-0.076	0.011
1000	8	1	0.2	0.5	0.5	0.15	0.15	0.15	0.25	0.50	0.50	0.049	-0.076	0.011
1000	8	1	0.2	0.5	0.5	0.15	0.15	0.15	0.50	0.00	0.00	0.040	-0.036	0.015
1000	8	1	0.2	0.5	0.5	0.15	0.15	0.15	0.50	0.00	0.25	0.040	-0.038	0.016
1000	8	1	0.2	0.5	0.5	0.15	0.15	0.15	0.50	0.00	0.50	0.040	-0.037	0.016
1000	8	1	0.2	0.5	0.5	0.15	0.15	0.15	0.50	0.25	0.00	0.044	-0.046	0.014
1000	8	1	0.2	0.5	0.5	0.15	0.15	0.15	0.50	0.25	0.25	0.045	-0.047	0.014
1000	8	1	0.2	0.5	0.5	0.15	0.15	0.15	0.50	0.25	0.50	0.045	-0.047	0.014
1000	8	1	0.2	0.5	0.5	0.15	0.15	0.15	0.50	0.50	0.00	0.049	-0.054	0.011
1000	8	1	0.2	0.5	0.5	0.15	0.15	0.15	0.50	0.50	0.25	0.048	-0.054	0.011
1000	8	1	0.2	0.5	0.5	0.15	0.15	0.15	0.50	0.50	0.50	0.049	-0.055	0.011
1000	8	1	0.2	0.5	0.5	0.30	0.30	0.30	0.00	0.00	0.00	0.148	-0.213	0.064
1000	8	1	0.2	0.5	0.5	0.30	0.30	0.30	0.00	0.00	0.25	0.147	-0.212	0.064
1000	8	1	0.2	0.5	0.5	0.30	0.30	0.30	0.00	0.00	0.50	0.148	-0.213	0.064
1000	8	1	0.2	0.5	0.5	0.30	0.30	0.30	0.00	0.25	0.00	0.164	-0.226	0.056
1000	8	1	0.2	0.5	0.5	0.30	0.30	0.30	0.00	0.25	0.25	0.165	-0.225	0.057

(continued)

N	m_1	$\frac{m_2}{m_1}$	$E(C)$	$E(R)$	$E(U)$	σ_C	σ_R	σ_U	ρ_{CR}	ρ_{CU}	ρ_{RU}	Mean Bias c	Mean Bias r	Mean Bias u
1000	8	1	0.2	0.5	0.5	0.30	0.30	0.30	0.00	0.25	0.50	0.165	-0.226	0.056
1000	8	1	0.2	0.5	0.5	0.30	0.30	0.30	0.00	0.50	0.00	0.179	-0.236	0.047
1000	8	1	0.2	0.5	0.5	0.30	0.30	0.30	0.00	0.50	0.25	0.178	-0.236	0.047
1000	8	1	0.2	0.5	0.5	0.30	0.30	0.30	0.00	0.50	0.50	0.179	-0.235	0.047
1000	8	1	0.2	0.5	0.5	0.30	0.30	0.30	0.25	0.00	0.00	0.149	-0.160	0.064
1000	8	1	0.2	0.5	0.5	0.30	0.30	0.30	0.25	0.00	0.25	0.149	-0.159	0.064
1000	8	1	0.2	0.5	0.5	0.30	0.30	0.30	0.25	0.00	0.50	0.148	-0.160	0.064
1000	8	1	0.2	0.5	0.5	0.30	0.30	0.30	0.25	0.25	0.00	0.165	-0.175	0.057
1000	8	1	0.2	0.5	0.5	0.30	0.30	0.30	0.25	0.25	0.25	0.165	-0.176	0.056
1000	8	1	0.2	0.5	0.5	0.30	0.30	0.30	0.25	0.25	0.50	0.164	-0.176	0.056
1000	8	1	0.2	0.5	0.5	0.30	0.30	0.30	0.25	0.50	0.00	0.178	-0.188	0.047
1000	8	1	0.2	0.5	0.5	0.30	0.30	0.30	0.25	0.50	0.25	0.179	-0.187	0.047
1000	8	1	0.2	0.5	0.5	0.30	0.30	0.30	0.25	0.50	0.50	0.179	-0.188	0.047
1000	8	1	0.2	0.5	0.5	0.30	0.30	0.30	0.50	0.00	0.00	0.147	-0.108	0.064
1000	8	1	0.2	0.5	0.5	0.30	0.30	0.30	0.50	0.00	0.25	0.148	-0.108	0.065
1000	8	1	0.2	0.5	0.5	0.30	0.30	0.30	0.50	0.00	0.50	0.148	-0.107	0.064
1000	8	1	0.2	0.5	0.5	0.30	0.30	0.30	0.50	0.25	0.00	0.164	-0.125	0.057
1000	8	1	0.2	0.5	0.5	0.30	0.30	0.30	0.50	0.25	0.25	0.164	-0.125	0.056
1000	8	1	0.2	0.5	0.5	0.30	0.30	0.30	0.50	0.25	0.50	0.165	-0.124	0.056
1000	8	1	0.2	0.5	0.5	0.30	0.30	0.30	0.50	0.50	0.00	0.179	-0.138	0.046
1000	8	1	0.2	0.5	0.5	0.30	0.30	0.30	0.50	0.50	0.25	0.179	-0.140	0.047
1000	8	1	0.2	0.5	0.5	0.30	0.30	0.30	0.50	0.50	0.50	0.179	-0.138	0.047
1000	8	1	0.2	0.8	0.5	0.00	0.00	0.00	0.00	0.00	0.00	0.000	0.002	0.000
1000	8	1	0.2	0.8	0.5	0.15	0.15	0.15	0.00	0.00	0.00	0.040	-0.132	0.016
1000	8	1	0.2	0.8	0.5	0.15	0.15	0.15	0.00	0.00	0.25	0.039	-0.130	0.016
1000	8	1	0.2	0.8	0.5	0.15	0.15	0.15	0.00	0.00	0.50	0.039	-0.131	0.016
1000	8	1	0.2	0.8	0.5	0.15	0.15	0.15	0.00	0.25	0.00	0.044	-0.144	0.014
1000	8	1	0.2	0.8	0.5	0.15	0.15	0.15	0.00	0.25	0.25	0.044	-0.145	0.014
1000	8	1	0.2	0.8	0.5	0.15	0.15	0.15	0.00	0.25	0.50	0.044	-0.144	0.013
1000	8	1	0.2	0.8	0.5	0.15	0.15	0.15	0.00	0.50	0.00	0.048	-0.155	0.011
1000	8	1	0.2	0.8	0.5	0.15	0.15	0.15	0.00	0.50	0.25	0.049	-0.157	0.011
1000	8	1	0.2	0.8	0.5	0.15	0.15	0.15	0.00	0.50	0.50	0.049	-0.156	0.011
1000	8	1	0.2	0.8	0.5	0.15	0.15	0.15	0.25	0.00	0.00	0.040	-0.111	0.016
1000	8	1	0.2	0.8	0.5	0.15	0.15	0.15	0.25	0.00	0.25	0.040	-0.110	0.016
1000	8	1	0.2	0.8	0.5	0.15	0.15	0.15	0.25	0.00	0.50	0.039	-0.111	0.016
1000	8	1	0.2	0.8	0.5	0.15	0.15	0.15	0.25	0.25	0.00	0.045	-0.124	0.014
1000	8	1	0.2	0.8	0.5	0.15	0.15	0.15	0.25	0.25	0.25	0.044	-0.125	0.014
1000	8	1	0.2	0.8	0.5	0.15	0.15	0.15	0.25	0.25	0.50	0.044	-0.123	0.013
1000	8	1	0.2	0.8	0.5	0.15	0.15	0.15	0.25	0.50	0.00	0.048	-0.136	0.011

(continued)

												Mean Bias		
N	m_1	$\frac{m_2}{m_1}$	$E(C)$	$E(R)$	$E(U)$	σ_C	σ_R	σ_U	ρ_{CR}	ρ_{CU}	ρ_{RU}	c	r	u
1000	8	1	0.2	0.8	0.5	0.15	0.15	0.15	0.25	0.50	0.25	0.049	-0.136	0.011
1000	8	1	0.2	0.8	0.5	0.15	0.15	0.15	0.25	0.50	0.50	0.049	-0.136	0.011
1000	8	1	0.2	0.8	0.5	0.15	0.15	0.15	0.50	0.00	0.00	0.040	-0.091	0.016
1000	8	1	0.2	0.8	0.5	0.15	0.15	0.15	0.50	0.00	0.25	0.040	-0.091	0.016
1000	8	1	0.2	0.8	0.5	0.15	0.15	0.15	0.50	0.00	0.50	0.040	-0.091	0.016
1000	8	1	0.2	0.8	0.5	0.15	0.15	0.15	0.50	0.25	0.00	0.044	-0.104	0.013
1000	8	1	0.2	0.8	0.5	0.15	0.15	0.15	0.50	0.25	0.25	0.044	-0.104	0.013
1000	8	1	0.2	0.8	0.5	0.15	0.15	0.15	0.50	0.25	0.50	0.045	-0.105	0.013
1000	8	1	0.2	0.8	0.5	0.15	0.15	0.15	0.50	0.50	0.00	0.049	-0.118	0.011
1000	8	1	0.2	0.8	0.5	0.15	0.15	0.15	0.50	0.50	0.25	0.049	-0.116	0.011
1000	8	1	0.2	0.8	0.5	0.15	0.15	0.15	0.50	0.50	0.50	0.049	-0.117	0.011
1000	8	1	0.2	0.8	0.5	0.30	0.30	0.30	0.00	0.00	0.00	0.148	-0.340	0.064
1000	8	1	0.2	0.8	0.5	0.30	0.30	0.30	0.00	0.00	0.25	0.148	-0.340	0.064
1000	8	1	0.2	0.8	0.5	0.30	0.30	0.30	0.00	0.00	0.50	0.149	-0.340	0.064
1000	8	1	0.2	0.8	0.5	0.30	0.30	0.30	0.00	0.25	0.00	0.165	-0.361	0.057
1000	8	1	0.2	0.8	0.5	0.30	0.30	0.30	0.00	0.25	0.25	0.165	-0.362	0.056
1000	8	1	0.2	0.8	0.5	0.30	0.30	0.30	0.00	0.25	0.50	0.164	-0.362	0.056
1000	8	1	0.2	0.8	0.5	0.30	0.30	0.30	0.00	0.50	0.00	0.179	-0.377	0.047
1000	8	1	0.2	0.8	0.5	0.30	0.30	0.30	0.00	0.50	0.25	0.179	-0.377	0.047
1000	8	1	0.2	0.8	0.5	0.30	0.30	0.30	0.00	0.50	0.50	0.178	-0.379	0.047
1000	8	1	0.2	0.8	0.5	0.30	0.30	0.30	0.25	0.00	0.00	0.148	-0.299	0.065
1000	8	1	0.2	0.8	0.5	0.30	0.30	0.30	0.25	0.00	0.25	0.148	-0.299	0.065
1000	8	1	0.2	0.8	0.5	0.30	0.30	0.30	0.25	0.00	0.50	0.148	-0.298	0.065
1000	8	1	0.2	0.8	0.5	0.30	0.30	0.30	0.25	0.25	0.00	0.165	-0.321	0.056
1000	8	1	0.2	0.8	0.5	0.30	0.30	0.30	0.25	0.25	0.25	0.165	-0.321	0.056
1000	8	1	0.2	0.8	0.5	0.30	0.30	0.30	0.25	0.25	0.50	0.164	-0.321	0.056
1000	8	1	0.2	0.8	0.5	0.30	0.30	0.30	0.25	0.50	0.00	0.178	-0.338	0.047
1000	8	1	0.2	0.8	0.5	0.30	0.30	0.30	0.25	0.50	0.25	0.179	-0.340	0.047
1000	8	1	0.2	0.8	0.5	0.30	0.30	0.30	0.25	0.50	0.50	0.179	-0.338	0.047
1000	8	1	0.2	0.8	0.5	0.30	0.30	0.30	0.50	0.00	0.00	0.148	-0.264	0.064
1000	8	1	0.2	0.8	0.5	0.30	0.30	0.30	0.50	0.00	0.25	0.149	-0.265	0.064
1000	8	1	0.2	0.8	0.5	0.30	0.30	0.30	0.50	0.00	0.50	0.148	-0.263	0.064
1000	8	1	0.2	0.8	0.5	0.30	0.30	0.30	0.50	0.25	0.00	0.165	-0.288	0.057
1000	8	1	0.2	0.8	0.5	0.30	0.30	0.30	0.50	0.25	0.25	0.165	-0.289	0.056
1000	8	1	0.2	0.8	0.5	0.30	0.30	0.30	0.50	0.25	0.50	0.165	-0.288	0.056
1000	8	1	0.2	0.8	0.5	0.30	0.30	0.30	0.50	0.50	0.00	0.179	-0.307	0.047
1000	8	1	0.2	0.8	0.5	0.30	0.30	0.30	0.50	0.50	0.25	0.178	-0.308	0.047
1000	8	1	0.2	0.8	0.5	0.30	0.30	0.30	0.50	0.50	0.50	0.179	-0.307	0.047
1000	8	1	0.5	0.2	0.5	0.00	0.00	0.00	0.00	0.00	0.00	0.000	0.000	0.000

(continued)

												Mean Bias		
N	m_1	$\frac{m_2}{m_1}$	$E(C)$	$E(R)$	$E(U)$	σ_C	σ_R	σ_U	ρ_{CR}	ρ_{CU}	ρ_{RU}	c	r	u
1000	8	1	0.5	0.2	0.5	0.15	0.15	0.15	0.00	0.00	0.00	0.021	-0.008	0.011
1000	8	1	0.5	0.2	0.5	0.15	0.15	0.15	0.00	0.00	0.25	0.022	-0.009	0.011
1000	8	1	0.5	0.2	0.5	0.15	0.15	0.15	0.00	0.00	0.50	0.023	-0.009	0.012
1000	8	1	0.5	0.2	0.5	0.15	0.15	0.15	0.00	0.25	0.00	0.028	-0.011	0.009
1000	8	1	0.5	0.2	0.5	0.15	0.15	0.15	0.00	0.25	0.25	0.028	-0.010	0.009
1000	8	1	0.5	0.2	0.5	0.15	0.15	0.15	0.00	0.25	0.50	0.028	-0.010	0.009
1000	8	1	0.5	0.2	0.5	0.15	0.15	0.15	0.00	0.50	0.00	0.034	-0.012	0.006
1000	8	1	0.5	0.2	0.5	0.15	0.15	0.15	0.00	0.50	0.25	0.033	-0.013	0.005
1000	8	1	0.5	0.2	0.5	0.15	0.15	0.15	0.00	0.50	0.50	0.034	-0.013	0.006
1000	8	1	0.5	0.2	0.5	0.15	0.15	0.15	0.25	0.00	0.00	0.023	0.002	0.011
1000	8	1	0.5	0.2	0.5	0.15	0.15	0.15	0.25	0.00	0.25	0.022	0.002	0.011
1000	8	1	0.5	0.2	0.5	0.15	0.15	0.15	0.25	0.00	0.50	0.023	0.002	0.011
1000	8	1	0.5	0.2	0.5	0.15	0.15	0.15	0.25	0.25	0.00	0.028	0.000	0.009
1000	8	1	0.5	0.2	0.5	0.15	0.15	0.15	0.25	0.25	0.25	0.028	-0.001	0.009
1000	8	1	0.5	0.2	0.5	0.15	0.15	0.15	0.25	0.25	0.50	0.028	0.000	0.008
1000	8	1	0.5	0.2	0.5	0.15	0.15	0.15	0.25	0.50	0.00	0.034	-0.003	0.006
1000	8	1	0.5	0.2	0.5	0.15	0.15	0.15	0.25	0.50	0.25	0.034	-0.002	0.006
1000	8	1	0.5	0.2	0.5	0.15	0.15	0.15	0.25	0.50	0.50	0.033	-0.002	0.005
1000	8	1	0.5	0.2	0.5	0.15	0.15	0.15	0.50	0.00	0.00	0.022	0.012	0.012
1000	8	1	0.5	0.2	0.5	0.15	0.15	0.15	0.50	0.00	0.25	0.022	0.012	0.011
1000	8	1	0.5	0.2	0.5	0.15	0.15	0.15	0.50	0.00	0.50	0.022	0.012	0.011
1000	8	1	0.5	0.2	0.5	0.15	0.15	0.15	0.50	0.25	0.00	0.028	0.010	0.009
1000	8	1	0.5	0.2	0.5	0.15	0.15	0.15	0.50	0.25	0.25	0.027	0.010	0.008
1000	8	1	0.5	0.2	0.5	0.15	0.15	0.15	0.50	0.25	0.50	0.028	0.010	0.009
1000	8	1	0.5	0.2	0.5	0.15	0.15	0.15	0.50	0.50	0.00	0.034	0.008	0.005
1000	8	1	0.5	0.2	0.5	0.15	0.15	0.15	0.50	0.50	0.25	0.033	0.008	0.006
1000	8	1	0.5	0.2	0.5	0.15	0.15	0.15	0.50	0.50	0.50	0.033	0.007	0.006
1000	8	1	0.5	0.2	0.5	0.30	0.30	0.30	0.00	0.00	0.00	0.084	-0.029	0.046
1000	8	1	0.5	0.2	0.5	0.30	0.30	0.30	0.00	0.00	0.25	0.084	-0.029	0.046
1000	8	1	0.5	0.2	0.5	0.30	0.30	0.30	0.00	0.00	0.50	0.084	-0.029	0.046
1000	8	1	0.5	0.2	0.5	0.30	0.30	0.30	0.00	0.25	0.00	0.107	-0.035	0.036
1000	8	1	0.5	0.2	0.5	0.30	0.30	0.30	0.00	0.25	0.25	0.106	-0.035	0.036
1000	8	1	0.5	0.2	0.5	0.30	0.30	0.30	0.00	0.25	0.50	0.106	-0.035	0.036
1000	8	1	0.5	0.2	0.5	0.30	0.30	0.30	0.00	0.50	0.00	0.129	-0.041	0.025
1000	8	1	0.5	0.2	0.5	0.30	0.30	0.30	0.00	0.50	0.25	0.130	-0.041	0.025
1000	8	1	0.5	0.2	0.5	0.30	0.30	0.30	0.00	0.50	0.50	0.129	-0.042	0.025
1000	8	1	0.5	0.2	0.5	0.30	0.30	0.30	0.25	0.00	0.00	0.084	0.002	0.046
1000	8	1	0.5	0.2	0.5	0.30	0.30	0.30	0.25	0.00	0.25	0.083	0.003	0.046
1000	8	1	0.5	0.2	0.5	0.30	0.30	0.30	0.25	0.00	0.50	0.084	0.002	0.046

(continued)

												Mean Bias		
N	m_1	$\frac{m_2}{m_1}$	$E(C)$	$E(R)$	$E(U)$	σ_C	σ_R	σ_U	ρ_{CR}	ρ_{CU}	ρ_{RU}	c	r	u
1000	8	1	0.5	0.2	0.5	0.30	0.30	0.30	0.25	0.25	0.00	0.107	-0.006	0.035
1000	8	1	0.5	0.2	0.5	0.30	0.30	0.30	0.25	0.25	0.25	0.106	-0.005	0.035
1000	8	1	0.5	0.2	0.5	0.30	0.30	0.30	0.25	0.25	0.50	0.106	-0.005	0.036
1000	8	1	0.5	0.2	0.5	0.30	0.30	0.30	0.25	0.50	0.00	0.130	-0.012	0.025
1000	8	1	0.5	0.2	0.5	0.30	0.30	0.30	0.25	0.50	0.25	0.129	-0.012	0.025
1000	8	1	0.5	0.2	0.5	0.30	0.30	0.30	0.25	0.50	0.50	0.129	-0.011	0.025
1000	8	1	0.5	0.2	0.5	0.30	0.30	0.30	0.50	0.00	0.00	0.084	0.034	0.046
1000	8	1	0.5	0.2	0.5	0.30	0.30	0.30	0.50	0.00	0.25	0.085	0.034	0.046
1000	8	1	0.5	0.2	0.5	0.30	0.30	0.30	0.50	0.00	0.50	0.084	0.033	0.045
1000	8	1	0.5	0.2	0.5	0.30	0.30	0.30	0.50	0.25	0.00	0.106	0.026	0.036
1000	8	1	0.5	0.2	0.5	0.30	0.30	0.30	0.50	0.25	0.25	0.107	0.026	0.036
1000	8	1	0.5	0.2	0.5	0.30	0.30	0.30	0.50	0.25	0.50	0.106	0.025	0.035
1000	8	1	0.5	0.2	0.5	0.30	0.30	0.30	0.50	0.50	0.00	0.130	0.017	0.025
1000	8	1	0.5	0.2	0.5	0.30	0.30	0.30	0.50	0.50	0.25	0.129	0.017	0.024
1000	8	1	0.5	0.2	0.5	0.30	0.30	0.30	0.50	0.50	0.50	0.129	0.017	0.025
1000	8	1	0.5	0.5	0.5	0.00	0.00	0.00	0.00	0.00	0.00	0.000	0.000	0.000
1000	8	1	0.5	0.5	0.5	0.15	0.15	0.15	0.00	0.00	0.00	0.022	-0.021	0.011
1000	8	1	0.5	0.5	0.5	0.15	0.15	0.15	0.00	0.00	0.25	0.022	-0.021	0.011
1000	8	1	0.5	0.5	0.5	0.15	0.15	0.15	0.00	0.00	0.50	0.022	-0.021	0.011
1000	8	1	0.5	0.5	0.5	0.15	0.15	0.15	0.00	0.25	0.00	0.028	-0.026	0.009
1000	8	1	0.5	0.5	0.5	0.15	0.15	0.15	0.00	0.25	0.25	0.028	-0.026	0.008
1000	8	1	0.5	0.5	0.5	0.15	0.15	0.15	0.00	0.25	0.50	0.027	-0.026	0.008
1000	8	1	0.5	0.5	0.5	0.15	0.15	0.15	0.00	0.50	0.00	0.033	-0.032	0.006
1000	8	1	0.5	0.5	0.5	0.15	0.15	0.15	0.00	0.50	0.25	0.033	-0.031	0.006
1000	8	1	0.5	0.5	0.5	0.15	0.15	0.15	0.00	0.50	0.50	0.033	-0.031	0.006
1000	8	1	0.5	0.5	0.5	0.15	0.15	0.15	0.25	0.00	0.00	0.022	-0.010	0.011
1000	8	1	0.5	0.5	0.5	0.15	0.15	0.15	0.25	0.00	0.25	0.022	-0.011	0.011
1000	8	1	0.5	0.5	0.5	0.15	0.15	0.15	0.25	0.00	0.50	0.022	-0.010	0.011
1000	8	1	0.5	0.5	0.5	0.15	0.15	0.15	0.25	0.25	0.00	0.028	-0.016	0.009
1000	8	1	0.5	0.5	0.5	0.15	0.15	0.15	0.25	0.25	0.25	0.028	-0.016	0.008
1000	8	1	0.5	0.5	0.5	0.15	0.15	0.15	0.25	0.25	0.50	0.028	-0.016	0.008
1000	8	1	0.5	0.5	0.5	0.15	0.15	0.15	0.25	0.50	0.00	0.034	-0.021	0.006
1000	8	1	0.5	0.5	0.5	0.15	0.15	0.15	0.25	0.50	0.25	0.034	-0.021	0.006
1000	8	1	0.5	0.5	0.5	0.15	0.15	0.15	0.25	0.50	0.50	0.034	-0.021	0.006
1000	8	1	0.5	0.5	0.5	0.15	0.15	0.15	0.50	0.00	0.00	0.022	0.000	0.011
1000	8	1	0.5	0.5	0.5	0.15	0.15	0.15	0.50	0.00	0.25	0.022	0.000	0.011
1000	8	1	0.5	0.5	0.5	0.15	0.15	0.15	0.50	0.00	0.50	0.022	0.001	0.011
1000	8	1	0.5	0.5	0.5	0.15	0.15	0.15	0.50	0.25	0.00	0.028	-0.005	0.008
1000	8	1	0.5	0.5	0.5	0.15	0.15	0.15	0.50	0.25	0.25	0.027	-0.005	0.008

(continued)

N	m_1	$\frac{m_2}{m_1}$	$E(C)$	$E(R)$	$E(U)$	σ_C	σ_R	σ_U	ρ_{CR}	ρ_{CU}	ρ_{RU}	Mean Bias c	Mean Bias r	Mean Bias u
1000	8	1	0.5	0.5	0.5	0.15	0.15	0.15	0.50	0.25	0.50	0.028	-0.005	0.009
1000	8	1	0.5	0.5	0.5	0.15	0.15	0.15	0.50	0.50	0.00	0.033	-0.010	0.005
1000	8	1	0.5	0.5	0.5	0.15	0.15	0.15	0.50	0.50	0.25	0.033	-0.010	0.006
1000	8	1	0.5	0.5	0.5	0.15	0.15	0.15	0.50	0.50	0.50	0.034	-0.010	0.006
1000	8	1	0.5	0.5	0.5	0.30	0.30	0.30	0.00	0.00	0.00	0.084	-0.073	0.046
1000	8	1	0.5	0.5	0.5	0.30	0.30	0.30	0.00	0.00	0.25	0.084	-0.072	0.046
1000	8	1	0.5	0.5	0.5	0.30	0.30	0.30	0.00	0.00	0.50	0.084	-0.072	0.046
1000	8	1	0.5	0.5	0.5	0.30	0.30	0.30	0.00	0.25	0.00	0.106	-0.087	0.036
1000	8	1	0.5	0.5	0.5	0.30	0.30	0.30	0.00	0.25	0.25	0.106	-0.088	0.036
1000	8	1	0.5	0.5	0.5	0.30	0.30	0.30	0.00	0.25	0.50	0.107	-0.088	0.035
1000	8	1	0.5	0.5	0.5	0.30	0.30	0.30	0.00	0.50	0.00	0.130	-0.102	0.025
1000	8	1	0.5	0.5	0.5	0.30	0.30	0.30	0.00	0.50	0.25	0.129	-0.103	0.024
1000	8	1	0.5	0.5	0.5	0.30	0.30	0.30	0.00	0.50	0.50	0.129	-0.103	0.024
1000	8	1	0.5	0.5	0.5	0.30	0.30	0.30	0.25	0.00	0.00	0.084	-0.036	0.046
1000	8	1	0.5	0.5	0.5	0.30	0.30	0.30	0.25	0.00	0.25	0.084	-0.036	0.046
1000	8	1	0.5	0.5	0.5	0.30	0.30	0.30	0.25	0.00	0.50	0.084	-0.036	0.046
1000	8	1	0.5	0.5	0.5	0.30	0.30	0.30	0.25	0.25	0.00	0.107	-0.053	0.036
1000	8	1	0.5	0.5	0.5	0.30	0.30	0.30	0.25	0.25	0.25	0.107	-0.053	0.036
1000	8	1	0.5	0.5	0.5	0.30	0.30	0.30	0.25	0.25	0.50	0.107	-0.052	0.035
1000	8	1	0.5	0.5	0.5	0.30	0.30	0.30	0.25	0.50	0.00	0.129	-0.069	0.025
1000	8	1	0.5	0.5	0.5	0.30	0.30	0.30	0.25	0.50	0.25	0.130	-0.069	0.025
1000	8	1	0.5	0.5	0.5	0.30	0.30	0.30	0.25	0.50	0.50	0.129	-0.068	0.025
1000	8	1	0.5	0.5	0.5	0.30	0.30	0.30	0.50	0.00	0.00	0.084	0.001	0.047
1000	8	1	0.5	0.5	0.5	0.30	0.30	0.30	0.50	0.00	0.25	0.085	0.002	0.045
1000	8	1	0.5	0.5	0.5	0.30	0.30	0.30	0.50	0.00	0.50	0.085	0.002	0.046
1000	8	1	0.5	0.5	0.5	0.30	0.30	0.30	0.50	0.25	0.00	0.107	-0.017	0.036
1000	8	1	0.5	0.5	0.5	0.30	0.30	0.30	0.50	0.25	0.25	0.107	-0.017	0.036
1000	8	1	0.5	0.5	0.5	0.30	0.30	0.30	0.50	0.25	0.50	0.107	-0.016	0.035
1000	8	1	0.5	0.5	0.5	0.30	0.30	0.30	0.50	0.50	0.00	0.129	-0.034	0.025
1000	8	1	0.5	0.5	0.5	0.30	0.30	0.30	0.50	0.50	0.25	0.129	-0.034	0.024
1000	8	1	0.5	0.5	0.5	0.30	0.30	0.30	0.50	0.50	0.50	0.129	-0.034	0.025
1000	8	1	0.5	0.8	0.5	0.00	0.00	0.00	0.00	0.00	0.00	0.000	0.001	0.000
1000	8	1	0.5	0.8	0.5	0.15	0.15	0.15	0.00	0.00	0.00	0.022	-0.034	0.012
1000	8	1	0.5	0.8	0.5	0.15	0.15	0.15	0.00	0.00	0.25	0.022	-0.033	0.011
1000	8	1	0.5	0.8	0.5	0.15	0.15	0.15	0.00	0.00	0.50	0.022	-0.034	0.012
1000	8	1	0.5	0.8	0.5	0.15	0.15	0.15	0.00	0.25	0.00	0.028	-0.042	0.009
1000	8	1	0.5	0.8	0.5	0.15	0.15	0.15	0.00	0.25	0.25	0.028	-0.042	0.008
1000	8	1	0.5	0.8	0.5	0.15	0.15	0.15	0.00	0.25	0.50	0.027	-0.042	0.008
1000	8	1	0.5	0.8	0.5	0.15	0.15	0.15	0.00	0.50	0.00	0.033	-0.050	0.005

(continued)

												Mean Bias		
N	m_1	$\frac{m_2}{m_1}$	$E(C)$	$E(R)$	$E(U)$	σ_C	σ_R	σ_U	ρ_{CR}	ρ_{CU}	ρ_{RU}	c	r	u
1000	8	1	0.5	0.8	0.5	0.15	0.15	0.15	0.00	0.50	0.25	0.033	-0.050	0.006
1000	8	1	0.5	0.8	0.5	0.15	0.15	0.15	0.00	0.50	0.50	0.033	-0.050	0.005
1000	8	1	0.5	0.8	0.5	0.15	0.15	0.15	0.25	0.00	0.00	0.022	-0.024	0.011
1000	8	1	0.5	0.8	0.5	0.15	0.15	0.15	0.25	0.00	0.25	0.022	-0.023	0.011
1000	8	1	0.5	0.8	0.5	0.15	0.15	0.15	0.25	0.00	0.50	0.022	-0.024	0.011
1000	8	1	0.5	0.8	0.5	0.15	0.15	0.15	0.25	0.25	0.00	0.027	-0.032	0.009
1000	8	1	0.5	0.8	0.5	0.15	0.15	0.15	0.25	0.25	0.25	0.028	-0.032	0.008
1000	8	1	0.5	0.8	0.5	0.15	0.15	0.15	0.25	0.25	0.50	0.027	-0.032	0.008
1000	8	1	0.5	0.8	0.5	0.15	0.15	0.15	0.25	0.50	0.00	0.034	-0.040	0.006
1000	8	1	0.5	0.8	0.5	0.15	0.15	0.15	0.25	0.50	0.25	0.033	-0.040	0.006
1000	8	1	0.5	0.8	0.5	0.15	0.15	0.15	0.25	0.50	0.50	0.033	-0.040	0.006
1000	8	1	0.5	0.8	0.5	0.15	0.15	0.15	0.50	0.00	0.00	0.022	-0.013	0.012
1000	8	1	0.5	0.8	0.5	0.15	0.15	0.15	0.50	0.00	0.25	0.022	-0.013	0.011
1000	8	1	0.5	0.8	0.5	0.15	0.15	0.15	0.50	0.00	0.50	0.022	-0.013	0.011
1000	8	1	0.5	0.8	0.5	0.15	0.15	0.15	0.50	0.25	0.00	0.028	-0.022	0.009
1000	8	1	0.5	0.8	0.5	0.15	0.15	0.15	0.50	0.25	0.25	0.027	-0.021	0.008
1000	8	1	0.5	0.8	0.5	0.15	0.15	0.15	0.50	0.25	0.50	0.028	-0.022	0.009
1000	8	1	0.5	0.8	0.5	0.15	0.15	0.15	0.50	0.50	0.00	0.034	-0.030	0.006
1000	8	1	0.5	0.8	0.5	0.15	0.15	0.15	0.50	0.50	0.25	0.033	-0.030	0.006
1000	8	1	0.5	0.8	0.5	0.15	0.15	0.15	0.50	0.50	0.50	0.033	-0.030	0.006
1000	8	1	0.5	0.8	0.5	0.30	0.30	0.30	0.00	0.00	0.00	0.084	-0.116	0.047
1000	8	1	0.5	0.8	0.5	0.30	0.30	0.30	0.00	0.00	0.25	0.085	-0.116	0.046
1000	8	1	0.5	0.8	0.5	0.30	0.30	0.30	0.00	0.00	0.50	0.084	-0.116	0.046
1000	8	1	0.5	0.8	0.5	0.30	0.30	0.30	0.00	0.25	0.00	0.106	-0.140	0.036
1000	8	1	0.5	0.8	0.5	0.30	0.30	0.30	0.00	0.25	0.25	0.107	-0.141	0.036
1000	8	1	0.5	0.8	0.5	0.30	0.30	0.30	0.00	0.25	0.50	0.106	-0.140	0.036
1000	8	1	0.5	0.8	0.5	0.30	0.30	0.30	0.00	0.50	0.00	0.130	-0.164	0.025
1000	8	1	0.5	0.8	0.5	0.30	0.30	0.30	0.00	0.50	0.25	0.129	-0.164	0.025
1000	8	1	0.5	0.8	0.5	0.30	0.30	0.30	0.00	0.50	0.50	0.130	-0.164	0.025
1000	8	1	0.5	0.8	0.5	0.30	0.30	0.30	0.25	0.00	0.00	0.085	-0.084	0.046
1000	8	1	0.5	0.8	0.5	0.30	0.30	0.30	0.25	0.00	0.25	0.084	-0.084	0.047
1000	8	1	0.5	0.8	0.5	0.30	0.30	0.30	0.25	0.00	0.50	0.084	-0.083	0.046
1000	8	1	0.5	0.8	0.5	0.30	0.30	0.30	0.25	0.25	0.00	0.107	-0.110	0.035
1000	8	1	0.5	0.8	0.5	0.30	0.30	0.30	0.25	0.25	0.25	0.107	-0.110	0.035
1000	8	1	0.5	0.8	0.5	0.30	0.30	0.30	0.25	0.25	0.50	0.106	-0.110	0.036
1000	8	1	0.5	0.8	0.5	0.30	0.30	0.30	0.25	0.50	0.00	0.130	-0.135	0.025
1000	8	1	0.5	0.8	0.5	0.30	0.30	0.30	0.25	0.50	0.25	0.130	-0.134	0.025
1000	8	1	0.5	0.8	0.5	0.30	0.30	0.30	0.25	0.50	0.50	0.130	-0.135	0.025
1000	8	1	0.5	0.8	0.5	0.30	0.30	0.30	0.50	0.00	0.00	0.084	-0.052	0.046

(continued)

N	m_1	$\frac{m_2}{m_1}$	$E(C)$	$E(R)$	$E(U)$	σ_C	σ_R	σ_U	ρ_{CR}	ρ_{CU}	ρ_{RU}	Mean Bias c	Mean Bias r	Mean Bias u
1000	8	1	0.5	0.8	0.5	0.30	0.30	0.30	0.50	0.00	0.25	0.084	-0.053	0.046
1000	8	1	0.5	0.8	0.5	0.30	0.30	0.30	0.50	0.00	0.50	0.084	-0.052	0.046
1000	8	1	0.5	0.8	0.5	0.30	0.30	0.30	0.50	0.25	0.00	0.106	-0.080	0.036
1000	8	1	0.5	0.8	0.5	0.30	0.30	0.30	0.50	0.25	0.25	0.107	-0.080	0.036
1000	8	1	0.5	0.8	0.5	0.30	0.30	0.30	0.50	0.25	0.50	0.107	-0.079	0.036
1000	8	1	0.5	0.8	0.5	0.30	0.30	0.30	0.50	0.50	0.00	0.129	-0.106	0.025
1000	8	1	0.5	0.8	0.5	0.30	0.30	0.30	0.50	0.50	0.25	0.129	-0.106	0.024
1000	8	1	0.5	0.8	0.5	0.30	0.30	0.30	0.50	0.50	0.50	0.130	-0.106	0.025
1000	8	1	0.8	0.2	0.5	0.00	0.00	0.00	0.00	0.00	0.00	0.000	0.000	0.000
1000	8	1	0.8	0.2	0.5	0.15	0.15	0.15	0.00	0.00	0.00	0.008	-0.002	0.005
1000	8	1	0.8	0.2	0.5	0.15	0.15	0.15	0.00	0.00	0.25	0.007	-0.002	0.006
1000	8	1	0.8	0.2	0.5	0.15	0.15	0.15	0.00	0.00	0.50	0.007	-0.002	0.005
1000	8	1	0.8	0.2	0.5	0.15	0.15	0.15	0.00	0.25	0.00	0.014	-0.003	0.002
1000	8	1	0.8	0.2	0.5	0.15	0.15	0.15	0.00	0.25	0.25	0.013	-0.004	0.002
1000	8	1	0.8	0.2	0.5	0.15	0.15	0.15	0.00	0.25	0.50	0.014	-0.003	0.002
1000	8	1	0.8	0.2	0.5	0.15	0.15	0.15	0.00	0.50	0.00	0.021	-0.005	-0.001
1000	8	1	0.8	0.2	0.5	0.15	0.15	0.15	0.00	0.50	0.25	0.020	-0.005	0.000
1000	8	1	0.8	0.2	0.5	0.15	0.15	0.15	0.00	0.50	0.50	0.021	-0.005	0.000
1000	8	1	0.8	0.2	0.5	0.15	0.15	0.15	0.25	0.00	0.00	0.008	0.004	0.006
1000	8	1	0.8	0.2	0.5	0.15	0.15	0.15	0.25	0.00	0.25	0.008	0.004	0.005
1000	8	1	0.8	0.2	0.5	0.15	0.15	0.15	0.25	0.00	0.50	0.007	0.004	0.005
1000	8	1	0.8	0.2	0.5	0.15	0.15	0.15	0.25	0.25	0.00	0.014	0.003	0.002
1000	8	1	0.8	0.2	0.5	0.15	0.15	0.15	0.25	0.25	0.25	0.014	0.002	0.002
1000	8	1	0.8	0.2	0.5	0.15	0.15	0.15	0.25	0.25	0.50	0.014	0.003	0.002
1000	8	1	0.8	0.2	0.5	0.15	0.15	0.15	0.25	0.50	0.00	0.021	0.001	0.000
1000	8	1	0.8	0.2	0.5	0.15	0.15	0.15	0.25	0.50	0.25	0.021	0.001	0.000
1000	8	1	0.8	0.2	0.5	0.15	0.15	0.15	0.25	0.50	0.50	0.021	0.001	-0.001
1000	8	1	0.8	0.2	0.5	0.15	0.15	0.15	0.50	0.00	0.00	0.007	0.010	0.006
1000	8	1	0.8	0.2	0.5	0.15	0.15	0.15	0.50	0.00	0.25	0.007	0.010	0.005
1000	8	1	0.8	0.2	0.5	0.15	0.15	0.15	0.50	0.00	0.50	0.007	0.010	0.005
1000	8	1	0.8	0.2	0.5	0.15	0.15	0.15	0.50	0.25	0.00	0.014	0.009	0.003
1000	8	1	0.8	0.2	0.5	0.15	0.15	0.15	0.50	0.25	0.25	0.014	0.008	0.002
1000	8	1	0.8	0.2	0.5	0.15	0.15	0.15	0.50	0.25	0.50	0.014	0.009	0.002
1000	8	1	0.8	0.2	0.5	0.15	0.15	0.15	0.50	0.50	0.00	0.021	0.007	-0.001
1000	8	1	0.8	0.2	0.5	0.15	0.15	0.15	0.50	0.50	0.25	0.021	0.007	-0.001
1000	8	1	0.8	0.2	0.5	0.15	0.15	0.15	0.50	0.50	0.50	0.021	0.007	-0.001
1000	8	1	0.8	0.2	0.5	0.30	0.30	0.30	0.00	0.00	0.00	0.029	-0.007	0.022
1000	8	1	0.8	0.2	0.5	0.30	0.30	0.30	0.00	0.00	0.25	0.029	-0.007	0.022
1000	8	1	0.8	0.2	0.5	0.30	0.30	0.30	0.00	0.00	0.50	0.029	-0.007	0.021

(continued)

												Mean Bias		
N	m_1	$\frac{m_2}{m_1}$	$E(C)$	$E(R)$	$E(U)$	σ_C	σ_R	σ_U	ρ_{CR}	ρ_{CU}	ρ_{RU}	c	r	u
1000	8	1	0.8	0.2	0.5	0.30	0.30	0.30	0.00	0.25	0.00	0.051	-0.013	0.011
1000	8	1	0.8	0.2	0.5	0.30	0.30	0.30	0.00	0.25	0.25	0.051	-0.012	0.012
1000	8	1	0.8	0.2	0.5	0.30	0.30	0.30	0.00	0.25	0.50	0.051	-0.012	0.011
1000	8	1	0.8	0.2	0.5	0.30	0.30	0.30	0.00	0.50	0.00	0.077	-0.018	0.003
1000	8	1	0.8	0.2	0.5	0.30	0.30	0.30	0.00	0.50	0.25	0.077	-0.018	0.003
1000	8	1	0.8	0.2	0.5	0.30	0.30	0.30	0.00	0.50	0.50	0.077	-0.017	0.003
1000	8	1	0.8	0.2	0.5	0.30	0.30	0.30	0.25	0.00	0.00	0.029	0.010	0.021
1000	8	1	0.8	0.2	0.5	0.30	0.30	0.30	0.25	0.00	0.25	0.029	0.011	0.022
1000	8	1	0.8	0.2	0.5	0.30	0.30	0.30	0.25	0.00	0.50	0.028	0.010	0.021
1000	8	1	0.8	0.2	0.5	0.30	0.30	0.30	0.25	0.25	0.00	0.052	0.005	0.011
1000	8	1	0.8	0.2	0.5	0.30	0.30	0.30	0.25	0.25	0.25	0.052	0.006	0.011
1000	8	1	0.8	0.2	0.5	0.30	0.30	0.30	0.25	0.25	0.50	0.052	0.006	0.011
1000	8	1	0.8	0.2	0.5	0.30	0.30	0.30	0.25	0.50	0.00	0.077	-0.001	0.002
1000	8	1	0.8	0.2	0.5	0.30	0.30	0.30	0.25	0.50	0.25	0.077	-0.001	0.003
1000	8	1	0.8	0.2	0.5	0.30	0.30	0.30	0.25	0.50	0.50	0.077	-0.001	0.003
1000	8	1	0.8	0.2	0.5	0.30	0.30	0.30	0.50	0.00	0.00	0.029	0.026	0.021
1000	8	1	0.8	0.2	0.5	0.30	0.30	0.30	0.50	0.00	0.25	0.029	0.025	0.021
1000	8	1	0.8	0.2	0.5	0.30	0.30	0.30	0.50	0.00	0.50	0.029	0.025	0.021
1000	8	1	0.8	0.2	0.5	0.30	0.30	0.30	0.50	0.25	0.00	0.052	0.019	0.012
1000	8	1	0.8	0.2	0.5	0.30	0.30	0.30	0.50	0.25	0.25	0.051	0.019	0.012
1000	8	1	0.8	0.2	0.5	0.30	0.30	0.30	0.50	0.25	0.50	0.051	0.019	0.011
1000	8	1	0.8	0.2	0.5	0.30	0.30	0.30	0.50	0.50	0.00	0.077	0.013	0.002
1000	8	1	0.8	0.2	0.5	0.30	0.30	0.30	0.50	0.50	0.25	0.076	0.013	0.002
1000	8	1	0.8	0.2	0.5	0.30	0.30	0.30	0.50	0.50	0.50	0.076	0.013	0.003
1000	8	1	0.8	0.5	0.5	0.00	0.00	0.00	0.00	0.00	0.00	0.000	0.000	0.000
1000	8	1	0.8	0.5	0.5	0.15	0.15	0.15	0.00	0.00	0.00	0.007	-0.004	0.005
1000	8	1	0.8	0.5	0.5	0.15	0.15	0.15	0.00	0.00	0.25	0.007	-0.005	0.006
1000	8	1	0.8	0.5	0.5	0.15	0.15	0.15	0.00	0.00	0.50	0.008	-0.005	0.005
1000	8	1	0.8	0.5	0.5	0.15	0.15	0.15	0.00	0.25	0.00	0.014	-0.009	0.002
1000	8	1	0.8	0.5	0.5	0.15	0.15	0.15	0.00	0.25	0.25	0.014	-0.008	0.002
1000	8	1	0.8	0.5	0.5	0.15	0.15	0.15	0.00	0.25	0.50	0.014	-0.008	0.002
1000	8	1	0.8	0.5	0.5	0.15	0.15	0.15	0.00	0.50	0.00	0.021	-0.012	-0.001
1000	8	1	0.8	0.5	0.5	0.15	0.15	0.15	0.00	0.50	0.25	0.021	-0.013	-0.001
1000	8	1	0.8	0.5	0.5	0.15	0.15	0.15	0.00	0.50	0.50	0.021	-0.013	0.000
1000	8	1	0.8	0.5	0.5	0.15	0.15	0.15	0.25	0.00	0.00	0.007	0.003	0.005
1000	8	1	0.8	0.5	0.5	0.15	0.15	0.15	0.25	0.00	0.25	0.007	0.002	0.005
1000	8	1	0.8	0.5	0.5	0.15	0.15	0.15	0.25	0.00	0.50	0.008	0.002	0.005
1000	8	1	0.8	0.5	0.5	0.15	0.15	0.15	0.25	0.25	0.00	0.014	-0.002	0.002
1000	8	1	0.8	0.5	0.5	0.15	0.15	0.15	0.25	0.25	0.25	0.014	-0.002	0.002

(continued)

N	m_1	$\frac{m_2}{m_1}$	$E(C)$	$E(R)$	$E(U)$	σ_C	σ_R	σ_U	ρ_{CR}	ρ_{CU}	ρ_{RU}	Mean Bias c	r	u
1000	8	1	0.8	0.5	0.5	0.15	0.15	0.15	0.25	0.25	0.50	0.014	-0.002	0.002
1000	8	1	0.8	0.5	0.5	0.15	0.15	0.15	0.25	0.50	0.00	0.021	-0.006	-0.001
1000	8	1	0.8	0.5	0.5	0.15	0.15	0.15	0.25	0.50	0.25	0.021	-0.006	-0.001
1000	8	1	0.8	0.5	0.5	0.15	0.15	0.15	0.25	0.50	0.50	0.021	-0.006	-0.001
1000	8	1	0.8	0.5	0.5	0.15	0.15	0.15	0.50	0.00	0.00	0.008	0.009	0.006
1000	8	1	0.8	0.5	0.5	0.15	0.15	0.15	0.50	0.00	0.25	0.008	0.009	0.006
1000	8	1	0.8	0.5	0.5	0.15	0.15	0.15	0.50	0.00	0.50	0.008	0.009	0.005
1000	8	1	0.8	0.5	0.5	0.15	0.15	0.15	0.50	0.25	0.00	0.014	0.005	0.003
1000	8	1	0.8	0.5	0.5	0.15	0.15	0.15	0.50	0.25	0.25	0.013	0.004	0.002
1000	8	1	0.8	0.5	0.5	0.15	0.15	0.15	0.50	0.25	0.50	0.014	0.005	0.002
1000	8	1	0.8	0.5	0.5	0.15	0.15	0.15	0.50	0.50	0.00	0.021	0.001	-0.001
1000	8	1	0.8	0.5	0.5	0.15	0.15	0.15	0.50	0.50	0.25	0.021	0.000	-0.001
1000	8	1	0.8	0.5	0.5	0.15	0.15	0.15	0.50	0.50	0.50	0.020	0.001	-0.001
1000	8	1	0.8	0.5	0.5	0.30	0.30	0.30	0.00	0.00	0.00	0.029	-0.018	0.021
1000	8	1	0.8	0.5	0.5	0.30	0.30	0.30	0.00	0.00	0.25	0.029	-0.017	0.021
1000	8	1	0.8	0.5	0.5	0.30	0.30	0.30	0.00	0.00	0.50	0.029	-0.017	0.021
1000	8	1	0.8	0.5	0.5	0.30	0.30	0.30	0.00	0.25	0.00	0.051	-0.029	0.012
1000	8	1	0.8	0.5	0.5	0.30	0.30	0.30	0.00	0.25	0.25	0.052	-0.030	0.011
1000	8	1	0.8	0.5	0.5	0.30	0.30	0.30	0.00	0.25	0.50	0.052	-0.031	0.011
1000	8	1	0.8	0.5	0.5	0.30	0.30	0.30	0.00	0.50	0.00	0.077	-0.043	0.002
1000	8	1	0.8	0.5	0.5	0.30	0.30	0.30	0.00	0.50	0.25	0.077	-0.044	0.003
1000	8	1	0.8	0.5	0.5	0.30	0.30	0.30	0.00	0.50	0.50	0.076	-0.044	0.002
1000	8	1	0.8	0.5	0.5	0.30	0.30	0.30	0.25	0.00	0.00	0.029	0.005	0.021
1000	8	1	0.8	0.5	0.5	0.30	0.30	0.30	0.25	0.00	0.25	0.029	0.005	0.021
1000	8	1	0.8	0.5	0.5	0.30	0.30	0.30	0.25	0.00	0.50	0.029	0.005	0.021
1000	8	1	0.8	0.5	0.5	0.30	0.30	0.30	0.25	0.25	0.00	0.051	-0.008	0.012
1000	8	1	0.8	0.5	0.5	0.30	0.30	0.30	0.25	0.25	0.25	0.052	-0.009	0.012
1000	8	1	0.8	0.5	0.5	0.30	0.30	0.30	0.25	0.25	0.50	0.052	-0.009	0.011
1000	8	1	0.8	0.5	0.5	0.30	0.30	0.30	0.25	0.50	0.00	0.077	-0.022	0.002
1000	8	1	0.8	0.5	0.5	0.30	0.30	0.30	0.25	0.50	0.25	0.077	-0.023	0.002
1000	8	1	0.8	0.5	0.5	0.30	0.30	0.30	0.25	0.50	0.50	0.076	-0.023	0.002
1000	8	1	0.8	0.5	0.5	0.30	0.30	0.30	0.50	0.00	0.00	0.029	0.027	0.021
1000	8	1	0.8	0.5	0.5	0.30	0.30	0.30	0.50	0.00	0.25	0.029	0.027	0.021
1000	8	1	0.8	0.5	0.5	0.30	0.30	0.30	0.50	0.00	0.50	0.028	0.027	0.022
1000	8	1	0.8	0.5	0.5	0.30	0.30	0.30	0.50	0.25	0.00	0.051	0.013	0.011
1000	8	1	0.8	0.5	0.5	0.30	0.30	0.30	0.50	0.25	0.25	0.051	0.013	0.011
1000	8	1	0.8	0.5	0.5	0.30	0.30	0.30	0.50	0.25	0.50	0.051	0.013	0.011
1000	8	1	0.8	0.5	0.5	0.30	0.30	0.30	0.50	0.50	0.00	0.077	-0.001	0.003
1000	8	1	0.8	0.5	0.5	0.30	0.30	0.30	0.50	0.50	0.25	0.077	-0.001	0.002

(continued)

												Mean Bias		
N	m_1	$\frac{m_2}{m_1}$	$E(C)$	$E(R)$	$E(U)$	σ_C	σ_R	σ_U	ρ_{CR}	ρ_{CU}	ρ_{RU}	c	r	u
1000	8	1	0.8	0.5	0.5	0.30	0.30	0.30	0.50	0.50	0.50	0.077	-0.001	0.003
1000	8	1	0.8	0.8	0.5	0.00	0.00	0.00	0.00	0.00	0.00	0.000	0.000	0.000
1000	8	1	0.8	0.8	0.5	0.15	0.15	0.15	0.00	0.00	0.00	0.007	-0.007	0.005
1000	8	1	0.8	0.8	0.5	0.15	0.15	0.15	0.00	0.00	0.25	0.008	-0.007	0.006
1000	8	1	0.8	0.8	0.5	0.15	0.15	0.15	0.00	0.00	0.50	0.008	-0.007	0.005
1000	8	1	0.8	0.8	0.5	0.15	0.15	0.15	0.00	0.25	0.00	0.014	-0.014	0.002
1000	8	1	0.8	0.8	0.5	0.15	0.15	0.15	0.00	0.25	0.25	0.014	-0.014	0.002
1000	8	1	0.8	0.8	0.5	0.15	0.15	0.15	0.00	0.25	0.50	0.014	-0.014	0.002
1000	8	1	0.8	0.8	0.5	0.15	0.15	0.15	0.00	0.50	0.00	0.021	-0.020	-0.001
1000	8	1	0.8	0.8	0.5	0.15	0.15	0.15	0.00	0.50	0.25	0.021	-0.020	-0.001
1000	8	1	0.8	0.8	0.5	0.15	0.15	0.15	0.00	0.50	0.50	0.021	-0.020	0.000
1000	8	1	0.8	0.8	0.5	0.15	0.15	0.15	0.25	0.00	0.00	0.008	-0.001	0.006
1000	8	1	0.8	0.8	0.5	0.15	0.15	0.15	0.25	0.00	0.25	0.007	0.000	0.005
1000	8	1	0.8	0.8	0.5	0.15	0.15	0.15	0.25	0.00	0.50	0.008	-0.001	0.005
1000	8	1	0.8	0.8	0.5	0.15	0.15	0.15	0.25	0.25	0.00	0.014	-0.007	0.002
1000	8	1	0.8	0.8	0.5	0.15	0.15	0.15	0.25	0.25	0.25	0.014	-0.007	0.002
1000	8	1	0.8	0.8	0.5	0.15	0.15	0.15	0.25	0.25	0.50	0.014	-0.007	0.002
1000	8	1	0.8	0.8	0.5	0.15	0.15	0.15	0.25	0.50	0.00	0.020	-0.013	-0.001
1000	8	1	0.8	0.8	0.5	0.15	0.15	0.15	0.25	0.50	0.25	0.020	-0.014	-0.001
1000	8	1	0.8	0.8	0.5	0.15	0.15	0.15	0.25	0.50	0.50	0.021	-0.014	0.000
1000	8	1	0.8	0.8	0.5	0.15	0.15	0.15	0.50	0.00	0.00	0.007	0.006	0.005
1000	8	1	0.8	0.8	0.5	0.15	0.15	0.15	0.50	0.00	0.25	0.008	0.006	0.005
1000	8	1	0.8	0.8	0.5	0.15	0.15	0.15	0.50	0.00	0.50	0.008	0.006	0.005
1000	8	1	0.8	0.8	0.5	0.15	0.15	0.15	0.50	0.25	0.00	0.014	0.000	0.002
1000	8	1	0.8	0.8	0.5	0.15	0.15	0.15	0.50	0.25	0.25	0.014	0.000	0.002
1000	8	1	0.8	0.8	0.5	0.15	0.15	0.15	0.50	0.25	0.50	0.014	0.000	0.002
1000	8	1	0.8	0.8	0.5	0.15	0.15	0.15	0.50	0.50	0.00	0.021	-0.007	0.000
1000	8	1	0.8	0.8	0.5	0.15	0.15	0.15	0.50	0.50	0.25	0.021	-0.007	0.000
1000	8	1	0.8	0.8	0.5	0.15	0.15	0.15	0.50	0.50	0.50	0.021	-0.007	-0.001
1000	8	1	0.8	0.8	0.5	0.30	0.30	0.30	0.00	0.00	0.00	0.029	-0.028	0.021
1000	8	1	0.8	0.8	0.5	0.30	0.30	0.30	0.00	0.00	0.25	0.029	-0.027	0.022
1000	8	1	0.8	0.8	0.5	0.30	0.30	0.30	0.00	0.00	0.50	0.029	-0.029	0.021
1000	8	1	0.8	0.8	0.5	0.30	0.30	0.30	0.00	0.25	0.00	0.051	-0.048	0.011
1000	8	1	0.8	0.8	0.5	0.30	0.30	0.30	0.00	0.25	0.25	0.051	-0.049	0.012
1000	8	1	0.8	0.8	0.5	0.30	0.30	0.30	0.00	0.25	0.50	0.052	-0.048	0.011
1000	8	1	0.8	0.8	0.5	0.30	0.30	0.30	0.00	0.50	0.00	0.077	-0.069	0.003
1000	8	1	0.8	0.8	0.5	0.30	0.30	0.30	0.00	0.50	0.25	0.077	-0.070	0.002
1000	8	1	0.8	0.8	0.5	0.30	0.30	0.30	0.00	0.50	0.50	0.077	-0.070	0.002
1000	8	1	0.8	0.8	0.5	0.30	0.30	0.30	0.25	0.00	0.00	0.029	-0.007	0.021

(continued)

N	m_1	$\frac{m_2}{m_1}$	$E(C)$	$E(R)$	$E(U)$	σ_C	σ_R	σ_U	ρ_{CR}	ρ_{CU}	ρ_{RU}	Mean Bias c	Mean Bias r	Mean Bias u
1000	8	1	0.8	0.8	0.5	0.30	0.30	0.30	0.25	0.00	0.25	0.029	-0.007	0.022
1000	8	1	0.8	0.8	0.5	0.30	0.30	0.30	0.25	0.00	0.50	0.028	-0.007	0.021
1000	8	1	0.8	0.8	0.5	0.30	0.30	0.30	0.25	0.25	0.00	0.051	-0.029	0.011
1000	8	1	0.8	0.8	0.5	0.30	0.30	0.30	0.25	0.25	0.25	0.052	-0.028	0.011
1000	8	1	0.8	0.8	0.5	0.30	0.30	0.30	0.25	0.25	0.50	0.052	-0.028	0.011
1000	8	1	0.8	0.8	0.5	0.30	0.30	0.30	0.25	0.50	0.00	0.076	-0.050	0.002
1000	8	1	0.8	0.8	0.5	0.30	0.30	0.30	0.25	0.50	0.25	0.077	-0.051	0.002
1000	8	1	0.8	0.8	0.5	0.30	0.30	0.30	0.25	0.50	0.50	0.077	-0.051	0.002
1000	8	1	0.8	0.8	0.5	0.30	0.30	0.30	0.50	0.00	0.00	0.028	0.016	0.022
1000	8	1	0.8	0.8	0.5	0.30	0.30	0.30	0.50	0.00	0.25	0.029	0.016	0.021
1000	8	1	0.8	0.8	0.5	0.30	0.30	0.30	0.50	0.00	0.50	0.029	0.016	0.021
1000	8	1	0.8	0.8	0.5	0.30	0.30	0.30	0.50	0.25	0.00	0.052	-0.005	0.011
1000	8	1	0.8	0.8	0.5	0.30	0.30	0.30	0.50	0.25	0.25	0.052	-0.006	0.011
1000	8	1	0.8	0.8	0.5	0.30	0.30	0.30	0.50	0.25	0.50	0.052	-0.006	0.011
1000	8	1	0.8	0.8	0.5	0.30	0.30	0.30	0.50	0.50	0.00	0.076	-0.027	0.002
1000	8	1	0.8	0.8	0.5	0.30	0.30	0.30	0.50	0.50	0.25	0.076	-0.028	0.002
1000	8	1	0.8	0.8	0.5	0.30	0.30	0.30	0.50	0.50	0.50	0.076	-0.029	0.003
1000	20	0	0.2	0.2	0.5	0.00	0.00	0.00	0.00	0.00	0.00	0.000	0.000	0.000
1000	20	0	0.2	0.2	0.5	0.15	0.15	0.15	0.00	0.00	0.00	0.066	-0.050	0.045
1000	20	0	0.2	0.2	0.5	0.15	0.15	0.15	0.00	0.00	0.25	0.066	-0.050	0.045
1000	20	0	0.2	0.2	0.5	0.15	0.15	0.15	0.00	0.00	0.50	0.066	-0.049	0.045
1000	20	0	0.2	0.2	0.5	0.15	0.15	0.15	0.00	0.25	0.00	0.067	-0.050	0.039
1000	20	0	0.2	0.2	0.5	0.15	0.15	0.15	0.00	0.25	0.25	0.067	-0.050	0.038
1000	20	0	0.2	0.2	0.5	0.15	0.15	0.15	0.00	0.25	0.50	0.067	-0.050	0.039
1000	20	0	0.2	0.2	0.5	0.15	0.15	0.15	0.00	0.50	0.00	0.067	-0.051	0.032
1000	20	0	0.2	0.2	0.5	0.15	0.15	0.15	0.00	0.50	0.25	0.068	-0.050	0.032
1000	20	0	0.2	0.2	0.5	0.15	0.15	0.15	0.00	0.50	0.50	0.067	-0.051	0.032
1000	20	0	0.2	0.2	0.5	0.15	0.15	0.15	0.25	0.00	0.00	0.066	-0.029	0.045
1000	20	0	0.2	0.2	0.5	0.15	0.15	0.15	0.25	0.00	0.25	0.066	-0.030	0.045
1000	20	0	0.2	0.2	0.5	0.15	0.15	0.15	0.25	0.00	0.50	0.066	-0.030	0.045
1000	20	0	0.2	0.2	0.5	0.15	0.15	0.15	0.25	0.25	0.00	0.067	-0.031	0.039
1000	20	0	0.2	0.2	0.5	0.15	0.15	0.15	0.25	0.25	0.25	0.067	-0.031	0.039
1000	20	0	0.2	0.2	0.5	0.15	0.15	0.15	0.25	0.25	0.50	0.067	-0.031	0.039
1000	20	0	0.2	0.2	0.5	0.15	0.15	0.15	0.25	0.50	0.00	0.068	-0.031	0.032
1000	20	0	0.2	0.2	0.5	0.15	0.15	0.15	0.25	0.50	0.25	0.068	-0.031	0.032
1000	20	0	0.2	0.2	0.5	0.15	0.15	0.15	0.25	0.50	0.50	0.068	-0.031	0.032
1000	20	0	0.2	0.2	0.5	0.15	0.15	0.15	0.50	0.00	0.00	0.066	-0.009	0.045
1000	20	0	0.2	0.2	0.5	0.15	0.15	0.15	0.50	0.00	0.25	0.066	-0.009	0.045
1000	20	0	0.2	0.2	0.5	0.15	0.15	0.15	0.50	0.00	0.50	0.066	-0.009	0.045

(continued)

N	m_1	$\frac{m_2}{m_1}$	$E(C)$	$E(R)$	$E(U)$	σ_C	σ_R	σ_U	ρ_{CR}	ρ_{CU}	ρ_{RU}	Mean Bias c	r	u
1000	20	0	0.2	0.2	0.5	0.15	0.15	0.15	0.50	0.25	0.00	0.067	-0.010	0.039
1000	20	0	0.2	0.2	0.5	0.15	0.15	0.15	0.50	0.25	0.25	0.067	-0.011	0.039
1000	20	0	0.2	0.2	0.5	0.15	0.15	0.15	0.50	0.25	0.50	0.067	-0.010	0.039
1000	20	0	0.2	0.2	0.5	0.15	0.15	0.15	0.50	0.50	0.00	0.068	-0.011	0.032
1000	20	0	0.2	0.2	0.5	0.15	0.15	0.15	0.50	0.50	0.25	0.068	-0.010	0.032
1000	20	0	0.2	0.2	0.5	0.15	0.15	0.15	0.50	0.50	0.50	0.067	-0.010	0.032
1000	20	0	0.2	0.2	0.5	0.30	0.30	0.30	0.00	0.00	0.00	0.211	-0.103	0.180
1000	20	0	0.2	0.2	0.5	0.30	0.30	0.30	0.00	0.00	0.25	0.212	-0.103	0.179
1000	20	0	0.2	0.2	0.5	0.30	0.30	0.30	0.00	0.00	0.50	0.212	-0.103	0.180
1000	20	0	0.2	0.2	0.5	0.30	0.30	0.30	0.00	0.25	0.00	0.224	-0.105	0.163
1000	20	0	0.2	0.2	0.5	0.30	0.30	0.30	0.00	0.25	0.25	0.224	-0.106	0.163
1000	20	0	0.2	0.2	0.5	0.30	0.30	0.30	0.00	0.25	0.50	0.223	-0.105	0.163
1000	20	0	0.2	0.2	0.5	0.30	0.30	0.30	0.00	0.50	0.00	0.233	-0.107	0.141
1000	20	0	0.2	0.2	0.5	0.30	0.30	0.30	0.00	0.50	0.25	0.233	-0.108	0.141
1000	20	0	0.2	0.2	0.5	0.30	0.30	0.30	0.00	0.50	0.50	0.233	-0.108	0.141
1000	20	0	0.2	0.2	0.5	0.30	0.30	0.30	0.25	0.00	0.00	0.211	-0.062	0.180
1000	20	0	0.2	0.2	0.5	0.30	0.30	0.30	0.25	0.00	0.25	0.212	-0.062	0.180
1000	20	0	0.2	0.2	0.5	0.30	0.30	0.30	0.25	0.00	0.50	0.211	-0.061	0.179
1000	20	0	0.2	0.2	0.5	0.30	0.30	0.30	0.25	0.25	0.00	0.224	-0.066	0.163
1000	20	0	0.2	0.2	0.5	0.30	0.30	0.30	0.25	0.25	0.25	0.224	-0.066	0.163
1000	20	0	0.2	0.2	0.5	0.30	0.30	0.30	0.25	0.25	0.50	0.225	-0.065	0.163
1000	20	0	0.2	0.2	0.5	0.30	0.30	0.30	0.25	0.50	0.00	0.234	-0.068	0.141
1000	20	0	0.2	0.2	0.5	0.30	0.30	0.30	0.25	0.50	0.25	0.233	-0.069	0.141
1000	20	0	0.2	0.2	0.5	0.30	0.30	0.30	0.25	0.50	0.50	0.233	-0.068	0.141
1000	20	0	0.2	0.2	0.5	0.30	0.30	0.30	0.50	0.00	0.00	0.212	-0.014	0.180
1000	20	0	0.2	0.2	0.5	0.30	0.30	0.30	0.50	0.00	0.25	0.211	-0.015	0.180
1000	20	0	0.2	0.2	0.5	0.30	0.30	0.30	0.50	0.00	0.50	0.212	-0.014	0.180
1000	20	0	0.2	0.2	0.5	0.30	0.30	0.30	0.50	0.25	0.00	0.224	-0.020	0.163
1000	20	0	0.2	0.2	0.5	0.30	0.30	0.30	0.50	0.25	0.25	0.225	-0.019	0.163
1000	20	0	0.2	0.2	0.5	0.30	0.30	0.30	0.50	0.25	0.50	0.224	-0.020	0.163
1000	20	0	0.2	0.2	0.5	0.30	0.30	0.30	0.50	0.50	0.00	0.233	-0.024	0.141
1000	20	0	0.2	0.2	0.5	0.30	0.30	0.30	0.50	0.50	0.25	0.233	-0.024	0.141
1000	20	0	0.2	0.2	0.5	0.30	0.30	0.30	0.50	0.50	0.50	0.233	-0.023	0.141
1000	20	0	0.2	0.5	0.5	0.00	0.00	0.00	0.00	0.00	0.00	0.000	0.001	0.000
1000	20	0	0.2	0.5	0.5	0.15	0.15	0.15	0.00	0.00	0.00	0.066	-0.124	0.045
1000	20	0	0.2	0.5	0.5	0.15	0.15	0.15	0.00	0.00	0.25	0.066	-0.124	0.045
1000	20	0	0.2	0.5	0.5	0.15	0.15	0.15	0.00	0.00	0.50	0.066	-0.124	0.045
1000	20	0	0.2	0.5	0.5	0.15	0.15	0.15	0.00	0.25	0.00	0.067	-0.126	0.039
1000	20	0	0.2	0.5	0.5	0.15	0.15	0.15	0.00	0.25	0.25	0.067	-0.126	0.039

(continued)

												Mean Bias		
N	m_1	$\frac{m_2}{m_1}$	$E(C)$	$E(R)$	$E(U)$	σ_C	σ_R	σ_U	ρ_{CR}	ρ_{CU}	ρ_{RU}	c	r	u
1000	20	0	0.2	0.5	0.5	0.15	0.15	0.15	0.00	0.25	0.50	0.067	-0.126	0.038
1000	20	0	0.2	0.5	0.5	0.15	0.15	0.15	0.00	0.50	0.00	0.067	-0.126	0.032
1000	20	0	0.2	0.5	0.5	0.15	0.15	0.15	0.00	0.50	0.25	0.067	-0.127	0.032
1000	20	0	0.2	0.5	0.5	0.15	0.15	0.15	0.00	0.50	0.50	0.068	-0.126	0.032
1000	20	0	0.2	0.5	0.5	0.15	0.15	0.15	0.25	0.00	0.00	0.066	-0.104	0.045
1000	20	0	0.2	0.5	0.5	0.15	0.15	0.15	0.25	0.00	0.25	0.066	-0.104	0.045
1000	20	0	0.2	0.5	0.5	0.15	0.15	0.15	0.25	0.00	0.50	0.066	-0.104	0.045
1000	20	0	0.2	0.5	0.5	0.15	0.15	0.15	0.25	0.25	0.00	0.067	-0.106	0.038
1000	20	0	0.2	0.5	0.5	0.15	0.15	0.15	0.25	0.25	0.25	0.067	-0.105	0.038
1000	20	0	0.2	0.5	0.5	0.15	0.15	0.15	0.25	0.25	0.50	0.067	-0.106	0.039
1000	20	0	0.2	0.5	0.5	0.15	0.15	0.15	0.25	0.50	0.00	0.068	-0.106	0.032
1000	20	0	0.2	0.5	0.5	0.15	0.15	0.15	0.25	0.50	0.25	0.068	-0.107	0.031
1000	20	0	0.2	0.5	0.5	0.15	0.15	0.15	0.25	0.50	0.50	0.067	-0.107	0.031
1000	20	0	0.2	0.5	0.5	0.15	0.15	0.15	0.50	0.00	0.00	0.066	-0.084	0.045
1000	20	0	0.2	0.5	0.5	0.15	0.15	0.15	0.50	0.00	0.25	0.065	-0.082	0.045
1000	20	0	0.2	0.5	0.5	0.15	0.15	0.15	0.50	0.00	0.50	0.066	-0.083	0.045
1000	20	0	0.2	0.5	0.5	0.15	0.15	0.15	0.50	0.25	0.00	0.067	-0.085	0.039
1000	20	0	0.2	0.5	0.5	0.15	0.15	0.15	0.50	0.25	0.25	0.067	-0.086	0.039
1000	20	0	0.2	0.5	0.5	0.15	0.15	0.15	0.50	0.25	0.50	0.067	-0.085	0.038
1000	20	0	0.2	0.5	0.5	0.15	0.15	0.15	0.50	0.50	0.00	0.068	-0.086	0.032
1000	20	0	0.2	0.5	0.5	0.15	0.15	0.15	0.50	0.50	0.25	0.068	-0.086	0.032
1000	20	0	0.2	0.5	0.5	0.15	0.15	0.15	0.50	0.50	0.50	0.068	-0.087	0.032
1000	20	0	0.2	0.5	0.5	0.30	0.30	0.30	0.00	0.00	0.00	0.212	-0.257	0.179
1000	20	0	0.2	0.5	0.5	0.30	0.30	0.30	0.00	0.00	0.25	0.211	-0.257	0.180
1000	20	0	0.2	0.5	0.5	0.30	0.30	0.30	0.00	0.00	0.50	0.212	-0.257	0.180
1000	20	0	0.2	0.5	0.5	0.30	0.30	0.30	0.00	0.25	0.00	0.225	-0.264	0.162
1000	20	0	0.2	0.5	0.5	0.30	0.30	0.30	0.00	0.25	0.25	0.224	-0.264	0.163
1000	20	0	0.2	0.5	0.5	0.30	0.30	0.30	0.00	0.25	0.50	0.224	-0.264	0.163
1000	20	0	0.2	0.5	0.5	0.30	0.30	0.30	0.00	0.50	0.00	0.233	-0.269	0.141
1000	20	0	0.2	0.5	0.5	0.30	0.30	0.30	0.00	0.50	0.25	0.234	-0.269	0.140
1000	20	0	0.2	0.5	0.5	0.30	0.30	0.30	0.00	0.50	0.50	0.234	-0.269	0.141
1000	20	0	0.2	0.5	0.5	0.30	0.30	0.30	0.25	0.00	0.00	0.211	-0.212	0.180
1000	20	0	0.2	0.5	0.5	0.30	0.30	0.30	0.25	0.00	0.25	0.212	-0.212	0.180
1000	20	0	0.2	0.5	0.5	0.30	0.30	0.30	0.25	0.00	0.50	0.212	-0.213	0.179
1000	20	0	0.2	0.5	0.5	0.30	0.30	0.30	0.25	0.25	0.00	0.224	-0.221	0.163
1000	20	0	0.2	0.5	0.5	0.30	0.30	0.30	0.25	0.25	0.25	0.224	-0.221	0.163
1000	20	0	0.2	0.5	0.5	0.30	0.30	0.30	0.25	0.25	0.50	0.224	-0.220	0.163
1000	20	0	0.2	0.5	0.5	0.30	0.30	0.30	0.25	0.50	0.00	0.233	-0.227	0.141
1000	20	0	0.2	0.5	0.5	0.30	0.30	0.30	0.25	0.50	0.25	0.233	-0.227	0.141

(continued)

N	m_1	$\frac{m_2}{m_1}$	$E(C)$	$E(R)$	$E(U)$	σ_C	σ_R	σ_U	ρ_{CR}	ρ_{CU}	ρ_{RU}	Mean Bias c	Mean Bias r	Mean Bias u
1000	20	0	0.2	0.5	0.5	0.30	0.30	0.30	0.25	0.50	0.50	0.233	-0.226	0.141
1000	20	0	0.2	0.5	0.5	0.30	0.30	0.30	0.50	0.00	0.00	0.211	-0.169	0.179
1000	20	0	0.2	0.5	0.5	0.30	0.30	0.30	0.50	0.00	0.25	0.212	-0.168	0.180
1000	20	0	0.2	0.5	0.5	0.30	0.30	0.30	0.50	0.00	0.50	0.212	-0.168	0.180
1000	20	0	0.2	0.5	0.5	0.30	0.30	0.30	0.50	0.25	0.00	0.224	-0.177	0.163
1000	20	0	0.2	0.5	0.5	0.30	0.30	0.30	0.50	0.25	0.25	0.225	-0.177	0.163
1000	20	0	0.2	0.5	0.5	0.30	0.30	0.30	0.50	0.25	0.50	0.224	-0.178	0.163
1000	20	0	0.2	0.5	0.5	0.30	0.30	0.30	0.50	0.50	0.00	0.233	-0.185	0.140
1000	20	0	0.2	0.5	0.5	0.30	0.30	0.30	0.50	0.50	0.25	0.233	-0.184	0.141
1000	20	0	0.2	0.5	0.5	0.30	0.30	0.30	0.50	0.50	0.50	0.233	-0.184	0.141
1000	20	0	0.2	0.8	0.5	0.00	0.00	0.00	0.00	0.00	0.00	0.000	0.001	0.000
1000	20	0	0.2	0.8	0.5	0.15	0.15	0.15	0.00	0.00	0.00	0.065	-0.198	0.045
1000	20	0	0.2	0.8	0.5	0.15	0.15	0.15	0.00	0.00	0.25	0.066	-0.198	0.045
1000	20	0	0.2	0.8	0.5	0.15	0.15	0.15	0.00	0.00	0.50	0.066	-0.198	0.045
1000	20	0	0.2	0.8	0.5	0.15	0.15	0.15	0.00	0.25	0.00	0.067	-0.202	0.039
1000	20	0	0.2	0.8	0.5	0.15	0.15	0.15	0.00	0.25	0.25	0.067	-0.200	0.038
1000	20	0	0.2	0.8	0.5	0.15	0.15	0.15	0.00	0.25	0.50	0.067	-0.201	0.039
1000	20	0	0.2	0.8	0.5	0.15	0.15	0.15	0.00	0.50	0.00	0.068	-0.202	0.031
1000	20	0	0.2	0.8	0.5	0.15	0.15	0.15	0.00	0.50	0.25	0.068	-0.203	0.032
1000	20	0	0.2	0.8	0.5	0.15	0.15	0.15	0.00	0.50	0.50	0.068	-0.202	0.032
1000	20	0	0.2	0.8	0.5	0.15	0.15	0.15	0.25	0.00	0.00	0.066	-0.179	0.045
1000	20	0	0.2	0.8	0.5	0.15	0.15	0.15	0.25	0.00	0.25	0.066	-0.180	0.045
1000	20	0	0.2	0.8	0.5	0.15	0.15	0.15	0.25	0.00	0.50	0.066	-0.179	0.045
1000	20	0	0.2	0.8	0.5	0.15	0.15	0.15	0.25	0.25	0.00	0.067	-0.183	0.039
1000	20	0	0.2	0.8	0.5	0.15	0.15	0.15	0.25	0.25	0.25	0.068	-0.182	0.039
1000	20	0	0.2	0.8	0.5	0.15	0.15	0.15	0.25	0.25	0.50	0.067	-0.182	0.038
1000	20	0	0.2	0.8	0.5	0.15	0.15	0.15	0.25	0.50	0.00	0.067	-0.184	0.031
1000	20	0	0.2	0.8	0.5	0.15	0.15	0.15	0.25	0.50	0.25	0.067	-0.183	0.032
1000	20	0	0.2	0.8	0.5	0.15	0.15	0.15	0.25	0.50	0.50	0.068	-0.184	0.032
1000	20	0	0.2	0.8	0.5	0.15	0.15	0.15	0.50	0.00	0.00	0.066	-0.161	0.045
1000	20	0	0.2	0.8	0.5	0.15	0.15	0.15	0.50	0.00	0.25	0.066	-0.162	0.045
1000	20	0	0.2	0.8	0.5	0.15	0.15	0.15	0.50	0.00	0.50	0.066	-0.163	0.045
1000	20	0	0.2	0.8	0.5	0.15	0.15	0.15	0.50	0.25	0.00	0.067	-0.163	0.038
1000	20	0	0.2	0.8	0.5	0.15	0.15	0.15	0.50	0.25	0.25	0.068	-0.164	0.039
1000	20	0	0.2	0.8	0.5	0.15	0.15	0.15	0.50	0.25	0.50	0.067	-0.166	0.039
1000	20	0	0.2	0.8	0.5	0.15	0.15	0.15	0.50	0.50	0.00	0.068	-0.166	0.032
1000	20	0	0.2	0.8	0.5	0.15	0.15	0.15	0.50	0.50	0.25	0.068	-0.166	0.032
1000	20	0	0.2	0.8	0.5	0.15	0.15	0.15	0.50	0.50	0.50	0.067	-0.166	0.032
1000	20	0	0.2	0.8	0.5	0.30	0.30	0.30	0.00	0.00	0.00	0.211	-0.411	0.180

(continued)

												Mean Bias		
N	m_1	$\frac{m_2}{m_1}$	$E(C)$	$E(R)$	$E(U)$	σ_C	σ_R	σ_U	ρ_{CR}	ρ_{CU}	ρ_{RU}	c	r	u
1000	20	0	0.2	0.8	0.5	0.30	0.30	0.30	0.00	0.00	0.25	0.212	-0.412	0.179
1000	20	0	0.2	0.8	0.5	0.30	0.30	0.30	0.00	0.00	0.50	0.211	-0.411	0.180
1000	20	0	0.2	0.8	0.5	0.30	0.30	0.30	0.00	0.25	0.00	0.224	-0.423	0.163
1000	20	0	0.2	0.8	0.5	0.30	0.30	0.30	0.00	0.25	0.25	0.225	-0.422	0.163
1000	20	0	0.2	0.8	0.5	0.30	0.30	0.30	0.00	0.25	0.50	0.224	-0.423	0.163
1000	20	0	0.2	0.8	0.5	0.30	0.30	0.30	0.00	0.50	0.00	0.233	-0.430	0.141
1000	20	0	0.2	0.8	0.5	0.30	0.30	0.30	0.00	0.50	0.25	0.233	-0.431	0.140
1000	20	0	0.2	0.8	0.5	0.30	0.30	0.30	0.00	0.50	0.50	0.233	-0.431	0.140
1000	20	0	0.2	0.8	0.5	0.30	0.30	0.30	0.25	0.00	0.00	0.211	-0.377	0.180
1000	20	0	0.2	0.8	0.5	0.30	0.30	0.30	0.25	0.00	0.25	0.211	-0.376	0.179
1000	20	0	0.2	0.8	0.5	0.30	0.30	0.30	0.25	0.00	0.50	0.212	-0.377	0.180
1000	20	0	0.2	0.8	0.5	0.30	0.30	0.30	0.25	0.25	0.00	0.224	-0.388	0.163
1000	20	0	0.2	0.8	0.5	0.30	0.30	0.30	0.25	0.25	0.25	0.225	-0.388	0.163
1000	20	0	0.2	0.8	0.5	0.30	0.30	0.30	0.25	0.25	0.50	0.224	-0.389	0.163
1000	20	0	0.2	0.8	0.5	0.30	0.30	0.30	0.25	0.50	0.00	0.232	-0.397	0.141
1000	20	0	0.2	0.8	0.5	0.30	0.30	0.30	0.25	0.50	0.25	0.234	-0.396	0.141
1000	20	0	0.2	0.8	0.5	0.30	0.30	0.30	0.25	0.50	0.50	0.234	-0.397	0.141
1000	20	0	0.2	0.8	0.5	0.30	0.30	0.30	0.50	0.00	0.00	0.212	-0.346	0.180
1000	20	0	0.2	0.8	0.5	0.30	0.30	0.30	0.50	0.00	0.25	0.212	-0.346	0.180
1000	20	0	0.2	0.8	0.5	0.30	0.30	0.30	0.50	0.00	0.50	0.211	-0.346	0.180
1000	20	0	0.2	0.8	0.5	0.30	0.30	0.30	0.50	0.25	0.00	0.224	-0.359	0.163
1000	20	0	0.2	0.8	0.5	0.30	0.30	0.30	0.50	0.25	0.25	0.224	-0.359	0.163
1000	20	0	0.2	0.8	0.5	0.30	0.30	0.30	0.50	0.25	0.50	0.225	-0.360	0.162
1000	20	0	0.2	0.8	0.5	0.30	0.30	0.30	0.50	0.50	0.00	0.233	-0.370	0.141
1000	20	0	0.2	0.8	0.5	0.30	0.30	0.30	0.50	0.50	0.25	0.234	-0.370	0.141
1000	20	0	0.2	0.8	0.5	0.30	0.30	0.30	0.50	0.50	0.50	0.233	-0.369	0.141
1000	20	0	0.5	0.2	0.5	0.00	0.00	0.00	0.00	0.00	0.00	0.000	0.000	0.000
1000	20	0	0.5	0.2	0.5	0.15	0.15	0.15	0.00	0.00	0.00	0.041	-0.015	0.045
1000	20	0	0.5	0.2	0.5	0.15	0.15	0.15	0.00	0.00	0.25	0.041	-0.015	0.045
1000	20	0	0.5	0.2	0.5	0.15	0.15	0.15	0.00	0.00	0.50	0.041	-0.015	0.045
1000	20	0	0.5	0.2	0.5	0.15	0.15	0.15	0.00	0.25	0.00	0.043	-0.016	0.034
1000	20	0	0.5	0.2	0.5	0.15	0.15	0.15	0.00	0.25	0.25	0.043	-0.016	0.034
1000	20	0	0.5	0.2	0.5	0.15	0.15	0.15	0.00	0.25	0.50	0.043	-0.016	0.034
1000	20	0	0.5	0.2	0.5	0.15	0.15	0.15	0.00	0.50	0.00	0.044	-0.016	0.023
1000	20	0	0.5	0.2	0.5	0.15	0.15	0.15	0.00	0.50	0.25	0.044	-0.016	0.024
1000	20	0	0.5	0.2	0.5	0.15	0.15	0.15	0.00	0.50	0.50	0.044	-0.016	0.023
1000	20	0	0.5	0.2	0.5	0.15	0.15	0.15	0.25	0.00	0.00	0.041	-0.005	0.045
1000	20	0	0.5	0.2	0.5	0.15	0.15	0.15	0.25	0.00	0.25	0.041	-0.005	0.045
1000	20	0	0.5	0.2	0.5	0.15	0.15	0.15	0.25	0.00	0.50	0.041	-0.005	0.045

(continued)

N	m_1	$\frac{m_2}{m_1}$	$E(C)$	$E(R)$	$E(U)$	σ_C	σ_R	σ_U	ρ_{CR}	ρ_{CU}	ρ_{RU}	Mean Bias c	Mean Bias r	Mean Bias u
1000	20	0	0.5	0.2	0.5	0.15	0.15	0.15	0.25	0.25	0.00	0.043	-0.006	0.035
1000	20	0	0.5	0.2	0.5	0.15	0.15	0.15	0.25	0.25	0.25	0.043	-0.006	0.034
1000	20	0	0.5	0.2	0.5	0.15	0.15	0.15	0.25	0.25	0.50	0.043	-0.006	0.034
1000	20	0	0.5	0.2	0.5	0.15	0.15	0.15	0.25	0.50	0.00	0.044	-0.006	0.023
1000	20	0	0.5	0.2	0.5	0.15	0.15	0.15	0.25	0.50	0.25	0.044	-0.007	0.023
1000	20	0	0.5	0.2	0.5	0.15	0.15	0.15	0.25	0.50	0.50	0.044	-0.006	0.023
1000	20	0	0.5	0.2	0.5	0.15	0.15	0.15	0.50	0.00	0.00	0.041	0.005	0.045
1000	20	0	0.5	0.2	0.5	0.15	0.15	0.15	0.50	0.00	0.25	0.041	0.004	0.045
1000	20	0	0.5	0.2	0.5	0.15	0.15	0.15	0.50	0.00	0.50	0.041	0.004	0.045
1000	20	0	0.5	0.2	0.5	0.15	0.15	0.15	0.50	0.25	0.00	0.043	0.004	0.034
1000	20	0	0.5	0.2	0.5	0.15	0.15	0.15	0.50	0.25	0.25	0.043	0.004	0.035
1000	20	0	0.5	0.2	0.5	0.15	0.15	0.15	0.50	0.25	0.50	0.042	0.004	0.034
1000	20	0	0.5	0.2	0.5	0.15	0.15	0.15	0.50	0.50	0.00	0.044	0.004	0.024
1000	20	0	0.5	0.2	0.5	0.15	0.15	0.15	0.50	0.50	0.25	0.044	0.004	0.023
1000	20	0	0.5	0.2	0.5	0.15	0.15	0.15	0.50	0.50	0.50	0.044	0.004	0.024
1000	20	0	0.5	0.2	0.5	0.30	0.30	0.30	0.00	0.00	0.00	0.132	-0.042	0.180
1000	20	0	0.5	0.2	0.5	0.30	0.30	0.30	0.00	0.00	0.25	0.132	-0.042	0.180
1000	20	0	0.5	0.2	0.5	0.30	0.30	0.30	0.00	0.00	0.50	0.132	-0.042	0.181
1000	20	0	0.5	0.2	0.5	0.30	0.30	0.30	0.00	0.25	0.00	0.148	-0.045	0.150
1000	20	0	0.5	0.2	0.5	0.30	0.30	0.30	0.00	0.25	0.25	0.148	-0.046	0.150
1000	20	0	0.5	0.2	0.5	0.30	0.30	0.30	0.00	0.25	0.50	0.148	-0.046	0.150
1000	20	0	0.5	0.2	0.5	0.30	0.30	0.30	0.00	0.50	0.00	0.162	-0.049	0.113
1000	20	0	0.5	0.2	0.5	0.30	0.30	0.30	0.00	0.50	0.25	0.163	-0.049	0.112
1000	20	0	0.5	0.2	0.5	0.30	0.30	0.30	0.00	0.50	0.50	0.163	-0.049	0.113
1000	20	0	0.5	0.2	0.5	0.30	0.30	0.30	0.25	0.00	0.00	0.132	-0.013	0.180
1000	20	0	0.5	0.2	0.5	0.30	0.30	0.30	0.25	0.00	0.25	0.132	-0.013	0.180
1000	20	0	0.5	0.2	0.5	0.30	0.30	0.30	0.25	0.00	0.50	0.132	-0.013	0.180
1000	20	0	0.5	0.2	0.5	0.30	0.30	0.30	0.25	0.25	0.00	0.148	-0.018	0.150
1000	20	0	0.5	0.2	0.5	0.30	0.30	0.30	0.25	0.25	0.25	0.148	-0.017	0.151
1000	20	0	0.5	0.2	0.5	0.30	0.30	0.30	0.25	0.25	0.50	0.148	-0.017	0.149
1000	20	0	0.5	0.2	0.5	0.30	0.30	0.30	0.25	0.50	0.00	0.163	-0.022	0.113
1000	20	0	0.5	0.2	0.5	0.30	0.30	0.30	0.25	0.50	0.25	0.162	-0.021	0.113
1000	20	0	0.5	0.2	0.5	0.30	0.30	0.30	0.25	0.50	0.50	0.163	-0.021	0.113
1000	20	0	0.5	0.2	0.5	0.30	0.30	0.30	0.50	0.00	0.00	0.131	0.016	0.180
1000	20	0	0.5	0.2	0.5	0.30	0.30	0.30	0.50	0.00	0.25	0.132	0.016	0.180
1000	20	0	0.5	0.2	0.5	0.30	0.30	0.30	0.50	0.00	0.50	0.132	0.016	0.180
1000	20	0	0.5	0.2	0.5	0.30	0.30	0.30	0.50	0.25	0.00	0.148	0.011	0.150
1000	20	0	0.5	0.2	0.5	0.30	0.30	0.30	0.50	0.25	0.25	0.148	0.011	0.150
1000	20	0	0.5	0.2	0.5	0.30	0.30	0.30	0.50	0.25	0.50	0.148	0.012	0.149

(continued)

												Mean Bias		
N	m_1	$\frac{m_2}{m_1}$	$E(C)$	$E(R)$	$E(U)$	σ_C	σ_R	σ_U	ρ_{CR}	ρ_{CU}	ρ_{RU}	c	r	u
1000	20	0	0.5	0.2	0.5	0.30	0.30	0.30	0.50	0.50	0.00	0.162	0.007	0.112
1000	20	0	0.5	0.2	0.5	0.30	0.30	0.30	0.50	0.50	0.25	0.162	0.006	0.113
1000	20	0	0.5	0.2	0.5	0.30	0.30	0.30	0.50	0.50	0.50	0.163	0.007	0.113
1000	20	0	0.5	0.5	0.5	0.00	0.00	0.00	0.00	0.00	0.00	0.000	0.000	0.000
1000	20	0	0.5	0.5	0.5	0.15	0.15	0.15	0.00	0.00	0.00	0.042	-0.038	0.045
1000	20	0	0.5	0.5	0.5	0.15	0.15	0.15	0.00	0.00	0.25	0.041	-0.038	0.045
1000	20	0	0.5	0.5	0.5	0.15	0.15	0.15	0.00	0.00	0.50	0.041	-0.038	0.045
1000	20	0	0.5	0.5	0.5	0.15	0.15	0.15	0.00	0.25	0.00	0.043	-0.039	0.035
1000	20	0	0.5	0.5	0.5	0.15	0.15	0.15	0.00	0.25	0.25	0.042	-0.039	0.034
1000	20	0	0.5	0.5	0.5	0.15	0.15	0.15	0.00	0.25	0.50	0.043	-0.039	0.034
1000	20	0	0.5	0.5	0.5	0.15	0.15	0.15	0.00	0.50	0.00	0.044	-0.040	0.023
1000	20	0	0.5	0.5	0.5	0.15	0.15	0.15	0.00	0.50	0.25	0.044	-0.041	0.024
1000	20	0	0.5	0.5	0.5	0.15	0.15	0.15	0.00	0.50	0.50	0.044	-0.040	0.024
1000	20	0	0.5	0.5	0.5	0.15	0.15	0.15	0.25	0.00	0.00	0.041	-0.027	0.045
1000	20	0	0.5	0.5	0.5	0.15	0.15	0.15	0.25	0.00	0.25	0.041	-0.027	0.045
1000	20	0	0.5	0.5	0.5	0.15	0.15	0.15	0.25	0.00	0.50	0.041	-0.028	0.045
1000	20	0	0.5	0.5	0.5	0.15	0.15	0.15	0.25	0.25	0.00	0.043	-0.029	0.035
1000	20	0	0.5	0.5	0.5	0.15	0.15	0.15	0.25	0.25	0.25	0.043	-0.029	0.034
1000	20	0	0.5	0.5	0.5	0.15	0.15	0.15	0.25	0.25	0.50	0.043	-0.029	0.035
1000	20	0	0.5	0.5	0.5	0.15	0.15	0.15	0.25	0.50	0.00	0.044	-0.030	0.024
1000	20	0	0.5	0.5	0.5	0.15	0.15	0.15	0.25	0.50	0.25	0.044	-0.030	0.023
1000	20	0	0.5	0.5	0.5	0.15	0.15	0.15	0.25	0.50	0.50	0.044	-0.030	0.023
1000	20	0	0.5	0.5	0.5	0.15	0.15	0.15	0.50	0.00	0.00	0.041	-0.017	0.045
1000	20	0	0.5	0.5	0.5	0.15	0.15	0.15	0.50	0.00	0.25	0.041	-0.017	0.045
1000	20	0	0.5	0.5	0.5	0.15	0.15	0.15	0.50	0.00	0.50	0.041	-0.018	0.045
1000	20	0	0.5	0.5	0.5	0.15	0.15	0.15	0.50	0.25	0.00	0.042	-0.019	0.034
1000	20	0	0.5	0.5	0.5	0.15	0.15	0.15	0.50	0.25	0.25	0.043	-0.019	0.034
1000	20	0	0.5	0.5	0.5	0.15	0.15	0.15	0.50	0.25	0.50	0.043	-0.018	0.034
1000	20	0	0.5	0.5	0.5	0.15	0.15	0.15	0.50	0.50	0.00	0.044	-0.019	0.023
1000	20	0	0.5	0.5	0.5	0.15	0.15	0.15	0.50	0.50	0.25	0.044	-0.019	0.024
1000	20	0	0.5	0.5	0.5	0.15	0.15	0.15	0.50	0.50	0.50	0.044	-0.020	0.024
1000	20	0	0.5	0.5	0.5	0.30	0.30	0.30	0.00	0.00	0.00	0.132	-0.104	0.181
1000	20	0	0.5	0.5	0.5	0.30	0.30	0.30	0.00	0.00	0.25	0.132	-0.105	0.180
1000	20	0	0.5	0.5	0.5	0.30	0.30	0.30	0.00	0.00	0.50	0.132	-0.104	0.180
1000	20	0	0.5	0.5	0.5	0.30	0.30	0.30	0.00	0.25	0.00	0.149	-0.114	0.149
1000	20	0	0.5	0.5	0.5	0.30	0.30	0.30	0.00	0.25	0.25	0.148	-0.114	0.150
1000	20	0	0.5	0.5	0.5	0.30	0.30	0.30	0.00	0.25	0.50	0.148	-0.114	0.150
1000	20	0	0.5	0.5	0.5	0.30	0.30	0.30	0.00	0.50	0.00	0.163	-0.123	0.113
1000	20	0	0.5	0.5	0.5	0.30	0.30	0.30	0.00	0.50	0.25	0.162	-0.122	0.113

(continued)

												Mean Bias		
N	m_1	$\frac{m_2}{m_1}$	$E(C)$	$E(R)$	$E(U)$	σ_C	σ_R	σ_U	ρ_{CR}	ρ_{CU}	ρ_{RU}	c	r	u
1000	20	0	0.5	0.5	0.5	0.30	0.30	0.30	0.00	0.50	0.50	0.163	-0.123	0.113
1000	20	0	0.5	0.5	0.5	0.30	0.30	0.30	0.25	0.00	0.00	0.132	-0.071	0.180
1000	20	0	0.5	0.5	0.5	0.30	0.30	0.30	0.25	0.00	0.25	0.132	-0.071	0.180
1000	20	0	0.5	0.5	0.5	0.30	0.30	0.30	0.25	0.00	0.50	0.133	-0.070	0.180
1000	20	0	0.5	0.5	0.5	0.30	0.30	0.30	0.25	0.25	0.00	0.148	-0.082	0.150
1000	20	0	0.5	0.5	0.5	0.30	0.30	0.30	0.25	0.25	0.25	0.148	-0.081	0.150
1000	20	0	0.5	0.5	0.5	0.30	0.30	0.30	0.25	0.25	0.50	0.148	-0.081	0.149
1000	20	0	0.5	0.5	0.5	0.30	0.30	0.30	0.25	0.50	0.00	0.163	-0.090	0.113
1000	20	0	0.5	0.5	0.5	0.30	0.30	0.30	0.25	0.50	0.25	0.163	-0.090	0.113
1000	20	0	0.5	0.5	0.5	0.30	0.30	0.30	0.25	0.50	0.50	0.162	-0.091	0.113
1000	20	0	0.5	0.5	0.5	0.30	0.30	0.30	0.50	0.00	0.00	0.132	-0.036	0.179
1000	20	0	0.5	0.5	0.5	0.30	0.30	0.30	0.50	0.00	0.25	0.132	-0.036	0.180
1000	20	0	0.5	0.5	0.5	0.30	0.30	0.30	0.50	0.00	0.50	0.132	-0.037	0.180
1000	20	0	0.5	0.5	0.5	0.30	0.30	0.30	0.50	0.25	0.00	0.148	-0.048	0.150
1000	20	0	0.5	0.5	0.5	0.30	0.30	0.30	0.50	0.25	0.25	0.148	-0.048	0.150
1000	20	0	0.5	0.5	0.5	0.30	0.30	0.30	0.50	0.25	0.50	0.149	-0.047	0.150
1000	20	0	0.5	0.5	0.5	0.30	0.30	0.30	0.50	0.50	0.00	0.163	-0.057	0.113
1000	20	0	0.5	0.5	0.5	0.30	0.30	0.30	0.50	0.50	0.25	0.163	-0.057	0.112
1000	20	0	0.5	0.5	0.5	0.30	0.30	0.30	0.50	0.50	0.50	0.162	-0.058	0.113
1000	20	0	0.5	0.8	0.5	0.00	0.00	0.00	0.00	0.00	0.00	0.000	0.001	0.000
1000	20	0	0.5	0.8	0.5	0.15	0.15	0.15	0.00	0.00	0.00	0.041	-0.061	0.045
1000	20	0	0.5	0.8	0.5	0.15	0.15	0.15	0.00	0.00	0.25	0.041	-0.060	0.045
1000	20	0	0.5	0.8	0.5	0.15	0.15	0.15	0.00	0.00	0.50	0.041	-0.061	0.045
1000	20	0	0.5	0.8	0.5	0.15	0.15	0.15	0.00	0.25	0.00	0.043	-0.063	0.034
1000	20	0	0.5	0.8	0.5	0.15	0.15	0.15	0.00	0.25	0.25	0.043	-0.063	0.035
1000	20	0	0.5	0.8	0.5	0.15	0.15	0.15	0.00	0.25	0.50	0.042	-0.062	0.034
1000	20	0	0.5	0.8	0.5	0.15	0.15	0.15	0.00	0.50	0.00	0.044	-0.065	0.024
1000	20	0	0.5	0.8	0.5	0.15	0.15	0.15	0.00	0.50	0.25	0.044	-0.065	0.023
1000	20	0	0.5	0.8	0.5	0.15	0.15	0.15	0.00	0.50	0.50	0.044	-0.064	0.024
1000	20	0	0.5	0.8	0.5	0.15	0.15	0.15	0.25	0.00	0.00	0.041	-0.051	0.045
1000	20	0	0.5	0.8	0.5	0.15	0.15	0.15	0.25	0.00	0.25	0.041	-0.051	0.045
1000	20	0	0.5	0.8	0.5	0.15	0.15	0.15	0.25	0.00	0.50	0.041	-0.051	0.045
1000	20	0	0.5	0.8	0.5	0.15	0.15	0.15	0.25	0.25	0.00	0.043	-0.053	0.034
1000	20	0	0.5	0.8	0.5	0.15	0.15	0.15	0.25	0.25	0.25	0.043	-0.053	0.035
1000	20	0	0.5	0.8	0.5	0.15	0.15	0.15	0.25	0.25	0.50	0.043	-0.053	0.035
1000	20	0	0.5	0.8	0.5	0.15	0.15	0.15	0.25	0.50	0.00	0.044	-0.055	0.024
1000	20	0	0.5	0.8	0.5	0.15	0.15	0.15	0.25	0.50	0.25	0.043	-0.054	0.024
1000	20	0	0.5	0.8	0.5	0.15	0.15	0.15	0.25	0.50	0.50	0.044	-0.054	0.024
1000	20	0	0.5	0.8	0.5	0.15	0.15	0.15	0.50	0.00	0.00	0.041	-0.041	0.044

(continued)

												Mean Bias		
N	m_1	$\frac{m_2}{m_1}$	$E(C)$	$E(R)$	$E(U)$	σ_C	σ_R	σ_U	ρ_{CR}	ρ_{CU}	ρ_{RU}	c	r	u
1000	20	0	0.5	0.8	0.5	0.15	0.15	0.15	0.50	0.00	0.25	0.041	-0.041	0.045
1000	20	0	0.5	0.8	0.5	0.15	0.15	0.15	0.50	0.00	0.50	0.041	-0.041	0.045
1000	20	0	0.5	0.8	0.5	0.15	0.15	0.15	0.50	0.25	0.00	0.043	-0.043	0.035
1000	20	0	0.5	0.8	0.5	0.15	0.15	0.15	0.50	0.25	0.25	0.043	-0.043	0.034
1000	20	0	0.5	0.8	0.5	0.15	0.15	0.15	0.50	0.25	0.50	0.043	-0.043	0.034
1000	20	0	0.5	0.8	0.5	0.15	0.15	0.15	0.50	0.50	0.00	0.044	-0.045	0.024
1000	20	0	0.5	0.8	0.5	0.15	0.15	0.15	0.50	0.50	0.25	0.044	-0.045	0.024
1000	20	0	0.5	0.8	0.5	0.15	0.15	0.15	0.50	0.50	0.50	0.044	-0.044	0.023
1000	20	0	0.5	0.8	0.5	0.30	0.30	0.30	0.00	0.00	0.00	0.133	-0.168	0.179
1000	20	0	0.5	0.8	0.5	0.30	0.30	0.30	0.00	0.00	0.25	0.132	-0.167	0.180
1000	20	0	0.5	0.8	0.5	0.30	0.30	0.30	0.00	0.00	0.50	0.132	-0.167	0.180
1000	20	0	0.5	0.8	0.5	0.30	0.30	0.30	0.00	0.25	0.00	0.148	-0.182	0.150
1000	20	0	0.5	0.8	0.5	0.30	0.30	0.30	0.00	0.25	0.25	0.148	-0.183	0.150
1000	20	0	0.5	0.8	0.5	0.30	0.30	0.30	0.00	0.25	0.50	0.148	-0.182	0.150
1000	20	0	0.5	0.8	0.5	0.30	0.30	0.30	0.00	0.50	0.00	0.163	-0.196	0.113
1000	20	0	0.5	0.8	0.5	0.30	0.30	0.30	0.00	0.50	0.25	0.163	-0.196	0.114
1000	20	0	0.5	0.8	0.5	0.30	0.30	0.30	0.00	0.50	0.50	0.163	-0.196	0.113
1000	20	0	0.5	0.8	0.5	0.30	0.30	0.30	0.25	0.00	0.00	0.132	-0.138	0.180
1000	20	0	0.5	0.8	0.5	0.30	0.30	0.30	0.25	0.00	0.25	0.132	-0.138	0.180
1000	20	0	0.5	0.8	0.5	0.30	0.30	0.30	0.25	0.00	0.50	0.133	-0.138	0.180
1000	20	0	0.5	0.8	0.5	0.30	0.30	0.30	0.25	0.25	0.00	0.148	-0.154	0.151
1000	20	0	0.5	0.8	0.5	0.30	0.30	0.30	0.25	0.25	0.25	0.148	-0.154	0.150
1000	20	0	0.5	0.8	0.5	0.30	0.30	0.30	0.25	0.25	0.50	0.148	-0.155	0.150
1000	20	0	0.5	0.8	0.5	0.30	0.30	0.30	0.25	0.50	0.00	0.162	-0.169	0.113
1000	20	0	0.5	0.8	0.5	0.30	0.30	0.30	0.25	0.50	0.25	0.163	-0.169	0.113
1000	20	0	0.5	0.8	0.5	0.30	0.30	0.30	0.25	0.50	0.50	0.163	-0.169	0.113
1000	20	0	0.5	0.8	0.5	0.30	0.30	0.30	0.50	0.00	0.00	0.132	-0.109	0.180
1000	20	0	0.5	0.8	0.5	0.30	0.30	0.30	0.50	0.00	0.25	0.133	-0.109	0.180
1000	20	0	0.5	0.8	0.5	0.30	0.30	0.30	0.50	0.00	0.50	0.132	-0.109	0.180
1000	20	0	0.5	0.8	0.5	0.30	0.30	0.30	0.50	0.25	0.00	0.148	-0.125	0.149
1000	20	0	0.5	0.8	0.5	0.30	0.30	0.30	0.50	0.25	0.25	0.148	-0.126	0.149
1000	20	0	0.5	0.8	0.5	0.30	0.30	0.30	0.50	0.25	0.50	0.148	-0.126	0.150
1000	20	0	0.5	0.8	0.5	0.30	0.30	0.30	0.50	0.50	0.00	0.162	-0.141	0.113
1000	20	0	0.5	0.8	0.5	0.30	0.30	0.30	0.50	0.50	0.25	0.163	-0.140	0.113
1000	20	0	0.5	0.8	0.5	0.30	0.30	0.30	0.50	0.50	0.50	0.163	-0.141	0.113
1000	20	0	0.8	0.2	0.5	0.00	0.00	0.00	0.00	0.00	0.00	0.000	0.000	0.000
1000	20	0	0.8	0.2	0.5	0.15	0.15	0.15	0.00	0.00	0.00	0.016	-0.004	0.045
1000	20	0	0.8	0.2	0.5	0.15	0.15	0.15	0.00	0.00	0.25	0.017	-0.004	0.045
1000	20	0	0.8	0.2	0.5	0.15	0.15	0.15	0.00	0.00	0.50	0.017	-0.004	0.045

(continued)

												Mean Bias		
N	m_1	$\frac{m_2}{m_1}$	$E(C)$	$E(R)$	$E(U)$	σ_C	σ_R	σ_U	ρ_{CR}	ρ_{CU}	ρ_{RU}	c	r	u
1000	20	0	0.8	0.2	0.5	0.15	0.15	0.15	0.00	0.25	0.00	0.018	-0.005	0.019
1000	20	0	0.8	0.2	0.5	0.15	0.15	0.15	0.00	0.25	0.25	0.018	-0.005	0.020
1000	20	0	0.8	0.2	0.5	0.15	0.15	0.15	0.00	0.25	0.50	0.018	-0.004	0.020
1000	20	0	0.8	0.2	0.5	0.15	0.15	0.15	0.00	0.50	0.00	0.019	-0.005	-0.006
1000	20	0	0.8	0.2	0.5	0.15	0.15	0.15	0.00	0.50	0.25	0.019	-0.005	-0.007
1000	20	0	0.8	0.2	0.5	0.15	0.15	0.15	0.00	0.50	0.50	0.019	-0.005	-0.006
1000	20	0	0.8	0.2	0.5	0.15	0.15	0.15	0.25	0.00	0.00	0.017	0.002	0.045
1000	20	0	0.8	0.2	0.5	0.15	0.15	0.15	0.25	0.00	0.25	0.017	0.002	0.045
1000	20	0	0.8	0.2	0.5	0.15	0.15	0.15	0.25	0.00	0.50	0.017	0.002	0.044
1000	20	0	0.8	0.2	0.5	0.15	0.15	0.15	0.25	0.25	0.00	0.018	0.002	0.020
1000	20	0	0.8	0.2	0.5	0.15	0.15	0.15	0.25	0.25	0.25	0.018	0.002	0.020
1000	20	0	0.8	0.2	0.5	0.15	0.15	0.15	0.25	0.25	0.50	0.018	0.002	0.020
1000	20	0	0.8	0.2	0.5	0.15	0.15	0.15	0.25	0.50	0.00	0.019	0.001	-0.006
1000	20	0	0.8	0.2	0.5	0.15	0.15	0.15	0.25	0.50	0.25	0.019	0.001	-0.006
1000	20	0	0.8	0.2	0.5	0.15	0.15	0.15	0.25	0.50	0.50	0.019	0.001	-0.006
1000	20	0	0.8	0.2	0.5	0.15	0.15	0.15	0.50	0.00	0.00	0.017	0.008	0.045
1000	20	0	0.8	0.2	0.5	0.15	0.15	0.15	0.50	0.00	0.25	0.017	0.008	0.045
1000	20	0	0.8	0.2	0.5	0.15	0.15	0.15	0.50	0.00	0.50	0.016	0.008	0.045
1000	20	0	0.8	0.2	0.5	0.15	0.15	0.15	0.50	0.25	0.00	0.018	0.008	0.020
1000	20	0	0.8	0.2	0.5	0.15	0.15	0.15	0.50	0.25	0.25	0.018	0.008	0.020
1000	20	0	0.8	0.2	0.5	0.15	0.15	0.15	0.50	0.25	0.50	0.018	0.007	0.020
1000	20	0	0.8	0.2	0.5	0.15	0.15	0.15	0.50	0.50	0.00	0.019	0.007	-0.007
1000	20	0	0.8	0.2	0.5	0.15	0.15	0.15	0.50	0.50	0.25	0.020	0.007	-0.006
1000	20	0	0.8	0.2	0.5	0.15	0.15	0.15	0.50	0.50	0.50	0.019	0.007	-0.007
1000	20	0	0.8	0.2	0.5	0.30	0.30	0.30	0.00	0.00	0.00	0.053	-0.012	0.179
1000	20	0	0.8	0.2	0.5	0.30	0.30	0.30	0.00	0.00	0.25	0.053	-0.013	0.180
1000	20	0	0.8	0.2	0.5	0.30	0.30	0.30	0.00	0.00	0.50	0.053	-0.012	0.179
1000	20	0	0.8	0.2	0.5	0.30	0.30	0.30	0.00	0.25	0.00	0.067	-0.016	0.114
1000	20	0	0.8	0.2	0.5	0.30	0.30	0.30	0.00	0.25	0.25	0.067	-0.015	0.114
1000	20	0	0.8	0.2	0.5	0.30	0.30	0.30	0.00	0.25	0.50	0.067	-0.016	0.114
1000	20	0	0.8	0.2	0.5	0.30	0.30	0.30	0.00	0.50	0.00	0.081	-0.018	0.030
1000	20	0	0.8	0.2	0.5	0.30	0.30	0.30	0.00	0.50	0.25	0.080	-0.018	0.029
1000	20	0	0.8	0.2	0.5	0.30	0.30	0.30	0.00	0.50	0.50	0.081	-0.018	0.030
1000	20	0	0.8	0.2	0.5	0.30	0.30	0.30	0.25	0.00	0.00	0.053	0.004	0.180
1000	20	0	0.8	0.2	0.5	0.30	0.30	0.30	0.25	0.00	0.25	0.053	0.004	0.181
1000	20	0	0.8	0.2	0.5	0.30	0.30	0.30	0.25	0.00	0.50	0.053	0.005	0.180
1000	20	0	0.8	0.2	0.5	0.30	0.30	0.30	0.25	0.25	0.00	0.067	0.001	0.114
1000	20	0	0.8	0.2	0.5	0.30	0.30	0.30	0.25	0.25	0.25	0.067	0.002	0.114
1000	20	0	0.8	0.2	0.5	0.30	0.30	0.30	0.25	0.25	0.50	0.067	0.001	0.114

(continued)

N	m_1	$\frac{m_2}{m_1}$	$E(C)$	$E(R)$	$E(U)$	σ_C	σ_R	σ_U	ρ_{CR}	ρ_{CU}	ρ_{RU}	Mean Bias c	r	u
1000	20	0	0.8	0.2	0.5	0.30	0.30	0.30	0.25	0.50	0.00	0.081	-0.002	0.030
1000	20	0	0.8	0.2	0.5	0.30	0.30	0.30	0.25	0.50	0.25	0.081	-0.002	0.030
1000	20	0	0.8	0.2	0.5	0.30	0.30	0.30	0.25	0.50	0.50	0.081	-0.002	0.031
1000	20	0	0.8	0.2	0.5	0.30	0.30	0.30	0.50	0.00	0.00	0.053	0.018	0.180
1000	20	0	0.8	0.2	0.5	0.30	0.30	0.30	0.50	0.00	0.25	0.053	0.019	0.179
1000	20	0	0.8	0.2	0.5	0.30	0.30	0.30	0.50	0.00	0.50	0.053	0.019	0.180
1000	20	0	0.8	0.2	0.5	0.30	0.30	0.30	0.50	0.25	0.00	0.067	0.015	0.114
1000	20	0	0.8	0.2	0.5	0.30	0.30	0.30	0.50	0.25	0.25	0.067	0.015	0.115
1000	20	0	0.8	0.2	0.5	0.30	0.30	0.30	0.50	0.25	0.50	0.068	0.015	0.115
1000	20	0	0.8	0.2	0.5	0.30	0.30	0.30	0.50	0.50	0.00	0.081	0.012	0.031
1000	20	0	0.8	0.2	0.5	0.30	0.30	0.30	0.50	0.50	0.25	0.081	0.012	0.030
1000	20	0	0.8	0.2	0.5	0.30	0.30	0.30	0.50	0.50	0.50	0.081	0.012	0.031
1000	20	0	0.8	0.5	0.5	0.00	0.00	0.00	0.00	0.00	0.00	0.000	0.000	0.000
1000	20	0	0.8	0.5	0.5	0.15	0.15	0.15	0.00	0.00	0.00	0.016	-0.010	0.045
1000	20	0	0.8	0.5	0.5	0.15	0.15	0.15	0.00	0.00	0.25	0.016	-0.010	0.044
1000	20	0	0.8	0.5	0.5	0.15	0.15	0.15	0.00	0.00	0.50	0.016	-0.010	0.045
1000	20	0	0.8	0.5	0.5	0.15	0.15	0.15	0.00	0.25	0.00	0.018	-0.011	0.020
1000	20	0	0.8	0.5	0.5	0.15	0.15	0.15	0.00	0.25	0.25	0.018	-0.011	0.020
1000	20	0	0.8	0.5	0.5	0.15	0.15	0.15	0.00	0.25	0.50	0.018	-0.011	0.020
1000	20	0	0.8	0.5	0.5	0.15	0.15	0.15	0.00	0.50	0.00	0.019	-0.012	-0.006
1000	20	0	0.8	0.5	0.5	0.15	0.15	0.15	0.00	0.50	0.25	0.019	-0.011	-0.007
1000	20	0	0.8	0.5	0.5	0.15	0.15	0.15	0.00	0.50	0.50	0.019	-0.012	-0.006
1000	20	0	0.8	0.5	0.5	0.15	0.15	0.15	0.25	0.00	0.00	0.017	-0.004	0.045
1000	20	0	0.8	0.5	0.5	0.15	0.15	0.15	0.25	0.00	0.25	0.016	-0.004	0.045
1000	20	0	0.8	0.5	0.5	0.15	0.15	0.15	0.25	0.00	0.50	0.016	-0.003	0.044
1000	20	0	0.8	0.5	0.5	0.15	0.15	0.15	0.25	0.25	0.00	0.018	-0.004	0.019
1000	20	0	0.8	0.5	0.5	0.15	0.15	0.15	0.25	0.25	0.25	0.018	-0.004	0.020
1000	20	0	0.8	0.5	0.5	0.15	0.15	0.15	0.25	0.25	0.50	0.018	-0.005	0.020
1000	20	0	0.8	0.5	0.5	0.15	0.15	0.15	0.25	0.50	0.00	0.019	-0.005	-0.006
1000	20	0	0.8	0.5	0.5	0.15	0.15	0.15	0.25	0.50	0.25	0.019	-0.005	-0.006
1000	20	0	0.8	0.5	0.5	0.15	0.15	0.15	0.25	0.50	0.50	0.019	-0.005	-0.006
1000	20	0	0.8	0.5	0.5	0.15	0.15	0.15	0.50	0.00	0.00	0.017	0.003	0.045
1000	20	0	0.8	0.5	0.5	0.15	0.15	0.15	0.50	0.00	0.25	0.016	0.003	0.045
1000	20	0	0.8	0.5	0.5	0.15	0.15	0.15	0.50	0.00	0.50	0.016	0.003	0.044
1000	20	0	0.8	0.5	0.5	0.15	0.15	0.15	0.50	0.25	0.00	0.018	0.003	0.020
1000	20	0	0.8	0.5	0.5	0.15	0.15	0.15	0.50	0.25	0.25	0.018	0.002	0.020
1000	20	0	0.8	0.5	0.5	0.15	0.15	0.15	0.50	0.25	0.50	0.018	0.002	0.019
1000	20	0	0.8	0.5	0.5	0.15	0.15	0.15	0.50	0.50	0.00	0.019	0.002	-0.007
1000	20	0	0.8	0.5	0.5	0.15	0.15	0.15	0.50	0.50	0.25	0.019	0.002	-0.006

(continued)

N	m_1	$\frac{m_2}{m_1}$	$E(C)$	$E(R)$	$E(U)$	σ_C	σ_R	σ_U	ρ_{CR}	ρ_{CU}	ρ_{RU}	Mean Bias c	Mean Bias r	Mean Bias u
1000	20	0	0.8	0.5	0.5	0.15	0.15	0.15	0.50	0.50	0.50	0.019	0.002	-0.006
1000	20	0	0.8	0.5	0.5	0.30	0.30	0.30	0.00	0.00	0.00	0.053	-0.031	0.180
1000	20	0	0.8	0.5	0.5	0.30	0.30	0.30	0.00	0.00	0.25	0.053	-0.031	0.180
1000	20	0	0.8	0.5	0.5	0.30	0.30	0.30	0.00	0.00	0.50	0.053	-0.030	0.180
1000	20	0	0.8	0.5	0.5	0.30	0.30	0.30	0.00	0.25	0.00	0.067	-0.039	0.115
1000	20	0	0.8	0.5	0.5	0.30	0.30	0.30	0.00	0.25	0.25	0.067	-0.038	0.114
1000	20	0	0.8	0.5	0.5	0.30	0.30	0.30	0.00	0.25	0.50	0.067	-0.039	0.114
1000	20	0	0.8	0.5	0.5	0.30	0.30	0.30	0.00	0.50	0.00	0.081	-0.045	0.030
1000	20	0	0.8	0.5	0.5	0.30	0.30	0.30	0.00	0.50	0.25	0.081	-0.046	0.030
1000	20	0	0.8	0.5	0.5	0.30	0.30	0.30	0.00	0.50	0.50	0.081	-0.046	0.030
1000	20	0	0.8	0.5	0.5	0.30	0.30	0.30	0.25	0.00	0.00	0.053	-0.009	0.180
1000	20	0	0.8	0.5	0.5	0.30	0.30	0.30	0.25	0.00	0.25	0.053	-0.009	0.179
1000	20	0	0.8	0.5	0.5	0.30	0.30	0.30	0.25	0.00	0.50	0.053	-0.010	0.179
1000	20	0	0.8	0.5	0.5	0.30	0.30	0.30	0.25	0.25	0.00	0.067	-0.017	0.115
1000	20	0	0.8	0.5	0.5	0.30	0.30	0.30	0.25	0.25	0.25	0.067	-0.017	0.114
1000	20	0	0.8	0.5	0.5	0.30	0.30	0.30	0.25	0.25	0.50	0.067	-0.018	0.115
1000	20	0	0.8	0.5	0.5	0.30	0.30	0.30	0.25	0.50	0.00	0.081	-0.025	0.030
1000	20	0	0.8	0.5	0.5	0.30	0.30	0.30	0.25	0.50	0.25	0.081	-0.025	0.032
1000	20	0	0.8	0.5	0.5	0.30	0.30	0.30	0.25	0.50	0.50	0.081	-0.025	0.030
1000	20	0	0.8	0.5	0.5	0.30	0.30	0.30	0.50	0.00	0.00	0.052	0.013	0.181
1000	20	0	0.8	0.5	0.5	0.30	0.30	0.30	0.50	0.00	0.25	0.053	0.012	0.179
1000	20	0	0.8	0.5	0.5	0.30	0.30	0.30	0.50	0.00	0.50	0.052	0.012	0.180
1000	20	0	0.8	0.5	0.5	0.30	0.30	0.30	0.50	0.25	0.00	0.067	0.003	0.114
1000	20	0	0.8	0.5	0.5	0.30	0.30	0.30	0.50	0.25	0.25	0.068	0.004	0.114
1000	20	0	0.8	0.5	0.5	0.30	0.30	0.30	0.50	0.25	0.50	0.067	0.004	0.114
1000	20	0	0.8	0.5	0.5	0.30	0.30	0.30	0.50	0.50	0.00	0.081	-0.004	0.031
1000	20	0	0.8	0.5	0.5	0.30	0.30	0.30	0.50	0.50	0.25	0.081	-0.004	0.031
1000	20	0	0.8	0.5	0.5	0.30	0.30	0.30	0.50	0.50	0.50	0.081	-0.004	0.030
1000	20	0	0.8	0.8	0.5	0.00	0.00	0.00	0.00	0.00	0.00	0.000	0.000	0.000
1000	20	0	0.8	0.8	0.5	0.15	0.15	0.15	0.00	0.00	0.00	0.016	-0.016	0.045
1000	20	0	0.8	0.8	0.5	0.15	0.15	0.15	0.00	0.00	0.25	0.016	-0.016	0.045
1000	20	0	0.8	0.8	0.5	0.15	0.15	0.15	0.00	0.00	0.50	0.016	-0.016	0.045
1000	20	0	0.8	0.8	0.5	0.15	0.15	0.15	0.00	0.25	0.00	0.018	-0.018	0.020
1000	20	0	0.8	0.8	0.5	0.15	0.15	0.15	0.00	0.25	0.25	0.018	-0.018	0.020
1000	20	0	0.8	0.8	0.5	0.15	0.15	0.15	0.00	0.25	0.50	0.018	-0.017	0.020
1000	20	0	0.8	0.8	0.5	0.15	0.15	0.15	0.00	0.50	0.00	0.019	-0.019	-0.006
1000	20	0	0.8	0.8	0.5	0.15	0.15	0.15	0.00	0.50	0.25	0.019	-0.018	-0.007
1000	20	0	0.8	0.8	0.5	0.15	0.15	0.15	0.00	0.50	0.50	0.019	-0.019	-0.006
1000	20	0	0.8	0.8	0.5	0.15	0.15	0.15	0.25	0.00	0.00	0.017	-0.010	0.045

(continued)

N	m_1	$\frac{m_2}{m_1}$	$E(C)$	$E(R)$	$E(U)$	σ_C	σ_R	σ_U	ρ_{CR}	ρ_{CU}	ρ_{RU}	Mean Bias c	Mean Bias r	Mean Bias u
1000	20	0	0.8	0.8	0.5	0.15	0.15	0.15	0.25	0.00	0.25	0.017	-0.010	0.045
1000	20	0	0.8	0.8	0.5	0.15	0.15	0.15	0.25	0.00	0.50	0.016	-0.009	0.044
1000	20	0	0.8	0.8	0.5	0.15	0.15	0.15	0.25	0.25	0.00	0.018	-0.011	0.020
1000	20	0	0.8	0.8	0.5	0.15	0.15	0.15	0.25	0.25	0.25	0.018	-0.011	0.020
1000	20	0	0.8	0.8	0.5	0.15	0.15	0.15	0.25	0.25	0.50	0.018	-0.011	0.020
1000	20	0	0.8	0.8	0.5	0.15	0.15	0.15	0.25	0.50	0.00	0.019	-0.013	-0.006
1000	20	0	0.8	0.8	0.5	0.15	0.15	0.15	0.25	0.50	0.25	0.019	-0.012	-0.006
1000	20	0	0.8	0.8	0.5	0.15	0.15	0.15	0.25	0.50	0.50	0.019	-0.012	-0.006
1000	20	0	0.8	0.8	0.5	0.15	0.15	0.15	0.50	0.00	0.00	0.016	-0.003	0.045
1000	20	0	0.8	0.8	0.5	0.15	0.15	0.15	0.50	0.00	0.25	0.016	-0.003	0.045
1000	20	0	0.8	0.8	0.5	0.15	0.15	0.15	0.50	0.00	0.50	0.017	-0.003	0.045
1000	20	0	0.8	0.8	0.5	0.15	0.15	0.15	0.50	0.25	0.00	0.018	-0.004	0.020
1000	20	0	0.8	0.8	0.5	0.15	0.15	0.15	0.50	0.25	0.25	0.018	-0.004	0.020
1000	20	0	0.8	0.8	0.5	0.15	0.15	0.15	0.50	0.25	0.50	0.018	-0.004	0.020
1000	20	0	0.8	0.8	0.5	0.15	0.15	0.15	0.50	0.50	0.00	0.019	-0.005	-0.006
1000	20	0	0.8	0.8	0.5	0.15	0.15	0.15	0.50	0.50	0.25	0.019	-0.005	-0.007
1000	20	0	0.8	0.8	0.5	0.15	0.15	0.15	0.50	0.50	0.50	0.019	-0.006	-0.006
1000	20	0	0.8	0.8	0.5	0.30	0.30	0.30	0.00	0.00	0.00	0.053	-0.049	0.179
1000	20	0	0.8	0.8	0.5	0.30	0.30	0.30	0.00	0.00	0.25	0.053	-0.049	0.180
1000	20	0	0.8	0.8	0.5	0.30	0.30	0.30	0.00	0.00	0.50	0.053	-0.050	0.180
1000	20	0	0.8	0.8	0.5	0.30	0.30	0.30	0.00	0.25	0.00	0.067	-0.062	0.114
1000	20	0	0.8	0.8	0.5	0.30	0.30	0.30	0.00	0.25	0.25	0.067	-0.062	0.114
1000	20	0	0.8	0.8	0.5	0.30	0.30	0.30	0.00	0.25	0.50	0.067	-0.062	0.114
1000	20	0	0.8	0.8	0.5	0.30	0.30	0.30	0.00	0.50	0.00	0.081	-0.073	0.029
1000	20	0	0.8	0.8	0.5	0.30	0.30	0.30	0.00	0.50	0.25	0.081	-0.073	0.031
1000	20	0	0.8	0.8	0.5	0.30	0.30	0.30	0.00	0.50	0.50	0.081	-0.074	0.031
1000	20	0	0.8	0.8	0.5	0.30	0.30	0.30	0.25	0.00	0.00	0.053	-0.030	0.180
1000	20	0	0.8	0.8	0.5	0.30	0.30	0.30	0.25	0.00	0.25	0.053	-0.030	0.179
1000	20	0	0.8	0.8	0.5	0.30	0.30	0.30	0.25	0.00	0.50	0.053	-0.029	0.179
1000	20	0	0.8	0.8	0.5	0.30	0.30	0.30	0.25	0.25	0.00	0.067	-0.043	0.114
1000	20	0	0.8	0.8	0.5	0.30	0.30	0.30	0.25	0.25	0.25	0.067	-0.042	0.115
1000	20	0	0.8	0.8	0.5	0.30	0.30	0.30	0.25	0.25	0.50	0.067	-0.042	0.114
1000	20	0	0.8	0.8	0.5	0.30	0.30	0.30	0.25	0.50	0.00	0.081	-0.055	0.031
1000	20	0	0.8	0.8	0.5	0.30	0.30	0.30	0.25	0.50	0.25	0.081	-0.054	0.030
1000	20	0	0.8	0.8	0.5	0.30	0.30	0.30	0.25	0.50	0.50	0.081	-0.054	0.031
1000	20	0	0.8	0.8	0.5	0.30	0.30	0.30	0.50	0.00	0.00	0.053	-0.006	0.179
1000	20	0	0.8	0.8	0.5	0.30	0.30	0.30	0.50	0.00	0.25	0.053	-0.007	0.180
1000	20	0	0.8	0.8	0.5	0.30	0.30	0.30	0.50	0.00	0.50	0.053	-0.007	0.179
1000	20	0	0.8	0.8	0.5	0.30	0.30	0.30	0.50	0.25	0.00	0.067	-0.020	0.115

(continued)

												Mean Bias		
N	m_1	$\frac{m_2}{m_1}$	$E(C)$	$E(R)$	$E(U)$	σ_C	σ_R	σ_U	ρ_{CR}	ρ_{CU}	ρ_{RU}	c	r	u
1000	20	0	0.8	0.8	0.5	0.30	0.30	0.30	0.50	0.25	0.25	0.067	-0.020	0.115
1000	20	0	0.8	0.8	0.5	0.30	0.30	0.30	0.50	0.25	0.50	0.067	-0.020	0.114
1000	20	0	0.8	0.8	0.5	0.30	0.30	0.30	0.50	0.50	0.00	0.081	-0.032	0.032
1000	20	0	0.8	0.8	0.5	0.30	0.30	0.30	0.50	0.50	0.25	0.081	-0.032	0.031
1000	20	0	0.8	0.8	0.5	0.30	0.30	0.30	0.50	0.50	0.50	0.081	-0.032	0.030
1000	20	1	0.2	0.2	0.5	0.00	0.00	0.00	0.00	0.00	0.00	0.000	0.001	0.000
1000	20	1	0.2	0.2	0.5	0.15	0.15	0.15	0.00	0.00	0.00	0.039	-0.033	0.016
1000	20	1	0.2	0.2	0.5	0.15	0.15	0.15	0.00	0.00	0.25	0.040	-0.033	0.016
1000	20	1	0.2	0.2	0.5	0.15	0.15	0.15	0.00	0.00	0.50	0.040	-0.033	0.016
1000	20	1	0.2	0.2	0.5	0.15	0.15	0.15	0.00	0.25	0.00	0.044	-0.036	0.013
1000	20	1	0.2	0.2	0.5	0.15	0.15	0.15	0.00	0.25	0.25	0.044	-0.037	0.013
1000	20	1	0.2	0.2	0.5	0.15	0.15	0.15	0.00	0.25	0.50	0.045	-0.036	0.013
1000	20	1	0.2	0.2	0.5	0.15	0.15	0.15	0.00	0.50	0.00	0.049	-0.039	0.011
1000	20	1	0.2	0.2	0.5	0.15	0.15	0.15	0.00	0.50	0.25	0.048	-0.039	0.011
1000	20	1	0.2	0.2	0.5	0.15	0.15	0.15	0.00	0.50	0.50	0.049	-0.039	0.011
1000	20	1	0.2	0.2	0.5	0.15	0.15	0.15	0.25	0.00	0.00	0.040	-0.011	0.015
1000	20	1	0.2	0.2	0.5	0.15	0.15	0.15	0.25	0.00	0.25	0.039	-0.011	0.016
1000	20	1	0.2	0.2	0.5	0.15	0.15	0.15	0.25	0.00	0.50	0.039	-0.011	0.016
1000	20	1	0.2	0.2	0.5	0.15	0.15	0.15	0.25	0.25	0.00	0.044	-0.015	0.014
1000	20	1	0.2	0.2	0.5	0.15	0.15	0.15	0.25	0.25	0.25	0.044	-0.015	0.013
1000	20	1	0.2	0.2	0.5	0.15	0.15	0.15	0.25	0.25	0.50	0.044	-0.015	0.013
1000	20	1	0.2	0.2	0.5	0.15	0.15	0.15	0.25	0.50	0.00	0.049	-0.018	0.011
1000	20	1	0.2	0.2	0.5	0.15	0.15	0.15	0.25	0.50	0.25	0.049	-0.018	0.011
1000	20	1	0.2	0.2	0.5	0.15	0.15	0.15	0.25	0.50	0.50	0.049	-0.018	0.011
1000	20	1	0.2	0.2	0.5	0.15	0.15	0.15	0.50	0.00	0.00	0.039	0.012	0.016
1000	20	1	0.2	0.2	0.5	0.15	0.15	0.15	0.50	0.00	0.25	0.040	0.011	0.016
1000	20	1	0.2	0.2	0.5	0.15	0.15	0.15	0.50	0.00	0.50	0.040	0.011	0.016
1000	20	1	0.2	0.2	0.5	0.15	0.15	0.15	0.50	0.25	0.00	0.044	0.007	0.013
1000	20	1	0.2	0.2	0.5	0.15	0.15	0.15	0.50	0.25	0.25	0.044	0.008	0.013
1000	20	1	0.2	0.2	0.5	0.15	0.15	0.15	0.50	0.25	0.50	0.045	0.008	0.013
1000	20	1	0.2	0.2	0.5	0.15	0.15	0.15	0.50	0.50	0.00	0.048	0.004	0.010
1000	20	1	0.2	0.2	0.5	0.15	0.15	0.15	0.50	0.50	0.25	0.049	0.004	0.011
1000	20	1	0.2	0.2	0.5	0.15	0.15	0.15	0.50	0.50	0.50	0.049	0.004	0.011
1000	20	1	0.2	0.2	0.5	0.30	0.30	0.30	0.00	0.00	0.00	0.149	-0.085	0.065
1000	20	1	0.2	0.2	0.5	0.30	0.30	0.30	0.00	0.00	0.25	0.149	-0.085	0.065
1000	20	1	0.2	0.2	0.5	0.30	0.30	0.30	0.00	0.00	0.50	0.148	-0.086	0.064
1000	20	1	0.2	0.2	0.5	0.30	0.30	0.30	0.00	0.25	0.00	0.165	-0.090	0.057
1000	20	1	0.2	0.2	0.5	0.30	0.30	0.30	0.00	0.25	0.25	0.164	-0.090	0.057
1000	20	1	0.2	0.2	0.5	0.30	0.30	0.30	0.00	0.25	0.50	0.165	-0.091	0.056

(continued)

												Mean Bias		
N	m_1	$\frac{m_2}{m_1}$	$E(C)$	$E(R)$	$E(U)$	σ_C	σ_R	σ_U	ρ_{CR}	ρ_{CU}	ρ_{RU}	c	r	u
1000	20	1	0.2	0.2	0.5	0.30	0.30	0.30	0.00	0.50	0.00	0.179	-0.095	0.047
1000	20	1	0.2	0.2	0.5	0.30	0.30	0.30	0.00	0.50	0.25	0.179	-0.094	0.047
1000	20	1	0.2	0.2	0.5	0.30	0.30	0.30	0.00	0.50	0.50	0.179	-0.095	0.047
1000	20	1	0.2	0.2	0.5	0.30	0.30	0.30	0.25	0.00	0.00	0.148	-0.037	0.064
1000	20	1	0.2	0.2	0.5	0.30	0.30	0.30	0.25	0.00	0.25	0.148	-0.038	0.064
1000	20	1	0.2	0.2	0.5	0.30	0.30	0.30	0.25	0.00	0.50	0.148	-0.036	0.065
1000	20	1	0.2	0.2	0.5	0.30	0.30	0.30	0.25	0.25	0.00	0.165	-0.044	0.056
1000	20	1	0.2	0.2	0.5	0.30	0.30	0.30	0.25	0.25	0.25	0.165	-0.044	0.056
1000	20	1	0.2	0.2	0.5	0.30	0.30	0.30	0.25	0.25	0.50	0.165	-0.044	0.056
1000	20	1	0.2	0.2	0.5	0.30	0.30	0.30	0.25	0.50	0.00	0.179	-0.050	0.047
1000	20	1	0.2	0.2	0.5	0.30	0.30	0.30	0.25	0.50	0.25	0.179	-0.050	0.047
1000	20	1	0.2	0.2	0.5	0.30	0.30	0.30	0.25	0.50	0.50	0.179	-0.050	0.047
1000	20	1	0.2	0.2	0.5	0.30	0.30	0.30	0.50	0.00	0.00	0.148	0.020	0.065
1000	20	1	0.2	0.2	0.5	0.30	0.30	0.30	0.50	0.00	0.25	0.148	0.020	0.064
1000	20	1	0.2	0.2	0.5	0.30	0.30	0.30	0.50	0.00	0.50	0.148	0.020	0.064
1000	20	1	0.2	0.2	0.5	0.30	0.30	0.30	0.50	0.25	0.00	0.165	0.010	0.056
1000	20	1	0.2	0.2	0.5	0.30	0.30	0.30	0.50	0.25	0.25	0.165	0.009	0.056
1000	20	1	0.2	0.2	0.5	0.30	0.30	0.30	0.50	0.25	0.50	0.165	0.010	0.057
1000	20	1	0.2	0.2	0.5	0.30	0.30	0.30	0.50	0.50	0.00	0.178	0.001	0.047
1000	20	1	0.2	0.2	0.5	0.30	0.30	0.30	0.50	0.50	0.25	0.179	0.001	0.047
1000	20	1	0.2	0.2	0.5	0.30	0.30	0.30	0.50	0.50	0.50	0.179	0.002	0.047
1000	20	1	0.2	0.5	0.5	0.00	0.00	0.00	0.00	0.00	0.00	0.000	0.001	0.000
1000	20	1	0.2	0.5	0.5	0.15	0.15	0.15	0.00	0.00	0.00	0.039	-0.082	0.016
1000	20	1	0.2	0.5	0.5	0.15	0.15	0.15	0.00	0.00	0.25	0.039	-0.083	0.016
1000	20	1	0.2	0.5	0.5	0.15	0.15	0.15	0.00	0.00	0.50	0.040	-0.083	0.016
1000	20	1	0.2	0.5	0.5	0.15	0.15	0.15	0.00	0.25	0.00	0.044	-0.090	0.014
1000	20	1	0.2	0.5	0.5	0.15	0.15	0.15	0.00	0.25	0.25	0.044	-0.091	0.013
1000	20	1	0.2	0.5	0.5	0.15	0.15	0.15	0.00	0.25	0.50	0.044	-0.090	0.013
1000	20	1	0.2	0.5	0.5	0.15	0.15	0.15	0.00	0.50	0.00	0.049	-0.098	0.011
1000	20	1	0.2	0.5	0.5	0.15	0.15	0.15	0.00	0.50	0.25	0.049	-0.097	0.011
1000	20	1	0.2	0.5	0.5	0.15	0.15	0.15	0.00	0.50	0.50	0.049	-0.098	0.011
1000	20	1	0.2	0.5	0.5	0.15	0.15	0.15	0.25	0.00	0.00	0.039	-0.060	0.015
1000	20	1	0.2	0.5	0.5	0.15	0.15	0.15	0.25	0.00	0.25	0.039	-0.060	0.016
1000	20	1	0.2	0.5	0.5	0.15	0.15	0.15	0.25	0.00	0.50	0.040	-0.060	0.016
1000	20	1	0.2	0.5	0.5	0.15	0.15	0.15	0.25	0.25	0.00	0.044	-0.069	0.013
1000	20	1	0.2	0.5	0.5	0.15	0.15	0.15	0.25	0.25	0.25	0.044	-0.068	0.013
1000	20	1	0.2	0.5	0.5	0.15	0.15	0.15	0.25	0.25	0.50	0.044	-0.069	0.014
1000	20	1	0.2	0.5	0.5	0.15	0.15	0.15	0.25	0.50	0.00	0.048	-0.076	0.011
1000	20	1	0.2	0.5	0.5	0.15	0.15	0.15	0.25	0.50	0.25	0.048	-0.076	0.011

(continued)

N	m_1	$\frac{m_2}{m_1}$	$E(C)$	$E(R)$	$E(U)$	σ_C	σ_R	σ_U	ρ_{CR}	ρ_{CU}	ρ_{RU}	Mean Bias		
												c	r	u
1000	20	1	0.2	0.5	0.5	0.15	0.15	0.15	0.25	0.50	0.50	0.049	-0.076	0.011
1000	20	1	0.2	0.5	0.5	0.15	0.15	0.15	0.50	0.00	0.00	0.040	-0.038	0.016
1000	20	1	0.2	0.5	0.5	0.15	0.15	0.15	0.50	0.00	0.25	0.040	-0.038	0.016
1000	20	1	0.2	0.5	0.5	0.15	0.15	0.15	0.50	0.00	0.50	0.039	-0.037	0.016
1000	20	1	0.2	0.5	0.5	0.15	0.15	0.15	0.50	0.25	0.00	0.044	-0.046	0.013
1000	20	1	0.2	0.5	0.5	0.15	0.15	0.15	0.50	0.25	0.25	0.044	-0.047	0.014
1000	20	1	0.2	0.5	0.5	0.15	0.15	0.15	0.50	0.25	0.50	0.044	-0.046	0.013
1000	20	1	0.2	0.5	0.5	0.15	0.15	0.15	0.50	0.50	0.00	0.049	-0.054	0.011
1000	20	1	0.2	0.5	0.5	0.15	0.15	0.15	0.50	0.50	0.25	0.049	-0.054	0.011
1000	20	1	0.2	0.5	0.5	0.15	0.15	0.15	0.50	0.50	0.50	0.049	-0.055	0.011
1000	20	1	0.2	0.5	0.5	0.30	0.30	0.30	0.00	0.00	0.00	0.149	-0.212	0.064
1000	20	1	0.2	0.5	0.5	0.30	0.30	0.30	0.00	0.00	0.25	0.148	-0.213	0.064
1000	20	1	0.2	0.5	0.5	0.30	0.30	0.30	0.00	0.00	0.50	0.148	-0.212	0.064
1000	20	1	0.2	0.5	0.5	0.30	0.30	0.30	0.00	0.25	0.00	0.164	-0.226	0.056
1000	20	1	0.2	0.5	0.5	0.30	0.30	0.30	0.00	0.25	0.25	0.165	-0.225	0.056
1000	20	1	0.2	0.5	0.5	0.30	0.30	0.30	0.00	0.25	0.50	0.164	-0.226	0.056
1000	20	1	0.2	0.5	0.5	0.30	0.30	0.30	0.00	0.50	0.00	0.179	-0.236	0.047
1000	20	1	0.2	0.5	0.5	0.30	0.30	0.30	0.00	0.50	0.25	0.179	-0.237	0.046
1000	20	1	0.2	0.5	0.5	0.30	0.30	0.30	0.00	0.50	0.50	0.179	-0.237	0.047
1000	20	1	0.2	0.5	0.5	0.30	0.30	0.30	0.25	0.00	0.00	0.148	-0.160	0.064
1000	20	1	0.2	0.5	0.5	0.30	0.30	0.30	0.25	0.00	0.25	0.148	-0.161	0.064
1000	20	1	0.2	0.5	0.5	0.30	0.30	0.30	0.25	0.00	0.50	0.148	-0.161	0.064
1000	20	1	0.2	0.5	0.5	0.30	0.30	0.30	0.25	0.25	0.00	0.164	-0.175	0.057
1000	20	1	0.2	0.5	0.5	0.30	0.30	0.30	0.25	0.25	0.25	0.165	-0.177	0.056
1000	20	1	0.2	0.5	0.5	0.30	0.30	0.30	0.25	0.25	0.50	0.165	-0.176	0.056
1000	20	1	0.2	0.5	0.5	0.30	0.30	0.30	0.25	0.50	0.00	0.178	-0.188	0.047
1000	20	1	0.2	0.5	0.5	0.30	0.30	0.30	0.25	0.50	0.25	0.179	-0.188	0.047
1000	20	1	0.2	0.5	0.5	0.30	0.30	0.30	0.25	0.50	0.50	0.179	-0.187	0.047
1000	20	1	0.2	0.5	0.5	0.30	0.30	0.30	0.50	0.00	0.00	0.149	-0.107	0.064
1000	20	1	0.2	0.5	0.5	0.30	0.30	0.30	0.50	0.00	0.25	0.148	-0.107	0.065
1000	20	1	0.2	0.5	0.5	0.30	0.30	0.30	0.50	0.00	0.50	0.148	-0.108	0.064
1000	20	1	0.2	0.5	0.5	0.30	0.30	0.30	0.50	0.25	0.00	0.165	-0.125	0.057
1000	20	1	0.2	0.5	0.5	0.30	0.30	0.30	0.50	0.25	0.25	0.165	-0.125	0.057
1000	20	1	0.2	0.5	0.5	0.30	0.30	0.30	0.50	0.25	0.50	0.164	-0.125	0.056
1000	20	1	0.2	0.5	0.5	0.30	0.30	0.30	0.50	0.50	0.00	0.179	-0.139	0.047
1000	20	1	0.2	0.5	0.5	0.30	0.30	0.30	0.50	0.50	0.25	0.179	-0.139	0.047
1000	20	1	0.2	0.5	0.5	0.30	0.30	0.30	0.50	0.50	0.50	0.179	-0.139	0.047
1000	20	1	0.2	0.8	0.5	0.00	0.00	0.00	0.00	0.00	0.00	0.000	0.002	0.000
1000	20	1	0.2	0.8	0.5	0.15	0.15	0.15	0.00	0.00	0.00	0.040	-0.132	0.016

(continued)

N	m_1	$\frac{m_2}{m_1}$	$E(C)$	$E(R)$	$E(U)$	σ_C	σ_R	σ_U	ρ_{CR}	ρ_{CU}	ρ_{RU}	Mean Bias		
												c	r	u
1000	20	1	0.2	0.8	0.5	0.15	0.15	0.15	0.00	0.00	0.25	0.040	-0.133	0.016
1000	20	1	0.2	0.8	0.5	0.15	0.15	0.15	0.00	0.00	0.50	0.039	-0.133	0.016
1000	20	1	0.2	0.8	0.5	0.15	0.15	0.15	0.00	0.25	0.00	0.045	-0.145	0.013
1000	20	1	0.2	0.8	0.5	0.15	0.15	0.15	0.00	0.25	0.25	0.044	-0.144	0.013
1000	20	1	0.2	0.8	0.5	0.15	0.15	0.15	0.00	0.25	0.50	0.044	-0.145	0.013
1000	20	1	0.2	0.8	0.5	0.15	0.15	0.15	0.00	0.50	0.00	0.048	-0.156	0.011
1000	20	1	0.2	0.8	0.5	0.15	0.15	0.15	0.00	0.50	0.25	0.049	-0.156	0.011
1000	20	1	0.2	0.8	0.5	0.15	0.15	0.15	0.00	0.50	0.50	0.049	-0.157	0.011
1000	20	1	0.2	0.8	0.5	0.15	0.15	0.15	0.25	0.00	0.00	0.040	-0.112	0.016
1000	20	1	0.2	0.8	0.5	0.15	0.15	0.15	0.25	0.00	0.25	0.039	-0.111	0.016
1000	20	1	0.2	0.8	0.5	0.15	0.15	0.15	0.25	0.00	0.50	0.039	-0.112	0.016
1000	20	1	0.2	0.8	0.5	0.15	0.15	0.15	0.25	0.25	0.00	0.044	-0.124	0.013
1000	20	1	0.2	0.8	0.5	0.15	0.15	0.15	0.25	0.25	0.25	0.044	-0.124	0.013
1000	20	1	0.2	0.8	0.5	0.15	0.15	0.15	0.25	0.25	0.50	0.044	-0.124	0.013
1000	20	1	0.2	0.8	0.5	0.15	0.15	0.15	0.25	0.50	0.00	0.048	-0.136	0.011
1000	20	1	0.2	0.8	0.5	0.15	0.15	0.15	0.25	0.50	0.25	0.049	-0.136	0.011
1000	20	1	0.2	0.8	0.5	0.15	0.15	0.15	0.25	0.50	0.50	0.049	-0.136	0.010
1000	20	1	0.2	0.8	0.5	0.15	0.15	0.15	0.50	0.00	0.00	0.040	-0.091	0.015
1000	20	1	0.2	0.8	0.5	0.15	0.15	0.15	0.50	0.00	0.25	0.040	-0.091	0.016
1000	20	1	0.2	0.8	0.5	0.15	0.15	0.15	0.50	0.00	0.50	0.039	-0.091	0.016
1000	20	1	0.2	0.8	0.5	0.15	0.15	0.15	0.50	0.25	0.00	0.044	-0.105	0.013
1000	20	1	0.2	0.8	0.5	0.15	0.15	0.15	0.50	0.25	0.25	0.044	-0.105	0.013
1000	20	1	0.2	0.8	0.5	0.15	0.15	0.15	0.50	0.25	0.50	0.044	-0.105	0.014
1000	20	1	0.2	0.8	0.5	0.15	0.15	0.15	0.50	0.50	0.00	0.049	-0.117	0.011
1000	20	1	0.2	0.8	0.5	0.15	0.15	0.15	0.50	0.50	0.25	0.048	-0.118	0.011
1000	20	1	0.2	0.8	0.5	0.15	0.15	0.15	0.50	0.50	0.50	0.049	-0.117	0.011
1000	20	1	0.2	0.8	0.5	0.30	0.30	0.30	0.00	0.00	0.00	0.147	-0.341	0.065
1000	20	1	0.2	0.8	0.5	0.30	0.30	0.30	0.00	0.00	0.25	0.148	-0.341	0.064
1000	20	1	0.2	0.8	0.5	0.30	0.30	0.30	0.00	0.00	0.50	0.148	-0.340	0.065
1000	20	1	0.2	0.8	0.5	0.30	0.30	0.30	0.00	0.25	0.00	0.164	-0.361	0.056
1000	20	1	0.2	0.8	0.5	0.30	0.30	0.30	0.00	0.25	0.25	0.164	-0.361	0.056
1000	20	1	0.2	0.8	0.5	0.30	0.30	0.30	0.00	0.25	0.50	0.165	-0.361	0.057
1000	20	1	0.2	0.8	0.5	0.30	0.30	0.30	0.00	0.50	0.00	0.179	-0.378	0.047
1000	20	1	0.2	0.8	0.5	0.30	0.30	0.30	0.00	0.50	0.25	0.179	-0.377	0.047
1000	20	1	0.2	0.8	0.5	0.30	0.30	0.30	0.00	0.50	0.50	0.178	-0.378	0.047
1000	20	1	0.2	0.8	0.5	0.30	0.30	0.30	0.25	0.00	0.00	0.148	-0.299	0.064
1000	20	1	0.2	0.8	0.5	0.30	0.30	0.30	0.25	0.00	0.25	0.148	-0.299	0.064
1000	20	1	0.2	0.8	0.5	0.30	0.30	0.30	0.25	0.00	0.50	0.148	-0.298	0.065
1000	20	1	0.2	0.8	0.5	0.30	0.30	0.30	0.25	0.25	0.00	0.165	-0.321	0.057

(continued)

												Mean Bias		
N	m_1	$\frac{m_2}{m_1}$	$E(C)$	$E(R)$	$E(U)$	σ_C	σ_R	σ_U	ρ_{CR}	ρ_{CU}	ρ_{RU}	c	r	u
1000	20	1	0.2	0.8	0.5	0.30	0.30	0.30	0.25	0.25	0.25	0.165	-0.321	0.057
1000	20	1	0.2	0.8	0.5	0.30	0.30	0.30	0.25	0.25	0.50	0.164	-0.321	0.057
1000	20	1	0.2	0.8	0.5	0.30	0.30	0.30	0.25	0.50	0.00	0.179	-0.339	0.047
1000	20	1	0.2	0.8	0.5	0.30	0.30	0.30	0.25	0.50	0.25	0.179	-0.340	0.047
1000	20	1	0.2	0.8	0.5	0.30	0.30	0.30	0.25	0.50	0.50	0.179	-0.339	0.047
1000	20	1	0.2	0.8	0.5	0.30	0.30	0.30	0.50	0.00	0.00	0.148	-0.265	0.064
1000	20	1	0.2	0.8	0.5	0.30	0.30	0.30	0.50	0.00	0.25	0.148	-0.263	0.064
1000	20	1	0.2	0.8	0.5	0.30	0.30	0.30	0.50	0.00	0.50	0.148	-0.264	0.064
1000	20	1	0.2	0.8	0.5	0.30	0.30	0.30	0.50	0.25	0.00	0.165	-0.286	0.057
1000	20	1	0.2	0.8	0.5	0.30	0.30	0.30	0.50	0.25	0.25	0.165	-0.289	0.056
1000	20	1	0.2	0.8	0.5	0.30	0.30	0.30	0.50	0.25	0.50	0.165	-0.288	0.057
1000	20	1	0.2	0.8	0.5	0.30	0.30	0.30	0.50	0.50	0.00	0.179	-0.307	0.046
1000	20	1	0.2	0.8	0.5	0.30	0.30	0.30	0.50	0.50	0.25	0.179	-0.307	0.047
1000	20	1	0.2	0.8	0.5	0.30	0.30	0.30	0.50	0.50	0.50	0.179	-0.307	0.047
1000	20	1	0.5	0.2	0.5	0.00	0.00	0.00	0.00	0.00	0.00	0.000	0.000	0.000
1000	20	1	0.5	0.2	0.5	0.15	0.15	0.15	0.00	0.00	0.00	0.023	-0.008	0.011
1000	20	1	0.5	0.2	0.5	0.15	0.15	0.15	0.00	0.00	0.25	0.022	-0.008	0.011
1000	20	1	0.5	0.2	0.5	0.15	0.15	0.15	0.00	0.00	0.50	0.022	-0.009	0.011
1000	20	1	0.5	0.2	0.5	0.15	0.15	0.15	0.00	0.25	0.00	0.028	-0.011	0.009
1000	20	1	0.5	0.2	0.5	0.15	0.15	0.15	0.00	0.25	0.25	0.028	-0.011	0.008
1000	20	1	0.5	0.2	0.5	0.15	0.15	0.15	0.00	0.25	0.50	0.028	-0.011	0.008
1000	20	1	0.5	0.2	0.5	0.15	0.15	0.15	0.00	0.50	0.00	0.034	-0.013	0.006
1000	20	1	0.5	0.2	0.5	0.15	0.15	0.15	0.00	0.50	0.25	0.034	-0.013	0.006
1000	20	1	0.5	0.2	0.5	0.15	0.15	0.15	0.00	0.50	0.50	0.034	-0.013	0.006
1000	20	1	0.5	0.2	0.5	0.15	0.15	0.15	0.25	0.00	0.00	0.022	0.002	0.011
1000	20	1	0.5	0.2	0.5	0.15	0.15	0.15	0.25	0.00	0.25	0.022	0.002	0.011
1000	20	1	0.5	0.2	0.5	0.15	0.15	0.15	0.25	0.00	0.50	0.023	0.002	0.012
1000	20	1	0.5	0.2	0.5	0.15	0.15	0.15	0.25	0.25	0.00	0.028	0.000	0.009
1000	20	1	0.5	0.2	0.5	0.15	0.15	0.15	0.25	0.25	0.25	0.028	-0.001	0.009
1000	20	1	0.5	0.2	0.5	0.15	0.15	0.15	0.25	0.25	0.50	0.027	-0.001	0.008
1000	20	1	0.5	0.2	0.5	0.15	0.15	0.15	0.25	0.50	0.00	0.034	-0.003	0.006
1000	20	1	0.5	0.2	0.5	0.15	0.15	0.15	0.25	0.50	0.25	0.034	-0.003	0.006
1000	20	1	0.5	0.2	0.5	0.15	0.15	0.15	0.25	0.50	0.50	0.034	-0.002	0.006
1000	20	1	0.5	0.2	0.5	0.15	0.15	0.15	0.50	0.00	0.00	0.022	0.012	0.011
1000	20	1	0.5	0.2	0.5	0.15	0.15	0.15	0.50	0.00	0.25	0.022	0.012	0.011
1000	20	1	0.5	0.2	0.5	0.15	0.15	0.15	0.50	0.00	0.50	0.022	0.012	0.011
1000	20	1	0.5	0.2	0.5	0.15	0.15	0.15	0.50	0.25	0.00	0.028	0.010	0.009
1000	20	1	0.5	0.2	0.5	0.15	0.15	0.15	0.50	0.25	0.25	0.028	0.010	0.008
1000	20	1	0.5	0.2	0.5	0.15	0.15	0.15	0.50	0.25	0.50	0.028	0.010	0.009

(continued)

N	m_1	$\frac{m_2}{m_1}$	$E(C)$	$E(R)$	$E(U)$	σ_C	σ_R	σ_U	ρ_{CR}	ρ_{CU}	ρ_{RU}	Mean Bias c	Mean Bias r	Mean Bias u
1000	20	1	0.5	0.2	0.5	0.15	0.15	0.15	0.50	0.50	0.00	0.034	0.008	0.006
1000	20	1	0.5	0.2	0.5	0.15	0.15	0.15	0.50	0.50	0.25	0.033	0.007	0.005
1000	20	1	0.5	0.2	0.5	0.15	0.15	0.15	0.50	0.50	0.50	0.033	0.007	0.006
1000	20	1	0.5	0.2	0.5	0.30	0.30	0.30	0.00	0.00	0.00	0.084	-0.028	0.046
1000	20	1	0.5	0.2	0.5	0.30	0.30	0.30	0.00	0.00	0.25	0.084	-0.028	0.046
1000	20	1	0.5	0.2	0.5	0.30	0.30	0.30	0.00	0.00	0.50	0.084	-0.028	0.046
1000	20	1	0.5	0.2	0.5	0.30	0.30	0.30	0.00	0.25	0.00	0.107	-0.034	0.036
1000	20	1	0.5	0.2	0.5	0.30	0.30	0.30	0.00	0.25	0.25	0.106	-0.036	0.036
1000	20	1	0.5	0.2	0.5	0.30	0.30	0.30	0.00	0.25	0.50	0.107	-0.035	0.036
1000	20	1	0.5	0.2	0.5	0.30	0.30	0.30	0.00	0.50	0.00	0.129	-0.041	0.025
1000	20	1	0.5	0.2	0.5	0.30	0.30	0.30	0.00	0.50	0.25	0.129	-0.042	0.025
1000	20	1	0.5	0.2	0.5	0.30	0.30	0.30	0.00	0.50	0.50	0.130	-0.041	0.024
1000	20	1	0.5	0.2	0.5	0.30	0.30	0.30	0.25	0.00	0.00	0.084	0.003	0.046
1000	20	1	0.5	0.2	0.5	0.30	0.30	0.30	0.25	0.00	0.25	0.085	0.003	0.046
1000	20	1	0.5	0.2	0.5	0.30	0.30	0.30	0.25	0.00	0.50	0.085	0.002	0.045
1000	20	1	0.5	0.2	0.5	0.30	0.30	0.30	0.25	0.25	0.00	0.106	-0.005	0.036
1000	20	1	0.5	0.2	0.5	0.30	0.30	0.30	0.25	0.25	0.25	0.106	-0.005	0.036
1000	20	1	0.5	0.2	0.5	0.30	0.30	0.30	0.25	0.25	0.50	0.107	-0.005	0.036
1000	20	1	0.5	0.2	0.5	0.30	0.30	0.30	0.25	0.50	0.00	0.130	-0.011	0.025
1000	20	1	0.5	0.2	0.5	0.30	0.30	0.30	0.25	0.50	0.25	0.129	-0.012	0.025
1000	20	1	0.5	0.2	0.5	0.30	0.30	0.30	0.25	0.50	0.50	0.129	-0.012	0.025
1000	20	1	0.5	0.2	0.5	0.30	0.30	0.30	0.50	0.00	0.00	0.084	0.034	0.046
1000	20	1	0.5	0.2	0.5	0.30	0.30	0.30	0.50	0.00	0.25	0.084	0.035	0.046
1000	20	1	0.5	0.2	0.5	0.30	0.30	0.30	0.50	0.00	0.50	0.084	0.033	0.046
1000	20	1	0.5	0.2	0.5	0.30	0.30	0.30	0.50	0.25	0.00	0.107	0.026	0.036
1000	20	1	0.5	0.2	0.5	0.30	0.30	0.30	0.50	0.25	0.25	0.107	0.026	0.036
1000	20	1	0.5	0.2	0.5	0.30	0.30	0.30	0.50	0.25	0.50	0.106	0.025	0.036
1000	20	1	0.5	0.2	0.5	0.30	0.30	0.30	0.50	0.50	0.00	0.130	0.018	0.025
1000	20	1	0.5	0.2	0.5	0.30	0.30	0.30	0.50	0.50	0.25	0.130	0.017	0.025
1000	20	1	0.5	0.2	0.5	0.30	0.30	0.30	0.50	0.50	0.50	0.129	0.018	0.025
1000	20	1	0.5	0.5	0.5	0.00	0.00	0.00	0.00	0.00	0.00	0.000	0.000	0.000
1000	20	1	0.5	0.5	0.5	0.15	0.15	0.15	0.00	0.00	0.00	0.022	-0.021	0.011
1000	20	1	0.5	0.5	0.5	0.15	0.15	0.15	0.00	0.00	0.25	0.022	-0.021	0.011
1000	20	1	0.5	0.5	0.5	0.15	0.15	0.15	0.00	0.00	0.50	0.022	-0.021	0.011
1000	20	1	0.5	0.5	0.5	0.15	0.15	0.15	0.00	0.25	0.00	0.028	-0.026	0.008
1000	20	1	0.5	0.5	0.5	0.15	0.15	0.15	0.00	0.25	0.25	0.028	-0.026	0.009
1000	20	1	0.5	0.5	0.5	0.15	0.15	0.15	0.00	0.25	0.50	0.028	-0.026	0.008
1000	20	1	0.5	0.5	0.5	0.15	0.15	0.15	0.00	0.50	0.00	0.034	-0.031	0.006
1000	20	1	0.5	0.5	0.5	0.15	0.15	0.15	0.00	0.50	0.25	0.034	-0.031	0.006

(continued)

N	m_1	$\frac{m_2}{m_1}$	$E(C)$	$E(R)$	$E(U)$	σ_C	σ_R	σ_U	ρ_{CR}	ρ_{CU}	ρ_{RU}	Mean Bias c	Mean Bias r	Mean Bias u
1000	20	1	0.5	0.5	0.5	0.15	0.15	0.15	0.00	0.50	0.50	0.033	-0.031	0.006
1000	20	1	0.5	0.5	0.5	0.15	0.15	0.15	0.25	0.00	0.00	0.022	-0.011	0.011
1000	20	1	0.5	0.5	0.5	0.15	0.15	0.15	0.25	0.00	0.25	0.022	-0.011	0.011
1000	20	1	0.5	0.5	0.5	0.15	0.15	0.15	0.25	0.00	0.50	0.022	-0.011	0.011
1000	20	1	0.5	0.5	0.5	0.15	0.15	0.15	0.25	0.25	0.00	0.028	-0.016	0.009
1000	20	1	0.5	0.5	0.5	0.15	0.15	0.15	0.25	0.25	0.25	0.028	-0.016	0.009
1000	20	1	0.5	0.5	0.5	0.15	0.15	0.15	0.25	0.25	0.50	0.028	-0.015	0.009
1000	20	1	0.5	0.5	0.5	0.15	0.15	0.15	0.25	0.50	0.00	0.034	-0.021	0.005
1000	20	1	0.5	0.5	0.5	0.15	0.15	0.15	0.25	0.50	0.25	0.033	-0.021	0.006
1000	20	1	0.5	0.5	0.5	0.15	0.15	0.15	0.25	0.50	0.50	0.034	-0.021	0.006
1000	20	1	0.5	0.5	0.5	0.15	0.15	0.15	0.50	0.00	0.00	0.022	0.000	0.011
1000	20	1	0.5	0.5	0.5	0.15	0.15	0.15	0.50	0.00	0.25	0.022	0.001	0.011
1000	20	1	0.5	0.5	0.5	0.15	0.15	0.15	0.50	0.00	0.50	0.022	0.000	0.011
1000	20	1	0.5	0.5	0.5	0.15	0.15	0.15	0.50	0.25	0.00	0.028	-0.005	0.009
1000	20	1	0.5	0.5	0.5	0.15	0.15	0.15	0.50	0.25	0.25	0.028	-0.005	0.008
1000	20	1	0.5	0.5	0.5	0.15	0.15	0.15	0.50	0.25	0.50	0.028	-0.005	0.009
1000	20	1	0.5	0.5	0.5	0.15	0.15	0.15	0.50	0.50	0.00	0.034	-0.010	0.006
1000	20	1	0.5	0.5	0.5	0.15	0.15	0.15	0.50	0.50	0.25	0.033	-0.010	0.005
1000	20	1	0.5	0.5	0.5	0.15	0.15	0.15	0.50	0.50	0.50	0.034	-0.010	0.006
1000	20	1	0.5	0.5	0.5	0.30	0.30	0.30	0.00	0.00	0.00	0.084	-0.072	0.046
1000	20	1	0.5	0.5	0.5	0.30	0.30	0.30	0.00	0.00	0.25	0.084	-0.072	0.046
1000	20	1	0.5	0.5	0.5	0.30	0.30	0.30	0.00	0.00	0.50	0.084	-0.072	0.046
1000	20	1	0.5	0.5	0.5	0.30	0.30	0.30	0.00	0.25	0.00	0.106	-0.088	0.036
1000	20	1	0.5	0.5	0.5	0.30	0.30	0.30	0.00	0.25	0.25	0.107	-0.088	0.035
1000	20	1	0.5	0.5	0.5	0.30	0.30	0.30	0.00	0.25	0.50	0.106	-0.088	0.035
1000	20	1	0.5	0.5	0.5	0.30	0.30	0.30	0.00	0.50	0.00	0.129	-0.103	0.025
1000	20	1	0.5	0.5	0.5	0.30	0.30	0.30	0.00	0.50	0.25	0.130	-0.102	0.025
1000	20	1	0.5	0.5	0.5	0.30	0.30	0.30	0.00	0.50	0.50	0.130	-0.103	0.024
1000	20	1	0.5	0.5	0.5	0.30	0.30	0.30	0.25	0.00	0.00	0.084	-0.036	0.046
1000	20	1	0.5	0.5	0.5	0.30	0.30	0.30	0.25	0.00	0.25	0.085	-0.035	0.046
1000	20	1	0.5	0.5	0.5	0.30	0.30	0.30	0.25	0.00	0.50	0.084	-0.036	0.046
1000	20	1	0.5	0.5	0.5	0.30	0.30	0.30	0.25	0.25	0.00	0.106	-0.053	0.036
1000	20	1	0.5	0.5	0.5	0.30	0.30	0.30	0.25	0.25	0.25	0.106	-0.052	0.035
1000	20	1	0.5	0.5	0.5	0.30	0.30	0.30	0.25	0.25	0.50	0.106	-0.053	0.035
1000	20	1	0.5	0.5	0.5	0.30	0.30	0.30	0.25	0.50	0.00	0.129	-0.069	0.025
1000	20	1	0.5	0.5	0.5	0.30	0.30	0.30	0.25	0.50	0.25	0.129	-0.069	0.025
1000	20	1	0.5	0.5	0.5	0.30	0.30	0.30	0.25	0.50	0.50	0.129	-0.069	0.025
1000	20	1	0.5	0.5	0.5	0.30	0.30	0.30	0.50	0.00	0.00	0.084	0.002	0.046
1000	20	1	0.5	0.5	0.5	0.30	0.30	0.30	0.50	0.00	0.25	0.084	0.002	0.046

(continued)

												Mean Bias		
N	m_1	$\frac{m_2}{m_1}$	$E(C)$	$E(R)$	$E(U)$	σ_C	σ_R	σ_U	ρ_{CR}	ρ_{CU}	ρ_{RU}	c	r	u
1000	20	1	0.5	0.5	0.5	0.30	0.30	0.30	0.50	0.00	0.50	0.084	0.002	0.046
1000	20	1	0.5	0.5	0.5	0.30	0.30	0.30	0.50	0.25	0.00	0.106	-0.017	0.036
1000	20	1	0.5	0.5	0.5	0.30	0.30	0.30	0.50	0.25	0.25	0.106	-0.017	0.035
1000	20	1	0.5	0.5	0.5	0.30	0.30	0.30	0.50	0.25	0.50	0.106	-0.017	0.035
1000	20	1	0.5	0.5	0.5	0.30	0.30	0.30	0.50	0.50	0.00	0.129	-0.034	0.025
1000	20	1	0.5	0.5	0.5	0.30	0.30	0.30	0.50	0.50	0.25	0.130	-0.035	0.025
1000	20	1	0.5	0.5	0.5	0.30	0.30	0.30	0.50	0.50	0.50	0.130	-0.034	0.025
1000	20	1	0.5	0.8	0.5	0.00	0.00	0.00	0.00	0.00	0.00	0.000	0.000	0.000
1000	20	1	0.5	0.8	0.5	0.15	0.15	0.15	0.00	0.00	0.00	0.022	-0.034	0.011
1000	20	1	0.5	0.8	0.5	0.15	0.15	0.15	0.00	0.00	0.25	0.022	-0.033	0.011
1000	20	1	0.5	0.8	0.5	0.15	0.15	0.15	0.00	0.00	0.50	0.022	-0.034	0.012
1000	20	1	0.5	0.8	0.5	0.15	0.15	0.15	0.00	0.25	0.00	0.028	-0.042	0.009
1000	20	1	0.5	0.8	0.5	0.15	0.15	0.15	0.00	0.25	0.25	0.028	-0.042	0.008
1000	20	1	0.5	0.8	0.5	0.15	0.15	0.15	0.00	0.25	0.50	0.028	-0.042	0.009
1000	20	1	0.5	0.8	0.5	0.15	0.15	0.15	0.00	0.50	0.00	0.033	-0.050	0.006
1000	20	1	0.5	0.8	0.5	0.15	0.15	0.15	0.00	0.50	0.25	0.033	-0.050	0.006
1000	20	1	0.5	0.8	0.5	0.15	0.15	0.15	0.00	0.50	0.50	0.034	-0.050	0.006
1000	20	1	0.5	0.8	0.5	0.15	0.15	0.15	0.25	0.00	0.00	0.022	-0.024	0.011
1000	20	1	0.5	0.8	0.5	0.15	0.15	0.15	0.25	0.00	0.25	0.022	-0.023	0.012
1000	20	1	0.5	0.8	0.5	0.15	0.15	0.15	0.25	0.00	0.50	0.022	-0.023	0.011
1000	20	1	0.5	0.8	0.5	0.15	0.15	0.15	0.25	0.25	0.00	0.028	-0.032	0.009
1000	20	1	0.5	0.8	0.5	0.15	0.15	0.15	0.25	0.25	0.25	0.028	-0.031	0.009
1000	20	1	0.5	0.8	0.5	0.15	0.15	0.15	0.25	0.25	0.50	0.028	-0.032	0.009
1000	20	1	0.5	0.8	0.5	0.15	0.15	0.15	0.25	0.50	0.00	0.034	-0.040	0.006
1000	20	1	0.5	0.8	0.5	0.15	0.15	0.15	0.25	0.50	0.25	0.033	-0.040	0.006
1000	20	1	0.5	0.8	0.5	0.15	0.15	0.15	0.25	0.50	0.50	0.034	-0.040	0.006
1000	20	1	0.5	0.8	0.5	0.15	0.15	0.15	0.50	0.00	0.00	0.022	-0.013	0.011
1000	20	1	0.5	0.8	0.5	0.15	0.15	0.15	0.50	0.00	0.25	0.022	-0.013	0.011
1000	20	1	0.5	0.8	0.5	0.15	0.15	0.15	0.50	0.00	0.50	0.022	-0.013	0.011
1000	20	1	0.5	0.8	0.5	0.15	0.15	0.15	0.50	0.25	0.00	0.028	-0.022	0.009
1000	20	1	0.5	0.8	0.5	0.15	0.15	0.15	0.50	0.25	0.25	0.028	-0.021	0.008
1000	20	1	0.5	0.8	0.5	0.15	0.15	0.15	0.50	0.25	0.50	0.028	-0.022	0.009
1000	20	1	0.5	0.8	0.5	0.15	0.15	0.15	0.50	0.50	0.00	0.034	-0.030	0.006
1000	20	1	0.5	0.8	0.5	0.15	0.15	0.15	0.50	0.50	0.25	0.033	-0.030	0.006
1000	20	1	0.5	0.8	0.5	0.15	0.15	0.15	0.50	0.50	0.50	0.034	-0.030	0.006
1000	20	1	0.5	0.8	0.5	0.30	0.30	0.30	0.00	0.00	0.00	0.084	-0.115	0.046
1000	20	1	0.5	0.8	0.5	0.30	0.30	0.30	0.00	0.00	0.25	0.085	-0.116	0.046
1000	20	1	0.5	0.8	0.5	0.30	0.30	0.30	0.00	0.00	0.50	0.084	-0.116	0.046
1000	20	1	0.5	0.8	0.5	0.30	0.30	0.30	0.00	0.25	0.00	0.107	-0.140	0.036

(continued)

N	m_1	$\frac{m_2}{m_1}$	$E(C)$	$E(R)$	$E(U)$	σ_C	σ_R	σ_U	ρ_{CR}	ρ_{CU}	ρ_{RU}	Mean Bias c	Mean Bias r	Mean Bias u
1000	20	1	0.5	0.8	0.5	0.30	0.30	0.30	0.00	0.25	0.25	0.106	-0.140	0.035
1000	20	1	0.5	0.8	0.5	0.30	0.30	0.30	0.00	0.25	0.50	0.107	-0.141	0.036
1000	20	1	0.5	0.8	0.5	0.30	0.30	0.30	0.00	0.50	0.00	0.129	-0.164	0.025
1000	20	1	0.5	0.8	0.5	0.30	0.30	0.30	0.00	0.50	0.25	0.130	-0.164	0.025
1000	20	1	0.5	0.8	0.5	0.30	0.30	0.30	0.00	0.50	0.50	0.129	-0.164	0.025
1000	20	1	0.5	0.8	0.5	0.30	0.30	0.30	0.25	0.00	0.00	0.084	-0.084	0.046
1000	20	1	0.5	0.8	0.5	0.30	0.30	0.30	0.25	0.00	0.25	0.085	-0.083	0.045
1000	20	1	0.5	0.8	0.5	0.30	0.30	0.30	0.25	0.00	0.50	0.084	-0.084	0.046
1000	20	1	0.5	0.8	0.5	0.30	0.30	0.30	0.25	0.25	0.00	0.107	-0.111	0.035
1000	20	1	0.5	0.8	0.5	0.30	0.30	0.30	0.25	0.25	0.25	0.107	-0.110	0.035
1000	20	1	0.5	0.8	0.5	0.30	0.30	0.30	0.25	0.25	0.50	0.106	-0.110	0.035
1000	20	1	0.5	0.8	0.5	0.30	0.30	0.30	0.25	0.50	0.00	0.129	-0.136	0.025
1000	20	1	0.5	0.8	0.5	0.30	0.30	0.30	0.25	0.50	0.25	0.130	-0.135	0.025
1000	20	1	0.5	0.8	0.5	0.30	0.30	0.30	0.25	0.50	0.50	0.129	-0.136	0.025
1000	20	1	0.5	0.8	0.5	0.30	0.30	0.30	0.50	0.00	0.00	0.085	-0.052	0.046
1000	20	1	0.5	0.8	0.5	0.30	0.30	0.30	0.50	0.00	0.25	0.084	-0.052	0.046
1000	20	1	0.5	0.8	0.5	0.30	0.30	0.30	0.50	0.00	0.50	0.084	-0.052	0.046
1000	20	1	0.5	0.8	0.5	0.30	0.30	0.30	0.50	0.25	0.00	0.106	-0.080	0.036
1000	20	1	0.5	0.8	0.5	0.30	0.30	0.30	0.50	0.25	0.25	0.106	-0.080	0.036
1000	20	1	0.5	0.8	0.5	0.30	0.30	0.30	0.50	0.25	0.50	0.107	-0.079	0.036
1000	20	1	0.5	0.8	0.5	0.30	0.30	0.30	0.50	0.50	0.00	0.129	-0.106	0.025
1000	20	1	0.5	0.8	0.5	0.30	0.30	0.30	0.50	0.50	0.25	0.129	-0.106	0.025
1000	20	1	0.5	0.8	0.5	0.30	0.30	0.30	0.50	0.50	0.50	0.129	-0.106	0.025
1000	20	1	0.8	0.2	0.5	0.00	0.00	0.00	0.00	0.00	0.00	0.000	0.000	0.000
1000	20	1	0.8	0.2	0.5	0.15	0.15	0.15	0.00	0.00	0.00	0.007	-0.002	0.005
1000	20	1	0.8	0.2	0.5	0.15	0.15	0.15	0.00	0.00	0.25	0.007	-0.002	0.005
1000	20	1	0.8	0.2	0.5	0.15	0.15	0.15	0.00	0.00	0.50	0.007	-0.002	0.005
1000	20	1	0.8	0.2	0.5	0.15	0.15	0.15	0.00	0.25	0.00	0.014	-0.004	0.003
1000	20	1	0.8	0.2	0.5	0.15	0.15	0.15	0.00	0.25	0.25	0.014	-0.003	0.002
1000	20	1	0.8	0.2	0.5	0.15	0.15	0.15	0.00	0.25	0.50	0.014	-0.004	0.002
1000	20	1	0.8	0.2	0.5	0.15	0.15	0.15	0.00	0.50	0.00	0.020	-0.005	-0.001
1000	20	1	0.8	0.2	0.5	0.15	0.15	0.15	0.00	0.50	0.25	0.021	-0.005	-0.001
1000	20	1	0.8	0.2	0.5	0.15	0.15	0.15	0.00	0.50	0.50	0.021	-0.005	0.000
1000	20	1	0.8	0.2	0.5	0.15	0.15	0.15	0.25	0.00	0.00	0.007	0.005	0.005
1000	20	1	0.8	0.2	0.5	0.15	0.15	0.15	0.25	0.00	0.25	0.007	0.004	0.005
1000	20	1	0.8	0.2	0.5	0.15	0.15	0.15	0.25	0.00	0.50	0.007	0.004	0.006
1000	20	1	0.8	0.2	0.5	0.15	0.15	0.15	0.25	0.25	0.00	0.014	0.003	0.002
1000	20	1	0.8	0.2	0.5	0.15	0.15	0.15	0.25	0.25	0.25	0.014	0.003	0.002
1000	20	1	0.8	0.2	0.5	0.15	0.15	0.15	0.25	0.25	0.50	0.014	0.003	0.002

(continued)

												Mean Bias		
N	m_1	$\frac{m_2}{m_1}$	$E(C)$	$E(R)$	$E(U)$	σ_C	σ_R	σ_U	ρ_{CR}	ρ_{CU}	ρ_{RU}	c	r	u
1000	20	1	0.8	0.2	0.5	0.15	0.15	0.15	0.25	0.50	0.00	0.020	0.001	-0.001
1000	20	1	0.8	0.2	0.5	0.15	0.15	0.15	0.25	0.50	0.25	0.021	0.001	0.000
1000	20	1	0.8	0.2	0.5	0.15	0.15	0.15	0.25	0.50	0.50	0.021	0.001	-0.001
1000	20	1	0.8	0.2	0.5	0.15	0.15	0.15	0.50	0.00	0.00	0.007	0.010	0.005
1000	20	1	0.8	0.2	0.5	0.15	0.15	0.15	0.50	0.00	0.25	0.007	0.010	0.005
1000	20	1	0.8	0.2	0.5	0.15	0.15	0.15	0.50	0.00	0.50	0.007	0.010	0.005
1000	20	1	0.8	0.2	0.5	0.15	0.15	0.15	0.50	0.25	0.00	0.014	0.009	0.002
1000	20	1	0.8	0.2	0.5	0.15	0.15	0.15	0.50	0.25	0.25	0.014	0.009	0.002
1000	20	1	0.8	0.2	0.5	0.15	0.15	0.15	0.50	0.25	0.50	0.014	0.008	0.002
1000	20	1	0.8	0.2	0.5	0.15	0.15	0.15	0.50	0.50	0.00	0.020	0.007	-0.001
1000	20	1	0.8	0.2	0.5	0.15	0.15	0.15	0.50	0.50	0.25	0.020	0.007	-0.001
1000	20	1	0.8	0.2	0.5	0.15	0.15	0.15	0.50	0.50	0.50	0.021	0.007	0.000
1000	20	1	0.8	0.2	0.5	0.30	0.30	0.30	0.00	0.00	0.00	0.029	-0.007	0.021
1000	20	1	0.8	0.2	0.5	0.30	0.30	0.30	0.00	0.00	0.25	0.028	-0.007	0.021
1000	20	1	0.8	0.2	0.5	0.30	0.30	0.30	0.00	0.00	0.50	0.029	-0.006	0.022
1000	20	1	0.8	0.2	0.5	0.30	0.30	0.30	0.00	0.25	0.00	0.052	-0.012	0.011
1000	20	1	0.8	0.2	0.5	0.30	0.30	0.30	0.00	0.25	0.25	0.051	-0.012	0.011
1000	20	1	0.8	0.2	0.5	0.30	0.30	0.30	0.00	0.25	0.50	0.051	-0.013	0.011
1000	20	1	0.8	0.2	0.5	0.30	0.30	0.30	0.00	0.50	0.00	0.076	-0.018	0.003
1000	20	1	0.8	0.2	0.5	0.30	0.30	0.30	0.00	0.50	0.25	0.077	-0.018	0.002
1000	20	1	0.8	0.2	0.5	0.30	0.30	0.30	0.00	0.50	0.50	0.077	-0.017	0.003
1000	20	1	0.8	0.2	0.5	0.30	0.30	0.30	0.25	0.00	0.00	0.029	0.011	0.021
1000	20	1	0.8	0.2	0.5	0.30	0.30	0.30	0.25	0.00	0.25	0.029	0.010	0.021
1000	20	1	0.8	0.2	0.5	0.30	0.30	0.30	0.25	0.00	0.50	0.029	0.011	0.022
1000	20	1	0.8	0.2	0.5	0.30	0.30	0.30	0.25	0.25	0.00	0.051	0.005	0.011
1000	20	1	0.8	0.2	0.5	0.30	0.30	0.30	0.25	0.25	0.25	0.052	0.005	0.012
1000	20	1	0.8	0.2	0.5	0.30	0.30	0.30	0.25	0.25	0.50	0.052	0.005	0.012
1000	20	1	0.8	0.2	0.5	0.30	0.30	0.30	0.25	0.50	0.00	0.077	-0.001	0.002
1000	20	1	0.8	0.2	0.5	0.30	0.30	0.30	0.25	0.50	0.25	0.077	0.000	0.002
1000	20	1	0.8	0.2	0.5	0.30	0.30	0.30	0.25	0.50	0.50	0.077	-0.001	0.001
1000	20	1	0.8	0.2	0.5	0.30	0.30	0.30	0.50	0.00	0.00	0.029	0.025	0.021
1000	20	1	0.8	0.2	0.5	0.30	0.30	0.30	0.50	0.00	0.25	0.029	0.026	0.022
1000	20	1	0.8	0.2	0.5	0.30	0.30	0.30	0.50	0.00	0.50	0.029	0.025	0.021
1000	20	1	0.8	0.2	0.5	0.30	0.30	0.30	0.50	0.25	0.00	0.052	0.020	0.011
1000	20	1	0.8	0.2	0.5	0.30	0.30	0.30	0.50	0.25	0.25	0.051	0.020	0.011
1000	20	1	0.8	0.2	0.5	0.30	0.30	0.30	0.50	0.25	0.50	0.052	0.020	0.012
1000	20	1	0.8	0.2	0.5	0.30	0.30	0.30	0.50	0.50	0.00	0.077	0.014	0.003
1000	20	1	0.8	0.2	0.5	0.30	0.30	0.30	0.50	0.50	0.25	0.077	0.013	0.003
1000	20	1	0.8	0.2	0.5	0.30	0.30	0.30	0.50	0.50	0.50	0.077	0.013	0.003

(continued)

N	m_1	$\frac{m_2}{m_1}$	$E(C)$	$E(R)$	$E(U)$	σ_C	σ_R	σ_U	ρ_{CR}	ρ_{CU}	ρ_{RU}	Mean Bias		
												c	r	u
1000	20	1	0.8	0.5	0.5	0.00	0.00	0.00	0.00	0.00	0.00	0.000	0.000	0.000
1000	20	1	0.8	0.5	0.5	0.15	0.15	0.15	0.00	0.00	0.00	0.007	-0.005	0.005
1000	20	1	0.8	0.5	0.5	0.15	0.15	0.15	0.00	0.00	0.25	0.008	-0.004	0.006
1000	20	1	0.8	0.5	0.5	0.15	0.15	0.15	0.00	0.00	0.50	0.007	-0.005	0.005
1000	20	1	0.8	0.5	0.5	0.15	0.15	0.15	0.00	0.25	0.00	0.014	-0.008	0.002
1000	20	1	0.8	0.5	0.5	0.15	0.15	0.15	0.00	0.25	0.25	0.014	-0.009	0.003
1000	20	1	0.8	0.5	0.5	0.15	0.15	0.15	0.00	0.25	0.50	0.014	-0.009	0.002
1000	20	1	0.8	0.5	0.5	0.15	0.15	0.15	0.00	0.50	0.00	0.021	-0.012	-0.001
1000	20	1	0.8	0.5	0.5	0.15	0.15	0.15	0.00	0.50	0.25	0.020	-0.012	-0.001
1000	20	1	0.8	0.5	0.5	0.15	0.15	0.15	0.00	0.50	0.50	0.021	-0.013	-0.001
1000	20	1	0.8	0.5	0.5	0.15	0.15	0.15	0.25	0.00	0.00	0.007	0.002	0.005
1000	20	1	0.8	0.5	0.5	0.15	0.15	0.15	0.25	0.00	0.25	0.008	0.002	0.005
1000	20	1	0.8	0.5	0.5	0.15	0.15	0.15	0.25	0.00	0.50	0.008	0.002	0.005
1000	20	1	0.8	0.5	0.5	0.15	0.15	0.15	0.25	0.25	0.00	0.014	-0.002	0.002
1000	20	1	0.8	0.5	0.5	0.15	0.15	0.15	0.25	0.25	0.25	0.014	-0.001	0.002
1000	20	1	0.8	0.5	0.5	0.15	0.15	0.15	0.25	0.25	0.50	0.014	-0.002	0.002
1000	20	1	0.8	0.5	0.5	0.15	0.15	0.15	0.25	0.50	0.00	0.021	-0.006	-0.001
1000	20	1	0.8	0.5	0.5	0.15	0.15	0.15	0.25	0.50	0.25	0.021	-0.006	-0.001
1000	20	1	0.8	0.5	0.5	0.15	0.15	0.15	0.25	0.50	0.50	0.021	-0.006	-0.001
1000	20	1	0.8	0.5	0.5	0.15	0.15	0.15	0.50	0.00	0.00	0.008	0.009	0.005
1000	20	1	0.8	0.5	0.5	0.15	0.15	0.15	0.50	0.00	0.25	0.007	0.009	0.005
1000	20	1	0.8	0.5	0.5	0.15	0.15	0.15	0.50	0.00	0.50	0.007	0.008	0.005
1000	20	1	0.8	0.5	0.5	0.15	0.15	0.15	0.50	0.25	0.00	0.014	0.004	0.002
1000	20	1	0.8	0.5	0.5	0.15	0.15	0.15	0.50	0.25	0.25	0.014	0.004	0.002
1000	20	1	0.8	0.5	0.5	0.15	0.15	0.15	0.50	0.25	0.50	0.014	0.004	0.002
1000	20	1	0.8	0.5	0.5	0.15	0.15	0.15	0.50	0.50	0.00	0.021	0.001	-0.001
1000	20	1	0.8	0.5	0.5	0.15	0.15	0.15	0.50	0.50	0.25	0.021	0.001	-0.001
1000	20	1	0.8	0.5	0.5	0.15	0.15	0.15	0.50	0.50	0.50	0.020	0.001	-0.001
1000	20	1	0.8	0.5	0.5	0.30	0.30	0.30	0.00	0.00	0.00	0.029	-0.017	0.022
1000	20	1	0.8	0.5	0.5	0.30	0.30	0.30	0.00	0.00	0.25	0.029	-0.017	0.021
1000	20	1	0.8	0.5	0.5	0.30	0.30	0.30	0.00	0.00	0.50	0.029	-0.018	0.021
1000	20	1	0.8	0.5	0.5	0.30	0.30	0.30	0.00	0.25	0.00	0.051	-0.030	0.011
1000	20	1	0.8	0.5	0.5	0.30	0.30	0.30	0.00	0.25	0.25	0.051	-0.031	0.011
1000	20	1	0.8	0.5	0.5	0.30	0.30	0.30	0.00	0.25	0.50	0.052	-0.030	0.011
1000	20	1	0.8	0.5	0.5	0.30	0.30	0.30	0.00	0.50	0.00	0.077	-0.043	0.003
1000	20	1	0.8	0.5	0.5	0.30	0.30	0.30	0.00	0.50	0.25	0.077	-0.044	0.002
1000	20	1	0.8	0.5	0.5	0.30	0.30	0.30	0.00	0.50	0.50	0.077	-0.043	0.002
1000	20	1	0.8	0.5	0.5	0.30	0.30	0.30	0.25	0.00	0.00	0.029	0.005	0.021
1000	20	1	0.8	0.5	0.5	0.30	0.30	0.30	0.25	0.00	0.25	0.029	0.005	0.021

(continued)

N	m_1	$\frac{m_2}{m_1}$	$E(C)$	$E(R)$	$E(U)$	σ_C	σ_R	σ_U	ρ_{CR}	ρ_{CU}	ρ_{RU}	Mean Bias		
												c	r	u
1000	20	1	0.8	0.5	0.5	0.30	0.30	0.30	0.25	0.00	0.50	0.029	0.004	0.021
1000	20	1	0.8	0.5	0.5	0.30	0.30	0.30	0.25	0.25	0.00	0.051	-0.009	0.011
1000	20	1	0.8	0.5	0.5	0.30	0.30	0.30	0.25	0.25	0.25	0.051	-0.008	0.012
1000	20	1	0.8	0.5	0.5	0.30	0.30	0.30	0.25	0.25	0.50	0.051	-0.009	0.011
1000	20	1	0.8	0.5	0.5	0.30	0.30	0.30	0.25	0.50	0.00	0.077	-0.022	0.002
1000	20	1	0.8	0.5	0.5	0.30	0.30	0.30	0.25	0.50	0.25	0.076	-0.023	0.002
1000	20	1	0.8	0.5	0.5	0.30	0.30	0.30	0.25	0.50	0.50	0.077	-0.023	0.002
1000	20	1	0.8	0.5	0.5	0.30	0.30	0.30	0.50	0.00	0.00	0.029	0.026	0.021
1000	20	1	0.8	0.5	0.5	0.30	0.30	0.30	0.50	0.00	0.25	0.029	0.027	0.021
1000	20	1	0.8	0.5	0.5	0.30	0.30	0.30	0.50	0.00	0.50	0.029	0.027	0.021
1000	20	1	0.8	0.5	0.5	0.30	0.30	0.30	0.50	0.25	0.00	0.051	0.013	0.011
1000	20	1	0.8	0.5	0.5	0.30	0.30	0.30	0.50	0.25	0.25	0.052	0.014	0.011
1000	20	1	0.8	0.5	0.5	0.30	0.30	0.30	0.50	0.25	0.50	0.051	0.013	0.012
1000	20	1	0.8	0.5	0.5	0.30	0.30	0.30	0.50	0.50	0.00	0.077	-0.002	0.002
1000	20	1	0.8	0.5	0.5	0.30	0.30	0.30	0.50	0.50	0.25	0.077	-0.002	0.002
1000	20	1	0.8	0.5	0.5	0.30	0.30	0.30	0.50	0.50	0.50	0.076	-0.002	0.002
1000	20	1	0.8	0.8	0.5	0.00	0.00	0.00	0.00	0.00	0.00	0.000	0.000	0.000
1000	20	1	0.8	0.8	0.5	0.15	0.15	0.15	0.00	0.00	0.00	0.007	-0.007	0.005
1000	20	1	0.8	0.8	0.5	0.15	0.15	0.15	0.00	0.00	0.25	0.007	-0.007	0.005
1000	20	1	0.8	0.8	0.5	0.15	0.15	0.15	0.00	0.00	0.50	0.007	-0.007	0.005
1000	20	1	0.8	0.8	0.5	0.15	0.15	0.15	0.00	0.25	0.00	0.014	-0.014	0.002
1000	20	1	0.8	0.8	0.5	0.15	0.15	0.15	0.00	0.25	0.25	0.014	-0.014	0.002
1000	20	1	0.8	0.8	0.5	0.15	0.15	0.15	0.00	0.25	0.50	0.014	-0.013	0.002
1000	20	1	0.8	0.8	0.5	0.15	0.15	0.15	0.00	0.50	0.00	0.021	-0.020	0.000
1000	20	1	0.8	0.8	0.5	0.15	0.15	0.15	0.00	0.50	0.25	0.021	-0.020	0.000
1000	20	1	0.8	0.8	0.5	0.15	0.15	0.15	0.00	0.50	0.50	0.021	-0.020	-0.001
1000	20	1	0.8	0.8	0.5	0.15	0.15	0.15	0.25	0.00	0.00	0.008	-0.001	0.005
1000	20	1	0.8	0.8	0.5	0.15	0.15	0.15	0.25	0.00	0.25	0.008	-0.001	0.005
1000	20	1	0.8	0.8	0.5	0.15	0.15	0.15	0.25	0.00	0.50	0.007	-0.001	0.005
1000	20	1	0.8	0.8	0.5	0.15	0.15	0.15	0.25	0.25	0.00	0.014	-0.007	0.002
1000	20	1	0.8	0.8	0.5	0.15	0.15	0.15	0.25	0.25	0.25	0.014	-0.007	0.002
1000	20	1	0.8	0.8	0.5	0.15	0.15	0.15	0.25	0.25	0.50	0.014	-0.007	0.003
1000	20	1	0.8	0.8	0.5	0.15	0.15	0.15	0.25	0.50	0.00	0.020	-0.014	-0.001
1000	20	1	0.8	0.8	0.5	0.15	0.15	0.15	0.25	0.50	0.25	0.021	-0.013	-0.001
1000	20	1	0.8	0.8	0.5	0.15	0.15	0.15	0.25	0.50	0.50	0.021	-0.013	0.000
1000	20	1	0.8	0.8	0.5	0.15	0.15	0.15	0.50	0.00	0.00	0.008	0.006	0.005
1000	20	1	0.8	0.8	0.5	0.15	0.15	0.15	0.50	0.00	0.25	0.007	0.006	0.005
1000	20	1	0.8	0.8	0.5	0.15	0.15	0.15	0.50	0.00	0.50	0.007	0.006	0.005
1000	20	1	0.8	0.8	0.5	0.15	0.15	0.15	0.50	0.25	0.00	0.014	0.000	0.002

(continued)

N	m_1	$\frac{m_2}{m_1}$	$E(C)$	$E(R)$	$E(U)$	σ_C	σ_R	σ_U	ρ_{CR}	ρ_{CU}	ρ_{RU}	Mean Bias		
												c	r	u
1000	20	1	0.8	0.8	0.5	0.15	0.15	0.15	0.50	0.25	0.25	0.014	0.000	0.002
1000	20	1	0.8	0.8	0.5	0.15	0.15	0.15	0.50	0.25	0.50	0.014	0.000	0.002
1000	20	1	0.8	0.8	0.5	0.15	0.15	0.15	0.50	0.50	0.00	0.021	-0.007	0.000
1000	20	1	0.8	0.8	0.5	0.15	0.15	0.15	0.50	0.50	0.25	0.021	-0.007	-0.001
1000	20	1	0.8	0.8	0.5	0.15	0.15	0.15	0.50	0.50	0.50	0.020	-0.007	-0.001
1000	20	1	0.8	0.8	0.5	0.30	0.30	0.30	0.00	0.00	0.00	0.029	-0.028	0.022
1000	20	1	0.8	0.8	0.5	0.30	0.30	0.30	0.00	0.00	0.25	0.029	-0.027	0.021
1000	20	1	0.8	0.8	0.5	0.30	0.30	0.30	0.00	0.00	0.50	0.029	-0.028	0.022
1000	20	1	0.8	0.8	0.5	0.30	0.30	0.30	0.00	0.25	0.00	0.051	-0.049	0.012
1000	20	1	0.8	0.8	0.5	0.30	0.30	0.30	0.00	0.25	0.25	0.052	-0.048	0.011
1000	20	1	0.8	0.8	0.5	0.30	0.30	0.30	0.00	0.25	0.50	0.052	-0.048	0.012
1000	20	1	0.8	0.8	0.5	0.30	0.30	0.30	0.00	0.50	0.00	0.077	-0.070	0.002
1000	20	1	0.8	0.8	0.5	0.30	0.30	0.30	0.00	0.50	0.25	0.077	-0.070	0.003
1000	20	1	0.8	0.8	0.5	0.30	0.30	0.30	0.00	0.50	0.50	0.077	-0.070	0.002
1000	20	1	0.8	0.8	0.5	0.30	0.30	0.30	0.25	0.00	0.00	0.029	-0.007	0.021
1000	20	1	0.8	0.8	0.5	0.30	0.30	0.30	0.25	0.00	0.25	0.029	-0.008	0.021
1000	20	1	0.8	0.8	0.5	0.30	0.30	0.30	0.25	0.00	0.50	0.028	-0.007	0.022
1000	20	1	0.8	0.8	0.5	0.30	0.30	0.30	0.25	0.25	0.00	0.052	-0.029	0.012
1000	20	1	0.8	0.8	0.5	0.30	0.30	0.30	0.25	0.25	0.25	0.052	-0.029	0.011
1000	20	1	0.8	0.8	0.5	0.30	0.30	0.30	0.25	0.25	0.50	0.051	-0.028	0.012
1000	20	1	0.8	0.8	0.5	0.30	0.30	0.30	0.25	0.50	0.00	0.077	-0.051	0.002
1000	20	1	0.8	0.8	0.5	0.30	0.30	0.30	0.25	0.50	0.25	0.076	-0.050	0.002
1000	20	1	0.8	0.8	0.5	0.30	0.30	0.30	0.25	0.50	0.50	0.077	-0.051	0.002
1000	20	1	0.8	0.8	0.5	0.30	0.30	0.30	0.50	0.00	0.00	0.028	0.016	0.021
1000	20	1	0.8	0.8	0.5	0.30	0.30	0.30	0.50	0.00	0.25	0.029	0.016	0.020
1000	20	1	0.8	0.8	0.5	0.30	0.30	0.30	0.50	0.00	0.50	0.029	0.017	0.022
1000	20	1	0.8	0.8	0.5	0.30	0.30	0.30	0.50	0.25	0.00	0.052	-0.006	0.011
1000	20	1	0.8	0.8	0.5	0.30	0.30	0.30	0.50	0.25	0.25	0.052	-0.006	0.012
1000	20	1	0.8	0.8	0.5	0.30	0.30	0.30	0.50	0.25	0.50	0.051	-0.005	0.012
1000	20	1	0.8	0.8	0.5	0.30	0.30	0.30	0.50	0.50	0.00	0.077	-0.028	0.002
1000	20	1	0.8	0.8	0.5	0.30	0.30	0.30	0.50	0.50	0.25	0.077	-0.028	0.003
1000	20	1	0.8	0.8	0.5	0.30	0.30	0.30	0.50	0.50	0.50	0.077	-0.028	0.003

Note. N = Number of simulated participants; m_1 = number of word pairs; m_2/m_1 = ratio of singletons to word pairs; $E(C), E(R), E(U)$ = expected values of model parameters; $\sigma_C, \sigma_R, \sigma_U$ = standard deviation of model parameters; $\rho_{CR}, \rho_{CU}, \rho_{RU}$ = true correlation between parameters
