# Supplementary material for: On aggregation invariance of multinomial processing tree models
Source: Behav Res Methods. 2024 Oct 14;56(8):8677–94. doi: 10.3758/s13428-024-02497-y (PMC11525265; doi:10.3758/s13428-024-02497-y)
Supplement: Supplementary file 3 — (pdf 212 KB) [file 13428_2024_2497_MOESM3_ESM.pdf]

On Aggregation Invariance of Multinomial Processing Tree Models

Supplemental Material III: Full Table of Simulation Results (4-Parameter Model)

Edgar Erdfelder, Julian Quevedo Pütter, & Martin Schnuerch

Table 1

Full table of results of the Monte Carlo simulation for the 4-parameter pair-clustering model comprising 1,000 replications per parameter combination. Results display the mean bias in aggregate estimates of the model parameters c, r, u, and a.

										Mean Bias			
N	m_1	$\frac{m_2}{m_1}$	$E(C)$, $E(R)$	$E(U)$, $E(A)$	σ_C, σ_R	σ_U, σ_A	ρ_{CR}	ρ_{CU}, ρ_{CA}	ρ_{RU}, ρ_{RA}	c	r	u	a
10	4	1	0.2	0.5	0.00	0.00	0.00	0.00	0.00	0.013	0.078	0.037	0.004
10	4	1	0.2	0.5	0.15	0.15	0.00	0.00	0.00	0.024	0.065	0.043	-0.003
10	4	1	0.2	0.5	0.15	0.15	0.00	0.00	0.25	0.018	0.074	0.041	-0.001
10	4	1	0.2	0.5	0.15	0.15	0.00	0.00	0.50	0.024	0.066	0.061	-0.001
10	4	1	0.2	0.5	0.15	0.15	0.00	0.25	0.00	0.020	0.066	0.044	0.000
10	4	1	0.2	0.5	0.15	0.15	0.00	0.25	0.25	0.023	0.060	0.040	0.003
10	4	1	0.2	0.5	0.15	0.15	0.00	0.25	0.50	0.017	0.080	0.036	0.003
10	4	1	0.2	0.5	0.15	0.15	0.00	0.50	0.00	0.023	0.065	0.032	0.001
10	4	1	0.2	0.5	0.15	0.15	0.00	0.50	0.25	0.031	0.065	0.025	0.005
10	4	1	0.2	0.5	0.15	0.15	0.00	0.50	0.50	0.024	0.069	0.038	0.002
10	4	1	0.2	0.5	0.15	0.15	0.25	0.00	0.00	0.021	0.093	0.039	0.004
10	4	1	0.2	0.5	0.15	0.15	0.25	0.00	0.25	0.019	0.105	0.029	0.006
10	4	1	0.2	0.5	0.15	0.15	0.25	0.00	0.50	0.020	0.100	0.051	0.003
10	4	1	0.2	0.5	0.15	0.15	0.25	0.25	0.00	0.020	0.109	0.029	0.006
10	4	1	0.2	0.5	0.15	0.15	0.25	0.25	0.25	0.017	0.127	0.024	0.001
10	4	1	0.2	0.5	0.15	0.15	0.25	0.25	0.50	0.023	0.104	0.034	0.007
10	4	1	0.2	0.5	0.15	0.15	0.25	0.50	0.00	0.026	0.111	0.022	0.003
10	4	1	0.2	0.5	0.15	0.15	0.25	0.50	0.25	0.015	0.093	0.017	0.001
10	4	1	0.2	0.5	0.15	0.15	0.25	0.50	0.50	0.016	0.107	0.016	0.000
10	4	1	0.2	0.5	0.15	0.15	0.50	0.00	0.00	0.019	0.126	0.048	0.003
10	4	1	0.2	0.5	0.15	0.15	0.50	0.00	0.25	0.025	0.124	0.062	0.003
10	4	1	0.2	0.5	0.15	0.15	0.50	0.00	0.50	0.012	0.134	0.033	0.006
10	4	1	0.2	0.5	0.15	0.15	0.50	0.25	0.00	0.024	0.130	0.039	-0.001
10	4	1	0.2	0.5	0.15	0.15	0.50	0.25	0.25	0.019	0.138	0.023	0.006

(continued)

N	m_1	$\frac{m_2}{m_1}$	$E(C)$, $E(R)$	$E(U)$, $E(A)$	σ_C, σ_R	σ_U, σ_A	ρ_{CR}	ρ_{CU}, ρ_{CA}	ρ_{RU}, ρ_{RA}	Mean Bias c	r	u	a
10	4	1	0.2	0.5	0.15	0.15	0.50	0.25	0.50	0.024	0.120	0.032	0.003
10	4	1	0.2	0.5	0.15	0.15	0.50	0.50	0.00	0.025	0.126	0.034	-0.002
10	4	1	0.2	0.5	0.15	0.15	0.50	0.50	0.25	0.015	0.142	0.017	0.002
10	4	1	0.2	0.5	0.15	0.15	0.50	0.50	0.50	0.023	0.117	0.019	0.002
10	4	1	0.2	0.5	0.30	0.30	0.00	0.00	0.00	0.016	0.078	0.042	0.003
10	4	1	0.2	0.5	0.30	0.30	0.00	0.00	0.25	0.019	0.054	0.040	0.000
10	4	1	0.2	0.5	0.30	0.30	0.00	0.00	0.50	0.019	0.065	0.050	0.000
10	4	1	0.2	0.5	0.30	0.30	0.00	0.25	0.00	0.027	0.047	-0.002	0.004
10	4	1	0.2	0.5	0.30	0.30	0.00	0.25	0.25	0.018	0.042	-0.004	0.006
10	4	1	0.2	0.5	0.30	0.30	0.00	0.25	0.50	0.021	0.050	-0.001	0.000
10	4	1	0.2	0.5	0.30	0.30	0.00	0.50	0.00	0.009	0.076	-0.053	0.002
10	4	1	0.2	0.5	0.30	0.30	0.00	0.50	0.25	0.020	0.083	-0.058	0.007
10	4	1	0.2	0.5	0.30	0.30	0.00	0.50	0.50	0.006	0.046	-0.050	0.000
10	4	1	0.2	0.5	0.30	0.30	0.25	0.00	0.00	0.026	0.117	0.062	0.011
10	4	1	0.2	0.5	0.30	0.30	0.25	0.00	0.25	0.025	0.142	0.056	0.002
10	4	1	0.2	0.5	0.30	0.30	0.25	0.00	0.50	0.018	0.123	0.044	-0.003
10	4	1	0.2	0.5	0.30	0.30	0.25	0.25	0.00	0.018	0.138	0.005	0.004
10	4	1	0.2	0.5	0.30	0.30	0.25	0.25	0.25	0.021	0.112	-0.003	0.008
10	4	1	0.2	0.5	0.30	0.30	0.25	0.25	0.50	0.024	0.145	-0.003	0.010
10	4	1	0.2	0.5	0.30	0.30	0.25	0.50	0.00	0.010	0.143	-0.056	0.007
10	4	1	0.2	0.5	0.30	0.30	0.25	0.50	0.25	0.022	0.150	-0.055	0.005
10	4	1	0.2	0.5	0.30	0.30	0.25	0.50	0.50	0.005	0.144	-0.059	0.004
10	4	1	0.2	0.5	0.30	0.30	0.50	0.00	0.00	0.022	0.220	0.052	0.006
10	4	1	0.2	0.5	0.30	0.30	0.50	0.00	0.25	0.024	0.208	0.053	0.005
10	4	1	0.2	0.5	0.30	0.30	0.50	0.00	0.50	0.023	0.219	0.048	0.005
10	4	1	0.2	0.5	0.30	0.30	0.50	0.25	0.00	0.032	0.219	0.015	0.004
10	4	1	0.2	0.5	0.30	0.30	0.50	0.25	0.25	0.026	0.205	0.004	0.007
10	4	1	0.2	0.5	0.30	0.30	0.50	0.25	0.50	0.028	0.216	0.008	0.007
10	4	1	0.2	0.5	0.30	0.30	0.50	0.50	0.00	0.024	0.240	-0.056	0.004
10	4	1	0.2	0.5	0.30	0.30	0.50	0.50	0.25	0.022	0.220	-0.038	-0.001
10	4	1	0.2	0.5	0.30	0.30	0.50	0.50	0.50	0.028	0.241	-0.053	0.005
10	4	1	0.5	0.5	0.00	0.00	0.00	0.00	0.00	0.009	0.019	0.054	0.004
10	4	1	0.5	0.5	0.15	0.15	0.00	0.00	0.00	0.006	0.021	0.039	0.006
10	4	1	0.5	0.5	0.15	0.15	0.00	0.00	0.25	0.009	0.025	0.044	0.005
10	4	1	0.5	0.5	0.15	0.15	0.00	0.00	0.50	0.010	0.019	0.049	0.004
10	4	1	0.5	0.5	0.15	0.15	0.00	0.25	0.00	0.008	0.012	0.028	0.005
10	4	1	0.5	0.5	0.15	0.15	0.00	0.25	0.25	0.000	0.029	0.007	0.006
10	4	1	0.5	0.5	0.15	0.15	0.00	0.25	0.50	0.004	0.032	0.011	0.002

(continued)

N	m_1	$\frac{m_2}{m_1}$	$E(C)$, $E(R)$	$E(U)$, $E(A)$	σ_C, σ_R	σ_U, σ_A	ρ_{CR}	ρ_{CU}, ρ_{CA}	ρ_{RU}, ρ_{RA}	Mean Bias			
										c	r	u	a
10	4	1	0.5	0.5	0.15	0.15	0.00	0.50	0.00	0.008	0.023	0.004	0.005
10	4	1	0.5	0.5	0.15	0.15	0.00	0.50	0.25	0.009	0.028	0.016	0.001
10	4	1	0.5	0.5	0.15	0.15	0.00	0.50	0.50	-0.003	0.040	-0.014	0.003
10	4	1	0.5	0.5	0.15	0.15	0.25	0.00	0.00	0.008	0.034	0.046	0.004
10	4	1	0.5	0.5	0.15	0.15	0.25	0.00	0.25	0.008	0.039	0.060	0.003
10	4	1	0.5	0.5	0.15	0.15	0.25	0.00	0.50	0.005	0.034	0.036	0.006
10	4	1	0.5	0.5	0.15	0.15	0.25	0.25	0.00	0.004	0.033	0.025	0.005
10	4	1	0.5	0.5	0.15	0.15	0.25	0.25	0.25	0.000	0.033	0.024	0.005
10	4	1	0.5	0.5	0.15	0.15	0.25	0.25	0.50	0.007	0.037	0.018	0.004
10	4	1	0.5	0.5	0.15	0.15	0.25	0.50	0.00	0.011	0.030	0.010	0.005
10	4	1	0.5	0.5	0.15	0.15	0.25	0.50	0.25	0.007	0.035	0.022	0.001
10	4	1	0.5	0.5	0.15	0.15	0.25	0.50	0.50	0.000	0.044	0.002	0.004
10	4	1	0.5	0.5	0.15	0.15	0.50	0.00	0.00	0.012	0.037	0.049	0.007
10	4	1	0.5	0.5	0.15	0.15	0.50	0.00	0.25	0.002	0.049	0.044	0.001
10	4	1	0.5	0.5	0.15	0.15	0.50	0.00	0.50	0.005	0.051	0.043	-0.001
10	4	1	0.5	0.5	0.15	0.15	0.50	0.25	0.00	0.004	0.044	0.011	0.006
10	4	1	0.5	0.5	0.15	0.15	0.50	0.25	0.25	0.008	0.047	0.027	0.002
10	4	1	0.5	0.5	0.15	0.15	0.50	0.25	0.50	-0.001	0.056	0.004	0.004
10	4	1	0.5	0.5	0.15	0.15	0.50	0.50	0.00	0.006	0.045	-0.007	0.005
10	4	1	0.5	0.5	0.15	0.15	0.50	0.50	0.25	0.005	0.039	0.020	0.001
10	4	1	0.5	0.5	0.15	0.15	0.50	0.50	0.50	0.003	0.056	-0.004	-0.002
10	4	1	0.5	0.5	0.30	0.30	0.00	0.00	0.00	-0.008	0.042	0.035	0.004
10	4	1	0.5	0.5	0.30	0.30	0.00	0.00	0.25	0.006	0.034	0.054	0.005
10	4	1	0.5	0.5	0.30	0.30	0.00	0.00	0.50	-0.004	0.035	0.030	0.003
10	4	1	0.5	0.5	0.30	0.30	0.00	0.25	0.00	0.007	0.029	-0.023	0.005
10	4	1	0.5	0.5	0.30	0.30	0.00	0.25	0.25	0.002	0.034	-0.033	-0.001
10	4	1	0.5	0.5	0.30	0.30	0.00	0.25	0.50	0.013	0.030	-0.028	0.004
10	4	1	0.5	0.5	0.30	0.30	0.00	0.50	0.00	0.015	0.034	-0.114	0.006
10	4	1	0.5	0.5	0.30	0.30	0.00	0.50	0.25	0.002	0.046	-0.103	-0.001
10	4	1	0.5	0.5	0.30	0.30	0.00	0.50	0.50	-0.011	0.046	-0.136	0.004
10	4	1	0.5	0.5	0.30	0.30	0.25	0.00	0.00	0.003	0.074	0.027	0.004
10	4	1	0.5	0.5	0.30	0.30	0.25	0.00	0.25	-0.008	0.086	0.018	0.004
10	4	1	0.5	0.5	0.30	0.30	0.25	0.00	0.50	0.005	0.071	0.037	0.002
10	4	1	0.5	0.5	0.30	0.30	0.25	0.25	0.00	0.009	0.077	-0.027	0.002
10	4	1	0.5	0.5	0.30	0.30	0.25	0.25	0.25	0.001	0.076	-0.030	0.001
10	4	1	0.5	0.5	0.30	0.30	0.25	0.25	0.50	0.005	0.072	-0.030	-0.001
10	4	1	0.5	0.5	0.30	0.30	0.25	0.50	0.00	0.014	0.083	-0.110	0.003
10	4	1	0.5	0.5	0.30	0.30	0.25	0.50	0.25	0.012	0.095	-0.119	0.012

(continued)

N	m_1	$\frac{m_2}{m_1}$	$E(C)$, $E(R)$	$E(U)$, $E(A)$	σ_C, σ_R	σ_U, σ_A	ρ_{CR}	ρ_{CU}, ρ_{CA}	ρ_{RU}, ρ_{RA}	Mean Bias c	r	u	a
10	4	1	0.5	0.5	0.30	0.30	0.25	0.50	0.50	0.010	0.077	-0.124	0.004
10	4	1	0.5	0.5	0.30	0.30	0.50	0.00	0.00	0.006	0.114	0.039	0.005
10	4	1	0.5	0.5	0.30	0.30	0.50	0.00	0.25	-0.002	0.123	0.025	0.008
10	4	1	0.5	0.5	0.30	0.30	0.50	0.00	0.50	-0.004	0.114	0.030	0.005
10	4	1	0.5	0.5	0.30	0.30	0.50	0.25	0.00	0.003	0.109	-0.027	-0.003
10	4	1	0.5	0.5	0.30	0.30	0.50	0.25	0.25	0.000	0.116	-0.026	0.005
10	4	1	0.5	0.5	0.30	0.30	0.50	0.25	0.50	0.010	0.109	-0.027	0.003
10	4	1	0.5	0.5	0.30	0.30	0.50	0.50	0.00	0.005	0.120	-0.126	-0.002
10	4	1	0.5	0.5	0.30	0.30	0.50	0.50	0.25	0.013	0.112	-0.109	0.004
10	4	1	0.5	0.5	0.30	0.30	0.50	0.50	0.50	0.009	0.115	-0.109	0.002
10	20	1	0.2	0.5	0.00	0.00	0.00	0.00	0.00	0.009	0.020	0.017	0.001
10	20	1	0.2	0.5	0.15	0.15	0.00	0.00	0.00	0.004	0.040	0.012	0.002
10	20	1	0.2	0.5	0.15	0.15	0.00	0.00	0.25	0.005	0.042	0.012	0.001
10	20	1	0.2	0.5	0.15	0.15	0.00	0.00	0.50	0.004	0.050	0.017	0.002
10	20	1	0.2	0.5	0.15	0.15	0.00	0.25	0.00	0.004	0.044	0.005	-0.005
10	20	1	0.2	0.5	0.15	0.15	0.00	0.25	0.25	0.002	0.043	-0.001	0.002
10	20	1	0.2	0.5	0.15	0.15	0.00	0.25	0.50	0.003	0.047	0.001	-0.002
10	20	1	0.2	0.5	0.15	0.15	0.00	0.50	0.00	0.004	0.046	-0.015	0.000
10	20	1	0.2	0.5	0.15	0.15	0.00	0.50	0.25	0.002	0.048	-0.016	0.001
10	20	1	0.2	0.5	0.15	0.15	0.00	0.50	0.50	0.001	0.037	-0.017	0.001
10	20	1	0.2	0.5	0.15	0.15	0.25	0.00	0.00	0.003	0.073	0.017	0.000
10	20	1	0.2	0.5	0.15	0.15	0.25	0.00	0.25	0.007	0.062	0.016	0.000
10	20	1	0.2	0.5	0.15	0.15	0.25	0.00	0.50	0.004	0.068	0.010	0.000
10	20	1	0.2	0.5	0.15	0.15	0.25	0.25	0.00	0.007	0.053	0.002	0.001
10	20	1	0.2	0.5	0.15	0.15	0.25	0.25	0.25	0.007	0.072	0.008	0.001
10	20	1	0.2	0.5	0.15	0.15	0.25	0.25	0.50	0.003	0.068	-0.001	0.000
10	20	1	0.2	0.5	0.15	0.15	0.25	0.50	0.00	0.005	0.074	-0.011	0.000
10	20	1	0.2	0.5	0.15	0.15	0.25	0.50	0.25	0.005	0.071	-0.019	0.002
10	20	1	0.2	0.5	0.15	0.15	0.25	0.50	0.50	0.004	0.067	-0.013	0.001
10	20	1	0.2	0.5	0.15	0.15	0.50	0.00	0.00	-0.002	0.106	0.008	-0.001
10	20	1	0.2	0.5	0.15	0.15	0.50	0.00	0.25	0.007	0.085	0.016	0.000
10	20	1	0.2	0.5	0.15	0.15	0.50	0.00	0.50	0.012	0.075	0.019	-0.001
10	20	1	0.2	0.5	0.15	0.15	0.50	0.25	0.00	0.011	0.093	0.010	-0.002
10	20	1	0.2	0.5	0.15	0.15	0.50	0.25	0.25	0.005	0.105	0.002	0.000
10	20	1	0.2	0.5	0.15	0.15	0.50	0.25	0.50	0.007	0.094	0.005	0.000
10	20	1	0.2	0.5	0.15	0.15	0.50	0.50	0.00	0.003	0.092	-0.016	-0.001
10	20	1	0.2	0.5	0.15	0.15	0.50	0.50	0.25	0.002	0.099	-0.021	0.004
10	20	1	0.2	0.5	0.15	0.15	0.50	0.50	0.50	0.001	0.097	-0.013	-0.001

(continued)

N	m_1	$\frac{m_2}{m_1}$	$E(C)$, $E(R)$	$E(U)$, $E(A)$	σ_C, σ_R	σ_U, σ_A	ρ_{CR}	ρ_{CU}, ρ_{CA}	ρ_{RU}, ρ_{RA}	Mean Bias c	Mean Bias r	Mean Bias u	Mean Bias a
10	20	1	0.2	0.5	0.30	0.30	0.00	0.00	0.00	0.015	0.070	0.030	0.005
10	20	1	0.2	0.5	0.30	0.30	0.00	0.00	0.25	0.010	0.086	0.020	0.000
10	20	1	0.2	0.5	0.30	0.30	0.00	0.00	0.50	0.013	0.072	0.033	-0.001
10	20	1	0.2	0.5	0.30	0.30	0.00	0.25	0.00	0.006	0.103	-0.015	-0.002
10	20	1	0.2	0.5	0.30	0.30	0.00	0.25	0.25	0.009	0.091	-0.025	0.001
10	20	1	0.2	0.5	0.30	0.30	0.00	0.25	0.50	0.004	0.100	-0.026	0.000
10	20	1	0.2	0.5	0.30	0.30	0.00	0.50	0.00	0.006	0.106	-0.082	0.002
10	20	1	0.2	0.5	0.30	0.30	0.00	0.50	0.25	0.002	0.107	-0.086	0.009
10	20	1	0.2	0.5	0.30	0.30	0.00	0.50	0.50	0.004	0.080	-0.078	0.000
10	20	1	0.2	0.5	0.30	0.30	0.25	0.00	0.00	0.021	0.135	0.036	0.002
10	20	1	0.2	0.5	0.30	0.30	0.25	0.00	0.25	0.019	0.129	0.040	0.003
10	20	1	0.2	0.5	0.30	0.30	0.25	0.00	0.50	0.007	0.166	0.027	0.001
10	20	1	0.2	0.5	0.30	0.30	0.25	0.25	0.00	0.016	0.165	-0.016	0.001
10	20	1	0.2	0.5	0.30	0.30	0.25	0.25	0.25	0.003	0.164	-0.018	-0.002
10	20	1	0.2	0.5	0.30	0.30	0.25	0.25	0.50	0.008	0.171	-0.016	0.002
10	20	1	0.2	0.5	0.30	0.30	0.25	0.50	0.00	0.002	0.194	-0.078	0.004
10	20	1	0.2	0.5	0.30	0.30	0.25	0.50	0.25	0.004	0.178	-0.075	0.001
10	20	1	0.2	0.5	0.30	0.30	0.25	0.50	0.50	0.001	0.175	-0.076	0.000
10	20	1	0.2	0.5	0.30	0.30	0.50	0.00	0.00	0.021	0.229	0.039	0.001
10	20	1	0.2	0.5	0.30	0.30	0.50	0.00	0.25	0.009	0.248	0.028	0.003
10	20	1	0.2	0.5	0.30	0.30	0.50	0.00	0.50	0.013	0.237	0.036	0.004
10	20	1	0.2	0.5	0.30	0.30	0.50	0.25	0.00	0.025	0.224	0.001	-0.002
10	20	1	0.2	0.5	0.30	0.30	0.50	0.25	0.25	0.017	0.244	-0.022	0.003
10	20	1	0.2	0.5	0.30	0.30	0.50	0.25	0.50	0.000	0.264	-0.024	0.007
10	20	1	0.2	0.5	0.30	0.30	0.50	0.50	0.00	0.001	0.257	-0.076	-0.001
10	20	1	0.2	0.5	0.30	0.30	0.50	0.50	0.25	0.004	0.271	-0.072	0.004
10	20	1	0.2	0.5	0.30	0.30	0.50	0.50	0.50	0.003	0.262	-0.075	-0.002
10	20	1	0.5	0.5	0.00	0.00	0.00	0.00	0.00	0.005	0.000	0.019	0.001
10	20	1	0.5	0.5	0.15	0.15	0.00	0.00	0.00	0.005	0.002	0.028	-0.002
10	20	1	0.5	0.5	0.15	0.15	0.00	0.00	0.25	0.005	0.005	0.017	0.000
10	20	1	0.5	0.5	0.15	0.15	0.00	0.00	0.50	0.001	0.008	0.019	-0.001
10	20	1	0.5	0.5	0.15	0.15	0.00	0.25	0.00	-0.002	0.000	-0.015	0.001
10	20	1	0.5	0.5	0.15	0.15	0.00	0.25	0.25	0.001	0.007	-0.010	-0.002
10	20	1	0.5	0.5	0.15	0.15	0.00	0.25	0.50	-0.002	0.007	-0.015	0.002
10	20	1	0.5	0.5	0.15	0.15	0.00	0.50	0.00	0.003	0.005	-0.024	-0.001
10	20	1	0.5	0.5	0.15	0.15	0.00	0.50	0.25	-0.001	0.005	-0.036	-0.003
10	20	1	0.5	0.5	0.15	0.15	0.00	0.50	0.50	0.004	0.004	-0.018	0.002
10	20	1	0.5	0.5	0.15	0.15	0.25	0.00	0.00	0.000	0.013	0.015	-0.001

(continued)

N	m_1	$\frac{m_2}{m_1}$	$E(C)$, $E(R)$	$E(U)$, $E(A)$	σ_C, σ_R	σ_U, σ_A	ρ_{CR}	ρ_{CU}, ρ_{CA}	ρ_{RU}, ρ_{RA}	Mean Bias			
										c	r	u	a
10	20	1	0.5	0.5	0.15	0.15	0.25	0.00	0.25	-0.001	0.018	0.011	0.000
10	20	1	0.5	0.5	0.15	0.15	0.25	0.00	0.50	0.003	0.016	0.016	0.002
10	20	1	0.5	0.5	0.15	0.15	0.25	0.25	0.00	0.000	0.017	-0.008	0.002
10	20	1	0.5	0.5	0.15	0.15	0.25	0.25	0.25	0.000	0.013	-0.002	-0.002
10	20	1	0.5	0.5	0.15	0.15	0.25	0.25	0.50	0.002	0.013	0.002	0.000
10	20	1	0.5	0.5	0.15	0.15	0.25	0.50	0.00	0.000	0.015	-0.025	-0.001
10	20	1	0.5	0.5	0.15	0.15	0.25	0.50	0.25	-0.002	0.018	-0.029	-0.002
10	20	1	0.5	0.5	0.15	0.15	0.25	0.50	0.50	0.000	0.015	-0.030	-0.001
10	20	1	0.5	0.5	0.15	0.15	0.50	0.00	0.00	0.003	0.025	0.015	0.003
10	20	1	0.5	0.5	0.15	0.15	0.50	0.00	0.25	-0.005	0.027	0.013	-0.001
10	20	1	0.5	0.5	0.15	0.15	0.50	0.00	0.50	0.001	0.029	0.014	0.000
10	20	1	0.5	0.5	0.15	0.15	0.50	0.25	0.00	0.003	0.024	-0.007	0.002
10	20	1	0.5	0.5	0.15	0.15	0.50	0.25	0.25	-0.002	0.029	-0.013	-0.001
10	20	1	0.5	0.5	0.15	0.15	0.50	0.25	0.50	0.000	0.021	-0.003	-0.001
10	20	1	0.5	0.5	0.15	0.15	0.50	0.50	0.00	0.001	0.029	-0.028	0.002
10	20	1	0.5	0.5	0.15	0.15	0.50	0.50	0.25	0.001	0.028	-0.025	0.000
10	20	1	0.5	0.5	0.15	0.15	0.50	0.50	0.50	0.005	0.027	-0.031	0.002
10	20	1	0.5	0.5	0.30	0.30	0.00	0.00	0.00	0.001	0.030	0.014	-0.003
10	20	1	0.5	0.5	0.30	0.30	0.00	0.00	0.25	-0.006	0.024	0.018	0.001
10	20	1	0.5	0.5	0.30	0.30	0.00	0.00	0.50	0.004	0.027	0.036	0.000
10	20	1	0.5	0.5	0.30	0.30	0.00	0.25	0.00	0.002	0.022	-0.064	0.004
10	20	1	0.5	0.5	0.30	0.30	0.00	0.25	0.25	-0.001	0.025	-0.068	0.005
10	20	1	0.5	0.5	0.30	0.30	0.00	0.25	0.50	0.003	0.017	-0.053	0.005
10	20	1	0.5	0.5	0.30	0.30	0.00	0.50	0.00	0.004	0.026	-0.145	0.001
10	20	1	0.5	0.5	0.30	0.30	0.00	0.50	0.25	-0.008	0.031	-0.147	0.002
10	20	1	0.5	0.5	0.30	0.30	0.00	0.50	0.50	0.000	0.029	-0.146	0.003
10	20	1	0.5	0.5	0.30	0.30	0.25	0.00	0.00	-0.001	0.062	0.026	0.002
10	20	1	0.5	0.5	0.30	0.30	0.25	0.00	0.25	-0.003	0.067	0.015	0.006
10	20	1	0.5	0.5	0.30	0.30	0.25	0.00	0.50	-0.004	0.069	0.014	-0.003
10	20	1	0.5	0.5	0.30	0.30	0.25	0.25	0.00	-0.003	0.063	-0.057	-0.003
10	20	1	0.5	0.5	0.30	0.30	0.25	0.25	0.25	0.004	0.057	-0.053	-0.005
10	20	1	0.5	0.5	0.30	0.30	0.25	0.25	0.50	-0.004	0.062	-0.055	0.003
10	20	1	0.5	0.5	0.30	0.30	0.25	0.50	0.00	-0.003	0.065	-0.141	-0.005
10	20	1	0.5	0.5	0.30	0.30	0.25	0.50	0.25	0.003	0.062	-0.140	-0.001
10	20	1	0.5	0.5	0.30	0.30	0.25	0.50	0.50	-0.001	0.069	-0.149	0.005
10	20	1	0.5	0.5	0.30	0.30	0.50	0.00	0.00	0.000	0.098	0.035	0.004
10	20	1	0.5	0.5	0.30	0.30	0.50	0.00	0.25	0.001	0.108	0.018	-0.003
10	20	1	0.5	0.5	0.30	0.30	0.50	0.00	0.50	-0.002	0.101	0.018	-0.001

(continued)

N	m_1	$\frac{m_2}{m_1}$	$E(C)$, $E(R)$	$E(U)$, $E(A)$	σ_C, σ_R	σ_U, σ_A	ρ_{CR}	ρ_{CU}, ρ_{CA}	ρ_{RU}, ρ_{RA}	Mean Bias			
										c	r	u	a
10	20	1	0.5	0.5	0.30	0.30	0.50	0.25	0.00	0.002	0.101	-0.060	0.001
10	20	1	0.5	0.5	0.30	0.30	0.50	0.25	0.25	-0.005	0.109	-0.062	-0.003
10	20	1	0.5	0.5	0.30	0.30	0.50	0.25	0.50	-0.004	0.104	-0.063	-0.001
10	20	1	0.5	0.5	0.30	0.30	0.50	0.50	0.00	-0.001	0.113	-0.150	0.008
10	20	1	0.5	0.5	0.30	0.30	0.50	0.50	0.25	-0.011	0.117	-0.159	0.002
10	20	1	0.5	0.5	0.30	0.30	0.50	0.50	0.50	-0.009	0.112	-0.150	0.000
1000	4	1	0.2	0.5	0.00	0.00	0.00	0.00	0.00	0.001	0.000	0.001	0.000
1000	4	1	0.2	0.5	0.15	0.15	0.00	0.00	0.00	0.000	0.001	0.001	0.000
1000	4	1	0.2	0.5	0.15	0.15	0.00	0.00	0.25	-0.001	0.002	0.001	0.000
1000	4	1	0.2	0.5	0.15	0.15	0.00	0.00	0.50	0.000	0.000	0.002	0.000
1000	4	1	0.2	0.5	0.15	0.15	0.00	0.25	0.00	0.000	0.002	-0.013	0.000
1000	4	1	0.2	0.5	0.15	0.15	0.00	0.25	0.25	0.000	0.001	-0.014	0.000
1000	4	1	0.2	0.5	0.15	0.15	0.00	0.25	0.50	0.000	0.000	-0.012	0.000
1000	4	1	0.2	0.5	0.15	0.15	0.00	0.50	0.00	-0.002	0.003	-0.028	-0.001
1000	4	1	0.2	0.5	0.15	0.15	0.00	0.50	0.25	-0.001	0.001	-0.027	0.000
1000	4	1	0.2	0.5	0.15	0.15	0.00	0.50	0.50	-0.002	0.003	-0.029	0.000
1000	4	1	0.2	0.5	0.15	0.15	0.25	0.00	0.00	-0.001	0.028	-0.001	0.000
1000	4	1	0.2	0.5	0.15	0.15	0.25	0.00	0.25	0.001	0.025	0.002	0.000
1000	4	1	0.2	0.5	0.15	0.15	0.25	0.00	0.50	0.000	0.027	0.000	0.000
1000	4	1	0.2	0.5	0.15	0.15	0.25	0.25	0.00	0.000	0.028	-0.014	0.000
1000	4	1	0.2	0.5	0.15	0.15	0.25	0.25	0.25	0.000	0.027	-0.012	-0.001
1000	4	1	0.2	0.5	0.15	0.15	0.25	0.25	0.50	0.000	0.027	-0.013	0.000
1000	4	1	0.2	0.5	0.15	0.15	0.25	0.50	0.00	-0.001	0.029	-0.028	0.000
1000	4	1	0.2	0.5	0.15	0.15	0.25	0.50	0.25	-0.001	0.030	-0.028	0.000
1000	4	1	0.2	0.5	0.15	0.15	0.25	0.50	0.50	-0.001	0.029	-0.028	0.000
1000	4	1	0.2	0.5	0.15	0.15	0.50	0.00	0.00	0.000	0.055	0.000	0.000
1000	4	1	0.2	0.5	0.15	0.15	0.50	0.00	0.25	0.001	0.055	0.000	0.000
1000	4	1	0.2	0.5	0.15	0.15	0.50	0.00	0.50	0.000	0.054	0.001	0.000
1000	4	1	0.2	0.5	0.15	0.15	0.50	0.25	0.00	0.000	0.056	-0.013	0.000
1000	4	1	0.2	0.5	0.15	0.15	0.50	0.25	0.25	0.000	0.055	-0.013	0.000
1000	4	1	0.2	0.5	0.15	0.15	0.50	0.25	0.50	-0.001	0.057	-0.014	0.000
1000	4	1	0.2	0.5	0.15	0.15	0.50	0.50	0.00	-0.001	0.056	-0.027	0.000
1000	4	1	0.2	0.5	0.15	0.15	0.50	0.50	0.25	-0.001	0.057	-0.029	0.000
1000	4	1	0.2	0.5	0.15	0.15	0.50	0.50	0.50	-0.001	0.056	-0.028	0.000
1000	4	1	0.2	0.5	0.30	0.30	0.00	0.00	0.00	0.000	0.001	0.000	0.000
1000	4	1	0.2	0.5	0.30	0.30	0.00	0.00	0.25	0.000	0.001	0.001	0.000
1000	4	1	0.2	0.5	0.30	0.30	0.00	0.00	0.50	0.000	0.001	0.002	0.000
1000	4	1	0.2	0.5	0.30	0.30	0.00	0.25	0.00	-0.004	0.006	-0.051	0.000

(continued)

N	m_1	$\frac{m_2}{m_1}$	$E(C)$, $E(R)$	$E(U)$, $E(A)$	σ_C, σ_R	σ_U, σ_A	ρ_{CR}	ρ_{CU}, ρ_{CA}	ρ_{RU}, ρ_{RA}	Mean Bias c	r	u	a
1000	4	1	0.2	0.5	0.30	0.30	0.00	0.25	0.25	-0.004	0.006	-0.051	0.000
1000	4	1	0.2	0.5	0.30	0.30	0.00	0.25	0.50	-0.003	0.005	-0.049	0.000
1000	4	1	0.2	0.5	0.30	0.30	0.00	0.50	0.00	-0.017	0.020	-0.111	0.000
1000	4	1	0.2	0.5	0.30	0.30	0.00	0.50	0.25	-0.018	0.023	-0.112	-0.001
1000	4	1	0.2	0.5	0.30	0.30	0.00	0.50	0.50	-0.017	0.021	-0.112	0.000
1000	4	1	0.2	0.5	0.30	0.30	0.25	0.00	0.00	-0.001	0.088	0.000	0.000
1000	4	1	0.2	0.5	0.30	0.30	0.25	0.00	0.25	0.001	0.086	0.001	0.000
1000	4	1	0.2	0.5	0.30	0.30	0.25	0.00	0.50	0.000	0.086	-0.001	0.000
1000	4	1	0.2	0.5	0.30	0.30	0.25	0.25	0.00	-0.004	0.092	-0.050	0.000
1000	4	1	0.2	0.5	0.30	0.30	0.25	0.25	0.25	-0.004	0.093	-0.050	0.000
1000	4	1	0.2	0.5	0.30	0.30	0.25	0.25	0.50	-0.003	0.094	-0.051	0.000
1000	4	1	0.2	0.5	0.30	0.30	0.25	0.50	0.00	-0.017	0.114	-0.112	0.000
1000	4	1	0.2	0.5	0.30	0.30	0.25	0.50	0.25	-0.018	0.117	-0.111	0.000
1000	4	1	0.2	0.5	0.30	0.30	0.25	0.50	0.50	-0.018	0.115	-0.112	0.000
1000	4	1	0.2	0.5	0.30	0.30	0.50	0.00	0.00	0.000	0.186	0.001	0.000
1000	4	1	0.2	0.5	0.30	0.30	0.50	0.00	0.25	-0.001	0.187	0.001	-0.001
1000	4	1	0.2	0.5	0.30	0.30	0.50	0.00	0.50	0.000	0.185	0.000	0.000
1000	4	1	0.2	0.5	0.30	0.30	0.50	0.25	0.00	-0.004	0.192	-0.050	-0.001
1000	4	1	0.2	0.5	0.30	0.30	0.50	0.25	0.25	-0.004	0.191	-0.050	0.000
1000	4	1	0.2	0.5	0.30	0.30	0.50	0.25	0.50	-0.004	0.192	-0.051	0.000
1000	4	1	0.2	0.5	0.30	0.30	0.50	0.50	0.00	-0.019	0.227	-0.111	-0.001
1000	4	1	0.2	0.5	0.30	0.30	0.50	0.50	0.25	-0.018	0.223	-0.111	0.000
1000	4	1	0.2	0.5	0.30	0.30	0.50	0.50	0.50	-0.018	0.225	-0.112	0.000
1000	4	1	0.5	0.5	0.00	0.00	0.00	0.00	0.00	0.000	0.001	0.001	0.000
1000	4	1	0.5	0.5	0.15	0.15	0.00	0.00	0.00	0.001	0.000	0.002	0.000
1000	4	1	0.5	0.5	0.15	0.15	0.00	0.00	0.25	-0.001	0.001	0.000	0.000
1000	4	1	0.5	0.5	0.15	0.15	0.00	0.00	0.50	0.000	0.000	0.001	0.000
1000	4	1	0.5	0.5	0.15	0.15	0.00	0.25	0.00	0.000	0.001	-0.023	-0.001
1000	4	1	0.5	0.5	0.15	0.15	0.00	0.25	0.25	0.000	0.000	-0.022	0.000
1000	4	1	0.5	0.5	0.15	0.15	0.00	0.25	0.50	0.001	0.000	-0.021	0.001
1000	4	1	0.5	0.5	0.15	0.15	0.00	0.50	0.00	0.001	0.000	-0.043	0.000
1000	4	1	0.5	0.5	0.15	0.15	0.00	0.50	0.25	0.000	0.000	-0.044	0.000
1000	4	1	0.5	0.5	0.15	0.15	0.00	0.50	0.50	0.000	0.000	-0.045	0.000
1000	4	1	0.5	0.5	0.15	0.15	0.25	0.00	0.00	0.000	0.011	0.002	-0.001
1000	4	1	0.5	0.5	0.15	0.15	0.25	0.00	0.25	0.000	0.012	-0.001	0.000
1000	4	1	0.5	0.5	0.15	0.15	0.25	0.00	0.50	0.001	0.010	0.002	0.001
1000	4	1	0.5	0.5	0.15	0.15	0.25	0.25	0.00	0.000	0.011	-0.021	0.001
1000	4	1	0.5	0.5	0.15	0.15	0.25	0.25	0.25	0.000	0.011	-0.021	0.000

(continued)

N	m_1	$\frac{m_2}{m_1}$	$E(C)$, $E(R)$	$E(U)$, $E(A)$	σ_C, σ_R	σ_U, σ_A	ρ_{CR}	ρ_{CU}, ρ_{CA}	ρ_{RU}, ρ_{RA}	Mean Bias c	r	u	a
1000	4	1	0.5	0.5	0.15	0.15	0.25	0.25	0.50	0.000	0.011	-0.021	0.000
1000	4	1	0.5	0.5	0.15	0.15	0.25	0.50	0.00	0.000	0.012	-0.044	0.000
1000	4	1	0.5	0.5	0.15	0.15	0.25	0.50	0.25	0.000	0.011	-0.044	0.000
1000	4	1	0.5	0.5	0.15	0.15	0.25	0.50	0.50	0.000	0.011	-0.043	0.000
1000	4	1	0.5	0.5	0.15	0.15	0.50	0.00	0.00	0.000	0.023	0.001	0.000
1000	4	1	0.5	0.5	0.15	0.15	0.50	0.00	0.25	0.000	0.023	0.001	0.000
1000	4	1	0.5	0.5	0.15	0.15	0.50	0.00	0.50	0.000	0.023	0.000	0.000
1000	4	1	0.5	0.5	0.15	0.15	0.50	0.25	0.00	0.000	0.023	-0.022	0.000
1000	4	1	0.5	0.5	0.15	0.15	0.50	0.25	0.25	-0.001	0.023	-0.022	0.000
1000	4	1	0.5	0.5	0.15	0.15	0.50	0.25	0.50	0.000	0.022	-0.022	0.000
1000	4	1	0.5	0.5	0.15	0.15	0.50	0.50	0.00	-0.001	0.024	-0.045	0.000
1000	4	1	0.5	0.5	0.15	0.15	0.50	0.50	0.25	-0.001	0.023	-0.045	0.000
1000	4	1	0.5	0.5	0.15	0.15	0.50	0.50	0.50	0.000	0.023	-0.044	0.000
1000	4	1	0.5	0.5	0.30	0.30	0.00	0.00	0.00	0.000	0.000	0.002	0.000
1000	4	1	0.5	0.5	0.30	0.30	0.00	0.00	0.25	0.000	0.000	0.001	-0.001
1000	4	1	0.5	0.5	0.30	0.30	0.00	0.00	0.50	0.000	-0.001	0.000	0.000
1000	4	1	0.5	0.5	0.30	0.30	0.00	0.25	0.00	0.000	0.001	-0.085	0.000
1000	4	1	0.5	0.5	0.30	0.30	0.00	0.25	0.25	0.000	0.001	-0.086	0.000
1000	4	1	0.5	0.5	0.30	0.30	0.00	0.25	0.50	0.000	0.000	-0.084	0.001
1000	4	1	0.5	0.5	0.30	0.30	0.00	0.50	0.00	-0.001	0.001	-0.173	-0.001
1000	4	1	0.5	0.5	0.30	0.30	0.00	0.50	0.25	0.000	0.001	-0.172	0.001
1000	4	1	0.5	0.5	0.30	0.30	0.00	0.50	0.50	-0.001	0.000	-0.172	-0.001
1000	4	1	0.5	0.5	0.30	0.30	0.25	0.00	0.00	0.001	0.042	0.002	0.000
1000	4	1	0.5	0.5	0.30	0.30	0.25	0.00	0.25	0.000	0.043	0.001	0.000
1000	4	1	0.5	0.5	0.30	0.30	0.25	0.00	0.50	0.001	0.042	0.003	0.000
1000	4	1	0.5	0.5	0.30	0.30	0.25	0.25	0.00	0.000	0.042	-0.084	0.000
1000	4	1	0.5	0.5	0.30	0.30	0.25	0.25	0.25	0.000	0.043	-0.085	0.000
1000	4	1	0.5	0.5	0.30	0.30	0.25	0.25	0.50	0.000	0.044	-0.085	0.000
1000	4	1	0.5	0.5	0.30	0.30	0.25	0.50	0.00	0.000	0.043	-0.172	0.000
1000	4	1	0.5	0.5	0.30	0.30	0.25	0.50	0.25	-0.001	0.043	-0.172	0.000
1000	4	1	0.5	0.5	0.30	0.30	0.25	0.50	0.50	0.002	0.042	-0.170	0.000
1000	4	1	0.5	0.5	0.30	0.30	0.50	0.00	0.00	0.000	0.087	0.001	0.000
1000	4	1	0.5	0.5	0.30	0.30	0.50	0.00	0.25	0.000	0.087	0.001	0.000
1000	4	1	0.5	0.5	0.30	0.30	0.50	0.00	0.50	0.000	0.086	0.001	-0.001
1000	4	1	0.5	0.5	0.30	0.30	0.50	0.25	0.00	0.001	0.087	-0.084	0.000
1000	4	1	0.5	0.5	0.30	0.30	0.50	0.25	0.25	0.000	0.086	-0.084	0.000
1000	4	1	0.5	0.5	0.30	0.30	0.50	0.25	0.50	-0.001	0.088	-0.086	-0.001
1000	4	1	0.5	0.5	0.30	0.30	0.50	0.50	0.00	0.000	0.087	-0.173	0.000

(continued)

N	m_1	$\frac{m_2}{m_1}$	$E(C)$, $E(R)$	$E(U)$, $E(A)$	σ_C, σ_R	σ_U, σ_A	ρ_{CR}	ρ_{CU}, ρ_{CA}	ρ_{RU}, ρ_{RA}	Mean Bias c	r	u	a
1000	4	1	0.5	0.5	0.30	0.30	0.50	0.50	0.25	-0.001	0.087	-0.172	-0.001
1000	4	1	0.5	0.5	0.30	0.30	0.50	0.50	0.50	0.000	0.087	-0.173	0.000
1000	20	1	0.2	0.5	0.00	0.00	0.00	0.00	0.00	0.000	0.000	0.000	0.000
1000	20	1	0.2	0.5	0.15	0.15	0.00	0.00	0.00	0.000	0.000	0.000	0.000
1000	20	1	0.2	0.5	0.15	0.15	0.00	0.00	0.25	0.000	0.000	0.000	0.000
1000	20	1	0.2	0.5	0.15	0.15	0.00	0.00	0.50	0.000	0.000	0.000	0.000
1000	20	1	0.2	0.5	0.15	0.15	0.00	0.25	0.00	0.000	0.000	-0.013	0.001
1000	20	1	0.2	0.5	0.15	0.15	0.00	0.25	0.25	0.000	0.000	-0.013	0.000
1000	20	1	0.2	0.5	0.15	0.15	0.00	0.25	0.50	0.000	0.000	-0.013	0.000
1000	20	1	0.2	0.5	0.15	0.15	0.00	0.50	0.00	-0.002	0.002	-0.029	0.000
1000	20	1	0.2	0.5	0.15	0.15	0.00	0.50	0.25	-0.002	0.002	-0.029	0.000
1000	20	1	0.2	0.5	0.15	0.15	0.00	0.50	0.50	-0.002	0.002	-0.029	0.000
1000	20	1	0.2	0.5	0.15	0.15	0.25	0.00	0.00	-0.001	0.027	0.000	0.000
1000	20	1	0.2	0.5	0.15	0.15	0.25	0.00	0.25	0.000	0.027	0.000	0.000
1000	20	1	0.2	0.5	0.15	0.15	0.25	0.00	0.50	0.000	0.026	0.000	0.000
1000	20	1	0.2	0.5	0.15	0.15	0.25	0.25	0.00	0.000	0.027	-0.014	0.000
1000	20	1	0.2	0.5	0.15	0.15	0.25	0.25	0.25	0.000	0.027	-0.014	0.000
1000	20	1	0.2	0.5	0.15	0.15	0.25	0.25	0.50	0.000	0.027	-0.014	0.000
1000	20	1	0.2	0.5	0.15	0.15	0.25	0.50	0.00	-0.002	0.028	-0.028	0.000
1000	20	1	0.2	0.5	0.15	0.15	0.25	0.50	0.25	-0.001	0.027	-0.028	0.000
1000	20	1	0.2	0.5	0.15	0.15	0.25	0.50	0.50	-0.002	0.028	-0.028	0.000
1000	20	1	0.2	0.5	0.15	0.15	0.50	0.00	0.00	0.000	0.054	0.000	0.000
1000	20	1	0.2	0.5	0.15	0.15	0.50	0.00	0.25	0.000	0.054	0.000	0.000
1000	20	1	0.2	0.5	0.15	0.15	0.50	0.00	0.50	0.000	0.054	0.000	0.000
1000	20	1	0.2	0.5	0.15	0.15	0.50	0.25	0.00	0.000	0.054	-0.013	0.000
1000	20	1	0.2	0.5	0.15	0.15	0.50	0.25	0.25	0.000	0.054	-0.014	0.000
1000	20	1	0.2	0.5	0.15	0.15	0.50	0.25	0.50	0.000	0.054	-0.014	0.000
1000	20	1	0.2	0.5	0.15	0.15	0.50	0.50	0.00	-0.002	0.056	-0.029	0.000
1000	20	1	0.2	0.5	0.15	0.15	0.50	0.50	0.25	-0.002	0.056	-0.028	0.000
1000	20	1	0.2	0.5	0.15	0.15	0.50	0.50	0.50	-0.001	0.055	-0.028	0.000
1000	20	1	0.2	0.5	0.30	0.30	0.00	0.00	0.00	0.000	0.000	0.000	0.000
1000	20	1	0.2	0.5	0.30	0.30	0.00	0.00	0.25	0.000	0.001	0.000	0.000
1000	20	1	0.2	0.5	0.30	0.30	0.00	0.00	0.50	0.000	0.000	0.000	0.000
1000	20	1	0.2	0.5	0.30	0.30	0.00	0.25	0.00	-0.003	0.005	-0.050	0.000
1000	20	1	0.2	0.5	0.30	0.30	0.00	0.25	0.25	-0.003	0.003	-0.050	0.000
1000	20	1	0.2	0.5	0.30	0.30	0.00	0.25	0.50	-0.004	0.005	-0.050	0.000
1000	20	1	0.2	0.5	0.30	0.30	0.00	0.50	0.00	-0.017	0.021	-0.111	0.000
1000	20	1	0.2	0.5	0.30	0.30	0.00	0.50	0.25	-0.018	0.020	-0.112	0.000

(continued)

N	m_1	$\frac{m_2}{m_1}$	$E(C)$, $E(R)$	$E(U)$, $E(A)$	σ_C, σ_R	σ_U, σ_A	ρ_{CR}	ρ_{CU}, ρ_{CA}	ρ_{RU}, ρ_{RA}	Mean Bias			
										c	r	u	a
1000	20	1	0.2	0.5	0.30	0.30	0.00	0.50	0.50	-0.019	0.019	-0.112	0.000
1000	20	1	0.2	0.5	0.30	0.30	0.25	0.00	0.00	0.000	0.085	0.001	0.000
1000	20	1	0.2	0.5	0.30	0.30	0.25	0.00	0.25	0.000	0.086	0.001	0.000
1000	20	1	0.2	0.5	0.30	0.30	0.25	0.00	0.50	0.000	0.085	0.000	0.000
1000	20	1	0.2	0.5	0.30	0.30	0.25	0.25	0.00	-0.004	0.092	-0.051	0.000
1000	20	1	0.2	0.5	0.30	0.30	0.25	0.25	0.25	-0.003	0.091	-0.051	0.000
1000	20	1	0.2	0.5	0.30	0.30	0.25	0.25	0.50	-0.004	0.091	-0.051	0.000
1000	20	1	0.2	0.5	0.30	0.30	0.25	0.50	0.00	-0.018	0.115	-0.112	0.000
1000	20	1	0.2	0.5	0.30	0.30	0.25	0.50	0.25	-0.018	0.113	-0.112	0.000
1000	20	1	0.2	0.5	0.30	0.30	0.25	0.50	0.50	-0.017	0.114	-0.111	0.000
1000	20	1	0.2	0.5	0.30	0.30	0.50	0.00	0.00	0.000	0.184	0.001	0.000
1000	20	1	0.2	0.5	0.30	0.30	0.50	0.00	0.25	0.000	0.183	0.001	-0.001
1000	20	1	0.2	0.5	0.30	0.30	0.50	0.00	0.50	0.000	0.183	0.000	0.001
1000	20	1	0.2	0.5	0.30	0.30	0.50	0.25	0.00	-0.004	0.191	-0.051	0.000
1000	20	1	0.2	0.5	0.30	0.30	0.50	0.25	0.25	-0.004	0.191	-0.050	0.000
1000	20	1	0.2	0.5	0.30	0.30	0.50	0.25	0.50	-0.004	0.192	-0.051	0.000
1000	20	1	0.2	0.5	0.30	0.30	0.50	0.50	0.00	-0.018	0.221	-0.111	0.000
1000	20	1	0.2	0.5	0.30	0.30	0.50	0.50	0.25	-0.017	0.220	-0.112	0.000
1000	20	1	0.2	0.5	0.30	0.30	0.50	0.50	0.50	-0.018	0.222	-0.112	0.000
1000	20	1	0.5	0.5	0.00	0.00	0.00	0.00	0.00	0.000	0.000	0.000	0.000
1000	20	1	0.5	0.5	0.15	0.15	0.00	0.00	0.00	0.000	0.000	0.000	0.000
1000	20	1	0.5	0.5	0.15	0.15	0.00	0.00	0.25	0.000	0.000	0.000	0.000
1000	20	1	0.5	0.5	0.15	0.15	0.00	0.00	0.50	0.000	-0.001	0.001	0.000
1000	20	1	0.5	0.5	0.15	0.15	0.00	0.25	0.00	0.000	0.000	-0.022	0.000
1000	20	1	0.5	0.5	0.15	0.15	0.00	0.25	0.25	0.000	0.000	-0.022	0.000
1000	20	1	0.5	0.5	0.15	0.15	0.00	0.25	0.50	0.000	0.000	-0.022	0.000
1000	20	1	0.5	0.5	0.15	0.15	0.00	0.50	0.00	0.000	0.000	-0.045	0.000
1000	20	1	0.5	0.5	0.15	0.15	0.00	0.50	0.25	-0.001	0.000	-0.045	0.000
1000	20	1	0.5	0.5	0.15	0.15	0.00	0.50	0.50	0.000	0.000	-0.045	0.000
1000	20	1	0.5	0.5	0.15	0.15	0.25	0.00	0.00	0.000	0.011	0.001	0.000
1000	20	1	0.5	0.5	0.15	0.15	0.25	0.00	0.25	0.000	0.011	0.000	0.000
1000	20	1	0.5	0.5	0.15	0.15	0.25	0.00	0.50	0.000	0.011	0.000	0.000
1000	20	1	0.5	0.5	0.15	0.15	0.25	0.25	0.00	0.000	0.011	-0.022	0.000
1000	20	1	0.5	0.5	0.15	0.15	0.25	0.25	0.25	0.000	0.011	-0.022	0.000
1000	20	1	0.5	0.5	0.15	0.15	0.25	0.25	0.50	0.000	0.011	-0.022	0.000
1000	20	1	0.5	0.5	0.15	0.15	0.25	0.50	0.00	0.000	0.012	-0.045	0.000
1000	20	1	0.5	0.5	0.15	0.15	0.25	0.50	0.25	0.000	0.012	-0.045	0.000
1000	20	1	0.5	0.5	0.15	0.15	0.25	0.50	0.50	-0.001	0.011	-0.045	0.000

(continued)

N	m_1	$\frac{m_2}{m_1}$	$E(C)$, $E(R)$	$E(U)$, $E(A)$	σ_C, σ_R	σ_U, σ_A	ρ_{CR}	ρ_{CU}, ρ_{CA}	ρ_{RU}, ρ_{RA}	Mean Bias c	Mean Bias r	Mean Bias u	Mean Bias a
1000	20	1	0.5	0.5	0.15	0.15	0.50	0.00	0.00	0.000	0.023	-0.001	0.000
1000	20	1	0.5	0.5	0.15	0.15	0.50	0.00	0.25	0.000	0.022	0.000	0.000
1000	20	1	0.5	0.5	0.15	0.15	0.50	0.00	0.50	0.000	0.022	0.000	0.000
1000	20	1	0.5	0.5	0.15	0.15	0.50	0.25	0.00	0.000	0.023	-0.022	0.000
1000	20	1	0.5	0.5	0.15	0.15	0.50	0.25	0.25	0.000	0.023	-0.022	0.000
1000	20	1	0.5	0.5	0.15	0.15	0.50	0.25	0.50	0.000	0.022	-0.022	0.000
1000	20	1	0.5	0.5	0.15	0.15	0.50	0.50	0.00	0.000	0.022	-0.044	0.000
1000	20	1	0.5	0.5	0.15	0.15	0.50	0.50	0.25	0.000	0.022	-0.044	0.000
1000	20	1	0.5	0.5	0.15	0.15	0.50	0.50	0.50	0.000	0.022	-0.044	0.000
1000	20	1	0.5	0.5	0.30	0.30	0.00	0.00	0.00	0.001	0.000	0.000	0.000
1000	20	1	0.5	0.5	0.30	0.30	0.00	0.00	0.25	0.000	0.000	-0.001	0.000
1000	20	1	0.5	0.5	0.30	0.30	0.00	0.00	0.50	0.000	0.000	0.000	0.000
1000	20	1	0.5	0.5	0.30	0.30	0.00	0.25	0.00	0.000	0.000	-0.085	0.000
1000	20	1	0.5	0.5	0.30	0.30	0.00	0.25	0.25	0.000	0.000	-0.085	0.000
1000	20	1	0.5	0.5	0.30	0.30	0.00	0.25	0.50	0.000	0.000	-0.085	0.000
1000	20	1	0.5	0.5	0.30	0.30	0.00	0.50	0.00	0.000	0.000	-0.173	0.000
1000	20	1	0.5	0.5	0.30	0.30	0.00	0.50	0.25	0.000	0.000	-0.172	-0.001
1000	20	1	0.5	0.5	0.30	0.30	0.00	0.50	0.50	0.000	0.000	-0.173	0.000
1000	20	1	0.5	0.5	0.30	0.30	0.25	0.00	0.00	0.000	0.043	0.002	0.000
1000	20	1	0.5	0.5	0.30	0.30	0.25	0.00	0.25	0.000	0.043	-0.001	0.000
1000	20	1	0.5	0.5	0.30	0.30	0.25	0.00	0.50	0.000	0.044	0.001	0.000
1000	20	1	0.5	0.5	0.30	0.30	0.25	0.25	0.00	0.000	0.043	-0.085	0.000
1000	20	1	0.5	0.5	0.30	0.30	0.25	0.25	0.25	0.000	0.042	-0.085	0.000
1000	20	1	0.5	0.5	0.30	0.30	0.25	0.25	0.50	0.001	0.043	-0.084	0.000
1000	20	1	0.5	0.5	0.30	0.30	0.25	0.50	0.00	0.000	0.043	-0.173	0.000
1000	20	1	0.5	0.5	0.30	0.30	0.25	0.50	0.25	0.000	0.044	-0.173	0.000
1000	20	1	0.5	0.5	0.30	0.30	0.25	0.50	0.50	0.000	0.043	-0.172	0.000
1000	20	1	0.5	0.5	0.30	0.30	0.50	0.00	0.00	0.000	0.086	0.000	0.001
1000	20	1	0.5	0.5	0.30	0.30	0.50	0.00	0.25	0.001	0.087	0.001	0.000
1000	20	1	0.5	0.5	0.30	0.30	0.50	0.00	0.50	-0.001	0.087	-0.001	0.000
1000	20	1	0.5	0.5	0.30	0.30	0.50	0.25	0.00	-0.001	0.087	-0.085	0.000
1000	20	1	0.5	0.5	0.30	0.30	0.50	0.25	0.25	0.001	0.087	-0.084	0.000
1000	20	1	0.5	0.5	0.30	0.30	0.50	0.25	0.50	0.000	0.087	-0.085	0.000
1000	20	1	0.5	0.5	0.30	0.30	0.50	0.50	0.00	0.000	0.087	-0.173	0.000
1000	20	1	0.5	0.5	0.30	0.30	0.50	0.50	0.25	-0.001	0.087	-0.173	-0.001
1000	20	1	0.5	0.5	0.30	0.30	0.50	0.50	0.50	0.000	0.086	-0.172	0.000

Note. N = Number of simulated participants; m_1 = number of word pairs; m_2/m_1 = ratio of singletons to word pairs; $E(C), E(R), E(U), E(A)$ = expected values of model parameters; $\sigma_C, \sigma_R, \sigma_U, \sigma_A$ = standard deviation of model parameters; $\rho_{CR}, \rho_{CU}, \rho_{RU}, \rho_{CA}, \rho_{RA}$ = true correlation between parameters.
